# The evolution of isochore patterns in vertebrate genomes

**DOI:** 10.1186/1471-2164-10-146

**Published:** 2009-04-03

**Authors:** Maria Costantini, Rosalia Cammarano, Giorgio Bernardi

**Affiliations:** 1 Stazione Zoologica Anton Dohrn, Naples, Italy

## Abstract

**Background:**

Previous work from our laboratory showed that (i) vertebrate genomes are mosaics of isochores, typically megabase-size DNA segments that are fairly homogeneous in base composition; (ii) isochores belong to a small number of families (five in the human genome) characterized by different GC levels; (iii) isochore family patterns are different in fishes/amphibians and mammals/birds, the latter showing GC-rich isochore families that are absent or very scarce in the former; (iv) there are two modes of genome evolution, a conservative one in which isochore patterns basically do not change (e.g., among mammalian orders), and a transitional one, in which they do change (e.g., between amphibians and mammals); and (v) isochores are tightly linked to a number of basic biological properties, such as gene density, gene expression, replication timing and recombination.

**Results:**

The present availability of a number of fully sequenced genomes ranging from fishes to mammals allowed us to carry out investigations that (i) more precisely quantified our previous conclusions; (ii) showed that the different isochore families of vertebrate genomes are largely conserved in GC levels and dinucleotide frequencies, as well as in isochore size; and (iii) isochore family patterns can be either conserved or change within both warm- and cold-blooded vertebrates.

**Conclusion:**

On the basis of the results presented, we propose that (i) the large conservation of GC levels and dinucleotide frequencies may reflect the conservation of chromatin structures; (ii) the conservation of isochore size may be linked to the role played by isochores in chromosome structure and replication; (iii) the formation, the maintainance and the changes of isochore patterns are due to natural selection.

## Background

Investigations carried out in our laboratory over many years led to a general picture of the organization of the vertebrate genome and its evolution. We recall here very briefly that the vertebrate genome is a mosaic of isochores, typically megabase-size DNA segments that belong in a small number of families characterized by different GC levels, and that are tightly associated with basic genome properties such as gene density, gene expression, replication timing and recombination (see refs. [1,2] for reviews). Remarkably, the isochore family patterns (hencefrom indicated as isochore patterns) of mammals and birds were found to be strikingly different from those of amphibians and fishes. Most of our previous results were obtained through a compositional approach that mainly involved (i) DNA fractionation using ultracentrifugation in density gradients in the presence of sequence-specific ligands; (ii) cytogenetic analyses; and (iii) gene and genome sequences, when this became possible.

The recent availability of a number of fully sequenced vertebrate genomes allowed us to quantify very precisely our previous results. This approach was started by scanning GC levels [3] in the first fully sequenced vertebrate genome, the human genome (see Materials and Methods for details and comments on segmentation approaches). The number-average size of compositionally fairly homogeneous regions, the isochores, was found to be 0.9 Mb (megabases), the weight-average 1.9 Mb (the number-average is the classical average of all size values; the weight-average is the average of values multiplied by their amounts). When isochores were pooled in bins of 1% GC, their distribution confirmed that they belonged in the five families that had been previously described (L1, L2, H1, H2 and H3, in order of increasing GC) [4].

The euchromatic regions of human chromosomes were completely covered by 3200 isochores that formed the ultimate chromosomal bands [5]. In contrast, the genomes of the cold-blooded vertebrates (fishes) explored so far at the sequence level expectedly showed a much lower compositional heterogeneity and were characterized by less complex isochore patterns [6]. We used here, as in previous papers [4], the old-fashioned distinction between cold- and warm-blooded vertebrates in order to stress the link that we proposed to exist between genome structure (and thermodynamic stability) and body temperature (whatever its origin, homeothermy, behavioural regulation, environmental temperature), a point clearly made in our previous work (see ref. [1] for a review).

In the present work we analyzed at the sequence level the genomes of Eutherians not yet explored by us, namely chimpanzee (*Pan troglodytes*), mouse (*Mus musculus*) and dog (*Canis familiaris*), a Marsupial, the opossum (*Monodelphis domestica*) and a Monotreme, the platypus (*Ornithorhynchus anatinus*). Some comparative data from a Reptile (*Anolis carolinensis*) and an Amphibian (*Xenopus tropicalis*) are also presented, even if these genome sequences are only available as scaffolds. Since we had previously investigated the fully sequenced human, fish and chicken genomes [3,6,7], we could approach here the general problem of the organization and evolution of vertebrate genomes at the sequence level.

## Results

### The isochore families: the patterns

When isochores from vertebrate genomes are pooled in bins of 1% GC [3], or 0.5% GC as in the present work, and plotted against their GC levels, isochore families appear, as expected from previous investigations. A comparison of the isochore patterns of vertebrate genomes involves the assessment of the relative amounts of the isochore families and of their average GC levels. These are reported below.

**Figure 1:**
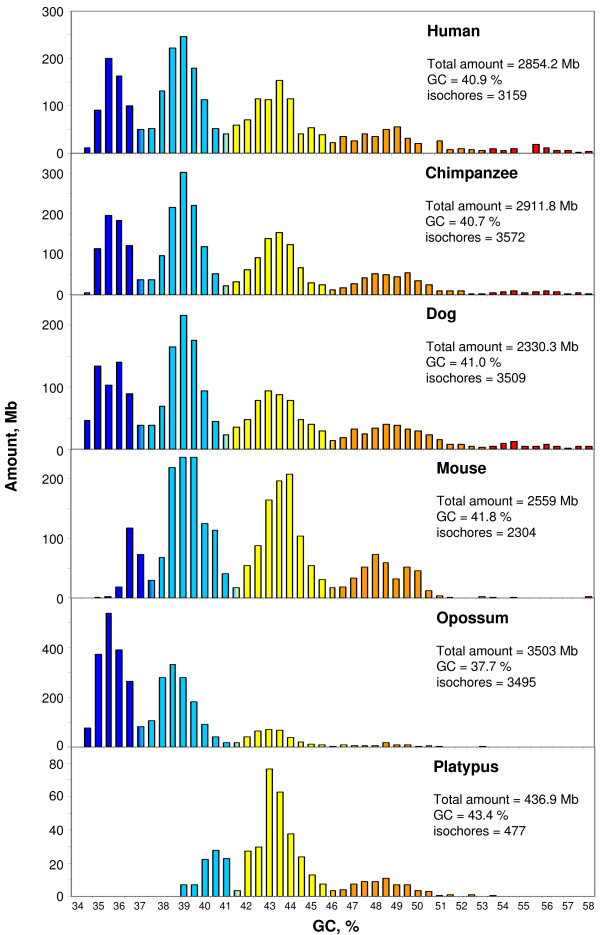
**Distribution of isochores according to GC levels**. The histograms show the distribution (by weight; see Text) of isochores as pooled in bins of 0.5% GC from chimpanzee, dog, mouse, opossum and platypus. Total amounts of sequences are calculated from the sums of isochores; colors represent the five isochore families. Values at minima were split between the two neighbouring families (histogram bars with mixed colors). A comparable plot for the human genome [3] is reported for the sake of comparison.

As expected, two Primates (human and chimpanzee) and a Carnivore (dog) showed a large similarity in the relative amounts of the isochore families, whereas in mouse L1 isochores were poorly represented and H3 isochores were essentially absent (Figure 1 and Table 1).

**Table 1:** Relative amounts, average GC and average size of isochore families from vertebrates

**Relative amount, %**	**L1**	**L2**	**H1**	**H2**	**H3**
					
**Human**	19.0	37.0	31.0	11.0	3.0
**Chimp**	22.6	36.6	25.4	13.2	2.1
**Dog**	23.8	35.5	23.7	13.4	3.6
					
**Overall average**	**21.8**	**36.4**	**26.7**	**12.5**	**2.9**
**Standard deviation**	**2.5**	**0.8**	**3.8**	**1.3**	**0.8**
					
**Chicken**	17.4	38.5	30.3	12.0	(1.6)^a^
					
**Overall average**	**20.7**	**36.9**	**27.6**	**12.4**	**2.9**
**Standard deviation**	**3.0**	**1.2**	**3.6**	**1.1**	**0.8**
					
**Mouse**	9.7	40.3	35.5	14.2	0.2
**Opossum**	49.2	37.9	10.0	2.3	
**Platypus**	---	14.5	71.8	15.1	0.14
**Zebrafish**	75.7	23.3	---	---	---
**Medaka**	---	71.0	23.7	---	---
**Stickleback**	---	---	77.5	21.0	---
**Pufferfish**	---	---	55.3	37.8	---
					
					
**Average GC**					
					
**Human**	36.0	38.9	43.1	48.7	54.5
**Chimp**	36.0	38.9	43.2	48.6	55.0
**Dog**	35.9	38.9	43.2	48.7	55.8
**Mouse^(b)^**	36.5	39.4	43.6	48.1	54.4
**Platypus**	---	40.0	42.9	47.9	54.9
**Opossum**	36.0	38.5	42.9	48.6	55.6
**Chicken**	36.6	39.3	43.4	48.8	54.7
**Zebrafish**	36.0	38.2	---	---	---
**Medaka**	---	39.9	42.3	---	---
**Stickleback**	---	---	44.2	47.3	---
**Pufferfish**	---	---	44.4	48.2	54.7
					
**Overall average**	**36.2**	**39.1**	**43.3**	**48.3**	**54.8**
**Standard deviation**	**0.3**	**0.6**	**0.7**	**0.5**	**0.5**
					
					
**Average size (Mb)**					
					
**Human**	0.90	0.90	0.80	0.70	0.70
**Chimp**	(1.4)^b^	0.80	0.60	0.70	0.60
**Dog**	1.02	0.69	0.53	0.55	0.59
**Mouse**	1.09	1.40	0.99	0.97	0.30
**Opossum**	(1.6)^b^	0.80	0.60	0.50	0.40
**Platypus**	---	0.90	1.10	0.50	---
**Chicken**	0.70	0.81	0.60	0.55	(0.34)^(a)^
**Zebrafish**	(1.9)^b^	0.60	---	---	---
**Medaka**	---	(2.8)^b^	0.90	---	---
**Stickleback**	---	---	(2.1)^b^	0.70	---
**Pufferfish**	---	---	0.90	0.70	---

^(a) ^These values concern only a small part of H3 (for more details see ref. 7)

They were not included in calculating the standard deviation in A.

^(b) ^Values in parenthesis are likely to be at least in part artefactually large.

In opossum, L1 isochores were much more represented than in Eutherians and GC-rich isochores H2 and H3 were very scarce (Figure 1 and Table 1). This pattern might be due to interspersed repeats that represent about 50% of this genome. This possibility was disproven, however, by two findings: (i) repeats were distributed over all isochore families with only a slightly higher concentration in GC-poor families; and (ii) the base composition of repeats was quite close to those of "unique" contiguous sequences (see Additional file 1).

In contrast to the genomes of Eutherians and chicken (see below), which showed an average GC level of about 41%, and to the GC-poorer genome of opossum (~38% GC), the platypus genome, which has a size of approximately 2.4 Gb (only 18% of which are assembled, the remaining sequences being available as supercontigs) showed a high GC level of 43.4%. This genome essentially consisted of L2 and H1 isochores with a small amount of H2 isochores (Figure 1 and Table 1), a result due in part to the missing assembly of GC-rich microchromosomes. Indeed, when the GC profile of the unassembled sequences was superimposed on the isochore profile of the platypus genome (see Additional file 2), it became clear that the unassembled parts essentially corresponded to GC-rich chromosomal regions (as in the case of chicken; see below).

**Figure 2:**
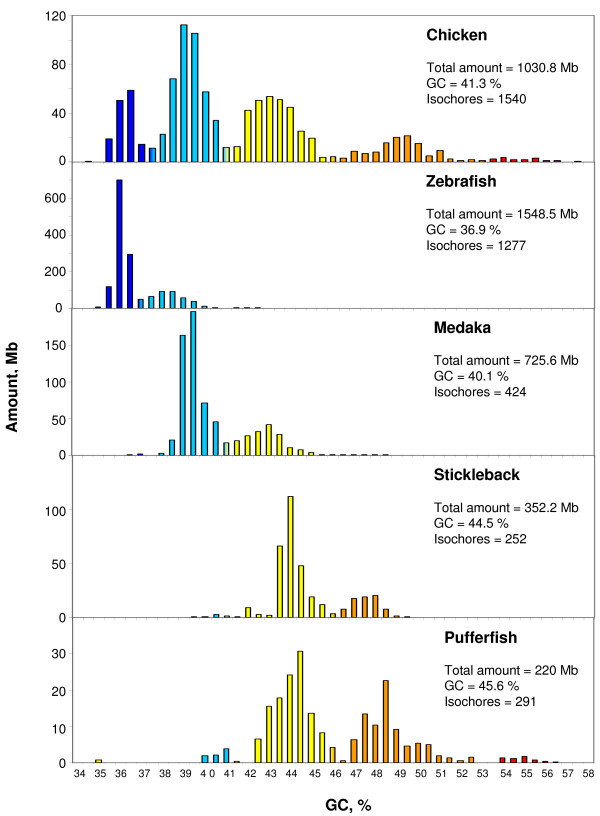
**Distribution of isochores according to GC levels**. The histograms show the distribution (by weight; see Text) of isochores as pooled in bins of 0.5% GC from fish [6] and chicken isochores [7]. See also legend of Figure 1.

In the chicken genome (Figure 2 and Table 1), which has a size about 1/3 of the human genome, all isochore families were very slightly shifted toward GC-rich values compared to the human distribution. Moreover, L1 isochores were underrepresented in the genome and a GC-richest H4 isochore family was present, even if in very small amounts in the currently available assembly. This data still lacks some microchromosomes, all of which are known to be very GC-rich [8].

The isochore families of the fully sequenced fish genomes (Figure 2 and Table 1) were already described [6]. In each of the four genome sequences only two isochore families were present (L1 and L2 in the case of zebrafish), or predominant (L2 and H1 in medaka, H1 and H2 in stickleback and pufferfish, H2 being more represented in the latter case).

**Figure 3:**
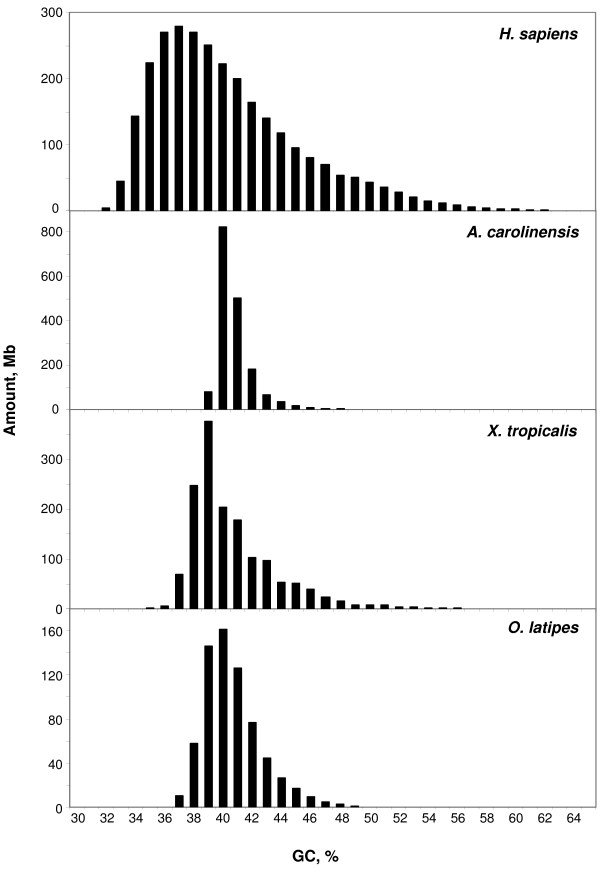
**The amounts of DNA in human and medaka chromosomes, as well as in scaffolds of anolis and xenopus were partitioned into non-overlapping 100 kb windows and pooled in 1% GC bins**.

Unfortunately, only scaffolds were available for the genomes of a reptile (*Anolis carolinensis*) and of an amphibian (*Xenopus tropicalis*). When-100 kb segments of these scaffolds were binned and compared with similar histograms for the human and medaka (*Oryzias latipes*) genomes, the compositional heterogeneities of the anolis and xenopus genomes were found to be much lower than that of the human genome and rather close to that of the genome of medaka, the compositionally closest fish genome (Figure 3).

### The isochore families: the GC levels and the dinucleotide frequencies

**Figure 4:**
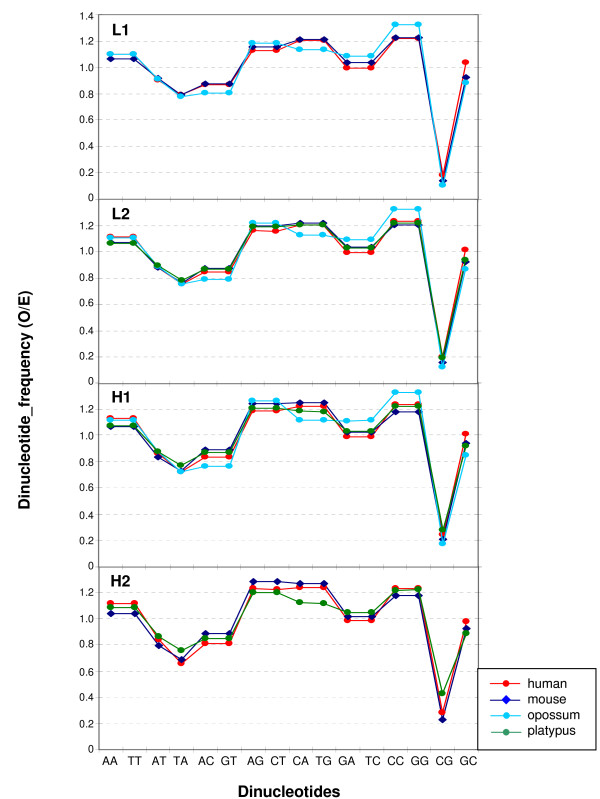
**Comparison of dinucleotide frequencies in eutherian genomes**. Observed/expected frequencies for dinucleotides in 100-kb DNA segments in the isochore families from human, mouse, opossum and platypus. The lines between points are only used to make an easier comparison of the values from each genome.

**Figure 5:**
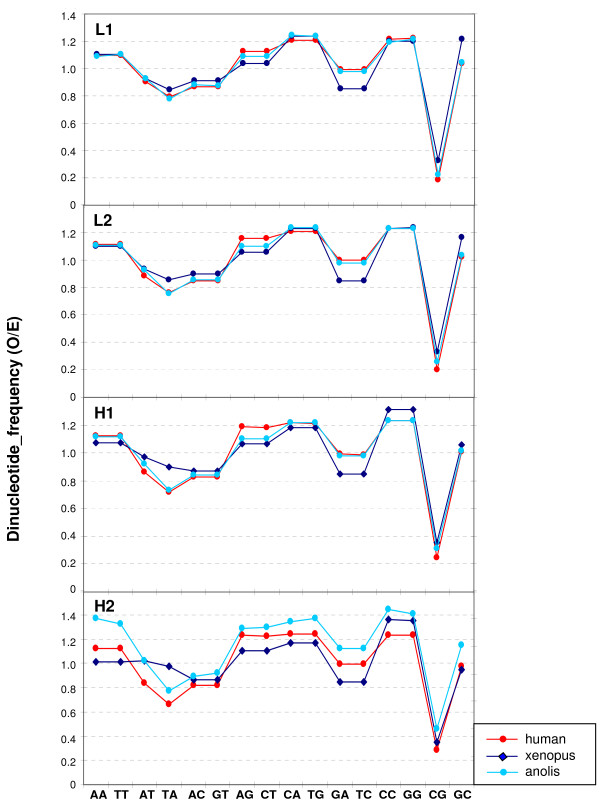
**Comparison of dinucleotide frequencies of human, reptiles and amphibians**. See also legend of Figure 4.

**Figure 6:**
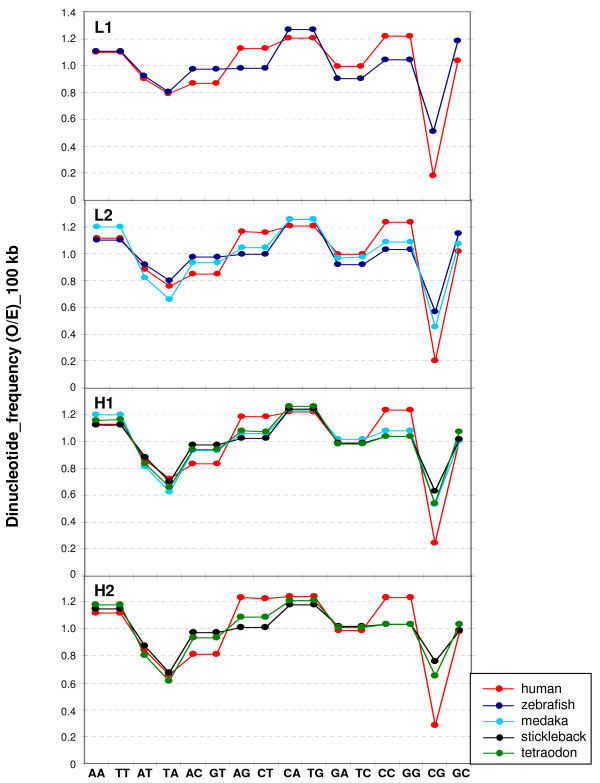
**Comparison of dinucleotide frequencies of human and fishes**. See also legend of Figure 4.

In spite of the different relative amounts of isochore families found within and among vertebrate classes (but practically not between Eutherians and chicken), the average GC levels of isochores belonging to the different families were remarkably conserved (see Figures 1 and 2 and Table 1). Because of their possible functional relevance in connection with chromatin structure [9], dinucleotide frequencies were also assessed as observed/expected ratios in different isochore families. These ratios were extremely close between human, mouse, opossum and platypus in each of the isochore families (Figure 4), whereas the ratios of anolis and xenopus (Figure 5) diverged slightly from the ratios seen in mammals. In the human/fish comparison (Figure 6), practically no differences were found in AA, TT, AT and TA, but CpG was higher in fish DNA than in human, as expected from the lower body temperature of fishes [10].

### The isochore sizes

**Figure 7:**
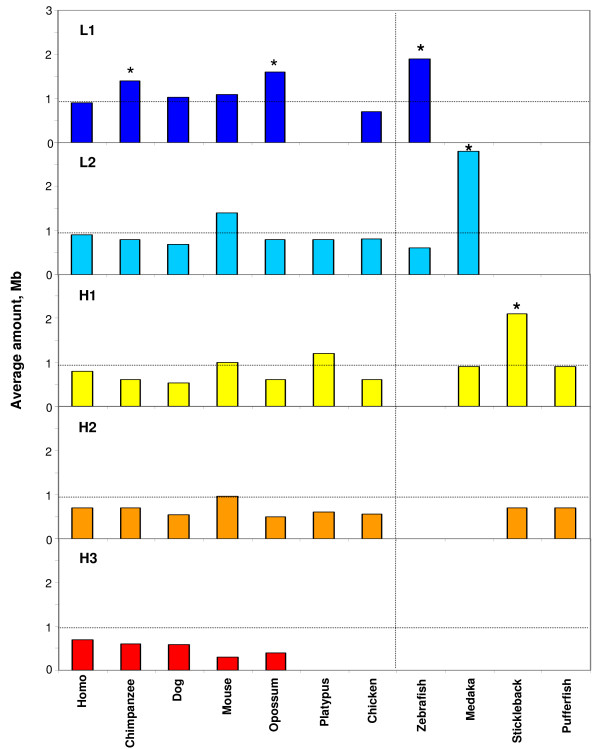
**Average size of isochores belonging in the five isochore families for all the vertebrates tested**. The data for human, fishes [6] and chicken [7] are reported for the sake of comparison. A horizontal guideline at 0.9 Mb correspond to the average size of isochores in the human genome [3]. A vertical line is drawn to divide mammals and chicken from the fishes. Asterisks refer to sizes that are probably overestimated (see Text and Table 1).

The average size of isochores in the different families showed a remarkable conservation in all vertebrates, from fish to human, again in spite of the differences in the relative amounts of isochore families (see Figure 7 and Table 1). This stability of isochore size within isochore families was accompanied, however, by systematic differences between isochore families, in particular (i) a larger size (>1 Mb) and a larger spread of the GC-poorest isochore families (L1 in zebrafish and mammals, except for mouse, L2 in medaka and H1 in stickleback; see Additional file 3 and Discussion); (ii) a smaller size (<1 Mb) and a narrower size distribution of the GC-rich isochores; and (iii) a regular decrease from L1 to H3 isochore families.

Additional Files 10, 11, 12, 13 and 14 (Tables T1–T5) present the coordinates, sizes, GC levels and differences in GC between isochores for the genomes of chimpanzee, dog, mouse, opossum and platypus. Additional Files 4, 5, 6, 7 and 8 display the corresponding GC profiles.

### Gene densities

**Figure 8:**
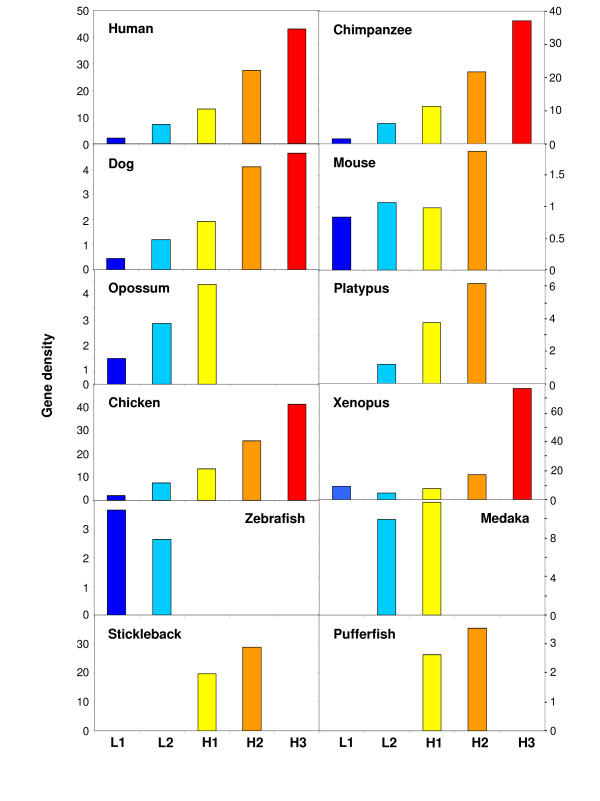
**Gene density**. The histograms represent the gene density, as density of available genes per megabase, in the five isochore families (L1, L2, H1, H2 and H3) for chimpanzee, dog, mouse, opossum and platypus; the data published on human, chicken [7] and fishes [6] are reported for the sake of comparison. The gene density for xenopus is calculated on scaffolds partitioned into non-overlapping 100 kb windows, according to the borders of human isochore families.

The gene densities of all isochore families (Figure 8) showed an increase with increasing GC in both warm- and cold-blooded vertebrates, as expected from previous results (see ref. [1] for a review). In the case of xenopus, genes were localized on the scaffolds and gene densities were shown to follow the general trend of all vertebrates (see Figure 8). The only exception to this general rule was that of zebrafish, in which case the most represented L1 family had a slightly higher gene density compared to L2 isochores. The reasons for such a situation are under current investigation. Gene density was not calculated in the case of anolis because the coordinates of genes on the scaffolds are not yet available.

## Discussion

### The two modes of genome evolution: the transitional mode

It should be recalled that a transitional (or shifting) mode in genome evolution was originally indicated by the gaussian analysis of buoyant density profiles of DNA (and DNA fractions) from cold- and warm-blooded vertebrates [11]. In this mode, large changes occurr in isochore patterns. More specifically, GC-rich isochores were found to be absent or scarce in the carp and xenopus genome, respectively, compared to the genomes of human, mouse and chicken [4], and similar differences were seen in orthologous genes [12]. Interestingly, the transitional mode of the genome evolution could also be observed at the cytogenetic level because the compositional heterogeneity of genomes is reflected in the chromosomal banding patterns (see ref [1] for a review). These findings indicate the existence of correlations linking compositional heterogeneity, chromatin structure and banding patterns. The transitional mode of evolution could now be checked on several vertebrate genomes at the sequence level with a much higher degree of precision.

The compositional differences of the genomes of human and xenopus were originally attributed to the different body temperature of warm- and cold-blooded vertebrates [13]. This "thermodynamic stability hypothesis" accounted for the higher GC level of DNA and RNA. Moreover, it was noted that GC-rich codons preferentially encode aminoacids that confer thermal stability to the corresponding proteins. This latter point was recently confirmed by showing [9] that, out of 18,795 human genes, those located in GC-rich isochores have an increased level of GC-rich codons leading to higher levels of stabilizing aminoacids (such as arginine and alanine) and lower levels of destabilizing aminoacids (such as lysine, isoleucine and asparagine). Expectedly, the opposite was found in genes located in GC-poor isochores.

The thermodynamic stability hypothesis is now supported by several new findings (i) the isochore patterns of anolis, xenopus and fishes (except for pufferfish; but see (iv) below) lack the GC-richest isochores present in the human pattern; (ii) the predominant GC-poor isochores of opossum might be related, at least in part, to the lower body temperature (32°C) of this marsupial; (iii) the small shift to the GC-rich side of the isochore distribution of chicken and the presence of a small GC-richest H4 isochore family might be related to the higher body temperature (41° – 43.5°C) [14] of birds compared to mammals; (iv) the shift to the GC-rich side of the tetraodon genome, a fish living in tropical freshwater contrasts with the isochore pattern of fugu, a fish (from the same family) living at a lower temperature in the Pacific Ocean [14] (see also Additional File 9 and Supplementary Figure S6 from ref. [6]); (v) the isochore patterns of reptiles, a class of vertebrates known to be characterized by different body temperatures and different thermal regulations cover a broad spectrum; indeed, genomes may either be even more compositionally homogeneous than the xenopus genome (e.g., the anolis genome; see Figure 3), or show the presence of GC-rich isochores (as in the case of *Testudo graeca *and *Crocodylus niloticus *[15]); the latter point was recently confirmed by comparing GC_3 _(the GC level of third codon position) of orthologous genes from *Alligator mississippiensis*, human and chicken [16]; (vi) both mammals and birds, two classes of vertebrates derived at different times from different ancestral reptiles (Therapsids about 220 Mya and Dinosaurs, about 150 Million years ago, respectively, [17]), showed the formation of the same families of GC-rich isochores (compare the human and the chicken patterns of Figures 1 and 2), a clear indication of a convergent compositional evolution; likewise, a convergent evolution may be the explanation for the similarity of GC_3 _values of orthologous genes from alligator and chicken; indeed, there is no compelling reason to consider common descent from archosaurs as the explanation [16], given the large phylogenetic distance [18,19], the complex endo-ectothermic evolution of crocodiles [20], and the contrasting data on the cold- or warm-bloodedness of the immediate ancestors of birds, dinosaurs; (vii) the excess of AT → GC over GC → AT changes observed in the genes of *Gillichthys seta*, a fish living at 40°C, compared to the orthologous genes of *Gillicthys mirabilis*, a congeneric fish living at 20°C [21]; interestingly, the former one was characterized by positive selection on some genes and by an expansion of a GC-rich minisatellites in gene-rich regions.

The explanation why only the gene-rich regions of the genome and not the whole genomes underwent a GC increase was provided by the finding that those regions have an open chromatin structure ([22], as also shown by accessibility to DNAse I [23], and to apoptotic and MNase degradation [24]), whereas the gene-poor regions could be stabilized by their own compact chromatin. This point is supported by the finding that, when the body temperature change is very rapid, as in the case of the divergence of *G. seta *from *G. mirabilis *(<0.66–0.75 Million years ago), the gene-rich regions of the genome are stabilized by the regional expansion of a very GC-rich minisatellite (see above).

Since the thermal stability hypothesis is based on general physical-chemical properties, it would be expected to be valid very widely. This is, indeed, the case as shown by the correlation of GC levels of paired sequences (stems) of ribosomal 18S RNAs with body temperature for vertebrates ranging from mammals to polar fishes (differences being seen even between eutherians, 37°C body temperature, and both marsupials and monotremes, 32°C body temperature, [25]) and by the correlation of GC levels and optimal growth temperatures of prokaryotes [26].

While body temperature seems to be a major determinant of the compositional properties of genome, other factors may also play a role. This is clearly indicated by the different isochore patterns of fishes. In this case not only temperature, but other environmental factors such as salinity, oxygen level, pH etc. are possibly involved. The compositional differences found between eutherians and monotremes that have different body temperatures (37° vs 32°) require further investigations to be understood. We know, however, that CpG and 5 mC values of monotremes are intermediate between the low values of eutherians and the high values of fishes [[1,27] and present work] (see Figure 6), as expected from their body temperature.

It should be stressed that, while the original observations pointed to a shifting mode of genome evolution in the case of the compositional transition between cold- and warm-blooded vertebrates, which is now confirmed on a sequence basis, the present results indicate the existence of a shifting mode even within cold- (e.g., fishes) and warm-blooded vertebrates (e.g., marsupials vs. eutherians).

### The two modes of genome of genome evolution: the conservative mode

The other, conservative, mode, in which the isochore patterns are maintained over evolutionary time, was found in eutherian genomes that displayed the "general compositional pattern" (e.g., human, chimp and dog genomes; as opposed to the mouse pattern; see below). Some differences in the relative amounts of isochore families were observed, but they were within narrow limits and appeared to be essentially due to differences in the relative amounts of interspersed sequences, as well as to insertions/deletions. Moreover, when isochores from MHC loci of human and mouse [28], or from synthenic chromosome regions of human and dog were examined (see Figure 4 from ref. [2] for an example), a high degree of conservation was found. Incidentally, the narrower isochore pattern of mouse was interpreted as due to an increased mutation rate [29,30] and a poor repair mechanism [31], two phenomena leading to some decrease of compositional heterogeneity.

The conservative mode was found in the present work to be further characterized by two remarkable properties that concerned the conservation in each isochore family of all vertebrate genomes investigated of (i) the average isochore size (with some limitations; see below); and (ii) the GC levels and dinucleotide frequencies. The conservation of the average isochore size may be correlated with the isochore role in chromosome organization. Indeed, it should be recalled here that the number of isochores estimated by us for the human genome, ~3200, is in agreement with the maximum number, 3000, of the highest resolution bands as assessed by Yunis et al. [32] and that the boundaries of isochores coincide with those of chromosomal bands as obtained at the resolution of 850 bands (see Figure 6 from ref. [5]). Moreover, isochores have been observed to coincide with replication units [33].

As far as the larger size of the GC-poorest isochore families of vertebrates is concerned, this may be due to the preferred insertion in these families of interspersed repeated sequences, as well as to sequence expansion phenomena [1]. Unfortunately, the presence of gaps (in medaka) or their surprising absence (in stickleback) may also contribute a possibly important artefactual component to the large size of GC-poor isochores [6]. This implies that more complete sequence data will be needed in order to obtain reliable assessments of the GC-poorest isochore size of medaka, stickleback (and also of zebrafish, opossum and chimpanzee).

The conservation of GC level and dinucleotide frequencies of isochore families can be understood by recalling that these frequencies were consistently different in the different isochore families from the human genome [9]. Such differences are likely to influence protein/DNA interactions and, therefore, chromatin structure, possibly through nucleosome positioning [34]. In turn, the existence of five isochore families suggested that a discrete number of chromatin structures are present in eutherian mammals. The different DNase accessibility of chromatin corresponding to isochores from different families [23,24] may be viewed as an indication along this line.

The conservative mode of evolution was originally explained by "negative selection acting at a regional (isochore) level to eliminate any strong deviation from the presumably functionally optimal composition of isochores" [35]. A number of findings, accumulated during the past twenty years [1,2] and those presented in this paper, support this hypothesis.

An alternative proposal for the formation and maintenance of isochores was that "biased gene conversion (BGC) is probably the most likely cause of isochores" [36]. This proposal has found a large number of supporters (see for example refs. [37,38]. While nobody disputes the existence and the importance of BGC, the link with the formation and maintenance of isochores has been the object of a debate. Indeed, there are some major problems with such a link. The first problem is that the randomness of a neutral process such as BGC and its changes in evolutionary time would lead to a tremendous variability of compositional patterns in vertebrate genomes. One would not expect, for instance, the conservation of isochore patterns in eutherian orders that have diverged about one hundred million years ago and have changed about half of the nucleotide that form their genomes [2]. The second problem is that entire vertebrate classes, orders and families (such as the class of amphibians, the vast majority of fish orders and a number of reptilian families) do not show the formation of GC-rich isochores and just show a conservative mode of evolution. The third problem is the lack of evidence, or even of models and hypotheses, concerning the expansion process from the rare, small-size BGC events (in the hundreds of bp scale) [39] to megabase regions.

In other words, if isochores were originating from BGC events, one should not expect the conservation of GC levels, sizes and (at least in Eutherians and chicken) of the relative amounts of isochore families, nor the very high similarity of GC and GC_3 _levels in orthologous genes from eutherians and birds. Instead, one should see differences in compositional patterns, and such differences should concern individual classes, orders and families of vertebrates.

## Conclusion

The present results reinforce our previous conclusions (see refs. [1,2] for reviews) concerning the mosaic organization of isochores in vertebrate genomes, the differences between the isochore patterns of warm- and cold-blooded vertebrates, the distribution of genes and the two modes (transitional and conservative) of genome evolution. Expectedly, the sequence level of the present data provide much more detailed pictures. In particular, they lead for the first time to the discovery that GC levels, dinucleotide frequencies (except for CpG in fishes) and isochore sizes from corresponding isochore families are conserved in all vertebrate genomes. These novel findings are not compatible with BGC as an explanation for the origin and conservation of isochores. This leaves the original proposal of natural selection [1,2,13] as the most plausible explanation for the origin and the maintenance of isochores, that represent, indeed, "a fundamental level of genome organization" [36].

## Methods

### Isochore mapping: the methodology

The methodology used for isochore mapping was described by Costantini et al. [3]. It essentially consists of scanning the GC levels of chromosomes by using non-overlapping 100 kb windows, the latter choice corresponding to the plateau values reached by the standard deviation of GC levels of isochores belonging to different families. A 1% GC standard deviation was accepted for 85% of the genome; a 2% GC standard deviation was accepted for the more heterogeneous GC-rich isochores, larger GC jumps being taken as borders between subsequent isochores.

After the completion of the present investigations, a paper [40] reported results obtained by using a "consensus" of four segmentation methods [41-44]. Apparently not noticed by the authors, their "consensus" results were, in fact, identical with our previous results on human, fish and chicken [3,6,7], as well as with the present results on other vertebrates. This is not surprising, because it is simply due to using our isochore boundaries and pooling all DNA segments within those boundaries. Incidentally, the differences between the human release hg18 used by Schmidt and Frishman, and that, hg17, used by us (considered to be "outdated") only consisted in the elimination of very few gaps, and our "subjective decisions" on isochore boundaries concerned a negligible number of them. In other words, the criticisms raised by Schmidt and Frishman, concerned two minor points that did not affect in the least our segmentation approach nor our conclusions. While the "consensus" approach expectedly led to isochore patterns that were identical with ours, very different results were obtained on isochore size by the four approaches compared by Schmidt and Frishman [40] when they were considered individually. Indeed, two of them led to very low average sizes (40 kb, 72 kb), the other two to very high values (~2,400 kb), the "consensus" being 100 kb. Given such differences, the utility of a "majority rule" between such different values seems to be highly disputable, and expectedly is in disagreement with our estimate.

### Isochore mapping: the resources

The entire chromosomal sequences of the finished genome assembly for five mammals, *P. troglodytes *(UCSC Release panTro2, ), *M. musculus *(UCSC Release mm9, ), *C. familiaris *(UCSC Release canFam2, ), *M. domestica *(Ensembl Release monDom5, ), *O. anatinus *(assembly deposited under the project accession AAPN00000000, NCBI ; [45]) were partitioned into non-overlapping 100 kb windows, and their GC levels calculated using the program draw_chromosome_gc.pl [46,47].

The platypus karyotype consisting of 52 chromosomes comprised a few macro- and many micro-chromosomes, as in the case of chicken genome [7]. Out of 1.84 gigabases (Gb) of assembled sequences, 437 megabases (Mb) were ordered using in situ hybridization (FISH) and oriented along 19 chromosomes [45], the remaining part of the genome being organized in ultracontigs and scaffolds that were not assigned.

In the case of the xenopus, whose genome sequence is still incomplete, the 19759 scaffolds for a total length of 1513.9 Mb, covering only half of the entire genome, were retrieved from JGI (Release v.4.1, ). The scaffolds were pooled in bins of 1% GC, in order to analyze the GC profile. The same procedure was applied in the case of the reptile *Anolis carolinensis*, whose genome was composed by 2286 scaffolds and was retrieved from NCBI ( accession number AAWZ00000000).

As far as the nomenclature of each isochore was concerned, we used a convention [3,6,7] in which the first number represented the chromosome number, the following two letters were the initials of the scientific name of the organisms under consideration, and the last number identified the isochore (see Additional Files 10, 11, 12, 13 and 14 Tables T1–T5).

### Gene distribution

The genes from chimpanzee (Release 49.21 h), dog (Release 49.2 g) and opossum (Release 49.5d) were retrieved from Ensembl , the mouse genes from Hovergen (Release 48, May 2007), the platypus genes from NCBI (Release July 2007; ), and the xenopus genes from JGI (Release v.4.1, ). Partial, putative, synthetic construct, predicted, not experimental, hypothetical protein, r-RNA, t-RNA, ribosomal and mitochondrial genes were eliminated. The cleanup program [48] was then applied, to remove redundancies from nucleotide sequences. For the remaining genes a script implemented by us was used in order to identify the coding sequences beginning with a start codon and ending with a stop codon in order to calculate reliable GC, GC_1_, GC_2 _and GC_3 _values (the GC levels of first, second and third codon position). Using this protocol, we obtained 4555 complete coding sequences for chimpanzee, 4216 for dog, 17880 for mouse, 7564 for opossum, 1995 for platypus and 27713 for xenopus.

The coordinates of the genes on the chromosomes were retrieved from the website from which the chromosomes were downloaded. The genes were localized in the isochores and gene density was calculated, with the only exception of xenopus, in which case genes were localized in the available scaffolds and gene density values were superimposed on GC profiles. In the case of anolis, the coordinates of genes were not annotated.

### Interspersed repeats of platypus and opossum

In the case of platypus and opossum, repeated sequences were retrieved from the UCSC website . We retrieved in the annotation database the files rmsk.txt.gz, which contain information on the classification of repeats. In order to calculate the percentage of repeated sequences in chromosomes we retrieved the sequences of masked chromosomes (identified by RepeatMasker and Tandem Repeat Finder).

## Abbreviations

Gb: (gigabases); GC: (molar fraction of guanine and cytosine in DNA); GC_3_: (GC level of third codon position); kb: (kilobases); Mb: (megabases).

## Competing interests

The authors declare that they have no competing interests.

## Authors' contributions

MC designed the research, analyzed the genomes and the gene sequences of the vertebrates; RC performed the analysis on reptile and amphibian sequences, and helped in the final analysis. GB designed the research and wrote the paper. All the authors contributed to the preparation of the manuscript, and all read and approved the final manuscript.

## Supplementary Material

Additional File 1 **The amount of repeated sequences**. The figure shows the amounts of repeated sequences in the platypus and opossum genome. Click here for file

Additional File 2 **Compositional patterns of platypus genome**. The figure shows the GC profile for the unassembled sequences of platypus superimposed on that of assembled chromosomes. Click here for file

Additional File 3 **Size distribution of isochores**. Size distributions of the chimpanzee, dog, mouse, opossum and platypus isochores are compared with human, chicken and fish isochores. Click here for file

Additional File 4 **Overview of chimpanzee chromosomes**. The color-coded maps show the compositional patterns of chimpanzee chromosomes. Click here for file

Additional File 5 **Overview of dog chromosomes**. The color-coded maps show the compositional patterns of dog chromosomes. Click here for file

Additional File 6 **Overview of mouse chromosomes**. The color-coded maps show the compositional patterns of the mouse chromosomes. Click here for file

Additional File 7 **Overview of opossum chromosomes**. The color-coded maps show the compositional patterns of the opossum chromosomes. Click here for file

Additional File 8 **Overview of platypus chromosomes**. The color-coded maps show the compositional patterns of platypus chromosomes. Click here for file

Additional File 9 **Compositional patterns of pufferfish and fugu**. The two panels reported the amount of DNA in pufferfish chromosomes and in scaffolds of fugu. Click here for file

Additional File 10 **Isochores in chimpanzee genome**. Coordinates, sizes, GC levels and GC standard deviations of the chimpanzee isochores. Click here for file

Additional File 11 **Isochores in dog genome**. Coordinates, sizes, GC levels and GC standard deviations of the dog isochores. Click here for file

Additional File 12 **Isochores in mouse genome**. Coordinates, sizes, GC levels and GC standard deviations of the mouse isochores. Click here for file

Additional File 13 **Isochores in opossum genome**. Coordinates, sizes, GC levels and GC standard deviations of the opossum isochores. Click here for file

Additional File 14 **Isochores in platypus genome**. Coordinates, sizes, GC levels and GC standard deviations of the platypus isochores. Click here for file
