# Supplementary figures and images for: The evolution of isochore patterns in vertebrate genomes
Source: BMC Genomics. 2009 Apr 3;10:146. doi: 10.1186/1471-2164-10-146 (PMC2678159; doi:10.1186/1471-2164-10-146)

**Figure S1**

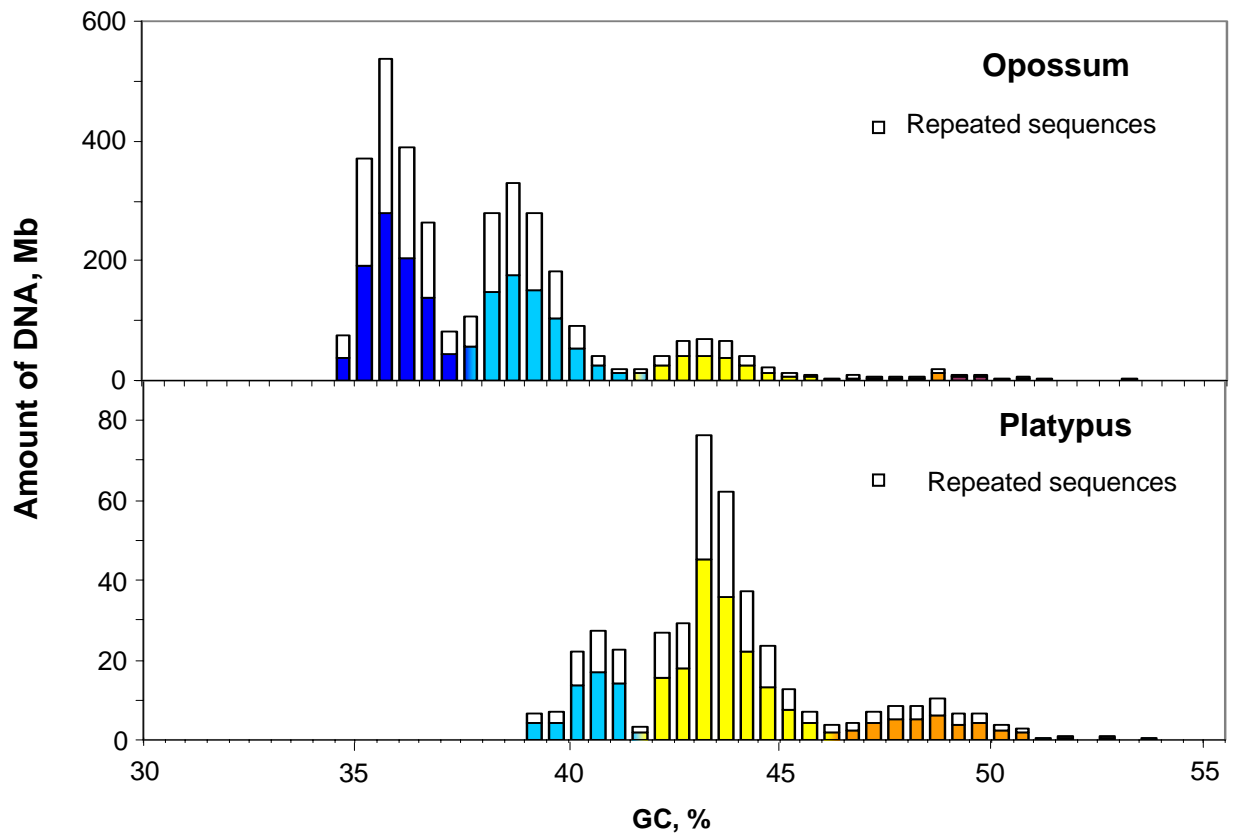

Supplement: Additional File 1 — The amount of repeated sequences. The figure shows the amounts of repeated sequences in the platypus and opossum genome. [file 1471-2164-10-146-S1.pdf]

Figure S2

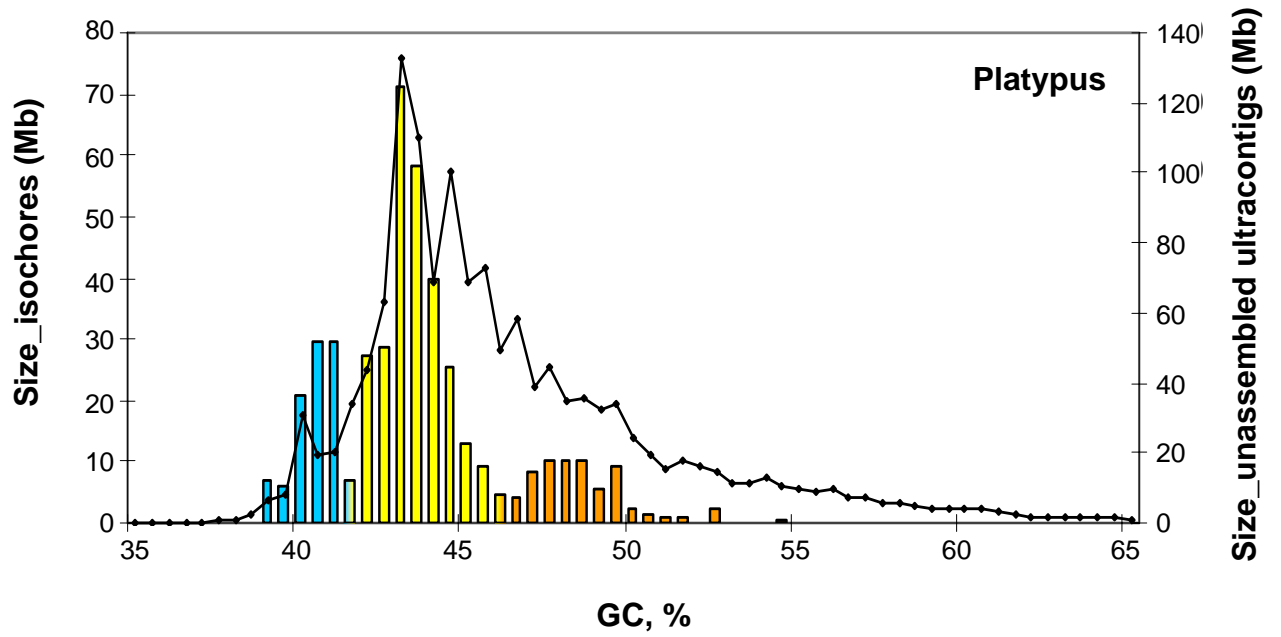

Supplement: Additional File 2 — Compositional patterns of platypus genome. The figure shows the GC profile for the unassembled sequences of platypus superimposed on that of assembled chromosomes. [file 1471-2164-10-146-S2.pdf]

Figure S3A

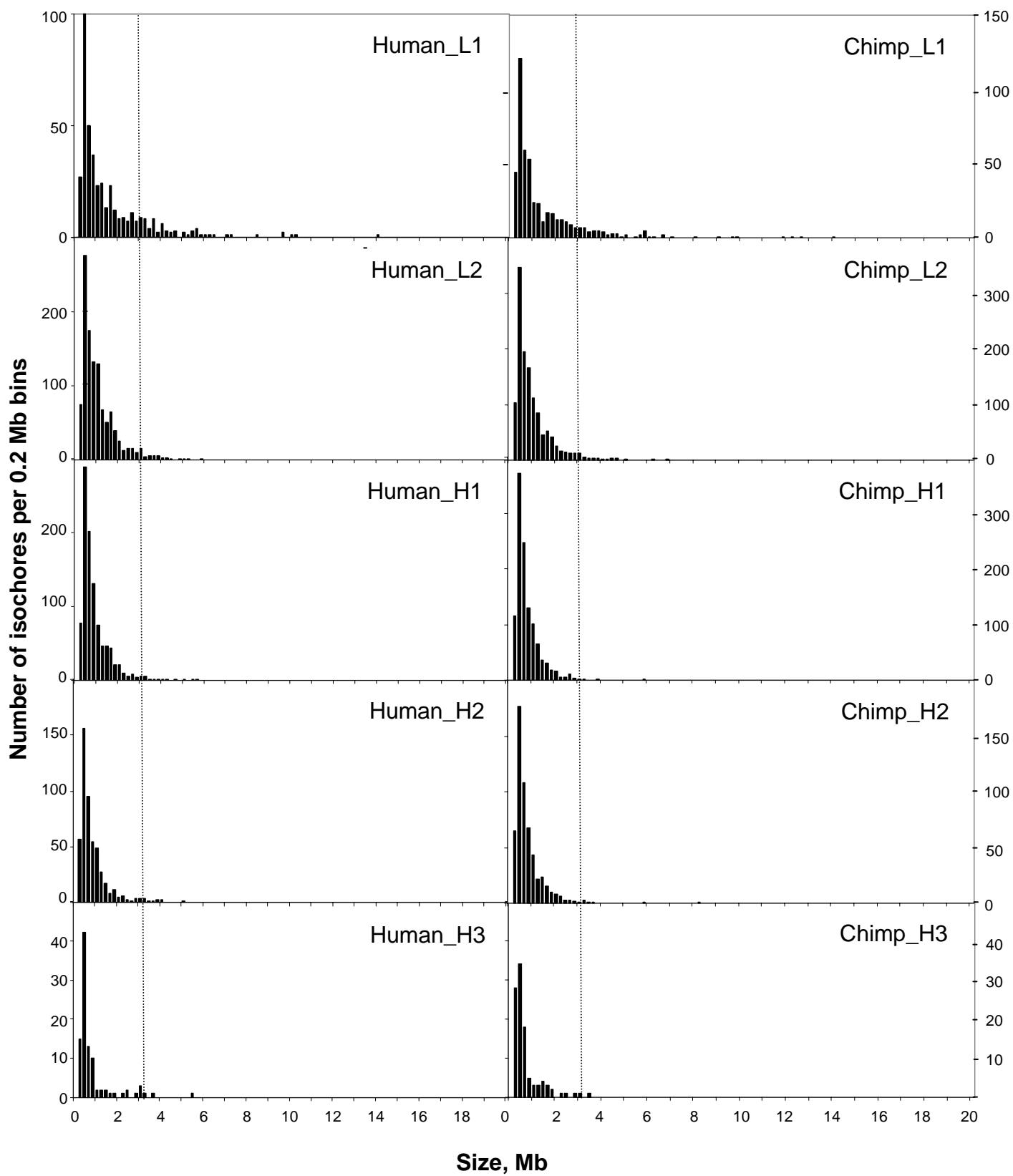

Figure S3B

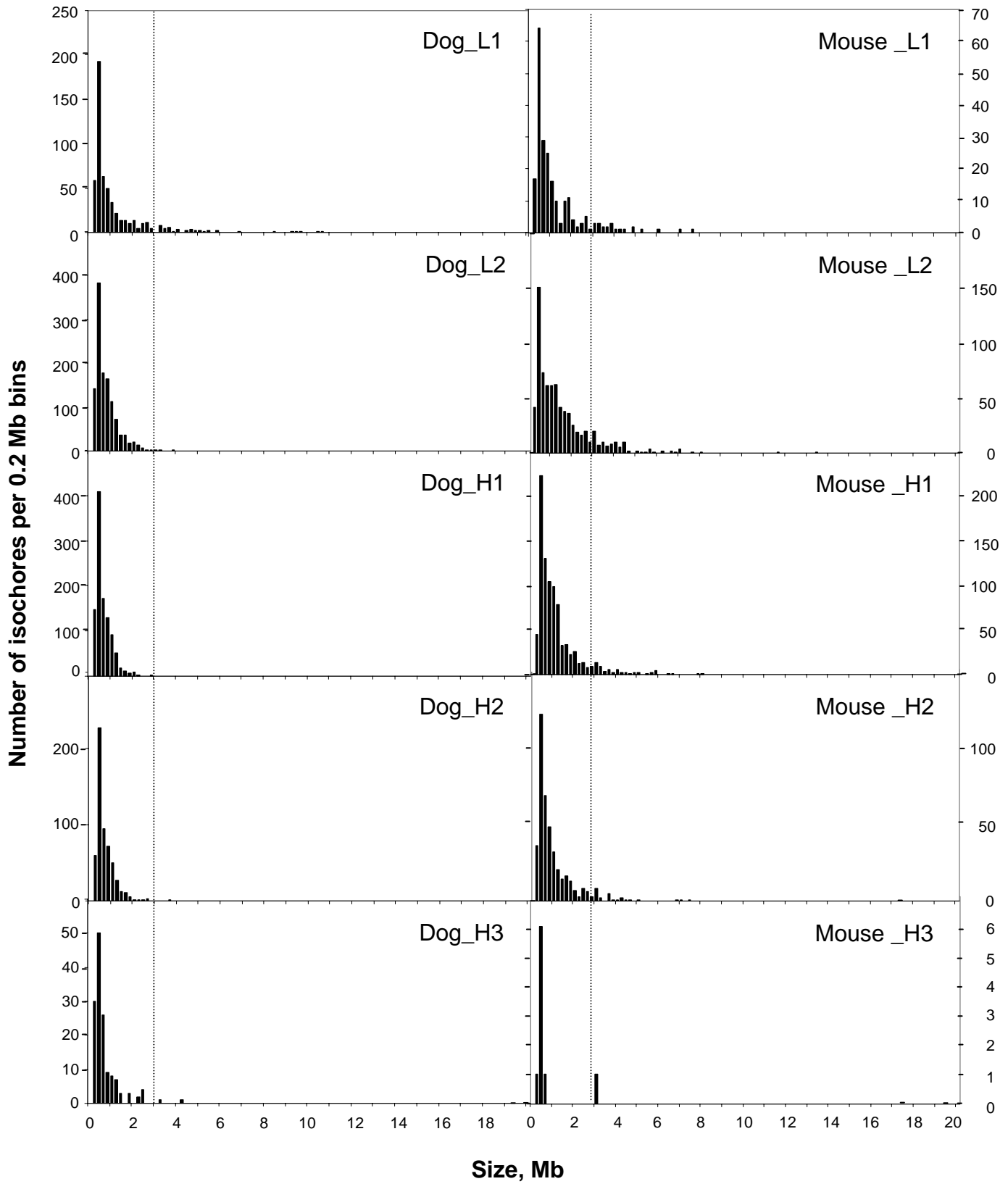

Figure S3C

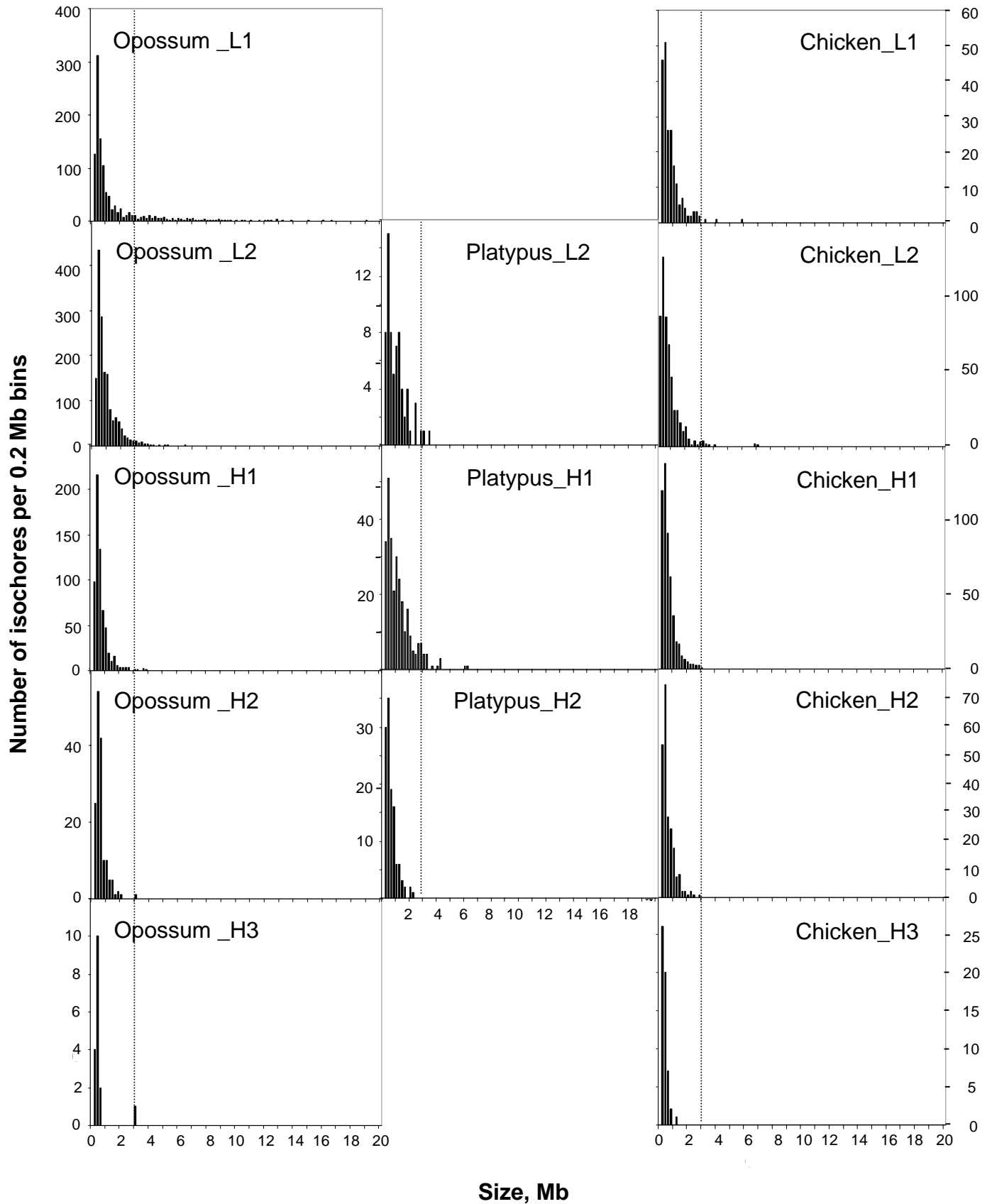

Supplement: Additional File 3 — Size distribution of isochores. Size distributions of the chimpanzee, dog, mouse, opossum and platypus isochores are compared with human, chicken and fish isochores. [file 1471-2164-10-146-S3.pdf]

Figure S4

## Chimpanzee

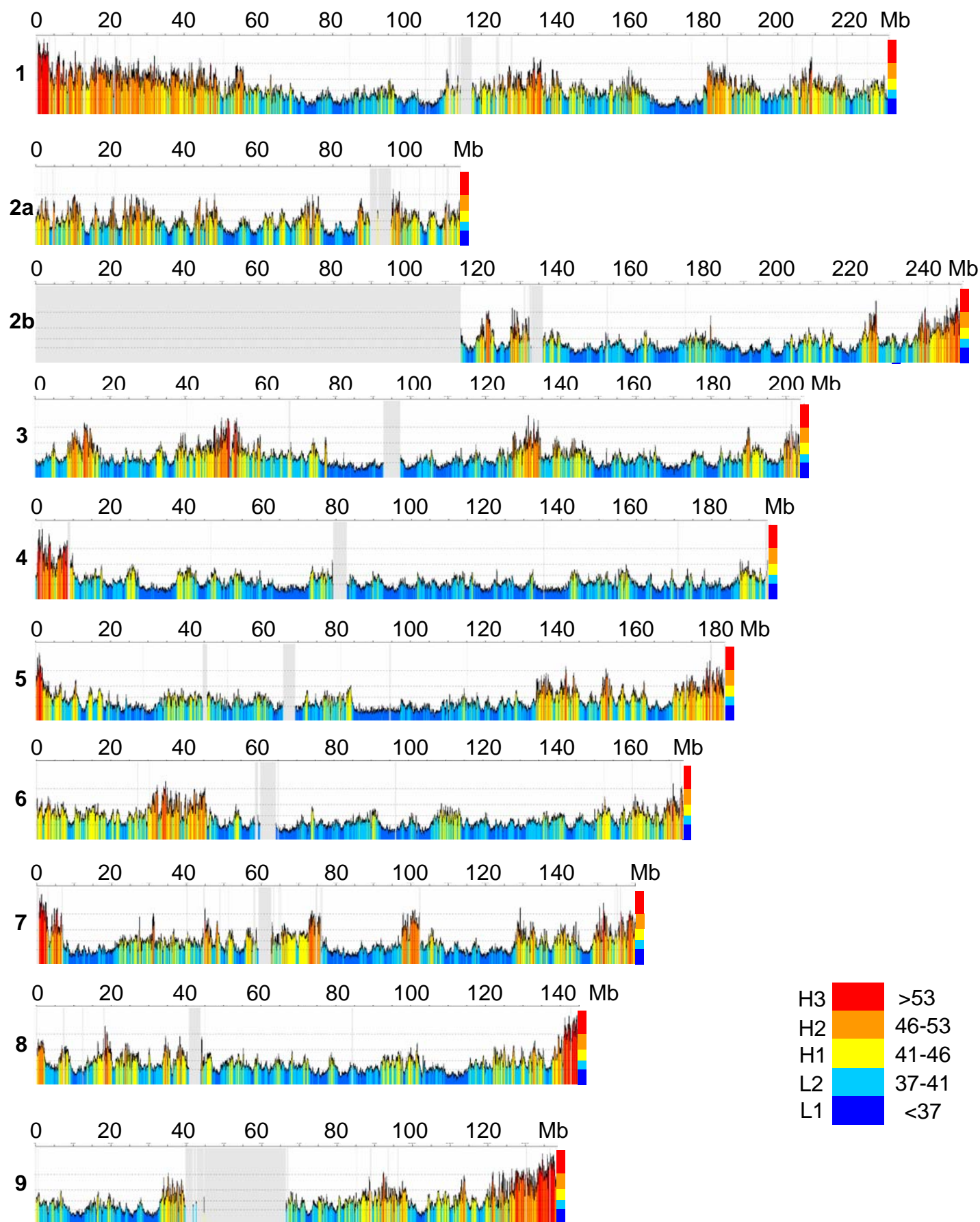

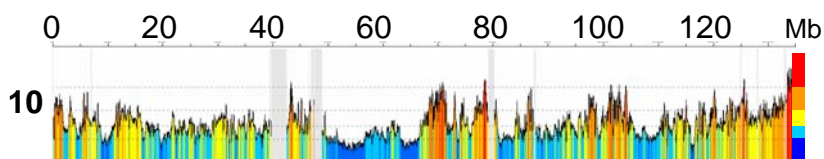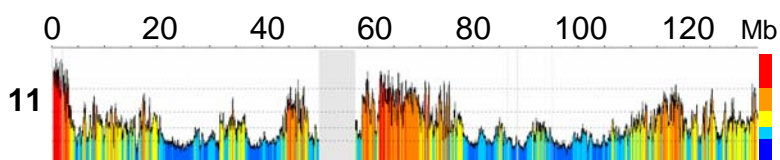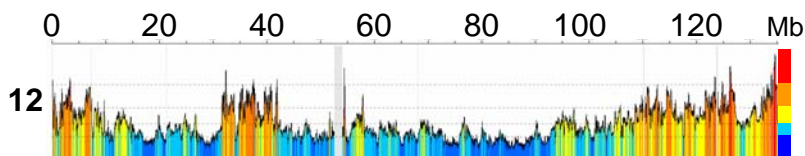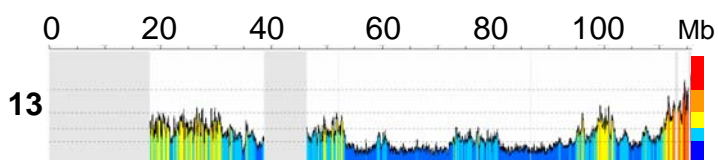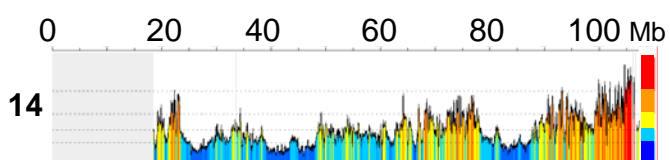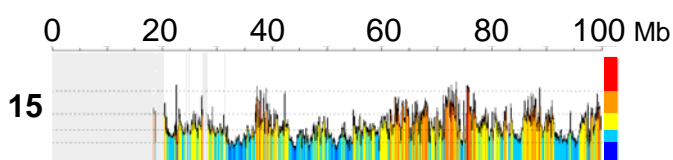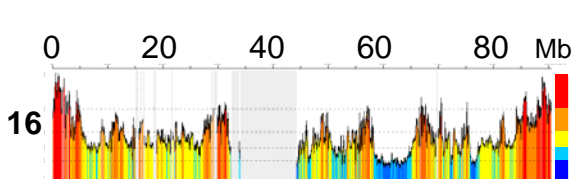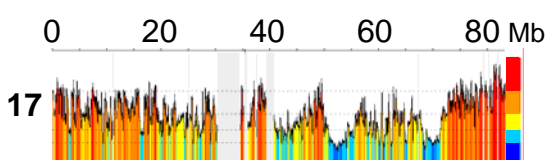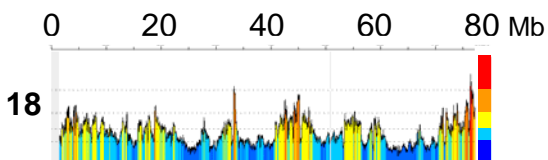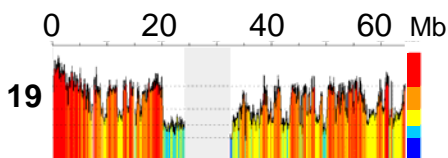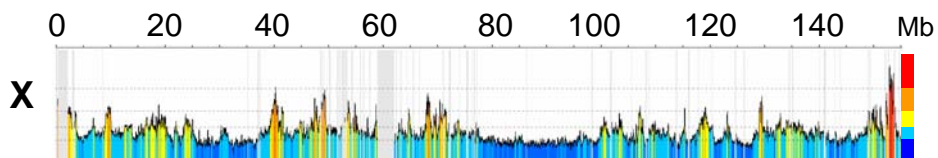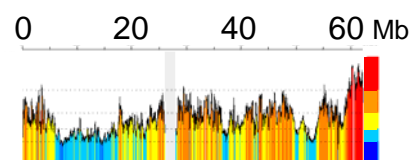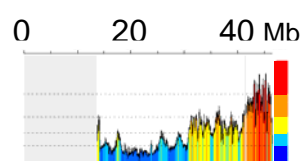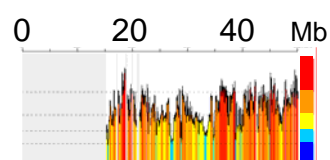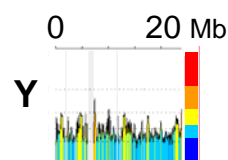

Supplement: Additional File 9 — Compositional patterns of pufferfish and fugu. The two panels reported the amount of DNA in pufferfish chromosomes and in scaffolds of fugu. [file 1471-2164-10-146-S9.pdf]

**Figure S5**

**Dog**

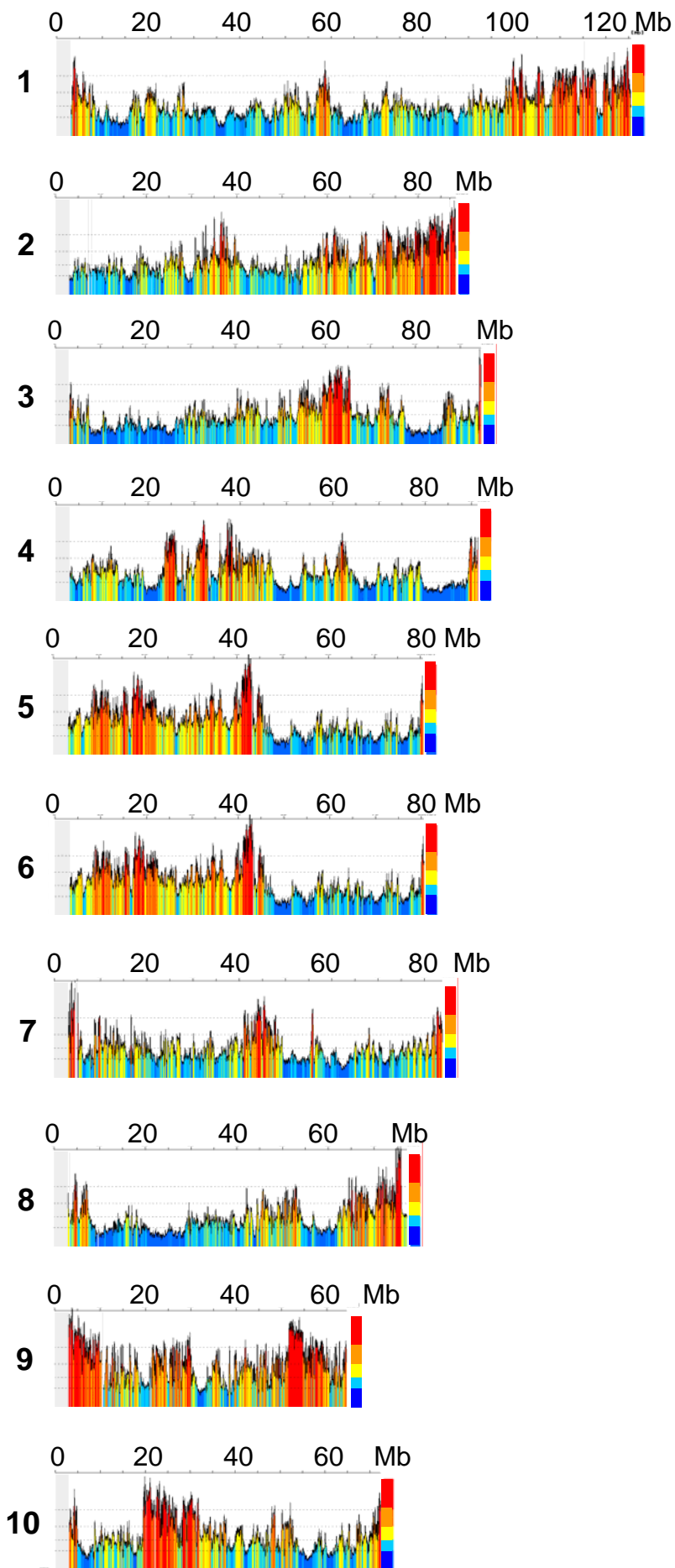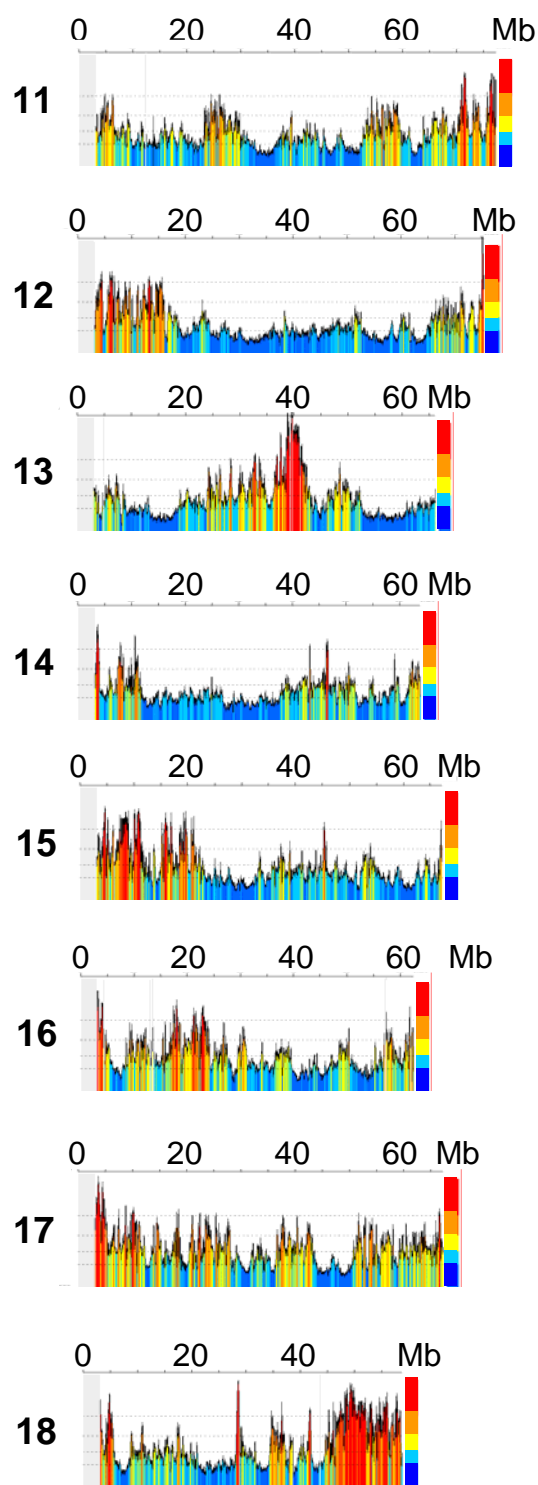

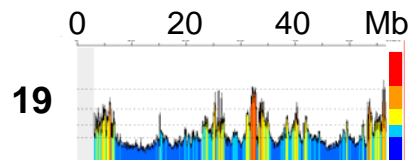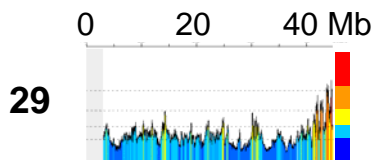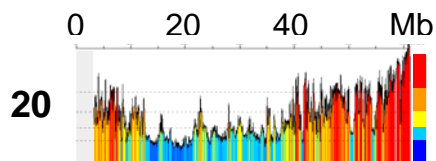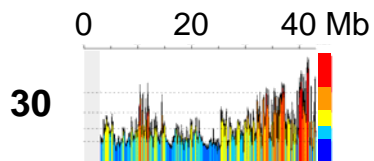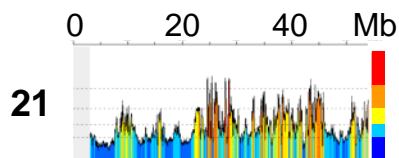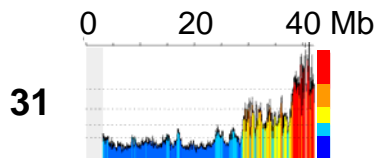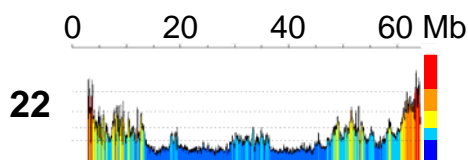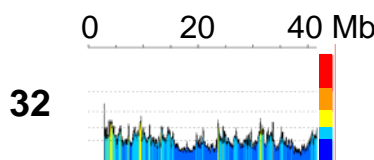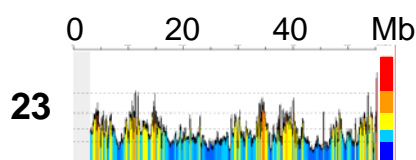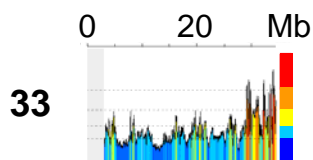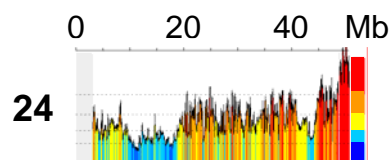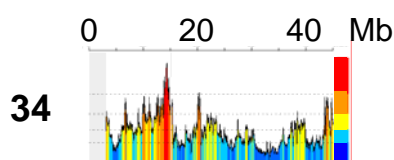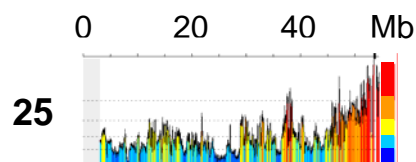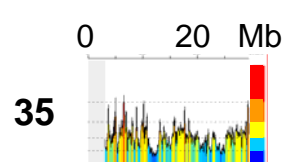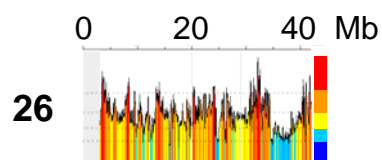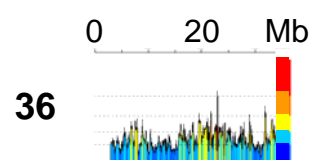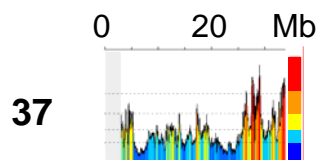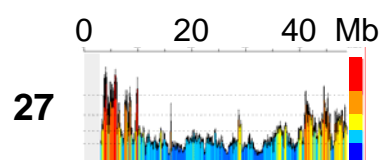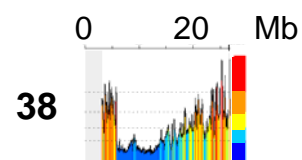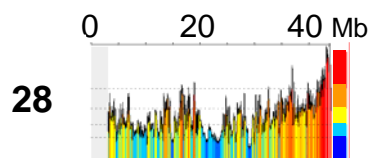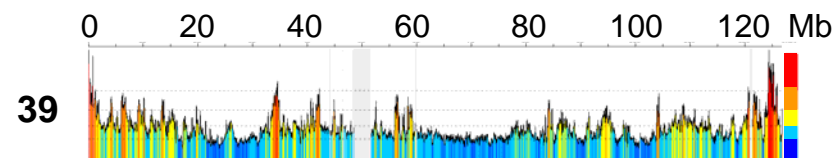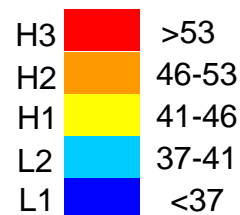

Supplement: Additional File 10 — Isochores in chimpanzee genome. Coordinates, sizes, GC levels and GC standard deviations of the chimpanzee isochores. [file 1471-2164-10-146-S10.pdf]

Figure S6

Mouse

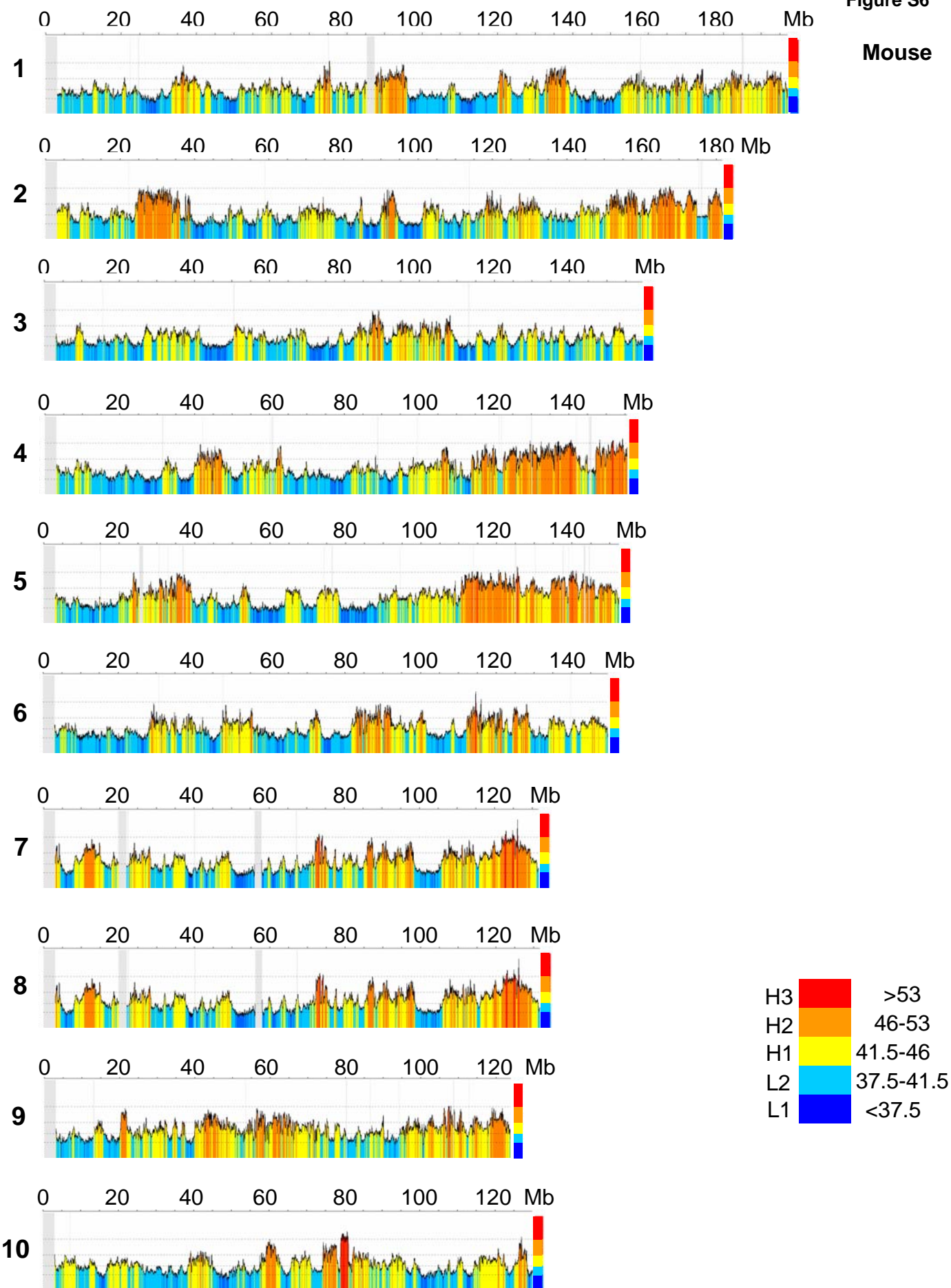

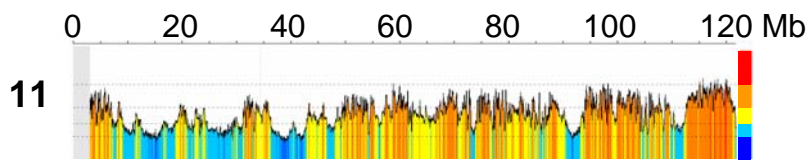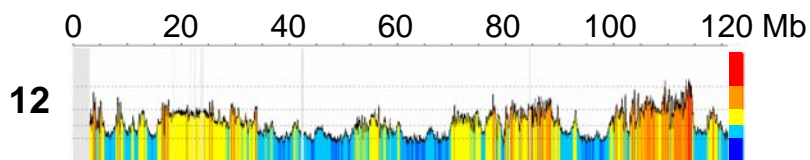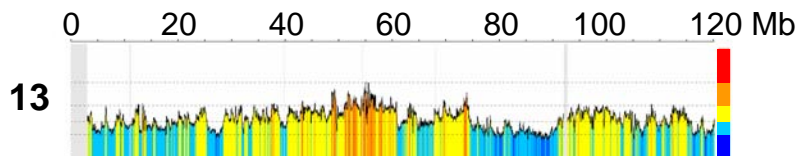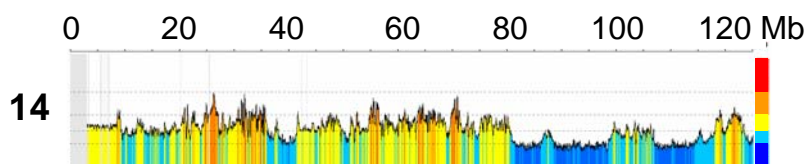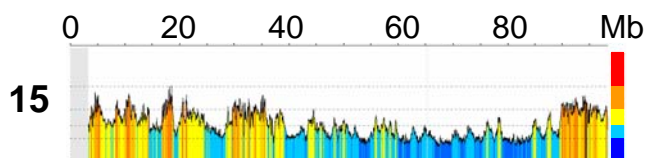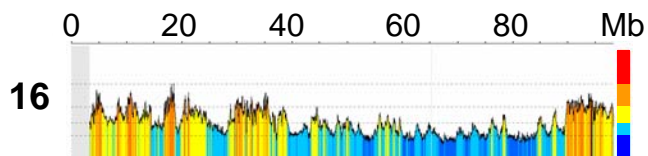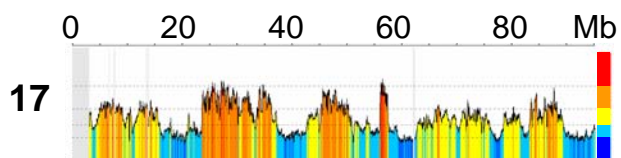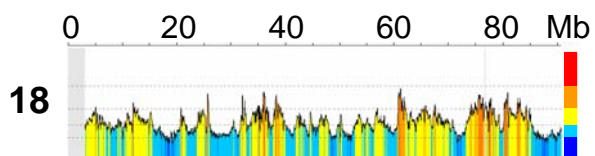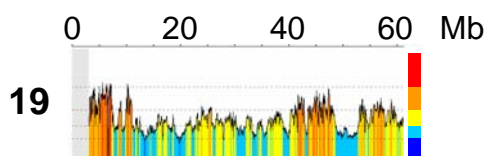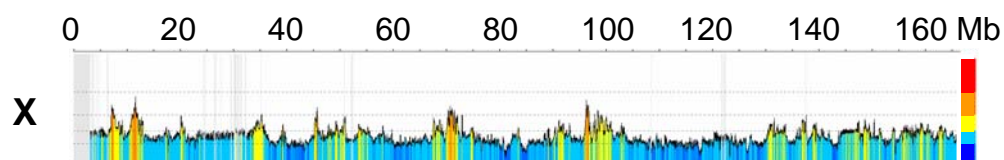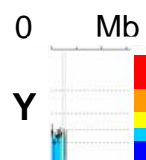

Supplement: Additional File 11 — Isochores in dog genome. Coordinates, sizes, GC levels and GC standard deviations of the dog isochores. [file 1471-2164-10-146-S11.pdf]

Figure S7

## Opossum

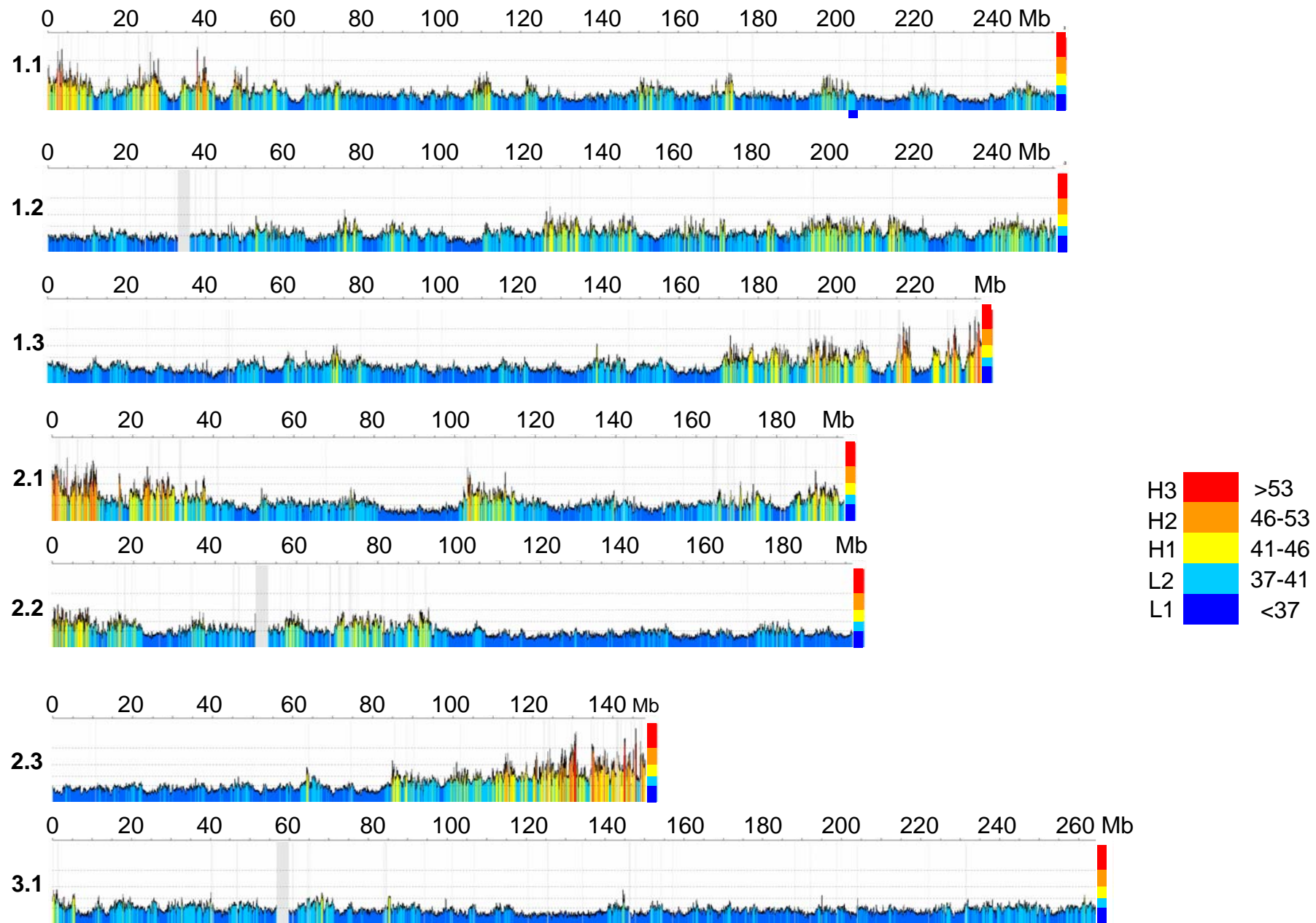

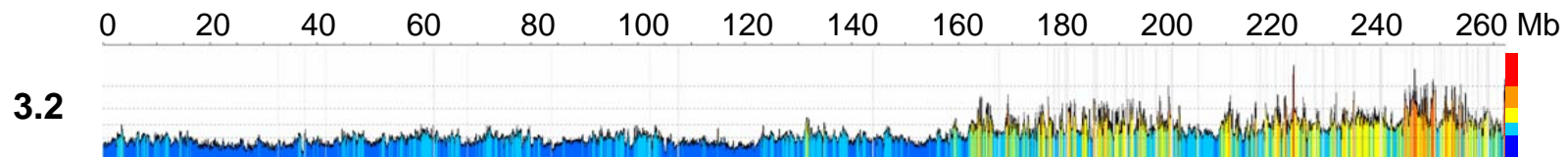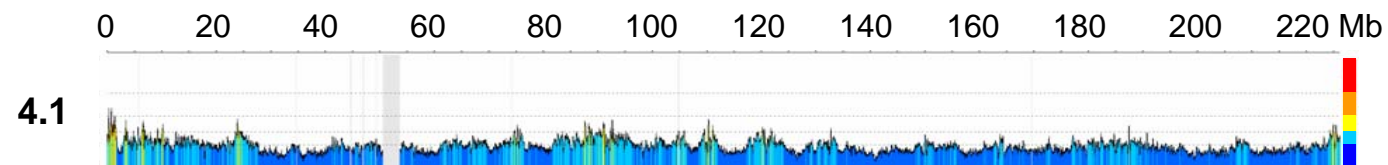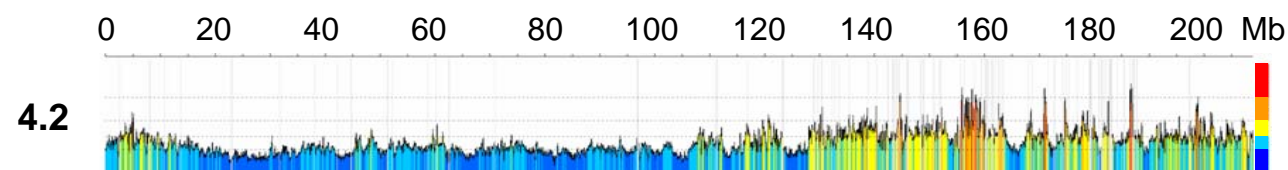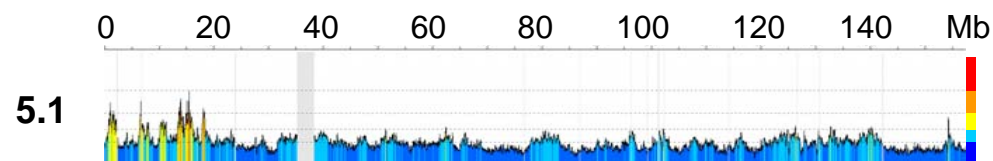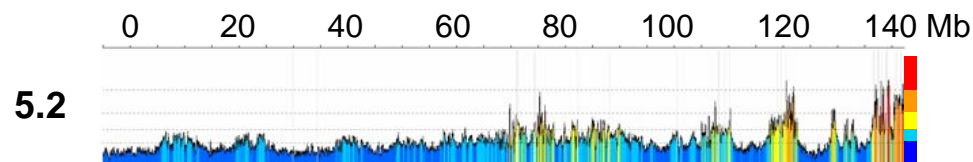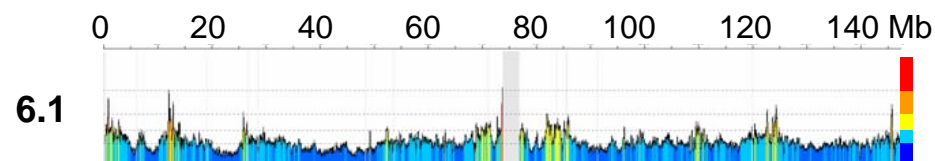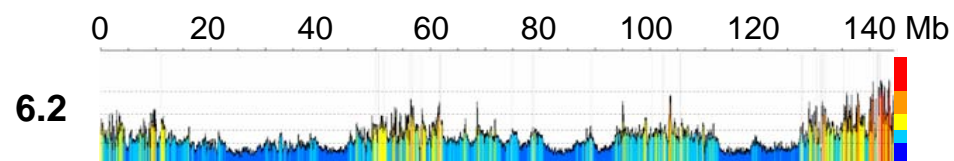

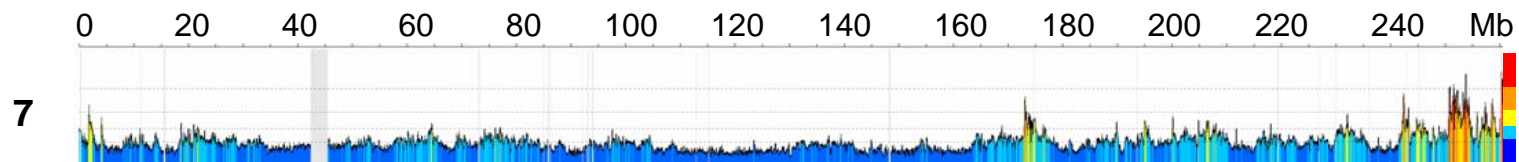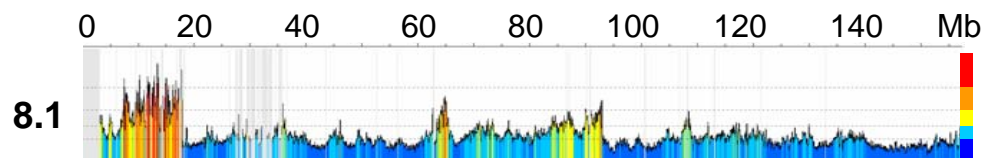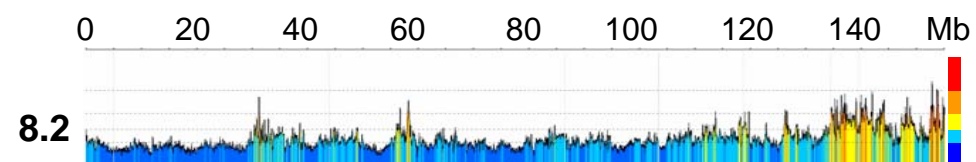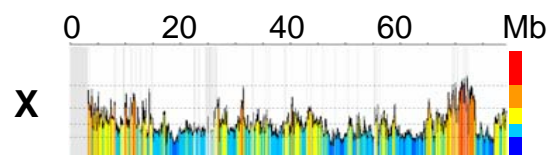

Supplement: Additional File 12 — Isochores in mouse genome. Coordinates, sizes, GC levels and GC standard deviations of the mouse isochores. [file 1471-2164-10-146-S12.pdf]

Figure S8

## Platypus

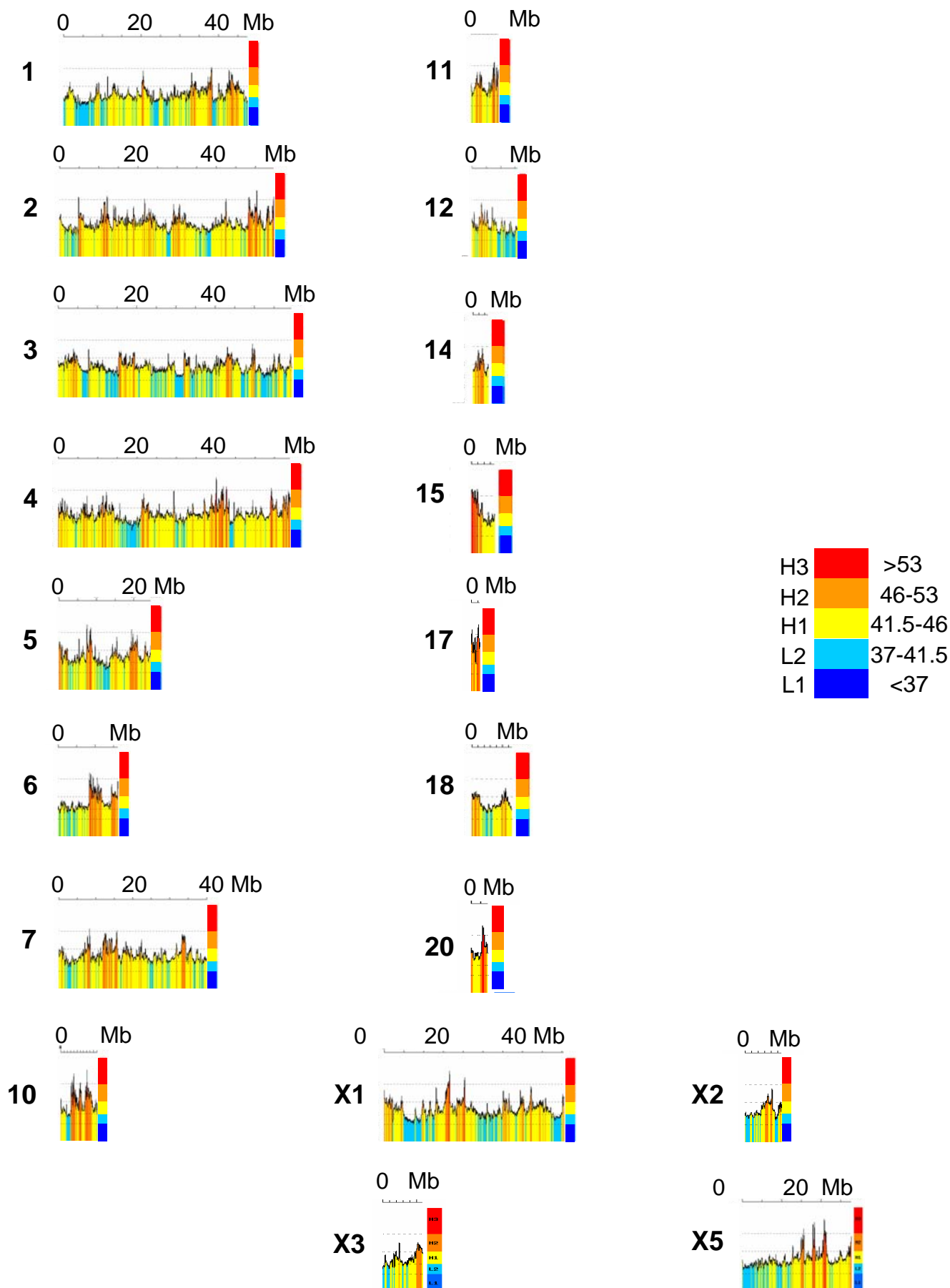

Supplement: Additional File 13 — Isochores in opossum genome. Coordinates, sizes, GC levels and GC standard deviations of the opossum isochores. [file 1471-2164-10-146-S13.pdf]

Suppl. Fig. S9

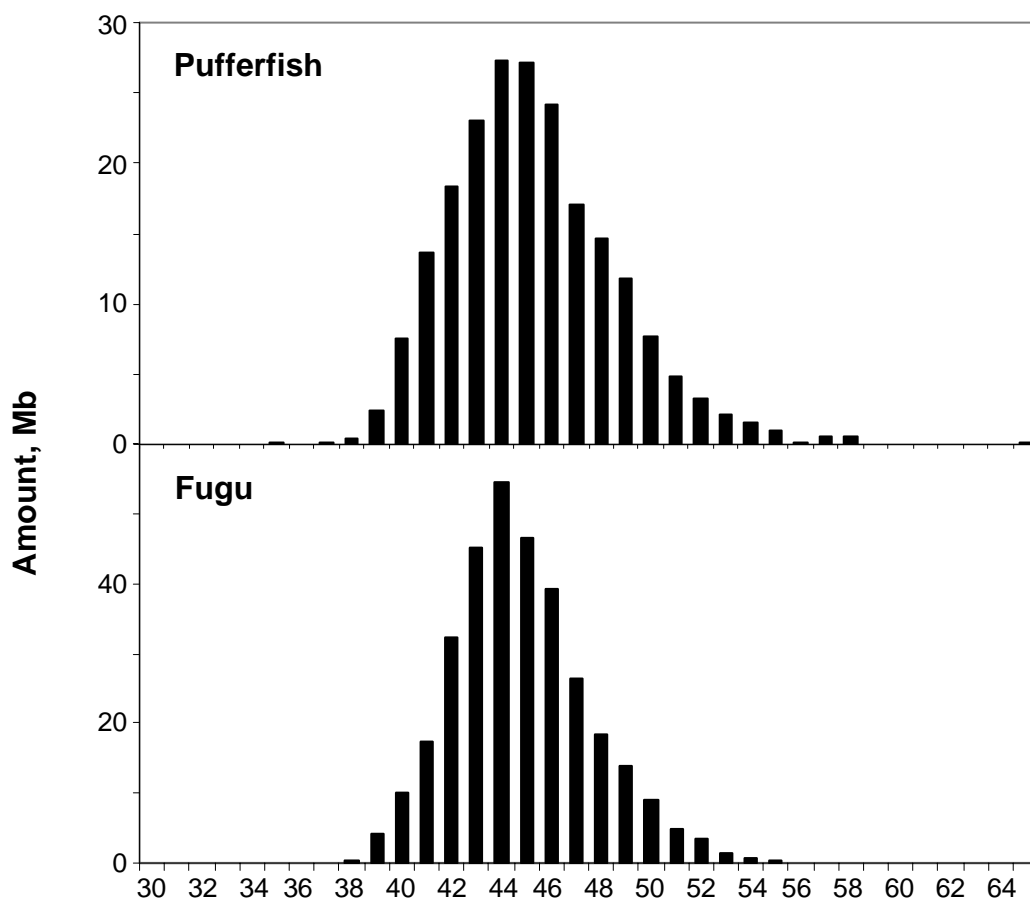

Supplement: Additional File 14 — Isochores in platypus genome. Coordinates, sizes, GC levels and GC standard deviations of the platypus isochores. [file 1471-2164-10-146-S14.pdf]
