# Supplementary material for: The evolution of isochore patterns in vertebrate genomes
Source: BMC Genomics. 2009 Apr 3;10:146. doi: 10.1186/1471-2164-10-146 (PMC2678159; doi:10.1186/1471-2164-10-146)
Supplement: Additional File 4 — Overview of chimpanzee chromosomes. The color-coded maps show the compositional patterns of chimpanzee chromosomes. [file 1471-2164-10-146-S4.pdf]

Additional Table T1. Coordinates, sizes, GC levels and GC standard deviations (SD) of the chimpanzee isochores. ΔGC indicates the difference in GC between subsequent isochores.

Isochore	Start	End	Length, Mb	GC, %	Δ GC	SD (w=100kb)
1pt1	0	0.3	0.3	43.3		0
1pt2	0.3	0.8	0.5	40.9	-2.4	4.09
1pt3	0.8	3.6	2.8	57.5	16.6	2.9
1pt4	3.6	4.7	1.1	49.4	-8.1	2.67
1pt5	4.7	5.2	0.5	43.7	-5.7	1.49
1pt6	5.2	6	0.8	48.2	4.5	2.68
1pt7	6	6.7	0.7	54.5	6.4	1.3
1pt8	6.7	7	0.3	44.4	-10.1	1.68
1pt9	7	7.2	0.2	48.0	3.6	0
1pt10	7.2	7.4	0.2	44.3	-3.7	0
1pt11	7.4	7.8	0.4	52.1	7.8	2.22
1pt12	7.8	8.2	0.4	43.5	-8.6	0.94
1pt13	8.2	8.5	0.3	47.8	4.3	1.6
1pt14	8.5	8.9	0.4	40.8	-7.0	1.29
1pt15	8.9	10.2	1.3	49.5	8.7	2.15
1pt16	10.2	10.5	0.3	43.1	-6.3	1.98
1pt17	10.5	10.8	0.3	45.9	2.8	0.78
1pt18	10.8	11	0.2	55.9	10.0	0
1pt19	11	11.8	0.8	49.2	-6.7	2.6
1pt20	11.8	12	0.2	53.4	4.2	0
1pt21	12	12.4	0.4	51.3	-2.1	1.19
1pt22	12.4	12.7	0.3	42.9	-8.4	1.85
1pt23	12.7	12.9	0.2	48.8	5.9	0
1pt24	12.9	13.1	0.2	44.4	-4.4	0
1pt25	13.1	13.7	0.6	46.8	2.4	1.39
1pt26	13.7	14.8	1.1	43.1	-3.7	1.43
1pt27	14.8	15.7	0.9	48.9	5.8	2.19
1pt28	15.7	16.2	0.5	47.4	-1.5	3.58
1pt29	16.2	16.4	0.2	55.3	7.9	0
1pt30	16.4	19.9	3.5	49.3	-6.0	2.68
1pt31	19.9	20.4	0.5	45.6	-3.8	2.66
1pt32	20.4	20.9	0.5	49.0	3.5	0.91
1pt33	20.9	21.3	0.4	39.2	-9.9	1.53
1pt34	21.3	23.1	1.8	49.6	10.4	4.06
1pt35	23.1	23.6	0.5	43.8	-5.8	2.22
1pt36	23.6	24	0.4	48.6	4.8	0.81
1pt37	24	24.2	0.2	45.4	-3.2	0
1pt38	24.2	24.6	0.4	48.2	2.7	1.67
1pt39	24.6	24.9	0.3	44.2	-4.0	0.4
1pt40	24.9	26.8	1.9	48.0	3.8	3.47
1pt41	26.8	27	0.2	45.1	-2.8	0
1pt42	27	27.9	0.9	49.7	4.5	2.19
1pt43	27.9	28.4	0.5	44.3	-5.3	2.6
1pt44	28.4	28.6	0.2	46.6	2.3	0
1pt45	28.6	29.4	0.8	45.1	-1.5	3.4
1pt46	29.4	31.6	2.2	47.9	2.7	2.64
1pt47	31.6	31.9	0.3	45.1	-2.7	0.13
1pt48	31.9	32.2	0.3	51.7	6.5	4.6
1pt49	32.2	32.6	0.4	45.2	-6.5	0.81
1pt50	32.6	32.9	0.3	49.0	3.9	2.24

1pt51	32.9	33.1	0.2	44.1	-4.9	0
1pt52	33.1	33.4	0.3	48.0	3.9	1.45
1pt53	33.4	33.6	0.2	45.8	-2.2	0
1pt54	33.6	33.8	0.2	47.5	1.7	0
1pt55	33.8	35.2	1.4	44.5	-3.0	1.51
1pt56	35.2	35.4	0.2	48.1	3.6	0
1pt57	35.4	36.6	1.2	42.3	-5.9	1.92
1pt58	36.6	38.8	2.2	48.2	6.0	2.19
1pt59	38.8	40.1	1.3	43.8	-4.5	1.73
1pt60	40.1	40.6	0.5	48.0	4.3	1.34
1pt61	40.6	41.4	0.8	43.2	-4.9	1.82
1pt62	41.4	41.7	0.3	48.5	5.4	1.97
1pt63	41.7	41.9	0.2	39.5	-9.0	0
1pt64	41.9	42.6	0.7	48.3	8.8	3.24
1pt65	42.6	43.3	0.7	40.7	-7.6	2.02
1pt66	43.3	44.4	1.1	46.7	6.0	3.59
1pt67	44.4	44.6	0.2	43.1	-3.6	0
1pt68	44.6	45.6	1	47.7	4.5	3.2
1pt69	45.6	45.8	0.2	43.5	-4.2	0
1pt70	45.8	46	0.2	40.4	-3.2	0
1pt71	46	46.9	0.9	42.7	2.3	2.21
1pt72	46.9	47.4	0.5	49.0	6.3	2.58
1pt73	47.4	48.2	0.8	42.7	-6.2	1.58
1pt74	48.2	48.8	0.6	48.1	5.3	1.42
1pt75	48.8	49.1	0.3	42.6	-5.5	3.56
1pt76	49.1	49.3	0.2	37.3	-5.3	0
1pt77	49.3	49.6	0.3	43.9	6.6	0.9
1pt78	49.6	50.4	0.8	38.6	-5.3	1.13
1pt79	50.4	50.8	0.4	36.6	-2.0	0.33
1pt80	50.8	51	0.2	39.3	2.8	0
1pt81	51	51.3	0.3	43.6	4.3	1.18
1pt82	51.3	52	0.7	38.4	-5.2	1.22
1pt83	52	52.2	0.2	45.1	6.7	0
1pt84	52.2	52.7	0.5	39.4	-5.6	1.9
1pt85	52.7	54	1.3	42.6	3.2	2.07
1pt86	54	54.8	0.8	48.2	5.7	3.78
1pt87	54.8	55.1	0.3	44.0	-4.2	0.46
1pt88	55.1	56	0.9	49.0	4.9	1.73
1pt89	56	56.6	0.6	42.1	-6.9	2.54
1pt90	56.6	57.4	0.8	39.6	-2.5	0.76
1pt91	57.4	57.6	0.2	43.3	3.7	0
1pt92	57.6	57.8	0.2	37.5	-5.7	0
1pt93	57.8	58.1	0.3	42.0	4.4	0.53
1pt94	58.1	59	0.9	40.4	-1.6	0.84
1pt95	59	60.3	1.3	41.8	1.4	1.69
1pt96	60.3	63.2	2.9	39.4	-2.3	1.48
1pt97	63.2	63.6	0.4	44.6	5.2	2.72
1pt98	63.6	64	0.4	37.1	-7.5	2.7
1pt99	64	64.8	0.8	39.6	2.5	2.35
1pt100	64.8	65.1	0.3	42.3	2.7	1.35
1pt101	65.1	66	0.9	40.3	-2.0	1.12
1pt102	66	66.4	0.4	42.2	1.9	0.93
1pt103	66.4	66.9	0.5	38.9	-3.4	1.51
1pt104	66.9	67.3	0.4	36.5	-2.3	0.11
1pt105	67.3	68.5	1.2	38.4	1.8	1.13
1pt106	68.5	69	0.5	42.2	3.9	1.02

1pt107	69	69.6	0.6	40.4	-1.9	1.18
1pt108	69.6	69.9	0.3	36.3	-4.0	0.33
1pt109	69.9	70.2	0.3	38.2	1.9	0.3
1pt110	70.2	71.5	1.3	36.0	-2.1	0.97
1pt111	71.5	72.5	1	38.0	1.9	0.76
1pt112	72.5	75.8	3.3	35.2	-2.8	0.99
1pt113	75.8	80.1	4.3	38.2	3.0	1.34
1pt114	80.1	82.8	2.7	35.4	-2.7	0.8
1pt115	82.8	83.2	0.4	37.9	2.5	0.85
1pt116	83.2	84.9	1.7	36.3	-1.7	1.07
1pt117	84.9	86.2	1.3	38.1	1.8	1.8
1pt118	86.2	86.4	0.2	41.2	3.2	0
1pt119	86.4	87.1	0.7	39.8	-1.4	1.59
1pt120	87.1	87.8	0.7	36.5	-3.3	0.76
1pt121	87.8	88.6	0.8	38.6	2.1	1.64
1pt122	88.6	88.8	0.2	41.8	3.2	0
1pt123	88.8	89.7	0.9	38.3	-3.5	1.12
1pt124	89.7	89.9	0.2	36.8	-1.4	0
1pt125	89.9	92.5	2.6	38.8	2.0	1.22
1pt126	92.5	92.7	0.2	41.7	2.9	0
1pt127	92.7	93	0.3	38.2	-3.6	1.21
1pt128	93	93.4	0.4	42.5	4.3	0.82
1pt129	93.4	95.1	1.7	39.3	-3.2	1.52
1pt130	95.1	95.6	0.5	43.6	4.3	2.09
1pt131	95.6	95.8	0.2	39.8	-3.8	0
1pt132	95.8	96.5	0.7	41.6	1.9	2.03
1pt133	96.5	97.4	0.9	38.8	-2.8	1.04
1pt134	97.4	97.6	0.2	35.4	-3.3	0
1pt135	97.6	98.3	0.7	37.4	2.0	0.96
1pt136	98.3	100.5	2.2	35.8	-1.6	1.14
1pt137	100.5	103.1	2.6	38.1	2.3	1.21
1pt138	103.1	108.9	5.8	35.0	-3.1	1.03
1pt139	108.9	110.5	1.6	39.0	4.0	1.34
1pt140	110.5	111.6	1.1	44.5	5.5	2.93
1pt141	111.6	111.8	0.2	0.0	-44.5	0
1pt142	111.8	112.6	0.8	42.2	42.2	1.61
1pt143	112.6	113	0.4	39.5	-2.7	0.95
1pt144	113	113.2	0.2	42.2	2.7	0
1pt145	113.2	113.6	0.4	40.2	-2.0	1.49
1pt146	113.6	114.2	0.6	43.5	3.3	2.07
1pt147	114.2	114.4	0.2	39.7	-3.8	0
1pt148	114.4	114.7	0.3	42.4	2.8	1.07
1pt149	114.7	116.9	2.2	0.0	-42.4	0
1pt150	116.9	117.1	0.2	37.2	37.2	0
1pt151	117.1	117.3	0.2	0.0	-37.2	0
1pt152	117.3	117.6	0.3	39.2	39.2	0.58
1pt153	117.6	118.3	0.7	42.4	3.2	1.09
1pt154	118.3	118.8	0.5	40.0	-2.4	1.98
1pt155	118.8	119.8	1	37.5	-2.5	1.13
1pt156	119.8	120	0.2	44.3	6.7	0
1pt157	120	120.3	0.3	38.1	-6.2	2.91
1pt158	120.3	121.5	1.2	43.2	5.1	0.95
1pt159	121.5	121.9	0.4	40.0	-3.2	0.76
1pt160	121.9	122.6	0.7	42.5	2.5	1.38
1pt161	122.6	123.3	0.7	38.4	-4.2	1.9
1pt162	123.3	123.8	0.5	43.8	5.5	2.25

1pt163	123.8	124.4	0.6	37.8	-6.0	1.26
1pt164	124.4	125.7	1.3	42.9	5.0	2.02
1pt165	125.7	125.9	0.2	47.4	4.5	0
1pt166	125.9	126.6	0.7	41.9	-5.5	1.89
1pt167	126.6	127	0.4	38.2	-3.6	0.65
1pt168	127	127.5	0.5	44.7	6.4	3.03
1pt169	127.5	128.2	0.7	47.1	2.4	3.09
1pt170	128.2	128.2	0	0.0	-47.1	0
1pt171	128.2	129.7	1.5	43.5	43.5	2.86
1pt172	129.7	129.9	0.2	40.2	-3.3	0
1pt173	129.9	130.4	0.5	46.9	6.7	2.34
1pt174	130.4	131.3	0.9	44.0	-2.8	1.73
1pt175	131.3	131.5	0.2	39.2	-4.8	0
1pt176	131.5	132.1	0.6	41.8	2.6	1.06
1pt177	132.1	132.3	0.2	38.3	-3.6	0
1pt178	132.3	132.6	0.3	44.9	6.6	1.48
1pt179	132.6	133.1	0.5	47.7	2.9	0.73
1pt180	133.1	133.4	0.3	44.2	-3.5	1.83
1pt181	133.4	133.7	0.3	47.5	3.3	0.89
1pt182	133.7	133.9	0.2	45.1	-2.4	0
1pt183	133.9	134.1	0.2	49.1	4.0	0
1pt184	134.1	134.3	0.2	53.7	4.7	0
1pt185	134.3	134.5	0.2	51.9	-1.8	0
1pt186	134.5	135.1	0.6	43.4	-8.4	2.04
1pt187	135.1	136.4	1.3	49.5	6.1	2.77
1pt188	136.4	136.8	0.4	42.8	-6.7	0.82
1pt189	136.8	137.7	0.9	41.3	-1.5	3.7
1pt190	137.7	138	0.3	36.4	-4.9	0.54
1pt191	138	139	1	38.5	2.1	2.07
1pt192	139	139.5	0.5	46.8	8.3	2.19
1pt193	139.5	139.7	0.2	44.3	-2.5	0
1pt194	139.7	139.9	0.2	40.3	-4.0	0
1pt195	139.9	140.3	0.4	43.4	3.0	1.48
1pt196	140.3	140.8	0.5	46.8	3.4	2.44
1pt197	140.8	141	0.2	42.3	-4.5	0
1pt198	141	141.2	0.2	38.6	-3.7	0
1pt199	141.2	141.7	0.5	42.8	4.2	1.61
1pt200	141.7	142.8	1.1	39.0	-3.8	1.24
1pt201	142.8	143	0.2	36.8	-2.1	0
1pt202	143	143.8	0.8	37.5	0.6	1.3
1pt203	143.8	144.1	0.3	41.9	4.4	0.46
1pt204	144.1	144.4	0.3	40.6	-1.3	0.15
1pt205	144.4	145.5	1.1	42.5	1.9	1.44
1pt206	145.5	146.1	0.6	38.7	-3.8	1.55
1pt207	146.1	146.6	0.5	41.9	3.2	1.61
1pt208	146.6	146.8	0.2	38.9	-3.0	0
1pt209	146.8	147.2	0.4	44.2	5.3	1.85
1pt210	147.2	147.5	0.3	39.2	-5.0	2.48
1pt211	147.5	147.8	0.3	41.9	2.6	0.18
1pt212	147.8	148.6	0.8	39.7	-2.2	1.12
1pt213	148.6	148.8	0.2	36.4	-3.2	0
1pt214	148.8	149.3	0.5	38.9	2.5	1
1pt215	149.3	149.5	0.2	35.4	-3.5	0
1pt216	149.5	153.7	4.2	38.9	3.5	1.52
1pt217	153.7	154	0.3	36.4	-2.5	0.66
1pt218	154	154.6	0.6	38.7	2.3	1.09

1pt219	154.6	155.5	0.9	43.6	4.9	1.39
1pt220	155.5	155.7	0.2	36.2	-7.4	0
1pt221	155.7	156.4	0.7	38.7	2.5	1.3
1pt222	156.4	157	0.6	41.6	2.9	0.79
1pt223	157	157.4	0.4	39.4	-2.2	0.86
1pt224	157.4	157.6	0.2	42.5	3.2	0
1pt225	157.6	158	0.4	37.8	-4.7	2.01
1pt226	158	158.2	0.2	43.3	5.5	0
1pt227	158.2	158.5	0.3	38.1	-5.2	2.76
1pt228	158.5	158.9	0.4	43.1	5.0	2.32
1pt229	158.9	159.3	0.4	39.2	-3.9	1.26
1pt230	159.3	159.5	0.2	43.5	4.3	0
1pt231	159.5	159.7	0.2	37.2	-6.4	0
1pt232	159.7	159.9	0.2	47.5	10.3	0
1pt233	159.9	160.5	0.6	38.0	-9.5	2.43
1pt234	160.5	161.5	1	44.3	6.3	2.55
1pt235	161.5	161.7	0.2	40.2	-4.1	0
1pt236	161.7	162.4	0.7	42.7	2.5	1.35
1pt237	162.4	162.8	0.4	39.8	-2.9	1.04
1pt238	162.8	163.1	0.3	43.7	3.9	2.05
1pt239	163.1	165.5	2.4	39.5	-4.2	1.33
1pt240	165.5	166.2	0.7	36.1	-3.4	1.28
1pt241	166.2	166.5	0.3	37.5	1.4	0.33
1pt242	166.5	172.6	6.1	34.6	-2.9	1.09
1pt243	172.6	173.4	0.8	37.3	2.7	0.94
1pt244	173.4	177.7	4.3	34.7	-2.6	1.1
1pt245	177.7	178.1	0.4	38.4	3.6	1.17
1pt246	178.1	179.9	1.8	36.1	-2.3	1.26
1pt247	179.9	180.9	1	41.2	5.0	1.03
1pt248	180.9	182.4	1.5	49.1	7.9	2.4
1pt249	182.4	182.6	0.2	38.8	-10.2	0
1pt250	182.6	183.1	0.5	45.3	6.5	1.79
1pt251	183.1	183.8	0.7	47.7	2.3	1.82
1pt252	183.8	184.1	0.3	42.7	-5.0	1.96
1pt253	184.1	185.2	1.1	47.8	5.2	2.43
1pt254	185.2	185.4	0.2	44.5	-3.3	0
1pt255	185.4	185.7	0.3	49.9	5.4	3.33
1pt256	185.7	186.8	1.1	44.6	-5.2	2.48
1pt257	186.8	187.5	0.7	45.5	0.9	2.14
1pt258	187.5	188.2	0.7	38.8	-6.7	1.09
1pt259	188.2	188.7	0.5	46.1	7.3	2.09
1pt260	188.7	189.8	1.1	40.5	-5.6	1
1pt261	189.8	190.3	0.5	43.9	3.5	2.29
1pt262	190.3	190.7	0.4	38.6	-5.3	2.23
1pt263	190.7	191.2	0.5	42.8	4.2	1.15
1pt264	191.2	191.9	0.7	40.4	-2.4	0.84
1pt265	191.9	192.4	0.5	43.8	3.3	2.69
1pt266	192.4	192.6	0.2	39.2	-4.6	0
1pt267	192.6	193.5	0.9	43.6	4.3	2.56
1pt268	193.5	193.8	0.3	38.5	-5.1	2.27
1pt269	193.8	195.1	1.3	41.6	3.1	1.03
1pt270	195.1	195.6	0.5	38.4	-3.2	1.48
1pt271	195.6	197.3	1.7	36.6	-1.8	0.88
1pt272	197.3	198.1	0.8	38.5	1.9	1.12
1pt273	198.1	198.5	0.4	35.7	-2.8	0.42
1pt274	198.5	199.6	1.1	38.5	2.8	1.25

1pt275	199.6	199.9	0.3	35.9	-2.7	0.24
1pt276	199.9	201.3	1.4	39.1	3.3	1.26
1pt277	201.3	201.6	0.3	42.4	3.3	1.44
1pt278	201.6	203.7	2.1	39.5	-2.9	2.24
1pt279	203.7	204.2	0.5	44.7	5.2	1.44
1pt280	204.2	204.5	0.3	46.8	2.1	0.32
1pt281	204.5	205.5	1	42.9	-3.9	1.53
1pt282	205.5	206	0.5	37.4	-5.5	0.7
1pt283	206	206.4	0.4	42.9	5.5	3.73
1pt284	206.4	206.8	0.4	47.6	4.7	2.35
1pt285	206.8	207	0.2	42.7	-4.9	0
1pt286	207	207.4	0.4	47.7	5.0	1.74
1pt287	207.4	207.7	0.3	45.1	-2.6	0.65
1pt288	207.7	208	0.3	36.9	-8.1	1.71
1pt289	208	208.5	0.5	44.1	7.2	3.88
1pt290	208.5	209.2	0.7	51.7	7.6	2.68
1pt291	209.2	210.7	1.5	43.8	-7.9	1.29
1pt292	210.7	211	0.3	47.2	3.4	1.5
1pt293	211	212	1	43.4	-3.8	2.33
1pt294	212	212.2	0.2	39.5	-3.9	0
1pt295	212.2	212.6	0.4	42.4	2.9	0.51
1pt296	212.6	212.8	0.2	40.5	-1.9	0
1pt297	212.8	213.7	0.9	42.2	1.8	1.41
1pt298	213.7	214.7	1	40.0	-2.2	1.08
1pt299	214.7	215.4	0.7	42.6	2.6	1.53
1pt300	215.4	215.9	0.5	47.0	4.4	0.99
1pt301	215.9	216.5	0.6	42.9	-4.1	3.14
1pt302	216.5	216.7	0.2	37.4	-5.5	0
1pt303	216.7	218	1.3	42.9	5.4	2.19
1pt304	218	219.3	1.3	38.3	-4.5	1.37
1pt305	219.3	220.1	0.8	36.0	-2.3	0.96
1pt306	220.1	221.5	1.4	39.1	3.0	1.47
1pt307	221.5	221.7	0.2	41.5	2.4	0
1pt308	221.7	222.8	1.1	39.0	-2.4	0.98
1pt309	222.8	223.1	0.3	43.1	4.1	2.23
1pt310	223.1	223.8	0.7	40.0	-3.1	0.47
1pt311	223.8	224.1	0.3	42.3	2.3	1.03
1pt312	224.1	224.9	0.8	37.9	-4.4	2.08
1pt313	224.9	225.4	0.5	43.5	5.6	1.21
1pt314	225.4	225.6	0.2	39.4	-4.1	0
1pt315	225.6	227.1	1.5	43.6	4.2	1.52
1pt316	227.1	227.5	0.4	39.3	-4.3	0.73
1pt317	227.5	228.6	1.1	43.2	4.0	2.26
1pt318	228.6	230	1.4	37.4	-5.8	2.6
2apt1	0	1.4	1.4	43.6		1.51
2apt2	1.4	1.8	0.4	49.3	5.6	1.07
2apt3	1.8	2.6	0.8	42.9	-6.4	2.39
2apt4	2.6	3.2	0.6	47.4	4.5	3.48
2apt5	3.2	3.7	0.5	43.0	-4.4	1.43
2apt6	3.7	4.5	0.8	37.5	-5.5	0.49
2apt7	4.5	4.7	0.2	43.0	5.5	0
2apt8	4.7	5.1	0.4	48.4	5.4	1.57
2apt9	5.1	6.9	1.8	40.0	-8.4	1.73
2apt10	6.9	7.3	0.4	43.2	3.2	1.02
2apt11	7.3	8.1	0.8	40.2	-3.0	0.94

2apt12	8.1	8.6	0.5	42.8	2.6	1.22
2apt13	8.6	8.9	0.3	47.9	5.0	1.61
2apt14	8.9	9.3	0.4	40.6	-7.2	2.17
2apt15	9.3	9.6	0.3	46.5	5.9	0.08
2apt16	9.6	9.8	0.2	42.3	-4.2	0
2apt17	9.8	10	0.2	48.5	6.2	0
2apt18	10	10.2	0.2	42.6	-5.9	0
2apt19	10.2	11.4	1.2	48.5	6.0	3.27
2apt20	11.4	11.6	0.2	38.3	-10.3	0
2apt21	11.6	11.8	0.2	44.8	6.6	0
2apt22	11.8	12	0.2	48.3	3.4	0
2apt23	12	12.4	0.4	43.2	-5.1	1.76
2apt24	12.4	13.2	0.8	40.1	-3.1	1.36
2apt25	13.2	14.5	1.3	35.5	-4.6	0.55
2apt26	14.5	15.9	1.4	39.1	3.6	1.46
2apt27	15.9	17.1	1.2	44.2	5.1	2.41
2apt28	17.1	17.7	0.6	38.7	-5.5	1.49
2apt29	17.7	17.9	0.2	36.2	-2.4	0
2apt30	17.9	20	2.1	39.5	3.2	1.46
2apt31	20	20.8	0.8	43.9	4.4	3.66
2apt32	20.8	21.1	0.3	49.4	5.5	2.53
2apt33	21.1	21.3	0.2	38.4	-11.0	0
2apt34	21.3	21.5	0.2	44.7	6.3	0
2apt35	21.5	23.5	2	37.9	-6.8	1.63
2apt36	23.5	23.8	0.3	42.7	4.8	0.35
2apt37	23.8	24.2	0.4	49.6	6.9	3.19
2apt38	24.2	24.4	0.2	38.4	-11.2	0
2apt39	24.4	25	0.6	42.7	4.2	2.04
2apt40	25	25.3	0.3	37.5	-5.2	1.28
2apt41	25.3	26	0.7	47.9	10.4	3
2apt42	26	26.2	0.2	40.5	-7.4	0
2apt43	26.2	27	0.8	43.2	2.7	2.93
2apt44	27	27.4	0.4	50.1	6.9	0.47
2apt45	27.4	27.6	0.2	45.5	-4.6	0
2apt46	27.6	28.1	0.5	48.8	3.3	1.86
2apt47	28.1	28.4	0.3	42.9	-5.9	2.25
2apt48	28.4	28.8	0.4	38.5	-4.5	1.75
2apt49	28.8	29.3	0.5	46.5	8.0	3.33
2apt50	29.3	31.1	1.8	43.4	-3.0	2.44
2apt51	31.1	31.3	0.2	37.7	-5.7	0
2apt52	31.3	32.1	0.8	44.9	7.2	1.92
2apt53	32.1	33.6	1.5	39.5	-5.4	1.91
2apt54	33.6	33.8	0.2	42.2	2.7	0
2apt55	33.8	35.1	1.3	39.5	-2.7	1.27
2apt56	35.1	36.9	1.8	35.9	-3.6	1.12
2apt57	36.9	38.9	2	39.7	3.8	1.42
2apt58	38.9	39.7	0.8	42.8	3.1	2.56
2apt59	39.7	41.4	1.7	39.1	-3.7	1.38
2apt60	41.4	42.5	1.1	35.4	-3.7	0.95
2apt61	42.5	42.8	0.3	38.6	3.2	1.43
2apt62	42.8	43.8	1	42.9	4.3	2.99
2apt63	43.8	44.3	0.5	49.3	6.4	1.27
2apt64	44.3	45	0.7	39.5	-9.7	1.81
2apt65	45	45.2	0.2	42.7	3.2	0
2apt66	45.2	45.8	0.6	38.2	-4.5	1.43
2apt67	45.8	46.4	0.6	45.2	7.0	2.55

2apt68	46.4	46.7	0.3	37.5	-7.7	1.64
2apt69	46.7	47.7	1	43.9	6.4	1.31
2apt70	47.7	47.9	0.2	39.6	-4.3	0
2apt71	47.9	49	1.1	45.8	6.2	2.94
2apt72	49	50	1	40.0	-5.8	1.26
2apt73	50	50.2	0.2	41.4	1.4	0
2apt74	50.2	50.8	0.6	37.5	-3.9	1.13
2apt75	50.8	54.5	3.7	35.8	-1.7	0.94
2apt76	54.5	55.8	1.3	39.4	3.6	1.4
2apt77	55.8	56	0.2	43.0	3.6	0
2apt78	56	57.8	1.8	39.3	-3.6	1.58
2apt79	57.8	59.6	1.8	35.5	-3.8	0.81
2apt80	59.6	59.9	0.3	37.4	1.9	0.21
2apt81	59.9	60.2	0.3	36.8	-0.6	0.09
2apt82	60.2	61.6	1.4	38.7	1.9	1.58
2apt83	61.6	62.2	0.6	43.8	5.2	1.05
2apt84	62.2	62.8	0.6	39.5	-4.4	1.48
2apt85	62.8	63.3	0.5	42.8	3.3	0.93
2apt86	63.3	63.5	0.2	40.3	-2.5	0
2apt87	63.5	63.9	0.4	44.3	3.9	0.27
2apt88	63.9	64.2	0.3	39.0	-5.2	1.36
2apt89	64.2	64.7	0.5	37.0	-2.1	1.37
2apt90	64.7	65.6	0.9	38.0	1.0	1.28
2apt91	65.6	67.4	1.8	42.8	4.9	1.95
2apt92	67.4	70.1	2.7	38.9	-3.9	1.47
2apt93	70.1	70.4	0.3	41.6	2.7	0.85
2apt94	70.4	70.6	0.2	39.9	-1.7	0
2apt95	70.6	72.4	1.8	43.2	3.3	1.74
2apt96	72.4	72.6	0.2	47.2	4.0	0
2apt97	72.6	72.8	0.2	43.3	-3.9	0
2apt98	72.8	73	0.2	38.7	-4.6	0
2apt99	73	73.8	0.8	46.7	8.0	2.97
2apt100	73.8	74.2	0.4	36.4	-10.2	0.87
2apt101	74.2	74.4	0.2	37.4	0.9	0
2apt102	74.4	74.9	0.5	48.2	10.8	2.49
2apt103	74.9	75.2	0.3	39.1	-9.1	1.37
2apt104	75.2	76	0.8	44.6	5.6	1.47
2apt105	76	76.2	0.2	49.6	4.9	0
2apt106	76.2	76.7	0.5	42.8	-6.8	2
2apt107	76.7	77.6	0.9	39.2	-3.6	1.42
2apt108	77.6	80.7	3.1	35.3	-3.8	0.99
2apt109	80.7	82.7	2	38.6	3.2	1.34
2apt110	82.7	85.8	3.1	35.5	-3.1	0.93
2apt111	85.8	86.7	0.9	38.8	3.4	1.2
2apt112	86.7	87.1	0.4	44.3	5.5	1.68
2apt113	87.1	87.7	0.6	48.2	3.9	1.43
2apt114	87.7	88.3	0.6	45.0	-3.2	1.69
2apt115	88.3	89	0.7	40.5	-4.5	1.66
2apt116	89	89.6	0.6	43.5	3.0	1.35
2apt117	89.6	90.1	0.5	40.3	-3.2	1.29
2apt118	90.1	91.8	1.7	40.6	0.3	5.64
2apt119	91.8	92.3	0.5	42.4	1.8	3.48
2apt120	92.3	95.5	3.2	39.5	-2.9	0
2apt121	95.5	95.7	0.2	37.3	-2.2	0
2apt122	95.7	96.9	1.2	43.8	6.5	4.84
2apt123	96.9	97.9	1	48.4	4.6	2.96

2apt124	97.9	98.3	0.4	41.4	-7.0	4.32
2apt125	98.3	98.5	0.2	38.9	-2.4	0
2apt126	98.5	98.7	0.2	46.2	7.3	0
2apt127	98.7	98.9	0.2	38.6	-7.6	0
2apt128	98.9	99.9	1	43.3	4.7	2.33
2apt129	99.9	100.5	0.6	39.5	-3.9	1.67
2apt130	100.5	100.9	0.4	42.2	2.8	2.23
2apt131	100.9	101.1	0.2	40.3	-2.0	0
2apt132	101.1	102.8	1.7	44.0	3.7	1.64
2apt133	102.8	103	0.2	40.3	-3.7	0
2apt134	103	103.4	0.4	42.6	2.2	1.8
2apt135	103.4	104.4	1	38.9	-3.6	1.52
2apt136	104.4	105.4	1	35.8	-3.1	0.64
2apt137	105.4	105.8	0.4	39.6	3.9	0.64
2apt138	105.8	108	2.2	42.8	3.2	1.98
2apt139	108	109.8	1.8	38.7	-4.1	1.23
2apt140	109.8	110.2	0.4	41.1	2.3	2.31
2apt141	110.2	110.9	0.7	47.5	6.5	1.83
2apt142	110.9	113.2	2.3	43.1	-4.4	2.31
2apt143	113.2	113.4	0.2	38.9	-4.2	0
2apt144	113.4	114	0.6	43.7	4.8	1.65
2apt145	114	114.2	0.2	48.2	4.5	0
2apt146	114.2	114.5	0.3	42.8	-5.4	2.05

2bpt1	0	114	114	0.0		0
2bpt2	114	114.2	0.2	41.8	41.8	0
2bpt3	114.2	115.4	1.2	39.3	-2.5	1.36
2bpt4	115.4	117.6	2.2	36.3	-3.0	0.87
2bpt5	117.6	117.8	0.2	37.7	1.4	0
2bpt6	117.8	118	0.2	36.5	-1.1	0
2bpt7	118	118.2	0.2	38.4	1.9	0
2bpt8	118.2	118.6	0.4	41.4	3.0	0.37
2bpt9	118.6	119.1	0.5	41.0	-0.5	1.05
2bpt10	119.1	119.7	0.6	43.4	2.4	2.25
2bpt11	119.7	120.4	0.7	45.4	2.0	3.38
2bpt12	120.4	120.8	0.4	37.7	-7.8	1.69
2bpt13	120.8	122	1.2	49.6	11.9	2.31
2bpt14	122	122.9	0.9	43.0	-6.6	1.78
2bpt15	122.9	123.6	0.7	38.4	-4.7	1.48
2bpt16	123.6	124.7	1.1	35.9	-2.4	0.74
2bpt17	124.7	125.7	1	38.8	2.9	0.45
2bpt18	125.7	126.3	0.6	36.2	-2.7	0.67
2bpt19	126.3	126.6	0.3	37.7	1.6	0.12
2bpt20	126.6	126.9	0.3	36.7	-1.0	0.16
2bpt21	126.9	127.4	0.5	38.6	2.0	0.61
2bpt22	127.4	127.8	0.4	45.3	6.6	0.67
2bpt23	127.8	128	0.2	49.5	4.2	0
2bpt24	128	128.4	0.4	43.6	-5.9	2.5
2bpt25	128.4	129.1	0.7	45.5	1.9	3.98
2bpt26	129.1	129.6	0.5	49.2	3.7	1.53
2bpt27	129.6	131.1	1.5	42.4	-6.8	2.25
2bpt28	131.1	131.6	0.5	46.5	4.1	2.89
2bpt29	131.6	132.6	1	43.3	-3.2	3.43
2bpt30	132.6	132.8	0.2	65.1	21.8	0
2bpt31	132.8	133.1	0.3	37.2	-27.9	0.3
2bpt32	133.1	136	2.9	0.0	-37.2	0

2bpt33	136	136.5	0.5	39.2	39.2	2.12
2bpt34	136.5	136.8	0.3	42.6	3.5	0.82
2bpt35	136.8	138.1	1.3	39.7	-3.0	1.15
2bpt36	138.1	139	0.9	42.9	3.2	1.48
2bpt37	139	139.9	0.9	38.9	-4.0	1.02
2bpt38	139.9	140.7	0.8	42.1	3.2	1.44
2bpt39	140.7	143.2	2.5	38.0	-4.1	0.97
2bpt40	143.2	148	4.8	35.5	-2.5	1.03
2bpt41	148	148.4	0.4	38.1	2.5	0.73
2bpt42	148.4	148.6	0.2	36.2	-1.9	0
2bpt43	148.6	149	0.4	38.3	2.0	0.65
2bpt44	149	151.4	2.4	35.8	-2.5	0.77
2bpt45	151.4	152.2	0.8	38.3	2.5	1.1
2bpt46	152.2	152.6	0.4	35.8	-2.5	0.67
2bpt47	152.6	153.2	0.6	38.3	2.6	2.77
2bpt48	153.2	153.6	0.4	42.2	3.9	0.62
2bpt49	153.6	158.1	4.5	38.7	-3.5	1.34
2bpt50	158.1	160.9	2.8	35.3	-3.5	0.99
2bpt51	160.9	161.1	0.2	40.5	5.2	0
2bpt52	161.1	161.9	0.8	36.4	-4.1	0.62
2bpt53	161.9	163.3	1.4	38.5	2.1	1.36
2bpt54	163.3	163.9	0.6	43.4	4.8	1.16
2bpt55	163.9	164.3	0.4	36.7	-6.7	1.4
2bpt56	164.3	166.3	2	38.4	1.7	1.31
2bpt57	166.3	166.7	0.4	35.2	-3.3	0.69
2bpt58	166.7	166.9	0.2	38.9	3.7	0
2bpt59	166.9	170.4	3.5	36.0	-2.9	0.78
2bpt60	170.4	170.6	0.2	37.5	1.5	0
2bpt61	170.6	172.5	1.9	36.0	-1.5	0.74
2bpt62	172.5	174.8	2.3	39.4	3.4	1.09
2bpt63	174.8	175	0.2	36.2	-3.3	0
2bpt64	175	175.5	0.5	40.2	4.1	0.58
2bpt65	175.5	175.9	0.4	42.5	2.2	1.05
2bpt66	175.9	177	1.1	39.6	-2.9	1.51
2bpt67	177	177.5	0.5	43.1	3.6	1.12
2bpt68	177.5	178.2	0.7	40.0	-3.1	1.02
2bpt69	178.2	178.4	0.2	42.0	1.9	0
2bpt70	178.4	179.5	1.1	40.1	-1.9	1.79
2bpt71	179.5	179.8	0.3	42.7	2.5	0.92
2bpt72	179.8	181	1.2	38.4	-4.3	1.39
2bpt73	181	181.2	0.2	46.5	8.1	0
2bpt74	181.2	184.5	3.3	38.7	-7.8	1.48
2bpt75	184.5	184.8	0.3	36.7	-2.0	0.51
2bpt76	184.8	185.2	0.4	37.1	0.4	0.91
2bpt77	185.2	186.5	1.3	36.0	-1.1	0.36
2bpt78	186.5	186.9	0.4	37.7	1.7	1.41
2bpt79	186.9	187.7	0.8	36.5	-1.1	0.32
2bpt80	187.7	188.7	1	37.6	1.1	0.86
2bpt81	188.7	191.6	2.9	34.8	-2.8	0.9
2bpt82	191.6	192.2	0.6	37.1	2.4	0.82
2bpt83	192.2	194.7	2.5	35.4	-1.7	0.85
2bpt84	194.7	197.3	2.6	38.7	3.2	1.19
2bpt85	197.3	200.6	3.3	34.4	-4.3	0.91
2bpt86	200.6	201	0.4	38.6	4.2	1.78
2bpt87	201	201.3	0.3	36.7	-1.8	0.23
2bpt88	201.3	202.6	1.3	38.7	2.0	1.18

2bpt89	202.6	203	0.4	41.8	3.1	1.03
2bpt90	203	203.7	0.7	38.1	-3.6	1.03
2bpt91	203.7	204.5	0.8	35.7	-2.4	0.5
2bpt92	204.5	206.1	1.6	39.4	3.7	0.98
2bpt93	206.1	206.8	0.7	41.8	2.4	1.35
2bpt94	206.8	207.3	0.5	39.0	-2.8	0.96
2bpt95	207.3	208.2	0.9	42.9	3.9	1.7
2bpt96	208.2	210.2	2	39.9	-3.0	1.37
2bpt97	210.2	210.7	0.5	36.5	-3.3	0.38
2bpt98	210.7	211.1	0.4	38.1	1.6	0.55
2bpt99	211.1	211.9	0.8	42.9	4.9	1.36
2bpt100	211.9	213.2	1.3	39.6	-3.4	1.2
2bpt101	213.2	213.9	0.7	42.7	3.1	1.06
2bpt102	213.9	214.6	0.7	37.5	-5.2	0.65
2bpt103	214.6	215.4	0.8	36.2	-1.3	0.59
2bpt104	215.4	216.2	0.8	37.7	1.5	0.92
2bpt105	216.2	218.5	2.3	35.3	-2.4	1.1
2bpt106	218.5	218.7	0.2	37.9	2.6	0
2bpt107	218.7	220.1	1.4	35.8	-2.1	0.8
2bpt108	220.1	221.7	1.6	38.4	2.6	1.46
2bpt109	221.7	223	1.3	43.6	5.2	2.24
2bpt110	223	223.4	0.4	40.5	-3.1	0.86
2bpt111	223.4	223.7	0.3	43.2	2.7	1.87
2bpt112	223.7	225.6	1.9	48.9	5.7	4.02
2bpt113	225.6	226.1	0.5	42.3	-6.5	0.95
2bpt114	226.1	229.7	3.6	39.2	-3.1	1.32
2bpt115	229.7	230.2	0.5	42.3	3.1	1.16
2bpt116	230.2	231.9	1.7	38.8	-3.5	1.96
2bpt117	231.9	232.3	0.4	36.3	-2.4	0.67
2bpt118	232.3	233.5	1.2	39.7	3.3	1.28
2bpt119	233.5	234	0.5	41.3	1.6	2.14
2bpt120	234	235.7	1.7	38.5	-2.8	1.33
2bpt121	235.7	235.9	0.2	41.9	3.4	0
2bpt122	235.9	236.1	0.2	39.0	-2.9	0
2bpt123	236.1	236.4	0.3	42.7	3.7	0.46
2bpt124	236.4	236.8	0.4	40.6	-2.1	0.38
2bpt125	236.8	237	0.2	43.6	3.1	0
2bpt126	237	238	1	46.9	3.3	3.38
2bpt127	238	238.2	0.2	45.2	-1.7	0
2bpt128	238.2	238.4	0.2	39.3	-5.9	0
2bpt129	238.4	239.7	1.3	47.7	8.4	3.92
2bpt130	239.7	239.9	0.2	40.3	-7.4	0
2bpt131	239.9	241.7	1.8	44.2	3.9	2.04
2bpt132	241.7	242	0.3	48.0	3.8	0.55
2bpt133	242	242.3	0.3	45.9	-2.1	0.1
2bpt134	242.3	242.5	0.2	48.1	2.2	0
2bpt135	242.5	242.8	0.3	43.5	-4.6	1.62
2bpt136	242.8	243.1	0.3	47.0	3.4	0.76
2bpt137	243.1	243.7	0.6	43.8	-3.2	1.18
2bpt138	243.7	244	0.3	48.9	5.1	2.1
2bpt139	244	244.4	0.4	45.6	-3.3	1.79
2bpt140	244.4	244.9	0.5	49.6	4.0	3.65
2bpt141	244.9	245.1	0.2	44.9	-4.7	0
2bpt142	245.1	247.1	2	50.1	5.2	2.87
2bpt143	247.1	247.4	0.3	56.4	6.4	3.1
2bpt144	247.4	248.1	0.7	49.3	-7.1	3.59

2bpt145	248.1	248.7	0.6	51.6	2.3	5.41
3pt1	0	0.3	0.3	38.2		0
3pt2	0.3	1.7	1.4	36.3	-1.9	0.67
3pt3	1.7	4.6	2.9	38.6	2.3	1.33
3pt4	4.6	5.5	0.9	42.7	4.2	1.81
3pt5	5.5	8.7	3.2	38.2	-4.5	1.19
3pt6	8.7	9.3	0.6	43.7	5.5	1.78
3pt7	9.3	9.5	0.2	46.4	2.7	0
3pt8	9.5	9.7	0.2	42.3	-4.1	0
3pt9	9.7	9.9	0.2	43.5	1.2	0
3pt10	9.9	11.4	1.5	48.1	4.6	2.01
3pt11	11.4	13	1.6	43.1	-5.0	2.49
3pt12	13	14.2	1.2	51.2	8.1	2.15
3pt13	14.2	14.6	0.4	43.3	-7.9	2.5
3pt14	14.6	15.3	0.7	49.0	5.7	3.24
3pt15	15.3	15.9	0.6	43.6	-5.4	2.37
3pt16	15.9	16.4	0.5	39.6	-3.9	1.84
3pt17	16.4	16.9	0.5	43.1	3.5	1.66
3pt18	16.9	17.7	0.8	39.8	-3.3	1.84
3pt19	17.7	18.1	0.4	36.0	-3.9	0.63
3pt20	18.1	18.7	0.6	38.9	2.9	1
3pt21	18.7	19	0.3	36.5	-2.3	0.45
3pt22	19	19.3	0.3	37.5	1.0	0.54
3pt23	19.3	19.9	0.6	36.2	-1.3	0.83
3pt24	19.9	21	1.1	38.9	2.7	1.16
3pt25	21	23.4	2.4	36.4	-2.5	0.74
3pt26	23.4	24.2	0.8	38.4	2.0	0.76
3pt27	24.2	24.6	0.4	42.4	4.0	1.36
3pt28	24.6	28.1	3.5	38.3	-4.1	1.23
3pt29	28.1	28.3	0.2	42.6	4.3	0
3pt30	28.3	29.6	1.3	37.9	-4.7	1.6
3pt31	29.6	30.7	1.1	36.4	-1.5	0.39
3pt32	30.7	32.4	1.7	38.5	2.1	1.35
3pt33	32.4	34	1.6	43.0	4.5	1.25
3pt34	34	34.3	0.3	40.2	-2.9	1.69
3pt35	34.3	34.5	0.2	36.4	-3.8	0
3pt36	34.5	34.7	0.2	38.7	2.2	0
3pt37	34.7	34.9	0.2	41.6	3.0	0
3pt38	34.9	35.7	0.8	38.1	-3.5	1.33
3pt39	35.7	36.9	1.2	36.1	-2.0	0.49
3pt40	36.9	37.5	0.6	38.4	2.3	1.18
3pt41	37.5	38.1	0.6	41.6	3.2	1.91
3pt42	38.1	38.3	0.2	39.4	-2.2	0
3pt43	38.3	38.8	0.5	44.1	4.7	1.2
3pt44	38.8	39	0.2	48.8	4.7	0
3pt45	39	39.6	0.6	44.6	-4.2	3.23
3pt46	39.6	39.8	0.2	40.4	-4.2	0
3pt47	39.8	40.4	0.6	44.0	3.6	2.49
3pt48	40.4	41	0.6	39.3	-4.7	1.39
3pt49	41	41.6	0.6	42.0	2.7	1.11
3pt50	41.6	41.8	0.2	40.7	-1.3	0
3pt51	41.8	42.1	0.3	42.1	1.4	1.31
3pt52	42.1	42.9	0.8	39.4	-2.7	0.98
3pt53	42.9	44.4	1.5	44.0	4.6	1.66
3pt54	44.4	44.7	0.3	39.8	-4.1	0.67

3pt55	44.7	47.2	2.5	43.5	3.6	2.07
3pt56	47.2	47.4	0.2	40.9	-2.6	0
3pt57	47.4	47.7	0.3	44.5	3.6	0.7
3pt58	47.7	49	1.3	46.5	2.0	4.46
3pt59	49	49.2	0.2	42.4	-4.1	0
3pt60	49.2	51.1	1.9	49.0	6.6	3.61
3pt61	51.1	51.3	0.2	44.3	-4.7	0
3pt62	51.3	51.8	0.5	55.9	11.6	0.94
3pt63	51.8	52.4	0.6	40.7	-15.2	4.31
3pt64	52.4	53.2	0.8	44.4	3.8	1.92
3pt65	53.2	53.8	0.6	54.0	9.5	2.63
3pt66	53.8	54	0.2	41.7	-12.2	0
3pt67	54	54.6	0.6	47.1	5.3	4.01
3pt68	54.6	56.3	1.7	42.8	-4.3	1.75
3pt69	56.3	56.6	0.3	40.3	-2.5	0.37
3pt70	56.6	57.1	0.5	42.8	2.5	1.58
3pt71	57.1	58	0.9	39.7	-3.1	1.06
3pt72	58	58.5	0.5	43.8	4.1	2.02
3pt73	58.5	58.9	0.4	39.2	-4.6	1.26
3pt74	58.9	59.1	0.2	41.5	2.2	0
3pt75	59.1	59.3	0.2	39.3	-2.2	0
3pt76	59.3	59.6	0.3	47.1	7.8	1.09
3pt77	59.6	59.9	0.3	44.1	-2.9	0.86
3pt78	59.9	60.1	0.2	47.7	3.6	0
3pt79	60.1	61	0.9	39.2	-8.5	1.41
3pt80	61	61.2	0.2	41.6	2.4	0
3pt81	61.2	62.9	1.7	38.8	-2.8	0.77
3pt82	62.9	63.1	0.2	41.9	3.1	0
3pt83	63.1	65.3	2.2	39.6	-2.3	1.26
3pt84	65.3	66	0.7	41.3	1.7	0.85
3pt85	66	67.4	1.4	39.7	-1.6	0.73
3pt86	67.4	67.7	0.3	42.0	2.3	0.24
3pt87	67.7	67.9	0.2	0.0	-42.0	0
3pt88	67.9	68.1	0.2	38.8	38.8	0
3pt89	68.1	68.6	0.5	42.4	3.6	1.99
3pt90	68.6	71	2.4	38.7	-3.7	0.85
3pt91	71	71.4	0.4	41.3	2.6	0.51
3pt92	71.4	72.1	0.7	38.5	-2.8	1.12
3pt93	72.1	72.5	0.4	36.7	-1.8	0.26
3pt94	72.5	73	0.5	39.1	2.4	1.41
3pt95	73	75.4	2.4	42.8	3.7	1.9
3pt96	75.4	76.6	1.2	38.2	-4.6	1.43
3pt97	76.6	77.2	0.6	36.4	-1.8	0.48
3pt98	77.2	77.8	0.6	44.4	7.9	2.37
3pt99	77.8	80.3	2.5	35.8	-8.5	0.83
3pt100	80.3	80.7	0.4	37.2	1.4	0.63
3pt101	80.7	92.9	12.2	35.3	-1.9	1.27
3pt102	92.9	97.2	4.3	0.0	-35.3	0
3pt103	97.2	98.3	1.1	37.8	37.8	1.07
3pt104	98.3	102	3.7	35.2	-2.6	1.11
3pt105	102	103.1	1.1	37.8	2.6	1.22
3pt106	103.1	103.4	0.3	36.8	-1.0	0.18
3pt107	103.4	105.5	2.1	38.3	1.5	1.21
3pt108	105.5	105.7	0.2	42.4	4.0	0
3pt109	105.7	106.3	0.6	38.9	-3.4	1.08
3pt110	106.3	106.6	0.3	35.8	-3.2	0.62

3pt111	106.6	106.8	0.2	37.3	1.5	0
3pt112	106.8	111.2	4.4	35.5	-1.8	0.93
3pt113	111.2	111.7	0.5	38.5	3.0	0.7
3pt114	111.7	113.4	1.7	38.3	-0.2	1.64
3pt115	113.4	113.6	0.2	42.4	4.1	0
3pt116	113.6	114	0.4	38.0	-4.4	0.11
3pt117	114	115.1	1.1	35.9	-2.1	0.87
3pt118	115.1	117.3	2.2	38.4	2.5	1.5
3pt119	117.3	117.5	0.2	43.3	4.9	0
3pt120	117.5	118.8	1.3	39.4	-3.9	1.2
3pt121	118.8	119.4	0.6	35.2	-4.2	0.87
3pt122	119.4	120.5	1.1	38.1	2.9	0.92
3pt123	120.5	121.2	0.7	36.8	-1.3	0.53
3pt124	121.2	122.1	0.9	37.0	0.3	0.34
3pt125	122.1	122.8	0.7	36.1	-1.0	0.64
3pt126	122.8	123.5	0.7	38.9	2.8	1.33
3pt127	123.5	124	0.5	42.2	3.3	0.9
3pt128	124	125.4	1.4	39.5	-2.7	2.36
3pt129	125.4	125.8	0.4	36.5	-3.0	0.94
3pt130	125.8	126	0.2	41.7	5.3	0
3pt131	126	127.2	1.2	39.5	-2.2	1.5
3pt132	127.2	127.8	0.6	46.6	7.0	3.83
3pt133	127.8	128.2	0.4	45.1	-1.5	3.15
3pt134	128.2	128.4	0.2	39.8	-5.2	0
3pt135	128.4	129.6	1.2	43.6	3.7	1.58
3pt136	129.6	129.8	0.2	38.4	-5.1	0
3pt137	129.8	130.2	0.4	42.8	4.4	2.23
3pt138	130.2	131	0.8	47.2	4.4	2.47
3pt139	131	131.3	0.3	44.9	-2.2	1.02
3pt140	131.3	131.5	0.2	54.6	9.7	0
3pt141	131.5	132.2	0.7	48.3	-6.3	1.85
3pt142	132.2	132.7	0.5	44.0	-4.3	1.86
3pt143	132.7	134.1	1.4	49.2	5.2	3.14
3pt144	134.1	134.3	0.2	39.2	-10.0	0
3pt145	134.3	134.7	0.4	45.9	6.7	3.12
3pt146	134.7	137.9	3.2	38.8	-7.0	1
3pt147	137.9	138.4	0.5	42.6	3.7	0.86
3pt148	138.4	138.6	0.2	47.2	4.6	0
3pt149	138.6	140	1.4	43.5	-3.7	2.25
3pt150	140	140.3	0.3	40.5	-3.0	0.63
3pt151	140.3	140.5	0.2	41.4	0.9	0
3pt152	140.5	141.3	0.8	39.7	-1.7	2.1
3pt153	141.3	141.7	0.4	41.7	1.9	2.22
3pt154	141.7	142.6	0.9	39.3	-2.3	1.19
3pt155	142.6	143.1	0.5	43.4	4.0	3.24
3pt156	143.1	143.4	0.3	39.8	-3.5	1.49
3pt157	143.4	145.3	1.9	42.7	2.9	2.15
3pt158	145.3	145.7	0.4	40.1	-2.6	1.36
3pt159	145.7	146.7	1	42.6	2.5	2.57
3pt160	146.7	147.8	1.1	38.6	-3.9	1.73
3pt161	147.8	148	0.2	42.4	3.8	0
3pt162	148	148.9	0.9	38.6	-3.8	1.1
3pt163	148.9	151.1	2.2	35.3	-3.3	0.98
3pt164	151.1	151.3	0.2	37.6	2.3	0
3pt165	151.3	153.5	2.2	35.7	-1.8	1.52
3pt166	153.5	155.6	2.1	39.0	3.2	1.43

3pt167	155.6	155.8	0.2	42.5	3.5	0
3pt168	155.8	156.9	1.1	38.4	-4.1	1.6
3pt169	156.9	157.8	0.9	36.3	-2.1	0.64
3pt170	157.8	158.3	0.5	39.4	3.0	1.21
3pt171	158.3	158.7	0.4	36.8	-2.6	0.59
3pt172	158.7	159.4	0.7	37.7	0.9	0.64
3pt173	159.4	159.9	0.5	36.3	-1.4	0.45
3pt174	159.9	160.7	0.8	38.7	2.4	0.82
3pt175	160.7	160.9	0.2	41.1	2.5	0
3pt176	160.9	163.2	2.3	39.1	-2.0	1.46
3pt177	163.2	163.6	0.4	35.5	-3.7	0.92
3pt178	163.6	165	1.4	39.3	3.8	1.32
3pt179	165	165.3	0.3	42.8	3.5	1.31
3pt180	165.3	166.6	1.3	38.2	-4.6	1.01
3pt181	166.6	173.2	6.6	35.1	-3.1	1.09
3pt182	173.2	173.5	0.3	37.8	2.7	0.23
3pt183	173.5	174.6	1.1	36.1	-1.8	0.57
3pt184	174.6	175.5	0.9	39.7	3.7	1.68
3pt185	175.5	175.9	0.4	41.8	2.1	0.28
3pt186	175.9	178.2	2.3	39.5	-2.3	1.07
3pt187	178.2	181.8	3.6	35.5	-4.0	0.79
3pt188	181.8	182.5	0.7	38.0	2.5	1.2
3pt189	182.5	183	0.5	42.0	4.0	1.17
3pt190	183	183.3	0.3	39.1	-2.9	0.55
3pt191	183.3	183.8	0.5	36.0	-3.1	0.46
3pt192	183.8	185	1.2	38.6	2.6	0.93
3pt193	185	185.2	0.2	42.1	3.5	0
3pt194	185.2	185.7	0.5	37.8	-4.3	0.63
3pt195	185.7	186.1	0.4	37.0	-0.8	0.81
3pt196	186.1	186.8	0.7	37.9	0.9	0.83
3pt197	186.8	187	0.2	36.5	-1.5	0
3pt198	187	188.5	1.5	38.9	2.4	1.79
3pt199	188.5	189.4	0.9	42.8	3.9	1.44
3pt200	189.4	190.4	1	48.1	5.3	3.06
3pt201	190.4	191	0.6	39.8	-8.3	1.36
3pt202	191	192.9	1.9	43.2	3.3	1.08
3pt203	192.9	193.2	0.3	40.0	-3.2	0.78
3pt204	193.2	193.9	0.7	42.1	2.1	0.85
3pt205	193.9	196.3	2.4	38.1	-4.0	1.3
3pt206	196.3	198	1.7	36.6	-1.5	0.68
3pt207	198	199.4	1.4	39.0	2.4	1.44
3pt208	199.4	199.7	0.3	44.7	5.7	0.71
3pt209	199.7	201.1	1.4	46.3	1.5	3.76
3pt210	201.1	201.3	0.2	42.3	-4.0	0
3pt211	201.3	201.9	0.6	48.8	6.5	3.73
3pt212	201.9	202.7	0.8	44.8	-4.0	2.7
3pt213	202.7	203	0.3	37.3	-7.5	2.11
3pt214	203	203.4	0.4	47.9	10.6	2.05
3pt215	203.4	204	0.6	42.3	-5.6	2.47
4pt1	0	0.3	0.3	39.8		0
4pt2	0.3	0.5	0.2	41.1	1.3	0
4pt3	0.5	0.7	0.2	50.0	8.9	0
4pt4	0.7	1.9	1.2	55.4	5.4	4.06
4pt5	1.9	3.5	1.6	48.5	-6.9	2.93
4pt6	3.5	4	0.5	55.6	7.1	1.7

4pt7	4	4.5	0.5	46.3	-9.3	2.3
4pt8	4.5	4.7	0.2	43.1	-3.2	0
4pt9	4.7	5	0.3	47.1	4.0	1.7
4pt10	5	5.8	0.8	43.4	-3.7	1.97
4pt11	5.8	6.4	0.6	48.5	5.1	1.03
4pt12	6.4	6.8	0.4	52.1	3.6	2.35
4pt13	6.8	8.2	1.4	50.4	-1.7	3.1
4pt14	8.2	8.6	0.4	54.2	3.8	1.85
4pt15	8.6	8.8	0.2	0.0		0
4pt16	8.8	9.1	0.3	54.5		0
4pt17	9.1	10.4	1.3	44.4	-10.0	2.76
4pt18	10.4	11.8	1.4	39.1	-5.3	1.22
4pt19	11.8	12.2	0.4	36.5	-2.6	0.31
4pt20	12.2	15.8	3.6	39.2	2.6	1.32
4pt21	15.8	16.1	0.3	43.3	4.2	0.63
4pt22	16.1	17.3	1.2	40.1	-3.2	0.67
4pt23	17.3	17.7	0.4	43.6	3.5	1.58
4pt24	17.7	19.1	1.4	37.6	-6.0	1.26
4pt25	19.1	20.6	1.5	36.2	-1.4	0.67
4pt26	20.6	23.3	2.7	38.1	1.9	0.93
4pt27	23.3	23.8	0.5	36.6	-1.5	0.73
4pt28	23.8	24.7	0.9	40.1	3.6	1.43
4pt29	24.7	26.7	2	42.8	2.7	1.76
4pt30	26.7	27.6	0.9	38.9	-3.9	2.19
4pt31	27.6	37.4	9.8	35.1	-3.8	0.98
4pt32	37.4	37.8	0.4	39.0	3.9	0.65
4pt33	37.8	39.3	1.5	42.0	3.0	0.86
4pt34	39.3	39.7	0.4	39.1	-2.8	0.89
4pt35	39.7	41.4	1.7	42.6	3.5	1.78
4pt36	41.4	41.6	0.2	40.6	-2.0	0
4pt37	41.6	41.9	0.3	41.4	0.8	0.33
4pt38	41.9	42.1	0.2	39.5	-1.9	0
4pt39	42.1	42.3	0.2	41.4	1.9	0
4pt40	42.3	42.5	0.2	38.2	-3.2	0
4pt41	42.5	42.8	0.3	42.4	4.2	0.4
4pt42	42.8	43.5	0.7	37.9	-4.5	0.65
4pt43	43.5	45.2	1.7	35.9	-1.9	0.7
4pt44	45.2	45.4	0.2	37.5	1.5	0
4pt45	45.4	45.6	0.2	36.2	-1.3	0
4pt46	45.6	47.1	1.5	39.0	2.9	1.4
4pt47	47.1	48	0.9	41.6	2.6	1.62
4pt48	48	49.4	1.4	38.9	-2.7	0.96
4pt49	49.4	50	0.6	35.5	-3.4	0.83
4pt50	50	50.3	0.3	38.2	2.7	1.32
4pt51	50.3	51.6	1.3	36.0	-2.2	1.12
4pt52	51.6	52.8	1.2	39.0	2.9	1.18
4pt53	52.8	54	1.2	41.6	2.6	1.02
4pt54	54	55.8	1.8	39.8	-1.8	1.18
4pt55	55.8	56.5	0.7	36.8	-2.9	0.48
4pt56	56.5	58	1.5	37.8	1.0	1.08
4pt57	58	59.2	1.2	36.7	-1.1	1.38
4pt58	59.2	60.2	1	39.2	2.5	1.32
4pt59	60.2	62.3	2.1	35.8	-3.5	1.07
4pt60	62.3	63.2	0.9	37.4	1.6	1.01
4pt61	63.2	72.7	9.5	34.9	-2.5	1.07
4pt62	72.7	73.3	0.6	38.8	3.9	1.1

4pt63	73.3	73.9	0.6	43.3	4.5	1.33
4pt64	73.9	75.9	2	39.7	-3.6	1.07
4pt65	75.9	77.1	1.2	41.5	1.8	1.3
4pt66	77.1	77.7	0.6	38.8	-2.7	1.14
4pt67	77.7	78.1	0.4	42.1	3.3	1.21
4pt68	78.1	79.1	1	40.1	-2.0	1.98
4pt69	79.1	79.3	0.2	36.7	-3.5	0
4pt70	79.3	82.2	2.9	0.0	-36.7	0
4pt71	82.2	82.4	0.2	44.5	44.5	0
4pt72	82.4	82.6	0.2	0.0	-44.5	0
4pt73	82.6	82.9	0.3	40.1	40.1	3.51
4pt74	82.9	83.5	0.6	36.5	-3.6	0.35
4pt75	83.5	85.4	1.9	38.7	2.2	1.52
4pt76	85.4	87.9	2.5	35.6	-3.0	0.94
4pt77	87.9	88.5	0.6	37.5	1.9	0.74
4pt78	88.5	89.2	0.7	36.6	-0.9	0.24
4pt79	89.2	89.5	0.3	38.0	1.4	0.59
4pt80	89.5	89.7	0.2	35.9	-2.2	0
4pt81	89.7	91.1	1.4	39.8	3.9	1.18
4pt82	91.1	91.3	0.2	41.3	1.6	0
4pt83	91.3	92.6	1.3	38.5	-2.9	1.05
4pt84	92.6	97.1	4.5	35.2	-3.2	1.07
4pt85	97.1	97.9	0.8	37.8	2.5	0.89
4pt86	97.9	98.2	0.3	36.3	-1.5	0.53
4pt87	98.2	98.4	0.2	39.0	2.7	0
4pt88	98.4	101.6	3.2	35.9	-3.2	0.81
4pt89	101.6	101.8	0.2	41.6	5.7	0
4pt90	101.8	103.6	1.8	38.7	-2.8	1.17
4pt91	103.6	104	0.4	35.8	-3.0	0.89
4pt92	104	104.3	0.3	37.2	1.4	0.16
4pt93	104.3	104.6	0.3	35.5	-1.7	0.95
4pt94	104.6	105	0.4	37.4	2.0	1.06
4pt95	105	105.4	0.4	35.5	-1.9	0.98
4pt96	105.4	106.9	1.5	37.8	2.3	1.09
4pt97	106.9	109.2	2.3	36.0	-1.8	0.96
4pt98	109.2	109.5	0.3	38.7	2.7	0.37
4pt99	109.5	109.8	0.3	35.5	-3.2	0.86
4pt100	109.8	110	0.2	37.2	1.7	0
4pt101	110	110.7	0.7	35.9	-1.3	0.9
4pt102	110.7	112.5	1.8	39.2	3.3	1.55
4pt103	112.5	114.2	1.7	39.2	0.1	1.74
4pt104	114.2	115.4	1.2	35.6	-3.6	0.68
4pt105	115.4	116.1	0.7	38.9	3.3	1.43
4pt106	116.1	116.3	0.2	41.5	2.6	0
4pt107	116.3	116.5	0.2	38.2	-3.3	0
4pt108	116.5	121.9	5.4	35.3	-2.9	1.33
4pt109	121.9	123	1.1	38.3	3.0	2
4pt110	123	124.2	1.2	36.4	-1.9	0.49
4pt111	124.2	125.6	1.4	38.0	1.7	1.07
4pt112	125.6	125.9	0.3	35.7	-2.4	0.95
4pt113	125.9	126.4	0.5	38.9	3.3	0.56
4pt114	126.4	126.6	0.2	36.3	-2.6	0
4pt115	126.6	127.2	0.6	39.0	2.7	1.37
4pt116	127.2	130.8	3.6	35.7	-3.3	0.97
4pt117	130.8	131.1	0.3	37.9	2.3	0.68
4pt118	131.1	131.3	0.2	36.8	-1.2	0

4pt119	131.3	132	0.7	39.6	2.8	1.61
4pt120	132	132.4	0.4	41.6	2.0	0.42
4pt121	132.4	132.8	0.4	36.4	-5.2	0.73
4pt122	132.8	133	0.2	37.9	1.6	0
4pt123	133	142	9	34.7	-3.2	1.01
4pt124	142	142.6	0.6	38.9	4.2	1.29
4pt125	142.6	143	0.4	41.6	2.6	1.86
4pt126	143	143.2	0.2	39.9	-1.7	0
4pt127	143.2	144.1	0.9	41.7	1.9	1.21
4pt128	144.1	144.4	0.3	38.2	-3.5	1.32
4pt129	144.4	144.6	0.2	41.4	3.1	0
4pt130	144.6	145.2	0.6	38.5	-2.9	0.97
4pt131	145.2	146.6	1.4	36.7	-1.8	0.58
4pt132	146.6	150.8	4.2	38.3	1.6	1.45
4pt133	150.8	151	0.2	36.4	-1.9	0
4pt134	151	153.3	2.3	38.6	2.2	1.65
4pt135	153.3	154	0.7	35.8	-2.8	0.44
4pt136	154	154.4	0.4	39.5	3.6	1.78
4pt137	154.4	155.1	0.7	35.7	-3.8	0.87
4pt138	155.1	155.5	0.4	41.5	5.8	1.31
4pt139	155.5	156	0.5	40.4	-1.1	1.84
4pt140	156	156.4	0.4	42.8	2.4	0.56
4pt141	156.4	156.7	0.3	37.2	-5.6	2.04
4pt142	156.7	156.9	0.2	42.0	4.8	0
4pt143	156.9	157.1	0.2	38.6	-3.4	0
4pt144	157.1	157.7	0.6	42.6	4.0	2.16
4pt145	157.7	159.3	1.6	39.0	-3.7	1.34
4pt146	159.3	159.6	0.3	36.0	-3.0	0.48
4pt147	159.6	160.2	0.6	37.4	1.4	1.09
4pt148	160.2	162.4	2.2	35.7	-1.7	0.69
4pt149	162.4	164	1.6	38.9	3.2	1.75
4pt150	164	167.3	3.3	34.9	-4.0	1.04
4pt151	167.3	167.9	0.6	37.6	2.7	0.59
4pt152	167.9	168.8	0.9	35.5	-2.1	0.78
4pt153	168.8	169.2	0.4	38.7	3.2	0.77
4pt154	169.2	169.4	0.2	43.2	4.6	0
4pt155	169.4	170.2	0.8	37.9	-5.3	1.15
4pt156	170.2	172.7	2.5	35.4	-2.6	0.88
4pt157	172.7	174.2	1.5	39.2	3.8	1.77
4pt158	174.2	174.5	0.3	41.5	2.3	0.33
4pt159	174.5	174.9	0.4	38.5	-3.0	1.12
4pt160	174.9	177.2	2.3	35.4	-3.1	0.95
4pt161	177.2	178.3	1.1	39.1	3.7	1.53
4pt162	178.3	178.8	0.5	36.1	-3.1	1.06
4pt163	178.8	179.1	0.3	37.4	1.4	0.42
4pt164	179.1	180.7	1.6	35.8	-1.6	0.78
4pt165	180.7	181.2	0.5	37.5	1.7	0.94
4pt166	181.2	181.7	0.5	36.0	-1.5	0.82
4pt167	181.7	182.1	0.4	38.9	2.9	0.98
4pt168	182.1	185.1	3	35.4	-3.5	0.81
4pt169	185.1	185.7	0.6	36.9	1.4	0.78
4pt170	185.7	187.4	1.7	38.4	1.6	1.15
4pt171	187.4	189.9	2.5	43.2	4.7	1.96
4pt172	189.9	190.7	0.8	40.4	-2.7	0.78
4pt173	190.7	191.5	0.8	42.1	1.7	1.21
4pt174	191.5	192.7	1.2	39.0	-3.1	0.92

4pt175	192.7	193.1	0.4	42.7	3.6	1.21
4pt176	193.1	194.3	1.2	38.9	-3.8	1.12
4pt177	194.3	194.9	0.6	42.6	3.7	2.14
5pt1	0	1	1	51.5		3.48
5pt2	1	1.6	0.6	55.5	3.9	2.69
5pt3	1.6	2.4	0.8	48.8	-6.6	2.95
5pt4	2.4	4.4	2	43.7	-5.1	1.83
5pt5	4.4	5.3	0.9	39.7	-4.0	1.23
5pt6	5.3	7.1	1.8	43.3	3.6	1.86
5pt7	7.1	10	2.9	39.6	-3.7	1.33
5pt8	10	10.7	0.7	42.9	3.3	1.78
5pt9	10.7	10.9	0.2	47.9	5.0	0
5pt10	10.9	11.2	0.3	43.0	-4.9	1.69
5pt11	11.2	12.2	1	39.1	-3.9	1.56
5pt12	12.2	13.6	1.4	35.5	-3.5	0.74
5pt13	13.6	14.2	0.6	38.9	3.4	0.5
5pt14	14.2	15.3	1.1	43.1	4.1	1.06
5pt15	15.3	16.8	1.5	39.4	-3.7	0.82
5pt16	16.8	18	1.2	43.1	3.7	1.29
5pt17	18	18.9	0.9	36.3	-6.7	1.08
5pt18	18.9	21.2	2.3	38.0	1.6	1.7
5pt19	21.2	21.4	0.2	36.4	-1.6	0
5pt20	21.4	21.6	0.2	37.3	0.9	0
5pt21	21.6	22.1	0.5	37.2	-0.1	3.03
5pt22	22.1	22.5	0.4	37.9	0.7	1.03
5pt23	22.5	24.1	1.6	35.2	-2.7	0.95
5pt24	24.1	24.6	0.5	39.1	3.9	1.48
5pt25	24.6	24.8	0.2	36.6	-2.5	0
5pt26	24.8	25.2	0.4	37.0	0.3	0.67
5pt27	25.2	27.2	2	36.4	-0.6	1.11
5pt28	27.2	27.7	0.5	37.4	1.0	0.98
5pt29	27.7	28.4	0.7	35.9	-1.5	0.43
5pt30	28.4	28.7	0.3	37.7	1.8	0.29
5pt31	28.7	32	3.3	35.2	-2.5	1.02
5pt32	32	32.2	0.2	37.9	2.7	0
5pt33	32.2	32.6	0.4	35.7	-2.1	1.07
5pt34	32.6	34	1.4	38.7	3.0	1.36
5pt35	34	34.2	0.2	35.5	-3.3	0
5pt36	34.2	35.4	1.2	39.7	4.2	1.8
5pt37	35.4	36	0.6	43.0	3.3	1.59
5pt38	36	36.5	0.5	38.9	-4.1	1.96
5pt39	36.5	36.7	0.2	41.8	2.9	0
5pt40	36.7	37	0.3	40.3	-1.5	0.29
5pt41	37	37.3	0.3	43.0	2.6	0.35
5pt42	37.3	37.6	0.3	39.2	-3.8	1.09
5pt43	37.6	37.8	0.2	36.3	-2.9	0
5pt44	37.8	38.9	1.1	40.2	3.8	1.58
5pt45	38.9	39.1	0.2	44.2	4.0	0
5pt46	39.1	39.3	0.2	39.9	-4.3	0
5pt47	39.3	39.6	0.3	42.5	2.6	0.74
5pt48	39.6	41.2	1.6	39.1	-3.4	1.28
5pt49	41.2	42.4	1.2	40.8	1.7	1.2
5pt50	42.4	42.8	0.4	42.9	2.1	0.56
5pt51	42.8	43.3	0.5	39.0	-3.9	1.06
5pt52	43.3	44.4	1.1	41.0	2.0	0.83

5pt53	44.4	45	0.6	37.9	-3.1	2.6
5pt54	45	45.2	0.2	0.0	-37.9	0
5pt55	45.2	45.9	0.7	37.0	37.0	3.44
5pt56	45.9	46.7	0.8	42.0	5.1	0.49
5pt57	46.7	49.2	2.5	39.3	-2.7	1.44
5pt58	49.2	49.4	0.2	41.5	2.2	0
5pt59	49.4	50.2	0.8	38.5	-3.0	1.7
5pt60	50.2	50.5	0.3	35.6	-2.9	0.9
5pt61	50.5	50.7	0.2	40.0	4.4	0
5pt62	50.7	50.9	0.2	35.3	-4.7	0
5pt63	50.9	51.2	0.3	38.6	3.2	0.95
5pt64	51.2	51.4	0.2	35.8	-2.8	0
5pt65	51.4	51.7	0.3	37.9	2.2	0.6
5pt66	51.7	52.6	0.9	36.2	-1.8	0.59
5pt67	52.6	53.9	1.3	39.6	3.4	1.68
5pt68	53.9	54.2	0.3	42.6	3.0	1.16
5pt69	54.2	55.5	1.3	38.7	-3.9	1.66
5pt70	55.5	55.8	0.3	36.4	-2.3	0.05
5pt71	55.8	56.1	0.3	37.7	1.4	0.67
5pt72	56.1	56.3	0.2	36.6	-1.2	0
5pt73	56.3	58.6	2.3	38.4	1.8	1.25
5pt74	58.6	58.8	0.2	42.0	3.6	0
5pt75	58.8	59.2	0.4	39.9	-2.2	1.41
5pt76	59.2	59.9	0.7	42.0	2.1	0.97
5pt77	59.9	61	1.1	39.6	-2.4	1.33
5pt78	61	61.5	0.5	41.6	2.0	0.85
5pt79	61.5	63.4	1.9	38.4	-3.2	1.17
5pt80	63.4	65.5	2.1	35.7	-2.8	1.49
5pt81	65.5	65.7	0.2	38.1	2.4	0
5pt82	65.7	65.9	0.2	36.6	-1.5	0
5pt83	65.9	66.2	0.3	38.0	1.4	0.48
5pt84	66.2	69.1	2.9	0.0	-38.0	0
5pt85	69.1	71.7	2.6	36.3	36.3	1.21
5pt86	71.7	72.1	0.4	38.8	2.5	0.96
5pt87	72.1	72.7	0.6	42.5	3.6	1.02
5pt88	72.7	73.5	0.8	37.8	-4.7	0.81
5pt89	73.5	73.7	0.2	36.5	-1.3	0
5pt90	73.7	75.4	1.7	38.1	1.7	1.37
5pt91	75.4	75.9	0.5	36.3	-1.8	0.46
5pt92	75.9	76.5	0.6	38.2	1.9	1.21
5pt93	76.5	76.7	0.2	35.7	-2.5	0
5pt94	76.7	77.2	0.5	40.9	5.2	0.62
5pt95	77.2	77.5	0.3	42.0	1.1	0.92
5pt96	77.5	77.7	0.2	40.1	-1.9	0
5pt97	77.7	77.9	0.2	43.0	3.0	0
5pt98	77.9	80.9	3	39.1	-4.0	1.9
5pt99	80.9	81.3	0.4	42.4	3.3	0.76
5pt100	81.3	83	1.7	38.8	-3.6	1.98
5pt101	83	84.2	1.2	43.9	5.1	1.91
5pt102	84.2	84.9	0.7	39.1	-4.8	1.51
5pt103	84.9	97.5	12.6	35.1	-3.9	1
5pt104	97.5	98.5	1	38.5	3.4	1.21
5pt105	98.5	99.3	0.8	36.4	-2.1	0.37
5pt106	99.3	100.2	0.9	37.7	1.2	1.1
5pt107	100.2	103.7	3.5	35.5	-2.2	1.18
5pt108	103.7	103.9	0.2	37.6	2.1	0

5pt109	103.9	104.3	0.4	35.7	-1.9	1.45
5pt110	104.3	104.6	0.3	37.7	2.0	0.56
5pt111	104.6	108.4	3.8	34.9	-2.8	1.17
5pt112	108.4	109.1	0.7	38.6	3.7	0.85
5pt113	109.1	109.6	0.5	35.8	-2.7	0.42
5pt114	109.6	110	0.4	39.4	3.5	0.36
5pt115	110	110.4	0.4	35.1	-4.3	0.78
5pt116	110.4	111.6	1.2	38.4	3.3	1.37
5pt117	111.6	112.1	0.5	35.6	-2.8	0.79
5pt118	112.1	112.4	0.3	37.8	2.3	0.93
5pt119	112.4	112.6	0.2	36.2	-1.6	0
5pt120	112.6	114.1	1.5	38.9	2.6	1.47
5pt121	114.1	114.5	0.4	41.9	3.0	1.78
5pt122	114.5	118.6	4.1	38.6	-3.3	1.53
5pt123	118.6	119.8	1.2	35.5	-3.1	0.66
5pt124	119.8	121.2	1.4	38.5	3.0	1.45
5pt125	121.2	123.5	2.3	35.5	-3.0	0.8
5pt126	123.5	125	1.5	38.9	3.4	1.24
5pt127	125	125.2	0.2	35.7	-3.2	0
5pt128	125.2	125.5	0.3	37.4	1.8	0.25
5pt129	125.5	125.7	0.2	36.5	-0.9	0
5pt130	125.7	127.2	1.5	38.7	2.2	1.61
5pt131	127.2	127.5	0.3	36.2	-2.5	0.31
5pt132	127.5	128	0.5	38.0	1.7	0.74
5pt133	128	128.5	0.5	42.9	4.9	0.72
5pt134	128.5	129.9	1.4	38.6	-4.3	1
5pt135	129.9	130.1	0.2	36.5	-2.1	0
5pt136	130.1	130.6	0.5	37.3	0.8	0.61
5pt137	130.6	132.4	1.8	36.2	-1.2	0.78
5pt138	132.4	133.6	1.2	38.8	2.7	1.19
5pt139	133.6	134.1	0.5	46.1	7.3	3.11
5pt140	134.1	134.8	0.7	43.3	-2.8	1.91
5pt141	134.8	135.1	0.3	47.3	4.0	1.89
5pt142	135.1	135.4	0.3	44.0	-3.3	1.89
5pt143	135.4	135.8	0.4	48.3	4.3	1.64
5pt144	135.8	136.1	0.3	42.5	-5.8	2.16
5pt145	136.1	136.7	0.6	45.0	2.6	2.84
5pt146	136.7	136.9	0.2	48.9	3.9	0
5pt147	136.9	137.1	0.2	44.9	-4.0	0
5pt148	137.1	137.3	0.2	47.1	2.2	0
5pt149	137.3	138	0.7	43.4	-3.8	2.42
5pt150	138	138.7	0.7	39.4	-4.0	0.88
5pt151	138.7	139.5	0.8	42.7	3.3	0.85
5pt152	139.5	139.7	0.2	38.9	-3.8	0
5pt153	139.7	140.1	0.4	43.6	4.7	1.78
5pt154	140.1	140.5	0.4	46.6	3.0	1.08
5pt155	140.5	141.1	0.6	43.3	-3.2	1.96
5pt156	141.1	141.7	0.6	49.8	6.4	3.03
5pt157	141.7	142.5	0.8	43.2	-6.6	2.69
5pt158	142.5	142.9	0.4	39.3	-3.8	1.2
5pt159	142.9	143.4	0.5	42.5	3.2	2.24
5pt160	143.4	143.7	0.3	50.3	7.8	0.47
5pt161	143.7	144	0.3	43.8	-6.5	1.56
5pt162	144	144.2	0.2	46.6	2.8	0
5pt163	144.2	145.1	0.9	42.8	-3.8	1.59
5pt164	145.1	146.5	1.4	38.4	-4.4	0.98

5pt165	146.5	147.2	0.7	36.4	-1.9	0.81
5pt166	147.2	147.8	0.6	38.8	2.3	0.83
5pt167	147.8	148	0.2	42.1	3.3	0
5pt168	148	149.9	1.9	39.5	-2.7	1.35
5pt169	149.9	150.2	0.3	35.9	-3.6	0.59
5pt170	150.2	150.8	0.6	38.7	2.8	1.19
5pt171	150.8	152	1.2	44.5	5.8	2.79
5pt172	152	152.7	0.7	50.5	6.0	1.7
5pt173	152.7	153	0.3	39.2	-11.3	0.84
5pt174	153	153.2	0.2	48.4	9.2	0
5pt175	153.2	153.9	0.7	43.9	-4.5	1.92
5pt176	153.9	155.6	1.7	38.7	-5.1	1.38
5pt177	155.6	156.6	1	42.9	4.1	2.11
5pt178	156.6	156.8	0.2	46.3	3.5	0
5pt179	156.8	157.3	0.5	42.0	-4.3	1.7
5pt180	157.3	158.9	1.6	38.7	-3.3	0.79
5pt181	158.9	160.3	1.4	42.6	3.9	1.69
5pt182	160.3	161.4	1.1	39.8	-2.8	0.77
5pt183	161.4	161.6	0.2	42.2	2.4	0
5pt184	161.6	161.8	0.2	40.6	-1.6	0
5pt185	161.8	162.2	0.4	42.6	2.0	1.28
5pt186	162.2	162.4	0.2	47.6	5.0	0
5pt187	162.4	162.8	0.4	42.1	-5.5	0.75
5pt188	162.8	163.4	0.6	38.7	-3.4	1.57
5pt189	163.4	165.3	1.9	36.1	-2.6	0.73
5pt190	165.3	166	0.7	38.3	2.3	0.78
5pt191	166	168.5	2.5	36.0	-2.3	0.67
5pt192	168.5	168.7	0.2	37.8	1.8	0
5pt193	168.7	168.9	0.2	36.5	-1.3	0
5pt194	168.9	170.1	1.2	38.5	2.0	1.06
5pt195	170.1	170.5	0.4	43.0	4.5	1.82
5pt196	170.5	170.7	0.2	46.7	3.7	0
5pt197	170.7	170.9	0.2	42.2	-4.5	0
5pt198	170.9	171.1	0.2	46.6	4.4	0
5pt199	171.1	171.6	0.5	43.8	-2.7	0.75
5pt200	171.6	171.9	0.3	40.0	-3.8	0.43
5pt201	171.9	172.6	0.7	43.7	3.7	1.38
5pt202	172.6	172.9	0.3	47.3	3.6	1.08
5pt203	172.9	173.1	0.2	44.1	-3.3	0
5pt204	173.1	173.5	0.4	37.0	-7.1	0.96
5pt205	173.5	174.3	0.8	44.7	7.7	2.52
5pt206	174.3	175.3	1	47.9	3.2	2.18
5pt207	175.3	175.5	0.2	43.6	-4.2	0
5pt208	175.5	175.7	0.2	47.3	3.7	0
5pt209	175.7	175.9	0.2	44.7	-2.6	0
5pt210	175.9	176.1	0.2	47.2	2.5	0
5pt211	176.1	178	1.9	43.2	-4.1	1.88
5pt212	178	178.3	0.3	48.6	5.4	1.25
5pt213	178.3	178.7	0.4	43.7	-4.8	1.85
5pt214	178.7	178.9	0.2	47.6	3.9	0
5pt215	178.9	179.1	0.2	53.4	5.8	0
5pt216	179.1	179.5	0.4	48.5	-4.9	4.08
5pt217	179.5	179.7	0.2	43.9	-4.7	0
5pt218	179.7	179.9	0.2	55.3	11.4	0
5pt219	179.9	181	1.1	49.1	-6.2	2.95
5pt220	181	181.6	0.6	45.3	-3.8	1.52

5pt221	181.6	181.8	0.2	53.5	8.2	0
5pt222	181.8	182.4	0.6	49.7	-3.8	2.64
5pt223	182.4	182.8	0.4	45.6	-4.1	3.28
5pt224	182.8	183.2	0.4	49.1	3.5	4.03
5pt225	183.2	183.6	0.4	43.3	-5.8	2.44
5pt226	183.6	183.8	0.2	49.1	5.8	0
5pt227	183.8	184	0.2	39.0	-10.1	0
6pt1	0	0.4	0.4	46.5		2.74
6pt2	0.4	1.5	1.1	43.4	-3.1	1.77
6pt3	1.5	1.7	0.2	46.4	3.0	0
6pt4	1.7	2.4	0.7	40.1	-6.3	1.62
6pt5	2.4	3.2	0.8	44.1	4.0	1.28
6pt6	3.2	3.5	0.3	47.4	3.2	1.12
6pt7	3.5	4	0.5	44.8	-2.6	1.08
6pt8	4	4.2	0.2	40.4	-4.4	0
6pt9	4.2	4.8	0.6	42.8	2.4	1.01
6pt10	4.8	5	0.2	40.6	-2.2	0
6pt11	5	5.4	0.4	43.5	2.9	1.87
6pt12	5.4	5.7	0.3	40.1	-3.4	1.09
6pt13	5.7	8.3	2.6	43.1	3.0	2.13
6pt14	8.3	9.4	1.1	37.9	-5.2	1.49
6pt15	9.4	9.6	0.2	36.1	-1.8	0
6pt16	9.6	10.5	0.9	38.3	2.2	1.18
6pt17	10.5	11.6	1.1	42.8	4.5	1.51
6pt18	11.6	11.8	0.2	39.5	-3.3	0
6pt19	11.8	12	0.2	42.4	2.8	0
6pt20	12	13.4	1.4	39.7	-2.7	1.09
6pt21	13.4	15.9	2.5	43.3	3.6	1.62
6pt22	15.9	16.1	0.2	39.9	-3.4	0
6pt23	16.1	17	0.9	42.6	2.7	1.78
6pt24	17	17.5	0.5	40.3	-2.3	0.92
6pt25	17.5	18.7	1.2	41.8	1.5	1.06
6pt26	18.7	20.4	1.7	38.5	-3.3	1.34
6pt27	20.4	20.9	0.5	42.3	3.8	0.92
6pt28	20.9	21.6	0.7	37.9	-4.4	0.69
6pt29	21.6	22.4	0.8	42.3	4.4	0.8
6pt30	22.4	22.9	0.5	38.4	-3.9	0.88
6pt31	22.9	23.7	0.8	36.8	-1.5	0.41
6pt32	23.7	24.1	0.4	37.3	0.4	0.84
6pt33	24.1	24.4	0.3	36.6	-0.7	0.36
6pt34	24.4	24.9	0.5	39.2	2.6	1.21
6pt35	24.9	25.4	0.5	42.0	2.8	0.97
6pt36	25.4	26.5	1.1	39.7	-2.4	1.13
6pt37	26.5	27.3	0.8	42.3	2.6	1.47
6pt38	27.3	27.6	0.3	37.4	-4.9	1.83
6pt39	27.6	27.8	0.2	42.4	5.0	0
6pt40	27.8	28	0.2	39.9	-2.5	0
6pt41	28	28.4	0.4	42.2	2.3	0.86
6pt42	28.4	28.7	0.3	39.2	-3.0	0.77
6pt43	28.7	28.9	0.2	41.3	2.1	0
6pt44	28.9	29.1	0.2	40.1	-1.2	0
6pt45	29.1	29.5	0.4	42.3	2.2	1.22
6pt46	29.5	30	0.5	37.6	-4.7	1.17
6pt47	30	31.1	1.1	44.3	6.7	1.18
6pt48	31.1	32.8	1.7	48.8	4.5	2.92

6pt49	32.8	33.5	0.7	40.3	-8.4	0.95
6pt50	33.5	33.8	0.3	42.2	1.8	0.8
6pt51	33.8	34.7	0.9	50.4	8.2	1.99
6pt52	34.7	34.9	0.2	53.8	3.4	0
6pt53	34.9	35.3	0.4	47.8	-6.0	4.22
6pt54	35.3	35.8	0.5	43.3	-4.5	1
6pt55	35.8	36.3	0.5	48.5	5.2	2
6pt56	36.3	37	0.7	43.2	-5.3	2.42
6pt57	37	37.2	0.2	46.9	3.8	0
6pt58	37.2	37.4	0.2	42.8	-4.1	0
6pt59	37.4	38	0.6	48.4	5.5	1.93
6pt60	38	38.3	0.3	43.4	-5.0	0.52
6pt61	38.3	38.7	0.4	49.3	6.0	2.7
6pt62	38.7	39.9	1.2	40.0	-9.4	2.49
6pt63	39.9	40.4	0.5	46.3	6.3	3.01
6pt64	40.4	40.6	0.2	38.2	-8.1	0
6pt65	40.6	41.4	0.8	44.2	6.0	2.58
6pt66	41.4	41.7	0.3	47.6	3.3	1.21
6pt67	41.7	42.3	0.6	44.1	-3.5	1.37
6pt68	42.3	43.3	1	48.4	4.3	2.95
6pt69	43.3	43.9	0.6	43.4	-5.0	2.49
6pt70	43.9	45.4	1.5	49.5	6.1	1.73
6pt71	45.4	45.9	0.5	44.0	-5.5	2.03
6pt72	45.9	46.4	0.5	36.3	-7.7	1.31
6pt73	46.4	46.6	0.2	38.5	2.2	0
6pt74	46.6	47.1	0.5	42.5	4.0	1.49
6pt75	47.1	49	1.9	39.2	-3.2	1.44
6pt76	49	51.8	2.8	35.9	-3.3	0.92
6pt77	51.8	53.4	1.6	38.1	2.1	1.41
6pt78	53.4	54.6	1.2	42.3	4.2	1.56
6pt79	54.6	55.2	0.6	39.8	-2.5	0.51
6pt80	55.2	57.5	2.3	35.7	-4.1	0.88
6pt81	57.5	58.9	1.4	38.1	2.4	1.11
6pt82	58.9	59.5	0.6	0.0	-38.1	0
6pt83	59.5	60.3	0.8	37.9	37.9	1.43
6pt84	60.3	63.2	2.9	0.0	-37.9	0
6pt85	63.2	63.4	0.2	36.1	36.1	0
6pt86	63.4	64.3	0.9	41.1	5.0	0
6pt87	64.3	70.1	5.8	34.8	-6.3	1.07
6pt88	70.1	70.4	0.3	37.5	2.7	0.37
6pt89	70.4	70.9	0.5	35.9	-1.6	0.95
6pt90	70.9	72.6	1.7	37.9	2.0	1.28
6pt91	72.6	73.7	1.1	36.3	-1.7	1.38
6pt92	73.7	74	0.3	38.4	2.2	1.21
6pt93	74	74.5	0.5	43.5	5.1	1.08
6pt94	74.5	74.9	0.4	39.4	-4.1	0.59
6pt95	74.9	76	1.1	36.4	-3.0	0.79
6pt96	76	77.1	1.1	38.2	1.8	0.81
6pt97	77.1	78.6	1.5	36.1	-2.1	0.72
6pt98	78.6	78.8	0.2	37.4	1.3	0
6pt99	78.8	79.6	0.8	36.1	-1.3	0.68
6pt100	79.6	79.9	0.3	37.7	1.5	0.28
6pt101	79.9	80.2	0.3	35.5	-2.2	1
6pt102	80.2	81.6	1.4	37.9	2.4	1.19
6pt103	81.6	82.8	1.2	35.6	-2.3	0.82
6pt104	82.8	88.9	6.1	38.3	2.8	1.19

6pt105	88.9	89.1	0.2	41.7	3.3	0
6pt106	89.1	90.2	1.1	38.6	-3.1	1.85
6pt107	90.2	91.3	1.1	42.4	3.8	0.94
6pt108	91.3	91.6	0.3	40.0	-2.4	0.28
6pt109	91.6	91.8	0.2	41.5	1.5	0
6pt110	91.8	92.6	0.8	37.6	-3.9	0.81
6pt111	92.6	98.2	5.6	34.9	-2.7	1.06
6pt112	98.2	98.9	0.7	38.0	3.0	1.26
6pt113	98.9	100.1	1.2	35.3	-2.6	0.98
6pt114	100.1	100.8	0.7	38.5	3.1	1.17
6pt115	100.8	101	0.2	41.4	2.9	0
6pt116	101	101.8	0.8	38.1	-3.2	1.47
6pt117	101.8	106.1	4.3	35.2	-3.0	1.52
6pt118	106.1	107	0.9	38.3	3.1	1.24
6pt119	107	107.2	0.2	41.2	2.9	0
6pt120	107.2	108.5	1.3	40.0	-1.2	1.09
6pt121	108.5	109	0.5	44.0	4.0	1.75
6pt122	109	109.4	0.4	39.7	-4.2	1.11
6pt123	109.4	110.2	0.8	42.5	2.8	1.62
6pt124	110.2	110.4	0.2	39.4	-3.1	0
6pt125	110.4	110.7	0.3	43.2	3.7	1.56
6pt126	110.7	111.1	0.4	39.1	-4.0	1.3
6pt127	111.1	111.4	0.3	42.9	3.8	0.84
6pt128	111.4	111.7	0.3	38.3	-4.6	1.01
6pt129	111.7	111.9	0.2	43.5	5.2	0
6pt130	111.9	112.2	0.3	38.5	-5.0	1.71
6pt131	112.2	112.5	0.3	42.4	3.9	0.47
6pt132	112.5	112.7	0.2	39.9	-2.6	0
6pt133	112.7	113.2	0.5	42.1	2.3	1.51
6pt134	113.2	113.4	0.2	36.9	-5.2	0
6pt135	113.4	114	0.6	42.4	5.5	1.36
6pt136	114	114.4	0.4	38.6	-3.8	1.76
6pt137	114.4	115.1	0.7	35.7	-2.9	0.71
6pt138	115.1	116.5	1.4	38.0	2.3	1.25
6pt139	116.5	117.8	1.3	35.8	-2.1	0.87
6pt140	117.8	118.7	0.9	37.6	1.8	0.98
6pt141	118.7	119	0.3	36.2	-1.5	0.79
6pt142	119	121.8	2.8	38.5	2.3	1.24
6pt143	121.8	123.4	1.6	35.3	-3.2	0.8
6pt144	123.4	123.7	0.3	38.6	3.3	0.57
6pt145	123.7	124.6	0.9	36.4	-2.3	0.66
6pt146	124.6	125	0.4	37.8	1.5	0.6
6pt147	125	126.9	1.9	35.9	-1.9	0.87
6pt148	126.9	128.4	1.5	38.5	2.5	0.93
6pt149	128.4	129.5	1.1	36.3	-2.2	0.61
6pt150	129.5	129.8	0.3	37.9	1.6	0.83
6pt151	129.8	131.7	1.9	35.8	-2.0	0.74
6pt152	131.7	135.6	3.9	38.2	2.4	0.97
6pt153	135.6	135.8	0.2	36.6	-1.6	0
6pt154	135.8	136.3	0.5	38.3	1.7	0.72
6pt155	136.3	136.6	0.3	42.0	3.7	0.49
6pt156	136.6	137.1	0.5	40.0	-2.0	1.16
6pt157	137.1	137.3	0.2	42.3	2.3	0
6pt158	137.3	140.6	3.3	39.2	-3.0	1.56
6pt159	140.6	141.3	0.7	41.3	2.1	0.42
6pt160	141.3	142.5	1.2	39.3	-2.0	1.81

6pt161	142.5	144.7	2.2	35.3	-4.0	1.03
6pt162	144.7	149.2	4.5	38.4	3.1	1.49
6pt163	149.2	149.4	0.2	35.6	-2.8	0
6pt164	149.4	149.8	0.4	38.1	2.5	0.3
6pt165	149.8	150	0.2	34.7	-3.4	0
6pt166	150	150.8	0.8	39.6	4.9	1.87
6pt167	150.8	151.2	0.4	42.9	3.3	0.62
6pt168	151.2	151.5	0.3	40.6	-2.3	0.49
6pt169	151.5	151.7	0.2	41.9	1.3	0
6pt170	151.7	152.1	0.4	40.1	-1.8	1.09
6pt171	152.1	152.6	0.5	43.2	3.1	1.72
6pt172	152.6	152.8	0.2	48.2	5.0	0
6pt173	152.8	154.3	1.5	43.1	-5.1	1.13
6pt174	154.3	156	1.7	38.6	-4.5	1.39
6pt175	156	156.6	0.6	35.8	-2.8	0.78
6pt176	156.6	157.2	0.6	39.0	3.2	1.34
6pt177	157.2	158.1	0.9	42.2	3.2	2.33
6pt178	158.1	159.4	1.3	38.7	-3.5	1.46
6pt179	159.4	159.7	0.3	42.2	3.4	0.39
6pt180	159.7	160	0.3	40.1	-2.1	0.76
6pt181	160	163.7	3.7	43.8	3.7	2.44
6pt182	163.7	164.1	0.4	39.2	-4.6	1.5
6pt183	164.1	164.9	0.8	42.5	3.3	1.6
6pt184	164.9	166.1	1.2	38.8	-3.7	1.32
6pt185	166.1	166.4	0.3	43.1	4.3	2.33
6pt186	166.4	166.6	0.2	37.8	-5.2	0
6pt187	166.6	167.3	0.7	42.2	4.3	1.65
6pt188	167.3	167.6	0.3	37.8	-4.4	1
6pt189	167.6	167.8	0.2	35.8	-2.0	0
6pt190	167.8	168.7	0.9	38.6	2.8	1.65
6pt191	168.7	169.5	0.8	43.1	4.5	1.51
6pt192	169.5	169.9	0.4	47.5	4.4	0.75
6pt193	169.9	170.3	0.4	43.7	-3.7	1.36
6pt194	170.3	172	1.7	46.9	3.1	3.31
6pt195	172	172.9	0.9	42.9	-3.9	1.92
6pt196	172.9	174	1.1	48.7	5.7	4.23
7pt1	0	0.7	0.7	52.2		4.67
7pt2	0.7	2.1	1.4	56.0	3.8	2.94
7pt3	2.1	2.4	0.3	51.4	-4.6	1.89
7pt4	2.4	2.6	0.2	57.3	6.0	0
7pt5	2.6	3	0.4	49.1	-8.2	3.08
7pt6	3	3.3	0.3	44.7	-4.3	2.1
7pt7	3.3	3.8	0.5	39.5	-5.3	1.07
7pt8	3.8	4.3	0.5	47.3	7.9	3.55
7pt9	4.3	4.6	0.3	44.3	-3.0	0.9
7pt10	4.6	5.2	0.6	49.1	4.8	3.37
7pt11	5.2	5.6	0.4	54.4	5.3	1.1
7pt12	5.6	7	1.4	47.3	-7.1	2.52
7pt13	7	8.8	1.8	38.3	-9.0	1.45
7pt14	8.8	10.6	1.8	35.0	-3.3	0.9
7pt15	10.6	11	0.4	37.5	2.6	0.98
7pt16	11	12.6	1.6	35.8	-1.7	1.52
7pt17	12.6	13	0.4	37.9	2.0	0.65
7pt18	13	16.5	3.5	35.6	-2.3	0.85
7pt19	16.5	17.2	0.7	38.3	2.8	0.72

7pt20	17.2	17.5	0.3	36.0	-2.3	1.06
7pt21	17.5	17.9	0.4	38.0	2.0	0.84
7pt22	17.9	20.6	2.7	36.3	-1.7	0.88
7pt23	20.6	22.6	2	39.0	2.7	1.44
7pt24	22.6	23.2	0.6	41.6	2.7	1.29
7pt25	23.2	23.7	0.5	40.1	-1.5	2.44
7pt26	23.7	23.9	0.2	42.2	2.0	0
7pt27	23.9	25	1.1	39.3	-2.9	1.65
7pt28	25	25.6	0.6	41.5	2.2	0.82
7pt29	25.6	25.9	0.3	40.6	-0.9	0.34
7pt30	25.9	27	1.1	42.2	1.6	1.12
7pt31	27	27.2	0.2	36.1	-6.1	0
7pt32	27.2	27.4	0.2	39.1	3.0	0
7pt33	27.4	27.6	0.2	49.2	10.2	0
7pt34	27.6	27.9	0.3	41.5	-7.7	0.13
7pt35	27.9	28.2	0.3	38.0	-3.5	2.25
7pt36	28.2	28.6	0.4	41.2	3.1	0.75
7pt37	28.6	28.8	0.2	39.9	-1.3	0
7pt38	28.8	29	0.2	41.1	1.2	0
7pt39	29	29.6	0.6	40.8	-0.4	0.73
7pt40	29.6	29.9	0.3	42.1	1.4	0.49
7pt41	29.9	30.1	0.2	39.5	-2.6	0
7pt42	30.1	31.2	1.1	42.8	3.3	3.75
7pt43	31.2	31.5	0.3	50.0	7.1	2.52
7pt44	31.5	32.5	1	39.3	-10.7	1.14
7pt45	32.5	32.9	0.4	41.8	2.5	0.69
7pt46	32.9	33.3	0.4	39.9	-1.9	1.83
7pt47	33.3	33.5	0.2	42.7	2.8	0
7pt48	33.5	34.1	0.6	37.8	-4.9	1.48
7pt49	34.1	34.6	0.5	41.5	3.7	1.3
7pt50	34.6	35.8	1.2	38.9	-2.6	1.13
7pt51	35.8	36	0.2	42.8	3.9	0
7pt52	36	36.4	0.4	39.3	-3.5	1.19
7pt53	36.4	37.5	1.1	42.3	3.0	2.14
7pt54	37.5	39.8	2.3	39.2	-3.1	1.51
7pt55	39.8	41.1	1.3	42.5	3.2	1.98
7pt56	41.1	42	0.9	39.5	-3.0	0.5
7pt57	42	42.3	0.3	42.0	2.5	0.74
7pt58	42.3	44.3	2	39.2	-2.8	1.23
7pt59	44.3	44.8	0.5	41.9	2.7	1.79
7pt60	44.8	46	1.2	48.6	6.6	3.68
7pt61	46	47	1	42.5	-6.1	2.38
7pt62	47	47.8	0.8	38.4	-4.1	0.56
7pt63	47.8	48.1	0.3	42.6	4.2	2.04
7pt64	48.1	48.5	0.4	48.4	5.8	1.13
7pt65	48.5	48.9	0.4	43.2	-5.2	1.44
7pt66	48.9	49.9	1	38.7	-4.4	1.14
7pt67	49.9	50.5	0.6	36.5	-2.3	0.94
7pt68	50.5	50.8	0.3	39.6	3.2	1.13
7pt69	50.8	51	0.2	35.9	-3.8	0
7pt70	51	51.2	0.2	39.7	3.9	0
7pt71	51.2	52.6	1.4	43.4	3.7	1.46
7pt72	52.6	53	0.4	39.2	-4.1	0.88
7pt73	53	53.2	0.2	36.4	-2.8	0
7pt74	53.2	53.4	0.2	39.3	2.8	0
7pt75	53.4	53.9	0.5	36.2	-3.0	0.59

7pt76	53.9	54.1	0.2	37.8	1.6	0
7pt77	54.1	55.1	1	36.1	-1.6	0.75
7pt78	55.1	56	0.9	38.3	2.2	1.69
7pt79	56	57.4	1.4	44.5	6.2	1.38
7pt80	57.4	58	0.6	39.0	-5.5	2.95
7pt81	58	58.3	0.3	45.0	6.0	3.23
7pt82	58.3	58.6	0.3	40.0	-5.0	0.25
7pt83	58.6	58.8	0.2	43.9	3.9	0
7pt84	58.8	59.5	0.7	37.3	-6.6	0.9
7pt85	59.5	62.4	2.9	0.0	-37.3	0
7pt86	62.4	62.7	0.3	45.0	45.0	0.74
7pt87	62.7	62.9	0.2	38.1	-6.9	0
7pt88	62.9	64	1.1	43.2	5.1	2.58
7pt89	64	64.2	0.2	38.5	-4.7	0
7pt90	64.2	65	0.8	42.2	3.7	1.55
7pt91	65	65.3	0.3	38.6	-3.6	0.71
7pt92	65.3	66.1	0.8	44.1	5.6	1.72
7pt93	66.1	66.4	0.3	47.5	3.4	1.15
7pt94	66.4	69.6	3.2	43.5	-4.0	1.63
7pt95	69.6	70.1	0.5	38.7	-4.8	0.84
7pt96	70.1	72.8	2.7	43.7	5.0	1.1
7pt97	72.8	75.9	3.1	49.6	5.9	2.58
7pt98	75.9	76.1	0.2	43.0	-6.6	0
7pt99	76.1	77.7	1.6	40.0	-3.0	3.09
7pt100	77.7	80.3	2.6	36.0	-4.0	0.91
7pt101	80.3	80.5	0.2	38.3	2.3	0
7pt102	80.5	86.3	5.8	35.0	-3.3	1.12
7pt103	86.3	87.2	0.9	38.1	3.1	1.31
7pt104	87.2	87.5	0.3	35.9	-2.2	0.81
7pt105	87.5	88.2	0.7	37.4	1.6	0.82
7pt106	88.2	89.7	1.5	35.5	-2.0	0.81
7pt107	89.7	92.8	3.1	38.4	2.9	1.14
7pt108	92.8	93.9	1.1	36.1	-2.3	1.06
7pt109	93.9	94.1	0.2	38.5	2.4	0
7pt110	94.1	94.9	0.8	36.7	-1.8	1
7pt111	94.9	96.6	1.7	38.4	1.7	0.86
7pt112	96.6	96.8	0.2	41.3	2.9	0
7pt113	96.8	97.6	0.8	39.0	-2.3	0.85
7pt114	97.6	99.3	1.7	47.2	8.2	2.52
7pt115	99.3	99.8	0.5	42.2	-5.1	1.54
7pt116	99.8	102.5	2.7	50.2	8.0	2.08
7pt117	102.5	104.7	2.2	38.8	-11.3	1.29
7pt118	104.7	104.9	0.2	43.0	4.2	0
7pt119	104.9	105.2	0.3	39.2	-3.9	0.77
7pt120	105.2	106.1	0.9	44.0	4.8	1.39
7pt121	106.1	107.2	1.1	40.0	-4.0	1.07
7pt122	107.2	107.5	0.3	36.4	-3.5	0.42
7pt123	107.5	107.9	0.4	39.5	3.1	0.63
7pt124	107.9	108.1	0.2	41.9	2.4	0
7pt125	108.1	108.9	0.8	39.0	-3.0	0.69
7pt126	108.9	110.6	1.7	35.5	-3.5	0.81
7pt127	110.6	110.8	0.2	37.8	2.3	0
7pt128	110.8	111.6	0.8	35.8	-2.0	0.59
7pt129	111.6	112.8	1.2	39.1	3.3	0.94
7pt130	112.8	114.8	2	35.9	-3.2	0.97
7pt131	114.8	115	0.2	37.3	1.3	0

7pt132	115	116.4	1.4	35.5	-1.7	0.87
7pt133	116.4	117.6	1.2	38.9	3.3	1.26
7pt134	117.6	117.8	0.2	36.1	-2.8	0
7pt135	117.8	118.4	0.6	38.2	2.1	0.71
7pt136	118.4	121.6	3.2	35.2	-3.0	1.14
7pt137	121.6	121.9	0.3	37.8	2.7	0.29
7pt138	121.9	122.5	0.6	36.3	-1.5	0.59
7pt139	122.5	122.7	0.2	38.4	2.1	0
7pt140	122.7	123	0.3	35.7	-2.7	0.83
7pt141	123	123.4	0.4	37.4	1.7	0.29
7pt142	123.4	124.1	0.7	36.7	-0.7	0.58
7pt143	124.1	124.4	0.3	38.2	1.5	0.21
7pt144	124.4	127.2	2.8	35.6	-2.6	0.97
7pt145	127.2	128.4	1.2	39.5	3.9	2.08
7pt146	128.4	128.9	0.5	47.6	8.1	2.1
7pt147	128.9	129.2	0.3	44.1	-3.5	1.31
7pt148	129.2	129.4	0.2	51.3	7.2	0
7pt149	129.4	130.9	1.5	43.4	-7.9	2.18
7pt150	130.9	131.3	0.4	39.9	-3.5	0.47
7pt151	131.3	131.7	0.4	42.2	2.3	0.39
7pt152	131.7	131.9	0.2	37.8	-4.4	0
7pt153	131.9	132.6	0.7	45.1	7.3	2.11
7pt154	132.6	133	0.4	47.3	2.2	1.5
7pt155	133	133.3	0.3	44.1	-3.2	1.01
7pt156	133.3	134.8	1.5	39.0	-5.1	1.53
7pt157	134.8	135.1	0.3	43.3	4.3	0.1
7pt158	135.1	135.7	0.6	39.2	-4.1	1.8
7pt159	135.7	135.9	0.2	45.5	6.4	0
7pt160	135.9	136.2	0.3	38.8	-6.7	2.27
7pt161	136.2	136.5	0.3	43.7	4.9	1.86
7pt162	136.5	137.1	0.6	37.9	-5.9	1.23
7pt163	137.1	137.8	0.7	36.3	-1.5	0.45
7pt164	137.8	138.4	0.6	38.7	2.4	1.12
7pt165	138.4	141.1	2.7	43.2	4.5	2.32
7pt166	141.1	141.4	0.3	46.6	3.3	1.38
7pt167	141.4	141.7	0.3	39.0	-7.5	1.89
7pt168	141.7	142.4	0.7	42.4	3.4	1.65
7pt169	142.4	142.9	0.5	39.4	-3.1	0.36
7pt170	142.9	144.2	1.3	43.0	3.6	2.78
7pt171	144.2	144.8	0.6	38.6	-4.3	1.65
7pt172	144.8	145	0.2	42.1	3.5	0
7pt173	145	145.7	0.7	37.5	-4.7	0.69
7pt174	145.7	148	2.3	36.0	-1.5	0.51
7pt175	148	148.7	0.7	38.3	2.3	0.89
7pt176	148.7	149.6	0.9	42.3	4.1	1.31
7pt177	149.6	150	0.4	47.3	5.0	0.92
7pt178	150	150.3	0.3	43.9	-3.4	0.75
7pt179	150.3	150.6	0.3	50.0	6.1	5.53
7pt180	150.6	150.8	0.2	41.5	-8.5	0
7pt181	150.8	151	0.2	48.1	6.6	0
7pt182	151	151.4	0.4	42.0	-6.1	2.53
7pt183	151.4	151.8	0.4	53.9	11.9	3.38
7pt184	151.8	152	0.2	49.1	-4.8	0
7pt185	152	152.2	0.2	44.0	-5.1	0
7pt186	152.2	152.5	0.3	50.0	6.0	0.92
7pt187	152.5	152.7	0.2	42.4	-7.6	0

7pt188	152.7	152.9	0.2	37.4	-5.0	0
7pt189	152.9	153.7	0.8	44.6	7.2	2.4
7pt190	153.7	153.9	0.2	40.3	-4.3	0
7pt191	153.9	154.2	0.3	41.6	1.3	0.91
7pt192	154.2	154.5	0.3	39.7	-1.9	0.41
7pt193	154.5	155.1	0.6	43.4	3.7	1.31
7pt194	155.1	155.3	0.2	38.8	-4.6	0
7pt195	155.3	155.9	0.6	45.1	6.4	2.2
7pt196	155.9	156.1	0.2	48.8	3.7	0
7pt197	156.1	156.4	0.3	52.2	3.4	2.61
7pt198	156.4	156.6	0.2	42.0	-10.1	0
7pt199	156.6	157	0.4	49.2	7.2	1.88
7pt200	157	157.5	0.5	44.0	-5.2	1.35
7pt201	157.5	157.7	0.2	38.5	-5.5	0
7pt202	157.7	158.6	0.9	48.5	10.0	3.61
7pt203	158.6	158.8	0.2	53.9	5.4	0
7pt204	158.8	159.5	0.7	50.0	-3.9	1.57
7pt205	159.5	159.8	0.3	43.6	-6.5	1.65
7pt206	159.8	160.1	0.3	48.0	4.4	0.87
7pt207	160.1	160.3	0.2	41.3	-6.7	0
8pt1	0	0.5	0.5	44.2		1.72
8pt2	0.5	2.1	1.6	47.9	3.7	1.32
8pt3	2.1	2.4	0.3	43.6	-4.3	1.77
8pt4	2.4	5.2	2.8	38.5	-5.1	1.2
8pt5	5.2	5.4	0.2	36.5	-2.0	0
8pt6	5.4	6.2	0.8	38.4	1.9	1.45
8pt7	6.2	8.3	2.1	44.2	5.8	3.21
8pt8	8.3	8.5	0.2	46.3	2.1	0
8pt9	8.5	9.1	0.6	42.2	-4.1	1.51
8pt10	9.1	9.5	0.4	38.5	-3.8	0.78
8pt11	9.5	11.5	2	35.8	-2.7	0.94
8pt12	11.5	11.7	0.2	38.0	2.3	0
8pt13	11.7	12	0.3	36.3	-1.7	0.42
8pt14	12	12.2	0.2	38.0	1.7	0
8pt15	12.2	12.9	0.7	35.7	-2.2	0.76
8pt16	12.9	13.7	0.8	38.2	2.5	1.51
8pt17	13.7	14	0.3	41.6	3.4	0.4
8pt18	14	15.5	1.5	39.6	-2.0	1.05
8pt19	15.5	16	0.5	41.9	2.4	0.88
8pt20	16	16.3	0.3	40.2	-1.7	0.74
8pt21	16.3	18	1.7	43.7	3.5	1.62
8pt22	18	19.4	1.4	48.5	4.8	4.39
8pt23	19.4	20.2	0.8	45.3	-3.3	2.39
8pt24	20.2	20.7	0.5	38.6	-6.6	1.47
8pt25	20.7	20.9	0.2	36.6	-2.0	0
8pt26	20.9	21.6	0.7	39.1	2.5	1.91
8pt27	21.6	21.8	0.2	42.3	3.2	0
8pt28	21.8	22.4	0.6	40.6	-1.6	0.8
8pt29	22.4	23.3	0.9	42.8	2.2	1.87
8pt30	23.3	23.7	0.4	39.0	-3.9	0.53
8pt31	23.7	24.1	0.4	47.3	8.3	1.37
8pt32	24.1	25.4	1.3	43.4	-3.9	2.98
8pt33	25.4	25.8	0.4	40.1	-3.3	2.51
8pt34	25.8	27.4	1.6	42.9	2.8	1.32
8pt35	27.4	30	2.6	38.2	-4.7	1.29

8pt36	30	30.5	0.5	43.5	5.3	2
8pt37	30.5	31.3	0.8	38.0	-5.6	1.16
8pt38	31.3	31.5	0.2	36.3	-1.6	0
8pt39	31.5	33.9	2.4	38.5	2.1	0.98
8pt40	33.9	34.3	0.4	43.4	4.9	1.43
8pt41	34.3	34.9	0.6	47.5	4.2	1.06
8pt42	34.9	35.2	0.3	41.6	-6.0	2.28
8pt43	35.2	35.4	0.2	47.6	6.1	0
8pt44	35.4	35.8	0.4	44.3	-3.4	1.4
8pt45	35.8	36.2	0.4	37.7	-6.6	0.9
8pt46	36.2	36.5	0.3	34.2	-3.5	0.11
8pt47	36.5	37.5	1	39.7	5.5	1.47
8pt48	37.5	38.3	0.8	43.7	4.1	2.14
8pt49	38.3	39.1	0.8	45.8	2.1	3.71
8pt50	39.1	40	0.9	43.3	-2.5	1.47
8pt51	40	40.9	0.9	38.7	-4.6	2.49
8pt52	40.9	43.8	2.9	0.0	-38.7	0
8pt53	43.8	44.1	0.3	37.5	37.5	1.62
8pt54	44.1	44.4	0.3	47.2	9.7	2.9
8pt55	44.4	45	0.6	39.5	-7.7	1.79
8pt56	45	45.2	0.2	41.9	2.4	0
8pt57	45.2	45.6	0.4	40.2	-1.7	0.27
8pt58	45.6	46.9	1.3	43.0	2.8	1.22
8pt59	46.9	47.1	0.2	39.5	-3.5	0
8pt60	47.1	49.3	2.2	36.1	-3.4	0.75
8pt61	49.3	51.6	2.3	39.3	3.3	1.12
8pt62	51.6	52.6	1	41.8	2.4	1.29
8pt63	52.6	53.9	1.3	39.4	-2.4	1.1
8pt64	53.9	54.3	0.4	42.9	3.5	0.9
8pt65	54.3	55.3	1	39.1	-3.8	1.32
8pt66	55.3	55.5	0.2	44.1	5.0	0
8pt67	55.5	57.5	2	38.3	-5.8	1.04
8pt68	57.5	58.1	0.6	36.0	-2.3	0.69
8pt69	58.1	59	0.9	39.7	3.7	1.27
8pt70	59	59.3	0.3	42.9	3.2	1.67
8pt71	59.3	60.2	0.9	38.9	-4.0	1.1
8pt72	60.2	60.7	0.5	36.7	-2.2	0.41
8pt73	60.7	61.8	1.1	38.4	1.8	1.04
8pt74	61.8	62.5	0.7	35.9	-2.5	1.08
8pt75	62.5	66.3	3.8	39.4	3.5	1.71
8pt76	66.3	66.5	0.2	36.5	-2.8	0
8pt77	66.5	67.2	0.7	37.0	0.5	0.85
8pt78	67.2	67.4	0.2	36.2	-0.8	0
8pt79	67.4	68.1	0.7	39.1	2.9	1.65
8pt80	68.1	68.5	0.4	42.9	3.8	1.22
8pt81	68.5	71.4	2.9	38.4	-4.5	1.05
8pt82	71.4	71.9	0.5	42.1	3.7	1.16
8pt83	71.9	72.3	0.4	38.1	-4.0	1.37
8pt84	72.3	72.7	0.4	41.5	3.4	0.39
8pt85	72.7	73.3	0.6	37.9	-3.7	1.31
8pt86	73.3	77.9	4.6	35.4	-2.5	0.95
8pt87	77.9	78.3	0.4	37.7	2.3	1.03
8pt88	78.3	78.5	0.2	42.3	4.7	0
8pt89	78.5	78.7	0.2	38.5	-3.8	0
8pt90	78.7	79.3	0.6	41.9	3.4	1.53
8pt91	79.3	80.5	1.2	38.8	-3.1	1.45

8pt92	80.5	83.6	3.1	35.1	-3.7	0.84
8pt93	83.6	85.8	2.2	38.7	3.6	3.45
8pt94	85.8	88.4	2.6	35.7	-3.1	0.96
8pt95	88.4	88.8	0.4	37.8	2.1	0.52
8pt96	88.8	89	0.2	36.6	-1.2	0
8pt97	89	89.6	0.6	38.0	1.3	0.74
8pt98	89.6	90.5	0.9	36.5	-1.5	0.69
8pt99	90.5	91.3	0.8	38.0	1.5	0.57
8pt100	91.3	91.9	0.6	35.9	-2.1	1.14
8pt101	91.9	92.6	0.7	39.4	3.5	1.09
8pt102	92.6	93.1	0.5	42.1	2.7	1.13
8pt103	93.1	93.7	0.6	40.0	-2.1	1.74
8pt104	93.7	94.3	0.6	42.0	2.1	0.98
8pt105	94.3	95	0.7	38.8	-3.2	0.8
8pt106	95	95.5	0.5	42.0	3.1	0.98
8pt107	95.5	96.3	0.8	39.6	-2.4	1.82
8pt108	96.3	97.4	1.1	42.8	3.2	1.41
8pt109	97.4	97.6	0.2	36.7	-6.1	0
8pt110	97.6	98.1	0.5	39.1	2.4	2.46
8pt111	98.1	98.6	0.5	35.5	-3.6	0.73
8pt112	98.6	99.3	0.7	38.9	3.4	1.82
8pt113	99.3	100.7	1.4	42.9	3.9	1.56
8pt114	100.7	101.4	0.7	39.7	-3.2	1.34
8pt115	101.4	102.3	0.9	43.7	4.0	2.11
8pt116	102.3	102.7	0.4	38.1	-5.6	0.72
8pt117	102.7	103.3	0.6	35.1	-2.9	1.05
8pt118	103.3	104.1	0.8	39.1	3.9	2.08
8pt119	104.1	105.6	1.5	36.5	-2.5	0.87
8pt120	105.6	106.1	0.5	37.5	0.9	1.84
8pt121	106.1	106.6	0.5	36.6	-0.9	0.44
8pt122	106.6	108.4	1.8	37.5	1.0	0.96
8pt123	108.4	108.7	0.3	35.8	-1.7	1.01
8pt124	108.7	108.9	0.2	38.3	2.5	0
8pt125	108.9	115.5	6.6	34.8	-3.5	1.13
8pt126	115.5	117.3	1.8	39.0	4.2	1.17
8pt127	117.3	117.6	0.3	41.4	2.4	0.36
8pt128	117.6	119.3	1.7	38.9	-2.5	1.05
8pt129	119.3	119.6	0.3	42.3	3.5	1.08
8pt130	119.6	122.1	2.5	38.0	-4.3	1.42
8pt131	122.1	123.3	1.2	43.6	5.6	2.5
8pt132	123.3	123.5	0.2	39.9	-3.6	0
8pt133	123.5	123.8	0.3	42.2	2.3	0.47
8pt134	123.8	124.1	0.3	40.5	-1.7	0.75
8pt135	124.1	124.6	0.5	43.7	3.2	1.88
8pt136	124.6	124.9	0.3	39.5	-4.2	1.19
8pt137	124.9	125.3	0.4	45.8	6.3	1.49
8pt138	125.3	126	0.7	38.7	-7.0	1.32
8pt139	126	126.2	0.2	42.7	3.9	0
8pt140	126.2	127	0.8	39.2	-3.5	1.04
8pt141	127	127.4	0.4	42.7	3.6	1.46
8pt142	127.4	127.6	0.2	47.4	4.7	0
8pt143	127.6	127.8	0.2	44.3	-3.2	0
8pt144	127.8	129.5	1.7	38.8	-5.4	1.16
8pt145	129.5	130.5	1	42.6	3.8	2.25
8pt146	130.5	131.7	1.2	38.6	-4.0	1.2
8pt147	131.7	132.2	0.5	43.3	4.8	0.76

8pt148	132.2	132.5	0.3	38.9	-4.5	2.26
8pt149	132.5	132.7	0.2	44.0	5.1	0
8pt150	132.7	133.3	0.6	47.1	3.2	1.36
8pt151	133.3	133.6	0.3	43.9	-3.2	1.03
8pt152	133.6	134	0.4	39.7	-4.2	0.36
8pt153	134	135.2	1.2	42.9	3.2	1.45
8pt154	135.2	135.4	0.2	38.0	-4.9	0
8pt155	135.4	135.6	0.2	44.3	6.3	0
8pt156	135.6	136.4	0.8	38.2	-6.1	1.28
8pt157	136.4	136.9	0.5	36.8	-1.5	0.58
8pt158	136.9	137.9	1	38.5	1.8	1.39
8pt159	137.9	138.4	0.5	42.7	4.1	1.34
8pt160	138.4	138.7	0.3	46.8	4.1	0.35
8pt161	138.7	139.4	0.7	42.7	-4.0	1.28
8pt162	139.4	140.1	0.7	48.6	5.8	3.12
8pt163	140.1	140.3	0.2	45.1	-3.4	0
8pt164	140.3	140.6	0.3	46.0	0.8	5
8pt165	140.6	140.8	0.2	38.8	-7.1	0
8pt166	140.8	141.1	0.3	50.8	12.0	1.45
8pt167	141.1	141.3	0.2	56.9	6.1	0
8pt168	141.3	142	0.7	53.3	-3.6	1.74
8pt169	142	142.5	0.5	57.5	4.2	2.57
8pt170	142.5	143.1	0.6	50.3	-7.3	3.54
8pt171	143.1	144.7	1.6	56.3	6.0	3.13
8pt172	144.7	145.1	0.4	49.1	-7.2	3.48
9pt1	0	0.3	0.3	39.4		0
9pt2	0.3	1.1	0.8	42.9	3.5	1.22
9pt3	1.1	3.3	2.2	39.5	-3.4	1.04
9pt4	3.3	3.6	0.3	35.3	-4.2	0.52
9pt5	3.6	4.5	0.9	39.8	4.5	0.79
9pt6	4.5	5	0.5	41.4	1.6	0.95
9pt7	5	6.6	1.6	39.1	-2.4	1.46
9pt8	6.6	7	0.4	43.5	4.4	1.81
9pt9	7	8.5	1.5	39.0	-4.5	1.29
9pt10	8.5	8.8	0.3	36.7	-2.3	0.27
9pt11	8.8	9.1	0.3	37.9	1.2	0.41
9pt12	9.1	13.2	4.1	35.1	-2.8	0.72
9pt13	13.2	13.4	0.2	37.6	2.5	0
9pt14	13.4	13.6	0.2	35.9	-1.7	0
9pt15	13.6	15.6	2	38.5	2.5	1.31
9pt16	15.6	15.8	0.2	41.6	3.1	0
9pt17	15.8	16.1	0.3	39.1	-2.5	1.2
9pt18	16.1	16.4	0.3	36.0	-3.1	0.84
9pt19	16.4	16.8	0.4	43.5	7.5	1
9pt20	16.8	17.7	0.9	39.1	-4.4	1.57
9pt21	17.7	17.9	0.2	35.2	-3.8	0
9pt22	17.9	19.3	1.4	38.7	3.4	1.08
9pt23	19.3	19.7	0.4	42.9	4.2	0.84
9pt24	19.7	22.8	3.1	38.5	-4.4	1.59
9pt25	22.8	24	1.2	35.9	-2.6	0.85
9pt26	24	24.4	0.4	38.8	3.0	0.65
9pt27	24.4	27	2.6	35.7	-3.1	0.97
9pt28	27	27.9	0.9	38.7	3.0	0.98
9pt29	27.9	28.1	0.2	41.8	3.1	0
9pt30	28.1	28.5	0.4	38.9	-2.8	0.62

9pt31	28.5	32.5	4	35.3	-3.6	0.86
9pt32	32.5	33.3	0.8	38.7	3.4	1.66
9pt33	33.3	34.2	0.9	43.8	5.1	2.71
9pt34	34.2	34.4	0.2	40.4	-3.4	0
9pt35	34.4	35	0.6	43.5	3.1	1.84
9pt36	35	35.4	0.4	49.4	5.9	2.64
9pt37	35.4	36.3	0.9	43.0	-6.4	2.75
9pt38	36.3	36.6	0.3	48.5	5.5	1.86
9pt39	36.6	37.5	0.9	43.0	-5.4	1.67
9pt40	37.5	37.9	0.4	47.5	4.4	3.01
9pt41	37.9	38.1	0.2	36.2	-11.3	0
9pt42	38.1	38.7	0.6	43.8	7.6	2.31
9pt43	38.7	38.9	0.2	49.5	5.7	0
9pt44	38.9	39.5	0.6	44.1	-5.4	2.27
9pt45	39.5	40.1	0.6	39.5	-4.6	1.48
9pt46	40.1	40.3	0.2	0.0	-39.5	0
9pt47	40.3	40.5	0.2	39.4	39.4	0
9pt48	40.5	41.7	1.2	37.8	-1.6	0
9pt49	41.7	43	1.3	39.3	1.5	2.3
9pt50	43	43.2	0.2	0.0	-39.3	0
9pt51	43.2	43.4	0.2	54.6	54.6	0
9pt52	43.4	43.6	0.2	44.6	-10.1	0
9pt53	43.6	44	0.4	34.5	-10.0	1.47
9pt54	44	44.5	0.5	40.0	5.5	3.96
9pt55	44.5	45.1	0.6	36.9	-3.1	2.28
9pt56	45.1	45.3	0.2	50.1	13.1	0
9pt57	45.3	63.2	17.9	0.0	-50.1	0
9pt58	63.2	63.9	0.7	37.3	37.3	4.4
9pt59	63.9	64.1	0.2	35.0	-2.4	0
9pt60	64.1	64.3	0.2	0.0	-35.0	0
9pt61	64.3	64.6	0.3	37.8	37.8	4.99
9pt62	64.6	64.8	0.2	49.7	11.9	0
9pt63	64.8	65	0.2	35.7	-14.1	0
9pt64	65	65.2	0.2	0.0	-35.7	0
9pt65	65.2	65.4	0.2	46.8	46.8	0
9pt66	65.4	65.4	0	0.0	-46.8	0
9pt67	65.4	65.8	0.4	34.6	34.6	2.42
9pt68	65.8	67.7	1.9	40.2	5.6	2.06
9pt69	67.7	68.3	0.6	43.1	2.9	1.06
9pt70	68.3	69.1	0.8	39.0	-4.1	1.48
9pt71	69.1	69.4	0.3	42.2	3.2	0.54
9pt72	69.4	70	0.6	37.8	-4.3	0.68
9pt73	70	70.2	0.2	36.4	-1.4	0
9pt74	70.2	70.8	0.6	39.3	2.8	1.18
9pt75	70.8	71	0.2	41.3	2.0	0
9pt76	71	71.7	0.7	39.7	-1.6	1.62
9pt77	71.7	73.4	1.7	35.6	-4.1	0.8
9pt78	73.4	75.1	1.7	39.3	3.7	1.12
9pt79	75.1	75.9	0.8	41.9	2.6	0.82
9pt80	75.9	76.3	0.4	37.5	-4.3	2.59
9pt81	76.3	76.6	0.3	41.9	4.4	0.57
9pt82	76.6	77.2	0.6	39.2	-2.8	0.64
9pt83	77.2	77.4	0.2	42.6	3.5	0
9pt84	77.4	79.2	1.8	38.5	-4.2	1.04
9pt85	79.2	80	0.8	36.4	-2.1	0.71
9pt86	80	80.5	0.5	38.5	2.1	1.55

9pt87	80.5	80.9	0.4	42.7	4.2	0.64
9pt88	80.9	82.2	1.3	38.6	-4.1	0.73
9pt89	82.2	82.6	0.4	41.5	2.9	0.17
9pt90	82.6	83.1	0.5	39.5	-2.0	1.45
9pt91	83.1	83.5	0.4	42.2	2.7	0.8
9pt92	83.5	83.9	0.4	40.3	-1.9	0.5
9pt93	83.9	84.5	0.6	41.0	0.7	0.97
9pt94	84.5	85	0.5	38.8	-2.3	1.66
9pt95	85	85.2	0.2	43.2	4.4	0
9pt96	85.2	85.7	0.5	39.5	-3.6	0.81
9pt97	85.7	86	0.3	41.6	2.1	0.61
9pt98	86	86.4	0.4	40.1	-1.5	0.24
9pt99	86.4	88.5	2.1	43.2	3.1	1.65
9pt100	88.5	89	0.5	48.9	5.7	2.08
9pt101	89	89.3	0.3	44.2	-4.7	1
9pt102	89.3	89.9	0.6	39.7	-4.5	1.31
9pt103	89.9	90.4	0.5	43.9	4.2	1.21
9pt104	90.4	90.7	0.3	40.2	-3.7	1.34
9pt105	90.7	90.9	0.2	41.4	1.2	0
9pt106	90.9	91.1	0.2	46.6	5.2	0
9pt107	91.1	91.5	0.4	43.7	-2.9	2.31
9pt108	91.5	91.8	0.3	39.4	-4.3	0.94
9pt109	91.8	92.6	0.8	48.4	9.0	3.87
9pt110	92.6	95	2.4	43.9	-4.5	3.21
9pt111	95	95.2	0.2	38.1	-5.8	0
9pt112	95.2	95.4	0.2	47.0	8.8	0
9pt113	95.4	95.9	0.5	43.4	-3.5	1.58
9pt114	95.9	96.1	0.2	40.3	-3.1	0
9pt115	96.1	96.3	0.2	43.1	2.8	0
9pt116	96.3	96.9	0.6	42.5	-0.6	3.63
9pt117	96.9	97.3	0.4	43.8	1.3	1.14
9pt118	97.3	97.6	0.3	47.5	3.7	1
9pt119	97.6	98.7	1.1	44.5	-2.9	1.99
9pt120	98.7	101.2	2.5	38.8	-5.7	1.26
9pt121	101.2	103.5	2.3	35.4	-3.5	0.81
9pt122	103.5	103.7	0.2	38.0	2.7	0
9pt123	103.7	104.1	0.4	36.8	-1.2	0.46
9pt124	104.1	104.6	0.5	43.0	6.2	0.67
9pt125	104.6	106.4	1.8	39.0	-4.1	1.13
9pt126	106.4	107.5	1.1	43.0	4.0	1.97
9pt127	107.5	107.9	0.4	39.3	-3.7	0.92
9pt128	107.9	108.3	0.4	42.1	2.8	0.62
9pt129	108.3	108.5	0.2	40.0	-2.1	0
9pt130	108.5	109	0.5	44.0	4.0	1.56
9pt131	109	109.2	0.2	40.0	-4.0	0
9pt132	109.2	109.8	0.6	42.7	2.8	0.68
9pt133	109.8	110.9	1.1	37.8	-4.9	0.7
9pt134	110.9	111.1	0.2	42.9	5.1	0
9pt135	111.1	111.4	0.3	39.1	-3.9	1.73
9pt136	111.4	111.9	0.5	41.6	2.6	2.02
9pt137	111.9	112.2	0.3	39.6	-2.0	0.99
9pt138	112.2	112.8	0.6	42.8	3.2	1.47
9pt139	112.8	113.1	0.3	48.1	5.3	2.18
9pt140	113.1	113.4	0.3	43.0	-5.1	2.58
9pt141	113.4	114	0.6	49.5	6.5	2.35
9pt142	114	114.3	0.3	43.6	-5.9	1.38

9pt143	114.3	115.7	1.4	39.1	-4.5	1.58
9pt144	115.7	117	1.3	41.8	2.7	0.72
9pt145	117	117.7	0.7	38.3	-3.5	0.99
9pt146	117.7	118	0.3	36.8	-1.5	0.31
9pt147	118	119.4	1.4	38.9	2.1	1.34
9pt148	119.4	120.6	1.2	43.1	4.2	1.95
9pt149	120.6	120.8	0.2	38.6	-4.5	0
9pt150	120.8	121.5	0.7	47.5	9.0	4.86
9pt151	121.5	121.7	0.2	43.8	-3.8	0
9pt152	121.7	121.9	0.2	47.2	3.4	0
9pt153	121.9	122.1	0.2	43.5	-3.7	0
9pt154	122.1	122.9	0.8	39.6	-3.9	1.98
9pt155	122.9	123.3	0.4	47.1	7.6	4.84
9pt156	123.3	123.6	0.3	39.3	-7.8	1.21
9pt157	123.6	124.2	0.6	49.1	9.8	2.99
9pt158	124.2	125.5	1.3	43.4	-5.7	3.67
9pt159	125.5	125.7	0.2	36.4	-7.0	0
9pt160	125.7	125.9	0.2	48.3	11.9	0
9pt161	125.9	126.2	0.3	44.8	-3.5	1.28
9pt162	126.2	126.5	0.3	51.1	6.3	1.78
9pt163	126.5	127.2	0.7	45.4	-5.8	2.2
9pt164	127.2	127.5	0.3	49.9	4.5	3.1
9pt165	127.5	128.1	0.6	53.4	3.6	2.11
9pt166	128.1	128.9	0.8	50.5	-2.9	2.09
9pt167	128.9	129.1	0.2	53.7	3.2	0
9pt168	129.1	129.4	0.3	51.9	-1.8	1.18
9pt169	129.4	129.6	0.2	53.7	1.8	0
9pt170	129.6	129.8	0.2	49.9	-3.8	0
9pt171	129.8	130	0.2	44.2	-5.8	0
9pt172	130	130.2	0.2	51.7	7.6	0
9pt173	130.2	130.5	0.3	55.4	3.7	1.11
9pt174	130.5	131.9	1.4	49.4	-5.9	3.32
9pt175	131.9	132.1	0.2	43.4	-6.1	0
9pt176	132.1	132.3	0.2	50.8	7.5	0
9pt177	132.3	132.5	0.2	43.6	-7.2	0
9pt178	132.5	133.4	0.9	49.0	5.4	3.05
9pt179	133.4	134.6	1.2	55.9	6.9	2.27
9pt180	134.6	134.8	0.2	51.2	-4.7	0
9pt181	134.8	135	0.2	56.1	4.9	0
9pt182	135	135.2	0.2	50.6	-5.5	0
9pt183	135.2	135.5	0.3	53.8	3.2	0.66
9pt184	135.5	135.7	0.2	52.3	-1.5	0
9pt185	135.7	137.9	2.2	57.4	5.1	3.54
9pt186	137.9	138.6	0.7	48.7	-8.7	1.91
10pt1	0	0.4	0.4	41.9		4.45
10pt2	0.4	1.8	1.4	47.4	5.5	1.83
10pt3	1.8	2	0.2	43.4	-4.0	0
10pt4	2	3	1	39.8	-3.6	0.71
10pt5	3	3.4	0.4	46.4	6.6	1.7
10pt6	3.4	4.2	0.8	43.0	-3.4	1.19
10pt7	4.2	5.6	1.4	39.2	-3.8	1.58
10pt8	5.6	5.8	0.2	50.6	11.4	0
10pt9	5.8	7	1.2	44.0	-6.6	2.97
10pt10	7	7.2	0.2	40.6	-3.4	0
10pt11	7.2	7.9	0.7	43.4	2.7	1.01

10pt12	7.9	8.2	0.3	40.0	-3.3	0.97
10pt13	8.2	8.7	0.5	42.6	2.6	3.21
10pt14	8.7	8.9	0.2	38.5	-4.1	0
10pt15	8.9	10.5	1.6	36.4	-2.1	0.68
10pt16	10.5	11.4	0.9	39.9	3.5	1.36
10pt17	11.4	11.9	0.5	42.9	2.9	3.58
10pt18	11.9	12.2	0.3	47.7	4.8	1.3
10pt19	12.2	12.6	0.4	43.3	-4.3	2.27
10pt20	12.6	12.8	0.2	46.2	2.9	0
10pt21	12.8	15.4	2.6	43.8	-2.4	1.7
10pt22	15.4	15.6	0.2	46.8	3.0	0
10pt23	15.6	16.2	0.6	43.5	-3.2	1.46
10pt24	16.2	17	0.8	38.4	-5.1	1.55
10pt25	17	17.5	0.5	41.4	3.0	0.93
10pt26	17.5	18	0.5	39.3	-2.1	0.95
10pt27	18	18.2	0.2	41.3	2.0	0
10pt28	18.2	19.5	1.3	39.8	-1.5	1.1
10pt29	19.5	20.1	0.6	35.7	-4.0	1.1
10pt30	20.1	20.5	0.4	37.8	2.1	0.55
10pt31	20.5	20.7	0.2	36.0	-1.8	0
10pt32	20.7	21.7	1	38.6	2.6	1.03
10pt33	21.7	22.1	0.4	44.1	5.4	0.89
10pt34	22.1	23	0.9	38.9	-5.2	1.38
10pt35	23	23.2	0.2	42.4	3.6	0
10pt36	23.2	23.6	0.4	39.5	-2.9	1.06
10pt37	23.6	24.1	0.5	41.7	2.2	0.98
10pt38	24.1	24.8	0.7	39.1	-2.6	0.83
10pt39	24.8	25.6	0.8	41.6	2.4	1.88
10pt40	25.6	25.9	0.3	39.3	-2.2	1.62
10pt41	25.9	26.1	0.2	36.0	-3.3	0
10pt42	26.1	27.1	1	39.3	3.2	1.85
10pt43	27.1	27.6	0.5	40.9	1.6	1.05
10pt44	27.6	28.9	1.3	39.0	-1.9	1.23
10pt45	28.9	31.6	2.7	42.9	3.9	1.58
10pt46	31.6	32.1	0.5	39.4	-3.5	2.04
10pt47	32.1	32.3	0.2	36.1	-3.2	0
10pt48	32.3	32.5	0.2	44.0	7.8	0
10pt49	32.5	32.8	0.3	39.0	-5.0	1.93
10pt50	32.8	33	0.2	43.9	4.9	0
10pt51	33	33.7	0.7	37.9	-5.9	0.88
10pt52	33.7	34.9	1.2	41.6	3.7	0.97
10pt53	34.9	35.4	0.5	38.9	-2.8	1.26
10pt54	35.4	36.6	1.2	42.9	4.0	1.53
10pt55	36.6	36.9	0.3	40.5	-2.4	0.16
10pt56	36.9	37.1	0.2	36.5	-3.9	0
10pt57	37.1	39.8	2.7	39.8	3.2	2.51
10pt58	39.8	42.2	2.4	0.0	-39.8	0
10pt59	42.2	42.9	0.7	40.5	40.5	2.73
10pt60	42.9	43.6	0.7	48.4	7.8	3.55
10pt61	43.6	44.7	1.1	43.8	-4.6	1.89
10pt62	44.7	45	0.3	40.2	-3.6	1.19
10pt63	45	45.3	0.3	45.7	5.5	2.89
10pt64	45.3	45.7	0.4	39.4	-6.3	0.71
10pt65	45.7	46.7	1	41.8	2.4	1.74
10pt66	46.7	47	0.3	46.9	5.1	0.79
10pt67	47	47.8	0.8	44.0	-2.8	3.12

10pt68	47.8	48	0.2	0.0	-44.0	0
10pt69	48	48.2	0.2	35.9	35.9	0
10pt70	48.2	48.4	0.2	53.4	17.5	0
10pt71	48.4	48.8	0.4	0.0	-53.4	0
10pt72	48.8	49.9	1.1	39.5	39.5	2.81
10pt73	49.9	51.1	1.2	36.3	-3.2	0.83
10pt74	51.1	51.3	0.2	37.5	1.2	0
10pt75	51.3	51.5	0.2	36.0	-1.6	0
10pt76	51.5	51.8	0.3	38.5	2.5	0.97
10pt77	51.8	56.8	5	35.0	-3.4	0.85
10pt78	56.8	57.9	1.1	38.5	3.5	0.78
10pt79	57.9	58.1	0.2	35.9	-2.6	0
10pt80	58.1	60.4	2.3	38.6	2.7	1.37
10pt81	60.4	60.6	0.2	36.0	-2.6	0
10pt82	60.6	61.5	0.9	38.9	2.9	1.5
10pt83	61.5	61.8	0.3	42.1	3.2	0.88
10pt84	61.8	63.2	1.4	39.5	-2.6	1.43
10pt85	63.2	66.2	3	35.6	-3.9	0.8
10pt86	66.2	67.2	1	38.6	3.0	1.91
10pt87	67.2	68.3	1.1	42.9	4.3	1.39
10pt88	68.3	69	0.7	48.7	5.7	2.07
10pt89	69	69.4	0.4	44.7	-4.0	0.85
10pt90	69.4	71.2	1.8	49.8	5.1	2.75
10pt91	71.2	71.6	0.4	45.7	-4.1	4.05
10pt92	71.6	72.7	1.1	39.9	-5.8	1
10pt93	72.7	73.2	0.5	46.4	6.5	3.01
10pt94	73.2	73.5	0.3	40.3	-6.0	1.97
10pt95	73.5	73.9	0.4	36.6	-3.7	0.77
10pt96	73.9	75.4	1.5	43.2	6.6	2.31
10pt97	75.4	76.2	0.8	38.9	-4.3	1.1
10pt98	76.2	76.7	0.5	43.1	4.2	1.66
10pt99	76.7	77.7	1	46.9	3.8	1.2
10pt100	77.7	78	0.3	45.5	-1.4	0.35
10pt101	78	78.4	0.4	49.4	3.9	3.03
10pt102	78.4	78.6	0.2	55.1	5.7	0
10pt103	78.6	78.9	0.3	49.4	-5.6	2.02
10pt104	78.9	79.6	0.7	42.1	-7.3	5.14
10pt105	79.6	79.8	0.2	39.7	-2.4	0
10pt106	79.8	80.1	0.3	0.0	-39.7	0
10pt107	80.1	80.4	0.3	42.7	42.7	1.94
10pt108	80.4	80.6	0.2	47.2	4.5	0
10pt109	80.6	80.8	0.2	43.6	-3.6	0
10pt110	80.8	81.1	0.3	38.2	-5.4	1.02
10pt111	81.1	82.1	1	36.4	-1.8	1.38
10pt112	82.1	83.3	1.2	37.4	0.9	0.68
10pt113	83.3	83.6	0.3	36.0	-1.3	0.61
10pt114	83.6	84	0.4	38.1	2.0	1.08
10pt115	84	84.4	0.4	43.8	5.7	2.43
10pt116	84.4	84.6	0.2	38.8	-5.0	0
10pt117	84.6	84.8	0.2	42.1	3.3	0
10pt118	84.8	86	1.2	39.4	-2.7	1.41
10pt119	86	86.2	0.2	42.6	3.2	0
10pt120	86.2	87.5	1.3	45.1	2.5	4.41
10pt121	87.5	89	1.5	38.0	-7.1	1.88
10pt122	89	89.5	0.5	38.2	0.2	1.06
10pt123	89.5	89.7	0.2	41.5	3.3	0

10pt124	89.7	93.4	3.7	39.4	-2.1	1.95
10pt125	93.4	94.2	0.8	42.9	3.6	2.12
10pt126	94.2	94.4	0.2	40.6	-2.4	0
10pt127	94.4	94.6	0.2	41.4	0.8	0
10pt128	94.6	95.4	0.8	38.8	-2.6	0.97
10pt129	95.4	95.9	0.5	43.7	4.9	1.17
10pt130	95.9	96.5	0.6	39.6	-4.2	1.2
10pt131	96.5	97.1	0.6	43.0	3.5	2.11
10pt132	97.1	97.3	0.2	41.0	-2.0	0
10pt133	97.3	98.8	1.5	47.0	6.0	3
10pt134	98.8	99.6	0.8	38.4	-8.6	1.18
10pt135	99.6	100.9	1.3	43.1	4.7	1.82
10pt136	100.9	101.7	0.8	49.2	6.1	2.72
10pt137	101.7	101.9	0.2	39.3	-9.9	0
10pt138	101.9	102.2	0.3	46.4	7.1	2.79
10pt139	102.2	102.4	0.2	43.4	-3.0	0
10pt140	102.4	103.3	0.9	47.6	4.2	2.57
10pt141	103.3	103.8	0.5	42.7	-4.9	1.91
10pt142	103.8	104.3	0.5	50.3	7.6	2.72
10pt143	104.3	105.4	1.1	42.1	-8.2	1.85
10pt144	105.4	106.5	1.1	38.6	-3.4	1.11
10pt145	106.5	106.7	0.2	35.9	-2.7	0
10pt146	106.7	107.8	1.1	39.0	3.1	1.39
10pt147	107.8	108.4	0.6	37.0	-2.1	0.86
10pt148	108.4	108.7	0.3	37.3	0.4	0.19
10pt149	108.7	108.9	0.2	36.4	-1.0	0
10pt150	108.9	110.4	1.5	38.3	2.0	1.22
10pt151	110.4	111.5	1.1	42.8	4.5	2.64
10pt152	111.5	111.7	0.2	36.0	-6.8	0
10pt153	111.7	112.7	1	40.4	4.3	2.04
10pt154	112.7	113.1	0.4	42.9	2.5	0.68
10pt155	113.1	113.4	0.3	38.0	-4.9	1.4
10pt156	113.4	115.4	2	43.3	5.2	2.42
10pt157	115.4	115.8	0.4	40.3	-3.0	1.18
10pt158	115.8	116.5	0.7	34.8	-5.4	1.06
10pt159	116.5	116.7	0.2	38.9	4.0	0
10pt160	116.7	117	0.3	43.5	4.7	0.7
10pt161	117	117.3	0.3	38.1	-5.4	1.65
10pt162	117.3	117.6	0.3	45.9	7.8	2.59
10pt163	117.6	117.8	0.2	38.9	-7.0	0
10pt164	117.8	118.3	0.5	44.7	5.8	4.44
10pt165	118.3	118.7	0.4	43.7	-1.0	1.93
10pt166	118.7	119.2	0.5	38.6	-5.1	0.9
10pt167	119.2	119.9	0.7	43.9	5.3	2.43
10pt168	119.9	120.5	0.6	48.0	4.1	2.83
10pt169	120.5	120.7	0.2	40.9	-7.2	0
10pt170	120.7	120.9	0.2	41.4	0.5	0
10pt171	120.9	121.1	0.2	40.8	-0.6	0
10pt172	121.1	121.4	0.3	42.3	1.5	1.02
10pt173	121.4	121.7	0.3	40.2	-2.1	0.86
10pt174	121.7	122.9	1.2	43.2	3.0	2.61
10pt175	122.9	123.5	0.6	46.8	3.6	3.48
10pt176	123.5	123.7	0.2	43.0	-3.8	0
10pt177	123.7	123.9	0.2	40.0	-3.0	0
10pt178	123.9	124.5	0.6	45.2	5.2	1.87
10pt179	124.5	125.7	1.2	49.0	3.9	2.88

10pt180	125.7	125.9	0.2	44.1	-5.0	0
10pt181	125.9	126.2	0.3	47.6	3.5	1.42
10pt182	126.2	127.9	1.7	42.6	-5.0	1.08
10pt183	127.9	128.1	0.2	47.5	4.8	0
10pt184	128.1	128.9	0.8	43.2	-4.3	2.48
10pt185	128.9	129.6	0.7	45.9	2.8	2.18
10pt186	129.6	130.8	1.2	43.8	-2.1	1.42
10pt187	130.8	133.4	2.6	47.7	3.8	2.17
10pt188	133.4	133.8	0.4	55.8	8.1	0.84
10pt189	133.8	134	0.2	52.3	-3.4	0
10pt190	134	134.7	0.7	56.9	4.5	2.55
10pt191	134.7	135.1	0.4	47.0	-9.8	2.9
11pt1	0	1.6	1.6	55.5		5.06
11pt2	1.6	1.8	0.2	51.8	-3.7	0
11pt3	1.8	3.2	1.4	54.2	2.4	4.04
11pt4	3.2	3.8	0.6	46.8	-7.4	3.96
11pt5	3.8	4.5	0.7	41.8	-5.1	1.09
11pt6	4.5	4.7	0.2	38.1	-3.7	0
11pt7	4.7	5	0.3	36.2	-1.8	0.46
11pt8	5	6	1	38.8	2.6	1.46
11pt9	6	6.6	0.6	44.5	5.7	2.89
11pt10	6.6	7.3	0.7	38.3	-6.2	1.25
11pt11	7.3	7.5	0.2	44.7	6.4	0
11pt12	7.5	7.8	0.3	39.0	-5.7	1.29
11pt13	7.8	8.3	0.5	46.0	7.0	3.11
11pt14	8.3	8.5	0.2	38.1	-7.9	0
11pt15	8.5	8.7	0.2	46.6	8.5	0
11pt16	8.7	9.7	1	43.3	-3.3	1.26
11pt17	9.7	10.2	0.5	38.4	-4.9	1.26
11pt18	10.2	11.2	1	42.8	4.4	1.54
11pt19	11.2	11.5	0.3	46.6	3.8	0.8
11pt20	11.5	12	0.5	41.5	-5.1	3.74
11pt21	12	12.2	0.2	46.3	4.8	0
11pt22	12.2	13.4	1.2	42.6	-3.8	1.43
11pt23	13.4	13.8	0.4	38.8	-3.8	1.52
11pt24	13.8	14.4	0.6	41.6	2.8	1.4
11pt25	14.4	15	0.6	38.7	-2.9	1.61
11pt26	15	15.2	0.2	43.0	4.4	0
11pt27	15.2	15.5	0.3	39.3	-3.7	0.83
11pt28	15.5	15.9	0.4	42.9	3.6	1.96
11pt29	15.9	16.5	0.6	35.5	-7.4	1.54
11pt30	16.5	16.7	0.2	37.9	2.4	0
11pt31	16.7	17.3	0.6	43.2	5.2	2.76
11pt32	17.3	17.8	0.5	48.7	5.5	1.39
11pt33	17.8	18.8	1	42.5	-6.2	2.44
11pt34	18.8	19	0.2	39.7	-2.8	0
11pt35	19	20.2	1.2	43.6	3.9	1.58
11pt36	20.2	20.5	0.3	38.2	-5.4	1.89
11pt37	20.5	20.7	0.2	42.6	4.4	0
11pt38	20.7	21.6	0.9	38.3	-4.3	1.43
11pt39	21.6	27	5.4	35.8	-2.4	0.91
11pt40	27	28.1	1.1	39.4	3.6	1.19
11pt41	28.1	28.5	0.4	35.3	-4.1	0.4
11pt42	28.5	28.9	0.4	38.2	2.9	0.81
11pt43	28.9	29.8	0.9	36.1	-2.1	0.62

11pt44	29.8	31.2	1.4	38.1	2.0	0.89
11pt45	31.2	32	0.8	36.0	-2.1	1.46
11pt46	32	32.8	0.8	43.2	7.2	1.51
11pt47	32.8	33.6	0.8	39.1	-4.0	1.56
11pt48	33.6	34.4	0.8	42.9	3.8	1.38
11pt49	34.4	34.6	0.2	48.3	5.3	0
11pt50	34.6	34.9	0.3	42.7	-5.6	0.69
11pt51	34.9	35.3	0.4	39.2	-3.5	1.89
11pt52	35.3	36.8	1.5	42.5	3.3	1.16
11pt53	36.8	37.3	0.5	39.0	-3.5	0.88
11pt54	37.3	40.3	3	35.7	-3.3	0.91
11pt55	40.3	40.7	0.4	38.3	2.5	0.97
11pt56	40.7	42.8	2.1	36.5	-1.8	0.53
11pt57	42.8	43.9	1.1	38.9	2.4	1.17
11pt58	43.9	44.1	0.2	41.5	2.6	0
11pt59	44.1	44.3	0.2	38.3	-3.2	0
11pt60	44.3	44.7	0.4	43.1	4.8	2.99
11pt61	44.7	47	2.3	47.6	4.4	3.43
11pt62	47	47.2	0.2	43.4	-4.2	0
11pt63	47.2	48.2	1	46.3	2.9	4.46
11pt64	48.2	48.5	0.3	44.1	-2.2	0.81
11pt65	48.5	48.8	0.3	45.1	1.0	2.42
11pt66	48.8	49.6	0.8	38.2	-6.9	1.1
11pt67	49.6	50	0.4	36.5	-1.6	0.29
11pt68	50	50.5	0.5	39.6	3.1	1.59
11pt69	50.5	50.7	0.2	36.9	-2.7	0
11pt70	50.7	51.1	0.4	38.3	1.4	1.24
11pt71	51.1	55.6	4.5	31.7	-6.6	0
11pt72	55.6	55.8	0.2	53.6	22.0	0
11pt73	55.8	57.6	1.8	44.6	-9.1	4.49
11pt74	57.6	58	0.4	41.0	-3.6	2.38
11pt75	58	59	1	39.0	-1.9	1.06
11pt76	59	60.4	1.4	50.7	11.6	3.06
11pt77	60.4	60.7	0.3	44.4	-6.3	1.45
11pt78	60.7	61.4	0.7	49.2	4.9	1.95
11pt79	61.4	61.8	0.4	39.7	-9.5	1.22
11pt80	61.8	62.2	0.4	44.1	4.3	1.58
11pt81	62.2	62.8	0.6	54.8	10.8	3.32
11pt82	62.8	63	0.2	48.8	-6.0	0
11pt83	63	63.3	0.3	54.9	6.0	1.76
11pt84	63.3	64.5	1.2	51.7	-3.2	3
11pt85	64.5	64.7	0.2	44.7	-7.1	0
11pt86	64.7	66.7	2	50.5	5.9	4.05
11pt87	66.7	66.9	0.2	43.7	-6.8	0
11pt88	66.9	67.4	0.5	50.8	7.1	2.69
11pt89	67.4	70.6	3.2	49.3	-1.4	3.21
11pt90	70.6	70.9	0.3	45.0	-4.3	0.83
11pt91	70.9	71.2	0.3	52.5	7.5	4.21
11pt92	71.2	71.4	0.2	38.2	-14.3	0
11pt93	71.4	71.9	0.5	46.6	8.5	5.18
11pt94	71.9	72.4	0.5	44.3	-2.3	2.3
11pt95	72.4	72.8	0.4	39.4	-5.0	1.87
11pt96	72.8	73.5	0.7	42.9	3.5	2.06
11pt97	73.5	74.2	0.7	49.3	6.5	3.22
11pt98	74.2	74.5	0.3	38.0	-11.3	0.85
11pt99	74.5	74.7	0.2	52.4	14.4	0

11pt100	74.7	75	0.3	41.6	-10.8	1.67
11pt101	75	75.7	0.7	46.8	5.2	5.65
11pt102	75.7	75.9	0.2	40.1	-6.7	0
11pt103	75.9	76.1	0.2	42.6	2.4	0
11pt104	76.1	76.3	0.2	39.5	-3.0	0
11pt105	76.3	76.7	0.4	44.5	5.0	2.68
11pt106	76.7	77.1	0.4	40.0	-4.5	1.14
11pt107	77.1	78.1	1	43.8	3.8	1.22
11pt108	78.1	79.2	1.1	38.3	-5.5	0.97
11pt109	79.2	81	1.8	35.9	-2.4	0.58
11pt110	81	82.5	1.5	39.0	3.2	1.07
11pt111	82.5	84	1.5	36.4	-2.6	0.65
11pt112	84	85.1	1.1	39.4	2.9	1.31
11pt113	85.1	85.6	0.5	41.9	2.5	1.1
11pt114	85.6	86.4	0.8	38.3	-3.5	1.84
11pt115	86.4	86.6	0.2	36.7	-1.6	0
11pt116	86.6	87.3	0.7	37.5	0.8	0.43
11pt117	87.3	88.4	1.1	35.7	-1.8	1.16
11pt118	88.4	88.8	0.4	36.9	1.2	1.24
11pt119	88.8	90.3	1.5	35.4	-1.6	0.84
11pt120	90.3	91.3	1	38.8	3.4	0.74
11pt121	91.3	91.5	0.2	41.7	2.9	0
11pt122	91.5	92.3	0.8	39.5	-2.2	2.28
11pt123	92.3	92.9	0.6	41.4	1.9	1.17
11pt124	92.9	93.1	0.2	39.1	-2.3	0
11pt125	93.1	93.7	0.6	41.5	2.4	1.09
11pt126	93.7	95.1	1.4	38.6	-2.9	1.69
11pt127	95.1	99	3.9	35.5	-3.1	0.91
11pt128	99	101.7	2.7	38.5	3.1	1.23
11pt129	101.7	102.2	0.5	35.6	-2.9	1.23
11pt130	102.2	102.7	0.5	38.6	3.0	0.82
11pt131	102.7	104.7	2	35.7	-2.9	0.75
11pt132	104.7	105.1	0.4	38.2	2.5	0.81
11pt133	105.1	106.1	1	36.7	-1.5	1.47
11pt134	106.1	106.4	0.3	38.4	1.7	1.12
11pt135	106.4	106.7	0.3	42.4	3.9	1.2
11pt136	106.7	109	2.3	38.9	-3.5	1.27
11pt137	109	109.3	0.3	41.3	2.4	0.15
11pt138	109.3	110	0.7	38.9	-2.4	1.13
11pt139	110	110.3	0.3	42.6	3.6	1.17
11pt140	110.3	110.5	0.2	40.0	-2.6	0
11pt141	110.5	111.7	1.2	42.7	2.8	1.69
11pt142	111.7	111.9	0.2	40.1	-2.7	0
11pt143	111.9	112.8	0.9	43.9	3.9	1.87
11pt144	112.8	113.1	0.3	47.2	3.2	1.2
11pt145	113.1	113.5	0.4	38.6	-8.6	1.42
11pt146	113.5	114	0.5	42.3	3.7	1.36
11pt147	114	114.3	0.3	38.2	-4.0	1.48
11pt148	114.3	115.2	0.9	44.4	6.2	2.22
11pt149	115.2	115.7	0.5	46.9	2.5	1.34
11pt150	115.7	115.9	0.2	39.6	-7.3	0
11pt151	115.9	117.1	1.2	48.2	8.6	2.29
11pt152	117.1	117.4	0.3	42.4	-5.9	0.36
11pt153	117.4	120	2.6	47.5	5.2	4.03
11pt154	120	120.6	0.6	42.4	-5.1	1.32
11pt155	120.6	121.5	0.9	39.3	-3.1	1.02

11pt156	121.5	122.7	1.2	44.2	4.9	1.8
11pt157	122.7	123.6	0.9	37.9	-6.3	1.09
11pt158	123.6	124.1	0.5	43.4	5.5	2.84
11pt159	124.1	124.6	0.5	46.3	2.9	2.55
11pt160	124.6	124.9	0.3	39.9	-6.5	0.48
11pt161	124.9	125.1	0.2	43.2	3.3	0
11pt162	125.1	125.6	0.5	49.5	6.3	2.83
11pt163	125.6	126.3	0.7	43.5	-6.0	1.87
11pt164	126.3	127.5	1.2	38.4	-5.1	1.24
11pt165	127.5	128	0.5	45.2	6.7	2.44
11pt166	128	128.3	0.3	37.5	-7.6	1.31
11pt167	128.3	130	1.7	43.4	5.9	2.42
11pt168	130	130.2	0.2	40.6	-2.8	0
11pt169	130.2	131.6	1.4	43.1	2.5	1.21
11pt170	131.6	132	0.4	40.3	-2.8	0.53
11pt171	132	133	1	42.8	2.5	1.07
11pt172	133	133.2	0.2	50.5	7.7	0
11pt173	133.2	133.5	0.3	44.2	-6.3	0.75
11pt174	133.5	133.8	0.3	50.5	6.3	2.73
11pt175	133.8	134.3	0.5	45.0	-5.5	1
12pt1	0	0.7	0.7	47.0		4.64
12pt2	0.7	1.1	0.4	41.2	-5.8	2.98
12pt3	1.1	1.5	0.4	38.6	-2.6	1.02
12pt4	1.5	2.2	0.7	45.3	6.7	2.09
12pt5	2.2	3.8	1.6	48.4	3.1	2.44
12pt6	3.8	4.6	0.8	43.4	-5.0	1.76
12pt7	4.6	4.9	0.3	39.5	-3.9	1.48
12pt8	4.9	6.2	1.3	44.0	4.6	1.34
12pt9	6.2	7.5	1.3	49.2	5.1	1.92
12pt10	7.5	7.9	0.4	38.3	-10.9	1.18
12pt11	7.9	9.3	1.4	43.4	5.1	1.98
12pt12	9.3	9.7	0.4	38.4	-5.0	1.21
12pt13	9.7	9.9	0.2	43.8	5.4	0
12pt14	9.9	10.7	0.8	38.3	-5.6	1.11
12pt15	10.7	10.9	0.2	35.5	-2.8	0
12pt16	10.9	11.3	0.4	37.4	1.9	0.99
12pt17	11.3	11.6	0.3	36.2	-1.2	0.44
12pt18	11.6	11.8	0.2	39.1	2.9	0
12pt19	11.8	14	2.2	42.3	3.3	1.51
12pt20	14	14.6	0.6	40.0	-2.3	0.55
12pt21	14.6	14.8	0.2	42.5	2.5	0
12pt22	14.8	16.9	2.1	38.0	-4.6	1.2
12pt23	16.9	19.3	2.4	35.6	-2.4	0.68
12pt24	19.3	20.7	1.4	38.7	3.2	1.8
12pt25	20.7	21.3	0.6	35.8	-2.9	0.62
12pt26	21.3	24.5	3.2	39.0	3.2	1.34
12pt27	24.5	25.2	0.7	41.9	2.9	0.95
12pt28	25.2	25.8	0.6	39.5	-2.3	0.99
12pt29	25.8	26	0.2	36.7	-2.8	0
12pt30	26	26.6	0.6	38.3	1.6	1.12
12pt31	26.6	26.9	0.3	42.7	4.4	1.41
12pt32	26.9	30.9	4	36.0	-6.7	1.22
12pt33	30.9	31.7	0.8	39.1	3.1	0.75
12pt34	31.7	32.7	1	48.4	9.3	3.59
12pt35	32.7	33.1	0.4	44.5	-3.9	2.14

12pt36	33.1	33.7	0.6	47.8	3.2	1.12
12pt37	33.7	34.1	0.4	45.5	-2.3	3.48
12pt38	34.1	34.8	0.7	37.7	-7.7	1.57
12pt39	34.8	35.3	0.5	45.8	8.1	3.09
12pt40	35.3	35.6	0.3	50.6	4.8	2.2
12pt41	35.6	36	0.4	43.8	-6.8	2.87
12pt42	36	37.9	1.9	49.0	5.2	2.3
12pt43	37.9	38.1	0.2	40.1	-8.9	0
12pt44	38.1	39.5	1.4	43.2	3.2	1.99
12pt45	39.5	41	1.5	47.8	4.6	2.79
12pt46	41	41.2	0.2	42.5	-5.4	0
12pt47	41.2	41.7	0.5	40.2	-2.3	1.22
12pt48	41.7	42.1	0.4	48.6	8.4	4.33
12pt49	42.1	43.8	1.7	39.4	-9.2	1.2
12pt50	43.8	44	0.2	35.9	-3.5	0
12pt51	44	44.8	0.8	39.2	3.3	0.88
12pt52	44.8	45.3	0.5	36.4	-2.8	0.64
12pt53	45.3	45.5	0.2	37.7	1.3	0
12pt54	45.5	45.7	0.2	36.1	-1.5	0
12pt55	45.7	46.1	0.4	38.2	2.1	1.1
12pt56	46.1	46.8	0.7	36.3	-1.9	0.62
12pt57	46.8	48.4	1.6	39.1	2.8	1.52
12pt58	48.4	49.2	0.8	35.8	-3.3	0.79
12pt59	49.2	49.4	0.2	38.5	2.7	0
12pt60	49.4	50.3	0.9	36.2	-2.3	0.8
12pt61	50.3	51	0.7	37.6	1.4	1.12
12pt62	51	51.7	0.7	36.5	-1.1	1.02
12pt63	51.7	51.9	0.2	42.4	5.9	0
12pt64	51.9	52.7	0.8	38.0	-4.3	1.97
12pt65	52.7	54	1.3	0.0	-38.0	0
12pt66	54	54.4	0.4	37.7	37.7	1.04
12pt67	54.4	54.6	0.2	51.9	14.1	0
12pt68	54.6	56.7	2.1	38.5	-13.4	2.25
12pt69	56.7	57.8	1.1	42.5	4.0	1.59
12pt70	57.8	58.1	0.3	47.1	4.6	2.93
12pt71	58.1	60.4	2.3	38.6	-8.5	0.96
12pt72	60.4	60.9	0.5	35.3	-3.3	0.92
12pt73	60.9	62.7	1.8	39.2	3.8	1.32
12pt74	62.7	62.9	0.2	41.1	2.0	0
12pt75	62.9	64.7	1.8	38.8	-2.3	1.32
12pt76	64.7	65.8	1.1	35.6	-3.2	1.15
12pt77	65.8	67.6	1.8	37.6	1.9	1.15
12pt78	67.6	68.1	0.5	35.9	-1.7	0.75
12pt79	68.1	69	0.9	39.1	3.2	1.69
12pt80	69	69.2	0.2	41.4	2.2	0
12pt81	69.2	70.5	1.3	39.7	-1.7	1.54
12pt82	70.5	70.7	0.2	35.8	-3.9	0
12pt83	70.7	71.6	0.9	38.1	2.3	1.31
12pt84	71.6	71.8	0.2	36.5	-1.6	0
12pt85	71.8	72.7	0.9	38.3	1.8	0.45
12pt86	72.7	75.9	3.2	35.2	-3.2	0.89
12pt87	75.9	76.6	0.7	38.9	3.8	1.25
12pt88	76.6	76.8	0.2	41.6	2.7	0
12pt89	76.8	77.9	1.1	38.5	-3.0	1.78
12pt90	77.9	80	2.1	35.6	-2.9	1.13
12pt91	80	80.8	0.8	38.0	2.4	1.84

12pt92	80.8	83.3	2.5	35.6	-2.4	0.95
12pt93	83.3	83.6	0.3	38.2	2.6	0.31
12pt94	83.6	83.8	0.2	36.3	-1.8	0
12pt95	83.8	84	0.2	37.3	1.0	0
12pt96	84	89.8	5.8	35.2	-2.2	1.1
12pt97	89.8	91	1.2	38.2	3.1	1.32
12pt98	91	92.7	1.7	35.7	-2.6	0.9
12pt99	92.7	93.8	1.1	38.7	3.1	1.37
12pt100	93.8	94.6	0.8	41.5	2.8	0.57
12pt101	94.6	94.8	0.2	40.2	-1.3	0
12pt102	94.8	95.3	0.5	42.4	2.3	1.49
12pt103	95.3	95.5	0.2	38.6	-3.8	0
12pt104	95.5	96.7	1.2	41.8	3.2	1.39
12pt105	96.7	96.9	0.2	39.4	-2.4	0
12pt106	96.9	97.5	0.6	41.9	2.5	2.64
12pt107	97.5	99.4	1.9	38.6	-3.3	1.02
12pt108	99.4	99.9	0.5	41.8	3.2	1.24
12pt109	99.9	100.3	0.4	38.8	-3.0	0.8
12pt110	100.3	100.9	0.6	36.3	-2.5	0.5
12pt111	100.9	102.9	2	39.6	3.3	1.43
12pt112	102.9	103.1	0.2	42.4	2.8	0
12pt113	103.1	103.9	0.8	38.6	-3.9	1.81
12pt114	103.9	104.1	0.2	41.4	2.8	0
12pt115	104.1	104.7	0.6	38.2	-3.2	1.05
12pt116	104.7	105.9	1.2	43.2	5.0	2.19
12pt117	105.9	106.4	0.5	39.1	-4.1	1.47
12pt118	106.4	107.5	1.1	42.3	3.2	1.37
12pt119	107.5	107.7	0.2	40.1	-2.2	0
12pt120	107.7	108	0.3	42.1	2.0	0.35
12pt121	108	108.4	0.4	39.6	-2.5	0.94
12pt122	108.4	110	1.6	44.6	5.1	2.2
12pt123	110	110.2	0.2	47.3	2.7	0
12pt124	110.2	110.4	0.2	44.3	-3.1	0
12pt125	110.4	111.2	0.8	48.4	4.1	3.26
12pt126	111.2	112.3	1.1	44.6	-3.8	2.07
12pt127	112.3	113.1	0.8	47.5	2.8	3.08
12pt128	113.1	114.4	1.3	43.8	-3.7	1.87
12pt129	114.4	115.4	1	49.4	5.6	1.76
12pt130	115.4	116	0.6	44.0	-5.4	1.31
12pt131	116	116.2	0.2	46.8	2.8	0
12pt132	116.2	117.4	1.2	42.7	-4.1	0.99
12pt133	117.4	117.7	0.3	37.1	-5.6	0.33
12pt134	117.7	119	1.3	46.4	9.3	1.75
12pt135	119	119.7	0.7	44.6	-1.8	1.1
12pt136	119.7	119.9	0.2	40.1	-4.5	0
12pt137	119.9	121.6	1.7	43.2	3.1	1.03
12pt138	121.6	124.1	2.5	48.2	5.0	2.65
12pt139	124.1	124.5	0.4	43.4	-4.9	1.68
12pt140	124.5	125.5	1	48.2	4.8	2.66
12pt141	125.5	125.7	0.2	44.5	-3.7	0
12pt142	125.7	126.2	0.5	46.9	2.3	1.95
12pt143	126.2	126.5	0.3	56.3	9.5	2.4
12pt144	126.5	127.2	0.7	49.8	-6.6	1.74
12pt145	127.2	128.1	0.9	42.1	-7.7	1.28
12pt146	128.1	129.1	1	40.3	-1.8	0.71
12pt147	129.1	130.7	1.6	42.8	2.5	1.82

12pt148	130.7	131	0.3	46.1	3.3	0.12
12pt149	131	132	1	42.9	-3.2	1.27
12pt150	132	132.2	0.2	48.2	5.3	0
12pt151	132.2	132.4	0.2	43.9	-4.3	0
12pt152	132.4	134.3	1.9	49.5	5.6	2.5
12pt153	134.3	134.8	0.5	58.3	8.8	3.2
12pt154	134.8	135.1	0.3	50.5	-7.8	2.74
12pt155	135.1	135.4	0.3	41.5	-9.0	1.45
13pt1	0	17.9	17.9	0.0		0
13pt2	17.9	18.3	0.4	41.5		2.26
13pt3	18.3	18.5	0.2	39.8	-1.8	0
13pt4	18.5	18.7	0.2	44.9	5.1	0
13pt5	18.7	19.1	0.4	39.7	-5.2	0.29
13pt6	19.1	20.9	1.8	42.7	3.0	2.5
13pt7	20.9	21.2	0.3	39.6	-3.1	0.38
13pt8	21.2	21.8	0.6	42.1	2.5	1.27
13pt9	21.8	23.2	1.4	39.4	-2.7	2.07
13pt10	23.2	25.1	1.9	42.3	2.9	1.87
13pt11	25.1	25.5	0.4	40.5	-1.8	0.59
13pt12	25.5	25.7	0.2	43.3	2.8	0
13pt13	25.7	26.1	0.4	39.4	-3.9	1.58
13pt14	26.1	27.2	1.1	43.5	4.1	2.58
13pt15	27.2	27.4	0.2	39.0	-4.5	0
13pt16	27.4	27.8	0.4	44.7	5.7	1.84
13pt17	27.8	29	1.2	40.6	-4.2	1.6
13pt18	29	29.3	0.3	44.1	3.5	2.69
13pt19	29.3	29.6	0.3	40.1	-3.9	0.66
13pt20	29.6	31	1.4	43.3	3.1	2.32
13pt21	31	32.6	1.6	39.0	-4.2	1.24
13pt22	32.6	32.8	0.2	41.9	2.9	0
13pt23	32.8	34.7	1.9	39.0	-2.9	1.17
13pt24	34.7	35.4	0.7	35.0	-4.0	1.32
13pt25	35.4	37.3	1.9	39.2	4.2	1.3
13pt26	37.3	38.5	1.2	35.7	-3.5	0.62
13pt27	38.5	38.8	0.3	38.0	2.3	0.73
13pt28	38.8	40.4	1.6	50.4	12.4	0
13pt29	40.4	40.6	0.2	47.5	-2.9	0
13pt30	40.6	46.3	5.7	47.3	-0.2	6.67
13pt31	46.3	46.5	0.2	41.9	-5.4	0
13pt32	46.5	49.4	2.9	38.9	-3.0	1.52
13pt33	49.4	49.9	0.5	42.0	3.1	2.08
13pt34	49.9	50.9	1	39.3	-2.7	1.01
13pt35	50.9	51.3	0.4	42.7	3.3	0.84
13pt36	51.3	51.7	0.4	39.4	-3.2	1.52
13pt37	51.7	52	0.3	43.9	4.5	2.38
13pt38	52	52.3	0.3	40.1	-3.8	0.29
13pt39	52.3	52.9	0.6	42.4	2.3	1.89
13pt40	52.9	53.4	0.5	39.4	-3.0	1.13
13pt41	53.4	59.1	5.7	34.8	-4.6	1.07
13pt42	59.1	59.6	0.5	39.0	4.2	0.66
13pt43	59.6	60.1	0.5	35.8	-3.2	0.73
13pt44	60.1	60.3	0.2	39.1	3.3	0
13pt45	60.3	60.5	0.2	36.7	-2.4	0
13pt46	60.5	60.9	0.4	38.2	1.5	1.15
13pt47	60.9	72.7	11.8	34.6	-3.6	0.98

13pt48	72.7	73	0.3	37.7	3.2	0.24
13pt49	73	73.2	0.2	36.7	-1.0	0
13pt50	73.2	76.2	3	38.3	1.6	1.31
13pt51	76.2	76.9	0.7	36.4	-1.9	0.65
13pt52	76.9	77.4	0.5	39.4	3.0	1.54
13pt53	77.4	77.6	0.2	35.2	-4.2	0
13pt54	77.6	78.3	0.7	37.9	2.7	0.75
13pt55	78.3	78.7	0.4	36.3	-1.6	0.78
13pt56	78.7	80.9	2.2	38.0	1.7	1.12
13pt57	80.9	94.8	13.9	35.0	-2.9	1.13
13pt58	94.8	95.9	1.1	39.8	4.8	1.09
13pt59	95.9	96.3	0.4	43.7	3.9	1.73
13pt60	96.3	97.3	1	38.4	-5.3	1.16
13pt61	97.3	97.5	0.2	36.8	-1.6	0
13pt62	97.5	98.8	1.3	40.1	3.3	0.99
13pt63	98.8	100.9	2.1	43.8	3.7	1.84
13pt64	100.9	101.3	0.4	39.4	-4.4	1.28
13pt65	101.3	101.7	0.4	43.6	4.2	1.3
13pt66	101.7	102.2	0.5	39.2	-4.4	1.32
13pt67	102.2	102.8	0.6	36.5	-2.7	0.41
13pt68	102.8	104.4	1.6	38.5	2.0	1.12
13pt69	104.4	106.7	2.3	35.9	-2.6	0.76
13pt70	106.7	110.7	4	38.7	2.9	1.49
13pt71	110.7	111.5	0.8	43.6	4.9	1.71
13pt72	111.5	111.8	0.3	49.0	5.4	1.83
13pt73	111.8	112.3	0.5	44.1	-5.0	0.65
13pt74	112.3	112.9	0.6	47.3	3.2	1.11
13pt75	112.9	113.2	0.3	0.0	-47.3	0
13pt76	113.2	113.8	0.6	47.4	47.4	1.55
13pt77	113.8	114.1	0.3	44.3	-3.1	1.36
13pt78	114.1	114.4	0.3	49.8	5.6	0.98
13pt79	114.4	114.6	0.2	54.3	4.5	0
13pt80	114.6	115.9	1.3	51.7	-2.7	4.85
14pt1	0	18	18	0.0		0
14pt2	18	18.2	0.2	35.4		0
14pt3	18.2	18.5	0.3	46.2	10.8	8.2
14pt4	18.5	18.8	0.3	39.4	-6.8	1.98
14pt5	18.8	19.1	0.3	36.1	-3.4	0.67
14pt6	19.1	19.3	0.2	39.0	2.9	0
14pt7	19.3	20.5	1.2	43.8	4.8	2.32
14pt8	20.5	21.3	0.8	38.6	-5.2	1.13
14pt9	21.3	21.7	0.4	43.0	4.4	1.45
14pt10	21.7	22.1	0.4	48.1	5.1	0.35
14pt11	22.1	22.9	0.8	44.8	-3.3	4.19
14pt12	22.9	23.4	0.5	49.7	4.9	1.67
14pt13	23.4	24.6	1.2	38.9	-10.8	1.17
14pt14	24.6	28.8	4.2	35.6	-3.3	1.11
14pt15	28.8	29.5	0.7	37.8	2.2	0.57
14pt16	29.5	29.7	0.2	36.2	-1.6	0
14pt17	29.7	32.1	2.4	39.0	2.9	1.56
14pt18	32.1	32.3	0.2	37.0	-2.1	0
14pt19	32.3	32.8	0.5	38.3	1.3	0.67
14pt20	32.8	34.5	1.7	42.1	3.8	2.07
14pt21	34.5	34.9	0.4	37.5	-4.6	2.15
14pt22	34.9	35.6	0.7	38.9	1.3	1.08

14pt23	35.6	35.8	0.2	42.5	3.6	0
14pt24	35.8	36.4	0.6	38.4	-4.0	0.82
14pt25	36.4	36.9	0.5	36.8	-1.6	2.66
14pt26	36.9	37.5	0.6	37.7	0.8	0.33
14pt27	37.5	37.7	0.2	36.6	-1.0	0
14pt28	37.7	39.1	1.4	38.5	1.9	1.33
14pt29	39.1	43.5	4.4	34.7	-3.8	0.84
14pt30	43.5	44.7	1.2	37.9	3.2	0.59
14pt31	44.7	48.2	3.5	35.4	-2.5	1.06
14pt32	48.2	48.7	0.5	39.4	4.0	1.02
14pt33	48.7	49.3	0.6	42.9	3.5	2.06
14pt34	49.3	50	0.7	39.3	-3.6	2.1
14pt35	50	50.2	0.2	42.9	3.6	0
14pt36	50.2	53.5	3.3	39.6	-3.3	1.25
14pt37	53.5	54.4	0.9	42.0	2.4	1.84
14pt38	54.4	54.6	0.2	40.7	-1.3	0
14pt39	54.6	54.8	0.2	41.7	1.0	0
14pt40	54.8	55	0.2	38.2	-3.6	0
14pt41	55	55.3	0.3	41.7	3.6	0.87
14pt42	55.3	55.9	0.6	39.8	-2.0	0.55
14pt43	55.9	56.2	0.3	41.8	2.0	0.84
14pt44	56.2	60.5	4.3	39.0	-2.8	1.48
14pt45	60.5	61	0.5	42.7	3.7	0.67
14pt46	61	61.9	0.9	38.7	-4.0	1.22
14pt47	61.9	62.5	0.6	36.7	-2.1	0.49
14pt48	62.5	62.9	0.4	39.2	2.6	1.49
14pt49	62.9	63.3	0.4	41.9	2.6	0.96
14pt50	63.3	64	0.7	41.1	-0.7	1.75
14pt51	64	64.9	0.9	45.3	4.2	2.75
14pt52	64.9	65.2	0.3	37.1	-8.3	0.95
14pt53	65.2	65.6	0.4	42.7	5.6	0.82
14pt54	65.6	66.2	0.6	38.9	-3.8	1.9
14pt55	66.2	66.7	0.5	36.0	-3.0	0.76
14pt56	66.7	66.9	0.2	39.3	3.3	0
14pt57	66.9	67.4	0.5	44.5	5.2	2.48
14pt58	67.4	67.9	0.5	38.2	-6.3	1.54
14pt59	67.9	68.1	0.2	42.3	4.1	0
14pt60	68.1	68.6	0.5	47.5	5.1	1.15
14pt61	68.6	69.9	1.3	44.0	-3.4	1.98
14pt62	69.9	70.3	0.4	40.0	-4.0	0.33
14pt63	70.3	70.5	0.2	44.6	4.5	0
14pt64	70.5	70.8	0.3	38.5	-6.1	2.62
14pt65	70.8	71	0.2	43.5	5.0	0
14pt66	71	71.3	0.3	38.9	-4.6	0.5
14pt67	71.3	71.6	0.3	41.5	2.6	0.35
14pt68	71.6	71.8	0.2	40.0	-1.5	0
14pt69	71.8	73.5	1.7	44.4	4.4	2.98
14pt70	73.5	73.9	0.4	42.8	-1.6	1.83
14pt71	73.9	74.7	0.8	46.8	4.0	3.7
14pt72	74.7	74.9	0.2	43.1	-3.6	0
14pt73	74.9	75.1	0.2	46.3	3.1	0
14pt74	75.1	75.4	0.3	45.6	-0.7	0.22
14pt75	75.4	76.1	0.7	42.1	-3.6	3.44
14pt76	76.1	76.3	0.2	48.5	6.4	0
14pt77	76.3	77.1	0.8	48.1	-0.4	2.31
14pt78	77.1	77.6	0.5	43.3	-4.8	1.01

14pt79	77.6	77.8	0.2	46.9	3.6	0
14pt80	77.8	78.2	0.4	43.4	-3.5	1.52
14pt81	78.2	80.4	2.2	38.6	-4.8	1.12
14pt82	80.4	80.8	0.4	36.1	-2.5	0.21
14pt83	80.8	82	1.2	39.7	3.6	1.62
14pt84	82	85.2	3.2	35.7	-4.0	0.74
14pt85	85.2	85.8	0.6	37.8	2.2	0.83
14pt86	85.8	87.3	1.5	36.2	-1.7	0.73
14pt87	87.3	88.2	0.9	39.0	2.9	1.5
14pt88	88.2	88.4	0.2	43.6	4.6	0
14pt89	88.4	89	0.6	38.9	-4.7	1.43
14pt90	89	89.9	0.9	43.6	4.8	1.15
14pt91	89.9	90.1	0.2	40.6	-3.0	0
14pt92	90.1	90.6	0.5	44.0	3.4	1.29
14pt93	90.6	91	0.4	47.0	3.0	2.97
14pt94	91	91.2	0.2	38.6	-8.4	0
14pt95	91.2	91.7	0.5	47.2	8.6	4.11
14pt96	91.7	92	0.3	38.7	-8.5	2.22
14pt97	92	92.5	0.5	43.0	4.3	1.92
14pt98	92.5	93.5	1	48.1	5.0	3.63
14pt99	93.5	93.9	0.4	39.3	-8.8	1.31
14pt100	93.9	94.3	0.4	49.2	10.0	2.16
14pt101	94.3	94.7	0.4	41.7	-7.6	3.42
14pt102	94.7	95.2	0.5	47.3	5.6	1.07
14pt103	95.2	95.5	0.3	43.4	-4.0	1.39
14pt104	95.5	96	0.5	47.1	3.8	1.97
14pt105	96	96.3	0.3	44.5	-2.6	0.86
14pt106	96.3	96.9	0.6	43.7	-0.8	3.67
14pt107	96.9	97.1	0.2	46.5	2.8	0
14pt108	97.1	97.9	0.8	42.5	-4.0	2.62
14pt109	97.9	98.1	0.2	40.5	-2.0	0
14pt110	98.1	98.7	0.6	41.8	1.2	1.08
14pt111	98.7	99.2	0.5	41.2	-0.5	0.99
14pt112	99.2	100.2	1	48.6	7.3	3.29
14pt113	100.2	100.5	0.3	43.5	-5.1	1.46
14pt114	100.5	101.3	0.8	51.2	7.7	2.51
14pt115	101.3	101.5	0.2	42.7	-8.5	0
14pt116	101.5	101.7	0.2	48.1	5.4	0
14pt117	101.7	101.9	0.2	43.6	-4.6	0
14pt118	101.9	102.1	0.2	50.8	7.2	0
14pt119	102.1	102.3	0.2	43.6	-7.2	0
14pt120	102.3	103.8	1.5	48.2	4.5	2.76
14pt121	103.8	104	0.2	43.8	-4.4	0
14pt122	104	104.6	0.6	48.8	5.0	3.44
14pt123	104.6	104.9	0.3	56.5	7.7	3.93
14pt124	104.9	105.1	0.2	50.5	-6.0	0
14pt125	105.1	106.4	1.3	55.5	5.0	3.27
14pt126	106.4	106.6	0.2	47.0	-8.5	0
14pt127	106.6	106.8	0.2	0.0	-47.0	0
14pt128	106.8	107.4	0.6	41.6	41.6	2.24
15pt1	0	18.2	18.2	0.0		0
15pt2	18.2	18.4	0.2	37.7		0
15pt3	18.4	18.9	0.5	43.7	6.0	5.43
15pt4	18.9	19.2	0.3	51.0	7.3	0
15pt5	19.2	19.7	0.5	46.1	-4.9	5.91

15pt6	19.7	20.2	0.5	0.0		0
15pt7	20.2	20.6	0.4	43.7		4.34
15pt8	20.6	20.9	0.3	43.2	-0.5	2.56
15pt9	20.9	22.4	1.5	39.7	-3.5	0.97
15pt10	22.4	22.7	0.3	44.8	5.1	5.03
15pt11	22.7	22.9	0.2	37.4	-7.4	0
15pt12	22.9	23.7	0.8	43.3	5.9	2.03
15pt13	23.7	25.3	1.6	40.8	-2.5	1.41
15pt14	25.3	27.6	2.3	43.3	2.5	2.71
15pt15	27.6	28.2	0.6	0.0		0
15pt16	28.2	28.7	0.5	44.0		2.9
15pt17	28.7	28.9	0.2	40.3	-3.7	0
15pt18	28.9	29.3	0.4	43.3	3.1	1.84
15pt19	29.3	30	0.7	40.2	-3.2	0.75
15pt20	30	30.2	0.2	41.3	1.1	0
15pt21	30.2	30.4	0.2	40.2	-1.1	0
15pt22	30.4	31.3	0.9	41.8	1.6	1.73
15pt23	31.3	32.2	0.9	39.4	-2.4	1.9
15pt24	32.2	32.7	0.5	36.4	-3.0	1
15pt25	32.7	32.9	0.2	37.3	0.9	0
15pt26	32.9	33.4	0.5	36.2	-1.1	0.27
15pt27	33.4	34.1	0.7	38.1	2.0	0.8
15pt28	34.1	34.7	0.6	36.5	-1.6	0.67
15pt29	34.7	35.5	0.8	38.6	2.0	1.54
15pt30	35.5	35.8	0.3	41.6	3.1	0.56
15pt31	35.8	36.4	0.6	38.9	-2.7	1.03
15pt32	36.4	36.6	0.2	41.3	2.4	0
15pt33	36.6	37	0.4	39.6	-1.7	0.92
15pt34	37	37.5	0.5	48.8	9.2	4.64
15pt35	37.5	37.7	0.2	43.4	-5.3	0
15pt36	37.7	38	0.3	50.1	6.7	1.76
15pt37	38	38.4	0.4	43.5	-6.6	1.58
15pt38	38.4	38.8	0.4	45.5	2.1	5.26
15pt39	38.8	39.2	0.4	49.8	4.3	1.55
15pt40	39.2	39.4	0.2	40.4	-9.4	0
15pt41	39.4	39.9	0.5	44.2	3.8	1.85
15pt42	39.9	40.2	0.3	39.8	-4.4	1.03
15pt43	40.2	41.1	0.9	44.3	4.5	1.92
15pt44	41.1	41.3	0.2	39.4	-4.9	0
15pt45	41.3	41.5	0.2	41.3	2.0	0
15pt46	41.5	41.7	0.2	40.6	-0.7	0
15pt47	41.7	42.2	0.5	41.6	1.0	0.64
15pt48	42.2	42.4	0.2	48.8	7.1	0
15pt49	42.4	43	0.6	42.3	-6.5	1.3
15pt50	43	43.7	0.7	38.3	-4.0	0.97
15pt51	43.7	44.5	0.8	35.9	-2.4	0.69
15pt52	44.5	46.6	2.1	39.1	3.2	1.01
15pt53	46.6	46.9	0.3	35.9	-3.2	0.87
15pt54	46.9	48	1.1	39.5	3.6	1.6
15pt55	48	48.7	0.7	42.1	2.6	1.97
15pt56	48.7	49.2	0.5	39.5	-2.6	1.78
15pt57	49.2	49.6	0.4	43.8	4.4	1.4
15pt58	49.6	51.5	1.9	39.0	-4.9	1.91
15pt59	51.5	52.1	0.6	35.9	-3.1	0.68
15pt60	52.1	53.8	1.7	39.2	3.4	1.65
15pt61	53.8	54.2	0.4	36.7	-2.5	1.68

15pt62	54.2	54.4	0.2	38.8	2.1	0
15pt63	54.4	54.7	0.3	36.7	-2.1	0.05
15pt64	54.7	55.3	0.6	43.0	6.3	1.94
15pt65	55.3	55.7	0.4	39.9	-3.1	1.45
15pt66	55.7	56.1	0.4	42.7	2.8	2.11
15pt67	56.1	56.6	0.5	39.3	-3.3	1.41
15pt68	56.6	57.6	1	42.0	2.6	1.59
15pt69	57.6	58.2	0.6	40.3	-1.7	1.11
15pt70	58.2	58.8	0.6	41.9	1.6	0.73
15pt71	58.8	59.4	0.6	40.2	-1.7	0.94
15pt72	59.4	59.6	0.2	34.4	-5.8	0
15pt73	59.6	61.1	1.5	43.3	8.9	1.6
15pt74	61.1	61.4	0.3	39.0	-4.3	1.53
15pt75	61.4	61.6	0.2	46.7	7.6	0
15pt76	61.6	62.3	0.7	42.7	-4.0	2.04
15pt77	62.3	63.1	0.8	47.9	5.1	2.22
15pt78	63.1	63.3	0.2	42.6	-5.3	0
15pt79	63.3	63.5	0.2	38.9	-3.7	0
15pt80	63.5	63.9	0.4	47.9	9.1	1.99
15pt81	63.9	64.2	0.3	44.7	-3.3	0.96
15pt82	64.2	64.9	0.7	47.8	3.2	1.66
15pt83	64.9	65.5	0.6	39.2	-8.6	1.65
15pt84	65.5	66.3	0.8	44.3	5.1	4.02
15pt85	66.3	66.5	0.2	48.4	4.1	0
15pt86	66.5	67.3	0.8	43.2	-5.2	3.06
15pt87	67.3	68.3	1	47.5	4.3	1.49
15pt88	68.3	68.7	0.4	41.8	-5.7	2.84
15pt89	68.7	68.9	0.2	37.9	-3.9	0
15pt90	68.9	69.6	0.7	42.5	4.6	1.25
15pt91	69.6	69.9	0.3	38.3	-4.2	1.94
15pt92	69.9	70.2	0.3	45.3	7.0	1.52
15pt93	70.2	70.4	0.2	39.6	-5.7	0
15pt94	70.4	70.8	0.4	42.7	3.2	1.29
15pt95	70.8	71.4	0.6	41.5	-1.2	5.14
15pt96	71.4	73.2	1.8	49.6	8.1	3.12
15pt97	73.2	73.4	0.2	44.1	-5.5	0
15pt98	73.4	73.7	0.3	49.4	5.2	6.14
15pt99	73.7	73.9	0.2	38.8	-10.6	0
15pt100	73.9	74.2	0.3	45.5	6.7	2.18
15pt101	74.2	74.8	0.6	37.9	-7.6	1.59
15pt102	74.8	75	0.2	46.0	8.1	0
15pt103	75	75.3	0.3	37.8	-8.1	0.56
15pt104	75.3	75.8	0.5	51.5	13.7	5.8
15pt105	75.8	76.3	0.5	48.0	-3.6	1.47
15pt106	76.3	76.5	0.2	41.8	-6.2	0
15pt107	76.5	77	0.5	48.5	6.7	1.89
15pt108	77	79.4	2.4	43.2	-5.3	2.01
15pt109	79.4	80.3	0.9	40.2	-3.0	2.7
15pt110	80.3	81.6	1.3	43.1	2.9	2.08
15pt111	81.6	82.1	0.5	39.5	-3.7	0.42
15pt112	82.1	82.4	0.3	44.3	4.8	2.42
15pt113	82.4	82.6	0.2	47.5	3.3	0
15pt114	82.6	83	0.4	42.6	-4.9	0.51
15pt115	83	83.3	0.3	39.7	-3.0	0.46
15pt116	83.3	83.8	0.5	44.0	4.4	3.1
15pt117	83.8	85.6	1.8	39.1	-5.0	1.23

15pt118	85.6	86.5	0.9	45.6	6.5	1.76
15pt119	86.5	86.7	0.2	48.3	2.7	0
15pt120	86.7	87.1	0.4	44.9	-3.5	1.02
15pt121	87.1	88	0.9	47.9	3.1	2.62
15pt122	88	88.4	0.4	43.4	-4.5	1.25
15pt123	88.4	88.8	0.4	47.5	4.1	5.04
15pt124	88.8	89.1	0.3	42.6	-4.9	0.78
15pt125	89.1	89.5	0.4	39.8	-2.9	0.99
15pt126	89.5	90.8	1.3	43.6	3.8	2.4
15pt127	90.8	91	0.2	47.1	3.5	0
15pt128	91	91.3	0.3	44.0	-3.1	0.68
15pt129	91.3	94.1	2.8	38.7	-5.3	1.12
15pt130	94.1	94.3	0.2	43.3	4.7	0
15pt131	94.3	96	1.7	38.6	-4.7	1.55
15pt132	96	97.2	1.2	44.0	5.4	1.42
15pt133	97.2	97.9	0.7	40.3	-3.7	4.27
15pt134	97.9	98.3	0.4	46.1	5.7	2.07
15pt135	98.3	99.3	1	44.8	-1.3	3.41
15pt136	99.3	100.1	0.8	44.3	-0.4	5.14
16pt1	0	3.3	3.3	56.2		4.59
16pt2	3.3	4.4	1.1	47.7	-8.5	2.43
16pt3	4.4	4.9	0.5	53.3	5.6	2.32
16pt4	4.9	5.4	0.5	49.8	-3.4	1.9
16pt5	5.4	6.8	1.4	43.1	-6.7	1.82
16pt6	6.8	7.4	0.6	40.9	-2.2	1.12
16pt7	7.4	7.8	0.4	42.0	1.1	0.49
16pt8	7.8	8.3	0.5	40.2	-1.8	0.65
16pt9	8.3	8.8	0.5	41.9	1.6	0.71
16pt10	8.8	9.5	0.7	47.3	5.4	1.45
16pt11	9.5	10.8	1.3	42.6	-4.6	1.69
16pt12	10.8	12.1	1.3	48.6	5.9	1.89
16pt13	12.1	12.4	0.3	44.6	-3.9	1.11
16pt14	12.4	13	0.6	47.2	2.6	0.67
16pt15	13	13.9	0.9	43.1	-4.0	1.32
16pt16	13.9	14.1	0.2	40.8	-2.4	0
16pt17	14.1	14.5	0.4	42.5	1.7	1.47
16pt18	14.5	14.7	0.2	38.6	-3.9	0
16pt19	14.7	15.1	0.4	45.2	6.6	3.51
16pt20	15.1	15.6	0.5	46.8	1.6	1.02
16pt21	15.6	15.8	0.2	44.3	-2.5	0
16pt22	15.8	16.7	0.9	47.7	3.4	1.52
16pt23	16.7	22.4	5.7	43.8	-4.0	2.37
16pt24	22.4	22.6	0.2	48.8	5.0	0
16pt25	22.6	23.6	1	43.3	-5.5	1.42
16pt26	23.6	23.9	0.3	47.2	3.8	0.98
16pt27	23.9	25.7	1.8	43.8	-3.3	1.63
16pt28	25.7	25.9	0.2	40.5	-3.3	0
16pt29	25.9	26.6	0.7	41.7	1.2	0.62
16pt30	26.6	26.9	0.3	39.8	-1.9	1.13
16pt31	26.9	27.4	0.5	42.9	3.2	0.76
16pt32	27.4	29.7	2.3	48.6	5.6	2.09
16pt33	29.7	29.9	0.2	43.6	-5.0	0
16pt34	29.9	32	2.1	50.1	6.5	2.85
16pt35	32	32.4	0.4	40.5	-9.7	1.78
16pt36	32.4	33	0.6	42.6	2.1	1.52

16pt37	33	33.8	0.8	41.6	-0.9	8.22
16pt38	33.8	34.5	0.7	43.0	1.4	6.75
16pt39	34.5	44.2	9.7	0.0	-43.0	0
16pt40	44.2	44.5	0.3	36.1	36.1	0.53
16pt41	44.5	45.3	0.8	39.9	3.7	2.21
16pt42	45.3	45.9	0.6	41.6	1.8	2.19
16pt43	45.9	46.3	0.4	45.6	3.9	2.91
16pt44	46.3	47.1	0.8	38.5	-7.1	1.36
16pt45	47.1	48.7	1.6	43.3	4.8	2.13
16pt46	48.7	49.2	0.5	50.4	7.2	1.09
16pt47	49.2	49.7	0.5	45.1	-5.4	3.13
16pt48	49.7	50	0.3	48.7	3.6	2.32
16pt49	50	50.8	0.8	43.4	-5.3	2.09
16pt50	50.8	52.3	1.5	39.7	-3.6	1.01
16pt51	52.3	52.5	0.2	42.7	3.0	0
16pt52	52.5	53.4	0.9	39.2	-3.5	2.33
16pt53	53.4	54.1	0.7	44.0	4.8	2.16
16pt54	54.1	54.3	0.2	40.8	-3.1	0
16pt55	54.3	56.3	2	43.9	3.1	3.39
16pt56	56.3	57.6	1.3	49.6	5.6	2.82
16pt57	57.6	58.3	0.7	44.6	-5.0	3.45
16pt58	58.3	58.9	0.6	38.7	-5.9	0.67
16pt59	58.9	59.6	0.7	36.6	-2.1	0.48
16pt60	59.6	59.9	0.3	38.2	1.6	0.39
16pt61	59.9	61.5	1.6	36.0	-2.3	0.57
16pt62	61.5	61.7	0.2	38.4	2.4	0
16pt63	61.7	62.1	0.4	36.7	-1.6	0.22
16pt64	62.1	62.3	0.2	37.5	0.8	0
16pt65	62.3	64.3	2	36.4	-1.2	0.74
16pt66	64.3	65.3	1	39.2	2.8	1.38
16pt67	65.3	65.9	0.6	43.4	4.3	0.56
16pt68	65.9	66.7	0.8	47.7	4.3	3.22
16pt69	66.7	66.9	0.2	45.3	-2.3	0
16pt70	66.9	67.8	0.9	51.1	5.7	3.16
16pt71	67.8	68	0.2	43.1	-7.9	0
16pt72	68	68.6	0.6	48.6	5.4	2.03
16pt73	68.6	68.9	0.3	43.5	-5.1	1.13
16pt74	68.9	69.2	0.3	45.0	1.5	3.2
16pt75	69.2	69.7	0.5	42.6	-2.4	2.57
16pt76	69.7	70.6	0.9	47.8	5.2	3.94
16pt77	70.6	70.9	0.3	44.1	-3.7	2.09
16pt78	70.9	71.2	0.3	39.4	-4.7	0.84
16pt79	71.2	72.1	0.9	44.3	4.9	2.3
16pt80	72.1	72.7	0.6	38.1	-6.1	1.9
16pt81	72.7	73.8	1.1	43.2	5.0	2.48
16pt82	73.8	74.1	0.3	39.1	-4.1	0.95
16pt83	74.1	74.4	0.3	45.7	6.6	3.08
16pt84	74.4	75	0.6	44.2	-1.5	2.47
16pt85	75	75.5	0.5	47.2	3.0	3.4
16pt86	75.5	75.7	0.2	44.1	-3.1	0
16pt87	75.7	75.9	0.2	38.7	-5.4	0
16pt88	75.9	76.8	0.9	36.4	-2.3	0.55
16pt89	76.8	77.5	0.7	39.3	2.9	1.9
16pt90	77.5	80.1	2.6	42.5	3.3	1.15
16pt91	80.1	80.4	0.3	40.6	-1.9	0.39
16pt92	80.4	80.8	0.4	42.0	1.4	0.95

16pt93	80.8	81	0.2	39.5	-2.5	0
16pt94	81	81.2	0.2	42.9	3.4	0
16pt95	81.2	82.1	0.9	48.1	5.2	2.62
16pt96	82.1	84.1	2	42.1	-6.0	1.14
16pt97	84.1	85.4	1.3	49.4	7.3	2.04
16pt98	85.4	86	0.6	55.8	6.4	0.92
16pt99	86	88	2	49.1	-6.7	1.96
16pt100	88	89.6	1.6	56.3	7.1	3.84
16pt101	89.6	89.9	0.3	51.2	-5.1	2.28
16pt102	89.9	90.7	0.8	52.8	1.6	4.03
17pt1	0	0.9	0.9	49.6		2.84
17pt2	0.9	1.6	0.7	52.6	3.0	3.19
17pt3	1.6	1.9	0.3	49.8	-2.8	3.41
17pt4	1.9	2.1	0.2	53.6	3.8	0
17pt5	2.1	2.7	0.6	46.0	-7.5	3.43
17pt6	2.7	3.1	0.4	50.6	4.5	2.52
17pt7	3.1	3.4	0.3	40.5	-10.1	0.51
17pt8	3.4	3.6	0.2	41.8	1.4	0
17pt9	3.6	3.9	0.3	51.4	9.6	1.57
17pt10	3.9	4.1	0.2	53.4	2.0	0
17pt11	4.1	4.5	0.4	44.1	-9.3	2.01
17pt12	4.5	4.7	0.2	54.3	10.3	0
17pt13	4.7	5.4	0.7	49.7	-4.6	2.5
17pt14	5.4	6.5	1.1	45.2	-4.5	1.76
17pt15	6.5	6.9	0.4	48.1	2.9	3.28
17pt16	6.9	7.1	0.2	42.1	-6.0	0
17pt17	7.1	7.4	0.3	49.1	7.0	2.44
17pt18	7.4	7.6	0.2	53.8	4.7	0
17pt19	7.6	9.3	1.7	48.5	-5.3	2.76
17pt20	9.3	9.6	0.3	39.0	-9.5	1.95
17pt21	9.6	10.1	0.5	49.3	10.3	1.57
17pt22	10.1	10.7	0.6	42.8	-6.4	1.8
17pt23	10.7	10.9	0.2	49.6	6.8	0
17pt24	10.9	11.5	0.6	42.8	-6.9	3.04
17pt25	11.5	14.3	2.8	49.1	6.4	2.61
17pt26	14.3	14.5	0.2	45.2	-3.9	0
17pt27	14.5	16.1	1.6	49.6	4.4	2.36
17pt28	16.1	16.3	0.2	44.1	-5.6	0
17pt29	16.3	16.9	0.6	39.9	-4.1	0.44
17pt30	16.9	17.6	0.7	49.4	9.4	2.14
17pt31	17.6	17.8	0.2	43.3	-6.1	0
17pt32	17.8	18	0.2	53.6	10.3	0
17pt33	18	18.3	0.3	43.9	-9.7	1.12
17pt34	18.3	19.7	1.4	48.8	4.9	3.65
17pt35	19.7	20.1	0.4	42.4	-6.4	1.12
17pt36	20.1	20.3	0.2	39.4	-3.0	0
17pt37	20.3	20.5	0.2	43.8	4.4	0
17pt38	20.5	21	0.5	48.8	5.0	1.33
17pt39	21	21.7	0.7	43.5	-5.3	1.98
17pt40	21.7	21.9	0.2	40.4	-3.1	0
17pt41	21.9	22.3	0.4	44.3	3.9	2.46
17pt42	22.3	22.8	0.5	48.3	4.0	1
17pt43	22.8	24.3	1.5	44.1	-4.2	1.33
17pt44	24.3	24.6	0.3	39.6	-4.5	1.25
17pt45	24.6	25.7	1.1	44.4	4.8	2.49

17pt46	25.7	25.9	0.2	50.9	6.6	0
17pt47	25.9	26.2	0.3	38.0	-13.0	1.73
17pt48	26.2	27.1	0.9	43.3	5.3	2.71
17pt49	27.1	27.6	0.5	39.6	-3.7	0.55
17pt50	27.6	28.1	0.5	44.3	4.7	4.18
17pt51	28.1	29	0.9	49.6	5.3	2.01
17pt52	29	29.7	0.7	43.9	-5.7	3.49
17pt53	29.7	30	0.3	49.2	5.3	1.82
17pt54	30	30.5	0.5	40.8	-8.4	3.17
17pt55	30.5	32.1	1.6	44.3	3.6	0
17pt56	32.1	32.3	0.2	50.7	6.3	0
17pt57	32.3	33.2	0.9	0.0		0
17pt58	33.2	33.5	0.3	55.0		7.71
17pt59	33.5	34.1	0.6	0.0		0
17pt60	34.1	34.8	0.7	43.1		1.85
17pt61	34.8	35	0.2	53.3	10.2	0
17pt62	35	35.4	0.4	46.3	-7.0	3.85
17pt63	35.4	35.6	0.2	0.0		0
17pt64	35.6	36.5	0.9	44.4		4.12
17pt65	36.5	37.3	0.8	48.7	4.3	3.36
17pt66	37.3	37.7	0.4	41.0	-7.7	4.44
17pt67	37.7	38.1	0.4	51.3	10.4	2.18
17pt68	38.1	38.4	0.3	49.1	-2.2	0.93
17pt69	38.4	38.8	0.4	53.9	4.7	2.55
17pt70	38.8	39.6	0.8	48.4	-5.5	4.16
17pt71	39.6	40.7	1.1	46.3	-2.1	9.76
17pt72	40.7	41.1	0.4	43.1	-3.2	1.81
17pt73	41.1	41.9	0.8	40.4	-2.7	1.41
17pt74	41.9	42.1	0.2	42.6	2.3	0
17pt75	42.1	42.3	0.2	39.2	-3.4	0
17pt76	42.3	42.5	0.2	41.5	2.3	0
17pt77	42.5	43.2	0.7	39.4	-2.1	1.43
17pt78	43.2	43.8	0.6	42.5	3.1	1.76
17pt79	43.8	44.4	0.6	39.4	-3.1	1.75
17pt80	44.4	45.9	1.5	43.1	3.7	1.48
17pt81	45.9	46.1	0.2	39.4	-3.7	0
17pt82	46.1	46.6	0.5	46.9	7.5	1.26
17pt83	46.6	47.2	0.6	43.7	-3.3	1.24
17pt84	47.2	47.8	0.6	48.0	4.3	2.12
17pt85	47.8	48.1	0.3	42.9	-5.1	2.06
17pt86	48.1	48.5	0.4	50.6	7.6	1.67
17pt87	48.5	48.8	0.3	45.1	-5.4	0.44
17pt88	48.8	49.2	0.4	52.8	7.6	2.06
17pt89	49.2	50	0.8	49.0	-3.8	3.36
17pt90	50	50.8	0.8	42.1	-6.9	2.03
17pt91	50.8	51.7	0.9	37.9	-4.2	1.25
17pt92	51.7	53.9	2.2	36.4	-1.5	0.87
17pt93	53.9	54.3	0.4	37.9	1.5	2.01
17pt94	54.3	54.6	0.3	42.6	4.7	0.04
17pt95	54.6	55.6	1	39.7	-2.9	0.84
17pt96	55.6	56.6	1	44.4	4.7	1.76
17pt97	56.6	57.1	0.5	47.8	3.4	1.95
17pt98	57.1	57.9	0.8	45.2	-2.6	1.68
17pt99	57.9	58.3	0.4	39.6	-5.6	0.53
17pt100	58.3	58.8	0.5	43.6	4.0	1.83
17pt101	58.8	59.6	0.8	41.5	-2.1	3.38

17pt102	59.6	60	0.4	42.1	0.5	0.59
17pt103	60	60.5	0.5	37.9	-4.1	1.58
17pt104	60.5	60.8	0.3	47.1	9.2	3.51
17pt105	60.8	61.3	0.5	37.4	-9.8	1.43
17pt106	61.3	62.2	0.9	43.8	6.4	2.55
17pt107	62.2	62.6	0.4	37.0	-6.8	0.94
17pt108	62.6	63.3	0.7	47.8	10.8	5.06
17pt109	63.3	63.8	0.5	45.0	-2.8	1.15
17pt110	63.8	64	0.2	40.5	-4.5	0
17pt111	64	64.9	0.9	45.1	4.6	2.41
17pt112	64.9	65.4	0.5	37.5	-7.6	0.46
17pt113	65.4	66	0.6	43.7	6.3	1.18
17pt114	66	66.3	0.3	48.3	4.6	0.94
17pt115	66.3	68.1	1.8	43.6	-4.8	2.43
17pt116	68.1	69.8	1.7	38.0	-5.6	1.68
17pt117	69.8	70.3	0.5	36.6	-1.4	0.29
17pt118	70.3	71.4	1.1	38.0	1.4	1.17
17pt119	71.4	71.8	0.4	43.9	5.9	1.79
17pt120	71.8	72.2	0.4	47.3	3.4	0.96
17pt121	72.2	72.6	0.4	45.4	-1.9	0.41
17pt122	72.6	73.7	1.1	49.3	3.8	2.31
17pt123	73.7	73.9	0.2	53.3	4.0	0
17pt124	73.9	74.3	0.4	49.3	-3.9	2.16
17pt125	74.3	74.5	0.2	54.6	5.3	0
17pt126	74.5	75.1	0.6	49.6	-5.0	3.06
17pt127	75.1	75.5	0.4	53.9	4.2	1.43
17pt128	75.5	77.8	2.3	49.8	-4.1	2.88
17pt129	77.8	78	0.2	54.3	4.5	0
17pt130	78	78.6	0.6	50.1	-4.2	2.58
17pt131	78.6	79.9	1.3	53.8	3.7	1.44
17pt132	79.9	81	1.1	49.7	-4.1	3.05
17pt133	81	82.7	1.7	56.8	7.0	2.47
17pt134	82.7	82.9	0.2	50.6	-6.2	0
17pt135	82.9	83.4	0.5	52.8	2.2	2.27
18pt1	0	1.4	1.4	0.0		0
18pt2	1.4	1.9	0.5	37.6		1.21
18pt3	1.9	5.1	3.2	43.2	5.6	2.58
18pt4	5.1	5.8	0.7	40.0	-3.2	1.37
18pt5	5.8	7.2	1.4	42.8	2.9	1.59
18pt6	7.2	7.5	0.3	38.4	-4.5	0.83
18pt7	7.5	8.3	0.8	43.8	5.4	0.98
18pt8	8.3	9	0.7	39.4	-4.3	0.96
18pt9	9	9.7	0.7	43.0	3.6	0.98
18pt10	9.7	12	2.3	39.4	-3.6	1.16
18pt11	12	12.5	0.5	36.6	-2.8	0.61
18pt12	12.5	12.9	0.4	39.2	2.5	1.02
18pt13	12.9	13.9	1	42.3	3.1	1.8
18pt14	13.9	14.7	0.8	38.2	-4.0	0.73
18pt15	14.7	15.6	0.9	36.3	-1.9	0.91
18pt16	15.6	15.8	0.2	37.6	1.4	0
18pt17	15.8	16.5	0.7	42.4	4.7	0.93
18pt18	16.5	17.2	0.7	39.2	-3.1	1.46
18pt19	17.2	17.5	0.3	42.5	3.2	0.69
18pt20	17.5	17.7	0.2	38.4	-4.0	0
18pt21	17.7	18.2	0.5	43.4	5.0	1.38

18pt22	18.2	18.8	0.6	38.7	-4.7	1.21
18pt23	18.8	19.1	0.3	46.5	7.8	2.36
18pt24	19.1	20	0.9	42.8	-3.7	1.17
18pt25	20	20.2	0.2	40.7	-2.1	0
18pt26	20.2	20.9	0.7	41.3	0.6	0.4
18pt27	20.9	22.3	1.4	38.9	-2.4	1.2
18pt28	22.3	22.6	0.3	42.0	3.1	0.65
18pt29	22.6	23.8	1.2	38.1	-4.0	1.01
18pt30	23.8	24	0.2	36.3	-1.7	0
18pt31	24	24.4	0.4	37.4	1.1	0.94
18pt32	24.4	27.3	2.9	35.2	-2.2	0.98
18pt33	27.3	27.8	0.5	39.6	4.4	1.21
18pt34	27.8	28	0.2	42.5	2.9	0
18pt35	28	28.8	0.8	39.0	-3.5	0.94
18pt36	28.8	30.5	1.7	35.7	-3.3	0.91
18pt37	30.5	31.7	1.2	39.2	3.5	1.64
18pt38	31.7	32.8	1.1	42.2	3.0	1.18
18pt39	32.8	33.2	0.4	37.4	-4.8	0.72
18pt40	33.2	33.6	0.4	50.6	13.3	3.1
18pt41	33.6	33.8	0.2	43.1	-7.6	0
18pt42	33.8	34.1	0.3	40.0	-3.1	0.64
18pt43	34.1	34.3	0.2	36.1	-3.9	0
18pt44	34.3	35	0.7	37.3	1.2	0.59
18pt45	35	35.4	0.4	36.4	-0.9	0.77
18pt46	35.4	35.9	0.5	37.4	1.0	0.51
18pt47	35.9	37.6	1.7	35.9	-1.5	1
18pt48	37.6	38	0.4	38.4	2.6	0.65
18pt49	38	38.2	0.2	36.3	-2.2	0
18pt50	38.2	38.6	0.4	38.4	2.2	0.19
18pt51	38.6	40.6	2	36.1	-2.3	0.74
18pt52	40.6	40.9	0.3	38.7	2.5	1.65
18pt53	40.9	41.2	0.3	42.2	3.6	0.58
18pt54	41.2	41.5	0.3	39.9	-2.4	1.24
18pt55	41.5	42.5	1	43.0	3.2	1.48
18pt56	42.5	43	0.5	46.9	3.9	1.66
18pt57	43	43.4	0.4	40.8	-6.1	1.78
18pt58	43.4	44.2	0.8	42.8	2.0	2.63
18pt59	44.2	44.6	0.4	46.8	4.0	2.84
18pt60	44.6	44.8	0.2	44.9	-1.9	0
18pt61	44.8	45.3	0.5	50.0	5.1	0.69
18pt62	45.3	45.7	0.4	38.0	-12.0	1.21
18pt63	45.7	47.6	1.9	43.3	5.3	1.89
18pt64	47.6	49.2	1.6	38.5	-4.8	1.22
18pt65	49.2	50.1	0.9	36.3	-2.2	0.5
18pt66	50.1	53.1	3	38.5	2.2	1.11
18pt67	53.1	53.4	0.3	35.8	-2.7	0.75
18pt68	53.4	56.4	3	43.0	7.2	1.32
18pt69	56.4	56.7	0.3	38.4	-4.5	2.17
18pt70	56.7	57.6	0.9	35.8	-2.6	0.59
18pt71	57.6	58.2	0.6	39.1	3.3	1.29
18pt72	58.2	58.4	0.2	42.2	3.1	0
18pt73	58.4	58.8	0.4	37.0	-5.2	2.56
18pt74	58.8	59.2	0.4	43.1	6.1	0.67
18pt75	59.2	59.5	0.3	40.1	-3.0	0.82
18pt76	59.5	59.9	0.4	42.6	2.5	1.36
18pt77	59.9	61	1.1	38.7	-4.0	1.08

18pt78	61	66.6	5.6	35.1	-3.6	1.03
18pt79	66.6	67.8	1.2	38.8	3.7	1.77
18pt80	67.8	69.3	1.5	35.4	-3.4	0.78
18pt81	69.3	69.9	0.6	37.6	2.2	0.75
18pt82	69.9	70.5	0.6	36.0	-1.6	0.72
18pt83	70.5	70.7	0.2	39.5	3.6	0
18pt84	70.7	71.5	0.8	43.2	3.7	2.22
18pt85	71.5	71.9	0.4	37.0	-6.2	0.99
18pt86	71.9	72.5	0.6	43.6	6.6	2.3
18pt87	72.5	73	0.5	40.5	-3.1	1.24
18pt88	73	73.9	0.9	43.9	3.4	1.68
18pt89	73.9	74.1	0.2	48.0	4.1	0
18pt90	74.1	74.3	0.2	42.3	-5.7	0
18pt91	74.3	74.9	0.6	40.6	-1.7	1.19
18pt92	74.9	75.4	0.5	42.6	1.9	1.68
18pt93	75.4	76	0.6	47.9	5.3	1.01
18pt94	76	76.3	0.3	41.4	-6.5	1.21
18pt95	76.3	76.6	0.3	54.3	12.9	1.38
18pt96	76.6	77	0.4	50.3	-4.0	2.05
18pt97	77	77.3	0.3	45.6	-4.7	1.77
19pt1	0	0.3	0.3	48.9		0
19pt2	0.3	2.5	2.2	58.1	9.3	1.84
19pt3	2.5	2.8	0.3	53.1	-5.0	1.66
19pt4	2.8	3	0.2	50.2	-2.9	0
19pt5	3	4.8	1.8	54.9	4.7	1.74
19pt6	4.8	5.1	0.3	51.7	-3.2	0.84
19pt7	5.1	5.4	0.3	55.4	3.6	1.94
19pt8	5.4	5.9	0.5	51.2	-4.2	1
19pt9	5.9	6.1	0.2	54.1	2.9	0
19pt10	6.1	7	0.9	49.5	-4.6	3.04
19pt11	7	7.3	0.3	44.0	-5.6	1.72
19pt12	7.3	7.7	0.4	49.2	5.2	2.52
19pt13	7.7	8.2	0.5	53.5	4.3	2.57
19pt14	8.2	8.9	0.7	51.7	-1.8	0.85
19pt15	8.9	10	1.1	44.3	-7.4	1.7
19pt16	10	10.5	0.5	50.6	6.4	1.3
19pt17	10.5	10.7	0.2	54.0	3.4	0
19pt18	10.7	11.9	1.2	52.0	-2.0	0.7
19pt19	11.9	13	1.1	44.3	-7.8	1.25
19pt20	13	13.6	0.6	53.6	9.4	1.08
19pt21	13.6	14.2	0.6	47.4	-6.2	2.11
19pt22	14.2	14.6	0.4	53.3	5.9	2.27
19pt23	14.6	15	0.4	51.7	-1.7	1.75
19pt24	15	15.5	0.5	44.1	-7.5	1.69
19pt25	15.5	17.6	2.1	49.5	5.3	2.93
19pt26	17.6	17.8	0.2	55.0	5.5	0
19pt27	17.8	18.2	0.4	51.7	-3.3	0.53
19pt28	18.2	19.4	1.2	54.1	2.5	1.03
19pt29	19.4	20.1	0.7	52.4	-1.8	1.38
19pt30	20.1	20.3	0.2	48.6	-3.8	0
19pt31	20.3	20.6	0.3	41.3	-7.3	1.3
19pt32	20.6	21.1	0.5	40.0	-1.3	0.81
19pt33	21.1	21.3	0.2	41.5	1.5	0
19pt34	21.3	22.8	1.5	40.5	-1.0	1.62
19pt35	22.8	24.2	1.4	41.4	0.9	1.5

19pt36	24.2	32.4	8.2	47.7	6.3	0
19pt37	32.4	32.6	0.2	37.8	-9.8	0
19pt38	32.6	33	0.4	37.6	-0.2	1.91
19pt39	33	34.7	1.7	43.3	5.6	2.06
19pt40	34.7	36.1	1.4	47.2	4.0	3.34
19pt41	36.1	36.6	0.5	42.8	-4.4	2
19pt42	36.6	36.8	0.2	46.4	3.7	0
19pt43	36.8	38.4	1.6	43.8	-2.6	2.07
19pt44	38.4	39.3	0.9	50.4	6.6	2.47
19pt45	39.3	39.8	0.5	43.7	-6.8	1.62
19pt46	39.8	40	0.2	48.3	4.6	0
19pt47	40	40.5	0.5	42.6	-5.8	1.38
19pt48	40.5	41.8	1.3	51.0	8.5	2.57
19pt49	41.8	43.4	1.6	41.8	-9.3	1.19
19pt50	43.4	45.1	1.7	51.1	9.3	2.16
19pt51	45.1	45.7	0.6	43.7	-7.4	4.44
19pt52	45.7	46.5	0.8	50.8	7.1	1.64
19pt53	46.5	46.7	0.2	44.4	-6.4	0
19pt54	46.7	47.1	0.4	49.5	5.2	2.64
19pt55	47.1	47.3	0.2	45.3	-4.2	0
19pt56	47.3	47.5	0.2	53.2	7.8	0
19pt57	47.5	47.7	0.2	51.5	-1.7	0
19pt58	47.7	47.9	0.2	55.4	3.9	0
19pt59	47.9	48.9	1	43.4	-12.0	1.35
19pt60	48.9	49.4	0.5	49.4	6.0	1.44
19pt61	49.4	50	0.6	39.6	-9.8	1.07
19pt62	50	50.2	0.2	43.5	3.9	0
19pt63	50.2	50.5	0.3	51.2	7.6	2.11
19pt64	50.5	53.8	3.3	50.4	-0.8	3.03
19pt65	53.8	54	0.2	44.5	-5.8	0
19pt66	54	54.7	0.7	52.7	8.2	1.47
19pt67	54.7	54.9	0.2	54.2	1.5	0
19pt68	54.9	55.1	0.2	47.5	-6.7	0
19pt69	55.1	55.7	0.6	53.5	6.0	0.94
19pt70	55.7	57.4	1.7	49.5	-4.0	2.96
19pt71	57.4	59.2	1.8	43.9	-5.7	1.48
19pt72	59.2	60.4	1.2	49.0	5.1	2.72
19pt73	60.4	60.6	0.2	45.4	-3.6	0
19pt74	60.6	60.8	0.2	50.3	4.8	0
19pt75	60.8	61.4	0.6	54.7	4.5	1.58
19pt76	61.4	63.8	2.4	44.5	-10.3	2.75
19pt77	63.8	64.5	0.7	49.5	5.0	3.22
20pt1	0	0.2	0.2	38.2		0
20pt2	0.2	1	0.8	48.0	9.8	2.36
20pt3	1	1.7	0.7	43.8	-4.2	1.9
20pt4	1.7	2.1	0.4	46.8	3.0	3.15
20pt5	2.1	2.5	0.4	44.2	-2.6	1.24
20pt6	2.5	2.7	0.2	47.4	3.2	0
20pt7	2.7	2.9	0.2	43.2	-4.2	0
20pt8	2.9	3.2	0.3	50.0	6.8	0.96
20pt9	3.2	3.5	0.3	41.5	-8.5	1.9
20pt10	3.5	3.8	0.3	50.8	9.2	2.54
20pt11	3.8	4	0.2	43.1	-7.7	0
20pt12	4	4.2	0.2	47.7	4.6	0
20pt13	4.2	6.1	1.9	43.2	-4.5	1.79

20pt14	6.1	6.9	0.8	39.1	-4.1	1.12
20pt15	6.9	7.7	0.8	36.6	-2.5	0.32
20pt16	7.7	14.4	6.7	39.0	2.4	1.48
20pt17	14.4	14.8	0.4	36.5	-2.5	0.48
20pt18	14.8	16.7	1.9	39.1	2.6	1.45
20pt19	16.7	16.9	0.2	42.3	3.2	0
20pt20	16.9	17.5	0.6	39.4	-2.9	0.49
20pt21	17.5	17.8	0.3	42.9	3.6	1.83
20pt22	17.8	18.3	0.5	47.5	4.5	2.43
20pt23	18.3	20.7	2.4	42.6	-4.9	1.56
20pt24	20.7	20.9	0.2	39.2	-3.4	0
20pt25	20.9	22.1	1.2	42.1	2.9	1.93
20pt26	22.1	22.5	0.4	38.8	-3.4	1.16
20pt27	22.5	23.3	0.8	42.1	3.4	1.37
20pt28	23.3	23.5	0.2	47.1	5.0	0
20pt29	23.5	24.9	1.4	44.2	-2.9	1.42
20pt30	24.9	26.3	1.4	46.2	2.0	3.16
20pt31	26.3	27.9	1.6	0.0		0
20pt32	27.9	28.3	0.4	40.4		1.04
20pt33	28.3	29.2	0.9	47.4	7.1	2.04
20pt34	29.2	29.4	0.2	43.5	-3.9	0
20pt35	29.4	29.6	0.2	53.8	10.3	0
20pt36	29.6	31	1.4	47.6	-6.2	2.12
20pt37	31	32.5	1.5	44.8	-2.8	3.09
20pt38	32.5	32.8	0.3	47.5	2.7	1.69
20pt39	32.8	33.2	0.4	44.3	-3.2	1.29
20pt40	33.2	34.1	0.9	49.0	4.7	1.82
20pt41	34.1	34.4	0.3	43.7	-5.3	0.78
20pt42	34.4	35.6	1.2	46.8	3.1	3.37
20pt43	35.6	35.8	0.2	43.2	-3.6	0
20pt44	35.8	36.3	0.5	46.5	3.4	1.75
20pt45	36.3	36.5	0.2	44.5	-2.1	0
20pt46	36.5	37.7	1.2	39.7	-4.8	0.95
20pt47	37.7	39.7	2	43.4	3.7	2.66
20pt48	39.7	39.9	0.2	40.3	-3.1	0
20pt49	39.9	40.8	0.9	43.6	3.3	1.31
20pt50	40.8	42.1	1.3	46.9	3.3	1.55
20pt51	42.1	42.9	0.8	43.6	-3.3	3.38
20pt52	42.9	43.1	0.2	40.6	-3.0	0
20pt53	43.1	43.8	0.7	49.2	8.6	2.22
20pt54	43.8	44.5	0.7	44.2	-5.0	2.29
20pt55	44.5	44.9	0.4	46.7	2.5	0.79
20pt56	44.9	45.1	0.2	42.4	-4.3	0
20pt57	45.1	46.3	1.2	48.3	5.9	1.84
20pt58	46.3	46.7	0.4	44.1	-4.2	1.63
20pt59	46.7	47.1	0.4	47.3	3.2	0.99
20pt60	47.1	47.3	0.2	44.4	-2.9	0
20pt61	47.3	48.6	1.3	48.5	4.1	2.11
20pt62	48.6	48.8	0.2	45.3	-3.2	0
20pt63	48.8	49.4	0.6	47.3	2.0	0.78
20pt64	49.4	49.8	0.4	43.4	-3.9	0.78
20pt65	49.8	50.5	0.7	40.1	-3.4	0.64
20pt66	50.5	51.8	1.3	42.6	2.5	1.33
20pt67	51.8	53.7	1.9	38.4	-4.2	1.31
20pt68	53.7	54.2	0.5	43.2	4.9	1.34
20pt69	54.2	55.9	1.7	48.4	5.2	1.34

20pt70	55.9	56.2	0.3	43.2	-5.2	0.55
20pt71	56.2	56.6	0.4	48.2	4.9	0.51
20pt72	56.6	57	0.4	44.2	-4.0	1.06
20pt73	57	58.1	1.1	45.0	0.8	3.88
20pt74	58.1	58.8	0.7	42.6	-2.3	1
20pt75	58.8	59.4	0.6	48.7	6.1	2.35
20pt76	59.4	62.3	2.9	55.7	7.0	3.35
21pt1	0	13.6	13.6	0.0		0
21pt2	13.6	14.2	0.6	41.8		4.04
21pt3	14.2	14.9	0.7	36.4	-5.4	0.47
21pt4	14.9	15.7	0.8	37.7	1.3	0.74
21pt5	15.7	17.4	1.7	36.1	-1.7	1.01
21pt6	17.4	18.2	0.8	39.7	3.6	1.27
21pt7	18.2	25.2	7	35.1	-4.6	0.96
21pt8	25.2	26.9	1.7	38.8	3.6	1.24
21pt9	26.9	28.2	1.3	36.2	-2.6	0.5
21pt10	28.2	29.7	1.5	39.1	2.9	1.31
21pt11	29.7	29.9	0.2	36.6	-2.4	0
21pt12	29.9	30.2	0.3	37.2	0.6	0.11
21pt13	30.2	30.5	0.3	36.1	-1.2	0.37
21pt14	30.5	30.8	0.3	38.9	2.8	1.66
21pt15	30.8	32.7	1.9	43.6	4.6	2.2
21pt16	32.7	32.9	0.2	46.4	2.8	0
21pt17	32.9	33.3	0.4	44.1	-2.3	1.39
21pt18	33.3	33.5	0.2	40.4	-3.7	0
21pt19	33.5	34.8	1.3	43.8	3.4	1.33
21pt20	34.8	35.4	0.6	40.3	-3.4	0.5
21pt21	35.4	37.8	2.4	43.7	3.4	2.55
21pt22	37.8	38	0.2	40.6	-3.1	0
21pt23	38	40	2	43.0	2.4	1.9
21pt24	40	40.3	0.3	39.3	-3.7	0.6
21pt25	40.3	40.8	0.5	41.6	2.3	0.68
21pt26	40.8	42.9	2.1	49.0	7.4	1.94
21pt27	42.9	43.1	0.2	56.7	7.7	0
21pt28	43.1	43.8	0.7	50.4	-6.3	3.53
21pt29	43.8	44.2	0.4	55.2	4.8	2.48
21pt30	44.2	44.5	0.3	49.7	-5.5	1.01
21pt31	44.5	44.7	0.2	55.8	6.2	0
21pt32	44.7	44.9	0.2	46.0	-9.8	0
21pt33	44.9	45.2	0.3	57.0	11.0	2.02
21pt34	45.2	45.6	0.4	48.1	-8.8	5.23
21pt35	45.6	45.9	0.3	54.2	6.1	1.71
21pt36	45.9	46.3	0.4	48.3	-5.9	2.76
21pt37	46.3	46.5	0.2	41.0	-7.3	0
22pt1	0	14.4	14.4	0.0		0
22pt2	14.4	14.6	0.2	44.6		0
22pt3	14.6	14.8	0.2	39.4	-5.1	0
22pt4	14.8	15.2	0.4	0.0	-39.4	0
22pt5	15.2	15.8	0.6	39.6	39.6	2.08
22pt6	15.8	16.2	0.4	47.5	7.9	2.01
22pt7	16.2	16.6	0.4	44.7	-2.8	1.84
22pt8	16.6	18.2	1.6	48.8	4.1	2.86
22pt9	18.2	19	0.8	55.7	6.9	2.75
22pt10	19	20.4	1.4	49.9	-5.8	3.77

22pt11	20.4	20.9	0.5	45.5	-4.4	4.32
22pt12	20.9	21.1	0.2	0.0		0
22pt13	21.1	24.2	3.1	49.2		4.49
22pt14	24.2	25.2	1	44.1	-5.1	2.05
22pt15	25.2	25.5	0.3	47.6	3.4	1
22pt16	25.5	26	0.5	45.3	-2.3	0.4
22pt17	26	26.9	0.9	47.0	1.7	3.45
22pt18	26.9	27.5	0.6	38.5	-8.5	1.11
22pt19	27.5	28	0.5	44.9	6.3	0.93
22pt20	28	28.7	0.7	49.6	4.7	1.88
22pt21	28.7	29.1	0.4	42.0	-7.6	2.32
22pt22	29.1	30.3	1.2	49.0	7.0	2.81
22pt23	30.3	33.1	2.8	44.4	-4.6	1.94
22pt24	33.1	33.7	0.6	40.3	-4.1	0.71
22pt25	33.7	34.2	0.5	44.5	4.2	1.46
22pt26	34.2	34.7	0.5	48.9	4.4	3.18
22pt27	34.7	34.9	0.2	38.8	-10.0	0
22pt28	34.9	35.1	0.2	44.5	5.7	0
22pt29	35.1	35.9	0.8	49.2	4.7	1.65
22pt30	35.9	36.7	0.8	54.1	4.9	1.5
22pt31	36.7	37.4	0.7	51.8	-2.3	1.88
22pt32	37.4	37.6	0.2	44.8	-7.0	0
22pt33	37.6	38.2	0.6	50.5	5.7	1.94
22pt34	38.2	38.7	0.5	54.0	3.5	1.89
22pt35	38.7	38.9	0.2	39.6	-14.4	0
22pt36	38.9	41.9	3	47.8	8.2	3.94
22pt37	41.9	42.2	0.3	48.7	1.0	2.55
22pt38	42.2	42.4	0.2	55.7	7.0	0
22pt39	42.4	42.7	0.3	49.8	-5.9	2.49
22pt40	42.7	42.9	0.2	42.1	-7.8	0
22pt41	42.9	45.1	2.2	49.7	7.7	3.62
22pt42	45.1	45.3	0.2	56.7	7.0	0
22pt43	45.3	45.5	0.2	50.3	-6.4	0
22pt44	45.5	45.9	0.4	54.4	4.2	1.67
22pt45	45.9	46.9	1	48.3	-6.1	2.49
22pt46	46.9	47.3	0.4	43.9	-4.4	1.06
22pt47	47.3	47.7	0.4	48.8	4.9	0.92
22pt48	47.7	48	0.3	55.5	6.7	1.34
22pt49	48	49.2	1.2	50.6	-4.9	1.76
22pt50	49.2	50.2	1	55.4	4.8	4.6
Xpt1	0	0.6	0.6	49.6		2.47
Xpt2	0.6	0.8	0.2	43.4	-6.2	0
Xpt3	0.8	1.3	0.5	47.2	3.8	1.79
Xpt4	1.3	1.5	0.2	54.5	7.3	0
Xpt5	1.5	1.7	0.2	48.6	-5.9	0
Xpt6	1.7	3.8	2.1	43.9	-4.7	2.3
Xpt7	3.8	4.8	1	37.9	-6.0	0.86
Xpt8	4.8	5.1	0.3	36.6	-1.4	0.36
Xpt9	5.1	6.7	1.6	38.7	2.2	0.79
Xpt10	6.7	7	0.3	42.0	3.3	1.47
Xpt11	7	9.1	2.1	39.2	-2.8	0.98
Xpt12	9.1	9.6	0.5	44.2	5.0	1.45
Xpt13	9.6	10.2	0.6	45.9	1.8	1.83
Xpt14	10.2	12.7	2.5	39.1	-6.8	0.83
Xpt15	12.7	13.1	0.4	41.4	2.3	0.19

Xpt16	13.1	13.6	0.5	40.6	-0.8	0.6
Xpt17	13.6	14	0.4	41.3	0.7	0.41
Xpt18	14	16.5	2.5	38.8	-2.5	1.39
Xpt19	16.5	16.9	0.4	42.6	3.9	1.18
Xpt20	16.9	17.1	0.2	40.3	-2.4	0
Xpt21	17.1	18.5	1.4	41.9	1.6	1.58
Xpt22	18.5	18.7	0.2	39.6	-2.3	0
Xpt23	18.7	20.2	1.5	43.0	3.4	1.75
Xpt24	20.2	21.3	1.1	38.4	-4.5	1.19
Xpt25	21.3	21.7	0.4	35.8	-2.6	0.92
Xpt26	21.7	22	0.3	39.3	3.5	0.83
Xpt27	22	22.2	0.2	41.5	2.2	0
Xpt28	22.2	22.8	0.6	38.6	-2.9	1.25
Xpt29	22.8	23	0.2	35.6	-3.0	0
Xpt30	23	23.7	0.7	38.8	3.2	1.42
Xpt31	23.7	24.8	1.1	42.4	3.6	1.1
Xpt32	24.8	25.9	1.1	38.6	-3.8	1.86
Xpt33	25.9	28.6	2.7	36.3	-2.3	1.29
Xpt34	28.6	28.8	0.2	37.8	1.6	0
Xpt35	28.8	30.1	1.3	35.9	-1.9	0.67
Xpt36	30.1	31.7	1.6	38.9	3.0	1.15
Xpt37	31.7	35.2	3.5	36.0	-2.9	0.92
Xpt38	35.2	35.4	0.2	37.4	1.4	0
Xpt39	35.4	37.2	1.8	36.4	-1.1	1
Xpt40	37.2	39.3	2.1	39.2	2.8	1.19
Xpt41	39.3	39.6	0.3	42.4	3.2	0.47
Xpt42	39.6	40.5	0.9	48.0	5.6	2.44
Xpt43	40.5	41.3	0.8	43.3	-4.7	2.58
Xpt44	41.3	41.5	0.2	39.9	-3.4	0
Xpt45	41.5	41.8	0.3	45.4	5.6	1.14
Xpt46	41.8	44.8	3	38.9	-6.5	1.16
Xpt47	44.8	45.2	0.4	42.3	3.5	0.35
Xpt48	45.2	46.7	1.5	39.0	-3.4	1.53
Xpt49	46.7	47.2	0.5	41.9	2.9	1.15
Xpt50	47.2	47.4	0.2	40.2	-1.7	0
Xpt51	47.4	48.1	0.7	45.2	5.1	2.53
Xpt52	48.1	48.3	0.2	40.1	-5.1	0
Xpt53	48.3	48.6	0.3	43.9	3.8	0.56
Xpt54	48.6	49.5	0.9	48.6	4.6	1.79
Xpt55	49.5	49.7	0.2	42.1	-6.4	0
Xpt56	49.7	52.7	3	39.8	-2.4	1.18
Xpt57	52.7	52.9	0.2	0.0		0
Xpt58	52.9	53.6	0.7	43.8		1.87
Xpt59	53.6	53.9	0.3	46.7	2.9	0.34
Xpt60	53.9	54.6	0.7	42.1	-4.6	2
Xpt61	54.6	54.8	0.2	40.3	-1.8	0
Xpt62	54.8	55	0.2	47.0	6.7	0
Xpt63	55	58.6	3.6	39.2	-7.8	1.37
Xpt64	58.6	58.8	0.2	44.0	4.8	0
Xpt65	58.8	59.1	0.3	37.9	-6.1	0.66
Xpt66	59.1	62	2.9	0.0		0
Xpt67	62	64.7	2.7	38.8		1.32
Xpt68	64.7	65.1	0.4	43.5	4.7	2.27
Xpt69	65.1	68	2.9	38.7	-4.9	1.3
Xpt70	68	68.7	0.7	47.7	9.1	1.18
Xpt71	68.7	68.9	0.2	42.4	-5.3	0

Xpt72	68.9	69.3	0.4	38.3	-4.1	0.91
Xpt73	69.3	69.9	0.6	42.8	4.5	1.79
Xpt74	69.9	70.4	0.5	39.5	-3.3	1.69
Xpt75	70.4	70.6	0.2	48.1	8.5	0
Xpt76	70.6	71.7	1.1	44.6	-3.5	1.56
Xpt77	71.7	72.1	0.4	38.6	-5.9	1.46
Xpt78	72.1	72.4	0.3	42.3	3.7	0
Xpt79	72.4	73.6	1.2	38.9	-3.4	1.34
Xpt80	73.6	73.9	0.3	42.4	3.5	0.53
Xpt81	73.9	78.4	4.5	38.7	-3.7	1.35
Xpt82	78.4	79.2	0.8	36.5	-2.2	0.41
Xpt83	79.2	79.9	0.7	37.3	0.8	0.75
Xpt84	79.9	80.1	0.2	36.1	-1.2	0
Xpt85	80.1	80.4	0.3	37.9	1.8	0.44
Xpt86	80.4	81	0.6	36.1	-1.8	0.75
Xpt87	81	81.2	0.2	37.1	1.0	0
Xpt88	81.2	84.9	3.7	36.1	-1.0	0.88
Xpt89	84.9	85.6	0.7	37.4	1.3	0.89
Xpt90	85.6	93.4	7.8	36.0	-1.5	0.8
Xpt91	93.4	93.6	0.2	37.7	1.8	0
Xpt92	93.6	95.7	2.1	35.9	-1.8	0.78
Xpt93	95.7	96.2	0.5	38.1	2.2	0.98
Xpt94	96.2	96.7	0.5	36.0	-2.0	0.95
Xpt95	96.7	97.4	0.7	38.1	2.1	0.7
Xpt96	97.4	97.8	0.4	36.4	-1.8	0.68
Xpt97	97.8	98	0.2	37.4	1.1	0
Xpt98	98	99.3	1.3	36.1	-1.4	0.73
Xpt99	99.3	100.5	1.2	38.8	2.7	1.67
Xpt100	100.5	101	0.5	42.3	3.5	0.77
Xpt101	101	101.8	0.8	39.2	-3.1	1.02
Xpt102	101.8	102	0.2	44.5	5.3	0
Xpt103	102	104.8	2.8	39.9	-4.6	1.8
Xpt104	104.8	105.5	0.7	36.4	-3.5	0.92
Xpt105	105.5	106.8	1.3	37.9	1.5	1.88
Xpt106	106.8	107.3	0.5	43.9	6.0	2.31
Xpt107	107.3	108	0.7	39.9	-4.0	1.49
Xpt108	108	108.7	0.7	36.4	-3.5	0.85
Xpt109	108.7	109.3	0.6	39.3	2.9	0.82
Xpt110	109.3	109.7	0.4	41.4	2.0	0.75
Xpt111	109.7	112.6	2.9	39.1	-2.2	1.39
Xpt112	112.6	113.3	0.7	36.7	-2.5	0.65
Xpt113	113.3	113.7	0.4	37.9	1.2	0.46
Xpt114	113.7	114.5	0.8	35.3	-2.5	0.85
Xpt115	114.5	115.5	1	39.7	4.3	2.79
Xpt116	115.5	116.7	1.2	36.5	-3.2	0.48
Xpt117	116.7	117.3	0.6	37.9	1.4	0.61
Xpt118	117.3	117.5	0.2	36.6	-1.4	0
Xpt119	117.5	118.2	0.7	39.9	3.4	1.65
Xpt120	118.2	119.1	0.9	43.0	3.0	1.41
Xpt121	119.1	119.3	0.2	46.7	3.7	0
Xpt122	119.3	119.7	0.4	42.7	-4.0	1.35
Xpt123	119.7	120.8	1.1	39.9	-2.8	1.17
Xpt124	120.8	122.3	1.5	36.5	-3.4	0.53
Xpt125	122.3	123.1	0.8	38.6	2.1	1.09
Xpt126	123.1	124	0.9	39.9	1.3	2.33
Xpt127	124	124.6	0.6	36.5	-3.4	0.38

Xpt128	124.6	124.9	0.3	37.7	1.2	0.78
Xpt129	124.9	127.8	2.9	35.6	-2.0	0.69
Xpt130	127.8	129	1.2	38.3	2.7	1.06
Xpt131	129	129.4	0.4	45.6	7.3	0.4
Xpt132	129.4	129.6	0.2	48.5	2.9	0
Xpt133	129.6	129.9	0.3	40.9	-7.6	1.05
Xpt134	129.9	132.6	2.7	39.4	-1.4	1.29
Xpt135	132.6	133	0.4	42.7	3.3	1.22
Xpt136	133	134	1	39.6	-3.2	0.76
Xpt137	134	136.5	2.5	41.3	1.7	1.07
Xpt138	136.5	136.8	0.3	38.9	-2.4	0.42
Xpt139	136.8	137	0.2	41.6	2.7	0
Xpt140	137	137.9	0.9	38.6	-3.0	0.98
Xpt141	137.9	138.1	0.2	36.5	-2.1	0
Xpt142	138.1	141.8	3.7	38.8	2.3	1.46
Xpt143	141.8	142.9	1.1	36.7	-2.2	0.45
Xpt144	142.9	143.2	0.3	38.7	2.0	0.92
Xpt145	143.2	144.4	1.2	36.2	-2.5	0.84
Xpt146	144.4	145.9	1.5	37.9	1.6	1.04
Xpt147	145.9	146.6	0.7	36.4	-1.5	0.68
Xpt148	146.6	146.9	0.3	37.8	1.4	0.7
Xpt149	146.9	147.1	0.2	36.5	-1.3	0
Xpt150	147.1	148.9	1.8	38.8	2.3	0.88
Xpt151	148.9	149.3	0.4	44.6	5.8	2.33
Xpt152	149.3	149.7	0.4	39.2	-5.4	1.29
Xpt153	149.7	150.6	0.9	43.9	4.7	2.03
Xpt154	150.6	151.2	0.6	40.2	-3.8	0.91
Xpt155	151.2	151.5	0.3	43.6	3.4	2.48
Xpt156	151.5	152.1	0.6	38.4	-5.2	1.31
Xpt157	152.1	152.3	0.2	43.3	4.9	0
Xpt158	152.3	152.5	0.2	48.3	5.1	0
Xpt159	152.5	152.7	0.2	42.9	-5.4	0
Xpt160	152.7	152.9	0.2	49.4	6.5	0
Xpt161	152.9	153.4	0.5	55.6	6.2	2.32
Xpt162	153.4	153.6	0.2	48.7	-6.9	0
Xpt163	153.6	153.9	0.3	54.5	5.8	1.37
Xpt164	153.9	154.1	0.2	45.1	-9.4	0
Xpt165	154.1	155.4	1.3	38.6	-6.4	2.33
Ypt1	0	0.9	0.9	40.1		2.01
Ypt2	0.9	1.4	0.5	44.5	4.4	1.28
Ypt3	1.4	4.1	2.7	39.0	-5.5	1.59
Ypt4	4.1	5.1	1	43.1	4.0	2.09
Ypt5	5.1	5.7	0.6	38.4	-4.7	1.34
Ypt6	5.7	5.9	0.2	42.6	4.2	0
Ypt7	5.9	6.1	0.2	37.1	-5.5	0
Ypt8	6.1	7.1	1	51.1	14.0	0
Ypt9	7.1	7.3	0.2	37.3	-13.8	0
Ypt10	7.3	7.7	0.4	46.5	9.2	1.91
Ypt11	7.7	9.8	2.1	39.9	-6.6	1.7
Ypt12	9.8	10.4	0.6	42.2	2.3	2.76
Ypt13	10.4	11.7	1.3	38.6	-3.6	1.42
Ypt14	11.7	11.9	0.2	42.1	3.5	0
Ypt15	11.9	12.8	0.9	38.2	-3.9	1.23
Ypt16	12.8	14.4	1.6	42.3	4.1	1.97
Ypt17	14.4	15.3	0.9	38.9	-3.4	1.43

Ypt18	15.3	15.6	0.3	36.2	-2.7	0.71
Ypt19	15.6	16.3	0.7	38.4	2.3	0.85
Ypt20	16.3	16.5	0.2	41.6	3.2	0
Ypt21	16.5	17.4	0.9	38.6	-3.1	1.25
Ypt22	17.4	17.7	0.3	44.1	5.5	0.89
Ypt23	17.7	22.7	5	39.0	-5.1	1.45
Ypt24	22.7	23.3	0.6	43.3	4.3	0.98
Ypt25	23.3	23.8	0.5	39.5	-3.8	1.23
Ypt26	23.8	24	0.2	45.0	5.5	0
