# Supplementary material for: The evolution of isochore patterns in vertebrate genomes
Source: BMC Genomics. 2009 Apr 3;10:146. doi: 10.1186/1471-2164-10-146 (PMC2678159; doi:10.1186/1471-2164-10-146)
Supplement: Additional File 5 — Overview of dog chromosomes. The color-coded maps show the compositional patterns of dog chromosomes. [file 1471-2164-10-146-S5.pdf]

Additional Table T2. Coordinates, sizes, GC levels and GC standard deviations (SD) of the dog isochores. ΔGC indicates the difference in GC between subsequent isochores.

Isochore	Start	End	Length, Mb	GC, %	Δ GC	SD (w=100kb)
1cf1	0	3	3	0.0		0
1cf2	3	3.2	0.2	46.0		0
1cf3	3.2	3.4	0.2	38.9	-7.1	0
1cf4	3.4	3.8	0.4	46.6	7.6	3.34
1cf5	3.8	4.2	0.4	56.1	9.5	2.05
1cf6	4.2	4.4	0.2	41.8	-14.2	0
1cf7	4.4	4.9	0.5	49.9	8.1	1.75
1cf8	4.9	5.8	0.9	44.0	-5.9	1.34
1cf9	5.8	6	0.2	51.0	7.0	0
1cf10	6	6.4	0.4	46.5	-4.6	3.04
1cf11	6.4	7.1	0.7	42.0	-4.5	1.47
1cf12	7.1	7.5	0.4	46.5	4.4	1.68
1cf13	7.5	7.8	0.3	38.0	-8.4	1.11
1cf14	7.8	8.5	0.7	43.2	5.2	2.88
1cf15	8.5	8.7	0.2	38.8	-4.4	0
1cf16	8.7	9	0.3	35.9	-3.0	0.44
1cf17	9	9.4	0.4	38.9	3.0	0.92
1cf18	9.4	10.6	1.2	35.7	-3.1	0.83
1cf19	10.6	10.9	0.3	38.6	2.9	1.09
1cf20	10.9	11.1	0.2	41.3	2.7	0
1cf21	11.1	11.7	0.6	38.2	-3.1	2.14
1cf22	11.7	13.2	1.5	35.4	-2.8	0.59
1cf23	13.2	13.4	0.2	37.3	1.9	0
1cf24	13.4	15.8	2.4	34.9	-2.5	0.73
1cf25	15.8	16.7	0.9	39.4	4.6	1.24
1cf26	16.7	17.1	0.4	43.8	4.4	1.16
1cf27	17.1	17.3	0.2	39.0	-4.8	0
1cf28	17.3	17.6	0.3	44.1	5.0	0.93
1cf29	17.6	17.8	0.2	34.8	-9.2	0
1cf30	17.8	18.2	0.4	43.1	8.3	1.8
1cf31	18.2	18.7	0.5	38.9	-4.3	1.44
1cf32	18.7	19.3	0.6	36.4	-2.5	0.59
1cf33	19.3	19.9	0.6	42.3	5.9	2.29
1cf34	19.9	20.1	0.2	46.6	4.4	0
1cf35	20.1	20.5	0.4	44.4	-2.2	1.11
1cf36	20.5	20.7	0.2	46.9	2.5	0
1cf37	20.7	21.8	1.1	44.6	-2.3	1.86
1cf38	21.8	23.4	1.6	39.1	-5.5	1.76
1cf39	23.4	23.6	0.2	42.7	3.6	0
1cf40	23.6	24.7	1.1	39.0	-3.7	1.46
1cf41	24.7	25.3	0.6	36.8	-2.2	0.87
1cf42	25.3	26.5	1.2	39.2	2.4	1.29
1cf43	26.5	27.2	0.7	44.2	4.9	2.93
1cf44	27.2	27.5	0.3	39.7	-4.5	0.27
1cf45	27.5	28	0.5	46.6	7.0	1.95
1cf46	28	29.2	1.2	38.2	-8.4	1.2
1cf47	29.2	29.8	0.6	36.8	-1.4	0.47
1cf48	29.8	31.1	1.3	39.1	2.3	1.27
1cf49	31.1	31.6	0.5	36.1	-3.0	0.78
1cf50	31.6	34.8	3.2	39.4	3.3	0.92
1cf51	34.8	37.3	2.5	35.0	-4.4	1.15

1cf52	37.3	38.9	1.6	39.0	4.0	1.05
1cf53	38.9	39.2	0.3	36.3	-2.7	0.53
1cf54	39.2	39.7	0.5	37.7	1.3	0.38
1cf55	39.7	40.6	0.9	36.7	-1.0	0.75
1cf56	40.6	41.2	0.6	37.1	0.5	0.68
1cf57	41.2	41.5	0.3	35.1	-2.1	1.29
1cf58	41.5	42.4	0.9	39.2	4.1	1.24
1cf59	42.4	42.6	0.2	41.6	2.4	0
1cf60	42.6	43.6	1	40.0	-1.6	1.18
1cf61	43.6	44	0.4	42.8	2.8	0.84
1cf62	44	44.5	0.5	40.8	-2.0	0.34
1cf63	44.5	44.8	0.3	42.4	1.6	1.1
1cf64	44.8	45.6	0.8	38.5	-3.9	1.26
1cf65	45.6	46.1	0.5	36.3	-2.2	1.03
1cf66	46.1	46.4	0.3	37.3	1.0	0.3
1cf67	46.4	47.1	0.7	35.3	-2.0	0.46
1cf68	47.1	47.7	0.6	38.4	3.1	2.22
1cf69	47.7	48.1	0.4	41.8	3.4	0.51
1cf70	48.1	48.3	0.2	38.1	-3.7	0
1cf71	48.3	48.7	0.4	36.5	-1.6	0.54
1cf72	48.7	49.8	1.1	39.4	2.9	1.08
1cf73	49.8	50.6	0.8	44.5	5.1	3.2
1cf74	50.6	50.9	0.3	39.8	-4.7	0.6
1cf75	50.9	52.1	1.2	43.1	3.3	1.65
1cf76	52.1	52.4	0.3	46.9	3.7	1
1cf77	52.4	53.3	0.9	42.2	-4.6	2.27
1cf78	53.3	54.1	0.8	38.5	-3.7	1.21
1cf79	54.1	54.4	0.3	36.6	-1.9	0.34
1cf80	54.4	54.7	0.3	38.2	1.7	1.41
1cf81	54.7	55.6	0.9	41.1	2.9	2.9
1cf82	55.6	56.1	0.5	36.7	-4.4	0.81
1cf83	56.1	56.7	0.6	38.6	1.9	1.87
1cf84	56.7	57.3	0.6	44.3	5.7	1.56
1cf85	57.3	58.4	1.1	48.4	4.1	1.65
1cf86	58.4	58.6	0.2	44.1	-4.3	0
1cf87	58.6	59.6	1	49.0	4.9	4.51
1cf88	59.6	59.8	0.2	44.3	-4.7	0
1cf89	59.8	61.5	1.7	38.8	-5.6	1.42
1cf90	61.5	61.7	0.2	35.7	-3.1	0
1cf91	61.7	62.5	0.8	38.7	3.0	1.5
1cf92	62.5	64.9	2.4	35.4	-3.3	1.07
1cf93	64.9	65.3	0.4	38.2	2.8	1.06
1cf94	65.3	65.9	0.6	35.5	-2.7	0.6
1cf95	65.9	66.3	0.4	37.4	1.9	0.79
1cf96	66.3	66.6	0.3	35.4	-2.1	0.82
1cf97	66.6	67.2	0.6	38.6	3.3	1.16
1cf98	67.2	67.9	0.7	42.2	3.6	1.83
1cf99	67.9	68.9	1	37.1	-5.1	1.98
1cf100	68.9	71.1	2.2	38.9	1.8	1.66
1cf101	71.1	72	0.9	44.5	5.6	1.78
1cf102	72	72.2	0.2	48.3	3.8	0
1cf103	72.2	72.7	0.5	43.5	-4.7	1.89
1cf104	72.7	73.6	0.9	39.5	-4.0	0.65
1cf105	73.6	74.4	0.8	42.1	2.6	3.47
1cf106	74.4	74.7	0.3	37.9	-4.3	0.53
1cf107	74.7	74.9	0.2	44.0	6.1	0

1cf108	74.9	75.1	0.2	40.0	-4.1	0
1cf109	75.1	75.7	0.6	43.7	3.7	2.07
1cf110	75.7	76	0.3	40.2	-3.6	0.5
1cf111	76	76.4	0.4	42.1	2.0	0.66
1cf112	76.4	76.9	0.5	40.0	-2.2	2.31
1cf113	76.9	77.4	0.5	39.5	-0.5	2.55
1cf114	77.4	77.9	0.5	41.5	2.1	0.51
1cf115	77.9	79.9	2	39.5	-2.1	1.52
1cf116	79.9	80.2	0.3	36.5	-2.9	0.28
1cf117	80.2	80.9	0.7	39.4	2.9	2.29
1cf118	80.9	81.5	0.6	36.5	-2.9	0.81
1cf119	81.5	83.3	1.8	38.7	2.2	1.27
1cf120	83.3	83.5	0.2	43.3	4.6	0
1cf121	83.5	84.3	0.8	39.6	-3.8	0.79
1cf122	84.3	84.5	0.2	34.6	-4.9	0
1cf123	84.5	86.7	2.2	39.7	5.1	1.11
1cf124	86.7	88	1.3	35.6	-4.1	0.87
1cf125	88	90	2	39.0	3.3	1.19
1cf126	90	90.2	0.2	44.6	5.6	0
1cf127	90.2	90.4	0.2	38.4	-6.2	0
1cf128	90.4	90.6	0.2	42.0	3.6	0
1cf129	90.6	90.9	0.3	38.5	-3.5	0.72
1cf130	90.9	92	1.1	42.6	4.1	2.12
1cf131	92	92.3	0.3	40.5	-2.1	0.56
1cf132	92.3	92.6	0.3	43.4	2.9	1.03
1cf133	92.6	92.9	0.3	46.7	3.3	0.49
1cf134	92.9	93.2	0.3	41.2	-5.5	1.33
1cf135	93.2	93.7	0.5	39.7	-1.5	1.11
1cf136	93.7	94.1	0.4	43.4	3.7	1.2
1cf137	94.1	94.3	0.2	39.9	-3.5	0
1cf138	94.3	94.6	0.3	42.3	2.4	0.74
1cf139	94.6	95.2	0.6	38.5	-3.8	1.8
1cf140	95.2	95.5	0.3	42.5	4.0	1.98
1cf141	95.5	96	0.5	40.8	-1.7	0.16
1cf142	96	96.3	0.3	42.9	2.1	0.83
1cf143	96.3	97.7	1.4	39.4	-3.6	1.89
1cf144	97.7	98.2	0.5	42.6	3.2	1.86
1cf145	98.2	98.4	0.2	49.7	7.1	0
1cf146	98.4	99.1	0.7	45.4	-4.2	4.41
1cf147	99.1	99.5	0.4	43.6	-1.8	2.39
1cf148	99.5	100	0.5	53.6	10.0	3.3
1cf149	100	100.7	0.7	49.0	-4.7	1.59
1cf150	100.7	101	0.3	43.6	-5.4	1.39
1cf151	101	101.5	0.5	50.5	6.9	4.66
1cf152	101.5	101.9	0.4	53.8	3.2	4.85
1cf153	101.9	102.4	0.5	41.4	-12.4	2.46
1cf154	102.4	102.7	0.3	48.4	7.1	0.78
1cf155	102.7	105.3	2.6	44.3	-4.1	3.22
1cf156	105.3	105.6	0.3	54.4	10.1	0.65
1cf157	105.6	105.9	0.3	44.5	-9.9	2.23
1cf158	105.9	106.6	0.7	48.3	3.7	3.28
1cf159	106.6	107.1	0.5	40.4	-7.9	0.84
1cf160	107.1	107.3	0.2	41.2	0.8	0
1cf161	107.3	107.7	0.4	40.7	-0.4	0.14
1cf162	107.7	108	0.3	42.8	2.0	0.73
1cf163	108	108.2	0.2	40.8	-1.9	0

1cf164	108.2	108.4	0.2	45.6	4.8	0
1cf165	108.4	110.5	2.1	50.0	4.4	2.89
1cf166	110.5	110.7	0.2	53.8	3.8	0
1cf167	110.7	111	0.3	47.2	-6.6	4.88
1cf168	111	113.6	2.6	50.5	3.3	2.92
1cf169	113.6	113.8	0.2	44.8	-5.7	0
1cf170	113.8	114.2	0.4	39.4	-5.5	1.44
1cf171	114.2	114.9	0.7	47.1	7.7	2.49
1cf172	114.9	115.1	0.2	55.9	8.8	0
1cf173	115.1	117.2	2.1	50.3	-5.6	3.37
1cf174	117.2	117.6	0.4	53.1	2.8	1.66
1cf175	117.6	118	0.4	47.6	-5.4	4.46
1cf176	118	118.3	0.3	39.3	-8.4	0.53
1cf177	118.3	118.6	0.3	36.6	-2.7	0.49
1cf178	118.6	119.4	0.8	38.3	1.7	0.89
1cf179	119.4	120.5	1.1	50.3	12.1	3.7
1cf180	120.5	121	0.5	43.7	-6.6	2.73
1cf181	121	121.2	0.2	40.1	-3.6	0
1cf182	121.2	121.5	0.3	50.2	10.1	3.09
1cf183	121.5	121.9	0.4	54.0	3.8	2.94
1cf184	121.9	122.8	0.9	47.2	-6.8	3.15
1cf185	122.8	123	0.2	44.4	-2.8	0
1cf186	123	123.9	0.9	49.2	4.8	3.18
1cf187	123.9	124.2	0.3	55.2	6.0	0.44
1cf188	124.2	124.6	0.4	48.5	-6.7	3.16
1cf189	124.6	124.9	0.3	58.8	10.3	1.11
1cf190	124.9	125.4	0.5	48.4	-10.4	2.65
1cf191	125.4	125.7	0.3	41.0	-7.4	0
2cf1	0	3	3	0.0		0
2cf2	3	4	1	36.0		0.68
2cf3	4	5.4	1.4	39.4	3.3	2.2
2cf4	5.4	5.6	0.2	36.5	-2.9	0
2cf5	5.6	6.5	0.9	39.8	3.3	1.03
2cf6	6.5	6.7	0.2	41.9	2.1	0
2cf7	6.7	10.9	4.2	38.6	-3.3	1.32
2cf8	10.9	11.2	0.3	34.9	-3.7	1.03
2cf9	11.2	11.8	0.6	39.0	4.1	1.2
2cf10	11.8	12.1	0.3	43.0	3.9	0.97
2cf11	12.1	12.9	0.8	38.9	-4.0	1.22
2cf12	12.9	13.1	0.2	42.6	3.7	0
2cf13	13.1	13.4	0.3	38.9	-3.7	1.46
2cf14	13.4	13.6	0.2	42.4	3.5	0
2cf15	13.6	14	0.4	38.0	-4.4	0.96
2cf16	14	14.2	0.2	35.0	-3.0	0
2cf17	14.2	14.5	0.3	37.9	2.9	1.53
2cf18	14.5	14.8	0.3	43.0	5.1	0.76
2cf19	14.8	15.5	0.7	38.9	-4.1	0.84
2cf20	15.5	17	1.5	36.0	-2.9	0.82
2cf21	17	18	1	38.4	2.4	1.51
2cf22	18	18.2	0.2	43.8	5.3	0
2cf23	18.2	18.4	0.2	34.3	-9.4	0
2cf24	18.4	18.8	0.4	39.8	5.5	2.43
2cf25	18.8	20	1.2	42.4	2.6	2.43
2cf26	20	20.2	0.2	40.6	-1.8	0
2cf27	20.2	20.4	0.2	42.3	1.8	0

2cf28	20.4	23.3	2.9	38.8	-3.5	1.41
2cf29	23.3	23.8	0.5	35.5	-3.3	1.01
2cf30	23.8	24.2	0.4	39.0	3.4	0.96
2cf31	24.2	24.6	0.4	43.7	4.7	1.82
2cf32	24.6	24.8	0.2	39.4	-4.3	0
2cf33	24.8	25	0.2	41.9	2.5	0
2cf34	25	25.2	0.2	40.5	-1.4	0
2cf35	25.2	27	1.8	43.5	2.9	2.73
2cf36	27	27.3	0.3	38.9	-4.6	2.09
2cf37	27.3	28	0.7	44.9	6.0	3.99
2cf38	28	28.3	0.3	43.0	-1.9	0.67
2cf39	28.3	28.6	0.3	40.1	-2.9	0.63
2cf40	28.6	30.4	1.8	36.1	-4.0	0.89
2cf41	30.4	30.8	0.4	40.0	4.0	1
2cf42	30.8	31	0.2	45.8	5.7	0
2cf43	31	31.2	0.2	39.4	-6.4	0
2cf44	31.2	32.1	0.9	43.5	4.1	2.05
2cf45	32.1	32.5	0.4	39.6	-3.9	1.1
2cf46	32.5	32.7	0.2	47.7	8.1	0
2cf47	32.7	32.9	0.2	42.1	-5.6	0
2cf48	32.9	33.3	0.4	47.9	5.8	5.62
2cf49	33.3	33.6	0.3	43.7	-4.1	2.72
2cf50	33.6	34	0.4	39.0	-4.7	1.22
2cf51	34	34.7	0.7	43.5	4.5	1.73
2cf52	34.7	35	0.3	48.1	4.5	1.87
2cf53	35	35.2	0.2	54.3	6.2	0
2cf54	35.2	36.3	1.1	43.5	-10.8	2.06
2cf55	36.3	36.7	0.4	54.5	11.0	5.52
2cf56	36.7	37.1	0.4	49.0	-5.5	1.78
2cf57	37.1	37.3	0.2	53.7	4.7	0
2cf58	37.3	37.7	0.4	44.7	-9.0	1.37
2cf59	37.7	38	0.3	51.2	6.5	1.38
2cf60	38	38.9	0.9	42.2	-9.0	3.66
2cf61	38.9	39.1	0.2	40.0	-2.2	0
2cf62	39.1	39.4	0.3	42.4	2.4	1.6
2cf63	39.4	39.7	0.3	49.2	6.9	2.71
2cf64	39.7	40.6	0.9	43.7	-5.6	1.48
2cf65	40.6	40.8	0.2	40.1	-3.6	0
2cf66	40.8	41.2	0.4	40.9	0.8	1.15
2cf67	41.2	42.2	1	39.3	-1.6	1.11
2cf68	42.2	42.8	0.6	36.7	-2.6	0.44
2cf69	42.8	43.2	0.4	39.4	2.7	1.08
2cf70	43.2	43.4	0.2	42.7	3.3	0
2cf71	43.4	43.6	0.2	37.9	-4.8	0
2cf72	43.6	44	0.4	41.8	3.8	1.93
2cf73	44	44.5	0.5	40.3	-1.5	0.5
2cf74	44.5	44.7	0.2	43.3	3.0	0
2cf75	44.7	50.6	5.9	38.7	-4.6	1.42
2cf76	50.6	51.5	0.9	42.0	3.3	2.53
2cf77	51.5	52.3	0.8	38.3	-3.7	2.9
2cf78	52.3	52.7	0.4	36.1	-2.1	1.1
2cf79	52.7	53.3	0.6	38.1	2.0	0.93
2cf80	53.3	53.5	0.2	41.7	3.6	0
2cf81	53.5	54.3	0.8	36.7	-5.1	2.74
2cf82	54.3	54.7	0.4	38.5	1.8	2.2
2cf83	54.7	55.6	0.9	42.7	4.2	1.35

2cf84	55.6	56	0.4	38.8	-3.9	1.61
2cf85	56	57.1	1.1	42.5	3.7	1.49
2cf86	57.1	57.3	0.2	40.6	-1.9	0
2cf87	57.3	58.3	1	43.1	2.5	2.42
2cf88	58.3	58.6	0.3	46.4	3.3	2.12
2cf89	58.6	58.9	0.3	38.9	-7.5	0.97
2cf90	58.9	59.2	0.3	49.6	10.7	3.13
2cf91	59.2	59.6	0.4	45.1	-4.5	1.69
2cf92	59.6	60.1	0.5	50.9	5.8	2.16
2cf93	60.1	60.6	0.5	41.0	-9.9	1.99
2cf94	60.6	61.3	0.7	42.7	1.7	2.6
2cf95	61.3	61.6	0.3	50.2	7.5	2.07
2cf96	61.6	61.8	0.2	54.3	4.1	0
2cf97	61.8	62.4	0.6	48.3	-6.0	3.65
2cf98	62.4	63.1	0.7	43.7	-4.6	3.02
2cf99	63.1	63.3	0.2	47.3	3.6	0
2cf100	63.3	63.5	0.2	45.1	-2.2	0
2cf101	63.5	63.8	0.3	47.4	2.3	1.91
2cf102	63.8	64.3	0.5	44.1	-3.3	1.94
2cf103	64.3	64.6	0.3	48.8	4.7	0.98
2cf104	64.6	64.8	0.2	41.5	-7.3	0
2cf105	64.8	65.2	0.4	39.3	-2.2	2.04
2cf106	65.2	65.4	0.2	36.0	-3.4	0
2cf107	65.4	65.7	0.3	41.2	5.2	1.74
2cf108	65.7	65.9	0.2	41.6	0.4	0
2cf109	65.9	66.4	0.5	40.1	-1.5	1.02
2cf110	66.4	67.1	0.7	42.5	2.4	1
2cf111	67.1	68	0.9	47.8	5.3	3.13
2cf112	68	68.3	0.3	42.7	-5.1	2.11
2cf113	68.3	68.8	0.5	51.2	8.5	2.07
2cf114	68.8	69.9	1.1	42.2	-9.0	1.76
2cf115	69.9	70.2	0.3	38.9	-3.3	1.44
2cf116	70.2	70.7	0.5	35.8	-3.0	0.46
2cf117	70.7	71	0.3	43.5	7.6	1.51
2cf118	71	71.5	0.5	47.8	4.3	1.23
2cf119	71.5	71.7	0.2	42.1	-5.7	0
2cf120	71.7	71.9	0.2	48.7	6.6	0
2cf121	71.9	72.2	0.3	43.2	-5.5	1.65
2cf122	72.2	72.5	0.3	52.9	9.7	2.9
2cf123	72.5	72.9	0.4	43.7	-9.2	3.23
2cf124	72.9	73.6	0.7	52.5	8.8	1.91
2cf125	73.6	74.3	0.7	50.8	-1.7	1.12
2cf126	74.3	75.3	1	41.9	-8.9	3.14
2cf127	75.3	76.2	0.9	48.1	6.2	2.99
2cf128	76.2	76.4	0.2	43.5	-4.6	0
2cf129	76.4	77.3	0.9	48.6	5.1	1.74
2cf130	77.3	77.5	0.2	43.3	-5.2	0
2cf131	77.5	77.8	0.3	51.1	7.8	4.18
2cf132	77.8	78.1	0.3	43.2	-8.0	1.1
2cf133	78.1	78.4	0.3	48.7	5.6	2.52
2cf134	78.4	78.7	0.3	45.4	-3.4	0.8
2cf135	78.7	78.9	0.2	50.2	4.8	0
2cf136	78.9	79.3	0.4	43.0	-7.2	1.06
2cf137	79.3	79.7	0.4	53.7	10.7	0.77
2cf138	79.7	80.2	0.5	49.4	-4.3	3.64
2cf139	80.2	80.5	0.3	50.7	1.3	6.94

2cf140	80.5	80.7	0.2	49.6	-1.1	0
2cf141	80.7	81.1	0.4	37.8	-11.8	1.87
2cf142	81.1	82.5	1.4	49.7	11.9	2.94
2cf143	82.5	84.8	2.3	54.0	4.3	4.45
2cf144	84.8	85.2	0.4	49.7	-4.3	3.3
2cf145	85.2	85.7	0.5	54.5	4.8	2.78
2cf146	85.7	86	0.3	45.6	-8.9	0.3
2cf147	86	86.5	0.5	48.2	2.6	3.12
2cf148	86.5	88.2	1.7	55.4	7.1	5.94
2cf149	88.2	88.5	0.3	47.9	-7.4	0

3cf1	0	3	3	0.0		0
3cf2	3	3.4	0.4	44.3		4.08
3cf3	3.4	3.6	0.2	49.3	5.0	0
3cf4	3.6	4.1	0.5	43.8	-5.5	2.72
3cf5	4.1	4.6	0.5	38.7	-5.2	1.06
3cf6	4.6	4.9	0.3	38.0	-0.6	1.85
3cf7	4.9	5.4	0.5	43.5	5.5	1.93
3cf8	5.4	5.8	0.4	40.7	-2.8	3.6
3cf9	5.8	6.1	0.3	36.5	-4.2	0.63
3cf10	6.1	6.9	0.8	38.2	1.7	2.66
3cf11	6.9	7.5	0.6	43.7	5.5	2.11
3cf12	7.5	10.6	3.1	34.9	-8.7	1.21
3cf13	10.6	11.4	0.8	39.2	4.3	2.17
3cf14	11.4	14	2.6	35.4	-3.9	0.97
3cf15	14	14.6	0.6	37.8	2.4	0.96
3cf16	14.6	15.4	0.8	35.4	-2.4	0.91
3cf17	15.4	16.2	0.8	39.3	3.9	1.5
3cf18	16.2	16.4	0.2	36.7	-2.6	0
3cf19	16.4	17.7	1.3	38.3	1.6	2.19
3cf20	17.7	18.4	0.7	35.7	-2.7	1.39
3cf21	18.4	19	0.6	38.5	2.9	1.62
3cf22	19	20.5	1.5	35.1	-3.4	0.97
3cf23	20.5	20.9	0.4	38.8	3.7	1.25
3cf24	20.9	26.7	5.8	35.7	-3.1	1.5
3cf25	26.7	27.2	0.5	39.0	3.3	0.62
3cf26	27.2	27.5	0.3	35.1	-3.9	1.5
3cf27	27.5	28.5	1	38.3	3.2	1.17
3cf28	28.5	28.8	0.3	34.6	-3.7	1.19
3cf29	28.8	29.7	0.9	40.0	5.4	2.3
3cf30	29.7	30.1	0.4	43.1	3.1	0.71
3cf31	30.1	30.8	0.7	39.2	-4.0	2.4
3cf32	30.8	31.2	0.4	43.5	4.3	1.47
3cf33	31.2	31.6	0.4	35.7	-7.8	1.99
3cf34	31.6	32.6	1	40.0	4.3	1.94
3cf35	32.6	32.8	0.2	44.5	4.5	0
3cf36	32.8	33	0.2	40.2	-4.3	0
3cf37	33	33.3	0.3	43.7	3.5	1.8
3cf38	33.3	39	5.7	38.8	-5.0	1.74
3cf39	39	39.2	0.2	36.8	-2.0	0
3cf40	39.2	40.3	1.1	39.5	2.7	1.16
3cf41	40.3	40.6	0.3	46.5	7.0	3.45
3cf42	40.6	40.8	0.2	42.7	-3.8	0
3cf43	40.8	41	0.2	39.8	-2.9	0
3cf44	41	41.4	0.4	45.2	5.4	2.81
3cf45	41.4	42.3	0.9	39.8	-5.4	1.49

3cf46	42.3	43.2	0.9	44.6	4.8	1.96
3cf47	43.2	43.4	0.2	40.0	-4.6	0
3cf48	43.4	43.9	0.5	43.5	3.5	1.57
3cf49	43.9	44.2	0.3	40.1	-3.4	5.89
3cf50	44.2	44.4	0.2	37.5	-2.5	0
3cf51	44.4	45.4	1	43.2	5.6	1.62
3cf52	45.4	46.9	1.5	38.3	-4.9	1.52
3cf53	46.9	47.1	0.2	43.3	5.0	0
3cf54	47.1	49.6	2.5	38.3	-5.0	1.14
3cf55	49.6	50.6	1	44.2	5.9	2.94
3cf56	50.6	50.8	0.2	37.8	-6.3	0
3cf57	50.8	51.1	0.3	46.5	8.7	0.48
3cf58	51.1	51.6	0.5	40.2	-6.3	1.1
3cf59	51.6	51.8	0.2	41.5	1.3	0
3cf60	51.8	52.1	0.3	40.3	-1.2	0.06
3cf61	52.1	52.5	0.4	44.9	4.6	2.04
3cf62	52.5	53.8	1.3	38.8	-6.1	1.04
3cf63	53.8	54.2	0.4	43.5	4.7	2.03
3cf64	54.2	54.4	0.2	49.3	5.9	0
3cf65	54.4	54.6	0.2	45.2	-4.1	0
3cf66	54.6	55	0.4	47.1	1.9	1.03
3cf67	55	55.2	0.2	43.3	-3.8	0
3cf68	55.2	56.3	1.1	47.9	4.6	3.46
3cf69	56.3	56.9	0.6	43.2	-4.6	2.42
3cf70	56.9	57.1	0.2	47.6	4.4	0
3cf71	57.1	58.3	1.2	43.5	-4.1	1.95
3cf72	58.3	58.5	0.2	39.4	-4.1	0
3cf73	58.5	59	0.5	44.1	4.7	3.68
3cf74	59	59.2	0.2	40.7	-3.4	0
3cf75	59.2	60.9	1.7	49.1	8.4	3.13
3cf76	60.9	61.4	0.5	53.9	4.8	1.36
3cf77	61.4	61.9	0.5	48.7	-5.2	2.37
3cf78	61.9	62.5	0.6	54.8	6.0	1.97
3cf79	62.5	62.7	0.2	49.9	-4.8	0
3cf80	62.7	63.8	1.1	55.6	5.7	2.15
3cf81	63.8	64.4	0.6	47.1	-8.5	2.94
3cf82	64.4	64.9	0.5	47.0	-0.2	5.32
3cf83	64.9	65.5	0.6	54.4	7.4	4.39
3cf84	65.5	65.9	0.4	43.7	-10.7	1.69
3cf85	65.9	67	1.1	40.0	-3.6	0.64
3cf86	67	67.3	0.3	43.2	3.2	2.32
3cf87	67.3	67.8	0.5	40.5	-2.6	1.37
3cf88	67.8	68.3	0.5	39.8	-0.7	2.25
3cf89	68.3	69.2	0.9	38.8	-1.0	1.2
3cf90	69.2	69.7	0.5	41.4	2.6	3.01
3cf91	69.7	70.2	0.5	38.7	-2.7	1.35
3cf92	70.2	70.6	0.4	36.8	-2.0	0.2
3cf93	70.6	71.8	1.2	39.5	2.8	1.88
3cf94	71.8	72.1	0.3	42.3	2.8	0.8
3cf95	72.1	73.2	1.1	47.7	5.4	2.49
3cf96	73.2	73.5	0.3	41.3	-6.4	1.42
3cf97	73.5	74.3	0.8	48.0	6.7	3.02
3cf98	74.3	74.5	0.2	39.7	-8.3	0
3cf99	74.5	75.1	0.6	41.6	1.9	1.28
3cf100	75.1	76.6	1.5	38.4	-3.2	1.89
3cf101	76.6	77.5	0.9	43.6	5.1	1.77

3cf102	77.5	77.8	0.3	39.4	-4.2	1.01
3cf103	77.8	83.1	5.3	34.6	-4.8	0.99
3cf104	83.1	83.3	0.2	37.8	3.1	0
3cf105	83.3	86	2.7	34.8	-3.0	1.05
3cf106	86	86.3	0.3	39.5	4.7	1
3cf107	86.3	86.6	0.3	42.8	3.3	5.45
3cf108	86.6	87	0.4	40.3	-2.5	4.69
3cf109	87	87.2	0.2	43.4	3.1	0
3cf110	87.2	87.7	0.5	47.6	4.2	2.09
3cf111	87.7	87.9	0.2	40.5	-7.2	0
3cf112	87.9	88.2	0.3	47.1	6.7	1.7
3cf113	88.2	89	0.8	43.1	-4.1	1.18
3cf114	89	89.2	0.2	39.9	-3.1	0
3cf115	89.2	89.6	0.4	36.2	-3.7	0.4
3cf116	89.6	90.1	0.5	39.0	2.8	0.94
3cf117	90.1	90.4	0.3	42.8	3.8	1.8
3cf118	90.4	91.7	1.3	39.3	-3.5	0.82
3cf119	91.7	92.2	0.5	42.9	3.6	2.23
3cf120	92.2	93.3	1.1	38.4	-4.5	1.08
3cf121	93.3	94.2	0.9	42.5	4.1	3.25
3cf122	94.2	94.8	0.6	59.5	17.0	4.88
4cf1	0	3	3	0.0		0
4cf2	3	4.3	1.3	39.4		1.82
4cf3	4.3	4.7	0.4	35.6	-3.8	0.95
4cf4	4.7	5.7	1	37.8	2.2	1.4
4cf5	5.7	6	0.3	37.1	-0.7	1.83
4cf6	6	6.4	0.4	41.9	4.8	0.35
4cf7	6.4	6.6	0.2	40.2	-1.6	0
4cf8	6.6	7.1	0.5	42.7	2.4	1.7
4cf9	7.1	7.3	0.2	37.5	-5.2	0
4cf10	7.3	7.8	0.5	39.8	2.3	2.65
4cf11	7.8	8.2	0.4	46.5	6.6	0.68
4cf12	8.2	8.9	0.7	42.9	-3.6	1.86
4cf13	8.9	9.6	0.7	40.4	-2.5	1.11
4cf14	9.6	9.8	0.2	44.0	3.6	0
4cf15	9.8	10	0.2	39.4	-4.6	0
4cf16	10	10.9	0.9	42.3	2.8	1.28
4cf17	10.9	11.1	0.2	38.7	-3.5	0
4cf18	11.1	11.5	0.4	42.7	4.0	1.6
4cf19	11.5	11.7	0.2	47.3	4.6	0
4cf20	11.7	11.9	0.2	42.1	-5.2	0
4cf21	11.9	12.1	0.2	48.1	6.0	0
4cf22	12.1	13.6	1.5	42.7	-5.4	2.3
4cf23	13.6	13.9	0.3	36.3	-6.4	0.36
4cf24	13.9	14.5	0.6	37.5	1.3	0.17
4cf25	14.5	14.9	0.4	36.2	-1.3	1.57
4cf26	14.9	15.1	0.2	38.1	1.9	0
4cf27	15.1	15.3	0.2	41.5	3.3	0
4cf28	15.3	16.5	1.2	38.9	-2.6	0.95
4cf29	16.5	17	0.5	36.0	-2.9	0.88
4cf30	17	18.2	1.2	40.2	4.2	1.88
4cf31	18.2	18.5	0.3	36.2	-4.0	0.24
4cf32	18.5	18.7	0.2	38.6	2.4	0
4cf33	18.7	19	0.3	41.7	3.0	0.73
4cf34	19	22.2	3.2	35.0	-6.7	1.07

4cf35	22.2	22.4	0.2	39.0	4.0	0
4cf36	22.4	22.6	0.2	35.5	-3.5	0
4cf37	22.6	23.4	0.8	39.5	4.0	1.56
4cf38	23.4	23.7	0.3	42.5	3.1	2.34
4cf39	23.7	24.2	0.5	51.4	8.9	3.07
4cf40	24.2	24.4	0.2	44.8	-6.6	0
4cf41	24.4	25.5	1.1	51.6	6.7	2.46
4cf42	25.5	25.8	0.3	54.2	2.7	1.16
4cf43	25.8	26.2	0.4	47.6	-6.6	5.95
4cf44	26.2	27.3	1.1	37.4	-10.2	1.17
4cf45	27.3	27.6	0.3	45.3	7.9	3.89
4cf46	27.6	27.8	0.2	39.0	-6.3	0
4cf47	27.8	28.2	0.4	34.4	-4.7	0.55
4cf48	28.2	28.5	0.3	39.0	4.7	1.52
4cf49	28.5	28.8	0.3	45.9	6.9	2.67
4cf50	28.8	29.5	0.7	42.7	-3.2	1.6
4cf51	29.5	30.1	0.6	39.2	-3.5	0.75
4cf52	30.1	30.5	0.4	43.7	4.4	1.58
4cf53	30.5	31.9	1.4	49.8	6.1	2.51
4cf54	31.9	32.4	0.5	57.1	7.3	2.21
4cf55	32.4	33	0.6	49.0	-8.0	3.69
4cf56	33	33.2	0.2	38.8	-10.2	0
4cf57	33.2	33.6	0.4	35.0	-3.8	0.87
4cf58	33.6	35.4	1.8	38.5	3.5	1.19
4cf59	35.4	36.9	1.5	43.8	5.2	3.74
4cf60	36.9	37.1	0.2	47.1	3.3	0
4cf61	37.1	37.6	0.5	52.7	5.6	8.1
4cf62	37.6	37.9	0.3	45.3	-7.4	1.6
4cf63	37.9	38.3	0.4	54.9	9.6	1.7
4cf64	38.3	38.6	0.3	45.1	-9.8	4.1
4cf65	38.6	38.8	0.2	42.1	-3.0	0
4cf66	38.8	39	0.2	40.4	-1.7	0
4cf67	39	39.2	0.2	52.4	12.1	0
4cf68	39.2	39.7	0.5	42.4	-10.1	3.76
4cf69	39.7	40	0.3	50.6	8.2	0.93
4cf70	40	40.3	0.3	41.5	-9.0	3.05
4cf71	40.3	40.6	0.3	46.3	4.8	2.71
4cf72	40.6	41.7	1.1	44.8	-1.5	1.37
4cf73	41.7	41.9	0.2	39.7	-5.0	0
4cf74	41.9	42.1	0.2	47.0	7.3	0
4cf75	42.1	42.6	0.5	43.9	-3.1	3.05
4cf76	42.6	43.1	0.5	47.8	3.9	1.06
4cf77	43.1	44.1	1	43.7	-4.1	3.01
4cf78	44.1	44.4	0.3	36.4	-7.3	3.1
4cf79	44.4	45.1	0.7	45.7	9.2	2.09
4cf80	45.1	45.7	0.6	39.1	-6.5	1.65
4cf81	45.7	47	1.3	43.7	4.5	2.38
4cf82	47	47.3	0.3	39.3	-4.4	1.9
4cf83	47.3	50.8	3.5	35.1	-4.3	1.16
4cf84	50.8	51.1	0.3	38.3	3.2	1.28
4cf85	51.1	53	1.9	35.5	-2.8	0.53
4cf86	53	53.4	0.4	39.1	3.6	1.25
4cf87	53.4	54.4	1	43.1	4.0	1.65
4cf88	54.4	55.9	1.5	39.3	-3.8	1.09
4cf89	55.9	56.1	0.2	45.0	5.7	0
4cf90	56.1	58.1	2	38.8	-6.2	1.1

4cf91	58.1	58.5	0.4	45.7	6.9	2.66
4cf92	58.5	58.9	0.4	44.1	-1.7	1.14
4cf93	58.9	59.8	0.9	39.3	-4.8	1.55
4cf94	59.8	60	0.2	35.6	-3.6	0
4cf95	60	60.5	0.5	39.0	3.4	1.17
4cf96	60.5	60.8	0.3	41.7	2.7	0.97
4cf97	60.8	61	0.2	46.5	4.8	0
4cf98	61	61.2	0.2	42.9	-3.6	0
4cf99	61.2	61.4	0.2	48.8	5.9	0
4cf100	61.4	61.7	0.3	41.2	-7.6	1.18
4cf101	61.7	62	0.3	51.6	10.3	0.99
4cf102	62	62.2	0.2	54.0	2.5	0
4cf103	62.2	63	0.8	46.7	-7.3	3.73
4cf104	63	63.3	0.3	44.6	-2.0	1.08
4cf105	63.3	63.8	0.5	39.4	-5.2	1.67
4cf106	63.8	64.2	0.4	41.5	2.1	0.18
4cf107	64.2	65.5	1.3	38.4	-3.1	1.48
4cf108	65.5	69.4	3.9	35.6	-2.7	1.18
4cf109	69.4	69.8	0.4	39.1	3.5	1.32
4cf110	69.8	70.1	0.3	43.5	4.4	0.92
4cf111	70.1	70.5	0.4	40.0	-3.6	0.35
4cf112	70.5	70.9	0.4	36.2	-3.7	0.24
4cf113	70.9	72.1	1.2	38.5	2.3	0.8
4cf114	72.1	72.6	0.5	35.9	-2.6	0.52
4cf115	72.6	73	0.4	38.8	2.8	1.01
4cf116	73	73.2	0.2	34.9	-3.9	0
4cf117	73.2	73.9	0.7	42.7	7.8	0.72
4cf118	73.9	74.3	0.4	41.2	-1.5	3.45
4cf119	74.3	74.5	0.2	36.5	-4.7	0
4cf120	74.5	74.8	0.3	37.9	1.4	0.31
4cf121	74.8	75	0.2	34.1	-3.8	0
4cf122	75	76.4	1.4	39.5	5.4	1.16
4cf123	76.4	77.4	1	42.5	3.0	0.96
4cf124	77.4	78	0.6	37.1	-5.5	1.55
4cf125	78	78.9	0.9	42.8	5.7	1.67
4cf126	78.9	79.4	0.5	38.8	-3.9	1.07
4cf127	79.4	88.9	9.5	35.1	-3.8	1.12
4cf128	88.9	89.4	0.5	38.1	3.1	1.49
4cf129	89.4	89.6	0.2	43.7	5.5	0
4cf130	89.6	89.8	0.2	48.2	4.6	0
4cf131	89.8	90.2	0.4	52.1	3.9	1.35
4cf132	90.2	90.6	0.4	43.7	-8.5	1.54
4cf133	90.6	91	0.4	48.6	4.9	3.89
4cf134	91	91.5	0.5	44.5	-4.1	2.47
5cf1	0	3	3	0.0		0
5cf2	3	3.2	0.2	39.8		0
5cf3	3.2	3.6	0.4	43.2	3.4	1.1
5cf4	3.6	3.8	0.2	57.3	14.1	0
5cf5	3.8	4.2	0.4	44.0	-13.3	0.95
5cf6	4.2	4.4	0.2	55.6	11.6	0
5cf7	4.4	4.8	0.4	43.8	-11.8	2.04
5cf8	4.8	5.1	0.3	46.7	2.9	0.93
5cf9	5.1	5.4	0.3	44.0	-2.7	1.33
5cf10	5.4	5.6	0.2	40.6	-3.3	0
5cf11	5.6	6.3	0.7	43.8	3.2	1.82

5cf12	6.3	6.6	0.3	48.6	4.7	0.27
5cf13	6.6	7.4	0.8	42.8	-5.8	2.21
5cf14	7.4	7.7	0.3	46.2	3.4	0.77
5cf15	7.7	8.4	0.7	44.7	-1.5	1.76
5cf16	8.4	8.7	0.3	36.8	-7.9	0.99
5cf17	8.7	9.2	0.5	48.0	11.2	2.22
5cf18	9.2	9.8	0.6	38.7	-9.4	1.31
5cf19	9.8	10	0.2	36.5	-2.2	0
5cf20	10	10.3	0.3	38.7	2.2	1.49
5cf21	10.3	10.7	0.4	43.6	4.9	2.15
5cf22	10.7	11	0.3	47.4	3.8	1.25
5cf23	11	11.2	0.2	54.3	6.9	0
5cf24	11.2	12	0.8	44.2	-10.1	3.22
5cf25	12	12.8	0.8	46.8	2.6	3.87
5cf26	12.8	13	0.2	37.7	-9.1	0
5cf27	13	13.3	0.3	36.7	-1.0	0.17
5cf28	13.3	13.7	0.4	38.0	1.3	0.74
5cf29	13.7	14	0.3	47.2	9.2	3.78
5cf30	14	14.7	0.7	44.7	-2.5	1.8
5cf31	14.7	15.4	0.7	40.1	-4.6	0.98
5cf32	15.4	16.1	0.7	43.2	3.2	2.5
5cf33	16.1	16.5	0.4	50.0	6.8	1.66
5cf34	16.5	16.7	0.2	39.4	-10.7	0
5cf35	16.7	18.2	1.5	50.1	10.7	3.15
5cf36	18.2	18.5	0.3	42.7	-7.5	1.12
5cf37	18.5	19.5	1	49.2	6.6	2.47
5cf38	19.5	19.7	0.2	38.4	-10.8	0
5cf39	19.7	20.9	1.2	47.0	8.6	1.65
5cf40	20.9	21.1	0.2	40.0	-7.0	0
5cf41	21.1	21.6	0.5	44.9	4.9	2.92
5cf42	21.6	22	0.4	37.0	-7.9	1.41
5cf43	22	22.5	0.5	46.5	9.5	3.84
5cf44	22.5	22.7	0.2	45.1	-1.4	0
5cf45	22.7	23	0.3	49.2	4.1	1.57
5cf46	23	23.6	0.6	43.3	-5.9	2.01
5cf47	23.6	24.6	1	40.9	-2.4	2.13
5cf48	24.6	24.8	0.2	44.8	3.9	0
5cf49	24.8	26.7	1.9	39.5	-5.2	1.58
5cf50	26.7	26.9	0.2	36.8	-2.7	0
5cf51	26.9	27.5	0.6	38.9	2.1	1.84
5cf52	27.5	27.7	0.2	42.4	3.4	0
5cf53	27.7	28.5	0.8	38.7	-3.7	1.35
5cf54	28.5	28.9	0.4	36.9	-1.7	0.63
5cf55	28.9	29.6	0.7	38.7	1.7	1.24
5cf56	29.6	29.9	0.3	36.8	-1.9	0.09
5cf57	29.9	30.6	0.7	38.2	1.4	0.54
5cf58	30.6	31	0.4	38.2	0.0	1.49
5cf59	31	31.4	0.4	43.2	5.0	1.25
5cf60	31.4	31.8	0.4	38.5	-4.7	0.81
5cf61	31.8	32.6	0.8	43.0	4.5	1.44
5cf62	32.6	33	0.4	39.9	-3.0	0.79
5cf63	33	33.4	0.4	46.4	6.4	2.49
5cf64	33.4	34.8	1.4	54.2	7.9	4.51
5cf65	34.8	35.1	0.3	48.8	-5.4	4.62
5cf66	35.1	35.4	0.3	56.2	7.4	2.06
5cf67	35.4	36.1	0.7	52.5	-3.7	1.4

5cf68	36.1	36.9	0.8	48.9	-3.6	2.31
5cf69	36.9	37.1	0.2	42.6	-6.3	0
5cf70	37.1	37.7	0.6	48.4	5.7	2.65
5cf71	37.7	37.9	0.2	40.4	-8.0	0
5cf72	37.9	38.1	0.2	48.3	8.0	0
5cf73	38.1	40.1	2	43.4	-4.9	2.73
5cf74	40.1	40.9	0.8	40.8	-2.5	1.27
5cf75	40.9	41.2	0.3	44.9	4.0	3.02
5cf76	41.2	41.8	0.6	40.6	-4.3	1.07
5cf77	41.8	42.2	0.4	44.0	3.4	0.74
5cf78	42.2	42.4	0.2	39.7	-4.3	0
5cf79	42.4	43.1	0.7	43.3	3.6	3.71
5cf80	43.1	43.4	0.3	40.2	-3.1	0.61
5cf81	43.4	43.8	0.4	44.5	4.2	1.75
5cf82	43.8	44.6	0.8	50.1	5.7	4.45
5cf83	44.6	44.8	0.2	56.0	5.8	0
5cf84	44.8	45.5	0.7	48.6	-7.4	2.73
5cf85	45.5	45.7	0.2	43.2	-5.4	0
5cf86	45.7	46	0.3	49.8	6.5	6.63
5cf87	46	46.4	0.4	38.8	-10.9	0.9
5cf88	46.4	46.7	0.3	36.2	-2.7	1.11
5cf89	46.7	49.4	2.7	39.3	3.1	1.69
5cf90	49.4	49.6	0.2	42.0	2.7	0
5cf91	49.6	50.7	1.1	37.8	-4.1	1.8
5cf92	50.7	50.9	0.2	44.3	6.4	0
5cf93	50.9	51.9	1	39.3	-5.0	1.98
5cf94	51.9	52.1	0.2	42.6	3.3	0
5cf95	52.1	52.5	0.4	37.8	-4.9	1.02
5cf96	52.5	52.7	0.2	36.4	-1.4	0
5cf97	52.7	52.9	0.2	38.4	2.1	0
5cf98	52.9	54	1.1	42.0	3.5	2.75
5cf99	54	54.5	0.5	40.3	-1.6	0.84
5cf100	54.5	54.7	0.2	41.4	1.1	0
5cf101	54.7	55.1	0.4	39.9	-1.5	0.62
5cf102	55.1	55.7	0.6	43.1	3.2	0.92
5cf103	55.7	55.9	0.2	37.9	-5.1	0
5cf104	55.9	56.2	0.3	44.7	6.7	1.34
5cf105	56.2	56.5	0.3	39.3	-5.4	0.75
5cf106	56.5	57	0.5	43.4	4.1	2.25
5cf107	57	57.2	0.2	40.6	-2.8	0
5cf108	57.2	57.9	0.7	50.8	10.2	2.01
5cf109	57.9	58.2	0.3	41.9	-8.8	1.18
5cf110	58.2	58.9	0.7	48.5	6.6	2.88
5cf111	58.9	59.1	0.2	40.3	-8.2	0
5cf112	59.1	59.7	0.6	55.4	15.1	2.21
5cf113	59.7	60.1	0.4	49.8	-5.7	3.6
5cf114	60.1	61.3	1.2	57.8	8.0	2.62
5cf115	61.3	62.1	0.8	49.4	-8.4	2.78
5cf116	62.1	62.4	0.3	44.4	-5.0	1.34
5cf117	62.4	62.9	0.5	49.7	5.4	1.78
5cf118	62.9	63.5	0.6	53.2	3.4	1.9
5cf119	63.5	64.4	0.9	46.8	-6.4	4.26
5cf120	64.4	64.9	0.5	44.2	-2.6	3.1
5cf121	64.9	65.2	0.3	39.0	-5.2	1.51
5cf122	65.2	66.1	0.9	48.7	9.7	3.55
5cf123	66.1	66.5	0.4	41.2	-7.5	1.53

5cf124	66.5	67.2	0.7	48.6	7.4	2.82
5cf125	67.2	68.1	0.9	56.5	7.9	3.55
5cf126	68.1	68.3	0.2	50.7	-5.9	0
5cf127	68.3	68.5	0.2	54.3	3.7	0
5cf128	68.5	70	1.5	50.8	-3.6	1.75
5cf129	70	70.5	0.5	57.4	6.6	1.16
5cf130	70.5	71.6	1.1	49.4	-8.0	3.4
5cf131	71.6	72	0.4	43.1	-6.3	0.59
5cf132	72	72.4	0.4	40.5	-2.6	0.23
5cf133	72.4	73.1	0.7	41.6	1.1	1.18
5cf134	73.1	73.5	0.4	50.9	9.3	1.5
5cf135	73.5	73.9	0.4	44.2	-6.7	1.27
5cf136	73.9	74.7	0.8	41.0	-3.1	1.52
5cf137	74.7	75.1	0.4	43.9	2.8	1.47
5cf138	75.1	75.3	0.2	46.6	2.7	0
5cf139	75.3	77	1.7	43.0	-3.5	1.2
5cf140	77	77.6	0.6	38.4	-4.6	0.8
5cf141	77.6	77.9	0.3	36.2	-2.2	0.92
5cf142	77.9	78.1	0.2	38.5	2.3	0
5cf143	78.1	78.4	0.3	42.1	3.6	2.9
5cf144	78.4	78.6	0.2	48.7	6.6	0
5cf145	78.6	79.4	0.8	43.7	-5.0	3.98
5cf146	79.4	79.9	0.5	46.6	2.9	5.18
5cf147	79.9	80.1	0.2	38.7	-7.9	0
5cf148	80.1	80.9	0.8	42.3	3.6	2.62
5cf149	80.9	81.1	0.2	38.5	-3.8	0
5cf150	81.1	81.5	0.4	36.3	-2.2	2.71
5cf151	81.5	81.7	0.2	48.0	11.7	0
5cf152	81.7	82.1	0.4	42.9	-5.1	1.15
5cf153	82.1	82.8	0.7	39.2	-3.7	1.94
5cf154	82.8	83.8	1	41.5	2.3	2.82
5cf155	83.8	87.2	3.4	46.6	5.1	3.99
5cf156	87.2	87.4	0.2	38.8	-7.8	0
5cf157	87.4	88.8	1.4	36.7	-2.1	0.72
5cf158	88.8	90.9	2.1	39.0	2.2	2.51
5cf159	90.9	91.1	0.2	41.5	2.5	0
5cf160	91.1	91.3	0.2	40.0	-1.5	0
5cf161	91.3	91.6	0.3	42.9	2.9	1.53
5cf162	91.6	92	0.4	39.1	-3.8	1.21
6cf1	0	3	3	0.0		0
6cf2	3	3.3	0.3	47.0		1.69
6cf3	3.3	3.9	0.6	42.0	-5.0	1.97
6cf4	3.9	4.7	0.8	40.2	-1.8	1.39
6cf5	4.7	5.7	1	44.1	3.8	1.77
6cf6	5.7	5.9	0.2	46.8	2.8	0
6cf7	5.9	6.2	0.3	43.2	-3.6	2.08
6cf8	6.2	6.9	0.7	38.9	-4.4	1.41
6cf9	6.9	8.4	1.5	43.4	4.5	1.27
6cf10	8.4	8.7	0.3	48.9	5.5	2.5
6cf11	8.7	8.9	0.2	44.0	-4.8	0
6cf12	8.9	9.1	0.2	55.0	11.0	0
6cf13	9.1	9.9	0.8	48.7	-6.3	2.79
6cf14	9.9	10.2	0.3	45.3	-3.4	0.82
6cf15	10.2	10.4	0.2	48.1	2.8	0
6cf16	10.4	10.9	0.5	52.0	3.9	3.11

6cf17	10.9	12.4	1.5	49.3	-2.6	2.4
6cf18	12.4	12.7	0.3	44.7	-4.6	1.67
6cf19	12.7	12.9	0.2	40.6	-4.1	0
6cf20	12.9	13.1	0.2	42.2	1.6	0
6cf21	13.1	13.3	0.2	47.2	5.0	0
6cf22	13.3	13.6	0.3	43.3	-3.9	2.37
6cf23	13.6	14.2	0.6	46.6	3.2	1.28
6cf24	14.2	14.8	0.6	43.4	-3.1	0.97
6cf25	14.8	15	0.2	48.5	5.1	0
6cf26	15	15.3	0.3	43.3	-5.2	1.79
6cf27	15.3	16.3	1	50.4	7.1	2.97
6cf28	16.3	16.5	0.2	44.1	-6.3	0
6cf29	16.5	16.8	0.3	39.4	-4.7	1.06
6cf30	16.8	17.1	0.3	38.3	-1.1	2.3
6cf31	17.1	17.6	0.5	48.7	10.5	3.88
6cf32	17.6	17.8	0.2	57.0	8.2	0
6cf33	17.8	18.2	0.4	51.6	-5.3	1.08
6cf34	18.2	19	0.8	54.6	3.0	4.03
6cf35	19	19.2	0.2	49.2	-5.5	0
6cf36	19.2	19.5	0.3	55.8	6.6	0.96
6cf37	19.5	19.7	0.2	48.9	-6.8	0
6cf38	19.7	20.1	0.4	44.0	-4.9	1.18
6cf39	20.1	22.6	2.5	49.0	5.0	2.05
6cf40	22.6	24	1.4	43.1	-5.9	1.43
6cf41	24	24.3	0.3	46.4	3.3	0.55
6cf42	24.3	24.6	0.3	41.0	-5.4	0.6
6cf43	24.6	25.4	0.8	44.5	3.4	0.93
6cf44	25.4	25.6	0.2	46.5	2.0	0
6cf45	25.6	26.6	1	43.8	-2.8	2.38
6cf46	26.6	27.7	1.1	39.1	-4.7	1.69
6cf47	27.7	29.7	2	42.7	3.6	1.65
6cf48	29.7	30.1	0.4	47.6	4.9	0.65
6cf49	30.1	30.8	0.7	42.4	-5.3	1.64
6cf50	30.8	31.3	0.5	48.2	5.9	1.96
6cf51	31.3	32.4	1.1	43.5	-4.7	3.1
6cf52	32.4	32.8	0.4	40.7	-2.9	0.24
6cf53	32.8	33.3	0.5	43.6	2.9	0.64
6cf54	33.3	33.8	0.5	47.9	4.4	2.04
6cf55	33.8	34.2	0.4	44.1	-3.8	1.27
6cf56	34.2	35.4	1.2	49.3	5.2	4.22
6cf57	35.4	35.9	0.5	44.6	-4.7	0.99
6cf58	35.9	36.7	0.8	49.3	4.7	1.97
6cf59	36.7	38	1.3	42.8	-6.5	1.77
6cf60	38	38.7	0.7	39.8	-3.0	0.62
6cf61	38.7	39.4	0.7	42.9	3.0	1.08
6cf62	39.4	40	0.6	50.4	7.6	2.03
6cf63	40	40.2	0.2	54.0	3.6	0
6cf64	40.2	40.5	0.3	44.2	-9.8	0.66
6cf65	40.5	41.1	0.6	47.1	2.9	1.27
6cf66	41.1	43.5	2.4	58.4	11.3	4.62
6cf67	43.5	43.9	0.4	43.8	-14.6	1.17
6cf68	43.9	44.3	0.4	39.0	-4.8	1.51
6cf69	44.3	44.8	0.5	47.2	8.2	3.84
6cf70	44.8	45	0.2	54.3	7.2	0
6cf71	45	45.7	0.7	47.8	-6.6	2.86
6cf72	45.7	47.9	2.2	39.3	-8.5	1.91

6cf73	47.9	51.8	3.9	34.9	-4.4	1.03
6cf74	51.8	52	0.2	41.7	6.8	0
6cf75	52	54	2	38.2	-3.5	1.19
6cf76	54	56	2	35.3	-2.9	1.41
6cf77	56	56.2	0.2	38.1	2.8	0
6cf78	56.2	56.7	0.5	36.1	-2.0	0.61
6cf79	56.7	57.4	0.7	39.3	3.3	0.76
6cf80	57.4	57.8	0.4	44.4	5.0	0.85
6cf81	57.8	58.1	0.3	38.1	-6.3	2.45
6cf82	58.1	58.6	0.5	44.5	6.4	1.51
6cf83	58.6	59.1	0.5	37.0	-7.5	1.85
6cf84	59.1	59.3	0.2	38.2	1.3	0
6cf85	59.3	60	0.7	36.9	-1.4	2.03
6cf86	60	60.2	0.2	39.6	2.7	0
6cf87	60.2	60.4	0.2	42.0	2.4	0
6cf88	60.4	60.7	0.3	38.1	-3.8	3.84
6cf89	60.7	60.9	0.2	36.3	-1.8	0
6cf90	60.9	61.7	0.8	39.7	3.4	1.65
6cf91	61.7	62	0.3	42.6	2.8	0.36
6cf92	62	63.9	1.9	38.4	-4.1	1.51
6cf93	63.9	64.2	0.3	44.0	5.6	2.28
6cf94	64.2	65.4	1.2	36.4	-7.5	2.01
6cf95	65.4	66	0.6	39.4	3.0	1.26
6cf96	66	66.3	0.3	42.3	2.9	0.38
6cf97	66.3	66.7	0.4	38.8	-3.5	2.18
6cf98	66.7	67	0.3	35.5	-3.3	1.16
6cf99	67	67.3	0.3	41.0	5.5	1.62
6cf100	67.3	68.3	1	36.0	-5.0	0.89
6cf101	68.3	68.7	0.4	37.3	1.3	0.63
6cf102	68.7	68.9	0.2	34.6	-2.7	0
6cf103	68.9	69.3	0.4	38.2	3.6	0.7
6cf104	69.3	71.4	2.1	35.5	-2.7	0.8
6cf105	71.4	72.3	0.9	38.0	2.4	1.26
6cf106	72.3	72.5	0.2	42.4	4.5	0
6cf107	72.5	72.7	0.2	38.1	-4.3	0
6cf108	72.7	73.1	0.4	41.4	3.3	0.9
6cf109	73.1	73.4	0.3	39.2	-2.3	0.64
6cf110	73.4	73.7	0.3	43.6	4.4	2.23
6cf111	73.7	74.7	1	37.7	-5.9	0.7
6cf112	74.7	75	0.3	43.1	5.4	0.84
6cf113	75	75.4	0.4	35.3	-7.8	1.35
6cf114	75.4	75.6	0.2	37.5	2.2	0
6cf115	75.6	77.3	1.7	35.5	-2.0	0.8
6cf116	77.3	77.8	0.5	39.0	3.5	1.48
6cf117	77.8	78.1	0.3	41.8	2.8	0.42
6cf118	78.1	80	1.9	39.2	-2.7	2.19
6cf119	80	80.7	0.7	51.5	12.3	2.55
7cf1	0	3	3	0.0		0
7cf2	3	3.2	0.2	53.8		0
7cf3	3.2	3.7	0.5	47.1	-6.8	8.03
7cf4	3.7	4	0.3	58.4	11.3	6.25
7cf5	4	4.2	0.2	49.2	-9.2	0
7cf6	4.2	5.2	1	58.1	8.9	2.8
7cf7	5.2	5.7	0.5	41.1	-17.0	3.11
7cf8	5.7	6.1	0.4	44.6	3.4	2.37

7cf9	6.1	6.3	0.2	38.7	-5.9	0
7cf10	6.3	6.9	0.6	35.8	-2.9	0.7
7cf11	6.9	8	1.1	38.6	2.8	1.57
7cf12	8	8.7	0.7	36.0	-2.6	2.55
7cf13	8.7	9.2	0.5	47.0	11.0	3.19
7cf14	9.2	9.6	0.4	39.3	-7.7	0.59
7cf15	9.6	10.1	0.5	50.0	10.7	2.87
7cf16	10.1	10.4	0.3	43.3	-6.8	0.61
7cf17	10.4	10.8	0.4	40.4	-2.8	0.84
7cf18	10.8	11.5	0.7	45.2	4.7	2.96
7cf19	11.5	11.8	0.3	36.8	-8.3	1.39
7cf20	11.8	12.8	1	42.8	6.0	2.7
7cf21	12.8	13	0.2	47.5	4.7	0
7cf22	13	13.2	0.2	42.1	-5.3	0
7cf23	13.2	13.5	0.3	39.5	-2.6	2.15
7cf24	13.5	14.1	0.6	44.4	4.9	2.79
7cf25	14.1	14.3	0.2	36.4	-8.0	0
7cf26	14.3	15.5	1.2	42.4	5.9	0.88
7cf27	15.5	16.8	1.3	39.2	-3.2	4.23
7cf28	16.8	17.3	0.5	37.2	-2.0	3.1
7cf29	17.3	18.2	0.9	43.6	6.4	1.58
7cf30	18.2	18.4	0.2	39.0	-4.6	0
7cf31	18.4	18.7	0.3	42.1	3.0	0.38
7cf32	18.7	18.9	0.2	39.7	-2.4	0
7cf33	18.9	19.1	0.2	44.1	4.4	0
7cf34	19.1	19.5	0.4	39.1	-5.0	1.56
7cf35	19.5	19.7	0.2	45.3	6.2	0
7cf36	19.7	21.2	1.5	40.5	-4.8	1.36
7cf37	21.2	21.4	0.2	36.3	-4.1	0
7cf38	21.4	21.8	0.4	38.5	2.2	0.58
7cf39	21.8	22.5	0.7	35.7	-2.8	1.45
7cf40	22.5	24.3	1.8	39.8	4.0	3.5
7cf41	24.3	24.5	0.2	35.7	-4.1	0
7cf42	24.5	24.9	0.4	41.9	6.2	1.83
7cf43	24.9	25.2	0.3	38.6	-3.4	0.79
7cf44	25.2	25.8	0.6	43.4	4.8	0.94
7cf45	25.8	26.3	0.5	39.9	-3.4	0.44
7cf46	26.3	26.5	0.2	34.7	-5.2	0
7cf47	26.5	27.3	0.8	44.4	9.7	0.8
7cf48	27.3	27.6	0.3	38.8	-5.6	1.37
7cf49	27.6	28.1	0.5	35.6	-3.2	0.25
7cf50	28.1	29.6	1.5	38.3	2.7	1.13
7cf51	29.6	30.1	0.5	36.9	-1.5	0.78
7cf52	30.1	30.4	0.3	42.8	6.0	2.07
7cf53	30.4	30.7	0.3	39.4	-3.5	1.36
7cf54	30.7	30.9	0.2	36.6	-2.7	0
7cf55	30.9	31.5	0.6	38.3	1.7	1.19
7cf56	31.5	31.7	0.2	34.2	-4.1	0
7cf57	31.7	32.1	0.4	39.5	5.3	0.52
7cf58	32.1	32.4	0.3	36.4	-3.1	0.42
7cf59	32.4	33	0.6	39.9	3.5	1.66
7cf60	33	34.5	1.5	42.0	2.1	3.24
7cf61	34.5	34.7	0.2	39.4	-2.6	0
7cf62	34.7	35.7	1	36.5	-2.9	0.9
7cf63	35.7	38.1	2.4	39.3	2.8	1.45
7cf64	38.1	38.4	0.3	43.5	4.2	0.9

7cf65	38.4	38.7	0.3	37.9	-5.6	2.16
7cf66	38.7	39.8	1.1	43.5	5.6	2.15
7cf67	39.8	40.8	1	40.0	-3.5	2.15
7cf68	40.8	41	0.2	36.5	-3.5	0
7cf69	41	41.5	0.5	50.4	14.0	2.48
7cf70	41.5	41.7	0.2	43.4	-7.0	0
7cf71	41.7	42.3	0.6	47.3	3.9	3.59
7cf72	42.3	42.6	0.3	38.9	-8.4	1.28
7cf73	42.6	42.9	0.3	48.8	9.9	3.45
7cf74	42.9	43.2	0.3	44.1	-4.7	1.71
7cf75	43.2	43.5	0.3	50.0	5.8	3.84
7cf76	43.5	43.7	0.2	44.2	-5.8	0
7cf77	43.7	44.8	1.1	54.0	9.8	1.96
7cf78	44.8	45	0.2	46.9	-7.0	0
7cf79	45	45.2	0.2	39.4	-7.6	0
7cf80	45.2	45.6	0.4	56.5	17.1	2.41
7cf81	45.6	46.6	1	49.0	-7.5	2.65
7cf82	46.6	46.8	0.2	42.7	-6.3	0
7cf83	46.8	47.2	0.4	46.9	4.2	0.83
7cf84	47.2	47.6	0.4	41.4	-5.5	2.84
7cf85	47.6	48	0.4	49.2	7.7	2.31
7cf86	48	48.3	0.3	41.5	-7.7	0.74
7cf87	48.3	48.5	0.2	47.8	6.3	0
7cf88	48.5	48.8	0.3	42.5	-5.2	1.23
7cf89	48.8	49	0.2	39.6	-2.9	0
7cf90	49	49.4	0.4	43.2	3.6	1.18
7cf91	49.4	49.6	0.2	38.0	-5.2	0
7cf92	49.6	51.1	1.5	36.1	-2.0	0.7
7cf93	51.1	52.4	1.3	38.1	2.0	1.04
7cf94	52.4	53.7	1.3	35.8	-2.2	0.53
7cf95	53.7	53.9	0.2	37.7	1.8	0
7cf96	53.9	54.8	0.9	36.4	-1.3	0.74
7cf97	54.8	55	0.2	37.2	0.8	0
7cf98	55	55.2	0.2	36.0	-1.2	0
7cf99	55.2	55.5	0.3	39.3	3.3	1.69
7cf100	55.5	55.7	0.2	43.8	4.5	0
7cf101	55.7	56	0.3	53.9	10.1	1.29
7cf102	56	56.4	0.4	36.6	-17.3	1.14
7cf103	56.4	57	0.6	43.3	6.8	1.24
7cf104	57	57.2	0.2	38.2	-5.1	0
7cf105	57.2	57.6	0.4	41.9	3.7	1.69
7cf106	57.6	58.6	1	39.1	-2.9	1.44
7cf107	58.6	59.8	1.2	35.2	-3.9	1.16
7cf108	59.8	61.1	1.3	38.6	3.4	1.19
7cf109	61.1	63.9	2.8	35.1	-3.5	1.27
7cf110	63.9	65	1.1	38.1	3.1	1.15
7cf111	65	65.3	0.3	42.8	4.7	0.7
7cf112	65.3	66.4	1.1	38.3	-4.5	1.49
7cf113	66.4	66.7	0.3	41.7	3.4	0.39
7cf114	66.7	67.4	0.7	39.5	-2.2	1.25
7cf115	67.4	68	0.6	42.0	2.6	0.67
7cf116	68	68.2	0.2	47.2	5.2	0
7cf117	68.2	68.8	0.6	38.6	-8.6	1.66
7cf118	68.8	69.2	0.4	42.6	4.0	1.15
7cf119	69.2	69.9	0.7	37.8	-4.8	1.16
7cf120	69.9	70.2	0.3	35.9	-1.8	0.67

7cf121	70.2	70.9	0.7	40.3	4.4	1.85
7cf122	70.9	72.3	1.4	35.8	-4.5	0.63
7cf123	72.3	73.1	0.8	38.7	2.9	2.13
7cf124	73.1	73.3	0.2	41.5	2.8	0
7cf125	73.3	73.7	0.4	38.8	-2.8	1.27
7cf126	73.7	74.2	0.5	36.6	-2.2	1.09
7cf127	74.2	76.4	2.2	39.2	2.7	1.75
7cf128	76.4	76.8	0.4	42.1	2.9	1.22
7cf129	76.8	77.6	0.8	39.4	-2.7	0.92
7cf130	77.6	78.2	0.6	43.2	3.8	1.64
7cf131	78.2	78.7	0.5	38.4	-4.8	2.17
7cf132	78.7	79.5	0.8	42.8	4.4	1.44
7cf133	79.5	80.3	0.8	38.7	-4.1	1.3
7cf134	80.3	80.9	0.6	43.9	5.2	2.02
7cf135	80.9	81.6	0.7	38.5	-5.4	1.09
7cf136	81.6	81.9	0.3	46.0	7.4	1.24
7cf137	81.9	82.4	0.5	47.7	1.7	3.33
7cf138	82.4	82.6	0.2	39.1	-8.5	0
7cf139	82.6	83	0.4	52.0	12.9	5.24
7cf140	83	84	1	49.9	-2.1	2.41
8cf1	0	3	3	0.0		0
8cf2	3	3.4	0.4	43.4		3.81
8cf3	3.4	4.1	0.7	39.9	-3.4	1.54
8cf4	4.1	4.4	0.3	42.2	2.3	3.02
8cf5	4.4	4.6	0.2	49.4	7.1	0
8cf6	4.6	4.8	0.2	55.0	5.6	0
8cf7	4.8	5.2	0.4	49.4	-5.6	3.7
8cf8	5.2	5.8	0.6	40.0	-9.4	0.91
8cf9	5.8	6.1	0.3	42.6	2.5	0.82
8cf10	6.1	6.4	0.3	47.5	4.9	1.03
8cf11	6.4	6.6	0.2	44.3	-3.1	0
8cf12	6.6	7.5	0.9	48.8	4.4	3.47
8cf13	7.5	7.7	0.2	42.5	-6.2	0
8cf14	7.7	9.1	1.4	38.5	-4.1	1.49
8cf15	9.1	14	4.9	35.7	-2.8	1.41
8cf16	14	14.7	0.7	38.6	3.0	1.83
8cf17	14.7	15.3	0.6	36.7	-1.9	0.82
8cf18	15.3	15.7	0.4	38.6	1.9	0.75
8cf19	15.7	16.5	0.8	42.0	3.4	1.5
8cf20	16.5	16.9	0.4	37.4	-4.7	1.49
8cf21	16.9	17.2	0.3	40.3	2.9	3.19
8cf22	17.2	17.6	0.4	35.5	-4.8	1.02
8cf23	17.6	18	0.4	37.7	2.2	0.74
8cf24	18	18.2	0.2	41.4	3.7	0
8cf25	18.2	18.6	0.4	38.4	-3.0	2.05
8cf26	18.6	19.3	0.7	35.7	-2.7	1.53
8cf27	19.3	20.6	1.3	37.2	1.5	0.9
8cf28	20.6	25.2	4.6	34.7	-2.5	0.87
8cf29	25.2	25.4	0.2	37.5	2.8	0
8cf30	25.4	28.8	3.4	35.0	-2.5	1.01
8cf31	28.8	29.9	1.1	39.1	4.1	2.32
8cf32	29.9	30.1	0.2	35.7	-3.4	0
8cf33	30.1	31.7	1.6	39.8	4.0	1.29
8cf34	31.7	31.9	0.2	35.7	-4.1	0
8cf35	31.9	33.5	1.6	38.8	3.1	1.4

8cf36	33.5	34	0.5	41.1	2.3	2.04
8cf37	34	35.3	1.3	39.4	-1.7	1.76
8cf38	35.3	35.5	0.2	42.3	2.9	0
8cf39	35.5	36.7	1.2	39.3	-3.1	1.73
8cf40	36.7	36.9	0.2	36.1	-3.1	0
8cf41	36.9	38.1	1.2	39.1	3.0	0.91
8cf42	38.1	38.3	0.2	36.2	-2.9	0
8cf43	38.3	38.8	0.5	39.2	3.0	2.33
8cf44	38.8	39.1	0.3	36.6	-2.6	2.56
8cf45	39.1	39.5	0.4	41.5	5.0	0.33
8cf46	39.5	40.1	0.6	38.5	-3.0	1.12
8cf47	40.1	40.7	0.6	36.1	-2.3	0.24
8cf48	40.7	42.1	1.4	39.1	3.0	1.5
8cf49	42.1	42.3	0.2	48.0	8.9	0
8cf50	42.3	42.7	0.4	43.7	-4.4	1.03
8cf51	42.7	43	0.3	35.2	-8.5	0.87
8cf52	43	43.4	0.4	43.0	7.8	1
8cf53	43.4	43.6	0.2	39.6	-3.4	0
8cf54	43.6	44.4	0.8	35.3	-4.4	1.5
8cf55	44.4	44.6	0.2	46.8	11.6	0
8cf56	44.6	44.8	0.2	42.5	-4.4	0
8cf57	44.8	45.2	0.4	37.0	-5.5	1.17
8cf58	45.2	45.4	0.2	41.3	4.3	0
8cf59	45.4	45.9	0.5	48.0	6.7	2
8cf60	45.9	46.1	0.2	40.4	-7.6	0
8cf61	46.1	47	0.9	45.4	4.9	2.01
8cf62	47	47.4	0.4	39.7	-5.7	1.34
8cf63	47.4	47.9	0.5	41.2	1.5	4.1
8cf64	47.9	48.8	0.9	39.9	-1.3	1.41
8cf65	48.8	49	0.2	43.7	3.8	0
8cf66	49	49.2	0.2	46.6	2.9	0
8cf67	49.2	49.4	0.2	44.9	-1.6	0
8cf68	49.4	50.5	1.1	41.5	-3.5	3.29
8cf69	50.5	51.5	1	45.2	3.7	3.91
8cf70	51.5	51.7	0.2	45.4	0.2	0
8cf71	51.7	51.9	0.2	38.6	-6.7	0
8cf72	51.9	52.3	0.4	43.6	4.9	2.77
8cf73	52.3	53.2	0.9	48.9	5.3	2.43
8cf74	53.2	54	0.8	42.7	-6.2	2.29
8cf75	54	55.4	1.4	38.4	-4.3	1.31
8cf76	55.4	56.5	1.1	36.4	-2.0	0.9
8cf77	56.5	57.5	1	38.9	2.5	2.07
8cf78	57.5	60.2	2.7	35.8	-3.1	0.87
8cf79	60.2	60.4	0.2	39.0	3.3	0
8cf80	60.4	61.5	1.1	35.8	-3.3	0.7
8cf81	61.5	61.7	0.2	37.9	2.1	0
8cf82	61.7	61.9	0.2	36.8	-1.0	0
8cf83	61.9	62.1	0.2	38.8	2.0	0
8cf84	62.1	62.8	0.7	42.0	3.3	1.84
8cf85	62.8	63.2	0.4	38.8	-3.2	2.44
8cf86	63.2	63.9	0.7	44.9	6.1	1.76
8cf87	63.9	64.1	0.2	40.7	-4.3	0
8cf88	64.1	65.3	1.2	46.8	6.1	4.79
8cf89	65.3	65.8	0.5	39.0	-7.8	1.95
8cf90	65.8	66.4	0.6	46.9	7.9	5.66
8cf91	66.4	68.1	1.7	48.3	1.4	2.54

8cf92	68.1	68.3	0.2	40.3	-8.0	0
8cf93	68.3	68.9	0.6	46.8	6.5	3.78
8cf94	68.9	70	1.1	42.9	-3.9	1.3
8cf95	70	70.2	0.2	40.5	-2.4	0
8cf96	70.2	71.2	1	48.9	8.4	4.03
8cf97	71.2	71.4	0.2	44.4	-4.5	0
8cf98	71.4	71.9	0.5	50.5	6.1	2.64
8cf99	71.9	72.1	0.2	55.8	5.3	0
8cf100	72.1	73.2	1.1	48.6	-7.2	5.49
8cf101	73.2	73.4	0.2	45.8	-2.8	0
8cf102	73.4	73.6	0.2	49.1	3.3	0
8cf103	73.6	73.8	0.2	43.6	-5.5	0
8cf104	73.8	74.8	1	48.4	4.8	3.75
8cf105	74.8	76	1.2	60.8	12.4	5.13
8cf106	76	77.2	1.2	42.0	-18.8	0.99
8cf107	77.2	77.4	0.2	40.7	-1.3	0
9cf1	0	3	3	0.0		0
9cf2	3	4.1	1.1	60.2		3.63
9cf3	4.1	4.3	0.2	49.8	-10.4	0
9cf4	4.3	6.7	2.4	56.0	6.1	3.95
9cf5	6.7	8.5	1.8	50.3	-5.6	2.92
9cf6	8.5	8.7	0.2	55.2	4.9	0
9cf7	8.7	9	0.3	46.7	-8.5	3.2
9cf8	9	9.2	0.2	55.5	8.8	0
9cf9	9.2	9.6	0.4	51.6	-3.8	1.53
9cf10	9.6	9.9	0.3	55.6	4.0	1.07
9cf11	9.9	10.5	0.6	48.7	-6.9	1.86
9cf12	10.5	10.7	0.2	38.2	-10.5	0
9cf13	10.7	11	0.3	36.6	-1.7	0.1
9cf14	11	11.2	0.2	39.3	2.7	0
9cf15	11.2	11.8	0.6	47.3	8.0	3.58
9cf16	11.8	12.6	0.8	38.7	-8.6	1.75
9cf17	12.6	14	1.4	44.7	6.0	5.27
9cf18	14	14.2	0.2	43.7	-1.0	0
9cf19	14.2	14.5	0.3	35.9	-7.8	0.82
9cf20	14.5	14.9	0.4	44.6	8.7	4.67
9cf21	14.9	15.1	0.2	50.3	5.7	0
9cf22	15.1	15.4	0.3	43.8	-6.5	0.56
9cf23	15.4	15.7	0.3	39.7	-4.1	3.56
9cf24	15.7	15.9	0.2	39.2	-0.5	0
9cf25	15.9	16.6	0.7	42.2	2.9	2.6
9cf26	16.6	16.8	0.2	50.2	8.0	0
9cf27	16.8	17.2	0.4	43.2	-7.0	1.57
9cf28	17.2	17.4	0.2	36.5	-6.7	0
9cf29	17.4	17.6	0.2	38.6	2.1	0
9cf30	17.6	18.3	0.7	47.1	8.5	1.41
9cf31	18.3	18.5	0.2	43.9	-3.2	0
9cf32	18.5	18.7	0.2	39.0	-4.8	0
9cf33	18.7	18.9	0.2	36.0	-3.1	0
9cf34	18.9	20.9	2	38.6	2.6	1.4
9cf35	20.9	21.5	0.6	41.1	2.6	2.05
9cf36	21.5	22.1	0.6	49.7	8.5	3.31
9cf37	22.1	22.4	0.3	43.8	-5.9	1.18
9cf38	22.4	23.8	1.4	48.5	4.7	2.2
9cf39	23.8	24	0.2	44.2	-4.3	0

9cf40	24	24.7	0.7	50.4	6.2	2.15
9cf41	24.7	24.9	0.2	41.7	-8.7	0
9cf42	24.9	25.4	0.5	39.5	-2.2	0.93
9cf43	25.4	25.9	0.5	48.6	9.1	1.42
9cf44	25.9	26.8	0.9	46.0	-2.6	4.73
9cf45	26.8	27.1	0.3	53.4	7.4	0.04
9cf46	27.1	27.8	0.7	46.5	-6.9	5.25
9cf47	27.8	28	0.2	37.5	-9.0	0
9cf48	28	28.3	0.3	48.3	10.8	4.05
9cf49	28.3	28.7	0.4	45.5	-2.8	0.42
9cf50	28.7	29	0.3	49.6	4.1	4.25
9cf51	29	29.2	0.2	43.8	-5.8	0
9cf52	29.2	30.2	1	51.0	7.3	4
9cf53	30.2	30.4	0.2	39.8	-11.2	0
9cf54	30.4	30.9	0.5	42.8	3.0	0.95
9cf55	30.9	31.5	0.6	39.0	-3.8	1.58
9cf56	31.5	33.2	1.7	36.0	-2.9	0.54
9cf57	33.2	33.6	0.4	37.5	1.4	1.26
9cf58	33.6	33.8	0.2	43.1	5.6	0
9cf59	33.8	34.7	0.9	40.1	-3.0	0.98
9cf60	34.7	35	0.3	46.0	6.0	1.02
9cf61	35	35.5	0.5	45.0	-1.1	1.09
9cf62	35.5	36	0.5	48.9	3.9	0.96
9cf63	36	36.6	0.6	44.3	-4.5	2.66
9cf64	36.6	37.4	0.8	38.4	-5.9	1.86
9cf65	37.4	37.6	0.2	36.5	-1.9	0
9cf66	37.6	38	0.4	38.0	1.4	0.61
9cf67	38	39.1	1.1	38.2	0.2	5.49
9cf68	39.1	39.6	0.5	38.2	0.0	1.31
9cf69	39.6	39.8	0.2	45.3	7.1	0
9cf70	39.8	40.1	0.3	49.9	4.6	1.18
9cf71	40.1	40.7	0.6	39.7	-10.1	1.4
9cf72	40.7	41.3	0.6	44.2	4.5	2.49
9cf73	41.3	41.5	0.2	39.7	-4.5	0
9cf74	41.5	42.2	0.7	48.3	8.6	3.27
9cf75	42.2	42.4	0.2	44.4	-3.9	0
9cf76	42.4	42.6	0.2	48.4	3.9	0
9cf77	42.6	43.5	0.9	45.4	-3.0	1.09
9cf78	43.5	43.8	0.3	39.6	-5.7	1.21
9cf79	43.8	44.2	0.4	43.0	3.4	2.46
9cf80	44.2	44.4	0.2	39.1	-3.9	0
9cf81	44.4	44.6	0.2	43.4	4.3	0
9cf82	44.6	44.8	0.2	50.7	7.3	0
9cf83	44.8	45.1	0.3	36.8	-13.9	1.83
9cf84	45.1	46.7	1.6	46.6	9.8	4.48
9cf85	46.7	47.8	1.1	39.3	-7.3	3.25
9cf86	47.8	48.2	0.4	46.9	7.6	3.82
9cf87	48.2	48.6	0.4	43.9	-3.0	1.97
9cf88	48.6	49.2	0.6	47.2	3.3	3.17
9cf89	49.2	50.1	0.9	44.2	-3.0	3.87
9cf90	50.1	50.4	0.3	46.7	2.5	1.02
9cf91	50.4	50.7	0.3	39.7	-7.0	0.4
9cf92	50.7	51.6	0.9	48.3	8.7	2.62
9cf93	51.6	54.7	3.1	58.2	9.9	1.93
9cf94	54.7	56.3	1.6	48.0	-10.2	3.49
9cf95	56.3	56.5	0.2	55.0	7.0	0

9cf96	56.5	57.4	0.9	48.1	-6.9	5.47
9cf97	57.4	57.9	0.5	53.4	5.3	1.33
9cf98	57.9	58.8	0.9	50.1	-3.2	2.4
9cf99	58.8	59.6	0.8	49.4	-0.7	3.82
9cf100	59.6	59.8	0.2	43.7	-5.7	0
9cf101	59.8	60.1	0.3	50.1	6.4	2.98
9cf102	60.1	60.6	0.5	44.6	-5.5	1.35
9cf103	60.6	61.8	1.2	40.6	-4.0	3.51
9cf104	61.8	62.2	0.4	49.5	9.0	1.75
9cf105	62.2	62.6	0.4	40.3	-9.3	2.77
9cf106	62.6	62.9	0.3	48.1	7.8	4.85
9cf107	62.9	63.5	0.6	39.5	-8.5	2.11
9cf108	63.5	64.5	1	48.2	8.7	3.84
10cf1	0	3	3	0.0		0
10cf2	3	3.7	0.7	47.8		1.76
10cf3	3.7	4.1	0.4	42.8	-4.9	1.72
10cf4	4.1	4.4	0.3	53.3	10.5	2.81
10cf5	4.4	5	0.6	47.1	-6.2	5.34
10cf6	5	5.8	0.8	39.3	-7.8	0.91
10cf7	5.8	8.1	2.3	35.9	-3.4	1.24
10cf8	8.1	8.5	0.4	38.7	2.8	0.85
10cf9	8.5	8.8	0.3	36.1	-2.6	2.13
10cf10	8.8	9.1	0.3	44.8	8.7	2.49
10cf11	9.1	10.2	1.1	39.1	-5.7	1.67
10cf12	10.2	10.6	0.4	43.8	4.7	1.97
10cf13	10.6	11	0.4	38.2	-5.5	1.61
10cf14	11	11.9	0.9	42.2	4.0	1.29
10cf15	11.9	12.7	0.8	39.8	-2.5	1.4
10cf16	12.7	13.2	0.5	43.3	3.5	0.54
10cf17	13.2	13.9	0.7	39.6	-3.7	0.72
10cf18	13.9	14.9	1	42.4	2.8	2.54
10cf19	14.9	15.2	0.3	37.0	-5.4	1.34
10cf20	15.2	15.4	0.2	43.8	6.8	0
10cf21	15.4	16	0.6	39.6	-4.2	0.34
10cf22	16	16.2	0.2	43.5	3.9	0
10cf23	16.2	16.4	0.2	39.6	-3.9	0
10cf24	16.4	16.8	0.4	42.2	2.6	3.17
10cf25	16.8	17.1	0.3	37.8	-4.5	0.3
10cf26	17.1	17.3	0.2	42.1	4.3	0
10cf27	17.3	18.1	0.8	35.9	-6.2	0.77
10cf28	18.1	19.6	1.5	39.7	3.9	1.84
10cf29	19.6	21.4	1.8	57.0	17.3	2.81
10cf30	21.4	22.1	0.7	51.7	-5.3	1.84
10cf31	22.1	22.3	0.2	55.8	4.1	0
10cf32	22.3	22.6	0.3	51.6	-4.2	1.53
10cf33	22.6	22.9	0.3	58.6	7.0	0.96
10cf34	22.9	23.1	0.2	52.7	-5.9	0
10cf35	23.1	23.3	0.2	59.2	6.6	0
10cf36	23.3	23.5	0.2	43.8	-15.4	0
10cf37	23.5	24.3	0.8	52.4	8.6	5.64
10cf38	24.3	24.9	0.6	55.0	2.6	2.48
10cf39	24.9	25.1	0.2	44.1	-10.9	0
10cf40	25.1	27.1	2	50.8	6.7	3.5
10cf41	27.1	27.6	0.5	41.8	-9.0	2.43
10cf42	27.6	28.1	0.5	41.4	-0.4	3.24

10cf43	28.1	28.4	0.3	42.7	1.3	3.54
10cf44	28.4	28.9	0.5	54.3	11.6	2.87
10cf45	28.9	29.2	0.3	50.3	-4.0	1.24
10cf46	29.2	29.4	0.2	43.3	-7.0	0
10cf47	29.4	30.5	1.1	54.4	11.1	2.28
10cf48	30.5	31.3	0.8	50.6	-3.8	2.05
10cf49	31.3	31.6	0.3	40.7	-10.0	1.44
10cf50	31.6	31.9	0.3	54.6	13.9	1.26
10cf51	31.9	32.2	0.3	47.0	-7.6	2.73
10cf52	32.2	33	0.8	42.2	-4.9	1.15
10cf53	33	33.2	0.2	46.8	4.6	0
10cf54	33.2	34.3	1.1	43.8	-3.0	1.78
10cf55	34.3	34.6	0.3	48.0	4.3	0.64
10cf56	34.6	34.8	0.2	42.1	-6.0	0
10cf57	34.8	35.1	0.3	38.5	-3.5	1.76
10cf58	35.1	35.3	0.2	44.3	5.8	0
10cf59	35.3	35.5	0.2	40.8	-3.5	0
10cf60	35.5	35.7	0.2	47.4	6.6	0
10cf61	35.7	36.7	1	41.2	-6.1	2.02
10cf62	36.7	36.9	0.2	47.2	6.0	0
10cf63	36.9	37.2	0.3	43.9	-3.3	1.68
10cf64	37.2	37.4	0.2	39.7	-4.2	0
10cf65	37.4	37.8	0.4	46.7	7.0	2.77
10cf66	37.8	38.1	0.3	45.3	-1.4	0.42
10cf67	38.1	39.2	1.1	38.3	-7.0	1.68
10cf68	39.2	39.4	0.2	36.8	-1.6	0
10cf69	39.4	40	0.6	38.7	2.0	0.91
10cf70	40	41.8	1.8	43.6	4.9	1.44
10cf71	41.8	42.2	0.4	39.3	-4.3	1.33
10cf72	42.2	43	0.8	36.4	-2.8	0.66
10cf73	43	43.9	0.9	39.6	3.1	0.9
10cf74	43.9	44.2	0.3	43.6	4.0	1.02
10cf75	44.2	44.6	0.4	40.0	-3.6	0.6
10cf76	44.6	45.6	1	42.8	2.8	1.81
10cf77	45.6	46.8	1.2	38.8	-3.9	1.93
10cf78	46.8	47	0.2	42.9	4.0	0
10cf79	47	47.2	0.2	39.3	-3.6	0
10cf80	47.2	47.4	0.2	45.3	6.0	0
10cf81	47.4	48	0.6	38.5	-6.8	1.25
10cf82	48	49	1	48.2	9.7	3.29
10cf83	49	50.3	1.3	38.5	-9.7	1.7
10cf84	50.3	50.6	0.3	47.2	8.8	2.42
10cf85	50.6	50.8	0.2	45.7	-1.5	0
10cf86	50.8	51.1	0.3	38.3	-7.5	2.79
10cf87	51.1	52.4	1.3	46.1	7.9	3.34
10cf88	52.4	52.9	0.5	44.0	-2.2	3.14
10cf89	52.9	53.7	0.8	39.5	-4.4	1.35
10cf90	53.7	53.9	0.2	41.4	1.9	0
10cf91	53.9	54.2	0.3	38.8	-2.6	0.61
10cf92	54.2	57.4	3.2	35.5	-3.3	1.15
10cf93	57.4	58.6	1.2	38.6	3.2	1.37
10cf94	58.6	58.8	0.2	43.1	4.4	0
10cf95	58.8	59.1	0.3	39.6	-3.4	0.77
10cf96	59.1	59.3	0.2	41.5	1.8	0
10cf97	59.3	59.6	0.3	36.1	-5.4	0.6
10cf98	59.6	60.4	0.8	39.0	2.9	2.11

10cf99	60.4	61.9	1.5	35.6	-3.4	0.65
10cf100	61.9	62.1	0.2	37.5	1.9	0
10cf101	62.1	62.3	0.2	36.1	-1.4	0
10cf102	62.3	63.1	0.8	39.0	2.9	1.58
10cf103	63.1	64.1	1	42.6	3.5	1.64
10cf104	64.1	65.2	1.1	38.5	-4.1	1.76
10cf105	65.2	65.5	0.3	45.3	6.8	2
10cf106	65.5	65.7	0.2	37.9	-7.3	0
10cf107	65.7	66.4	0.7	35.7	-2.2	1.29
10cf108	66.4	67	0.6	37.7	2.0	0.84
10cf109	67	67.5	0.5	44.2	6.5	2.37
10cf110	67.5	67.7	0.2	48.6	4.4	0
10cf111	67.7	67.9	0.2	41.0	-7.6	0
10cf112	67.9	68.2	0.3	44.9	3.9	2.15
10cf113	68.2	68.5	0.3	40.1	-4.8	1.05
10cf114	68.5	69.3	0.8	42.6	2.5	1.65
10cf115	69.3	70	0.7	39.4	-3.2	2.23
10cf116	70	70.7	0.7	43.1	3.6	2.11
10cf117	70.7	71.4	0.7	47.8	4.7	2.73
10cf118	71.4	71.7	0.3	43.7	-4.1	2.09
10cf119	71.7	72.5	0.8	51.8	8.1	4.91
11cf1	0	3	3	0.0		0
11cf2	3	3.5	0.5	43.8		2.4
11cf3	3.5	4.1	0.6	39.7	-4.2	1.12
11cf4	4.1	4.6	0.5	44.9	5.3	4.1
11cf5	4.6	5.2	0.6	45.3	0.4	3.96
11cf6	5.2	5.5	0.3	49.8	4.5	1
11cf7	5.5	6	0.5	43.0	-6.8	1.01
11cf8	6	6.5	0.5	48.6	5.6	2.26
11cf9	6.5	8.1	1.6	40.2	-8.4	1.17
11cf10	8.1	8.4	0.3	42.6	2.4	0.69
11cf11	8.4	8.8	0.4	39.8	-2.8	1.13
11cf12	8.8	9	0.2	36.4	-3.4	0
11cf13	9	9.5	0.5	43.6	7.3	1.18
11cf14	9.5	9.9	0.4	38.8	-4.8	1.49
11cf15	9.9	10.7	0.8	36.4	-2.4	0.66
11cf16	10.7	11.8	1.1	38.6	2.2	2.13
11cf17	11.8	12	0.2	41.5	2.9	0
11cf18	12	12.4	0.4	36.6	-4.9	0.74
11cf19	12.4	12.7	0.3	38.1	1.5	1.48
11cf20	12.7	13.1	0.4	36.7	-1.4	0.7
11cf21	13.1	13.3	0.2	38.0	1.3	0
11cf22	13.3	13.7	0.4	36.7	-1.3	0.12
11cf23	13.7	14.2	0.5	37.9	1.2	1.33
11cf24	14.2	14.7	0.5	36.2	-1.8	0.58
11cf25	14.7	15.8	1.1	38.9	2.7	1.99
11cf26	15.8	16.1	0.3	42.9	4.0	1.23
11cf27	16.1	17.1	1	38.9	-4.0	1.3
11cf28	17.1	17.5	0.4	42.1	3.1	1.17
11cf29	17.5	18.2	0.7	38.4	-3.7	1.35
11cf30	18.2	18.5	0.3	36.2	-2.3	0.9
11cf31	18.5	18.7	0.2	38.7	2.6	0
11cf32	18.7	19.3	0.6	41.6	2.8	0.97
11cf33	19.3	20.4	1.1	38.3	-3.3	1.1
11cf34	20.4	20.7	0.3	36.6	-1.6	0.59

11cf35	20.7	21.3	0.6	37.6	1.0	1.08
11cf36	21.3	22.4	1.1	35.8	-1.8	1.12
11cf37	22.4	22.8	0.4	37.9	2.1	0.47
11cf38	22.8	23.2	0.4	36.4	-1.5	0.51
11cf39	23.2	24.5	1.3	42.8	6.4	3.09
11cf40	24.5	25.4	0.9	47.2	4.4	2.55
11cf41	25.4	26.1	0.7	42.3	-5.0	4.11
11cf42	26.1	26.3	0.2	49.7	7.4	0
11cf43	26.3	26.5	0.2	45.8	-3.9	0
11cf44	26.5	27.2	0.7	45.9	0.2	3.82
11cf45	27.2	27.4	0.2	40.4	-5.6	0
11cf46	27.4	28.5	1.1	43.3	2.9	1.72
11cf47	28.5	28.7	0.2	38.1	-5.2	0
11cf48	28.7	29.9	1.2	43.4	5.3	2.6
11cf49	29.9	31.7	1.8	38.4	-5.0	1.5
11cf50	31.7	32	0.3	36.5	-1.9	0.18
11cf51	32	32.2	0.2	37.6	1.1	0
11cf52	32.2	32.5	0.3	35.9	-1.7	0.31
11cf53	32.5	32.7	0.2	37.9	2.1	0
11cf54	32.7	37	4.3	34.9	-3.1	1.1
11cf55	37	38.9	1.9	39.1	4.3	1.64
11cf56	38.9	39.2	0.3	36.6	-2.5	1.29
11cf57	39.2	39.5	0.3	45.3	8.8	1.98
11cf58	39.5	40.2	0.7	38.1	-7.2	1.4
11cf59	40.2	40.5	0.3	35.1	-3.1	1.15
11cf60	40.5	41.6	1.1	38.1	3.1	0.66
11cf61	41.6	41.8	0.2	41.4	3.3	0
11cf62	41.8	42.2	0.4	39.3	-2.1	1.96
11cf63	42.2	42.4	0.2	43.4	4.1	0
11cf64	42.4	43.7	1.3	39.1	-4.2	1.39
11cf65	43.7	43.9	0.2	42.5	3.3	0
11cf66	43.9	44.5	0.6	39.3	-3.2	1.23
11cf67	44.5	45.3	0.8	35.6	-3.7	0.97
11cf68	45.3	45.8	0.5	38.9	3.3	1.64
11cf69	45.8	47.8	2	35.2	-3.7	0.96
11cf70	47.8	48.1	0.3	39.0	3.8	0.57
11cf71	48.1	48.3	0.2	35.6	-3.4	0
11cf72	48.3	49.2	0.9	38.9	3.3	1.56
11cf73	49.2	52.6	3.4	35.3	-3.6	0.95
11cf74	52.6	53.2	0.6	39.8	4.5	1.28
11cf75	53.2	53.6	0.4	44.1	4.3	3.32
11cf76	53.6	53.8	0.2	39.1	-5.0	0
11cf77	53.8	54.2	0.4	42.5	3.5	1.71
11cf78	54.2	54.4	0.2	50.5	8.0	0
11cf79	54.4	55.2	0.8	41.6	-8.9	2.53
11cf80	55.2	55.4	0.2	48.8	7.2	0
11cf81	55.4	56.1	0.7	41.6	-7.1	2.54
11cf82	56.1	56.5	0.4	46.7	5.0	3.08
11cf83	56.5	56.7	0.2	35.4	-11.3	0
11cf84	56.7	56.9	0.2	46.0	10.6	0
11cf85	56.9	57.2	0.3	41.1	-4.8	1.52
11cf86	57.2	57.4	0.2	48.1	7.0	0
11cf87	57.4	58.3	0.9	43.7	-4.4	2.09
11cf88	58.3	59.2	0.9	47.7	4.0	0.74
11cf89	59.2	59.5	0.3	43.0	-4.7	2.24
11cf90	59.5	61.6	2.1	38.9	-4.1	1.44

11cf91	61.6	63.8	2.2	35.2	-3.7	1.29
11cf92	63.8	64	0.2	43.7	8.5	0
11cf93	64	65.4	1.4	38.9	-4.8	1.52
11cf94	65.4	66.6	1.2	43.8	4.9	1.98
11cf95	66.6	66.8	0.2	40.6	-3.3	0
11cf96	66.8	67.1	0.3	42.8	2.2	1.12
11cf97	67.1	67.3	0.2	39.5	-3.3	0
11cf98	67.3	68.3	1	44.6	5.1	2.24
11cf99	68.3	70.2	1.9	39.3	-5.3	1.79
11cf100	70.2	70.7	0.5	43.5	4.2	1.76
11cf101	70.7	71.3	0.6	49.3	5.8	3.71
11cf102	71.3	71.7	0.4	57.3	8.0	2.09
11cf103	71.7	71.9	0.2	50.5	-6.8	0
11cf104	71.9	72.3	0.4	43.6	-6.9	2.21
11cf105	72.3	73	0.7	39.7	-3.9	0.31
11cf106	73	73.3	0.3	47.7	8.1	1.19
11cf107	73.3	73.5	0.2	43.1	-4.7	0
11cf108	73.5	74.2	0.7	48.1	5.1	2.14
11cf109	74.2	74.7	0.5	41.5	-6.7	1.72
11cf110	74.7	75.5	0.8	38.9	-2.6	1.1
11cf111	75.5	76.2	0.7	46.8	7.9	2.63
11cf112	76.2	76.7	0.5	56.0	9.2	2.33
11cf113	76.7	77.5	0.8	47.8	-8.2	6.88
12cf1	0	3	3	0.0		0
12cf2	3	3.8	0.8	43.6		3.95
12cf3	3.8	4.7	0.9	49.9	6.2	3.36
12cf4	4.7	5.1	0.4	36.7	-13.2	0.39
12cf5	5.1	5.4	0.3	39.4	2.7	0.61
12cf6	5.4	5.6	0.2	42.6	3.1	0
12cf7	5.6	6	0.4	49.0	6.5	1.01
12cf8	6	6.5	0.5	53.2	4.2	0.44
12cf9	6.5	6.9	0.4	46.5	-6.7	4.81
12cf10	6.9	7.2	0.3	39.9	-6.6	0.77
12cf11	7.2	7.8	0.6	46.5	6.6	3.69
12cf12	7.8	8	0.2	43.1	-3.4	0
12cf13	8	8.2	0.2	39.7	-3.4	0
12cf14	8.2	8.7	0.5	43.6	3.9	2.36
12cf15	8.7	9.2	0.5	48.6	5.0	2.83
12cf16	9.2	9.5	0.3	42.8	-5.8	0.88
12cf17	9.5	9.8	0.3	50.2	7.4	0.1
12cf18	9.8	10.2	0.4	39.9	-10.3	5.32
12cf19	10.2	10.9	0.7	38.8	-1.0	0.92
12cf20	10.9	11.3	0.4	47.2	8.4	3.88
12cf21	11.3	11.6	0.3	39.1	-8.1	1.49
12cf22	11.6	12.1	0.5	42.8	3.7	2.09
12cf23	12.1	12.6	0.5	48.7	6.0	1.96
12cf24	12.6	13.1	0.5	44.2	-4.6	1.61
12cf25	13.1	13.8	0.7	49.2	5.1	4.31
12cf26	13.8	14.5	0.7	42.6	-6.6	3.46
12cf27	14.5	15.7	1.2	49.4	6.8	3.81
12cf28	15.7	16.2	0.5	44.3	-5.0	2.46
12cf29	16.2	16.7	0.5	35.1	-9.3	1.02
12cf30	16.7	17.5	0.8	42.6	7.5	1.61
12cf31	17.5	18.3	0.8	40.3	-2.3	1.37
12cf32	18.3	18.5	0.2	41.2	0.9	0

12cf33	18.5	19.1	0.6	37.4	-3.8	1.6
12cf34	19.1	21.4	2.3	35.8	-1.6	0.78
12cf35	21.4	22.7	1.3	38.4	2.6	1.18
12cf36	22.7	23.8	1.1	41.6	3.2	1.54
12cf37	23.8	24.5	0.7	39.5	-2.1	1.23
12cf38	24.5	26.5	2	35.6	-3.9	0.79
12cf39	26.5	28.1	1.6	37.7	2.1	1.03
12cf40	28.1	29.9	1.8	35.0	-2.7	0.93
12cf41	29.9	30.3	0.4	37.0	2.0	1.38
12cf42	30.3	36.1	5.8	34.6	-2.4	1.01
12cf43	36.1	36.6	0.5	37.4	2.8	1.47
12cf44	36.6	38.2	1.6	35.7	-1.7	1.16
12cf45	38.2	39.1	0.9	39.2	3.4	1.78
12cf46	39.1	39.6	0.5	35.6	-3.5	0.63
12cf47	39.6	40.1	0.5	37.8	2.2	1.15
12cf48	40.1	40.5	0.4	35.6	-2.2	0.94
12cf49	40.5	40.7	0.2	37.9	2.3	0
12cf50	40.7	42.7	2	35.8	-2.1	0.64
12cf51	42.7	42.9	0.2	37.1	1.3	0
12cf52	42.9	43.5	0.6	35.8	-1.3	1.74
12cf53	43.5	44.3	0.8	37.8	2.0	0.84
12cf54	44.3	45.8	1.5	35.7	-2.1	1.12
12cf55	45.8	46	0.2	37.2	1.4	0
12cf56	46	46.3	0.3	36.9	-0.3	0.12
12cf57	46.3	51	4.7	37.9	1.1	1.28
12cf58	51	51.2	0.2	35.3	-2.6	0
12cf59	51.2	51.4	0.2	38.7	3.4	0
12cf60	51.4	51.9	0.5	42.0	3.3	0.73
12cf61	51.9	52.7	0.8	40.1	-2.0	1.31
12cf62	52.7	53	0.3	36.6	-3.4	0.22
12cf63	53	53.4	0.4	36.9	0.3	0.79
12cf64	53.4	58.1	4.7	34.8	-2.1	1.01
12cf65	58.1	58.6	0.5	38.6	3.8	1.08
12cf66	58.6	59.8	1.2	35.1	-3.5	1.13
12cf67	59.8	60.4	0.6	39.6	4.5	0.72
12cf68	60.4	60.6	0.2	41.8	2.2	0
12cf69	60.6	61.6	1	38.6	-3.2	1.88
12cf70	61.6	64.8	3.2	34.4	-4.2	1.17
12cf71	64.8	66.2	1.4	38.9	4.5	1.91
12cf72	66.2	67.4	1.2	41.6	2.7	2.32
12cf73	67.4	67.7	0.3	38.2	-3.4	1.31
12cf74	67.7	68	0.3	44.8	6.6	2.65
12cf75	68	69	1	41.3	-3.6	3.41
12cf76	69	69.2	0.2	36.2	-5.0	0
12cf77	69.2	70	0.8	40.6	4.4	2.66
12cf78	70	70.8	0.8	41.7	1.1	2.6
12cf79	70.8	71	0.2	36.1	-5.6	0
12cf80	71	71.8	0.8	45.4	9.3	2.25
12cf81	71.8	72.1	0.3	38.6	-6.8	1.04
12cf82	72.1	72.5	0.4	35.4	-3.2	0.41
12cf83	72.5	73.6	1.1	42.9	7.5	2.09
12cf84	73.6	74.4	0.8	37.5	-5.4	0.92
12cf85	74.4	75.1	0.7	45.9	8.3	3.38
12cf86	75.1	75.3	0.2	64.3	18.4	0
12cf87	75.3	75.6	0.3	49.8	-14.5	0

13cf1	0	3	3	0.0		0
13cf2	3	3.7	0.7	42.2		1.15
13cf3	3.7	3.9	0.2	35.4	-6.8	0
13cf4	3.9	4.1	0.2	40.0	4.6	0
13cf5	4.1	5.6	1.5	37.7	-2.3	3.09
13cf6	5.6	6.4	0.8	42.7	4.9	1.54
13cf7	6.4	7.1	0.7	39.1	-3.6	1.78
13cf8	7.1	7.7	0.6	43.6	4.6	1.89
13cf9	7.7	8	0.3	39.6	-4.1	1.57
13cf10	8	8.5	0.5	34.1	-5.5	1.66
13cf11	8.5	8.7	0.2	42.0	7.9	0
13cf12	8.7	9.1	0.4	38.4	-3.6	1.65
13cf13	9.1	11.5	2.4	36.1	-2.3	0.89
13cf14	11.5	11.8	0.3	38.1	1.9	0.77
13cf15	11.8	12.5	0.7	36.1	-2.0	0.95
13cf16	12.5	12.8	0.3	38.0	1.8	0.51
13cf17	12.8	13.1	0.3	36.0	-1.9	0.6
13cf18	13.1	13.4	0.3	38.8	2.7	1.49
13cf19	13.4	18.7	5.3	34.4	-4.4	1.03
13cf20	18.7	20.2	1.5	38.4	4.0	1.01
13cf21	20.2	20.6	0.4	42.3	3.9	0.82
13cf22	20.6	22.5	1.9	38.8	-3.4	1.25
13cf23	22.5	22.7	0.2	41.1	2.2	0
13cf24	22.7	23.6	0.9	38.2	-2.8	0.89
13cf25	23.6	23.9	0.3	36.4	-1.8	0.54
13cf26	23.9	24.1	0.2	39.0	2.6	0
13cf27	24.1	24.3	0.2	44.1	5.0	0
13cf28	24.3	24.5	0.2	48.0	3.9	0
13cf29	24.5	25.4	0.9	42.1	-5.9	1.95
13cf30	25.4	25.6	0.2	39.2	-2.9	0
13cf31	25.6	26.1	0.5	43.9	4.7	1.79
13cf32	26.1	26.3	0.2	39.2	-4.7	0
13cf33	26.3	26.7	0.4	46.5	7.3	3.23
13cf34	26.7	28	1.3	39.4	-7.1	1.41
13cf35	28	28.3	0.3	43.9	4.5	1.89
13cf36	28.3	28.6	0.3	50.0	6.1	2.4
13cf37	28.6	29.8	1.2	39.6	-10.3	0.97
13cf38	29.8	30.4	0.6	44.0	4.3	3.2
13cf39	30.4	30.6	0.2	46.9	2.9	0
13cf40	30.6	31	0.4	43.4	-3.5	0.88
13cf41	31	31.5	0.5	39.7	-3.6	0.78
13cf42	31.5	32.4	0.9	43.1	3.4	3.36
13cf43	32.4	33.1	0.7	50.4	7.3	2.21
13cf44	33.1	33.6	0.5	43.2	-7.2	2.01
13cf45	33.6	34.1	0.5	47.7	4.5	4.54
13cf46	34.1	34.9	0.8	43.5	-4.2	2.15
13cf47	34.9	36.2	1.3	38.8	-4.8	1.2
13cf48	36.2	36.5	0.3	43.1	4.4	2.42
13cf49	36.5	37.1	0.6	49.2	6.1	2.76
13cf50	37.1	37.5	0.4	44.4	-4.8	1
13cf51	37.5	37.8	0.3	57.1	12.6	3.1
13cf52	37.8	38.4	0.6	47.6	-9.4	2.69
13cf53	38.4	38.6	0.2	43.0	-4.6	0
13cf54	38.6	41	2.4	60.6	17.7	3.37
13cf55	41	42.4	1.4	49.4	-11.2	3.76
13cf56	42.4	43	0.6	42.7	-6.7	1.83

13cf57	43	43.5	0.5	38.6	-4.1	1.69
13cf58	43.5	43.8	0.3	36.5	-2.1	0.76
13cf59	43.8	44.3	0.5	39.1	2.6	1.22
13cf60	44.3	45.2	0.9	35.5	-3.6	0.92
13cf61	45.2	45.5	0.3	38.2	2.7	1
13cf62	45.5	45.8	0.3	36.1	-2.1	0.44
13cf63	45.8	46	0.2	38.1	1.9	0
13cf64	46	46.2	0.2	36.3	-1.8	0
13cf65	46.2	46.8	0.6	40.6	4.3	1.64
13cf66	46.8	47	0.2	41.5	1.0	0
13cf67	47	47.5	0.5	39.0	-2.5	2.4
13cf68	47.5	48.4	0.9	43.1	4.1	1.4
13cf69	48.4	48.7	0.3	47.1	4.0	3.27
13cf70	48.7	49	0.3	37.3	-9.8	0.68
13cf71	49	50.3	1.3	43.2	5.9	1.44
13cf72	50.3	51.7	1.4	38.9	-4.3	1.73
13cf73	51.7	51.9	0.2	41.6	2.8	0
13cf74	51.9	52.5	0.6	40.2	-1.4	1.89
13cf75	52.5	61.7	9.2	34.8	-5.4	1.03
13cf76	61.7	62	0.3	38.0	3.2	0.77
13cf77	62	62.8	0.8	35.6	-2.4	0.72
13cf78	62.8	63.8	1	38.1	2.5	0.97
13cf79	63.8	64.1	0.3	35.8	-2.2	0.97
13cf80	64.1	64.8	0.7	37.9	2.1	0.83
13cf81	64.8	65.1	0.3	35.5	-2.4	0.88
13cf82	65.1	66.2	1.1	39.0	3.4	0.77
14cf1	0	3	3	0.0		0
14cf2	3	3.3	0.3	40.8		1.05
14cf3	3.3	3.6	0.3	47.9	7.1	4.11
14cf4	3.6	3.9	0.3	56.0	8.1	0.71
14cf5	3.9	4.1	0.2	49.6	-6.4	0
14cf6	4.1	5.9	1.8	38.2	-11.4	1.07
14cf7	5.9	6.4	0.5	42.7	4.5	2.2
14cf8	6.4	7.4	1	39.2	-3.5	1.65
14cf9	7.4	8.6	1.2	47.2	8.1	3.03
14cf10	8.6	9	0.4	38.7	-8.5	1.94
14cf11	9	9.2	0.2	42.1	3.4	0
14cf12	9.2	9.5	0.3	40.0	-2.1	0.83
14cf13	9.5	10.3	0.8	42.7	2.7	3.21
14cf14	10.3	10.7	0.4	40.6	-2.1	1.55
14cf15	10.7	11.4	0.7	47.5	6.9	4.44
14cf16	11.4	12.2	0.8	39.1	-8.4	2.33
14cf17	12.2	14.5	2.3	35.6	-3.5	0.95
14cf18	14.5	15.1	0.6	37.9	2.3	0.82
14cf19	15.1	15.8	0.7	34.6	-3.3	0.67
14cf20	15.8	16.7	0.9	38.4	3.8	1.16
14cf21	16.7	17.2	0.5	36.2	-2.2	1.06
14cf22	17.2	17.5	0.3	38.2	2.0	0.42
14cf23	17.5	18.9	1.4	35.9	-2.3	0.44
14cf24	18.9	20.5	1.6	38.0	2.1	0.95
14cf25	20.5	20.9	0.4	36.0	-2.0	1.1
14cf26	20.9	21.9	1	38.1	2.1	1.54
14cf27	21.9	22.7	0.8	36.4	-1.7	0.87
14cf28	22.7	23.1	0.4	37.4	1.1	1.42
14cf29	23.1	23.5	0.4	35.8	-1.6	0.24

14cf30	23.5	24.5	1	39.3	3.6	1.01
14cf31	24.5	24.8	0.3	36.5	-2.8	0.56
14cf32	24.8	27	2.2	38.2	1.7	1.6
14cf33	27	30.4	3.4	35.1	-3.1	1.25
14cf34	30.4	30.6	0.2	37.8	2.7	0
14cf35	30.6	33.9	3.3	35.0	-2.8	0.92
14cf36	33.9	34.3	0.4	37.7	2.7	0.56
14cf37	34.3	34.9	0.6	36.2	-1.5	0.57
14cf38	34.9	35.1	0.2	37.9	1.6	0
14cf39	35.1	37.7	2.6	35.6	-2.3	0.96
14cf40	37.7	38.7	1	38.8	3.2	1.07
14cf41	38.7	39	0.3	42.3	3.4	1.04
14cf42	39	41	2	39.3	-2.9	1.48
14cf43	41	41.2	0.2	36.8	-2.6	0
14cf44	41.2	41.5	0.3	41.5	4.8	0.42
14cf45	41.5	41.9	0.4	39.9	-1.7	0.7
14cf46	41.9	42.9	1	42.6	2.7	0.5
14cf47	42.9	43.2	0.3	37.1	-5.5	0.78
14cf48	43.2	43.4	0.2	50.3	13.2	0
14cf49	43.4	43.9	0.5	38.8	-11.5	1.28
14cf50	43.9	44.1	0.2	42.8	4.0	0
14cf51	44.1	44.7	0.6	40.4	-2.4	0.36
14cf52	44.7	45	0.3	41.7	1.3	0.9
14cf53	45	45.2	0.2	40.1	-1.6	0
14cf54	45.2	46.3	1.1	41.9	1.8	2.64
14cf55	46.3	46.7	0.4	52.5	10.6	2.25
14cf56	46.7	47.2	0.5	40.5	-12.0	1.92
14cf57	47.2	48.2	1	40.1	-0.4	1.5
14cf58	48.2	48.7	0.5	36.5	-3.6	1.7
14cf59	48.7	48.9	0.2	42.6	6.1	0
14cf60	48.9	49.9	1	39.5	-3.2	1.92
14cf61	49.9	50.2	0.3	42.8	3.3	1.31
14cf62	50.2	50.5	0.3	39.2	-3.6	1.35
14cf63	50.5	50.7	0.2	46.9	7.7	0
14cf64	50.7	51.7	1	41.2	-5.8	1.3
14cf65	51.7	52.1	0.4	38.3	-2.9	0.68
14cf66	52.1	54.3	2.2	35.8	-2.5	1
14cf67	54.3	55.2	0.9	40.3	4.5	1.71
14cf68	55.2	55.4	0.2	36.5	-3.8	0
14cf69	55.4	55.6	0.2	37.7	1.3	0
14cf70	55.6	56.8	1.2	35.4	-2.3	1.24
14cf71	56.8	57.5	0.7	38.2	2.8	0.56
14cf72	57.5	58.1	0.6	36.1	-2.1	1.01
14cf73	58.1	59.4	1.3	40.0	4.0	2.55
14cf74	59.4	59.8	0.4	42.6	2.5	1.32
14cf75	59.8	60	0.2	39.4	-3.2	0
14cf76	60	61.2	1.2	35.4	-4.0	0.66
14cf77	61.2	61.8	0.6	38.6	3.2	0.95
14cf78	61.8	62.3	0.5	43.0	4.5	1.78
14cf79	62.3	62.5	0.2	48.0	5.0	0
14cf80	62.5	63.4	0.9	42.6	-5.4	1.96
14cf81	63.4	63.6	0.2	46.8	4.2	0
14cf82	63.6	63.8	0.2	43.8	-3.0	0
14cf83	63.8	64	0.2	38.0	-5.7	0
15cf1	0	3	3	0.0		0

15cf2	3	3.7	0.7	44.5		4.27
15cf3	3.7	4.1	0.4	40.9	-3.5	3.23
15cf4	4.1	4.3	0.2	44.8	3.9	0
15cf5	4.3	4.6	0.3	51.5	6.6	1.4
15cf6	4.6	4.8	0.2	57.5	6.0	0
15cf7	4.8	5.4	0.6	46.8	-10.7	5.49
15cf8	5.4	5.8	0.4	41.2	-5.6	2.74
15cf9	5.8	6.1	0.3	44.0	2.8	1.46
15cf10	6.1	6.4	0.3	50.7	6.7	1.58
15cf11	6.4	6.9	0.5	42.5	-8.1	1.44
15cf12	6.9	8	1.1	49.3	6.7	3.5
15cf13	8	8.8	0.8	54.4	5.2	2.06
15cf14	8.8	9.1	0.3	51.4	-3.0	1.05
15cf15	9.1	9.4	0.3	42.1	-9.3	3.08
15cf16	9.4	9.6	0.2	47.9	5.8	0
15cf17	9.6	10.1	0.5	41.4	-6.5	4.3
15cf18	10.1	10.3	0.2	55.8	14.4	0
15cf19	10.3	10.7	0.4	48.5	-7.3	2.5
15cf20	10.7	11.3	0.6	54.9	6.4	2.67
15cf21	11.3	11.8	0.5	43.9	-11.0	3.5
15cf22	11.8	13.3	1.5	39.2	-4.7	2.47
15cf23	13.3	13.7	0.4	35.7	-3.5	1.34
15cf24	13.7	14	0.3	44.9	9.2	2.58
15cf25	14	14.8	0.8	36.5	-8.4	1.27
15cf26	14.8	15.1	0.3	39.3	2.8	1.23
15cf27	15.1	15.8	0.7	46.3	7.0	5.72
15cf28	15.8	16	0.2	55.7	9.3	0
15cf29	16	17.2	1.2	48.1	-7.6	5.03
15cf30	17.2	17.4	0.2	41.6	-6.4	0
15cf31	17.4	17.7	0.3	38.9	-2.7	1.4
15cf32	17.7	18.1	0.4	42.4	3.5	2.37
15cf33	18.1	18.3	0.2	37.6	-4.8	0
15cf34	18.3	18.5	0.2	42.1	4.5	0
15cf35	18.5	18.8	0.3	50.4	8.3	0.64
15cf36	18.8	20.1	1.3	48.1	-2.3	4.87
15cf37	20.1	20.6	0.5	38.8	-9.3	0.42
15cf38	20.6	21.2	0.6	49.4	10.6	5
15cf39	21.2	21.7	0.5	42.9	-6.5	1.93
15cf40	21.7	22	0.3	39.6	-3.4	0.46
15cf41	22	22.3	0.3	43.4	3.8	1.14
15cf42	22.3	23.4	1.1	39.0	-4.4	1.89
15cf43	23.4	24.8	1.4	36.4	-2.6	1.1
15cf44	24.8	25.7	0.9	37.8	1.4	2.09
15cf45	25.7	26	0.3	34.8	-3.0	1.04
15cf46	26	26.2	0.2	39.1	4.3	0
15cf47	26.2	27.5	1.3	35.9	-3.2	0.96
15cf48	27.5	28.1	0.6	38.2	2.3	1.67
15cf49	28.1	32.5	4.4	34.8	-3.4	0.99
15cf50	32.5	32.7	0.2	38.0	3.2	0
15cf51	32.7	33	0.3	36.0	-2.0	0.82
15cf52	33	34.1	1.1	39.5	3.5	2.11
15cf53	34.1	35.4	1.3	36.2	-3.3	1.28
15cf54	35.4	37.1	1.7	39.8	3.6	1.44
15cf55	37.1	37.4	0.3	44.7	4.9	2.41
15cf56	37.4	37.6	0.2	38.4	-6.3	0
15cf57	37.6	38	0.4	43.0	4.6	1.11

15cf58	38	38.7	0.7	37.9	-5.1	0.53
15cf59	38.7	39	0.3	43.6	5.7	3.13
15cf60	39	41.1	2.1	39.0	-4.7	1.2
15cf61	41.1	41.3	0.2	43.6	4.6	0
15cf62	41.3	41.5	0.2	39.9	-3.7	0
15cf63	41.5	42.3	0.8	36.7	-3.2	1.45
15cf64	42.3	43.7	1.4	39.2	2.5	1.08
15cf65	43.7	43.9	0.2	42.1	3.0	0
15cf66	43.9	44.2	0.3	36.3	-5.8	0.27
15cf67	44.2	45.1	0.9	40.3	4.0	1.2
15cf68	45.1	45.8	0.7	45.5	5.1	4.49
15cf69	45.8	47.3	1.5	38.4	-7.1	1.13
15cf70	47.3	47.8	0.5	41.5	3.2	1.19
15cf71	47.8	48.4	0.6	36.9	-4.6	1.3
15cf72	48.4	49.8	1.4	38.4	1.5	1.33
15cf73	49.8	50.9	1.1	36.0	-2.4	0.69
15cf74	50.9	51.3	0.4	39.1	3.1	2.07
15cf75	51.3	52	0.7	34.1	-5.0	0.95
15cf76	52	52.5	0.5	39.4	5.3	1.09
15cf77	52.5	53.2	0.7	43.2	3.9	1.14
15cf78	53.2	53.4	0.2	33.9	-9.3	0
15cf79	53.4	53.8	0.4	40.6	6.6	2.51
15cf80	53.8	54.1	0.3	44.5	3.9	2.01
15cf81	54.1	54.3	0.2	40.0	-4.5	0
15cf82	54.3	54.6	0.3	42.2	2.2	1.06
15cf83	54.6	55.7	1.1	38.8	-3.4	1.07
15cf84	55.7	56.1	0.4	36.7	-2.1	0.37
15cf85	56.1	56.5	0.4	37.4	0.7	0.81
15cf86	56.5	57	0.5	35.0	-2.5	0.64
15cf87	57	57.4	0.4	37.6	2.6	1.33
15cf88	57.4	58.4	1	35.1	-2.5	0.76
15cf89	58.4	59.1	0.7	38.9	3.9	1.36
15cf90	59.1	62.3	3.2	35.0	-3.9	1.31
15cf91	62.3	63	0.7	38.9	3.9	1.13
15cf92	63	63.5	0.5	35.9	-3.0	0.59
15cf93	63.5	64	0.5	38.3	2.4	0.9
15cf94	64	64.5	0.5	42.3	4.0	0.58
15cf95	64.5	65	0.5	39.2	-3.1	1.07
15cf96	65	65.6	0.6	35.9	-3.3	0.79
15cf97	65.6	66.1	0.5	38.3	2.5	1.15
15cf98	66.1	66.7	0.6	37.7	-0.6	3.13
15cf99	66.7	66.9	0.2	43.3	5.6	0
15cf100	66.9	67.1	0.2	49.5	6.2	0
15cf101	67.1	67.3	0.2	42.0	-7.5	0
16cf1	0	3	3	0.0		0
16cf2	3	3.5	0.5	56.3		5.55
16cf3	3.5	4	0.5	50.0	-6.3	2.17
16cf4	4	4.3	0.3	53.9	3.9	4.94
16cf5	4.3	4.5	0.2	44.6	-9.3	0
16cf6	4.5	4.7	0.2	39.4	-5.2	0
16cf7	4.7	5.5	0.8	44.2	4.8	2.18
16cf8	5.5	6.2	0.7	38.6	-5.6	0.8
16cf9	6.2	6.8	0.6	36.2	-2.4	0.52
16cf10	6.8	7	0.2	37.4	1.1	0
16cf11	7	7.9	0.9	35.3	-2.1	0.81

16cf12	7.9	9	1.1	38.6	3.4	2.29
16cf13	9	9.2	0.2	42.1	3.5	0
16cf14	9.2	9.4	0.2	47.2	5.0	0
16cf15	9.4	9.6	0.2	39.6	-7.6	0
16cf16	9.6	10	0.4	45.7	6.1	3.5
16cf17	10	10.4	0.4	39.4	-6.2	0.72
16cf18	10.4	10.6	0.2	42.8	3.4	0
16cf19	10.6	10.8	0.2	46.3	3.5	0
16cf20	10.8	11.1	0.3	44.2	-2.1	1.91
16cf21	11.1	11.3	0.2	37.2	-7.0	0
16cf22	11.3	12.1	0.8	44.9	7.7	2.35
16cf23	12.1	12.3	0.2	48.1	3.3	0
16cf24	12.3	12.5	0.2	38.6	-9.5	0
16cf25	12.5	13	0.5	42.5	3.8	0.99
16cf26	13	13.3	0.3	38.5	-4.0	1.36
16cf27	13.3	13.6	0.3	50.6	12.2	5.56
16cf28	13.6	13.9	0.3	42.2	-8.4	0.88
16cf29	13.9	15.3	1.4	38.8	-3.4	1.03
16cf30	15.3	15.8	0.5	41.4	2.6	4.67
16cf31	15.8	16	0.2	46.1	4.7	0
16cf32	16	16.3	0.3	40.3	-5.8	0.16
16cf33	16.3	17.2	0.9	43.2	2.9	2.92
16cf34	17.2	17.7	0.5	50.4	7.2	3.17
16cf35	17.7	17.9	0.2	44.7	-5.8	0
16cf36	17.9	18.2	0.3	54.5	9.8	2.77
16cf37	18.2	18.8	0.6	48.3	-6.2	3.23
16cf38	18.8	19.5	0.7	40.3	-8.0	2.66
16cf39	19.5	20.8	1.3	44.7	4.4	1.93
16cf40	20.8	21.2	0.4	48.4	3.7	2.42
16cf41	21.2	21.4	0.2	54.0	5.6	0
16cf42	21.4	22.2	0.8	48.0	-6.0	3.51
16cf43	22.2	22.4	0.2	38.4	-9.6	0
16cf44	22.4	22.8	0.4	47.2	8.7	3.11
16cf45	22.8	23.1	0.3	55.3	8.1	1.64
16cf46	23.1	23.6	0.5	50.6	-4.7	1.93
16cf47	23.6	23.8	0.2	42.9	-7.7	0
16cf48	23.8	24.3	0.5	45.5	2.6	3.94
16cf49	24.3	25.1	0.8	38.2	-7.3	1.41
16cf50	25.1	25.7	0.6	42.2	4.0	1.99
16cf51	25.7	26.3	0.6	39.6	-2.6	1.62
16cf52	26.3	26.5	0.2	43.8	4.2	0
16cf53	26.5	26.7	0.2	38.3	-5.5	0
16cf54	26.7	27	0.3	48.0	9.6	0.6
16cf55	27	27.2	0.2	39.5	-8.5	0
16cf56	27.2	27.6	0.4	43.9	4.4	2.45
16cf57	27.6	28.3	0.7	38.6	-5.3	1.03
16cf58	28.3	29.5	1.2	35.3	-3.2	1.18
16cf59	29.5	30.5	1	42.9	7.6	2.16
16cf60	30.5	30.8	0.3	48.2	5.4	1.76
16cf61	30.8	31.2	0.4	43.8	-4.4	1.27
16cf62	31.2	32.7	1.5	38.4	-5.4	1.01
16cf63	32.7	32.9	0.2	36.2	-2.2	0
16cf64	32.9	33.3	0.4	37.3	1.1	0.06
16cf65	33.3	33.6	0.3	36.4	-0.9	0.16
16cf66	33.6	34	0.4	39.3	2.8	1.38
16cf67	34	34.2	0.2	43.8	4.5	0

16cf68	34.2	35.3	1.1	38.2	-5.6	0.92
16cf69	35.3	35.9	0.6	36.4	-1.8	0.78
16cf70	35.9	36.1	0.2	38.2	1.8	0
16cf71	36.1	36.3	0.2	35.2	-3.0	0
16cf72	36.3	36.9	0.6	40.4	5.2	2.21
16cf73	36.9	37.1	0.2	41.7	1.3	0
16cf74	37.1	37.3	0.2	40.6	-1.1	0
16cf75	37.3	37.8	0.5	43.1	2.6	1.92
16cf76	37.8	38	0.2	35.2	-7.9	0
16cf77	38	39.1	1.1	42.1	6.9	1.6
16cf78	39.1	39.7	0.6	39.9	-2.2	1.61
16cf79	39.7	42.9	3.2	35.2	-4.7	1.2
16cf80	42.9	43.1	0.2	38.5	3.3	0
16cf81	43.1	43.6	0.5	35.8	-2.7	0.75
16cf82	43.6	44.7	1.1	38.5	2.6	1.61
16cf83	44.7	46.4	1.7	35.5	-3.0	1.2
16cf84	46.4	48.5	2.1	38.4	2.9	1.28
16cf85	48.5	48.8	0.3	44.5	6.1	2.23
16cf86	48.8	49	0.2	40.0	-4.5	0
16cf87	49	50	1	42.9	2.9	1.55
16cf88	50	50.2	0.2	40.7	-2.2	0
16cf89	50.2	50.5	0.3	45.2	4.5	4.11
16cf90	50.5	51.2	0.7	38.8	-6.4	0.62
16cf91	51.2	51.4	0.2	36.7	-2.1	0
16cf92	51.4	51.6	0.2	38.9	2.1	0
16cf93	51.6	51.8	0.2	36.2	-2.6	0
16cf94	51.8	52	0.2	37.8	1.6	0
16cf95	52	55.2	3.2	35.8	-2.0	1.45
16cf96	55.2	55.4	0.2	43.9	8.0	0
16cf97	55.4	56.6	1.2	38.6	-5.3	1.92
16cf98	56.6	56.9	0.3	37.5	-1.0	1.72
16cf99	56.9	57.1	0.2	45.2	7.7	0
16cf100	57.1	57.4	0.3	57.1	11.9	4.63
16cf101	57.4	58.3	0.9	45.5	-11.6	2.59
16cf102	58.3	58.5	0.2	47.8	2.3	0
16cf103	58.5	58.8	0.3	42.3	-5.5	2.71
16cf104	58.8	59	0.2	39.3	-3.0	0
16cf105	59	59.2	0.2	42.1	2.9	0
16cf106	59.2	60.9	1.7	39.5	-2.6	1.77
16cf107	60.9	61.5	0.6	44.7	5.2	2.13
16cf108	61.5	61.7	0.2	38.1	-6.6	0
16cf109	61.7	61.9	0.2	49.7	11.5	0
16cf110	61.9	62.2	0.3	40.7	-8.9	7.59
16cf111	62.2	62.6	0.4	47.0	6.2	1.54
17cf1	0	3	3	0.0		0
17cf2	3	3.3	0.3	49.0		2.13
17cf3	3.3	4.1	0.8	57.6	8.6	3.46
17cf4	4.1	4.4	0.3	49.0	-8.7	3.78
17cf5	4.4	4.7	0.3	57.8	8.8	4.34
17cf6	4.7	5.4	0.7	51.6	-6.2	2.94
17cf7	5.4	5.7	0.3	42.5	-9.1	1.92
17cf8	5.7	5.9	0.2	40.4	-2.2	0
17cf9	5.9	7	1.1	44.0	3.6	2.55
17cf10	7	7.3	0.3	40.7	-3.3	0.33
17cf11	7.3	7.7	0.4	47.4	6.7	4.16

17cf12	7.7	7.9	0.2	43.1	-4.3	0
17cf13	7.9	8.1	0.2	40.2	-2.8	0
17cf14	8.1	8.6	0.5	43.5	3.2	1.71
17cf15	8.6	8.9	0.3	50.6	7.2	4.24
17cf16	8.9	10	1.1	44.1	-6.5	3.17
17cf17	10	10.9	0.9	50.3	6.2	3
17cf18	10.9	11.2	0.3	40.5	-9.7	5.56
17cf19	11.2	11.5	0.3	49.1	8.6	1.1
17cf20	11.5	11.8	0.3	42.8	-6.4	1.44
17cf21	11.8	12	0.2	40.0	-2.7	0
17cf22	12	12.4	0.4	43.3	3.3	1.42
17cf23	12.4	13.3	0.9	36.5	-6.8	0.85
17cf24	13.3	14.1	0.8	41.4	4.9	2.97
17cf25	14.1	14.4	0.3	38.3	-3.1	1.53
17cf26	14.4	14.9	0.5	48.3	10.0	2.26
17cf27	14.9	15.4	0.5	43.8	-4.5	1.44
17cf28	15.4	16	0.6	37.9	-5.9	0.71
17cf29	16	16.5	0.5	41.3	3.5	2.84
17cf30	16.5	17.6	1.1	39.4	-1.9	2.02
17cf31	17.6	18.9	1.3	45.0	5.6	4.01
17cf32	18.9	19.1	0.2	36.7	-8.3	0
17cf33	19.1	19.6	0.5	38.1	1.4	1.38
17cf34	19.6	20.3	0.7	36.3	-1.7	0.64
17cf35	20.3	20.6	0.3	38.9	2.5	1.74
17cf36	20.6	21	0.4	44.2	5.4	1.03
17cf37	21	21.2	0.2	51.4	7.1	0
17cf38	21.2	21.6	0.4	41.1	-10.3	3.49
17cf39	21.6	21.9	0.3	39.2	-1.9	0.8
17cf40	21.9	22.1	0.2	36.0	-3.2	0
17cf41	22.1	22.7	0.6	46.8	10.8	4.28
17cf42	22.7	23	0.3	38.8	-8.0	1.91
17cf43	23	23.3	0.3	43.2	4.4	0.41
17cf44	23.3	23.5	0.2	40.8	-2.4	0
17cf45	23.5	23.8	0.3	50.7	9.9	2.3
17cf46	23.8	25	1.2	45.1	-5.5	3.02
17cf47	25	25.3	0.3	38.3	-6.9	1.69
17cf48	25.3	25.7	0.4	47.1	8.9	3.58
17cf49	25.7	26.1	0.4	41.5	-5.7	4.19
17cf50	26.1	27.4	1.3	42.4	0.9	2.69
17cf51	27.4	27.6	0.2	45.0	2.6	0
17cf52	27.6	28.1	0.5	47.3	2.2	1.59
17cf53	28.1	28.7	0.6	37.5	-9.7	1.62
17cf54	28.7	28.9	0.2	35.4	-2.1	0
17cf55	28.9	30	1.1	39.2	3.8	1.51
17cf56	30	31.7	1.7	35.4	-3.8	0.82
17cf57	31.7	32.4	0.7	39.8	4.3	1.55
17cf58	32.4	32.6	0.2	36.0	-3.7	0
17cf59	32.6	33	0.4	38.6	2.5	1.25
17cf60	33	33.3	0.3	42.1	3.5	0.46
17cf61	33.3	34.3	1	39.7	-2.4	2.58
17cf62	34.3	34.6	0.3	37.0	-2.7	1.74
17cf63	34.6	34.8	0.2	41.6	4.6	0
17cf64	34.8	35.5	0.7	38.5	-3.1	0.64
17cf65	35.5	36.4	0.9	35.2	-3.3	0.68
17cf66	36.4	36.6	0.2	38.6	3.4	0
17cf67	36.6	36.8	0.2	46.0	7.4	0

17cf68	36.8	37.2	0.4	40.1	-5.9	2.69
17cf69	37.2	37.4	0.2	39.4	-0.7	0
17cf70	37.4	38.2	0.8	47.4	8.0	2.18
17cf71	38.2	38.9	0.7	42.0	-5.4	1.88
17cf72	38.9	39.1	0.2	46.7	4.6	0
17cf73	39.1	40.3	1.2	42.0	-4.6	2.62
17cf74	40.3	40.5	0.2	49.0	7.0	0
17cf75	40.5	41.5	1	42.5	-6.5	1.77
17cf76	41.5	41.8	0.3	37.6	-4.9	0.61
17cf77	41.8	42.3	0.5	43.0	5.4	2.14
17cf78	42.3	42.9	0.6	47.8	4.7	1.54
17cf79	42.9	43.2	0.3	43.7	-4.0	2.34
17cf80	43.2	44	0.8	39.0	-4.7	1.36
17cf81	44	46.6	2.6	35.0	-4.1	0.62
17cf82	46.6	47.4	0.8	38.5	3.6	0.92
17cf83	47.4	47.8	0.4	36.0	-2.5	0.58
17cf84	47.8	48	0.2	38.0	1.9	0
17cf85	48	50.5	2.5	34.8	-3.1	0.99
17cf86	50.5	51.2	0.7	39.4	4.6	2.03
17cf87	51.2	52.8	1.6	43.7	4.3	3.46
17cf88	52.8	53.1	0.3	47.1	3.4	5.09
17cf89	53.1	53.5	0.4	35.5	-11.7	0.44
17cf90	53.5	53.7	0.2	38.9	3.4	0
17cf91	53.7	54.3	0.6	48.3	9.4	1.97
17cf92	54.3	54.8	0.5	39.3	-9.0	2.58
17cf93	54.8	55.1	0.3	45.2	6.0	3.15
17cf94	55.1	55.8	0.7	38.0	-7.3	2.39
17cf95	55.8	57	1.2	43.6	5.6	1.32
17cf96	57	57.3	0.3	46.8	3.1	0.76
17cf97	57.3	58.1	0.8	42.8	-4.0	3.78
17cf98	58.1	58.7	0.6	38.4	-4.3	1.06
17cf99	58.7	59.5	0.8	38.8	0.4	3.12
17cf100	59.5	60	0.5	43.3	4.4	1.31
17cf101	60	60.4	0.4	39.7	-3.6	0.92
17cf102	60.4	60.7	0.3	45.5	5.8	3.67
17cf103	60.7	60.9	0.2	39.0	-6.5	0
17cf104	60.9	61.1	0.2	36.8	-2.1	0
17cf105	61.1	61.4	0.3	42.7	5.9	1.13
17cf106	61.4	61.7	0.3	40.1	-2.6	0.76
17cf107	61.7	62.3	0.6	44.5	4.4	2.33
17cf108	62.3	62.5	0.2	40.4	-4.1	0
17cf109	62.5	62.9	0.4	42.6	2.2	1.91
17cf110	62.9	63.1	0.2	37.9	-4.7	0
17cf111	63.1	63.8	0.7	44.3	6.4	2.68
17cf112	63.8	64	0.2	47.9	3.6	0
17cf113	64	65.6	1.6	41.5	-6.4	1.87
17cf114	65.6	66.5	0.9	42.8	1.3	3.35
17cf115	66.5	66.8	0.3	52.1	9.4	0.52
17cf116	66.8	67.4	0.6	41.6	-10.5	2.45
18cf1	0	3	3	0.0		0
18cf2	3	3.3	0.3	54.0		3.27
18cf3	3.3	3.6	0.3	48.3	-5.7	2.35
18cf4	3.6	4.1	0.5	41.3	-7.0	2.07
18cf5	4.1	4.5	0.4	43.3	2.0	3.53
18cf6	4.5	4.7	0.2	49.2	5.9	0

18cf7	4.7	5	0.3	56.0	6.8	2.62
18cf8	5	5.7	0.7	48.8	-7.2	3.69
18cf9	5.7	6.4	0.7	38.6	-10.3	1.6
18cf10	6.4	8.3	1.9	35.6	-2.9	1.15
18cf11	8.3	8.8	0.5	37.6	2.0	0.85
18cf12	8.8	9.1	0.3	45.9	8.3	1.1
18cf13	9.1	9.4	0.3	41.7	-4.3	2.62
18cf14	9.4	10.4	1	40.3	-1.4	0.6
18cf15	10.4	10.8	0.4	41.3	1.0	1.11
18cf16	10.8	11.1	0.3	47.6	6.3	0.99
18cf17	11.1	11.3	0.2	42.4	-5.2	0
18cf18	11.3	12.9	1.6	39.0	-3.4	1.77
18cf19	12.9	13.5	0.6	42.2	3.1	1.3
18cf20	13.5	15	1.5	39.9	-2.3	1.74
18cf21	15	15.8	0.8	42.7	2.8	1.36
18cf22	15.8	16.1	0.3	39.7	-2.9	1.79
18cf23	16.1	16.3	0.2	35.2	-4.5	0
18cf24	16.3	16.7	0.4	42.3	7.1	1.72
18cf25	16.7	17.4	0.7	38.8	-3.5	1.19
18cf26	17.4	18	0.6	46.0	7.3	3.39
18cf27	18	18.3	0.3	39.5	-6.6	0.15
18cf28	18.3	18.6	0.3	43.5	4.0	0.92
18cf29	18.6	20.9	2.3	38.9	-4.6	1.87
18cf30	20.9	21.1	0.2	41.9	3.0	0
18cf31	21.1	21.3	0.2	38.4	-3.6	0
18cf32	21.3	24.8	3.5	35.8	-2.5	1.06
18cf33	24.8	25.2	0.4	37.8	2.0	0.56
18cf34	25.2	25.7	0.5	35.9	-1.9	0.87
18cf35	25.7	25.9	0.2	39.0	3.1	0
18cf36	25.9	26.1	0.2	36.6	-2.5	0
18cf37	26.1	26.4	0.3	37.7	1.2	0.6
18cf38	26.4	27.4	1	36.3	-1.5	1.3
18cf39	27.4	27.7	0.3	37.8	1.5	0.55
18cf40	27.7	28	0.3	36.4	-1.5	0.98
18cf41	28	28.2	0.2	38.3	2.0	0
18cf42	28.2	28.4	0.2	47.0	8.6	0
18cf43	28.4	28.9	0.5	60.2	13.2	3.66
18cf44	28.9	29.6	0.7	42.8	-17.4	1.37
18cf45	29.6	31.9	2.3	40.0	-2.8	1.64
18cf46	31.9	34.2	2.3	36.0	-4.0	0.75
18cf47	34.2	34.7	0.5	40.7	4.7	2.84
18cf48	34.7	35.9	1.2	47.6	6.9	3.06
18cf49	35.9	36.1	0.2	39.9	-7.7	0
18cf50	36.1	37.1	1	46.1	6.2	4.42
18cf51	37.1	37.3	0.2	42.8	-3.3	0
18cf52	37.3	37.8	0.5	38.4	-4.3	0.91
18cf53	37.8	38	0.2	35.8	-2.6	0
18cf54	38	38.7	0.7	44.1	8.3	2.66
18cf55	38.7	39.5	0.8	35.4	-8.7	1.18
18cf56	39.5	39.9	0.4	39.4	4.0	1
18cf57	39.9	40.3	0.4	42.0	2.6	2.4
18cf58	40.3	41.2	0.9	39.8	-2.1	2.31
18cf59	41.2	41.6	0.4	43.0	3.2	1.58
18cf60	41.6	42.1	0.5	49.7	6.7	4.95
18cf61	42.1	42.4	0.3	38.5	-11.3	0.64
18cf62	42.4	43.4	1	35.8	-2.7	0.91

18cf63	43.4	43.7	0.3	38.3	2.6	1.66
18cf64	43.7	44.2	0.5	37.0	-1.4	0.67
18cf65	44.2	44.6	0.4	38.5	1.6	0.98
18cf66	44.6	45.2	0.6	43.4	4.9	2.09
18cf67	45.2	46.3	1.1	45.2	1.8	5.83
18cf68	46.3	46.5	0.2	37.9	-7.3	0
18cf69	46.5	48	1.5	51.1	13.2	2.53
18cf70	48	48.2	0.2	44.1	-7.0	0
18cf71	48.2	48.8	0.6	56.2	12.1	4.81
18cf72	48.8	49	0.2	50.6	-5.6	0
18cf73	49	50.3	1.3	57.3	6.8	3.08
18cf74	50.3	50.6	0.3	51.9	-5.5	0.28
18cf75	50.6	50.9	0.3	55.3	3.5	1.54
18cf76	50.9	51.1	0.2	49.9	-5.5	0
18cf77	51.1	52.1	1	54.5	4.6	1.45
18cf78	52.1	52.3	0.2	49.9	-4.6	0
18cf79	52.3	52.8	0.5	45.9	-4.0	3.74
18cf80	52.8	53.4	0.6	51.2	5.3	5.93
18cf81	53.4	53.8	0.4	45.2	-6.0	0.58
18cf82	53.8	55.3	1.5	49.1	3.9	4.17
18cf83	55.3	56.1	0.8	54.3	5.2	2.72
18cf84	56.1	56.5	0.4	46.4	-8.0	4.46
18cf85	56.5	56.7	0.2	39.5	-6.9	0
18cf86	56.7	57.2	0.5	49.7	10.3	1.83
18cf87	57.2	57.4	0.2	44.7	-5.0	0
18cf88	57.4	57.7	0.3	48.8	4.1	4.41
18cf89	57.7	58	0.3	55.4	6.6	1.95
18cf90	58	58.6	0.6	49.5	-5.9	2.96
18cf91	58.6	58.9	0.3	37.8	-11.8	0
19cf1	0	3	3	0.0		0
19cf2	3	3.7	0.7	40.3		2.79
19cf3	3.7	4.4	0.7	42.1	1.8	2.19
19cf4	4.4	4.6	0.2	38.5	-3.5	0
19cf5	4.6	5.3	0.7	44.5	6.0	1.58
19cf6	5.3	5.5	0.2	38.2	-6.3	0
19cf7	5.5	6	0.5	43.6	5.3	1.27
19cf8	6	6.2	0.2	47.9	4.4	0
19cf9	6.2	7	0.8	41.4	-6.5	3.09
19cf10	7	15.3	8.3	34.9	-6.5	1.58
19cf11	15.3	16	0.7	38.0	3.1	1.45
19cf12	16	19.7	3.7	35.4	-2.5	1.04
19cf13	19.7	20.1	0.4	38.6	3.1	1.28
19cf14	20.1	20.4	0.3	35.1	-3.4	0.71
19cf15	20.4	20.7	0.3	37.8	2.7	0.65
19cf16	20.7	21.1	0.4	35.3	-2.5	0.82
19cf17	21.1	22.2	1.1	37.5	2.2	0.84
19cf18	22.2	22.8	0.6	36.3	-1.2	0.37
19cf19	22.8	23.3	0.5	40.0	3.7	1.73
19cf20	23.3	24	0.7	41.8	1.9	0.86
19cf21	24	25	1	39.2	-2.6	1.56
19cf22	25	25.4	0.4	46.6	7.4	3.17
19cf23	25.4	25.9	0.5	39.6	-7.0	2.08
19cf24	25.9	26.1	0.2	48.2	8.7	0
19cf25	26.1	26.3	0.2	39.2	-9.0	0
19cf26	26.3	27	0.7	43.3	4.1	2.98

19cf27	27	27.5	0.5	37.3	-6.0	1.31
19cf28	27.5	28.6	1.1	35.8	-1.5	0.76
19cf29	28.6	29.5	0.9	38.3	2.5	0.56
19cf30	29.5	30.5	1	35.5	-2.8	0.77
19cf31	30.5	31	0.5	37.6	2.1	0.51
19cf32	31	31.9	0.9	42.9	5.4	2.04
19cf33	31.9	33	1.1	49.3	6.4	2.72
19cf34	33	33.3	0.3	36.1	-13.2	1.67
19cf35	33.3	35.2	1.9	43.8	7.7	2.33
19cf36	35.2	35.7	0.5	38.0	-5.8	1.09
19cf37	35.7	37.3	1.6	35.4	-2.6	0.78
19cf38	37.3	38.4	1.1	38.0	2.7	1.54
19cf39	38.4	38.7	0.3	43.0	5.0	1.38
19cf40	38.7	39.9	1.2	39.4	-3.6	1.72
19cf41	39.9	40.6	0.7	43.4	4.1	2.23
19cf42	40.6	41.1	0.5	38.0	-5.5	0.93
19cf43	41.1	41.6	0.5	36.3	-1.7	2.04
19cf44	41.6	42.2	0.6	41.3	5.0	2.32
19cf45	42.2	43	0.8	38.2	-3.1	1.14
19cf46	43	43.5	0.5	36.6	-1.6	0.43
19cf47	43.5	43.9	0.4	37.2	0.6	0.54
19cf48	43.9	44.3	0.4	36.1	-1.1	0.54
19cf49	44.3	44.6	0.3	38.6	2.5	0.52
19cf50	44.6	48.2	3.6	34.7	-3.9	0.86
19cf51	48.2	48.4	0.2	37.5	2.8	0
19cf52	48.4	48.7	0.3	36.1	-1.4	0.56
19cf53	48.7	49.2	0.5	38.6	2.5	1.01
19cf54	49.2	49.4	0.2	36.6	-2.0	0
19cf55	49.4	49.6	0.2	42.1	5.5	0
19cf56	49.6	49.8	0.2	38.6	-3.5	0
19cf57	49.8	51.8	2	35.9	-2.6	0.93
19cf58	51.8	52	0.2	37.7	1.8	0
19cf59	52	52.3	0.3	36.5	-1.2	0.32
19cf60	52.3	52.7	0.4	40.2	3.8	1.69
19cf61	52.7	53.5	0.8	37.1	-3.1	4.17
19cf62	53.5	54.1	0.6	45.4	8.3	2.13
19cf63	54.1	54.5	0.4	38.6	-6.8	1.31
19cf64	54.5	55.6	1.1	42.1	3.5	1.76
19cf65	55.6	55.9	0.3	39.8	-2.3	0.51
19cf66	55.9	56.2	0.3	47.2	7.3	3.87
19cf67	56.2	56.4	0.2	43.7	-3.5	0
19cf68	56.4	56.8	0.4	48.1	4.4	3.69
20cf1	0	3	3	0.0		0
20cf2	3	3.3	0.3	44.1		3.02
20cf3	3.3	3.5	0.2	43.0	-1.1	0
20cf4	3.5	3.8	0.3	48.9	5.9	2.02
20cf5	3.8	4	0.2	42.6	-6.3	0
20cf6	4	4.3	0.3	51.3	8.7	5.83
20cf7	4.3	4.5	0.2	44.9	-6.4	0
20cf8	4.5	4.9	0.4	49.9	5.0	0.74
20cf9	4.9	5.3	0.4	43.7	-6.2	2.52
20cf10	5.3	5.5	0.2	52.8	9.1	0
20cf11	5.5	6.2	0.7	45.3	-7.6	2.1
20cf12	6.2	6.8	0.6	50.8	5.6	2.51
20cf13	6.8	7	0.2	53.6	2.7	0

20cf14	7	7.2	0.2	47.9	-5.7	0
20cf15	7.2	7.5	0.3	44.6	-3.3	3.67
20cf16	7.5	7.7	0.2	53.3	8.7	0
20cf17	7.7	8.3	0.6	44.9	-8.5	1.9
20cf18	8.3	8.5	0.2	37.2	-7.7	0
20cf19	8.5	8.8	0.3	48.5	11.4	1.93
20cf20	8.8	9.2	0.4	42.5	-6.0	1.83
20cf21	9.2	10.1	0.9	40.1	-2.4	2.58
20cf22	10.1	10.3	0.2	43.4	3.3	0
20cf23	10.3	10.5	0.2	47.7	4.3	0
20cf24	10.5	10.8	0.3	44.8	-2.9	1.34
20cf25	10.8	11.6	0.8	46.5	1.7	2.66
20cf26	11.6	12.3	0.7	42.4	-4.1	2.65
20cf27	12.3	12.5	0.2	49.0	6.6	0
20cf28	12.5	12.7	0.2	43.1	-5.9	0
20cf29	12.7	13.5	0.8	38.3	-4.8	0.84
20cf30	13.5	15	1.5	36.4	-1.8	0.64
20cf31	15	15.4	0.4	38.7	2.2	1.23
20cf32	15.4	15.9	0.5	42.4	3.8	1.19
20cf33	15.9	16.8	0.9	38.3	-4.1	1.2
20cf34	16.8	17	0.2	36.7	-1.7	0
20cf35	17	17.5	0.5	38.5	1.8	1.43
20cf36	17.5	19.9	2.4	35.7	-2.8	0.9
20cf37	19.9	20.2	0.3	37.7	2.0	0.21
20cf38	20.2	21.2	1	36.2	-1.5	0.89
20cf39	21.2	21.6	0.4	38.3	2.1	1.04
20cf40	21.6	22.1	0.5	42.1	3.8	1.07
20cf41	22.1	22.4	0.3	39.3	-2.8	0.31
20cf42	22.4	22.8	0.4	44.5	5.2	2.19
20cf43	22.8	23	0.2	47.3	2.8	0
20cf44	23	23.8	0.8	42.0	-5.3	1.94
20cf45	23.8	24.3	0.5	39.4	-2.6	1.4
20cf46	24.3	24.6	0.3	36.4	-3.0	0.35
20cf47	24.6	27.8	3.2	38.8	2.4	1.35
20cf48	27.8	28.4	0.6	42.4	3.6	2.29
20cf49	28.4	29.7	1.3	40.0	-2.4	1.09
20cf50	29.7	30.3	0.6	42.8	2.8	0.94
20cf51	30.3	34	3.7	39.3	-3.5	1.51
20cf52	34	34.2	0.2	41.8	2.5	0
20cf53	34.2	34.5	0.3	39.6	-2.2	1.16
20cf54	34.5	34.7	0.2	36.3	-3.3	0
20cf55	34.7	34.9	0.2	37.2	0.9	0
20cf56	34.9	35.6	0.7	45.2	8.0	2.68
20cf57	35.6	36.2	0.6	37.4	-7.8	1.12
20cf58	36.2	36.6	0.4	42.8	5.4	1.74
20cf59	36.6	37.6	1	38.5	-4.3	1.14
20cf60	37.6	39.1	1.5	42.5	4.0	1.62
20cf61	39.1	39.3	0.2	47.1	4.6	0
20cf62	39.3	40.2	0.9	44.0	-3.1	3.98
20cf63	40.2	40.4	0.2	53.4	9.3	0
20cf64	40.4	40.6	0.2	50.0	-3.3	0
20cf65	40.6	40.8	0.2	54.4	4.4	0
20cf66	40.8	41.3	0.5	43.1	-11.3	3.16
20cf67	41.3	41.7	0.4	37.2	-5.9	0.8
20cf68	41.7	42.2	0.5	55.3	18.1	2.5
20cf69	42.2	42.4	0.2	43.1	-12.2	0

20cf70	42.4	42.7	0.3	50.3	7.2	0.84
20cf71	42.7	43	0.3	44.3	-6.0	1.49
20cf72	43	43.2	0.2	49.2	5.0	0
20cf73	43.2	43.4	0.2	41.5	-7.7	0
20cf74	43.4	43.7	0.3	52.2	10.6	1.4
20cf75	43.7	44.8	1.1	42.8	-9.4	3.23
20cf76	44.8	45	0.2	54.2	11.4	0
20cf77	45	45.3	0.3	48.4	-5.8	3.06
20cf78	45.3	45.5	0.2	42.7	-5.8	0
20cf79	45.5	45.9	0.4	48.7	6.0	2.4
20cf80	45.9	46.1	0.2	44.6	-4.1	0
20cf81	46.1	47.3	1.2	48.6	4.1	2.86
20cf82	47.3	47.5	0.2	53.5	4.9	0
20cf83	47.5	47.7	0.2	51.9	-1.6	0
20cf84	47.7	48	0.3	54.3	2.4	0.45
20cf85	48	49.4	1.4	51.1	-3.2	2.35
20cf86	49.4	49.6	0.2	43.1	-8.0	0
20cf87	49.6	50	0.4	49.5	6.4	4.05
20cf88	50	50.2	0.2	43.6	-5.8	0
20cf89	50.2	50.9	0.7	39.6	-4.1	0.68
20cf90	50.9	51.1	0.2	44.1	4.5	0
20cf91	51.1	51.7	0.6	53.4	9.3	0.78
20cf92	51.7	51.9	0.2	45.5	-7.9	0
20cf93	51.9	52.5	0.6	53.0	7.5	2.55
20cf94	52.5	52.8	0.3	42.1	-10.9	1.42
20cf95	52.8	53.1	0.3	53.0	10.8	1.28
20cf96	53.1	54.2	1.1	50.6	-2.4	2.12
20cf97	54.2	54.7	0.5	40.0	-10.6	0.35
20cf98	54.7	55	0.3	43.0	3.0	1.98
20cf99	55	55.3	0.3	49.1	6.1	2.85
20cf100	55.3	55.8	0.5	53.8	4.8	0.74
20cf101	55.8	56.2	0.4	51.5	-2.3	0.81
20cf102	56.2	56.4	0.2	40.6	-10.9	0
20cf103	56.4	57.1	0.7	50.2	9.6	1.96
20cf104	57.1	61.3	4.2	58.5	8.3	5.35
21cf1	0	3	3	0.0		0
21cf2	3	3.5	0.5	38.5		1.61
21cf3	3.5	3.8	0.3	35.9	-2.6	1.23
21cf4	3.8	4.1	0.3	37.5	1.5	0.3
21cf5	4.1	7.6	3.5	34.8	-2.7	0.79
21cf6	7.6	7.9	0.3	38.6	3.8	1.42
21cf7	7.9	8.1	0.2	42.1	3.6	0
21cf8	8.1	8.9	0.8	39.2	-3.0	1.87
21cf9	8.9	10.3	1.4	42.5	3.3	2.51
21cf10	10.3	10.6	0.3	39.3	-3.2	0.41
21cf11	10.6	10.8	0.2	43.8	4.5	0
21cf12	10.8	11.7	0.9	39.3	-4.5	1.08
21cf13	11.7	13.2	1.5	35.6	-3.7	0.76
21cf14	13.2	13.6	0.4	38.3	2.7	1.33
21cf15	13.6	14.3	0.7	36.2	-2.0	0.39
21cf16	14.3	15.4	1.1	38.6	2.4	2.08
21cf17	15.4	15.6	0.2	35.0	-3.6	0
21cf18	15.6	16.1	0.5	43.1	8.1	2.2
21cf19	16.1	18.4	2.3	36.8	-6.2	1.66
21cf20	18.4	19.7	1.3	39.0	2.2	1.33

21cf21	19.7	21.6	1.9	35.6	-3.4	0.58
21cf22	21.6	22.3	0.7	38.6	3.0	0.91
21cf23	22.3	23.3	1	44.7	6.1	1.72
21cf24	23.3	23.6	0.3	38.9	-5.9	2.27
21cf25	23.6	23.8	0.2	44.6	5.7	0
21cf26	23.8	24.5	0.7	38.6	-6.0	1.21
21cf27	24.5	24.7	0.2	51.5	12.9	0
21cf28	24.7	24.9	0.2	38.8	-12.7	0
21cf29	24.9	25.1	0.2	50.8	12.0	0
21cf30	25.1	25.3	0.2	40.2	-10.6	0
21cf31	25.3	25.5	0.2	51.4	11.2	0
21cf32	25.5	25.8	0.3	37.4	-14.0	1.87
21cf33	25.8	26	0.2	45.2	7.8	0
21cf34	26	26.4	0.4	50.9	5.7	1.4
21cf35	26.4	26.9	0.5	42.7	-8.2	1.3
21cf36	26.9	27.2	0.3	38.2	-4.4	1.16
21cf37	27.2	27.4	0.2	42.6	4.4	0
21cf38	27.4	27.8	0.4	39.3	-3.3	1.61
21cf39	27.8	28.1	0.3	49.9	10.6	2.76
21cf40	28.1	28.4	0.3	36.2	-13.6	1.08
21cf41	28.4	28.7	0.3	51.9	15.6	2.91
21cf42	28.7	28.9	0.2	43.9	-8.0	0
21cf43	28.9	29.1	0.2	48.0	4.1	0
21cf44	29.1	29.7	0.6	42.3	-5.7	0.51
21cf45	29.7	30.4	0.7	40.6	-1.6	2.5
21cf46	30.4	30.8	0.4	36.4	-4.2	0.71
21cf47	30.8	31	0.2	37.8	1.3	0
21cf48	31	31.2	0.2	36.5	-1.3	0
21cf49	31.2	31.5	0.3	38.2	1.7	0.7
21cf50	31.5	31.7	0.2	41.8	3.6	0
21cf51	31.7	32	0.3	39.1	-2.6	1.29
21cf52	32	32.2	0.2	35.9	-3.2	0
21cf53	32.2	32.7	0.5	37.8	1.9	0.77
21cf54	32.7	33.2	0.5	45.4	7.6	2.68
21cf55	33.2	34.2	1	38.9	-6.5	0.92
21cf56	34.2	34.7	0.5	43.4	4.6	3.11
21cf57	34.7	35.4	0.7	45.5	2.0	4.66
21cf58	35.4	35.7	0.3	41.8	-3.7	0.53
21cf59	35.7	36	0.3	38.9	-2.9	0.96
21cf60	36	36.2	0.2	42.5	3.6	0
21cf61	36.2	36.6	0.4	36.3	-6.2	0.95
21cf62	36.6	37.5	0.9	42.6	6.3	1.1
21cf63	37.5	37.9	0.4	47.6	5.1	1.93
21cf64	37.9	38.2	0.3	38.7	-9.0	3.37
21cf65	38.2	38.5	0.3	49.5	10.8	2.03
21cf66	38.5	38.9	0.4	42.9	-6.6	0.93
21cf67	38.9	39.1	0.2	46.4	3.5	0
21cf68	39.1	39.5	0.4	45.9	-0.5	1.91
21cf69	39.5	39.8	0.3	38.0	-7.9	0.4
21cf70	39.8	40.4	0.6	42.9	4.9	1.73
21cf71	40.4	41.1	0.7	39.5	-3.4	1.55
21cf72	41.1	41.9	0.8	44.5	5.1	1.87
21cf73	41.9	42.6	0.7	35.8	-8.7	2.42
21cf74	42.6	42.9	0.3	45.1	9.3	1.41
21cf75	42.9	43.1	0.2	37.9	-7.3	0
21cf76	43.1	43.6	0.5	49.7	11.8	1.62

21cf77	43.6	44.2	0.6	41.8	-7.8	3.22
21cf78	44.2	44.4	0.2	48.2	6.4	0
21cf79	44.4	44.6	0.2	43.9	-4.3	0
21cf80	44.6	45.4	0.8	49.0	5.0	1.36
21cf81	45.4	46	0.6	42.3	-6.7	4.33
21cf82	46	48	2	38.4	-3.9	1.15
21cf83	48	48.8	0.8	35.8	-2.6	0.67
21cf84	48.8	49.3	0.5	38.1	2.3	0.64
21cf85	49.3	50.3	1	36.4	-1.7	0.58
21cf86	50.3	50.8	0.5	38.8	2.4	0.97
21cf87	50.8	51.7	0.9	44.3	5.5	2.45
21cf88	51.7	52	0.3	35.4	-8.9	1.36
21cf89	52	52.4	0.4	40.9	5.4	2.12
21cf90	52.4	52.6	0.2	36.2	-4.7	0
21cf91	52.6	53.3	0.7	40.0	3.8	1.92
21cf92	53.3	53.5	0.2	48.0	8.0	0
21cf93	53.5	54.1	0.6	42.8	-5.2	2.91
22cf1	0	3	3	0.0		0
22cf2	3	3.9	0.9	48.4		5.23
22cf3	3.9	5.4	1.5	42.2	-6.2	2.19
22cf4	5.4	5.7	0.3	38.5	-3.7	2.71
22cf5	5.7	6	0.3	44.5	6.0	0.52
22cf6	6	6.2	0.2	36.0	-8.5	0
22cf7	6.2	7.5	1.3	38.9	2.9	1.53
22cf8	7.5	8	0.5	43.8	4.9	1.54
22cf9	8	8.2	0.2	37.9	-5.9	0
22cf10	8.2	8.8	0.6	43.7	5.8	4.42
22cf11	8.8	9.7	0.9	42.5	-1.2	2.9
22cf12	9.7	10.4	0.7	38.4	-4.1	1.23
22cf13	10.4	10.7	0.3	43.5	5.1	0.94
22cf14	10.7	11.8	1.1	39.2	-4.3	1.25
22cf15	11.8	12	0.2	36.6	-2.6	0
22cf16	12	12.2	0.2	43.9	7.2	0
22cf17	12.2	12.7	0.5	38.0	-5.9	0.9
22cf18	12.7	13.3	0.6	42.6	4.6	2.1
22cf19	13.3	13.5	0.2	39.0	-3.6	0
22cf20	13.5	18.1	4.6	34.4	-4.6	0.93
22cf21	18.1	18.6	0.5	38.4	3.9	0.92
22cf22	18.6	18.9	0.3	36.4	-1.9	0.46
22cf23	18.9	19.2	0.3	38.7	2.2	1.11
22cf24	19.2	29.5	10.3	34.4	-4.2	1.05
22cf25	29.5	29.7	0.2	37.8	3.4	0
22cf26	29.7	30.1	0.4	35.8	-2.0	0.91
22cf27	30.1	31.3	1.2	37.9	2.1	0.86
22cf28	31.3	32	0.7	36.3	-1.5	0.3
22cf29	32	32.7	0.7	37.9	1.5	0.68
22cf30	32.7	33.1	0.4	35.9	-2.0	0.75
22cf31	33.1	33.6	0.5	38.9	3.0	1.8
22cf32	33.6	33.9	0.3	34.7	-4.2	1.63
22cf33	33.9	34.5	0.6	37.3	2.6	0.54
22cf34	34.5	34.7	0.2	35.8	-1.5	0
22cf35	34.7	35.4	0.7	38.5	2.6	1.17
22cf36	35.4	35.8	0.4	36.2	-2.3	1.42
22cf37	35.8	36.1	0.3	38.1	2.0	0.85
22cf38	36.1	45.4	9.3	34.3	-3.8	1.06

22cf39	45.4	45.6	0.2	37.7	3.4	0
22cf40	45.6	47.1	1.5	35.1	-2.6	0.61
22cf41	47.1	48.8	1.7	38.6	3.5	1.82
22cf42	48.8	49.3	0.5	42.5	3.9	1.3
22cf43	49.3	51.2	1.9	39.2	-3.3	1.45
22cf44	51.2	51.9	0.7	43.6	4.4	1.67
22cf45	51.9	52.8	0.9	40.8	-2.8	1.74
22cf46	52.8	53	0.2	44.2	3.4	0
22cf47	53	53.3	0.3	36.7	-7.6	0.73
22cf48	53.3	53.8	0.5	41.4	4.7	1.18
22cf49	53.8	54.4	0.6	37.5	-3.9	0.82
22cf50	54.4	54.7	0.3	36.7	-0.8	0.24
22cf51	54.7	55.8	1.1	38.9	2.2	1.15
22cf52	55.8	57.3	1.5	35.8	-3.1	1.02
22cf53	57.3	58.4	1.1	38.3	2.6	1.43
22cf54	58.4	58.8	0.4	42.3	4.0	0.9
22cf55	58.8	60.6	1.8	39.3	-3.1	1.01
22cf56	60.6	61.6	1	44.6	5.3	2.1
22cf57	61.6	63.3	1.7	48.1	3.5	1.89
22cf58	63.3	63.6	0.3	55.0	7.0	2.13
22cf59	63.6	63.9	0.3	50.6	-4.4	3.04
22cf60	63.9	64.1	0.2	53.7	3.0	0
22cf61	64.1	64.5	0.4	45.3	-8.3	3.68
23cf1	0	3	3	0.0		0
23cf2	3	3.6	0.6	40.7		1.36
23cf3	3.6	4.9	1.3	44.1	3.5	2.08
23cf4	4.9	5.2	0.3	38.7	-5.5	2.85
23cf5	5.2	5.6	0.4	43.8	5.2	1.76
23cf6	5.6	5.9	0.3	39.4	-4.4	1.02
23cf7	5.9	6.2	0.3	43.2	3.8	1.54
23cf8	6.2	6.6	0.4	37.6	-5.6	2.02
23cf9	6.6	7	0.4	44.0	6.4	1.65
23cf10	7	7.2	0.2	39.5	-4.5	0
23cf11	7.2	7.6	0.4	41.3	1.8	0.72
23cf12	7.6	8	0.4	38.1	-3.1	1.02
23cf13	8	8.7	0.7	35.9	-2.2	0.85
23cf14	8.7	8.9	0.2	38.5	2.6	0
23cf15	8.9	9.1	0.2	36.8	-1.7	0
23cf16	9.1	9.4	0.3	38.2	1.5	1.19
23cf17	9.4	9.9	0.5	42.2	4.0	1.96
23cf18	9.9	10.2	0.3	39.3	-2.9	1.73
23cf19	10.2	10.7	0.5	44.0	4.7	2.1
23cf20	10.7	10.9	0.2	47.3	3.3	0
23cf21	10.9	11.1	0.2	41.3	-6.0	0
23cf22	11.1	11.5	0.4	46.8	5.5	3.64
23cf23	11.5	11.7	0.2	40.3	-6.5	0
23cf24	11.7	12.1	0.4	44.8	4.5	3.46
23cf25	12.1	12.6	0.5	40.0	-4.7	0.62
23cf26	12.6	13.7	1.1	42.1	2.1	1.12
23cf27	13.7	14.2	0.5	38.4	-3.7	0.72
23cf28	14.2	16.1	1.9	43.4	5.0	2.56
23cf29	16.1	17.5	1.4	39.2	-4.2	1.77
23cf30	17.5	18	0.5	36.1	-3.1	0.4
23cf31	18	18.8	0.8	37.9	1.8	1.42
23cf32	18.8	19.2	0.4	36.3	-1.6	0.39

23cf33	19.2	19.7	0.5	39.8	3.5	1.92
23cf34	19.7	21.2	1.5	38.2	-1.6	1.39
23cf35	21.2	21.4	0.2	41.5	3.4	0
23cf36	21.4	23	1.6	38.5	-3.1	1.07
23cf37	23	23.2	0.2	43.5	5.0	0
23cf38	23.2	23.5	0.3	35.9	-7.6	0.07
23cf39	23.5	23.8	0.3	38.3	2.4	0.26
23cf40	23.8	26.2	2.4	36.1	-2.2	1.09
23cf41	26.2	26.5	0.3	38.1	2.0	0.68
23cf42	26.5	26.8	0.3	36.5	-1.6	0.32
23cf43	26.8	28.3	1.5	38.0	1.4	0.87
23cf44	28.3	28.7	0.4	34.7	-3.3	0.56
23cf45	28.7	29.3	0.6	40.0	5.3	2.7
23cf46	29.3	29.8	0.5	43.7	3.8	0.7
23cf47	29.8	30.1	0.3	37.5	-6.2	2.37
23cf48	30.1	30.4	0.3	43.8	6.3	2.46
23cf49	30.4	32.4	2	39.2	-4.6	1.54
23cf50	32.4	32.8	0.4	37.0	-2.2	0.46
23cf51	32.8	33.7	0.9	39.7	2.7	1.83
23cf52	33.7	34.4	0.7	44.7	5.0	3.57
23cf53	34.4	34.9	0.5	47.6	3.0	0.79
23cf54	34.9	35.4	0.5	42.8	-4.8	1.52
23cf55	35.4	35.9	0.5	38.1	-4.7	2.01
23cf56	35.9	36.2	0.3	34.7	-3.4	0.56
23cf57	36.2	37.6	1.4	39.4	4.8	2.16
23cf58	37.6	37.8	0.2	44.9	5.5	0
23cf59	37.8	38.1	0.3	37.8	-7.1	1.68
23cf60	38.1	40	1.9	43.6	5.8	1.94
23cf61	40	40.2	0.2	47.0	3.4	0
23cf62	40.2	40.4	0.2	43.4	-3.6	0
23cf63	40.4	41.1	0.7	39.0	-4.4	1.44
23cf64	41.1	41.5	0.4	36.2	-2.8	0.83
23cf65	41.5	41.7	0.2	39.5	3.3	0
23cf66	41.7	42	0.3	42.7	3.3	1.42
23cf67	42	42.7	0.7	37.7	-5.1	0.81
23cf68	42.7	44.4	1.7	35.3	-2.3	1
23cf69	44.4	44.6	0.2	38.4	3.0	0
23cf70	44.6	46.6	2	36.2	-2.2	2.36
23cf71	46.6	47.2	0.6	38.6	2.4	1.59
23cf72	47.2	47.5	0.3	41.7	3.1	0.55
23cf73	47.5	47.8	0.3	38.4	-3.3	2.78
23cf74	47.8	48	0.2	43.8	5.4	0
23cf75	48	48.3	0.3	38.0	-5.8	1.27
23cf76	48.3	48.8	0.5	43.7	5.7	2.39
23cf77	48.8	49.3	0.5	38.8	-4.9	1.06
23cf78	49.3	49.8	0.5	36.0	-2.8	0.66
23cf79	49.8	50.7	0.9	39.4	3.4	0.91
23cf80	50.7	51.3	0.6	36.6	-2.8	0.4
23cf81	51.3	51.5	0.2	41.9	5.3	0
23cf82	51.5	51.9	0.4	36.4	-5.5	0.51
23cf83	51.9	52.2	0.3	39.9	3.6	1
23cf84	52.2	53.4	1.2	43.0	3.0	2
23cf85	53.4	53.9	0.5	42.5	-0.5	5.58
23cf86	53.9	54.6	0.7	44.8	2.3	2.8
23cf87	54.6	54.9	0.3	34.9	-9.9	1.13
23cf88	54.9	55.2	0.3	42.4	7.5	1.85

23cf89	55.2	55.4	0.2	54.9	12.5	0
24cf1	0	3	3	0.0		0
24cf2	3	3.3	0.3	43.8		1.81
24cf3	3.3	3.6	0.3	46.7	3.0	0.51
24cf4	3.6	4.1	0.5	42.7	-4.0	1.41
24cf5	4.1	4.7	0.6	40.1	-2.6	0.96
24cf6	4.7	5	0.3	43.8	3.7	1.78
24cf7	5	5.3	0.3	39.1	-4.6	1.57
24cf8	5.3	5.7	0.4	42.0	2.8	0.78
24cf9	5.7	5.9	0.2	38.7	-3.3	0
24cf10	5.9	6.1	0.2	43.6	4.9	0
24cf11	6.1	6.3	0.2	40.3	-3.4	0
24cf12	6.3	8.1	1.8	42.9	2.6	1.12
24cf13	8.1	8.4	0.3	47.8	4.9	2.16
24cf14	8.4	8.6	0.2	42.6	-5.2	0
24cf15	8.6	9	0.4	39.6	-3.0	0.94
24cf16	9	9.3	0.3	41.9	2.2	0.97
24cf17	9.3	10.5	1.2	39.1	-2.8	1.63
24cf18	10.5	11.7	1.2	35.8	-3.2	0.81
24cf19	11.7	12	0.3	38.3	2.4	0.34
24cf20	12	12.2	0.2	36.2	-2.1	0
24cf21	12.2	17.3	5.1	39.0	2.8	1.66
24cf22	17.3	18.1	0.8	36.6	-2.4	0.37
24cf23	18.1	18.7	0.6	38.4	1.8	1.13
24cf24	18.7	20.5	1.8	42.8	4.5	2.44
24cf25	20.5	20.7	0.2	51.6	8.7	0
24cf26	20.7	21	0.3	40.0	-11.6	0.56
24cf27	21	22.2	1.2	46.5	6.5	2.92
24cf28	22.2	22.9	0.7	44.4	-2.1	2.77
24cf29	22.9	23.3	0.4	49.5	5.1	0.7
24cf30	23.3	23.6	0.3	45.0	-4.5	3.91
24cf31	23.6	23.9	0.3	38.7	-6.2	1.3
24cf32	23.9	24.2	0.3	46.2	7.5	3.28
24cf33	24.2	24.5	0.3	47.6	1.4	1.14
24cf34	24.5	24.8	0.3	44.5	-3.1	1.17
24cf35	24.8	25	0.2	52.5	8.0	0
24cf36	25	25.2	0.2	45.7	-6.8	0
24cf37	25.2	25.5	0.3	48.4	2.7	2.17
24cf38	25.5	27.6	2.1	43.2	-5.3	2.66
24cf39	27.6	27.8	0.2	40.6	-2.6	0
24cf40	27.8	28.6	0.8	47.0	6.4	2.81
24cf41	28.6	28.8	0.2	40.5	-6.4	0
24cf42	28.8	29.2	0.4	48.2	7.7	3.48
24cf43	29.2	30.5	1.3	46.4	-1.8	3.68
24cf44	30.5	31.1	0.6	43.3	-3.1	1.57
24cf45	31.1	31.3	0.2	40.1	-3.2	0
24cf46	31.3	31.7	0.4	43.8	3.8	2.04
24cf47	31.7	32.5	0.8	46.2	2.3	3.78
24cf48	32.5	34.5	2	43.5	-2.7	1.73
24cf49	34.5	35.2	0.7	47.2	3.7	3.02
24cf50	35.2	36.1	0.9	44.0	-3.2	2.07
24cf51	36.1	36.6	0.5	51.2	7.2	2.82
24cf52	36.6	36.8	0.2	44.2	-7.0	0
24cf53	36.8	37.5	0.7	46.7	2.5	1.48
24cf54	37.5	37.7	0.2	39.9	-6.9	0

24cf55	37.7	38.6	0.9	50.9	11.1	1.51
24cf56	38.6	39	0.4	42.9	-8.0	1.74
24cf57	39	39.2	0.2	48.7	5.8	0
24cf58	39.2	39.4	0.2	42.9	-5.8	0
24cf59	39.4	40.9	1.5	49.1	6.2	2.73
24cf60	40.9	42.9	2	43.4	-5.7	1.29
24cf61	42.9	44.2	1.3	39.5	-3.8	1.46
24cf62	44.2	44.4	0.2	42.9	3.4	0
24cf63	44.4	45.5	1.1	50.0	7.0	2.96
24cf64	45.5	45.8	0.3	55.1	5.1	0.78
24cf65	45.8	46	0.2	51.4	-3.7	0
24cf66	46	46.2	0.2	43.4	-8.0	0
24cf67	46.2	48.6	2.4	49.9	6.5	3.02
24cf68	48.6	50.8	2.2	61.0	11.0	3.87
25cf1	0	3	3	0.0		0
25cf2	3	3.7	0.7	40.6		1.22
25cf3	3.7	4.2	0.5	42.4	1.8	1.1
25cf4	4.2	5.6	1.4	38.7	-3.8	1.74
25cf5	5.6	6.7	1.1	36.1	-2.6	0.67
25cf6	6.7	7.7	1	38.3	2.2	0.82
25cf7	7.7	7.9	0.2	41.2	2.8	0
25cf8	7.9	8.5	0.6	34.4	-6.7	1.82
25cf9	8.5	10.1	1.6	38.0	3.6	1.13
25cf10	10.1	10.3	0.2	41.1	3.1	0
25cf11	10.3	10.7	0.4	36.6	-4.5	3
25cf12	10.7	11.9	1.2	37.9	1.2	0.85
25cf13	11.9	13.4	1.5	42.1	4.3	2.89
25cf14	13.4	14.1	0.7	39.4	-2.7	0.69
25cf15	14.1	14.4	0.3	42.2	2.8	0.78
25cf16	14.4	14.6	0.2	36.0	-6.2	0
25cf17	14.6	14.9	0.3	43.7	7.7	1.23
25cf18	14.9	15.1	0.2	40.2	-3.5	0
25cf19	15.1	15.3	0.2	44.7	4.5	0
25cf20	15.3	15.5	0.2	40.6	-4.1	0
25cf21	15.5	16	0.5	42.7	2.1	2.4
25cf22	16	16.4	0.4	37.2	-5.5	1.54
25cf23	16.4	16.6	0.2	42.5	5.3	0
25cf24	16.6	17	0.4	39.3	-3.2	1.02
25cf25	17	18	1	41.1	1.9	1.8
25cf26	18	18.5	0.5	38.5	-2.6	0.96
25cf27	18.5	18.9	0.4	35.6	-2.9	0.82
25cf28	18.9	21.8	2.9	38.8	3.2	2.16
25cf29	21.8	22.1	0.3	43.5	4.7	2.68
25cf30	22.1	23.3	1.2	38.5	-5.0	1.6
25cf31	23.3	23.6	0.3	35.5	-3.0	0.67
25cf32	23.6	24.1	0.5	38.7	3.2	1.14
25cf33	24.1	26.4	2.3	34.7	-4.0	0.77
25cf34	26.4	27.2	0.8	38.9	4.2	1.99
25cf35	27.2	28.9	1.7	35.6	-3.3	1
25cf36	28.9	29.5	0.6	45.3	9.6	1.84
25cf37	29.5	29.8	0.3	39.2	-6.0	1.15
25cf38	29.8	30.7	0.9	43.4	4.1	3
25cf39	30.7	31.5	0.8	39.7	-3.6	1.93
25cf40	31.5	31.7	0.2	35.3	-4.4	0
25cf41	31.7	32.1	0.4	38.6	3.3	1.24

25cf42	32.1	32.3	0.2	42.6	4.0	0
25cf43	32.3	32.5	0.2	37.9	-4.7	0
25cf44	32.5	32.7	0.2	44.8	6.9	0
25cf45	32.7	32.9	0.2	39.0	-5.8	0
25cf46	32.9	33.3	0.4	46.8	7.8	0.53
25cf47	33.3	33.9	0.6	39.1	-7.7	1.57
25cf48	33.9	34.1	0.2	44.5	5.4	0
25cf49	34.1	34.3	0.2	38.4	-6.1	0
25cf50	34.3	34.6	0.3	43.6	5.2	1.02
25cf51	34.6	35.5	0.9	39.9	-3.7	1.4
25cf52	35.5	36.4	0.9	36.0	-3.9	0.31
25cf53	36.4	36.6	0.2	39.1	3.0	0
25cf54	36.6	36.9	0.3	42.3	3.2	2.26
25cf55	36.9	37.5	0.6	46.3	4.0	2.31
25cf56	37.5	37.7	0.2	53.4	7.1	0
25cf57	37.7	38	0.3	41.2	-12.2	2.14
25cf58	38	38.6	0.6	49.3	8.1	5.46
25cf59	38.6	39.2	0.6	42.2	-7.1	0.74
25cf60	39.2	40.2	1	40.1	-2.1	1.73
25cf61	40.2	40.7	0.5	45.9	5.7	1.94
25cf62	40.7	42.5	1.8	37.8	-8.0	1.23
25cf63	42.5	42.9	0.4	42.5	4.7	2.18
25cf64	42.9	44.6	1.7	38.9	-3.6	1.59
25cf65	44.6	45	0.4	43.0	4.1	0.57
25cf66	45	45.2	0.2	38.8	-4.2	0
25cf67	45.2	45.8	0.6	41.9	3.1	1.66
25cf68	45.8	46.6	0.8	46.7	4.9	4.9
25cf69	46.6	47	0.4	41.3	-5.4	2.02
25cf70	47	48.3	1.3	47.0	5.7	4.75
25cf71	48.3	48.6	0.3	44.5	-2.5	0.58
25cf72	48.6	51.1	2.5	48.7	4.2	2.06
25cf73	51.1	51.3	0.2	55.2	6.5	0
25cf74	51.3	51.6	0.3	49.1	-6.1	1.23
25cf75	51.6	51.8	0.2	55.7	6.5	0
25cf76	51.8	52.2	0.4	49.7	-5.9	1.82
25cf77	52.2	53	0.8	57.8	8.1	2.48
25cf78	53	53.3	0.3	41.9	-15.9	1.55
25cf79	53.3	54.6	1.3	60.1	18.2	5.82
26cf1	0	3	3	0.0		0
26cf2	3	3.3	0.3	42.8		0.84
26cf3	3.3	3.5	0.2	47.5	4.8	0
26cf4	3.5	3.8	0.3	56.3	8.8	0.22
26cf5	3.8	5.1	1.3	49.8	-6.5	2.61
26cf6	5.1	5.6	0.5	44.6	-5.2	0.9
26cf7	5.6	6.2	0.6	47.8	3.2	1.72
26cf8	6.2	7.8	1.6	44.6	-3.2	1.26
26cf9	7.8	8.3	0.5	50.3	5.7	1.36
26cf10	8.3	8.6	0.3	53.8	3.5	2.41
26cf11	8.6	9.4	0.8	43.9	-9.9	1.62
26cf12	9.4	9.7	0.3	49.9	6.0	2.62
26cf13	9.7	10.4	0.7	42.8	-7.1	2.71
26cf14	10.4	10.7	0.3	48.1	5.3	1.69
26cf15	10.7	11	0.3	44.0	-4.1	0.91
26cf16	11	11.2	0.2	39.5	-4.5	0
26cf17	11.2	11.9	0.7	43.3	3.7	2.37

26cf18	11.9	12.1	0.2	48.9	5.7	0
26cf19	12.1	12.5	0.4	41.7	-7.3	3.64
26cf20	12.5	12.7	0.2	38.0	-3.7	0
26cf21	12.7	13.3	0.6	42.4	4.4	1.35
26cf22	13.3	14.8	1.5	49.4	7.0	2.82
26cf23	14.8	15.8	1	45.3	-4.1	0.86
26cf24	15.8	16.1	0.3	36.4	-8.9	0.6
26cf25	16.1	17.1	1	46.9	10.5	1.54
26cf26	17.1	17.6	0.5	44.2	-2.8	1.55
26cf27	17.6	17.8	0.2	37.9	-6.3	0
26cf28	17.8	19.1	1.3	43.3	5.4	1.67
26cf29	19.1	19.9	0.8	45.5	2.2	3.71
26cf30	19.9	20.3	0.4	43.3	-2.2	1.5
26cf31	20.3	20.6	0.3	52.8	9.5	0.98
26cf32	20.6	21.5	0.9	45.8	-7.0	1.22
26cf33	21.5	21.8	0.3	48.6	2.8	1.68
26cf34	21.8	22	0.2	44.0	-4.7	0
26cf35	22	22.8	0.8	48.1	4.1	3.02
26cf36	22.8	23	0.2	45.3	-2.8	0
26cf37	23	24.1	1.1	48.9	3.5	2.25
26cf38	24.1	24.3	0.2	54.2	5.3	0
26cf39	24.3	24.6	0.3	43.5	-10.6	1.98
26cf40	24.6	25.1	0.5	37.9	-5.7	1.29
26cf41	25.1	25.5	0.4	43.3	5.4	1.18
26cf42	25.5	26.1	0.6	47.5	4.2	3.63
26cf43	26.1	26.4	0.3	38.6	-8.9	1.52
26cf44	26.4	27.2	0.8	48.4	9.8	3.99
26cf45	27.2	27.9	0.7	43.6	-4.8	2.28
26cf46	27.9	28.1	0.2	40.8	-2.8	0
26cf47	28.1	30.8	2.7	44.8	4.0	1.36
26cf48	30.8	32.1	1.3	49.7	4.9	3.76
26cf49	32.1	32.7	0.6	56.5	6.9	4.12
26cf50	32.7	33.6	0.9	47.9	-8.7	3.83
26cf51	33.6	33.8	0.2	44.9	-3.0	0
26cf52	33.8	34.5	0.7	47.9	3.0	4.69
26cf53	34.5	35.3	0.8	38.2	-9.7	1.56
26cf54	35.3	35.5	0.2	42.3	4.1	0
26cf55	35.5	38.3	2.8	38.8	-3.5	1.36
26cf56	38.3	38.9	0.6	42.6	3.8	2.53
26cf57	38.9	39.2	0.3	40.6	-2.1	0.44
26cf58	39.2	40.3	1.1	43.8	3.2	1.36
26cf59	40.3	41.4	1.1	48.1	4.3	5.6
26cf60	41.4	41.6	0.2	44.0	-4.0	0
26cf61	41.6	42.1	0.5	51.8	7.7	3.83
27cf1	0	3	3	0.0		0
27cf2	3	3.3	0.3	36.8		0.53
27cf3	3.3	3.7	0.4	42.7	5.9	1.47
27cf4	3.7	4.1	0.4	52.4	9.7	3.31
27cf5	4.1	4.3	0.2	57.8	5.4	0
27cf6	4.3	5.7	1.4	49.6	-8.2	4.04
27cf7	5.7	6.2	0.5	55.7	6.1	2.76
27cf8	6.2	6.5	0.3	42.8	-12.9	1.29
27cf9	6.5	6.7	0.2	48.2	5.4	0
27cf10	6.7	6.9	0.2	41.9	-6.3	0
27cf11	6.9	7.6	0.7	39.3	-2.6	1.16

27cf12	7.6	8.1	0.5	50.0	10.7	2.21
27cf13	8.1	8.5	0.4	43.1	-6.9	1.81
27cf14	8.5	8.8	0.3	50.8	7.7	2.34
27cf15	8.8	9.5	0.7	38.9	-11.9	1.55
27cf16	9.5	9.7	0.2	41.7	2.9	0
27cf17	9.7	10.1	0.4	50.5	8.7	3.36
27cf18	10.1	10.4	0.3	41.7	-8.7	0.49
27cf19	10.4	11.3	0.9	39.8	-1.9	1.4
27cf20	11.3	11.7	0.4	35.8	-4.0	0.76
27cf21	11.7	12.6	0.9	38.5	2.7	1.22
27cf22	12.6	13	0.4	36.7	-1.8	0.82
27cf23	13	13.3	0.3	38.4	1.7	1.14
27cf24	13.3	14	0.7	36.3	-2.1	0.67
27cf25	14	15.1	1.1	38.8	2.5	1.77
27cf26	15.1	19	3.9	36.2	-2.6	2.21
27cf27	19	22.3	3.3	38.5	2.4	0.94
27cf28	22.3	22.7	0.4	34.7	-3.8	1.23
27cf29	22.7	23.9	1.2	38.4	3.6	1.04
27cf30	23.9	25.5	1.6	37.5	-0.9	1.78
27cf31	25.5	25.8	0.3	38.2	0.7	1.13
27cf32	25.8	27.8	2	35.8	-2.4	1.36
27cf33	27.8	28.7	0.9	38.0	2.2	1.08
27cf34	28.7	29.2	0.5	43.9	5.9	1.33
27cf35	29.2	30.4	1.2	36.2	-7.7	1.24
27cf36	30.4	30.8	0.4	38.2	2.0	0.97
27cf37	30.8	31	0.2	36.4	-1.8	0
27cf38	31	31.3	0.3	38.1	1.7	0.43
27cf39	31.3	33.4	2.1	34.8	-3.3	0.92
27cf40	33.4	33.8	0.4	37.6	2.7	0.5
27cf41	33.8	34.1	0.3	36.6	-1.0	0.19
27cf42	34.1	35.8	1.7	38.2	1.7	1.64
27cf43	35.8	36.9	1.1	41.6	3.3	1.24
27cf44	36.9	37.2	0.3	37.6	-3.9	2.02
27cf45	37.2	37.6	0.4	42.9	5.2	0.51
27cf46	37.6	38.5	0.9	38.4	-4.5	1.58
27cf47	38.5	39.5	1	36.2	-2.2	1.07
27cf48	39.5	39.8	0.3	37.7	1.6	0.74
27cf49	39.8	40.5	0.7	41.1	3.4	1.33
27cf50	40.5	40.9	0.4	38.4	-2.8	1.8
27cf51	40.9	42	1.1	47.3	8.9	2.91
27cf52	42	43.1	1.1	43.6	-3.7	2.39
27cf53	43.1	43.5	0.4	39.1	-4.5	0.63
27cf54	43.5	43.9	0.4	43.2	4.1	1.42
27cf55	43.9	44.1	0.2	39.8	-3.4	0
27cf56	44.1	44.5	0.4	44.6	4.8	1.18
27cf57	44.5	45.8	1.3	46.2	1.6	4.59
27cf58	45.8	46.5	0.7	36.8	-9.4	1.44
27cf59	46.5	47.8	1.3	44.0	7.2	1.95
27cf60	47.8	48	0.2	47.1	3.0	0
27cf61	48	49	1	42.6	-4.5	2.92
28cf1	0	3	3	0.0		0
28cf2	3	3.4	0.4	41.3		3.48
28cf3	3.4	3.6	0.2	47.8	6.6	0
28cf4	3.6	4.6	1	44.5	-3.3	1.93
28cf5	4.6	5.4	0.8	39.4	-5.1	2.31

28cf6	5.4	6.8	1.4	42.6	3.2	2.07
28cf7	6.8	7.1	0.3	48.1	5.4	1.06
28cf8	7.1	8.6	1.5	39.6	-8.5	1.67
28cf9	8.6	8.8	0.2	41.8	2.2	0
28cf10	8.8	10.2	1.4	39.0	-2.8	1.26
28cf11	10.2	10.4	0.2	35.6	-3.4	0
28cf12	10.4	10.9	0.5	43.1	7.6	0.97
28cf13	10.9	11.7	0.8	39.8	-3.3	1.28
28cf14	11.7	12.1	0.4	44.3	4.5	1.6
28cf15	12.1	12.7	0.6	39.0	-5.3	1.5
28cf16	12.7	12.9	0.2	44.6	5.7	0
28cf17	12.9	13.3	0.4	39.7	-4.9	2.81
28cf18	13.3	13.8	0.5	45.9	6.2	2.09
28cf19	13.8	14.6	0.8	46.7	0.8	3.49
28cf20	14.6	14.8	0.2	38.7	-8.0	0
28cf21	14.8	15.3	0.5	36.4	-2.4	1.19
28cf22	15.3	16.3	1	41.8	5.4	2.15
28cf23	16.3	17.1	0.8	47.2	5.4	5.06
28cf24	17.1	18.3	1.2	45.1	-2.1	3.52
28cf25	18.3	18.8	0.5	40.2	-4.8	1.25
28cf26	18.8	19.2	0.4	50.1	9.8	3.06
28cf27	19.2	19.8	0.6	41.3	-8.8	2.5
28cf28	19.8	20.4	0.6	42.8	1.5	1.88
28cf29	20.4	21.1	0.7	38.3	-4.5	1.21
28cf30	21.1	21.5	0.4	35.9	-2.5	0.3
28cf31	21.5	22.4	0.9	38.9	3.1	1.31
28cf32	22.4	22.6	0.2	36.4	-2.6	0
28cf33	22.6	22.8	0.2	38.0	1.6	0
28cf34	22.8	23.8	1	36.1	-2.0	0.58
28cf35	23.8	24.2	0.4	38.4	2.4	0.83
28cf36	24.2	25.5	1.3	42.5	4.0	3.86
28cf37	25.5	26.2	0.7	39.2	-3.2	1.24
28cf38	26.2	26.5	0.3	43.1	3.9	0.9
28cf39	26.5	26.8	0.3	38.0	-5.1	2.25
28cf40	26.8	28.5	1.7	44.1	6.1	3.16
28cf41	28.5	28.8	0.3	39.2	-4.9	0.32
28cf42	28.8	29.5	0.7	34.6	-4.6	0.91
28cf43	29.5	29.9	0.4	42.1	7.5	2.51
28cf44	29.9	30.1	0.2	38.0	-4.1	0
28cf45	30.1	30.4	0.3	45.7	7.8	4.01
28cf46	30.4	30.6	0.2	39.9	-5.9	0
28cf47	30.6	31.2	0.6	45.9	6.0	2.99
28cf48	31.2	31.4	0.2	43.5	-2.4	0
28cf49	31.4	31.8	0.4	39.9	-3.6	2.16
28cf50	31.8	32.4	0.6	43.5	3.6	2.84
28cf51	32.4	32.7	0.3	49.7	6.2	3.07
28cf52	32.7	32.9	0.2	43.2	-6.5	0
28cf53	32.9	33.1	0.2	40.2	-2.9	0
28cf54	33.1	34.2	1.1	43.3	3.1	1.83
28cf55	34.2	34.5	0.3	47.9	4.6	1.57
28cf56	34.5	34.7	0.2	39.3	-8.6	0
28cf57	34.7	35.3	0.6	47.6	8.4	3.58
28cf58	35.3	35.6	0.3	43.9	-3.7	1.41
28cf59	35.6	37.5	1.9	48.6	4.7	3.5
28cf60	37.5	39.6	2.1	45.2	-3.4	1.72
28cf61	39.6	40.8	1.2	48.5	3.3	2.66

28cf62	40.8	41	0.2	44.0	-4.6	0
28cf63	41	42.1	1.1	49.5	5.6	2.67
28cf64	42.1	44.2	2.1	58.0	8.5	4.59
29cf1	0	3	3	0.0		0
29cf2	3	4.3	1.3	39.2		1.37
29cf3	4.3	6.8	2.5	35.6	-3.6	1.07
29cf4	6.8	9.1	2.3	38.5	2.9	1.56
29cf5	9.1	9.3	0.2	36.7	-1.8	0
29cf6	9.3	13.1	3.8	38.7	2.0	1.4
29cf7	13.1	13.5	0.4	35.4	-3.3	0.64
29cf8	13.5	14.3	0.8	39.9	4.5	1.45
29cf9	14.3	14.5	0.2	44.9	4.9	0
29cf10	14.5	15.2	0.7	39.4	-5.5	1.18
29cf11	15.2	15.4	0.2	36.4	-3.0	0
29cf12	15.4	16.6	1.2	38.1	1.7	0.76
29cf13	16.6	17.2	0.6	35.7	-2.4	0.95
29cf14	17.2	17.6	0.4	38.1	2.4	0.84
29cf15	17.6	17.8	0.2	36.0	-2.1	0
29cf16	17.8	18.4	0.6	37.9	1.9	0.62
29cf17	18.4	18.6	0.2	36.3	-1.5	0
29cf18	18.6	19.5	0.9	38.5	2.2	1.21
29cf19	19.5	19.9	0.4	36.0	-2.5	1.08
29cf20	19.9	20.5	0.6	37.9	1.9	0.62
29cf21	20.5	20.7	0.2	36.3	-1.6	0
29cf22	20.7	20.9	0.2	38.1	1.7	0
29cf23	20.9	21.5	0.6	35.5	-2.5	0.88
29cf24	21.5	22.1	0.6	39.5	4.0	1.64
29cf25	22.1	22.3	0.2	42.0	2.5	0
29cf26	22.3	24.7	2.4	38.1	-3.9	1.07
29cf27	24.7	25	0.3	42.5	4.3	1.2
29cf28	25	25.4	0.4	36.6	-5.8	1.41
29cf29	25.4	25.8	0.4	39.7	3.1	1.67
29cf30	25.8	27.8	2	35.3	-4.4	1
29cf31	27.8	28	0.2	38.7	3.3	0
29cf32	28	29.8	1.8	34.8	-3.8	1
29cf33	29.8	30.1	0.3	37.5	2.6	0.41
29cf34	30.1	30.6	0.5	41.6	4.2	3.07
29cf35	30.6	30.8	0.2	37.6	-4.0	0
29cf36	30.8	31	0.2	41.8	4.2	0
29cf37	31	32	1	39.0	-2.8	1.54
29cf38	32	34.6	2.6	35.1	-3.9	1.19
29cf39	34.6	35.4	0.8	38.6	3.5	0.69
29cf40	35.4	38.2	2.8	35.6	-3.1	1.16
29cf41	38.2	40.9	2.7	38.9	3.3	1.65
29cf42	40.9	41.1	0.2	35.0	-3.9	0
29cf43	41.1	41.7	0.6	41.6	6.7	2.29
29cf44	41.7	42.1	0.4	47.3	5.6	1.35
29cf45	42.1	42.5	0.4	39.2	-8.1	1.88
29cf46	42.5	43	0.5	47.9	8.7	1.88
29cf47	43	43.5	0.5	40.6	-7.4	1.38
29cf48	43.5	44.1	0.6	49.8	9.2	2.71
29cf49	44.1	44.4	0.3	44.6	-5.2	0.34
29cf50	44.4	44.9	0.5	46.5	1.8	5.88
30cf1	0	3	3	0.0		0

30cf2	3	3.9	0.9	38.7		2.2
30cf3	3.9	4.7	0.8	42.5	3.8	1.43
30cf4	4.7	5.2	0.5	40.0	-2.5	0.61
30cf5	5.2	5.5	0.3	43.2	3.2	2.06
30cf6	5.5	5.9	0.4	38.2	-5.0	0.73
30cf7	5.9	6.4	0.5	35.6	-2.7	0.98
30cf8	6.4	6.6	0.2	38.3	2.7	0
30cf9	6.6	7	0.4	36.2	-2.0	0.76
30cf10	7	7.7	0.7	39.1	2.8	1.38
30cf11	7.7	8.3	0.6	36.5	-2.6	0.44
30cf12	8.3	8.9	0.6	38.7	2.2	1.89
30cf13	8.9	10.1	1.2	40.1	1.4	2.08
30cf14	10.1	10.4	0.3	43.5	3.5	1.64
30cf15	10.4	11.1	0.7	47.5	4.0	4.8
30cf16	11.1	11.4	0.3	39.5	-8.0	1.06
30cf17	11.4	12.1	0.7	45.2	5.7	5.57
30cf18	12.1	12.4	0.3	41.6	-3.6	3.84
30cf19	12.4	13.3	0.9	39.7	-2.0	2.51
30cf20	13.3	13.7	0.4	42.3	2.6	1.64
30cf21	13.7	14	0.3	38.2	-4.1	1.46
30cf22	14	14.2	0.2	36.6	-1.6	0
30cf23	14.2	14.9	0.7	42.1	5.5	4.13
30cf24	14.9	15.1	0.2	42.5	0.5	0
30cf25	15.1	15.3	0.2	39.5	-3.0	0
30cf26	15.3	15.5	0.2	36.9	-2.6	0
30cf27	15.5	15.7	0.2	37.4	0.5	0
30cf28	15.7	16.6	0.9	35.9	-1.5	0.65
30cf29	16.6	18.5	1.9	39.0	3.1	1.67
30cf30	18.5	18.7	0.2	35.3	-3.7	0
30cf31	18.7	19.8	1.1	37.8	2.5	1.14
30cf32	19.8	20.1	0.3	42.9	5.1	1.09
30cf33	20.1	20.8	0.7	39.2	-3.7	1.68
30cf34	20.8	21	0.2	43.5	4.3	0
30cf35	21	22.1	1.1	38.7	-4.8	1.71
30cf36	22.1	23.5	1.4	35.7	-3.0	0.65
30cf37	23.5	23.7	0.2	37.8	2.1	0
30cf38	23.7	24	0.3	36.0	-1.8	0.6
30cf39	24	24.3	0.3	38.0	2.0	1.1
30cf40	24.3	24.9	0.6	35.6	-2.4	1.36
30cf41	24.9	25.1	0.2	37.8	2.2	0
30cf42	25.1	25.5	0.4	36.6	-1.2	3.02
30cf43	25.5	26.1	0.6	44.1	7.5	1.32
30cf44	26.1	26.3	0.2	39.9	-4.2	0
30cf45	26.3	26.6	0.3	44.7	4.8	1.08
30cf46	26.6	27	0.4	36.4	-8.3	1.05
30cf47	27	27.4	0.4	42.2	5.8	0.44
30cf48	27.4	28.5	1.1	39.1	-3.1	1.36
30cf49	28.5	29	0.5	42.8	3.7	0.77
30cf50	29	29.8	0.8	38.7	-4.1	2.51
30cf51	29.8	31.1	1.3	43.3	4.6	2.34
30cf52	31.1	31.4	0.3	38.4	-4.9	1.82
30cf53	31.4	31.7	0.3	45.1	6.7	1.81
30cf54	31.7	32.1	0.4	38.4	-6.7	0.79
30cf55	32.1	32.5	0.4	47.4	9.0	2.65
30cf56	32.5	32.8	0.3	46.5	-0.9	4.54
30cf57	32.8	33.2	0.4	38.7	-7.8	3.15

30cf58	33.2	33.6	0.4	48.8	10.2	2.06
30cf59	33.6	33.8	0.2	42.4	-6.5	0
30cf60	33.8	34.4	0.6	50.0	7.6	1.75
30cf61	34.4	34.9	0.5	38.9	-11.1	1.26
30cf62	34.9	35.3	0.4	43.5	4.7	5.57
30cf63	35.3	36.8	1.5	47.6	4.0	4.65
30cf64	36.8	37	0.2	54.3	6.7	0
30cf65	37	37.3	0.3	52.8	-1.5	1.75
30cf66	37.3	38.4	1.1	42.9	-9.9	2.8
30cf67	38.4	38.6	0.2	34.5	-8.4	0
30cf68	38.6	38.9	0.3	44.2	9.7	2.72
30cf69	38.9	39.1	0.2	37.5	-6.8	0
30cf70	39.1	39.4	0.3	43.6	6.1	1.73
30cf71	39.4	39.6	0.2	36.8	-6.8	0
30cf72	39.6	39.9	0.3	44.4	7.5	6.97
30cf73	39.9	40.6	0.7	53.6	9.3	3.34
30cf74	40.6	41.4	0.8	49.0	-4.6	3.71
30cf75	41.4	41.8	0.4	59.6	10.6	1.89
30cf76	41.8	42.4	0.6	43.3	-16.4	6.44
30cf77	42.4	42.7	0.3	35.9	-7.4	1.63
30cf78	42.7	43	0.3	47.9	12.0	6.55
30cf79	43	43.3	0.3	40.5	-7.4	0
31cf1	0	3	3	0.0		0
31cf2	3	3.4	0.4	39.4		4.37
31cf3	3.4	4	0.6	36.3	-3.1	0.52
31cf4	4	4.2	0.2	37.3	1.0	0
31cf5	4.2	14.8	10.6	34.9	-2.3	0.98
31cf6	14.8	15.5	0.7	37.2	2.2	0.38
31cf7	15.5	16.9	1.4	35.2	-2.0	1.03
31cf8	16.9	17.3	0.4	39.0	3.9	0.51
31cf9	17.3	24	6.7	34.5	-4.5	0.98
31cf10	24	25.4	1.4	38.4	3.8	0.95
31cf11	25.4	26.5	1.1	35.3	-3.1	0.45
31cf12	26.5	27.9	1.4	38.1	2.9	1.06
31cf13	27.9	28.8	0.9	36.1	-2.1	0.79
31cf14	28.8	29	0.2	39.0	2.9	0
31cf15	29	30.1	1.1	43.6	4.6	2.17
31cf16	30.1	30.3	0.2	47.8	4.3	0
31cf17	30.3	30.5	0.2	39.7	-8.1	0
31cf18	30.5	30.8	0.3	45.9	6.2	2.7
31cf19	30.8	31.2	0.4	44.1	-1.8	1.78
31cf20	31.2	31.4	0.2	38.2	-5.9	0
31cf21	31.4	32.6	1.2	44.6	6.4	2.48
31cf22	32.6	33.4	0.8	40.7	-3.9	0.62
31cf23	33.4	33.9	0.5	44.3	3.6	1.19
31cf24	33.9	34.1	0.2	47.0	2.8	0
31cf25	34.1	34.3	0.2	43.6	-3.5	0
31cf26	34.3	34.8	0.5	40.3	-3.3	2.06
31cf27	34.8	36	1.2	43.1	2.8	1.57
31cf28	36	36.2	0.2	48.4	5.4	0
31cf29	36.2	37.2	1	43.1	-5.4	2.48
31cf30	37.2	37.5	0.3	39.6	-3.4	0.93
31cf31	37.5	37.9	0.4	43.5	3.9	1.79
31cf32	37.9	38.4	0.5	50.1	6.6	1.88
31cf33	38.4	39.4	1	54.3	4.2	1.18

31cf34	39.4	39.6	0.2	51.8	-2.5	0
31cf35	39.6	41.3	1.7	58.2	6.5	5.11
31cf36	41.3	41.7	0.4	52.3	-6.0	2.22
31cf37	41.7	42.3	0.6	54.1	1.8	1.21
32cf1	0	3	3	0.0		0
32cf2	3	4.1	1.1	39.6		1.52
32cf3	4.1	4.4	0.3	42.7	3.1	0.17
32cf4	4.4	6.1	1.7	39.2	-3.5	1.65
32cf5	6.1	7.2	1.1	36.5	-2.6	0.69
32cf6	7.2	7.6	0.4	38.6	2.1	1.62
32cf7	7.6	8.3	0.7	36.4	-2.2	0.85
32cf8	8.3	9.4	1.1	39.4	3.0	1.08
32cf9	9.4	9.7	0.3	42.9	3.5	1.09
32cf10	9.7	10.5	0.8	38.8	-4.1	1.54
32cf11	10.5	10.9	0.4	36.8	-2.0	0.42
32cf12	10.9	11.8	0.9	38.0	1.2	1.75
32cf13	11.8	12.8	1	36.3	-1.7	0.93
32cf14	12.8	13.1	0.3	37.3	1.1	0.41
32cf15	13.1	13.3	0.2	35.6	-1.7	0
32cf16	13.3	14.1	0.8	38.7	3.1	1.77
32cf17	14.1	14.3	0.2	36.8	-2.0	0
32cf18	14.3	15.1	0.8	39.2	2.4	1.15
32cf19	15.1	15.3	0.2	35.4	-3.8	0
32cf20	15.3	15.8	0.5	37.7	2.3	1.25
32cf21	15.8	20.9	5.1	34.9	-2.7	1.23
32cf22	20.9	21.1	0.2	39.2	4.3	0
32cf23	21.1	23.6	2.5	35.0	-4.2	0.92
32cf24	23.6	23.8	0.2	42.3	7.3	0
32cf25	23.8	25.1	1.3	38.4	-3.9	1.27
32cf26	25.1	25.4	0.3	36.1	-2.3	0.41
32cf27	25.4	25.6	0.2	37.7	1.6	0
32cf28	25.6	26.5	0.9	35.6	-2.1	0.57
32cf29	26.5	27.4	0.9	37.9	2.2	1.21
32cf30	27.4	29.2	1.8	36.1	-1.7	0.81
32cf31	29.2	29.4	0.2	38.0	1.8	0
32cf32	29.4	29.6	0.2	35.7	-2.3	0
32cf33	29.6	29.8	0.2	39.1	3.4	0
32cf34	29.8	30.9	1.1	35.6	-3.5	0.77
32cf35	30.9	32.1	1.2	39.6	4.0	1.51
32cf36	32.1	32.7	0.6	36.3	-3.3	1.62
32cf37	32.7	33.7	1	39.0	2.7	1.74
32cf38	33.7	35	1.3	35.4	-3.6	0.91
32cf39	35	35.6	0.6	38.5	3.1	1.07
32cf40	35.6	40.5	4.9	35.0	-3.5	1.24
32cf41	40.5	41	0.5	38.4	3.4	1.17
32cf42	41	41.2	0.2	36.5	-1.9	0
32cf43	41.2	41.8	0.6	38.4	1.9	0.56
33cf1	0	3	3	0.0		0
33cf2	3	3.3	0.3	37.1		1.9
33cf3	3.3	4.5	1.2	38.9	1.8	2.07
33cf4	4.5	5	0.5	41.7	2.8	2.3
33cf5	5	5.3	0.3	39.5	-2.2	0.93
33cf6	5.3	7.9	2.6	35.6	-3.9	0.94
33cf7	7.9	8.8	0.9	40.0	4.4	2.98

33cf8	8.8	9.1	0.3	35.6	-4.4	0.49
33cf9	9.1	11.5	2.4	38.4	2.8	1.29
33cf10	11.5	11.7	0.2	36.4	-2.0	0
33cf11	11.7	11.9	0.2	37.9	1.5	0
33cf12	11.9	15.4	3.5	35.4	-2.5	1.28
33cf13	15.4	16.1	0.7	38.3	2.9	1.09
33cf14	16.1	16.4	0.3	42.5	4.2	1.26
33cf15	16.4	17.9	1.5	38.6	-3.8	1.48
33cf16	17.9	18.5	0.6	35.7	-3.0	0.48
33cf17	18.5	19.4	0.9	38.4	2.7	1.92
33cf18	19.4	19.8	0.4	42.6	4.2	1.72
33cf19	19.8	20.5	0.7	38.5	-4.1	1.43
33cf20	20.5	20.7	0.2	43.1	4.6	0
33cf21	20.7	21.4	0.7	38.6	-4.5	1.71
33cf22	21.4	21.7	0.3	42.0	3.3	0.67
33cf23	21.7	22.4	0.7	36.0	-6.0	1.32
33cf24	22.4	25	2.6	38.0	2.0	1
33cf25	25	25.3	0.3	36.0	-2.1	0.34
33cf26	25.3	25.9	0.6	39.4	3.4	1.17
33cf27	25.9	26.5	0.6	42.5	3.1	0.96
33cf28	26.5	26.7	0.2	35.3	-7.2	0
33cf29	26.7	27.1	0.4	41.1	5.8	1.8
33cf30	27.1	27.3	0.2	38.7	-2.4	0
33cf31	27.3	27.9	0.6	35.1	-3.6	1.47
33cf32	27.9	28.7	0.8	38.9	3.8	1.44
33cf33	28.7	29	0.3	41.1	2.3	1.5
33cf34	29	29.9	0.9	48.0	6.9	5.18
33cf35	29.9	30.1	0.2	40.0	-8.0	0
33cf36	30.1	30.7	0.6	44.9	4.9	0.67
33cf37	30.7	31.2	0.5	47.3	2.4	0.78
33cf38	31.2	31.7	0.5	40.6	-6.7	3.32
33cf39	31.7	32	0.3	38.1	-2.5	0.15
33cf40	32	33.1	1.1	44.6	6.4	4.65
33cf41	33.1	33.3	0.2	35.1	-9.4	0
33cf42	33.3	33.8	0.5	46.0	10.9	4.98
33cf43	33.8	34.2	0.4	52.8	6.8	2.9
33cf44	34.2	34.5	0.3	44.0	-8.8	0
34cf1	0	3	3	0.0		0
34cf2	3	3.8	0.8	43.8		1.99
34cf3	3.8	4.4	0.6	38.9	-4.9	0.91
34cf4	4.4	5.5	1.1	35.6	-3.3	0.92
34cf5	5.5	6	0.5	38.4	2.8	1.04
34cf6	6	6.6	0.6	41.6	3.2	0.56
34cf7	6.6	6.9	0.3	48.0	6.4	0.54
34cf8	6.9	8.1	1.2	42.4	-5.6	1.31
34cf9	8.1	9	0.9	40.1	-2.3	0.93
34cf10	9	9.6	0.6	42.9	2.9	2.44
34cf11	9.6	9.9	0.3	40.2	-2.7	0.21
34cf12	9.9	10.4	0.5	48.4	8.2	1.54
34cf13	10.4	11.1	0.7	44.4	-4.0	0.86
34cf14	11.1	11.4	0.3	47.6	3.3	1.34
34cf15	11.4	12.5	1.1	43.1	-4.5	1.93
34cf16	12.5	13	0.5	48.4	5.3	2.85
34cf17	13	13.2	0.2	44.0	-4.4	0
34cf18	13.2	13.9	0.7	48.4	4.4	1.19

34cf19	13.9	14.8	0.9	56.3	7.9	3.44
34cf20	14.8	15.5	0.7	48.6	-7.6	2
34cf21	15.5	16.4	0.9	38.8	-9.9	1.77
34cf22	16.4	17.8	1.4	36.3	-2.5	0.62
34cf23	17.8	18.8	1	38.9	2.6	1.2
34cf24	18.8	19	0.2	34.8	-4.1	0
34cf25	19	19.4	0.4	40.0	5.3	0.43
34cf26	19.4	19.6	0.2	41.9	1.9	0
34cf27	19.6	19.8	0.2	39.8	-2.1	0
34cf28	19.8	20.1	0.3	43.1	3.3	1.59
34cf29	20.1	20.6	0.5	49.1	6.0	3.16
34cf30	20.6	21.2	0.6	38.9	-10.2	1.52
34cf31	21.2	21.4	0.2	42.1	3.2	0
34cf32	21.4	21.7	0.3	39.5	-2.5	0.79
34cf33	21.7	23.7	2	43.1	3.6	1.53
34cf34	23.7	25.6	1.9	39.1	-4.1	1.37
34cf35	25.6	26.1	0.5	35.8	-3.3	0.59
34cf36	26.1	26.3	0.2	37.2	1.4	0
34cf37	26.3	27	0.7	35.6	-1.6	0.98
34cf38	27	27.4	0.4	38.3	2.6	0.79
34cf39	27.4	27.6	0.2	42.4	4.2	0
34cf40	27.6	28.9	1.3	38.1	-4.3	1.08
34cf41	28.9	29.3	0.4	41.8	3.7	0.41
34cf42	29.3	30.6	1.3	38.5	-3.3	1.25
34cf43	30.6	35.4	4.8	34.6	-3.9	1.15
34cf44	35.4	36.1	0.7	38.6	4.0	1.75
34cf45	36.1	36.7	0.6	36.2	-2.4	0.31
34cf46	36.7	37.2	0.5	38.9	2.7	0.58
34cf47	37.2	37.5	0.3	42.3	3.4	0.83
34cf48	37.5	37.9	0.4	39.0	-3.3	0.83
34cf49	37.9	39.9	2	42.3	3.2	1.77
34cf50	39.9	40.5	0.6	37.3	-4.9	1.14
34cf51	40.5	40.9	0.4	36.1	-1.2	0.7
34cf52	40.9	41.4	0.5	37.5	1.4	0.69
34cf53	41.4	41.9	0.5	35.6	-1.9	1.25
34cf54	41.9	42.7	0.8	37.5	1.9	0.95
34cf55	42.7	43.1	0.4	35.5	-2.0	0.65
34cf56	43.1	43.4	0.3	39.3	3.8	1.13
34cf57	43.4	43.8	0.4	43.7	4.4	4.09
34cf58	43.8	44.2	0.4	49.3	5.6	0.95
34cf59	44.2	44.5	0.3	43.1	-6.1	1.51
34cf60	44.5	44.7	0.2	40.0	-3.2	0
34cf61	44.7	44.9	0.2	48.7	8.7	0
34cf62	44.9	45.2	0.3	43.3	-5.4	0
35cf1	0	3	3	0.0		0
35cf2	3	3.6	0.6	39.7		3.14
35cf3	3.6	4.1	0.5	44.3	4.6	3.45
35cf4	4.1	4.5	0.4	39.7	-4.6	0.84
35cf5	4.5	4.8	0.3	42.3	2.5	1.17
35cf6	4.8	5.3	0.5	45.7	3.4	3.6
35cf7	5.3	5.7	0.4	38.2	-7.5	1.47
35cf8	5.7	6.4	0.7	43.6	5.5	1.67
35cf9	6.4	7.1	0.7	47.8	4.2	3.73
35cf10	7.1	8.3	1.2	42.1	-5.7	2.28
35cf11	8.3	8.7	0.4	39.3	-2.8	1.41

35cf12	8.7	9.1	0.4	45.3	6.0	3.04
35cf13	9.1	9.6	0.5	39.1	-6.2	0.88
35cf14	9.6	10.1	0.5	44.2	5.1	1.56
35cf15	10.1	10.3	0.2	48.4	4.2	0
35cf16	10.3	10.9	0.6	44.0	-4.4	2.43
35cf17	10.9	11.8	0.9	38.1	-6.0	1.44
35cf18	11.8	12.7	0.9	36.0	-2.0	0.48
35cf19	12.7	13	0.3	38.7	2.7	1.05
35cf20	13	13.4	0.4	43.6	4.9	2.4
35cf21	13.4	13.7	0.3	39.6	-3.9	1.22
35cf22	13.7	14.4	0.7	41.9	2.3	1.63
35cf23	14.4	15.4	1	39.1	-2.8	1.05
35cf24	15.4	19.2	3.8	42.8	3.7	2.18
35cf25	19.2	19.8	0.6	40.4	-2.5	1.42
35cf26	19.8	20.3	0.5	41.5	1.1	0.54
35cf27	20.3	22.4	2.1	38.9	-2.6	1.71
35cf28	22.4	22.6	0.2	36.7	-2.2	0
35cf29	22.6	22.8	0.2	39.2	2.5	0
35cf30	22.8	23.2	0.4	42.0	2.7	0.81
35cf31	23.2	24.2	1	38.8	-3.1	1.37
35cf32	24.2	24.5	0.3	36.1	-2.7	0.31
35cf33	24.5	25.2	0.7	37.5	1.4	0.93
35cf34	25.2	26.4	1.2	42.0	4.5	1.51
35cf35	26.4	26.9	0.5	40.6	-1.4	1.64
35cf36	26.9	28.1	1.2	42.9	2.3	1.26
35cf37	28.1	28.3	0.2	39.3	-3.6	0
35cf38	28.3	29	0.7	43.3	4.1	1.59
35cf39	29	29.2	0.2	40.4	-2.9	0
35cf40	29.2	29.6	0.4	49.2	8.8	1.82
36cf1	0	3	3	0.0		0
36cf2	3	3.7	0.7	37.5		0.71
36cf3	3.7	4.1	0.4	35.8	-1.7	1.09
36cf4	4.1	4.6	0.5	38.0	2.2	0.68
36cf5	4.6	5.3	0.7	35.7	-2.3	1.21
36cf6	5.3	6	0.7	39.4	3.7	1.24
36cf7	6	6.3	0.3	36.5	-2.9	0.41
36cf8	6.3	6.8	0.5	39.1	2.6	0.95
36cf9	6.8	7.1	0.3	42.3	3.2	0.58
36cf10	7.1	7.6	0.5	38.2	-4.0	1.62
36cf11	7.6	8	0.4	42.8	4.6	0.92
36cf12	8	9.9	1.9	38.1	-4.6	2.4
36cf13	9.9	10.4	0.5	35.4	-2.7	1.17
36cf14	10.4	10.8	0.4	38.6	3.1	2.31
36cf15	10.8	11.8	1	35.7	-2.9	0.42
36cf16	11.8	12.4	0.6	37.7	2.0	1.34
36cf17	12.4	12.6	0.2	35.5	-2.1	0
36cf18	12.6	14.3	1.7	37.3	1.8	1.61
36cf19	14.3	15.6	1.3	35.6	-1.8	0.98
36cf20	15.6	16	0.4	39.1	3.6	1.28
36cf21	16	16.2	0.2	42.4	3.3	0
36cf22	16.2	17.2	1	39.4	-3.0	1.66
36cf23	17.2	17.7	0.5	36.6	-2.8	2.36
36cf24	17.7	18.2	0.5	39.4	2.8	0.72
36cf25	18.2	18.4	0.2	42.3	2.9	0
36cf26	18.4	19.1	0.7	38.3	-4.0	1.31

36cf27	19.1	19.3	0.2	36.5	-1.8	0
36cf28	19.3	19.5	0.2	38.6	2.1	0
36cf29	19.5	19.9	0.4	44.7	6.1	1.6
36cf30	19.9	20.5	0.6	39.8	-4.9	1.72
36cf31	20.5	21	0.5	42.3	2.5	0.5
36cf32	21	21.6	0.6	39.6	-2.7	3.49
36cf33	21.6	21.8	0.2	45.1	5.5	0
36cf34	21.8	22.4	0.6	38.8	-6.2	1.25
36cf35	22.4	22.7	0.3	41.9	3.1	0.26
36cf36	22.7	22.9	0.2	38.1	-3.9	0
36cf37	22.9	23.6	0.7	42.6	4.5	5.47
36cf38	23.6	23.9	0.3	40.0	-2.6	0.9
36cf39	23.9	24.1	0.2	41.8	1.9	0
36cf40	24.1	24.3	0.2	35.9	-5.9	0
36cf41	24.3	24.5	0.2	43.1	7.2	0
36cf42	24.5	25.6	1.1	39.6	-3.5	1.35
36cf43	25.6	25.8	0.2	36.0	-3.6	0
36cf44	25.8	26.4	0.6	38.5	2.4	1.33
36cf45	26.4	26.6	0.2	36.4	-2.0	0
36cf46	26.6	28.4	1.8	38.8	2.3	1.85
36cf47	28.4	28.6	0.2	36.0	-2.8	0
36cf48	28.6	29.7	1.1	38.9	2.9	3
36cf49	29.7	31.6	1.9	35.8	-3.1	1.21
36cf50	31.6	31.8	0.2	38.4	2.6	0
36cf51	31.8	32	0.2	47.4	9.0	0
36cf52	32	33	1	38.3	-9.2	1.03
36cf53	33	33.3	0.3	43.6	5.3	1.01
36cf54	33.3	33.9	0.6	40.4	-3.2	1.79
37cf1	0	3	3	0.0		0
37cf2	3	3.5	0.5	43.0		1.93
37cf3	3.5	3.9	0.4	38.7	-4.3	2.5
37cf4	3.9	4.2	0.3	43.7	5.1	2.45
37cf5	4.2	4.5	0.3	38.8	-4.9	2.25
37cf6	4.5	5.4	0.9	44.7	5.9	2.53
37cf7	5.4	5.7	0.3	37.6	-7.0	1.39
37cf8	5.7	8.2	2.5	34.6	-3.0	1.17
37cf9	8.2	11.1	2.9	38.6	4.0	1.37
37cf10	11.1	11.4	0.3	35.4	-3.2	1.08
37cf11	11.4	11.7	0.3	38.3	2.9	1.8
37cf12	11.7	11.9	0.2	42.3	4.0	0
37cf13	11.9	12.7	0.8	40.2	-2.1	1.74
37cf14	12.7	13	0.3	41.6	1.3	0.22
37cf15	13	13.5	0.5	39.3	-2.2	1.67
37cf16	13.5	13.7	0.2	36.4	-2.9	0
37cf17	13.7	13.9	0.2	38.7	2.3	0
37cf18	13.9	14.2	0.3	43.4	4.7	1.63
37cf19	14.2	14.4	0.2	39.2	-4.2	0
37cf20	14.4	15.4	1	36.4	-2.9	0.96
37cf21	15.4	16.4	1	39.4	3.1	1.34
37cf22	16.4	16.9	0.5	36.9	-2.6	0.56
37cf23	16.9	17.2	0.3	38.3	1.4	1.19
37cf24	17.2	17.9	0.7	43.6	5.4	2.9
37cf25	17.9	19	1.1	40.0	-3.6	1.38
37cf26	19	19.2	0.2	43.7	3.7	0
37cf27	19.2	19.8	0.6	39.5	-4.2	1.76

37cf28	19.8	20.6	0.8	36.5	-3.0	0.71
37cf29	20.6	21.3	0.7	37.4	0.9	1.97
37cf30	21.3	24.8	3.5	35.1	-2.3	1.13
37cf31	24.8	25.9	1.1	38.2	3.1	1.21
37cf32	25.9	26.2	0.3	43.6	5.3	1.33
37cf33	26.2	26.7	0.5	48.6	5.0	2.47
37cf34	26.7	27.5	0.8	42.8	-5.8	2.2
37cf35	27.5	27.8	0.3	55.4	12.6	2.01
37cf36	27.8	28.9	1.1	48.2	-7.2	4.39
37cf37	28.9	29.1	0.2	57.2	9.0	0
37cf38	29.1	29.6	0.5	45.4	-11.7	4.1
37cf39	29.6	31.2	1.6	39.9	-5.5	1.56
37cf40	31.2	31.4	0.2	42.7	2.8	0
37cf41	31.4	31.6	0.2	47.4	4.6	0
37cf42	31.6	32	0.4	45.0	-2.4	1.34
37cf43	32	32.5	0.5	38.7	-6.3	1.27
37cf44	32.5	32.8	0.3	47.1	8.5	3.04
37cf45	32.8	33	0.2	42.4	-4.7	0
37cf46	33	33.3	0.3	49.1	6.6	3.12
37cf47	33.3	33.8	0.5	54.2	5.1	2.11
37cf48	33.8	34	0.2	52.5	-1.6	0
38cf1	0	3	3	0.0		0
38cf2	3	3.6	0.6	46.0		4.08
38cf3	3.6	5.4	1.8	48.8	2.8	3.2
38cf4	5.4	5.7	0.3	43.5	-5.3	0.59
38cf5	5.7	5.9	0.2	49.4	5.9	0
38cf6	5.9	9	3.1	34.9	-14.5	1.2
38cf7	9	9.3	0.3	37.7	2.8	0.34
38cf8	9.3	13.9	4.6	34.3	-3.4	1
38cf9	13.9	14.3	0.4	38.0	3.6	0.89
38cf10	14.3	14.7	0.4	36.3	-1.7	1.18
38cf11	14.7	15.3	0.6	38.9	2.6	1.18
38cf12	15.3	15.5	0.2	41.2	2.4	0
38cf13	15.5	15.7	0.2	38.5	-2.7	0
38cf14	15.7	16	0.3	35.2	-3.3	1.36
38cf15	16	16.8	0.8	39.4	4.2	1.73
38cf16	16.8	17.2	0.4	36.5	-2.9	0.47
38cf17	17.2	18	0.8	39.6	3.1	1.31
38cf18	18	18.6	0.6	43.0	3.4	2.71
38cf19	18.6	19.2	0.6	40.1	-2.9	0.82
38cf20	19.2	19.9	0.7	42.3	2.3	1.52
38cf21	19.9	20.1	0.2	40.1	-2.2	0
38cf22	20.1	20.3	0.2	43.2	3.1	0
38cf23	20.3	20.5	0.2	48.5	5.3	0
38cf24	20.5	20.8	0.3	44.3	-4.1	0.93
38cf25	20.8	21.1	0.3	48.5	4.2	1.47
38cf26	21.1	21.4	0.3	44.1	-4.4	1.24
38cf27	21.4	21.7	0.3	47.5	3.4	1.58
38cf28	21.7	21.9	0.2	42.2	-5.3	0
38cf29	21.9	22.4	0.5	38.8	-3.4	0.54
38cf30	22.4	23.3	0.9	42.6	3.8	1.59
38cf31	23.3	23.7	0.4	50.9	8.3	1.73
38cf32	23.7	24	0.3	41.3	-9.6	3.23
38cf33	24	24.5	0.5	50.3	9.0	3.28
38cf34	24.5	25	0.5	46.6	-3.7	3.97

38cf35	25	25.4	0.4	58.5	12.0	4.13
38cf36	25.4	25.9	0.5	48.1	-10.5	3.63
38cf37	25.9	26.5	0.6	43.6	-4.5	2.17
38cf38	26.5	26.7	0.2	64.9	21.3	0
38cf39	26.7	26.9	0.2	51.6	-13.3	0
Xcf1	0	0.5	0.5	57.2		3.72
Xcf2	0.5	1.5	1	49.8	-7.4	2.56
Xcf3	1.5	2.4	0.9	44.3	-5.5	2.65
Xcf4	2.4	2.8	0.4	40.4	-4.0	0.56
Xcf5	2.8	3.1	0.3	41.3	0.9	0.22
Xcf6	3.1	3.8	0.7	40.8	-0.5	1.32
Xcf7	3.8	4.2	0.4	43.3	2.5	1.15
Xcf8	4.2	4.5	0.3	48.3	5.0	1.6
Xcf9	4.5	4.9	0.4	42.1	-6.2	1.09
Xcf10	4.9	5.2	0.3	40.0	-2.1	0.21
Xcf11	5.2	5.5	0.3	43.5	3.5	0.99
Xcf12	5.5	6	0.5	39.7	-3.8	0.56
Xcf13	6	6.9	0.9	47.8	8.1	2.58
Xcf14	6.9	7.4	0.5	43.8	-4.0	1.15
Xcf15	7.4	7.6	0.2	39.4	-4.4	0
Xcf16	7.6	8.3	0.7	41.9	2.5	1.52
Xcf17	8.3	8.6	0.3	39.8	-2.1	0.27
Xcf18	8.6	9.2	0.6	42.1	2.3	1.91
Xcf19	9.2	9.5	0.3	48.1	6.0	1.01
Xcf20	9.5	10.2	0.7	43.9	-4.2	1.5
Xcf21	10.2	10.4	0.2	47.6	3.7	0
Xcf22	10.4	11.4	1	39.3	-8.3	1.25
Xcf23	11.4	12	0.6	42.9	3.6	1.35
Xcf24	12	12.7	0.7	40.2	-2.7	1.29
Xcf25	12.7	13.1	0.4	41.9	1.6	0.74
Xcf26	13.1	13.3	0.2	39.9	-2.0	0
Xcf27	13.3	13.5	0.2	44.5	4.7	0
Xcf28	13.5	13.9	0.4	46.8	2.3	3.57
Xcf29	13.9	14.1	0.2	42.4	-4.4	0
Xcf30	14.1	14.8	0.7	39.9	-2.5	1.78
Xcf31	14.8	16	1.2	43.1	3.1	1.71
Xcf32	16	16.5	0.5	39.6	-3.4	2.26
Xcf33	16.5	16.9	0.4	36.7	-3.0	0.32
Xcf34	16.9	17.1	0.2	37.4	0.7	0
Xcf35	17.1	17.4	0.3	35.3	-2.2	1.12
Xcf36	17.4	18.1	0.7	41.8	6.6	1.47
Xcf37	18.1	19.8	1.7	38.9	-3.0	1.77
Xcf38	19.8	20.2	0.4	43.1	4.2	2.28
Xcf39	20.2	20.4	0.2	38.6	-4.5	0
Xcf40	20.4	20.6	0.2	42.6	4.0	0
Xcf41	20.6	21.7	1.1	38.9	-3.7	1.65
Xcf42	21.7	23.6	1.9	36.2	-2.8	1.12
Xcf43	23.6	23.8	0.2	37.5	1.3	0
Xcf44	23.8	24.9	1.1	35.6	-1.9	0.81
Xcf45	24.9	25.8	0.9	39.2	3.5	1.21
Xcf46	25.8	26.4	0.6	41.5	2.3	0.82
Xcf47	26.4	26.7	0.3	39.2	-2.3	0.59
Xcf48	26.7	28.6	1.9	36.2	-3.0	0.79
Xcf49	28.6	28.8	0.2	37.2	1.0	0
Xcf50	28.8	29.4	0.6	36.3	-0.9	0.43

Xcf51	29.4	30.9	1.5	37.5	1.2	0.72
Xcf52	30.9	31.4	0.5	35.9	-1.6	0.74
Xcf53	31.4	33.3	1.9	39.7	3.8	1.61
Xcf54	33.3	33.8	0.5	43.9	4.2	1.44
Xcf55	33.8	35	1.2	49.6	5.8	2.12
Xcf56	35	35.6	0.6	39.5	-10.1	1.79
Xcf57	35.6	35.9	0.3	43.5	4.0	1.09
Xcf58	35.9	37.5	1.6	39.3	-4.2	1.35
Xcf59	37.5	38.1	0.6	42.1	2.9	0.51
Xcf60	38.1	38.7	0.6	40.3	-1.8	0.98
Xcf61	38.7	38.9	0.2	35.9	-4.4	0
Xcf62	38.9	39.1	0.2	42.3	6.5	0
Xcf63	39.1	39.9	0.8	39.3	-3.0	1.5
Xcf64	39.9	40.3	0.4	42.5	3.3	1.41
Xcf65	40.3	40.6	0.3	39.6	-3.0	0.84
Xcf66	40.6	41.9	1.3	43.7	4.2	2.81
Xcf67	41.9	42.3	0.4	49.4	5.7	2.38
Xcf68	42.3	42.6	0.3	42.5	-6.9	2.2
Xcf69	42.6	44.8	2.2	40.0	-2.5	0.99
Xcf70	44.8	45.2	0.4	44.7	4.8	2.2
Xcf71	45.2	46.1	0.9	38.8	-6.0	1.08
Xcf72	46.1	46.6	0.5	42.5	3.8	2.33
Xcf73	46.6	48.7	2.1	39.6	-2.9	1.53
Xcf74	48.7	51.6	2.9	0.0	-39.6	0
Xcf75	51.6	53.5	1.9	39.6	39.6	2.42
Xcf76	53.5	53.7	0.2	42.1	2.6	0
Xcf77	53.7	56	2.3	38.3	-3.8	1.01
Xcf78	56	56.7	0.7	47.0	8.6	2.68
Xcf79	56.7	57	0.3	41.3	-5.6	0.84
Xcf80	57	57.4	0.4	37.4	-3.9	0.6
Xcf81	57.4	57.6	0.2	42.3	4.9	0
Xcf82	57.6	57.9	0.3	42.2	-0.2	2.58
Xcf83	57.9	58.1	0.2	36.2	-5.9	0
Xcf84	58.1	58.4	0.3	38.9	2.7	1.44
Xcf85	58.4	58.6	0.2	46.4	7.5	0
Xcf86	58.6	58.8	0.2	40.7	-5.8	0
Xcf87	58.8	59.3	0.5	45.6	4.9	1.54
Xcf88	59.3	62.8	3.5	38.6	-7.0	1.09
Xcf89	62.8	63.1	0.3	36.0	-2.6	0.51
Xcf90	63.1	64.8	1.7	38.1	2.1	0.87
Xcf91	64.8	65.7	0.9	36.8	-1.3	0.93
Xcf92	65.7	65.9	0.2	37.8	0.9	0
Xcf93	65.9	66.2	0.3	36.9	-0.9	0.21
Xcf94	66.2	66.6	0.4	37.4	0.5	0.7
Xcf95	66.6	68.1	1.5	36.9	-0.5	0.82
Xcf96	68.1	68.5	0.4	37.3	0.4	0.74
Xcf97	68.5	68.9	0.4	35.7	-1.6	0.56
Xcf98	68.9	69.2	0.3	37.6	1.9	0.61
Xcf99	69.2	69.4	0.2	36.5	-1.1	0
Xcf100	69.4	69.9	0.5	37.7	1.2	1.22
Xcf101	69.9	72	2.1	36.6	-1.1	0.79
Xcf102	72	72.6	0.6	37.3	0.6	0.75
Xcf103	72.6	72.8	0.2	36.5	-0.8	0
Xcf104	72.8	73.1	0.3	38.1	1.6	0.44
Xcf105	73.1	74.1	1	35.8	-2.3	0.7
Xcf106	74.1	74.6	0.5	37.6	1.9	0.74

Xcf107	74.6	74.9	0.3	36.5	-1.1	0.39
Xcf108	74.9	75.7	0.8	37.4	0.9	0.45
Xcf109	75.7	76.3	0.6	36.3	-1.1	0.45
Xcf110	76.3	81.8	5.5	39.1	2.8	1.46
Xcf111	81.8	82	0.2	36.8	-2.3	0
Xcf112	82	82.3	0.3	37.1	0.3	0.14
Xcf113	82.3	82.6	0.3	36.5	-0.6	0.16
Xcf114	82.6	83.7	1.1	38.7	2.1	2.44
Xcf115	83.7	83.9	0.2	36.2	-2.4	0
Xcf116	83.9	84.1	0.2	41.9	5.6	0
Xcf117	84.1	84.5	0.4	46.1	4.2	2.82
Xcf118	84.5	84.7	0.2	40.6	-5.5	0
Xcf119	84.7	84.9	0.2	43.2	2.6	0
Xcf120	84.9	85.1	0.2	38.7	-4.5	0
Xcf121	85.1	85.3	0.2	35.9	-2.8	0
Xcf122	85.3	86.1	0.8	39.5	3.6	1.66
Xcf123	86.1	87.6	1.5	41.9	2.4	1.61
Xcf124	87.6	89.2	1.6	39.2	-2.7	1.64
Xcf125	89.2	89.7	0.5	36.3	-3.0	0.52
Xcf126	89.7	90	0.3	37.2	0.9	0.13
Xcf127	90	90.7	0.7	36.0	-1.2	0.45
Xcf128	90.7	91.4	0.7	39.2	3.2	1.98
Xcf129	91.4	91.7	0.3	42.4	3.2	1.22
Xcf130	91.7	92.1	0.4	38.3	-4.1	1.03
Xcf131	92.1	92.3	0.2	36.8	-1.6	0
Xcf132	92.3	93.7	1.4	39.0	2.2	1.58
Xcf133	93.7	95.5	1.8	43.7	4.7	1.83
Xcf134	95.5	96.3	0.8	40.2	-3.5	0.99
Xcf135	96.3	97.3	1	36.4	-3.9	0.58
Xcf136	97.3	99.2	1.9	39.0	2.6	2
Xcf137	99.2	99.5	0.3	36.8	-2.2	0.18
Xcf138	99.5	99.8	0.3	37.3	0.5	0.22
Xcf139	99.8	100.9	1.1	35.5	-1.8	0.72
Xcf140	100.9	101.5	0.6	37.2	1.7	0.48
Xcf141	101.5	103	1.5	36.0	-1.1	0.74
Xcf142	103	103.9	0.9	38.3	2.2	1.37
Xcf143	103.9	104.4	0.5	46.4	8.1	2.58
Xcf144	104.4	106.6	2.2	39.4	-7.0	1.18
Xcf145	106.6	107.5	0.9	42.5	3.1	1.39
Xcf146	107.5	107.8	0.3	40.7	-1.8	0.28
Xcf147	107.8	110.4	2.6	42.7	2.0	1.76
Xcf148	110.4	110.6	0.2	40.1	-2.6	0
Xcf149	110.6	110.9	0.3	43.8	3.7	2.22
Xcf150	110.9	111.8	0.9	40.2	-3.7	0.75
Xcf151	111.8	112.3	0.5	41.8	1.7	0.7
Xcf152	112.3	113.3	1	40.2	-1.6	0.92
Xcf153	113.3	113.8	0.5	42.1	1.8	1.86
Xcf154	113.8	117.5	3.7	38.2	-3.9	1.43
Xcf155	117.5	118.1	0.6	42.6	4.5	1.16
Xcf156	118.1	118.4	0.3	38.9	-3.7	1.15
Xcf157	118.4	118.9	0.5	36.5	-2.4	0.28
Xcf158	118.9	119.6	0.7	39.1	2.6	0.99
Xcf159	119.6	120.6	1	43.0	3.9	1.71
Xcf160	120.6	121.1	0.5	48.7	5.7	3.04
Xcf161	121.1	121.5	0.4	43.1	-5.6	1.69
Xcf162	121.5	122.2	0.7	48.4	5.4	1.92

Xcf163	122.2	123.1	0.9	42.3	-6.2	1.94
Xcf164	123.1	123.6	0.5	37.1	-5.1	1.14
Xcf165	123.6	124.2	0.6	44.9	7.7	1.44
Xcf166	124.2	125.3	1.1	56.0	11.1	4.58
Xcf167	125.3	125.9	0.6	48.2	-7.8	3.05
Xcf168	125.9	126.4	0.5	42.9	-5.3	1.69
Xcf169	126.4	126.9	0.5	37.6	-5.3	1.04
