# Supplementary material for: The evolution of isochore patterns in vertebrate genomes
Source: BMC Genomics. 2009 Apr 3;10:146. doi: 10.1186/1471-2164-10-146 (PMC2678159; doi:10.1186/1471-2164-10-146)
Supplement: Additional File 6 — Overview of mouse chromosomes. The color-coded maps show the compositional patterns of the mouse chromosomes. [file 1471-2164-10-146-S6.pdf]

Additional Table T3. Coordinates, sizes, GC levels and GC standard deviations (SD) of the mouse isochores. ΔGC indicates the difference in GC between subsequent isochores.

Isochore	Start	End	Length, Mb	GC, %	Δ GC	SD (w=100kb)
1Mm1	0	3	3	0.0		0
1Mm2	2.9	4.4	1.5	38.1		2.26
1Mm3	4.4	4.8	0.4	41.4	3.4	0.4
1Mm4	4.8	12.7	7.9	39.3	-2.1	1.11
1Mm5	12.7	13.7	1	43.5	4.2	1.15
1Mm6	13.7	16	2.3	40.5	-3.0	0.85
1Mm7	16	16.3	0.3	43.2	2.7	0.77
1Mm8	16.3	16.5	0.2	40.0	-3.2	0
1Mm9	16.5	16.9	0.4	41.8	1.9	0.85
1Mm10	16.9	18.1	1.2	39.1	-2.7	1.24
1Mm11	18.1	18.3	0.2	36.5	-2.6	0
1Mm12	18.3	20.7	2.4	38.9	2.4	1.07
1Mm13	20.7	21.4	0.7	43.6	4.7	1.21
1Mm14	21.4	23.6	2.2	39.1	-4.5	1.11
1Mm15	23.6	24	0.4	41.7	2.6	0.62
1Mm16	24	27.4	3.4	38.2	-3.5	1.56
1Mm17	27.4	30.3	2.9	36.5	-1.7	0.9
1Mm18	30.3	33.5	3.2	38.8	2.4	1.3
1Mm19	33.5	34.5	1	43.4	4.6	0.88
1Mm20	34.5	35	0.5	46.2	2.7	0.75
1Mm21	35	36.1	1.1	44.0	-2.2	1.44
1Mm22	36.1	36.9	0.8	47.9	3.9	2.24
1Mm23	36.9	37.3	0.4	44.4	-3.6	1.28
1Mm24	37.3	37.8	0.5	46.2	1.8	0.93
1Mm25	37.8	39.3	1.5	43.9	-2.2	1.7
1Mm26	39.3	39.8	0.5	46.1	2.2	1.36
1Mm27	39.8	41.1	1.3	43.6	-2.5	1.45
1Mm28	41.1	42.4	1.3	39.3	-4.3	1.26
1Mm29	42.4	43.9	1.5	42.9	3.7	1.02
1Mm30	43.9	47.4	3.5	39.1	-3.9	1.16
1Mm31	47.4	47.6	0.2	36.7	-2.4	0
1Mm32	47.6	47.9	0.3	37.6	1.0	0.44
1Mm33	47.9	48.1	0.2	36.2	-1.4	0
1Mm34	48.1	48.8	0.7	37.4	1.1	0.23
1Mm35	48.8	50.9	2.1	36.3	-1.1	0.58
1Mm36	50.9	51.3	0.4	38.2	1.9	0.45
1Mm37	51.3	52.9	1.6	41.6	3.4	1.04
1Mm38	52.9	54.8	1.9	40.4	-1.2	0.74
1Mm39	54.8	55.3	0.5	43.2	2.8	1.18
1Mm40	55.3	57	1.7	39.7	-3.6	1.51
1Mm41	57	59.7	2.7	42.8	3.1	1.59
1Mm42	59.7	62.6	2.9	40.1	-2.7	1.38
1Mm43	62.6	65.2	2.6	42.5	2.4	1.28
1Mm44	65.2	68.1	2.9	38.9	-3.6	1
1Mm45	68.1	69.1	1	35.9	-3.0	0.62
1Mm46	69.1	71.5	2.4	38.8	2.9	0.91
1Mm47	71.5	73.9	2.4	43.3	4.5	1.56
1Mm48	73.9	74.5	0.6	48.5	5.2	2.51
1Mm49	74.5	74.8	0.3	43.8	-4.8	2.78
1Mm50	74.8	75.6	0.8	48.9	5.1	2.67

1Mm51	75.6	76.1	0.5	43.4	-5.5	1.07
1Mm52	76.1	77	0.9	39.9	-3.5	0.56
1Mm53	77	78.9	1.9	42.0	2.1	1.28
1Mm54	78.9	79.6	0.7	39.9	-2.0	0.88
1Mm55	79.6	80.2	0.6	42.8	2.9	0.94
1Mm56	80.2	82.1	1.9	38.9	-3.9	0.83
1Mm57	82.1	83.2	1.1	42.2	3.3	1
1Mm58	83.2	84.2	1	39.8	-2.4	0.64
1Mm59	84.2	85.4	1.2	42.8	3.0	1.83
1Mm60	85.4	87.3	1.9	0.0	-42.8	0
1Mm61	87.3	88.1	0.8	44.6	44.6	1.12
1Mm62	88.1	88.6	0.5	46.8	2.2	0.95
1Mm63	88.6	88.8	0.2	40.1	-6.7	0
1Mm64	88.8	89.8	1	45.8	5.8	2.99
1Mm65	89.8	91.3	1.5	45.3	-0.5	1.61
1Mm66	91.3	93	1.7	47.5	2.2	1.08
1Mm67	93	93.2	0.2	45.6	-1.9	0
1Mm68	93.2	93.9	0.7	48.5	2.9	1.32
1Mm69	93.9	94.1	0.2	45.6	-2.9	0
1Mm70	94.1	94.3	0.2	48.0	2.5	0
1Mm71	94.3	94.5	0.2	44.7	-3.3	0
1Mm72	94.5	95.3	0.8	49.5	4.8	1.44
1Mm73	95.3	96	0.7	44.9	-4.7	1.7
1Mm74	96	96.6	0.6	38.5	-6.4	0.95
1Mm75	96.6	97.1	0.5	36.6	-1.9	0.31
1Mm76	97.1	97.8	0.7	37.2	0.6	0.47
1Mm77	97.8	98.3	0.5	36.5	-0.8	0.68
1Mm78	98.3	101.3	3	38.5	2.0	1.06
1Mm79	101.3	101.5	0.2	36.8	-1.6	0
1Mm80	101.5	105.7	4.2	38.1	1.3	0.77
1Mm81	105.7	106	0.3	36.5	-1.6	0.41
1Mm82	106	107	1	38.6	2.1	1.22
1Mm83	107	107.4	0.4	42.0	3.4	0.81
1Mm84	107.4	107.7	0.3	38.9	-3.1	1.28
1Mm85	107.7	108.8	1.1	42.5	3.6	1.22
1Mm86	108.8	110.3	1.5	38.6	-3.9	0.88
1Mm87	110.3	114.1	3.8	36.4	-2.1	0.73
1Mm88	114.1	120.2	6.1	38.5	2.1	0.97
1Mm89	120.2	120.5	0.3	44.4	5.9	0.1
1Mm90	120.5	121.4	0.9	48.2	3.8	1
1Mm91	121.4	121.9	0.5	41.8	-6.5	3.91
1Mm92	121.9	122.5	0.6	47.2	5.5	0.79
1Mm93	122.5	123.7	1.2	43.7	-3.5	1.38
1Mm94	123.7	127.1	3.4	38.9	-4.8	1.11
1Mm95	127.1	130.9	3.8	43.1	4.2	1.75
1Mm96	130.9	132.6	1.7	39.8	-3.4	1.06
1Mm97	132.6	132.9	0.3	44.3	4.5	0.98
1Mm98	132.9	133.2	0.3	47.9	3.7	1.26
1Mm99	133.2	133.4	0.2	40.3	-7.7	0
1Mm100	133.4	133.9	0.5	45.5	5.2	1.35
1Mm101	133.9	135.4	1.5	48.2	2.7	1.91
1Mm102	135.4	135.6	0.2	43.4	-4.8	0
1Mm103	135.6	136.3	0.7	48.2	4.8	0.78
1Mm104	136.3	136.5	0.2	44.4	-3.8	0
1Mm105	136.5	136.7	0.2	47.6	3.2	0
1Mm106	136.7	136.9	0.2	43.3	-4.4	0

1Mm107	136.9	138.2	1.3	48.5	5.2	1.71
1Mm108	138.2	139.2	1	43.6	-4.9	1.2
1Mm109	139.2	140.7	1.5	39.5	-4.1	0.94
1Mm110	140.7	140.9	0.2	41.5	2.0	0
1Mm111	140.9	142.2	1.3	38.1	-3.4	1.02
1Mm112	142.2	142.6	0.4	35.8	-2.3	0.3
1Mm113	142.6	143.2	0.6	37.6	1.8	0.52
1Mm114	143.2	144.9	1.7	36.3	-1.3	0.6
1Mm115	144.9	146.3	1.4	39.2	2.9	0.95
1Mm116	146.3	147.9	1.6	36.5	-2.7	0.73
1Mm117	147.9	148.1	0.2	37.9	1.4	0
1Mm118	148.1	151.3	3.2	36.4	-1.6	0.92
1Mm119	151.3	152.8	1.5	39.2	2.9	0.82
1Mm120	152.8	156.8	4	43.5	4.3	1.54
1Mm121	156.8	157.1	0.3	47.2	3.7	1.02
1Mm122	157.1	157.5	0.4	41.3	-5.9	1.38
1Mm123	157.5	157.7	0.2	46.3	5.0	0
1Mm124	157.7	157.9	0.2	41.0	-5.3	0
1Mm125	157.9	159.1	1.2	44.8	3.8	2.75
1Mm126	159.1	159.3	0.2	37.1	-7.6	0
1Mm127	159.3	159.7	0.4	42.7	5.6	1.3
1Mm128	159.7	160.1	0.4	40.0	-2.8	0.76
1Mm129	160.1	161	0.9	41.4	1.5	0.89
1Mm130	161	161.3	0.3	38.8	-2.7	1.31
1Mm131	161.3	162.1	0.8	43.0	4.3	0.54
1Mm132	162.1	162.9	0.8	39.3	-3.8	1.44
1Mm133	162.9	163.4	0.5	41.9	2.6	0.77
1Mm134	163.4	163.6	0.2	40.0	-1.9	0
1Mm135	163.6	164.8	1.2	41.4	1.4	1.34
1Mm136	164.8	166	1.2	40.0	-1.4	0.67
1Mm137	166	166.5	0.5	42.8	2.8	0.98
1Mm138	166.5	166.7	0.2	40.5	-2.3	0
1Mm139	166.7	167.5	0.8	42.9	2.4	1.58
1Mm140	167.5	167.8	0.3	46.6	3.7	0.37
1Mm141	167.8	168.4	0.6	42.8	-3.8	2.71
1Mm142	168.4	168.6	0.2	40.7	-2.1	0
1Mm143	168.6	170.4	1.8	44.1	3.4	1.8
1Mm144	170.4	171.2	0.8	39.9	-4.2	0.96
1Mm145	171.2	173	1.8	43.1	3.2	1.65
1Mm146	173	173.4	0.4	46.9	3.9	0.69
1Mm147	173.4	173.9	0.5	43.0	-3.9	1.37
1Mm148	173.9	174.6	0.7	46.5	3.5	2.73
1Mm149	174.6	175.2	0.6	39.2	-7.3	0.8
1Mm150	175.2	175.4	0.2	41.5	2.3	0
1Mm151	175.4	177.4	2	39.4	-2.2	1.12
1Mm152	177.4	178.6	1.2	41.7	2.3	0.83
1Mm153	178.6	179.2	0.6	39.6	-2.2	2.36
1Mm154	179.2	181.9	2.7	43.7	4.1	1.55
1Mm155	181.9	182.1	0.2	39.7	-4.0	0
1Mm156	182.1	183	0.9	47.4	7.7	1.58
1Mm157	183	183.3	0.3	44.0	-3.4	2.17
1Mm158	183.3	183.5	0.2	47.4	3.4	0
1Mm159	183.5	184.4	0.9	43.7	-3.7	1.65
1Mm160	184.4	185	0.6	47.4	3.7	1.04
1Mm161	185	185.3	0.3	0.0	-47.4	0
1Mm162	185.3	190.1	4.8	43.3	43.3	1.45

1Mm163	190.1	190.3	0.2	40.6	-2.7	0
1Mm164	190.3	191.5	1.2	43.3	2.7	1.21
1Mm165	191.5	191.9	0.4	47.2	3.9	1.11
1Mm166	191.9	192.2	0.3	45.6	-1.6	0.39
1Mm167	192.2	193.3	1.1	46.7	1.1	0.86
1Mm168	193.3	193.6	0.3	43.5	-3.3	1.42
1Mm169	193.6	193.9	0.3	48.0	4.6	0.61
1Mm170	193.9	194.7	0.8	45.8	-2.3	0.84
1Mm171	194.7	194.9	0.2	40.0	-5.7	0
1Mm172	194.9	195.3	0.4	46.5	6.4	1.33
1Mm173	195.3	195.7	0.4	43.0	-3.5	1.48
1Mm174	195.7	196.4	0.7	40.5	-2.5	0.26
1Mm175	196.4	196.9	0.5	42.2	1.7	1.46
1Mm176	196.9	197.2	0.3	38.7	-3.5	1.84
2Mm1	0	3	3	34.0		0
2Mm2	3	6	3	43.7		1.23
2Mm3	6	6.6	0.6	44.3	0.5	1.87
2Mm4	6.6	9.4	2.8	38.6	-5.6	1.16
2Mm5	9.4	10.8	1.4	42.8	4.2	1.27
2Mm6	10.8	11	0.2	40.8	-2.1	0
2Mm7	11	11.7	0.7	44.1	3.3	1.28
2Mm8	11.7	17.5	5.8	39.5	-4.6	1.27
2Mm9	17.5	18	0.5	43.1	3.6	0.9
2Mm10	18	18.4	0.4	39.4	-3.6	1.65
2Mm11	18.4	19.7	1.3	42.2	2.8	1.3
2Mm12	19.7	20.3	0.6	40.1	-2.2	0.65
2Mm13	20.3	21.3	1	42.4	2.3	0.51
2Mm14	21.3	24.2	2.9	39.5	-2.9	1.07
2Mm15	24.2	24.8	0.6	45.1	5.6	0.9
2Mm16	24.8	26.5	1.7	50.1	5.0	1.87
2Mm17	26.5	26.7	0.2	42.4	-7.6	0
2Mm18	26.7	34	7.3	49.5	7.0	1.85
2Mm19	34	35.1	1.1	43.4	-6.1	2.81
2Mm20	35.1	36.1	1	47.8	4.4	2.2
2Mm21	36.1	37.6	1.5	39.3	-8.5	0.79
2Mm22	37.6	37.9	0.3	45.4	6.1	4.62
2Mm23	37.9	38.1	0.2	39.2	-6.3	0
2Mm24	38.1	38.6	0.5	46.7	7.5	2.65
2Mm25	38.6	40.4	1.8	39.1	-7.6	2.32
2Mm26	40.4	42.6	2.2	36.4	-2.7	0.75
2Mm27	42.6	46.6	4	38.2	1.8	1.19
2Mm28	46.6	47.3	0.7	36.4	-1.8	0.95
2Mm29	47.3	49.2	1.9	38.2	1.8	1.27
2Mm30	49.2	50.1	0.9	42.9	4.7	2.13
2Mm31	50.1	51.4	1.3	40.3	-2.6	0.6
2Mm32	51.4	53	1.6	42.3	2.0	1.15
2Mm33	53	54.5	1.5	38.3	-4.0	1.11
2Mm34	54.5	55.6	1.1	36.6	-1.7	0.62
2Mm35	55.6	56.9	1.3	37.6	1.1	1.19
2Mm36	56.9	57.2	0.3	42.3	4.6	0.85
2Mm37	57.2	58.3	1.1	39.3	-3.0	0.95
2Mm38	58.3	60.8	2.5	42.8	3.5	1.55
2Mm39	60.8	61.9	1.1	39.6	-3.3	1.07
2Mm40	61.9	62.1	0.2	36.3	-3.2	0
2Mm41	62.1	62.6	0.5	39.3	2.9	1.64
2Mm42	62.6	63.6	1	36.8	-2.5	0.5

2Mm43	63.6	67.9	4.3	38.8	1.9	1.25
2Mm44	67.9	68.4	0.5	42.5	3.8	1.42
2Mm45	68.4	68.6	0.2	38.6	-3.9	0
2Mm46	68.6	69.7	1.1	42.1	3.5	1.07
2Mm47	69.7	69.9	0.2	39.9	-2.3	0
2Mm48	69.9	73.5	3.6	43.4	3.6	1.38
2Mm49	73.5	73.8	0.3	40.0	-3.4	0.86
2Mm50	73.8	77.8	4	42.1	2.0	1.63
2Mm51	77.8	81.7	3.9	39.1	-3.0	1.27
2Mm52	81.7	82.2	0.5	36.0	-3.1	0.9
2Mm53	82.2	84.3	2.1	38.7	2.7	1.68
2Mm54	84.3	84.6	0.3	43.2	4.5	0.83
2Mm55	84.6	84.9	0.3	47.1	3.9	0.83
2Mm56	84.9	85.1	0.2	44.4	-2.8	0
2Mm57	85.1	85.6	0.5	37.8	-6.5	1.1
2Mm58	85.6	87	1.4	36.3	-1.5	0.64
2Mm59	87	87.5	0.5	37.8	1.5	1.25
2Mm60	87.5	89.1	1.6	36.4	-1.4	0.82
2Mm61	89.1	90.2	1.1	38.4	2.0	1.56
2Mm62	90.2	91.7	1.5	44.0	5.6	2.27
2Mm63	91.7	91.9	0.2	48.8	4.7	0
2Mm64	91.9	92.2	0.3	42.7	-6.1	1.74
2Mm65	92.2	93.9	1.7	48.9	6.2	1.92
2Mm66	93.9	94.7	0.8	42.1	-6.8	1.66
2Mm67	94.7	96.2	1.5	38.2	-3.9	0.94
2Mm68	96.2	100.2	4	36.6	-1.6	0.66
2Mm69	100.2	100.4	0.2	37.3	0.7	0
2Mm70	100.4	100.6	0.2	36.2	-1.1	0
2Mm71	100.6	101.5	0.9	39.2	3.0	1.5
2Mm72	101.5	104.7	3.2	43.7	4.5	2.02
2Mm73	104.7	104.9	0.2	39.9	-3.8	0
2Mm74	104.9	105.6	0.7	44.2	4.3	0.76
2Mm75	105.6	106.5	0.9	39.2	-5.0	1.23
2Mm76	106.5	106.8	0.3	42.9	3.6	0.73
2Mm77	106.8	110.7	3.9	38.5	-4.4	1.36
2Mm78	110.7	111.4	0.7	36.3	-2.2	1
2Mm79	111.4	112.3	0.9	39.3	3.0	1.85
2Mm80	112.3	112.7	0.4	42.1	2.8	0.29
2Mm81	112.7	113	0.3	40.2	-1.9	0.23
2Mm82	113	114.1	1.1	41.5	1.3	0.58
2Mm83	114.1	115.5	1.4	38.2	-3.4	0.79
2Mm84	115.5	115.9	0.4	41.5	3.4	0.46
2Mm85	115.9	116.7	0.8	39.8	-1.7	1.41
2Mm86	116.7	117.4	0.7	42.4	2.6	1.35
2Mm87	117.4	117.8	0.4	40.4	-2.0	0.34
2Mm88	117.8	118.3	0.5	43.7	3.3	0.7
2Mm89	118.3	118.8	0.5	48.0	4.3	2.17
2Mm90	118.8	119	0.2	43.5	-4.5	0
2Mm91	119	119.2	0.2	48.8	5.3	0
2Mm92	119.2	119.8	0.6	42.9	-5.8	2.91
2Mm93	119.8	120.2	0.4	47.3	4.4	2.24
2Mm94	120.2	120.6	0.4	43.1	-4.2	1.58
2Mm95	120.6	120.8	0.2	39.9	-3.2	0
2Mm96	120.8	121.7	0.9	44.1	4.2	1.6
2Mm97	121.7	121.9	0.2	40.3	-3.8	0
2Mm98	121.9	122.7	0.8	43.7	3.4	2.81

2Mm99	122.7	124.4	1.7	39.1	-4.6	1.26
2Mm100	124.4	125.9	1.5	42.8	3.6	1.15
2Mm101	125.9	126.1	0.2	40.2	-2.6	0
2Mm102	126.1	127.1	1	42.5	2.2	1.81
2Mm103	127.1	127.6	0.5	47.8	5.3	1.69
2Mm104	127.6	128.1	0.5	43.9	-3.9	2.1
2Mm105	128.1	128.3	0.2	46.5	2.6	0
2Mm106	128.3	130.4	2.1	44.2	-2.3	1.58
2Mm107	130.4	130.6	0.2	47.3	3.0	0
2Mm108	130.6	130.9	0.3	43.2	-4.1	1.84
2Mm109	130.9	131.1	0.2	47.9	4.7	0
2Mm110	131.1	132.8	1.7	44.2	-3.7	1.57
2Mm111	132.8	135.5	2.7	40.0	-4.2	1.15
2Mm112	135.5	135.9	0.4	42.2	2.2	0.77
2Mm113	135.9	139.5	3.6	39.3	-2.9	1.31
2Mm114	139.5	139.7	0.2	41.6	2.3	0
2Mm115	139.7	142.4	2.7	39.1	-2.5	1.07
2Mm116	142.4	143	0.6	41.5	2.4	0.77
2Mm117	143	143.4	0.4	40.2	-1.3	0.42
2Mm118	143.4	144.7	1.3	44.4	4.2	1.78
2Mm119	144.7	144.9	0.2	40.7	-3.7	0
2Mm120	144.9	146.1	1.2	43.5	2.8	1.34
2Mm121	146.1	146.3	0.2	40.6	-2.9	0
2Mm122	146.3	146.8	0.5	42.3	1.7	0.76
2Mm123	146.8	147	0.2	40.1	-2.2	0
2Mm124	147	148.8	1.8	42.6	2.5	1.48
2Mm125	148.8	149.6	0.8	38.6	-4.0	1.02
2Mm126	149.6	149.9	0.3	41.6	3.0	0.36
2Mm127	149.9	150.4	0.5	40.7	-0.9	0.47
2Mm128	150.4	151.6	1.2	44.0	3.3	1.82
2Mm129	151.6	153.8	2.2	47.8	3.8	2.18
2Mm130	153.8	154.4	0.6	44.6	-3.2	1.68
2Mm131	154.4	154.6	0.2	47.4	2.8	0
2Mm132	154.6	156.1	1.5	44.7	-2.7	2.6
2Mm133	156.1	156.9	0.8	48.7	4.0	1.83
2Mm134	156.9	157.1	0.2	44.7	-4.0	0
2Mm135	157.1	158.8	1.7	47.9	3.2	2.39
2Mm136	158.8	159.5	0.7	42.2	-5.7	1.5
2Mm137	159.5	159.8	0.3	40.3	-2.0	0.2
2Mm138	159.8	160.1	0.3	43.8	3.5	1.62
2Mm139	160.1	160.4	0.3	46.3	2.6	0.21
2Mm140	160.4	162.7	2.3	43.0	-3.4	1.87
2Mm141	162.7	164	1.3	47.6	4.6	1.23
2Mm142	164	164.2	0.2	43.1	-4.5	0
2Mm143	164.2	169	4.8	49.6	6.5	2.12
2Mm144	169	169.4	0.4	45.3	-4.4	0.41
2Mm145	169.4	170.4	1	46.8	1.5	0.39
2Mm146	170.4	172.1	1.7	44.0	-2.8	1.5
2Mm147	172.1	174.8	2.7	47.9	3.9	2.28
2Mm148	174.8	177.8	3	40.2	-7.7	0.3
2Mm149	177.8	178.2	0.4	44.9	4.7	2.48
2Mm150	178.2	181	2.8	48.6	3.7	1.7
2Mm151	181	181.3	0.3	45.4	-3.2	1.2
2Mm152	181.3	181.5	0.2	46.4	1.0	0
2Mm153	181.5	181.8	0.3	42.3	-4.0	1.58
3Mm1	0	3	3	46.0		0

3Mm2	3	8.5	5.5	38.2		1.29
3Mm3	8.5	10.4	1.9	44.1	5.8	1.51
3Mm4	10.4	13.4	3	37.7	-6.3	1.1
3Mm5	13.4	14.2	0.8	36.8	-0.9	0.39
3Mm6	14.2	21.7	7.5	39.2	2.4	1.32
3Mm7	21.7	22.2	0.5	41.9	2.7	0.66
3Mm8	22.2	23.9	1.7	38.3	-3.6	1.18
3Mm9	23.9	24.1	0.2	35.8	-2.5	0
3Mm10	24.1	24.6	0.5	37.0	1.2	0.32
3Mm11	24.6	25	0.4	36.9	-0.1	0.06
3Mm12	25	25.7	0.7	38.1	1.3	0.45
3Mm13	25.7	26.2	0.5	36.9	-1.2	0.73
3Mm14	26.2	26.7	0.5	39.9	3.0	0.67
3Mm15	26.7	29	2.3	43.1	3.2	1.62
3Mm16	29	30.2	1.2	39.5	-3.5	0.77
3Mm17	30.2	31.4	1.2	43.0	3.5	1.04
3Mm18	31.4	31.6	0.2	39.3	-3.7	0
3Mm19	31.6	33.3	1.7	42.6	3.4	1.3
3Mm20	33.3	34	0.7	40.6	-2.0	1.3
3Mm21	34	35.5	1.5	43.2	2.6	1.28
3Mm22	35.5	35.8	0.3	39.6	-3.6	0.99
3Mm23	35.8	36.7	0.9	42.3	2.6	0.82
3Mm24	36.7	37	0.3	38.5	-3.8	1.57
3Mm25	37	37.3	0.3	43.4	4.9	0.97
3Mm26	37.3	37.5	0.2	39.0	-4.4	0
3Mm27	37.5	39	1.5	42.5	3.6	1.49
3Mm28	39	40.3	1.3	39.7	-2.8	0.71
3Mm29	40.3	40.5	0.2	43.1	3.4	0
3Mm30	40.5	40.8	0.3	40.1	-3.0	0.85
3Mm31	40.8	41.4	0.6	44.0	3.9	1.1
3Mm32	41.4	42.3	0.9	39.1	-5.0	1.17
3Mm33	42.3	43.7	1.4	36.7	-2.3	0.58
3Mm34	43.7	44	0.3	37.5	0.7	0.32
3Mm35	44	44.9	0.9	36.1	-1.4	0.66
3Mm36	44.9	45.4	0.5	37.2	1.0	0.99
3Mm37	45.4	45.9	0.5	36.4	-0.7	0.5
3Mm38	45.9	46.1	0.2	37.5	1.1	0
3Mm39	46.1	48.7	2.6	36.6	-0.9	0.45
3Mm40	48.7	50.6	1.9	38.6	2.0	1.08
3Mm41	50.6	51.3	0.7	43.9	5.3	1.63
3Mm42	51.3	51.6	0.3	46.5	2.6	0.12
3Mm43	51.6	53.7	2.1	43.8	-2.7	1.26
3Mm44	53.7	54.2	0.5	40.3	-3.5	0.36
3Mm45	54.2	55.6	1.4	42.9	2.6	1.04
3Mm46	55.6	57.2	1.6	39.4	-3.5	1.16
3Mm47	57.2	57.5	0.3	42.1	2.7	0.7
3Mm48	57.5	57.7	0.2	39.3	-2.8	0
3Mm49	57.7	58.2	0.5	41.5	2.2	0.77
3Mm50	58.2	58.4	0.2	39.1	-2.4	0
3Mm51	58.4	59	0.6	42.6	3.5	1.02
3Mm52	59	65.6	6.6	39.2	-3.4	1.24
3Mm53	65.6	66.5	0.9	41.7	2.5	1.18
3Mm54	66.5	67.2	0.7	38.9	-2.8	1.38
3Mm55	67.2	67.6	0.4	43.0	4.1	1.37
3Mm56	67.6	68.2	0.6	40.5	-2.5	0.72
3Mm57	68.2	68.7	0.5	43.9	3.4	0.68

3Mm58	68.7	68.9	0.2	40.1	-3.8	0
3Mm59	68.9	69.8	0.9	42.8	2.8	1.09
3Mm60	69.8	70.7	0.9	38.4	-4.4	1.37
3Mm61	70.7	74.9	4.2	36.5	-2.0	0.69
3Mm62	74.9	75.9	1	38.6	2.1	0.86
3Mm63	75.9	77.4	1.5	36.5	-2.1	0.85
3Mm64	77.4	77.7	0.3	37.3	0.8	0.13
3Mm65	77.7	77.9	0.2	36.8	-0.5	0
3Mm66	77.9	78.7	0.8	38.8	2.0	1.23
3Mm67	78.7	79.7	1	42.6	3.8	1.28
3Mm68	79.7	82.7	3	38.8	-3.8	1.18
3Mm69	82.7	84.1	1.4	43.1	4.3	1.7
3Mm70	84.1	84.3	0.2	47.1	4.0	0
3Mm71	84.3	84.6	0.3	43.6	-3.4	2.74
3Mm72	84.6	84.8	0.2	40.4	-3.3	0
3Mm73	84.8	86	1.2	43.7	3.3	1.26
3Mm74	86	86.6	0.6	38.1	-5.6	1.61
3Mm75	86.6	87.5	0.9	43.9	5.8	2.05
3Mm76	87.5	88.5	1	49.1	5.3	1.18
3Mm77	88.5	88.9	0.4	43.7	-5.4	0.56
3Mm78	88.9	89.8	0.9	49.0	5.3	1.89
3Mm79	89.8	90.6	0.8	44.4	-4.6	2
3Mm80	90.6	93	2.4	39.2	-5.3	1.24
3Mm81	93	94.9	1.9	43.3	4.1	2.04
3Mm82	94.9	95.1	0.2	47.2	3.9	0
3Mm83	95.1	99.3	4.2	43.9	-3.3	2.01
3Mm84	99.3	99.9	0.6	40.2	-3.7	1.08
3Mm85	99.9	101.1	1.2	43.8	3.7	2.34
3Mm86	101.1	101.4	0.3	47.8	4.0	1.01
3Mm87	101.4	102.6	1.2	44.8	-3.1	1.03
3Mm88	102.6	102.9	0.3	39.7	-5.1	1.74
3Mm89	102.9	103.3	0.4	43.2	3.5	1.85
3Mm90	103.3	103.5	0.2	46.7	3.5	0
3Mm91	103.5	103.7	0.2	43.1	-3.6	0
3Mm92	103.7	104	0.3	38.7	-4.4	1.35
3Mm93	104	105.2	1.2	44.0	5.4	1.66
3Mm94	105.2	105.4	0.2	46.7	2.6	0
3Mm95	105.4	105.8	0.4	43.3	-3.4	1.85
3Mm96	105.8	106.9	1.1	39.3	-4.0	1.31
3Mm97	106.9	107.2	0.3	44.9	5.5	1.83
3Mm98	107.2	107.9	0.7	47.0	2.1	1.4
3Mm99	107.9	108.1	0.2	45.4	-1.6	0
3Mm100	108.1	108.3	0.2	48.0	2.6	0
3Mm101	108.3	109.2	0.9	42.9	-5.2	1.17
3Mm102	109.2	110.5	1.3	39.1	-3.8	1.17
3Mm103	110.5	113	2.5	36.6	-2.5	0.68
3Mm104	113	113.7	0.7	37.9	1.3	0.86
3Mm105	113.7	114.8	1.1	36.5	-1.4	0.71
3Mm106	114.8	115.3	0.5	38.6	2.1	1.3
3Mm107	115.3	116.8	1.5	42.2	3.6	0.9
3Mm108	116.8	120.7	3.9	38.9	-3.3	1.23
3Mm109	120.7	122	1.3	43.9	5.0	1.66
3Mm110	122	122.2	0.2	46.6	2.7	0
3Mm111	122.2	123.4	1.2	41.7	-4.9	0.91
3Mm112	123.4	124.3	0.9	38.2	-3.5	0.79
3Mm113	124.3	125.3	1	36.5	-1.7	0.56

3Mm114	125.3	126.9	1.6	39.1	2.6	1.17
3Mm115	126.9	127.7	0.8	42.6	3.5	0.96
3Mm116	127.7	128.9	1.2	39.5	-3.1	1.19
3Mm117	128.9	130.8	1.9	43.2	3.7	1.27
3Mm118	130.8	131	0.2	46.7	3.5	0
3Mm119	131	131.4	0.4	44.0	-2.7	1.97
3Mm120	131.4	132.5	1.1	39.7	-4.4	0.67
3Mm121	132.5	132.7	0.2	42.4	2.7	0
3Mm122	132.7	132.9	0.2	39.9	-2.5	0
3Mm123	132.9	133.3	0.4	42.3	2.3	0.67
3Mm124	133.3	134.9	1.6	38.8	-3.4	0.91
3Mm125	134.9	135.5	0.6	42.7	3.9	1.55
3Mm126	135.5	137.2	1.7	39.5	-3.2	1.05
3Mm127	137.2	138.8	1.6	43.0	3.5	1.47
3Mm128	138.8	143.8	5	38.9	-4.1	1.61
3Mm129	143.8	144.4	0.6	44.1	5.2	1.27
3Mm130	144.4	145.2	0.8	40.9	-3.3	0.71
3Mm131	145.2	147	1.8	43.4	2.6	1.88
3Mm132	147	148.3	1.3	39.1	-4.3	0.82
3Mm133	148.3	149.1	0.8	42.1	2.9	0.71
3Mm134	149.1	149.8	0.7	38.5	-3.5	1.11
3Mm135	149.8	150	0.2	36.4	-2.2	0
3Mm136	150	151.5	1.5	38.0	1.6	1.23
3Mm137	151.5	154.5	3	44.0	5.9	1.36
3Mm138	154.5	159.6	5.1	39.1	-4.9	1.37
4Mm1	0	3	3	28.0		0
4Mm2	3	3.8	0.8	41.9		1.28
4Mm3	3.8	8	4.2	39.6	-2.3	1.07
4Mm4	8	10	2	42.6	3.0	1.67
4Mm5	10	10.6	0.6	40.0	-2.7	0.27
4Mm6	10.6	12.5	1.9	42.4	2.4	1.35
4Mm7	12.5	17	4.5	39.0	-3.3	1.06
4Mm8	17	17.2	0.2	36.3	-2.8	0
4Mm9	17.2	17.4	0.2	37.6	1.4	0
4Mm10	17.4	17.6	0.2	36.3	-1.4	0
4Mm11	17.6	17.8	0.2	37.7	1.5	0
4Mm12	17.8	18.1	0.3	36.0	-1.7	0.08
4Mm13	18.1	18.5	0.4	37.5	1.5	0.5
4Mm14	18.5	18.9	0.4	36.3	-1.2	0.31
4Mm15	18.9	21.5	2.6	38.8	2.5	0.98
4Mm16	21.5	22.1	0.6	41.8	3.0	0.88
4Mm17	22.1	23.6	1.5	38.6	-3.3	1.32
4Mm18	23.6	23.8	0.2	35.9	-2.7	0
4Mm19	23.8	26.6	2.8	38.6	2.7	1.18
4Mm20	26.6	28.3	1.7	36.2	-2.5	0.55
4Mm21	28.3	29.4	1.1	37.5	1.3	0.81
4Mm22	29.4	30.1	0.7	36.4	-1.1	0.48
4Mm23	30.1	31.7	1.6	38.3	1.8	1.3
4Mm24	31.7	33.4	1.7	43.6	5.4	1.75
4Mm25	33.4	33.6	0.2	39.4	-4.2	0
4Mm26	33.6	35.4	1.8	41.9	2.5	1.56
4Mm27	35.4	36	0.6	38.6	-3.3	0.87
4Mm28	36	38.8	2.8	36.6	-2.0	0.57
4Mm29	38.8	39.9	1.1	38.5	1.9	0.98
4Mm30	39.9	41.4	1.5	44.1	5.6	1.63
4Mm31	41.4	43.1	1.7	47.4	3.3	1.67

4Mm32	43.1	43.4	0.3	42.9	-4.5	1.81
4Mm33	43.4	44	0.6	47.5	4.5	2.23
4Mm34	44	44.4	0.4	44.3	-3.2	0.88
4Mm35	44.4	44.7	0.3	48.4	4.1	1.15
4Mm36	44.7	45.4	0.7	43.7	-4.7	3.84
4Mm37	45.4	47.3	1.9	48.1	4.4	1.38
4Mm38	47.3	48.2	0.9	43.4	-4.7	1.17
4Mm39	48.2	48.4	0.2	39.3	-4.1	0
4Mm40	48.4	48.8	0.4	43.1	3.9	0.98
4Mm41	48.8	51.4	2.6	38.7	-4.4	1.88
4Mm42	51.4	51.8	0.4	36.8	-1.9	0.72
4Mm43	51.8	53	1.2	38.8	2.0	0.78
4Mm44	53	54.1	1.1	42.8	4.0	1.2
4Mm45	54.1	54.6	0.5	40.2	-2.7	0.23
4Mm46	54.6	57	2.4	42.9	2.7	1.54
4Mm47	57	57.4	0.4	46.8	3.9	0.31
4Mm48	57.4	60	2.6	42.7	-4.1	1.97
4Mm49	60	61.8	1.8	40.8	-1.9	0.46
4Mm50	61.8	62.1	0.3	43.6	2.7	0.92
4Mm51	62.1	62.5	0.4	47.8	4.2	1.8
4Mm52	62.5	62.7	0.2	44.6	-3.2	0
4Mm53	62.7	63.3	0.6	49.0	4.5	2.17
4Mm54	63.3	63.7	0.4	42.7	-6.3	0.68
4Mm55	63.7	67.1	3.4	39.2	-3.5	1.26
4Mm56	67.1	67.7	0.6	36.7	-2.6	0.43
4Mm57	67.7	69.4	1.7	38.0	1.3	0.74
4Mm58	69.4	70.1	0.7	42.1	4.1	1.09
4Mm59	70.1	72.5	2.4	38.5	-3.6	0.96
4Mm60	72.5	72.8	0.3	36.9	-1.6	0.11
4Mm61	72.8	76.8	4	38.3	1.4	1.04
4Mm62	76.8	77.1	0.3	36.5	-1.8	0.38
4Mm63	77.1	77.3	0.2	37.3	0.8	0
4Mm64	77.3	79.7	2.4	36.6	-0.8	0.79
4Mm65	79.7	82.1	2.4	39.3	2.8	1.21
4Mm66	82.1	83.3	1.2	42.5	3.2	1.11
4Mm67	83.3	83.5	0.2	39.7	-2.8	0
4Mm68	83.5	83.9	0.4	44.4	4.7	1.1
4Mm69	83.9	85.9	2	40.1	-4.3	1.13
4Mm70	85.9	86.8	0.9	42.8	2.7	1.5
4Mm71	86.8	87.6	0.8	40.9	-1.9	0.83
4Mm72	87.6	88.8	1.2	41.3	0.4	1.93
4Mm73	88.8	89.2	0.4	42.0	0.7	0.95
4Mm74	89.2	91.7	2.5	38.4	-3.6	1.23
4Mm75	91.7	92.5	0.8	36.2	-2.2	0.36
4Mm76	92.5	92.7	0.2	37.7	1.4	0
4Mm77	92.7	93	0.3	36.5	-1.2	0.4
4Mm78	93	94.7	1.7	39.0	2.5	1.25
4Mm79	94.7	95.6	0.9	42.2	3.3	1.4
4Mm80	95.6	97.2	1.6	38.8	-3.5	0.61
4Mm81	97.2	98.6	1.4	42.5	3.8	1.65
4Mm82	98.6	98.8	0.2	40.2	-2.3	0
4Mm83	98.8	99.4	0.6	43.3	3.1	0.99
4Mm84	99.4	99.6	0.2	39.0	-4.3	0
4Mm85	99.6	101.7	2.1	43.4	4.4	1.14
4Mm86	101.7	102.1	0.4	40.0	-3.3	0.73
4Mm87	102.1	106.1	4	42.7	2.7	1.38

4Mm88	106.1	106.9	0.8	49.2	6.5	1.41
4Mm89	106.9	107.1	0.2	44.9	-4.3	0
4Mm90	107.1	107.7	0.6	49.4	4.5	1.12
4Mm91	107.7	108.1	0.4	44.7	-4.7	0.95
4Mm92	108.1	108.5	0.4	40.3	-4.4	0.87
4Mm93	108.5	109.3	0.8	42.5	2.1	1.22
4Mm94	109.3	109.6	0.3	37.4	-5.0	2.61
4Mm95	109.6	109.9	0.3	44.0	6.6	0.92
4Mm96	109.9	111.1	1.2	38.7	-5.3	1.37
4Mm97	111.1	111.6	0.5	42.0	3.2	2.64
4Mm98	111.6	113.2	1.6	37.6	-4.3	0.79
4Mm99	113.2	113.7	0.5	36.5	-1.2	0.42
4Mm100	113.7	114	0.3	39.3	2.8	0.41
4Mm101	114	114.2	0.2	45.2	5.9	0
4Mm102	114.2	114.6	0.4	47.0	1.7	0.72
4Mm103	114.6	115.5	0.9	42.1	-4.8	1.51
4Mm104	115.5	115.7	0.2	49.1	6.9	0
4Mm105	115.7	116.7	1	42.7	-6.4	1.95
4Mm106	116.7	118.3	1.6	48.2	5.5	1.98
4Mm107	118.3	118.7	0.4	43.8	-4.5	1.72
4Mm108	118.7	119	0.3	47.5	3.7	0.27
4Mm109	119	119.5	0.5	44.5	-3.0	2.85
4Mm110	119.5	120	0.5	49.5	5.0	1.63
4Mm111	120	120.2	0.2	42.1	-7.4	0
4Mm112	120.2	120.6	0.4	46.8	4.7	1.56
4Mm113	120.6	120.8	0.2	44.1	-2.7	0
4Mm114	120.8	122.5	1.7	40.5	-3.6	0.61
4Mm115	122.5	123	0.5	46.6	6.1	1.67
4Mm116	123	123.8	0.8	44.4	-2.2	1.92
4Mm117	123.8	126	2.2	48.7	4.3	1.48
4Mm118	126	126.8	0.8	43.3	-5.4	2.09
4Mm119	126.8	127.4	0.6	47.2	4.0	1.5
4Mm120	127.4	127.6	0.2	44.6	-2.6	0
4Mm121	127.6	129.4	1.8	48.0	3.4	1.17
4Mm122	129.4	129.6	0.2	45.2	-2.8	0
4Mm123	129.6	131.9	2.3	48.4	3.2	2.74
4Mm124	131.9	132.5	0.6	45.4	-2.9	1.1
4Mm125	132.5	136	3.5	48.9	3.5	1.71
4Mm126	136	136.2	0.2	43.1	-5.8	0
4Mm127	136.2	137.6	1.4	50.3	7.2	1.94
4Mm128	137.6	137.8	0.2	45.5	-4.8	0
4Mm129	137.8	142.2	4.4	50.3	4.8	1.74
4Mm130	142.2	143.3	1.1	44.8	-5.5	2.04
4Mm131	143.3	143.5	0.2	40.6	-4.2	0
4Mm132	143.5	145	1.5	42.9	2.3	2.08
4Mm133	145	147.2	2.2	40.2	-2.7	0.79
4Mm134	147.2	149.6	2.4	49.1	8.9	2.05
4Mm135	149.6	149.9	0.3	43.9	-5.2	1.75
4Mm136	149.9	151.6	1.7	49.1	5.2	2.01
4Mm137	151.6	151.9	0.3	53.5	4.4	0.46
4Mm138	151.9	155.7	3.8	49.8	-3.7	1.95
5Mm1	0	3	3	24.0		0
5Mm2	3	3.7	0.7	42.3		0.94
5Mm3	3.7	7	3.3	39.8	-2.6	1.52
5Mm4	7	7.3	0.3	36.3	-3.5	0.33
5Mm5	7.3	8.7	1.4	39.4	3.2	1.22

5Mm6	8.7	9.1	0.4	41.3	1.9	0.21
5Mm7	9.1	10.3	1.2	38.8	-2.5	1.23
5Mm8	10.3	10.5	0.2	36.4	-2.4	0
5Mm9	10.5	12.9	2.4	37.5	1.1	0.68
5Mm10	12.9	13.6	0.7	36.2	-1.3	0.45
5Mm11	13.6	14.9	1.3	38.0	1.8	1.11
5Mm12	14.9	15.2	0.3	41.5	3.5	0.18
5Mm13	15.2	20.1	4.9	38.3	-3.2	1.07
5Mm14	20.1	21.5	1.4	42.3	4.0	1.16
5Mm15	21.5	22.6	1.1	40.8	-1.5	0.72
5Mm16	22.6	23.7	1.1	42.5	1.7	1.33
5Mm17	23.7	24.6	0.9	49.0	6.5	2.44
5Mm18	24.6	24.8	0.2	45.0	-4.0	0
5Mm19	24.8	25	0.2	38.0	-7.0	0
5Mm20	25	25.4	0.4	45.0	7.0	1.53
5Mm21	25.4	26.4	1	42.5	-2.6	0
5Mm22	26.4	28.4	2	43.5	1.0	1.55
5Mm23	28.4	28.6	0.2	47.3	3.8	0
5Mm24	28.6	29.4	0.8	43.6	-3.7	1.39
5Mm25	29.4	29.7	0.3	40.8	-2.8	0.36
5Mm26	29.7	30.6	0.9	43.0	2.2	1.82
5Mm27	30.6	31.6	1	48.2	5.2	1.13
5Mm28	31.6	32	0.4	43.6	-4.7	1.23
5Mm29	32	32.2	0.2	40.2	-3.3	0
5Mm30	32.2	32.8	0.6	45.2	5.0	2.56
5Mm31	32.8	33	0.2	39.9	-5.4	0
5Mm32	33	33.5	0.5	44.5	4.7	1.95
5Mm33	33.5	34.1	0.6	48.6	4.1	2.24
5Mm34	34.1	34.5	0.4	42.8	-5.8	2.14
5Mm35	34.5	35	0.5	45.8	3.0	2.45
5Mm36	35	35.2	0.2	40.9	-4.9	0
5Mm37	35.2	37.9	2.7	49.6	8.7	2
5Mm38	37.9	38.1	0.2	44.6	-4.9	0
5Mm39	38.1	39	0.9	47.2	2.6	1.65
5Mm40	39	39.4	0.4	43.2	-4.0	1.4
5Mm41	39.4	42.1	2.7	39.0	-4.2	1.33
5Mm42	42.1	42.3	0.2	42.4	3.4	0
5Mm43	42.3	44.2	1.9	40.3	-2.1	1.3
5Mm44	44.2	45.1	0.9	42.7	2.4	1.43
5Mm45	45.1	45.8	0.7	40.7	-2.1	0.57
5Mm46	45.8	46	0.2	43.4	2.8	0
5Mm47	46	47.2	1.2	38.5	-4.9	0.87
5Mm48	47.2	48.2	1	36.9	-1.6	0.6
5Mm49	48.2	52.1	3.9	38.5	1.6	1.24
5Mm50	52.1	53.6	1.5	44.1	5.6	1.91
5Mm51	53.6	53.9	0.3	46.2	2.1	1
5Mm52	53.9	54.6	0.7	42.0	-4.2	1.72
5Mm53	54.6	55.5	0.9	38.2	-3.8	1.41
5Mm54	55.5	58	2.5	36.3	-1.9	0.78
5Mm55	58	58.2	0.2	38.8	2.4	0
5Mm56	58.2	62.6	4.4	36.5	-2.3	0.76
5Mm57	62.6	64.2	1.6	38.4	1.9	1.31
5Mm58	64.2	68.3	4.1	44.0	5.5	1.33
5Mm59	68.3	69.2	0.9	38.6	-5.3	1.33
5Mm60	69.2	69.4	0.2	36.2	-2.4	0
5Mm61	69.4	71.1	1.7	37.5	1.3	0.63

5Mm62	71.1	71.5	0.4	36.6	-0.9	0.43
5Mm63	71.5	72.5	1	38.2	1.6	0.81
5Mm64	72.5	78.3	5.8	43.7	5.5	1.56
5Mm65	78.3	78.9	0.6	38.4	-5.3	1.01
5Mm66	78.9	85.9	7	36.5	-1.9	0.88
5Mm67	85.9	88.9	3	38.0	1.5	0.96
5Mm68	88.9	89.5	0.6	42.1	4.1	1.1
5Mm69	89.5	90.9	1.4	40.2	-1.8	1.38
5Mm70	90.9	91.1	0.2	42.3	2.1	0
5Mm71	91.1	91.7	0.6	40.4	-1.9	0.67
5Mm72	91.7	94.1	2.4	43.2	2.7	1.7
5Mm73	94.1	96.7	2.6	40.8	-2.3	0.89
5Mm74	96.7	97.6	0.9	42.6	1.8	0.72
5Mm75	97.6	99.2	1.6	39.9	-2.7	1.42
5Mm76	99.2	104.9	5.7	43.3	3.4	1.6
5Mm77	104.9	105.3	0.4	40.6	-2.7	0.21
5Mm78	105.3	106.1	0.8	42.9	2.2	1.61
5Mm79	106.1	106.3	0.2	46.6	3.8	0
5Mm80	106.3	108.5	2.2	43.1	-3.5	1.52
5Mm81	108.5	108.7	0.2	38.7	-4.4	0
5Mm82	108.7	109.2	0.5	45.0	6.3	1.64
5Mm83	109.2	110.5	1.3	39.7	-5.4	1.42
5Mm84	110.5	110.7	0.2	43.5	3.8	0
5Mm85	110.7	111.3	0.6	47.3	3.8	2.61
5Mm86	111.3	111.6	0.3	45.0	-2.3	1.5
5Mm87	111.6	116.1	4.5	50.0	5.0	1.5
5Mm88	116.1	116.3	0.2	45.7	-4.3	0
5Mm89	116.3	122.9	6.6	48.4	2.7	1.88
5Mm90	122.9	123.1	0.2	45.1	-3.3	0
5Mm91	123.1	124	0.9	47.4	2.3	1.06
5Mm92	124	124.4	0.4	45.0	-2.4	0.89
5Mm93	124.4	124.7	0.3	51.0	5.9	1.03
5Mm94	124.7	124.9	0.2	45.0	-5.9	0
5Mm95	124.9	125.5	0.6	49.4	4.3	0.85
5Mm96	125.5	125.7	0.2	54.2	4.9	0
5Mm97	125.7	126.2	0.5	49.4	-4.9	1.64
5Mm98	126.2	127.9	1.7	43.5	-5.9	1.36
5Mm99	127.9	128.2	0.3	47.0	3.5	0.32
5Mm100	128.2	128.9	0.7	45.0	-1.9	0.66
5Mm101	128.9	130.1	1.2	48.4	3.4	0.99
5Mm102	130.1	130.7	0.6	45.6	-2.8	0.69
5Mm103	130.7	130.9	0.2	46.5	0.9	0
5Mm104	130.9	131.9	1	44.1	-2.4	0.83
5Mm105	131.9	132.1	0.2	46.3	2.2	0
5Mm106	132.1	134.5	2.4	43.2	-3.1	1.2
5Mm107	134.5	136.8	2.3	48.9	5.8	1.24
5Mm108	136.8	137.1	0.3	45.8	-3.1	0.57
5Mm109	137.1	138.3	1.2	49.2	3.4	1.12
5Mm110	138.3	138.8	0.5	44.4	-4.8	1.35
5Mm111	138.8	139.1	0.3	40.0	-4.4	1.6
5Mm112	139.1	141.6	2.5	50.0	9.9	1.88
5Mm113	141.6	142.4	0.8	43.7	-6.3	1.84
5Mm114	142.4	146	3.6	47.9	4.2	1.82
5Mm115	146	147.3	1.3	42.6	-5.3	1.51
5Mm116	147.3	148.2	0.9	46.9	4.4	0.4
5Mm117	148.2	148.5	0.3	44.0	-3.0	1.55

5Mm118	148.5	148.8	0.3	46.4	2.4	0.21
5Mm119	148.8	149.1	0.3	45.1	-1.3	0.68
5Mm120	149.1	150.5	1.4	46.7	1.5	1.02
5Mm121	150.5	151.4	0.9	44.2	-2.5	0.57
5Mm122	151.4	151.6	0.2	40.0	-4.2	0
5Mm123	151.6	152.1	0.5	43.8	3.8	1.04
5Mm124	152.1	152.6	0.5	40.3	-3.5	0.94
6Mm1	0	3	3	50.0		0
6Mm2	3	3.3	0.3	41.4		1.34
6Mm3	3.3	4.8	1.5	39.3	-2.0	1.08
6Mm4	4.8	6.3	1.5	41.8	2.4	0.79
6Mm5	6.3	7.6	1.3	40.5	-1.3	1.01
6Mm6	7.6	7.8	0.2	42.2	1.7	0
6Mm7	7.8	9.9	2.1	39.1	-3.1	1.7
6Mm8	9.9	10.5	0.6	36.7	-2.4	0.53
6Mm9	10.5	14.9	4.4	38.2	1.5	0.94
6Mm10	14.9	15.4	0.5	36.4	-1.8	0.58
6Mm11	15.4	17.4	2	38.7	2.3	1.31
6Mm12	17.4	18	0.6	41.3	2.6	0.4
6Mm13	18	19.6	1.6	38.4	-2.9	1.03
6Mm14	19.6	21.6	2	36.8	-1.6	0.62
6Mm15	21.6	26.2	4.6	39.3	2.5	1.36
6Mm16	26.2	26.9	0.7	36.5	-2.7	0.45
6Mm17	26.9	27.1	0.2	38.2	1.7	0
6Mm18	27.1	27.4	0.3	36.7	-1.5	0.45
6Mm19	27.4	28.3	0.9	39.2	2.5	1.46
6Mm20	28.3	28.8	0.5	43.0	3.8	1.74
6Mm21	28.8	29.5	0.7	47.3	4.3	1.91
6Mm22	29.5	30.7	1.2	44.1	-3.1	1.94
6Mm23	30.7	30.9	0.2	39.9	-4.3	0
6Mm24	30.9	32.8	1.9	44.4	4.5	1.74
6Mm25	32.8	33	0.2	40.5	-3.9	0
6Mm26	33	33.2	0.2	42.2	1.7	0
6Mm27	33.2	33.9	0.7	39.6	-2.7	1.16
6Mm28	33.9	35.4	1.5	43.8	4.2	2.38
6Mm29	35.4	37	1.6	39.6	-4.2	1.04
6Mm30	37	38	1	42.7	3.2	1.52
6Mm31	38	38.2	0.2	46.4	3.6	0
6Mm32	38.2	40.4	2.2	43.6	-2.8	1.96
6Mm33	40.4	45.9	5.5	38.7	-4.8	2.02
6Mm34	45.9	47	1.1	38.3	-0.4	0.67
6Mm35	47	47.4	0.4	41.8	3.5	0.21
6Mm36	47.4	47.6	0.2	40.1	-1.7	0
6Mm37	47.6	48	0.4	48.1	8.0	4.72
6Mm38	48	48.3	0.3	43.6	-4.5	0.71
6Mm39	48.3	48.6	0.3	48.4	4.8	2.32
6Mm40	48.6	51.8	3.2	43.8	-4.6	1.38
6Mm41	51.8	52	0.2	39.7	-4.1	0
6Mm42	52	52.3	0.3	46.3	6.6	3.7
6Mm43	52.3	52.5	0.2	43.9	-2.4	0
6Mm44	52.5	52.7	0.2	40.2	-3.7	0
6Mm45	52.7	55	2.3	44.1	3.9	1.02
6Mm46	55	55.5	0.5	47.7	3.6	1.44
6Mm47	55.5	56	0.5	40.8	-6.9	1.32
6Mm48	56	59.9	3.9	39.6	-1.2	1.32
6Mm49	59.9	60.1	0.2	36.8	-2.8	0

6Mm50	60.1	61.3	1.2	38.4	1.6	0.8
6Mm51	61.3	62.7	1.4	36.6	-1.9	0.54
6Mm52	62.7	63.2	0.5	37.5	1.0	0.45
6Mm53	63.2	63.9	0.7	36.3	-1.3	0.57
6Mm54	63.9	66.9	3	39.5	3.2	1.23
6Mm55	66.9	67.2	0.3	43.4	4.0	1.11
6Mm56	67.2	70.7	3.5	38.9	-4.6	1.15
6Mm57	70.7	72.1	1.4	44.0	5.1	1.63
6Mm58	72.1	72.8	0.7	47.4	3.5	0.84
6Mm59	72.8	73.5	0.7	43.9	-3.5	1.27
6Mm60	73.5	74.4	0.9	38.1	-5.8	1.16
6Mm61	74.4	76.3	1.9	36.6	-1.5	0.34
6Mm62	76.3	81.7	5.4	38.4	1.8	1.11
6Mm63	81.7	82.6	0.9	43.4	5.0	1.11
6Mm64	82.6	83	0.4	46.0	2.6	0.89
6Mm65	83	83.8	0.8	48.0	2.0	1.89
6Mm66	83.8	84	0.2	41.1	-6.9	0
6Mm67	84	84.6	0.6	47.9	6.8	1.42
6Mm68	84.6	85	0.4	39.7	-8.3	1.09
6Mm69	85	85.5	0.5	48.1	8.4	1.66
6Mm70	85.5	85.7	0.2	40.5	-7.6	0
6Mm71	85.7	86	0.3	44.9	4.4	0.97
6Mm72	86	86.8	0.8	46.4	1.5	1.35
6Mm73	86.8	87.9	1.1	45.3	-1.1	1.35
6Mm74	87.9	88.5	0.6	47.7	2.4	1.67
6Mm75	88.5	88.7	0.2	44.2	-3.5	0
6Mm76	88.7	89.4	0.7	49.3	5.1	1.85
6Mm77	89.4	89.9	0.5	42.9	-6.4	1.56
6Mm78	89.9	90.2	0.3	40.6	-2.3	0.36
6Mm79	90.2	92	1.8	48.5	7.9	1.85
6Mm80	92	95.7	3.7	42.7	-5.8	1.28
6Mm81	95.7	96.9	1.2	40.1	-2.5	0.47
6Mm82	96.9	98.1	1.2	43.0	2.8	1.2
6Mm83	98.1	98.7	0.6	39.9	-3.1	0.68
6Mm84	98.7	99.8	1.1	44.3	4.5	1.24
6Mm85	99.8	100.5	0.7	47.5	3.2	1.38
6Mm86	100.5	101.4	0.9	44.1	-3.4	0.8
6Mm87	101.4	105.7	4.3	38.0	-6.1	0.99
6Mm88	105.7	106	0.3	36.5	-1.5	0.26
6Mm89	106	108.1	2.1	39.3	2.8	1.07
6Mm90	108.1	109	0.9	43.6	4.3	1.56
6Mm91	109	112.1	3.1	38.4	-5.2	1.32
6Mm92	112.1	113.2	1.1	44.6	6.2	2.35
6Mm93	113.2	115	1.8	49.0	4.4	2.57
6Mm94	115	115.7	0.7	43.4	-5.6	2.72
6Mm95	115.7	116	0.3	49.2	5.8	1.43
6Mm96	116	117.9	1.9	43.4	-5.8	1.8
6Mm97	117.9	118.3	0.4	46.4	3.0	1.25
6Mm98	118.3	118.6	0.3	43.5	-2.9	1.75
6Mm99	118.6	119.5	0.9	46.7	3.2	1.2
6Mm100	119.5	120.4	0.9	42.2	-4.5	3.41
6Mm101	120.4	120.6	0.2	47.2	5.0	0
6Mm102	120.6	120.8	0.2	43.6	-3.7	0
6Mm103	120.8	121.5	0.7	47.7	4.2	1.53
6Mm104	121.5	122.2	0.7	40.4	-7.3	0.71
6Mm105	122.2	122.9	0.7	44.3	3.9	0.99

6Mm106	122.9	124.2	1.3	39.0	-5.3	0.81
6Mm107	124.2	124.6	0.4	44.4	5.5	2.24
6Mm108	124.6	125.7	1.1	48.6	4.1	1.05
6Mm109	125.7	126.5	0.8	44.0	-4.6	1.46
6Mm110	126.5	126.8	0.3	46.8	2.8	0.54
6Mm111	126.8	127	0.2	44.5	-2.3	0
6Mm112	127	127.3	0.3	46.7	2.1	0.21
6Mm113	127.3	127.5	0.2	45.1	-1.5	0
6Mm114	127.5	128.4	0.9	48.3	3.2	1.69
6Mm115	128.4	129	0.6	42.4	-5.9	1.06
6Mm116	129	130.1	1.1	39.1	-3.3	1.1
6Mm117	130.1	130.3	0.2	36.8	-2.3	0
6Mm118	130.3	132.7	2.4	38.3	1.5	1.16
6Mm119	132.7	132.9	0.2	36.3	-2.0	0
6Mm120	132.9	133.8	0.9	38.0	1.7	0.98
6Mm121	133.8	135	1.2	44.0	6.0	1.67
6Mm122	135	135.2	0.2	46.8	2.7	0
6Mm123	135.2	138.1	2.9	43.8	-3.0	1.1
6Mm124	138.1	139.9	1.8	39.0	-4.8	1.25
6Mm125	139.9	141.2	1.3	43.1	4.1	1.23
6Mm126	141.2	142.3	1.1	39.6	-3.5	0.73
6Mm127	142.3	148.8	6.5	43.6	4.0	1.46
6Mm128	148.8	149.6	0.8	40.2	-3.4	2.27
7Mm1	0	3	3	0.0		0
7Mm2	2.9	4.4	1.5	43.8		2.05
7Mm3	4.4	5.1	0.7	48.4	4.5	1.46
7Mm4	5.1	6	0.9	40.0	-8.4	1.09
7Mm5	6	7.1	1.1	44.1	4.1	1.28
7Mm6	7.1	11.4	4.3	38.4	-5.7	0.89
7Mm7	11.4	12.2	0.8	42.0	3.7	0.71
7Mm8	12.2	13.3	1.1	39.4	-2.6	1
7Mm9	13.3	14	0.7	43.6	4.2	1.99
7Mm10	14	15.1	1.1	39.2	-4.3	0.84
7Mm11	15.1	16.5	1.4	41.7	2.5	0.71
7Mm12	16.5	17.8	1.3	48.0	6.3	2.69
7Mm13	17.8	18.8	1	40.2	-7.9	0.94
7Mm14	18.8	19.4	0.6	43.4	3.2	0.48
7Mm15	19.4	20.5	1.1	50.7	7.4	1.48
7Mm16	20.5	21	0.5	41.9	-8.8	2.19
7Mm17	21	24.8	3.8	40.8	-1.1	0.97
7Mm18	24.8	25.1	0.3	44.3	3.5	1.17
7Mm19	25.1	26.2	1.1	50.5	6.3	1.84
7Mm20	26.2	26.4	0.2	44.0	-6.6	0
7Mm21	26.4	26.6	0.2	50.1	6.2	0
7Mm22	26.6	27	0.4	42.6	-7.5	1.82
7Mm23	27	27.5	0.5	40.1	-2.5	0.71
7Mm24	27.5	27.9	0.4	42.7	2.6	0.39
7Mm25	27.9	28.5	0.6	49.2	6.5	1.11
7Mm26	28.5	28.8	0.3	42.8	-6.5	0.75
7Mm27	28.8	30.3	1.5	49.6	6.9	1.31
7Mm28	30.3	31	0.7	44.6	-5.0	0.73
7Mm29	31	32	1	49.6	5.0	1.26
7Mm30	32	34.8	2.8	40.8	-8.8	0.57
7Mm31	34.8	35.3	0.5	44.7	3.8	1.18
7Mm32	35.3	36.9	1.6	47.4	2.7	1.89
7Mm33	36.9	37.4	0.5	44.4	-3.0	0.65

7Mm34	37.4	37.6	0.2	46.5	2.1	0
7Mm35	37.6	38.1	0.5	44.7	-1.9	0.43
7Mm36	38.1	38.4	0.3	46.6	1.9	0.49
7Mm37	38.4	39.4	1	44.5	-2.1	2.84
7Mm38	39.4	46.3	6.9	0.0	-44.5	0
7Mm39	46.3	48.6	2.3	42.0	42.0	1.21
7Mm40	48.6	50.5	1.9	39.6	-2.5	1.15
7Mm41	50.5	50.9	0.4	45.1	5.5	2.2
7Mm42	50.9	54	3.1	49.7	4.6	2.13
7Mm43	54	54.6	0.6	44.2	-5.5	2.38
7Mm44	54.6	56	1.4	39.9	-4.3	0.76
7Mm45	56	56.9	0.9	47.1	7.2	1.84
7Mm46	56.9	57.6	0.7	43.4	-3.7	1.74
7Mm47	57.6	62.9	5.3	38.7	-4.7	1.23
7Mm48	62.9	63.6	0.7	41.7	3.0	0.86
7Mm49	63.6	65.9	2.3	40.1	-1.6	0.81
7Mm50	65.9	66.1	0.2	42.4	2.3	0
7Mm51	66.1	66.6	0.5	38.2	-4.2	2.17
7Mm52	66.6	67.1	0.5	42.9	4.7	1.19
7Mm53	67.1	67.3	0.2	36.6	-6.3	0
7Mm54	67.3	68	0.7	38.1	1.5	0.38
7Mm55	68	68.8	0.8	36.9	-1.2	0.82
7Mm56	68.8	70.2	1.4	38.7	1.8	1.33
7Mm57	70.2	70.8	0.6	41.3	2.6	0.65
7Mm58	70.8	71	0.2	44.8	3.5	0
7Mm59	71	71.2	0.2	48.3	3.5	0
7Mm60	71.2	73	1.8	44.5	-3.8	1.13
7Mm61	73	73.6	0.6	46.8	2.3	0.95
7Mm62	73.6	74.8	1.2	44.0	-2.8	2.45
7Mm63	74.8	75	0.2	46.6	2.6	0
7Mm64	75	76.1	1.1	44.0	-2.7	2.01
7Mm65	76.1	77.4	1.3	39.2	-4.8	1.13
7Mm66	77.4	78	0.6	41.9	2.8	1.01
7Mm67	78	80.2	2.2	40.2	-1.7	0.79
7Mm68	80.2	81.9	1.7	44.2	4.0	1.94
7Mm69	81.9	82.2	0.3	40.3	-3.9	0.55
7Mm70	82.2	82.6	0.4	42.4	2.1	0.64
7Mm71	82.6	82.9	0.3	40.8	-1.6	0.58
7Mm72	82.9	83.4	0.5	43.8	3.0	2.64
7Mm73	83.4	85.5	2.1	39.0	-4.8	1.18
7Mm74	85.5	85.9	0.4	45.0	6.0	1.64
7Mm75	85.9	88.3	2.4	47.3	2.3	2.05
7Mm76	88.3	91.8	3.5	43.7	-3.6	1.8
7Mm77	91.8	92.2	0.4	39.2	-4.5	1.59
7Mm78	92.2	92.4	0.2	36.7	-2.5	0
7Mm79	92.4	92.7	0.3	37.9	1.2	0.75
7Mm80	92.7	93.9	1.2	36.4	-1.5	0.7
7Mm81	93.9	94.4	0.5	37.5	1.0	0.59
7Mm82	94.4	95	0.6	36.6	-0.9	0.68
7Mm83	95	96.5	1.5	38.4	1.8	0.94
7Mm84	96.5	97.6	1.1	42.0	3.6	1.2
7Mm85	97.6	100.6	3	39.0	-2.9	1.58
7Mm86	100.6	102.2	1.6	36.8	-2.3	0.52
7Mm87	102.2	103.1	0.9	39.4	2.6	1.22
7Mm88	103.1	103.8	0.7	44.1	4.7	1.34
7Mm89	103.8	104	0.2	46.5	2.5	0

7Mm90	104	104.4	0.4	41.5	-5.0	2.6
7Mm91	104.4	105.5	1.1	43.6	2.0	3.58
7Mm92	105.5	105.7	0.2	49.2	5.6	0
7Mm93	105.7	105.9	0.2	43.5	-5.7	0
7Mm94	105.9	106.1	0.2	49.6	6.1	0
7Mm95	106.1	106.3	0.2	44.7	-4.9	0
7Mm96	106.3	106.9	0.6	49.1	4.4	1.42
7Mm97	106.9	108	1.1	44.0	-5.1	2.15
7Mm98	108	108.2	0.2	50.3	6.3	0
7Mm99	108.2	108.4	0.2	40.7	-9.5	0
7Mm100	108.4	108.7	0.3	49.7	8.9	2.45
7Mm101	108.7	109.8	1.1	44.0	-5.7	2.05
7Mm102	109.8	110.7	0.9	38.7	-5.3	1.24
7Mm103	110.7	110.9	0.2	36.7	-2.0	0
7Mm104	110.9	111.3	0.4	39.3	2.6	1
7Mm105	111.3	111.5	0.2	42.3	3.0	0
7Mm106	111.5	112.5	1	39.5	-2.8	1.06
7Mm107	112.5	113	0.5	44.7	5.2	1.67
7Mm108	113	114.5	1.5	39.4	-5.3	0.71
7Mm109	114.5	114.9	0.4	43.9	4.4	1.26
7Mm110	114.9	116	1.1	39.0	-4.8	0.94
7Mm111	116	117.5	1.5	44.3	5.2	1.98
7Mm112	117.5	117.7	0.2	37.9	-6.4	0
7Mm113	117.7	119.3	1.6	44.4	6.4	1.68
7Mm114	119.3	119.6	0.3	47.6	3.2	0.73
7Mm115	119.6	121.5	1.9	44.1	-3.4	2.06
7Mm116	121.5	121.8	0.3	38.3	-5.8	1.91
7Mm117	121.8	122.7	0.9	43.0	4.6	1.43
7Mm118	122.7	123.2	0.5	39.8	-3.2	0.62
7Mm119	123.2	124.5	1.3	43.8	4.0	1.98
7Mm120	124.5	124.8	0.3	46.3	2.5	0.11
7Mm121	124.8	125.5	0.7	43.7	-2.5	1.87
7Mm122	125.5	126.2	0.7	46.9	3.2	0.96
7Mm123	126.2	127.4	1.2	42.8	-4.1	1.13
7Mm124	127.4	127.8	0.4	40.0	-2.8	0.83
7Mm125	127.8	129	1.2	44.6	4.6	1.51
7Mm126	129	129.4	0.4	46.5	1.8	1.8
7Mm127	129.4	132.4	3	43.4	-3.1	1.71
7Mm128	132.4	135.1	2.7	47.9	4.5	1.54
7Mm129	135.1	135.3	0.2	45.2	-2.8	0
7Mm130	135.3	135.5	0.2	46.8	1.6	0
7Mm131	135.5	137.3	1.8	43.9	-2.8	0.88
7Mm132	137.3	138.2	0.9	45.7	1.7	2.41
7Mm133	138.2	139	0.8	44.4	-1.3	1.41
7Mm134	139	140	1	47.3	3.0	1.61
7Mm135	140	140.2	0.2	42.4	-4.9	0
7Mm136	140.2	140.4	0.2	47.2	4.8	0
7Mm137	140.4	142.7	2.3	44.7	-2.5	1.02
7Mm138	142.7	142.9	0.2	47.0	2.3	0
7Mm139	142.9	144.3	1.4	44.8	-2.2	0.96
7Mm140	144.3	144.7	0.4	46.8	2.1	0.46
7Mm141	144.7	146.1	1.4	45.4	-1.4	0.53
7Mm142	146.1	147.4	1.3	48.9	3.4	1.3
7Mm143	147.4	147.7	0.3	42.4	-6.5	1.34
7Mm144	147.7	147.9	0.2	40.3	-2.1	0
7Mm145	147.9	149.9	2	48.7	8.4	2.26

7Mm146	149.9	150.1	0.2	43.1	-5.6	0
7Mm147	150.1	152.6	2.5	48.9	5.8	1.91
8Mm1	0	3	3	36.0		0
8Mm2	3	3.4	0.4	43.4		2.01
8Mm3	3.4	3.7	0.3	47.9	4.4	0.83
8Mm4	3.7	4.6	0.9	43.5	-4.3	2.4
8Mm5	4.6	5.7	1.1	38.9	-4.7	1.31
8Mm6	5.7	6.4	0.7	36.5	-2.3	0.41
8Mm7	6.4	6.8	0.4	37.3	0.8	0.24
8Mm8	6.8	7.5	0.7	37.0	-0.3	0.61
8Mm9	7.5	8.3	0.8	38.7	1.7	1.5
8Mm10	8.3	10.8	2.5	43.1	4.4	1.26
8Mm11	10.8	13.7	2.9	48.2	5.2	1.25
8Mm12	13.7	14.9	1.2	45.2	-3.1	1.02
8Mm13	14.9	15.1	0.2	49.3	4.2	0
8Mm14	15.1	16.5	1.4	41.9	-7.4	1.19
8Mm15	16.5	18.3	1.8	39.7	-2.2	0.72
8Mm16	18.3	19.2	0.9	43.1	3.4	1.78
8Mm17	19.2	19.5	0.3	40.8	-2.3	0.14
8Mm18	19.5	20.1	0.6	41.8	1.0	0.82
8Mm19	20.1	22	1.9	0.0	-41.8	0
8Mm20	22	23	1	40.2	40.2	0.46
8Mm21	23	24.1	1.1	43.8	3.7	1.35
8Mm22	24.1	24.3	0.2	49.1	5.3	0
8Mm23	24.3	26.5	2.2	43.6	-5.5	1.82
8Mm24	26.5	26.7	0.2	47.2	3.6	0
8Mm25	26.7	27.9	1.2	43.5	-3.7	1.22
8Mm26	27.9	28.3	0.4	47.3	3.8	1.03
8Mm27	28.3	28.6	0.3	44.0	-3.3	1.9
8Mm28	28.6	32	3.4	39.0	-5.0	1.08
8Mm29	32	32.5	0.5	43.5	4.5	1.58
8Mm30	32.5	34.7	2.2	39.9	-3.6	0.88
8Mm31	34.7	37.8	3.1	44.0	4.1	1.28
8Mm32	37.8	38.6	0.8	38.8	-5.2	1.07
8Mm33	38.6	39.8	1.2	36.1	-2.7	0.52
8Mm34	39.8	42	2.2	38.8	2.7	1.37
8Mm35	42	42.3	0.3	42.6	3.7	1.38
8Mm36	42.3	43.2	0.9	39.2	-3.4	1.4
8Mm37	43.2	43.6	0.4	36.3	-2.9	0.35
8Mm38	43.6	44.2	0.6	38.0	1.7	0.69
8Mm39	44.2	44.5	0.3	41.7	3.7	0.18
8Mm40	44.5	45.8	1.3	38.7	-3.0	0.89
8Mm41	45.8	46.6	0.8	42.0	3.3	1.1
8Mm42	46.6	46.9	0.3	40.5	-1.5	0.51
8Mm43	46.9	49.9	3	43.7	3.2	1.41
8Mm44	49.9	51	1.1	38.6	-5.1	1.24
8Mm45	51	54.6	3.6	36.3	-2.2	0.75
8Mm46	54.6	56.2	1.6	37.7	1.3	0.83
8Mm47	56.2	58	1.8	46.9	9.2	0
8Mm48	58	58.2	0.2	38.3	-8.6	0
8Mm49	58.2	58.6	0.4	36.2	-2.1	0.44
8Mm50	58.6	59.8	1.2	38.7	2.5	0.67
8Mm51	59.8	60.3	0.5	42.9	4.1	0.83
8Mm52	60.3	60.7	0.4	39.4	-3.5	1.06
8Mm53	60.7	62.4	1.7	36.7	-2.6	0.66
8Mm54	62.4	63.1	0.7	38.9	2.2	1.1

8Mm55	63.1	63.4	0.3	41.8	2.9	0.77
8Mm56	63.4	63.6	0.2	38.9	-2.9	0
8Mm57	63.6	64.3	0.7	43.0	4.1	1.31
8Mm58	64.3	65.5	1.2	37.9	-5.0	0.96
8Mm59	65.5	65.9	0.4	36.0	-2.0	0.18
8Mm60	65.9	67	1.1	37.9	2.0	1.04
8Mm61	67	67.7	0.7	43.0	5.1	0.85
8Mm62	67.7	70.7	3	39.2	-3.9	1.12
8Mm63	70.7	70.9	0.2	42.0	2.8	0
8Mm64	70.9	71.2	0.3	40.5	-1.5	0.34
8Mm65	71.2	72.3	1.1	41.9	1.5	1.2
8Mm66	72.3	73.2	0.9	50.8	8.9	1.92
8Mm67	73.2	73.4	0.2	53.8	2.9	0
8Mm68	73.4	73.8	0.4	43.8	-10.0	3.81
8Mm69	73.8	74.3	0.5	51.0	7.2	1.64
8Mm70	74.3	74.7	0.4	43.4	-7.6	1.74
8Mm71	74.7	75.3	0.6	48.1	4.7	0.45
8Mm72	75.3	75.9	0.6	41.4	-6.7	0.87
8Mm73	75.9	77.2	1.3	39.4	-2.0	0.63
8Mm74	77.2	77.8	0.6	44.7	5.3	1.66
8Mm75	77.8	79.6	1.8	38.8	-5.9	0.81
8Mm76	79.6	82.2	2.6	43.3	4.5	1.3
8Mm77	82.2	83.1	0.9	40.6	-2.6	0.6
8Mm78	83.1	83.5	0.4	43.3	2.6	1.75
8Mm79	83.5	86.1	2.6	40.4	-2.9	1.91
8Mm80	86.1	87.6	1.5	49.5	9.1	1.52
8Mm81	87.6	88.2	0.6	42.6	-6.9	2.24
8Mm82	88.2	88.7	0.5	39.9	-2.7	0.87
8Mm83	88.7	90.1	1.4	44.6	4.7	1.32
8Mm84	90.1	90.6	0.5	48.9	4.3	1.4
8Mm85	90.6	90.8	0.2	43.8	-5.1	0
8Mm86	90.8	91.6	0.8	47.4	3.6	2.36
8Mm87	91.6	94.1	2.5	43.0	-4.4	1.39
8Mm88	94.1	94.5	0.4	47.4	4.4	0.99
8Mm89	94.5	94.9	0.4	45.0	-2.4	0.54
8Mm90	94.9	95.1	0.2	46.8	1.8	0
8Mm91	95.1	96.8	1.7	44.0	-2.9	2
8Mm92	96.8	98.1	1.3	47.9	4.0	2.47
8Mm93	98.1	98.7	0.6	43.7	-4.2	2
8Mm94	98.7	100.8	2.1	38.1	-5.7	0.97
8Mm95	100.8	101.9	1.1	36.7	-1.4	0.56
8Mm96	101.9	103.2	1.3	37.5	0.7	0.55
8Mm97	103.2	104.3	1.1	36.8	-0.7	0.47
8Mm98	104.3	105.9	1.6	38.5	1.7	1.3
8Mm99	105.9	106.5	0.6	43.1	4.7	0.78
8Mm100	106.5	106.8	0.3	46.9	3.8	0.3
8Mm101	106.8	108	1.2	45.0	-1.8	2.23
8Mm102	108	108.5	0.5	47.5	2.4	1.43
8Mm103	108.5	109.9	1.4	45.2	-2.3	1.96
8Mm104	109.9	110.1	0.2	46.6	1.4	0
8Mm105	110.1	111.2	1.1	43.6	-3.0	0.88
8Mm106	111.2	111.5	0.3	48.4	4.9	0.77
8Mm107	111.5	112	0.5	41.6	-6.8	1.58
8Mm108	112	112.2	0.2	46.9	5.3	0
8Mm109	112.2	113.1	0.9	44.8	-2.2	2.03
8Mm110	113.1	113.6	0.5	47.5	2.7	2.43

8Mm111	113.6	114.1	0.5	44.5	-3.0	1.73
8Mm112	114.1	114.3	0.2	47.9	3.4	0
8Mm113	114.3	114.9	0.6	44.0	-3.9	1.8
8Mm114	114.9	116	1.1	40.2	-3.8	0.97
8Mm115	116	119.4	3.4	44.5	4.3	1.22
8Mm116	119.4	120.9	1.5	47.5	3.0	1.87
8Mm117	120.9	121.5	0.6	45.1	-2.4	0.61
8Mm118	121.5	122.7	1.2	49.7	4.6	2.1
8Mm119	122.7	123.1	0.4	54.2	4.5	1.19
8Mm120	123.1	124.7	1.6	51.3	-2.9	0.65
8Mm121	124.7	125.4	0.7	53.0	1.7	0.97
8Mm122	125.4	125.6	0.2	45.3	-7.7	0
8Mm123	125.6	126	0.4	49.9	4.5	1.18
8Mm124	126	126.2	0.2	55.3	5.4	0
8Mm125	126.2	128.6	2.4	48.2	-7.1	1.08
8Mm126	128.6	128.8	0.2	45.8	-2.4	0
8Mm127	128.8	129.5	0.7	47.2	1.4	0.57
8Mm128	129.5	131.2	1.7	42.4	-4.8	0.73
8Mm129	131.2	131.8	0.6	38.6	-3.8	0.9
9Mm1	0	3	3	38.0		0
9Mm2	3	3.3	0.3	42.3		0.46
9Mm3	3.3	7.6	4.3	38.6	-3.7	1.31
9Mm4	7.6	8.1	0.5	42.8	4.2	0.53
9Mm5	8.1	10.7	2.6	39.2	-3.6	1.15
9Mm6	10.7	10.9	0.2	36.3	-3.0	0
9Mm7	10.9	13.3	2.4	38.3	2.1	1.06
9Mm8	13.3	14.3	1	43.0	4.6	1.24
9Mm9	14.3	14.5	0.2	46.2	3.2	0
9Mm10	14.5	15.8	1.3	43.9	-2.2	1.61
9Mm11	15.8	20.2	4.4	38.6	-5.3	1.31
9Mm12	20.2	20.4	0.2	42.2	3.6	0
9Mm13	20.4	22	1.6	49.3	7.1	1.31
9Mm14	22	23.3	1.3	42.5	-6.8	0.96
9Mm15	23.3	24.5	1.2	40.1	-2.4	0.93
9Mm16	24.5	24.9	0.4	42.3	2.2	0.9
9Mm17	24.9	25.1	0.2	39.7	-2.6	0
9Mm18	25.1	25.4	0.3	43.2	3.5	1.62
9Mm19	25.4	26.2	0.8	39.4	-3.9	0.65
9Mm20	26.2	28.3	2.1	42.7	3.3	1.72
9Mm21	28.3	28.8	0.5	40.1	-2.5	0.61
9Mm22	28.8	32.8	4	43.2	3.0	1.57
9Mm23	32.8	34.2	1.4	39.3	-3.8	0.94
9Mm24	34.2	35.4	1.2	44.8	5.5	1.29
9Mm25	35.4	36.6	1.2	39.4	-5.4	0.82
9Mm26	36.6	37.5	0.9	44.3	4.9	1.95
9Mm27	37.5	40	2.5	37.7	-6.5	1.11
9Mm28	40	40.8	0.8	44.8	7.1	1.3
9Mm29	40.8	41	0.2	46.5	1.7	0
9Mm30	41	42.3	1.3	43.8	-2.7	1.61
9Mm31	42.3	44.7	2.4	47.7	3.9	1.79
9Mm32	44.7	44.9	0.2	44.3	-3.4	0
9Mm33	44.9	45.8	0.9	48.5	4.1	1.12
9Mm34	45.8	46	0.2	45.0	-3.4	0
9Mm35	46	46.5	0.5	47.4	2.4	1.28
9Mm36	46.5	48.4	1.9	44.2	-3.2	1.36
9Mm37	48.4	48.7	0.3	47.4	3.2	0.93

9Mm38	48.7	50.7	2	44.2	-3.2	1.4
9Mm39	50.7	50.9	0.2	40.5	-3.7	0
9Mm40	50.9	51.2	0.3	43.2	2.7	1.53
9Mm41	51.2	51.5	0.3	40.7	-2.5	0.15
9Mm42	51.5	52.1	0.6	41.7	1.0	0.87
9Mm43	52.1	52.7	0.6	40.5	-1.3	0.41
9Mm44	52.7	52.9	0.2	42.0	1.5	0
9Mm45	52.9	53.4	0.5	39.9	-2.1	2.22
9Mm46	53.4	54.4	1	42.6	2.8	1.48
9Mm47	54.4	54.7	0.3	46.5	3.8	0.51
9Mm48	54.7	55.4	0.7	43.6	-2.8	2.84
9Mm49	55.4	55.9	0.5	39.2	-4.5	1.28
9Mm50	55.9	56.1	0.2	45.7	6.6	0
9Mm51	56.1	56.3	0.2	39.8	-6.0	0
9Mm52	56.3	56.8	0.5	49.6	9.8	2.23
9Mm53	56.8	57.1	0.3	44.2	-5.3	1.74
9Mm54	57.1	58.4	1.3	48.1	3.8	2
9Mm55	58.4	59.6	1.2	43.1	-5.0	2.87
9Mm56	59.6	59.8	0.2	37.7	-5.4	0
9Mm57	59.8	60.8	1	45.3	7.6	0.83
9Mm58	60.8	62.7	1.9	47.8	2.5	2
9Mm59	62.7	62.9	0.2	42.8	-5.0	0
9Mm60	62.9	63.1	0.2	46.9	4.2	0
9Mm61	63.1	63.5	0.4	42.7	-4.2	2.24
9Mm62	63.5	64	0.5	49.1	6.3	1.13
9Mm63	64	64.2	0.2	44.1	-5.0	0
9Mm64	64.2	64.5	0.3	48.7	4.5	0.96
9Mm65	64.5	64.9	0.4	43.6	-5.1	1.35
9Mm66	64.9	65.5	0.6	47.0	3.4	1.43
9Mm67	65.5	65.7	0.2	43.4	-3.6	0
9Mm68	65.7	65.9	0.2	40.8	-2.6	0
9Mm69	65.9	70.3	4.4	44.0	3.2	1.94
9Mm70	70.3	70.6	0.3	40.0	-4.1	0.71
9Mm71	70.6	71.4	0.8	44.3	4.3	1.12
9Mm72	71.4	71.7	0.3	46.5	2.2	0.26
9Mm73	71.7	72.5	0.8	39.6	-6.9	2.18
9Mm74	72.5	73.3	0.8	44.0	4.3	1.4
9Mm75	73.3	74.5	1.2	40.7	-3.3	0.55
9Mm76	74.5	75.6	1.1	43.6	2.9	2.16
9Mm77	75.6	77.2	1.6	38.2	-5.4	0.74
9Mm78	77.2	78.5	1.3	44.2	6.0	1.19
9Mm79	78.5	79.7	1.2	39.5	-4.7	1.1
9Mm80	79.7	80.1	0.4	41.9	2.4	1.6
9Mm81	80.1	83.4	3.3	38.7	-3.2	1.38
9Mm82	83.4	83.9	0.5	42.6	3.8	1.17
9Mm83	83.9	86.4	2.5	39.4	-3.1	1.48
9Mm84	86.4	87.4	1	41.8	2.3	0.91
9Mm85	87.4	88	0.6	40.7	-1.0	0.62
9Mm86	88	89.2	1.2	41.5	0.8	0.95
9Mm87	89.2	89.4	0.2	40.3	-1.2	0
9Mm88	89.4	90.2	0.8	44.4	4.2	1.91
9Mm89	90.2	93.3	3.1	38.3	-6.1	1.2
9Mm90	93.3	93.5	0.2	36.8	-1.5	0
9Mm91	93.5	94.5	1	38.9	2.1	1.29
9Mm92	94.5	94.7	0.2	42.4	3.5	0
9Mm93	94.7	94.9	0.2	40.9	-1.5	0

9Mm94	94.9	95.6	0.7	42.9	2.1	0.93
9Mm95	95.6	95.9	0.3	39.2	-3.7	1.81
9Mm96	95.9	96.7	0.8	43.5	4.3	1.95
9Mm97	96.7	96.9	0.2	47.8	4.2	0
9Mm98	96.9	99.2	2.3	44.1	-3.7	1.3
9Mm99	99.2	99.4	0.2	47.5	3.4	0
9Mm100	99.4	100.4	1	42.7	-4.8	1.75
9Mm101	100.4	100.6	0.2	40.4	-2.3	0
9Mm102	100.6	100.8	0.2	36.1	-4.3	0
9Mm103	100.8	101.2	0.4	41.5	5.4	1.91
9Mm104	101.2	102.9	1.7	44.8	3.2	1.94
9Mm105	102.9	103.1	0.2	48.7	3.9	0
9Mm106	103.1	106	2.9	43.7	-5.0	1.41
9Mm107	106	106.6	0.6	49.1	5.4	2.73
9Mm108	106.6	106.9	0.3	42.8	-6.3	0.89
9Mm109	106.9	107.1	0.2	39.4	-3.3	0
9Mm110	107.1	108.1	1	49.2	9.8	3.92
9Mm111	108.1	108.3	0.2	45.5	-3.7	0
9Mm112	108.3	108.5	0.2	47.9	2.3	0
9Mm113	108.5	108.7	0.2	44.1	-3.7	0
9Mm114	108.7	109	0.3	50.8	6.7	1.34
9Mm115	109	110.2	1.2	43.2	-7.6	1.46
9Mm116	110.2	111	0.8	47.6	4.4	3.02
9Mm117	111	112.2	1.2	43.0	-4.6	1.48
9Mm118	112.2	113	0.8	40.4	-2.6	0.85
9Mm119	113	114.3	1.3	43.5	3.1	1.83
9Mm120	114.3	115.1	0.8	47.4	3.9	0.97
9Mm121	115.1	118.5	3.4	44.0	-3.4	1.43
9Mm122	118.5	122.6	4.1	47.9	3.9	1.66
9Mm123	122.6	123.9	1.3	43.2	-4.7	1.65
9Mm124	123.9	124.1	0.2	39.0	-4.2	0
10Mm1	0	3	3	48.0		0
10Mm2	3	3.3	0.3	44.1		1.23
10Mm3	3.3	5	1.7	40.1	-4.1	0.93
10Mm4	5	5.6	0.6	41.5	1.4	0.92
10Mm5	5.6	8.8	3.2	43.4	1.9	1.21
10Mm6	8.8	12.4	3.6	40.1	-3.3	0.69
10Mm7	12.4	14	1.6	42.4	2.3	0.95
10Mm8	14	15.1	1.1	38.6	-3.8	1.24
10Mm9	15.1	15.3	0.2	36.6	-2.0	0
10Mm10	15.3	15.7	0.4	37.2	0.7	0.45
10Mm11	15.7	16.6	0.9	36.3	-0.9	0.52
10Mm12	16.6	17.7	1.1	39.7	3.4	1.57
10Mm13	17.7	20.5	2.8	42.3	2.6	1.06
10Mm14	20.5	20.8	0.3	40.4	-1.9	0.45
10Mm15	20.8	22.7	1.9	43.0	2.6	0.91
10Mm16	22.7	23.5	0.8	40.2	-2.8	0.71
10Mm17	23.5	23.9	0.4	41.7	1.5	1.3
10Mm18	23.9	24.3	0.4	40.7	-1.0	0.19
10Mm19	24.3	26.1	1.8	42.0	1.3	0.83
10Mm20	26.1	32.8	6.7	38.7	-3.2	1.23
10Mm21	32.8	33.2	0.4	35.9	-2.8	0.42
10Mm22	33.2	35.6	2.4	38.5	2.6	1.44
10Mm23	35.6	35.8	0.2	36.1	-2.4	0
10Mm24	35.8	38.8	3	39.0	2.9	1.47
10Mm25	38.8	39.4	0.6	44.7	5.7	1.86

10Mm26	39.4	39.6	0.2	39.0	-5.7	0
10Mm27	39.6	44.2	4.6	44.2	5.2	1.57
10Mm28	44.2	44.7	0.5	40.8	-3.4	0.36
10Mm29	44.7	45.1	0.4	43.3	2.5	1.59
10Mm30	45.1	46.8	1.7	38.5	-4.9	1.11
10Mm31	46.8	47	0.2	35.8	-2.7	0
10Mm32	47	47.7	0.7	37.3	1.5	1.07
10Mm33	47.7	49.5	1.8	36.1	-1.2	0.95
10Mm34	49.5	50.3	0.8	38.6	2.5	0.71
10Mm35	50.3	50.5	0.2	36.1	-2.4	0
10Mm36	50.5	53.1	2.6	39.7	3.5	1.37
10Mm37	53.1	53.5	0.4	42.2	2.5	1.13
10Mm38	53.5	54.8	1.3	38.6	-3.6	1.26
10Mm39	54.8	55.2	0.4	36.7	-1.9	0.2
10Mm40	55.2	57.6	2.4	38.9	2.2	0.93
10Mm41	57.6	59.3	1.7	43.5	4.6	1.82
10Mm42	59.3	61.9	2.6	49.3	5.8	1.49
10Mm43	61.9	63.2	1.3	43.5	-5.7	1.92
10Mm44	63.2	64.5	1.3	38.8	-4.8	1.32
10Mm45	64.5	65	0.5	36.7	-2.1	0.22
10Mm46	65	65.9	0.9	38.3	1.6	1.12
10Mm47	65.9	68	2.1	43.5	5.2	1.53
10Mm48	68	68.3	0.3	40.3	-3.2	0.42
10Mm49	68.3	69.5	1.2	43.9	3.7	1.13
10Mm50	69.5	69.7	0.2	46.4	2.4	0
10Mm51	69.7	71.2	1.5	43.7	-2.6	1.47
10Mm52	71.2	74.3	3.1	38.9	-4.8	0.75
10Mm53	74.3	74.7	0.4	47.1	8.2	3.74
10Mm54	74.7	75	0.3	45.2	-1.9	0.68
10Mm55	75	75.4	0.4	48.7	3.5	1.71
10Mm56	75.4	75.7	0.3	44.2	-4.5	1.47
10Mm57	75.7	78	2.3	48.4	4.2	1.77
10Mm58	78	78.3	0.3	43.0	-5.4	0.82
10Mm59	78.3	78.9	0.6	39.2	-3.7	0.96
10Mm60	78.9	80.4	1.5	52.8	13.5	3.32
10Mm61	80.4	80.6	0.2	52.3	-0.4	0
10Mm62	80.6	81.1	0.5	54.2	1.8	1.82
10Mm63	81.1	81.5	0.4	42.3	-11.9	0.93
10Mm64	81.5	82.1	0.6	40.2	-2.1	1.27
10Mm65	82.1	82.3	0.2	44.2	4.0	0
10Mm66	82.3	82.7	0.4	48.1	3.9	0.83
10Mm67	82.7	83.8	1.1	44.6	-3.5	1.05
10Mm68	83.8	84	0.2	47.2	2.6	0
10Mm69	84	84.9	0.9	44.3	-2.9	1.6
10Mm70	84.9	85.1	0.2	46.7	2.4	0
10Mm71	85.1	87.4	2.3	43.5	-3.2	1.78
10Mm72	87.4	87.7	0.3	40.5	-3.0	0.45
10Mm73	87.7	88.3	0.6	43.2	2.8	1.36
10Mm74	88.3	88.7	0.4	40.4	-2.9	0.58
10Mm75	88.7	89.3	0.6	42.2	1.9	0.5
10Mm76	89.3	90	0.7	39.7	-2.5	0.71
10Mm77	90	91.1	1.1	42.5	2.8	1.06
10Mm78	91.1	92.2	1.1	40.5	-2.0	0.84
10Mm79	92.2	96.2	4	43.5	3.0	1.74
10Mm80	96.2	98.5	2.3	38.9	-4.6	1.3
10Mm81	98.5	99.1	0.6	42.7	3.8	1.05

10Mm82	99.1	100.3	1.2	39.2	-3.5	1.01
10Mm83	100.3	102.7	2.4	36.5	-2.7	0.74
10Mm84	102.7	103.2	0.5	37.9	1.4	0.53
10Mm85	103.2	103.9	0.7	36.5	-1.3	0.92
10Mm86	103.9	106.1	2.2	38.5	2.0	1.19
10Mm87	106.1	107.7	1.6	38.3	-0.3	1.69
10Mm88	107.7	108	0.3	42.9	4.6	0.75
10Mm89	108	110.1	2.1	38.5	-4.4	1.08
10Mm90	110.1	111.5	1.4	42.9	4.4	0.9
10Mm91	111.5	113	1.5	38.0	-4.9	1.02
10Mm92	113	114	1	36.8	-1.2	0.47
10Mm93	114	114.4	0.4	38.6	1.9	0.8
10Mm94	114.4	115.3	0.9	41.8	3.2	0.92
10Mm95	115.3	120.8	5.5	43.6	1.8	1.48
10Mm96	120.8	122.8	2	43.7	0.1	2.28
10Mm97	122.8	125.2	2.4	38.8	-4.9	1.31
10Mm98	125.2	126.3	1.1	42.6	3.9	0.91
10Mm99	126.3	127.2	0.9	49.3	6.6	2.74
10Mm100	127.2	127.8	0.6	45.5	-3.8	1.17
10Mm101	127.8	128.1	0.3	47.9	2.4	0.97
10Mm102	128.1	128.5	0.4	44.9	-3.0	2.39
10Mm103	128.5	128.9	0.4	38.7	-6.2	0.61
10Mm104	128.9	129.4	0.5	36.7	-2.0	0.27
10Mm105	129.4	130	0.6	38.0	1.2	0.76
11Mm1	0	3	3	34.0		0
11Mm2	3	3.6	0.6	45.8		2.52
11Mm3	3.6	4.2	0.6	48.7	2.9	1.31
11Mm4	4.2	4.5	0.3	42.3	-6.5	1.82
11Mm5	4.5	5.2	0.7	48.0	5.7	2.21
11Mm6	5.2	5.6	0.4	43.3	-4.7	1.61
11Mm7	5.6	6.7	1.1	47.4	4.1	1.92
11Mm8	6.7	7.5	0.8	43.5	-4.0	2.37
11Mm9	7.5	8.3	0.8	40.9	-2.6	0.9
11Mm10	8.3	8.6	0.3	47.1	6.2	0.75
11Mm11	8.6	9.1	0.5	43.5	-3.6	1.15
11Mm12	9.1	11.4	2.3	39.0	-4.5	1.03
11Mm13	11.4	12.5	1.1	42.1	3.1	1.09
11Mm14	12.5	15.2	2.7	37.7	-4.4	0.9
11Mm15	15.2	15.9	0.7	36.7	-1.0	0.7
11Mm16	15.9	18.8	2.9	39.8	3.0	1.1
11Mm17	18.8	21	2.2	43.7	4.0	1.84
11Mm18	21	22.4	1.4	39.9	-3.8	0.81
11Mm19	22.4	23.2	0.8	43.5	3.6	1.21
11Mm20	23.2	23.9	0.7	40.5	-3.1	1.87
11Mm21	23.9	24.3	0.4	44.7	4.3	1.26
11Mm22	24.3	31.2	6.9	39.2	-5.5	1.3
11Mm23	31.2	31.5	0.3	43.5	4.2	0.18
11Mm24	31.5	31.7	0.2	47.5	4.0	0
11Mm25	31.7	32.1	0.4	44.3	-3.2	2.52
11Mm26	32.1	33.1	1	46.7	2.5	1.59
11Mm27	33.1	33.4	0.3	40.6	-6.1	2.85
11Mm28	33.4	36.4	3	44.8	4.2	1.85
11Mm29	36.4	38.1	1.7	38.1	-6.7	0.64
11Mm30	38.1	39.8	1.7	36.4	-1.7	0.52
11Mm31	39.8	41.7	1.9	38.7	2.3	1.41
11Mm32	41.7	42.4	0.7	36.6	-2.0	0.53

11Mm33	42.4	43	0.6	39.3	2.7	1
11Mm34	43	43.3	0.3	43.1	3.8	0.61
11Mm35	43.3	43.5	0.2	47.0	3.9	0
11Mm36	43.5	45.9	2.4	42.8	-4.2	1.03
11Mm37	45.9	46.1	0.2	46.9	4.1	0
11Mm38	46.1	46.7	0.6	42.9	-4.0	1.07
11Mm39	46.7	48.2	1.5	39.7	-3.2	0.71
11Mm40	48.2	49.1	0.9	42.8	3.1	1.5
11Mm41	49.1	49.4	0.3	40.1	-2.6	0.46
11Mm42	49.4	49.9	0.5	45.1	5.0	1.95
11Mm43	49.9	50.7	0.8	47.1	2.0	1.87
11Mm44	50.7	51.1	0.4	43.3	-3.8	1.3
11Mm45	51.1	51.8	0.7	47.5	4.1	2.44
11Mm46	51.8	52	0.2	43.7	-3.7	0
11Mm47	52	53	1	46.9	3.2	1.11
11Mm48	53	54.2	1.2	45.7	-1.2	1.53
11Mm49	54.2	54.5	0.3	38.6	-7.2	0.82
11Mm50	54.5	54.7	0.2	44.1	5.5	0
11Mm51	54.7	55.3	0.6	47.9	3.9	0.86
11Mm52	55.3	56.2	0.9	43.4	-4.6	1.09
11Mm53	56.2	56.7	0.5	40.8	-2.6	0.59
11Mm54	56.7	57.4	0.7	44.4	3.7	1.1
11Mm55	57.4	58.2	0.8	47.4	2.9	1.5
11Mm56	58.2	58.7	0.5	43.4	-4.0	1.54
11Mm57	58.7	61.6	2.9	47.9	4.5	1.97
11Mm58	61.6	62.1	0.5	43.1	-4.8	2.44
11Mm59	62.1	62.3	0.2	40.2	-2.8	0
11Mm60	62.3	68	5.7	43.7	3.5	1.78
11Mm61	68	68.4	0.4	47.9	4.2	1.58
11Mm62	68.4	68.7	0.3	45.2	-2.7	0.69
11Mm63	68.7	70.6	1.9	48.3	3.1	1.81
11Mm64	70.6	71.8	1.2	43.9	-4.3	1.72
11Mm65	71.8	72	0.2	48.2	4.3	0
11Mm66	72	72.2	0.2	45.8	-2.4	0
11Mm67	72.2	72.4	0.2	49.4	3.6	0
11Mm68	72.4	72.7	0.3	41.5	-7.9	2.09
11Mm69	72.7	73.1	0.4	48.1	6.7	0.48
11Mm70	73.1	73.9	0.8	39.0	-9.1	1
11Mm71	73.9	74.2	0.3	41.5	2.5	0.68
11Mm72	74.2	74.7	0.5	46.8	5.3	2.9
11Mm73	74.7	74.9	0.2	42.2	-4.6	0
11Mm74	74.9	76.5	1.6	47.6	5.4	2.04
11Mm75	76.5	77	0.5	43.6	-4.0	1.45
11Mm76	77	77.2	0.2	39.4	-4.2	0
11Mm77	77.2	77.6	0.4	43.7	4.3	0.57
11Mm78	77.6	78.4	0.8	48.2	4.5	1.95
11Mm79	78.4	78.6	0.2	42.3	-6.0	0
11Mm80	78.6	79	0.4	48.5	6.2	1.32
11Mm81	79	79.4	0.4	42.3	-6.2	2.98
11Mm82	79.4	79.7	0.3	49.3	7.0	1.77
11Mm83	79.7	80.6	0.9	43.5	-5.8	1.96
11Mm84	80.6	81	0.4	46.5	3.0	0.48
11Mm85	81	82	1	44.6	-1.9	0.88
11Mm86	82	82.8	0.8	47.6	3.0	1.92
11Mm87	82.8	84	1.2	44.1	-3.5	1.86
11Mm88	84	84.2	0.2	40.1	-3.9	0

11Mm89	84.2	84.8	0.6	46.6	6.5	2.01
11Mm90	84.8	85.6	0.8	42.0	-4.6	2.06
11Mm91	85.6	85.8	0.2	48.4	6.4	0
11Mm92	85.8	86.2	0.4	39.9	-8.5	1.11
11Mm93	86.2	86.4	0.2	43.4	3.5	0
11Mm94	86.4	86.6	0.2	39.9	-3.5	0
11Mm95	86.6	87.5	0.9	43.6	3.7	1.81
11Mm96	87.5	87.7	0.2	49.1	5.5	0
11Mm97	87.7	87.9	0.2	45.2	-3.9	0
11Mm98	87.9	89.2	1.3	47.3	2.1	1.11
11Mm99	89.2	90.6	1.4	43.0	-4.3	1.33
11Mm100	90.6	93.2	2.6	38.4	-4.6	1.06
11Mm101	93.2	94.2	1	43.4	5.0	2.35
11Mm102	94.2	95.2	1	49.4	6.0	2.16
11Mm103	95.2	95.4	0.2	45.7	-3.6	0
11Mm104	95.4	96.3	0.9	47.6	1.8	1.62
11Mm105	96.3	96.6	0.3	43.6	-3.9	1.53
11Mm106	96.6	99	2.4	48.5	4.9	2.9
11Mm107	99	99.9	0.9	43.3	-5.2	1.49
11Mm108	99.9	101.2	1.3	48.3	5.0	1.45
11Mm109	101.2	101.6	0.4	45.9	-2.4	0.23
11Mm110	101.6	102.3	0.7	49.5	3.6	0.88
11Mm111	102.3	102.6	0.3	45.2	-4.3	1.25
11Mm112	102.6	103.3	0.7	49.3	4.1	0.76
11Mm113	103.3	103.5	0.2	42.1	-7.3	0
11Mm114	103.5	104.2	0.7	48.1	6.0	2.27
11Mm115	104.2	105.4	1.2	42.4	-5.7	3.06
11Mm116	105.4	105.7	0.3	38.6	-3.8	1.38
11Mm117	105.7	106.1	0.4	44.8	6.3	4.12
11Mm118	106.1	106.6	0.5	47.4	2.5	1.59
11Mm119	106.6	107.1	0.5	44.0	-3.4	2.19
11Mm120	107.1	107.5	0.4	45.7	1.7	0.99
11Mm121	107.5	107.9	0.4	47.5	1.8	1.16
11Mm122	107.9	108.3	0.4	44.5	-3.0	0.78
11Mm123	108.3	108.7	0.4	40.1	-4.4	1.51
11Mm124	108.7	109.5	0.8	46.7	6.6	0.83
11Mm125	109.5	109.8	0.3	44.3	-2.4	0.98
11Mm126	109.8	110.2	0.4	39.9	-4.5	0.58
11Mm127	110.2	110.5	0.3	43.1	3.3	1.32
11Mm128	110.5	112.4	1.9	40.0	-3.2	0.92
11Mm129	112.4	112.8	0.4	45.1	5.2	1.51
11Mm130	112.8	119.8	7	50.0	4.9	1.89
11Mm131	119.8	120	0.2	53.3	3.3	0
11Mm132	120	121.2	1.2	49.6	-3.8	2.21
11Mm133	121.2	121.7	0.5	45.2	-4.4	1.12
11Mm134	121.7	121.9	0.2	39.7	-5.5	0
12Mm1	0	3	3	48.0		0
12Mm2	3	3.8	0.8	42.7		2
12Mm3	3.8	4.3	0.5	47.5	4.8	2.8
12Mm4	4.3	4.7	0.4	40.8	-6.8	0.94
12Mm5	4.7	5.1	0.4	43.3	2.5	2.68
12Mm6	5.1	5.3	0.2	48.4	5.1	0
12Mm7	5.3	5.8	0.5	42.6	-5.7	1.75
12Mm8	5.8	8	2.2	38.8	-3.8	1.13
12Mm9	8	8.3	0.3	42.8	4.0	1.12
12Mm10	8.3	8.5	0.2	48.9	6.1	0

12Mm11	8.5	9.6	1.1	43.1	-5.7	2.07
12Mm12	9.6	12.2	2.6	39.8	-3.3	1.33
12Mm13	12.2	13.2	1	44.1	4.2	1.37
12Mm14	13.2	16.5	3.3	39.9	-4.1	1.97
12Mm15	16.5	17.3	0.8	45.9	6.0	3.35
12Mm16	17.3	17.5	0.2	45.5	-0.4	0
12Mm17	17.5	17.8	0.3	47.8	2.3	1.04
12Mm18	17.8	21.1	3.3	44.8	-2.9	0.66
12Mm19	21.1	21.3	0.2	46.7	1.9	0
12Mm20	21.3	29	7.7	43.8	-2.9	1.74
12Mm21	29	29.2	0.2	43.7	-0.2	0
12Mm22	29.2	29.6	0.4	47.3	3.6	0.74
12Mm23	29.6	32.3	2.7	43.2	-4.1	1.88
12Mm24	32.3	32.6	0.3	39.7	-3.5	1.07
12Mm25	32.6	33	0.4	42.4	2.8	0.4
12Mm26	33	33.2	0.2	40.6	-1.9	0
12Mm27	33.2	33.7	0.5	42.5	1.9	0.89
12Mm28	33.7	34.1	0.4	46.5	4.0	0.24
12Mm29	34.1	37.8	3.7	39.4	-7.1	1.31
12Mm30	37.8	39.2	1.4	36.7	-2.7	0.59
12Mm31	39.2	40.9	1.7	38.3	1.6	1.19
12Mm32	40.9	41.2	0.3	41.8	3.5	0.45
12Mm33	41.2	41.5	0.3	40.3	-1.5	0.31
12Mm34	41.5	41.7	0.2	42.0	1.7	0
12Mm35	41.7	42.3	0.6	39.1	-2.9	0.88
12Mm36	42.3	42.6	0.3	0.0		0
12Mm37	42.6	44	1.4	37.7		0.69
12Mm38	44	44.7	0.7	36.3	-1.4	0.43
12Mm39	44.7	48.5	3.8	38.5	2.2	1.37
12Mm40	48.5	49.5	1	36.5	-2.0	0.42
12Mm41	49.5	49.7	0.2	37.8	1.3	0
12Mm42	49.7	50	0.3	36.2	-1.6	0.51
12Mm43	50	51	1	38.2	2.0	0.99
12Mm44	51	51.3	0.3	36.3	-1.9	0.74
12Mm45	51.3	52.8	1.5	39.8	3.6	1.65
12Mm46	52.8	53.2	0.4	42.2	2.4	0.87
12Mm47	53.2	53.4	0.2	40.5	-1.8	0
12Mm48	53.4	54.1	0.7	41.4	1.0	2.22
12Mm49	54.1	54.8	0.7	40.4	-1.1	1
12Mm50	54.8	56.7	1.9	43.4	3.0	1.53
12Mm51	56.7	57.6	0.9	40.2	-3.2	1.48
12Mm52	57.6	57.8	0.2	43.3	3.2	0
12Mm53	57.8	61.6	3.8	39.3	-4.0	1.54
12Mm54	61.6	65.4	3.8	36.6	-2.7	0.73
12Mm55	65.4	67.7	2.3	38.4	1.8	1.21
12Mm56	67.7	68.3	0.6	36.2	-2.2	0.61
12Mm57	68.3	68.7	0.4	37.3	1.2	0.84
12Mm58	68.7	69.9	1.2	37.9	0.6	1.21
12Mm59	69.9	74.2	4.3	43.1	5.2	1.38
12Mm60	74.2	74.4	0.2	40.1	-3.0	0
12Mm61	74.4	75.5	1.1	43.3	3.2	1.81
12Mm62	75.5	76.4	0.9	39.4	-3.9	1.06
12Mm63	76.4	77.6	1.2	43.9	4.5	1.15
12Mm64	77.6	78.1	0.5	47.2	3.3	1.74
12Mm65	78.1	78.3	0.2	44.2	-2.9	0
12Mm66	78.3	78.6	0.3	38.3	-5.9	1.49

12Mm67	78.6	79.3	0.7	42.5	4.2	1.79
12Mm68	79.3	80	0.7	37.6	-4.9	1.71
12Mm69	80	80.2	0.2	46.7	9.1	0
12Mm70	80.2	80.4	0.2	44.2	-2.5	0
12Mm71	80.4	80.7	0.3	40.7	-3.6	0.48
12Mm72	80.7	80.9	0.2	43.4	2.7	0
12Mm73	80.9	81.4	0.5	47.2	3.9	1.26
12Mm74	81.4	82.5	1.1	44.6	-2.6	1.83
12Mm75	82.5	82.7	0.2	40.3	-4.4	0
12Mm76	82.7	84.2	1.5	44.4	4.1	1.43
12Mm77	84.2	84.9	0.7	46.8	2.4	0.69
12Mm78	84.9	85.9	1	44.3	-2.5	1.92
12Mm79	85.9	86.3	0.4	48.0	3.7	0.8
12Mm80	86.3	87.1	0.8	45.8	-2.2	2.41
12Mm81	87.1	87.4	0.3	44.2	-1.6	1.15
12Mm82	87.4	88.5	1.1	47.8	3.6	1.01
12Mm83	88.5	88.9	0.4	43.7	-4.1	0.5
12Mm84	88.9	90.1	1.2	42.5	-1.1	1.75
12Mm85	90.1	92.5	2.4	39.1	-3.5	0.82
12Mm86	92.5	93.2	0.7	42.9	3.8	1.5
12Mm87	93.2	94.8	1.6	37.9	-5.0	1.19
12Mm88	94.8	96.6	1.8	36.7	-1.1	0.57
12Mm89	96.6	97.4	0.8	38.2	1.5	0.55
12Mm90	97.4	98.4	1	36.6	-1.6	0.49
12Mm91	98.4	99.4	1	39.1	2.5	1.63
12Mm92	99.4	100	0.6	43.0	3.9	0.87
12Mm93	100	100.2	0.2	40.0	-3.0	0
12Mm94	100.2	101.8	1.6	45.5	5.5	1.58
12Mm95	101.8	102	0.2	40.3	-5.2	0
12Mm96	102	102.3	0.3	47.3	7.0	2.23
12Mm97	102.3	103	0.7	39.5	-7.8	1.23
12Mm98	103	103.3	0.3	43.3	3.8	1.5
12Mm99	103.3	104	0.7	48.2	4.9	2.03
12Mm100	104	104.4	0.4	41.9	-6.3	1.29
12Mm101	104.4	105.3	0.9	46.6	4.7	2.45
12Mm102	105.3	105.6	0.3	45.0	-1.6	1.02
12Mm103	105.6	107.5	1.9	47.8	2.8	1.46
12Mm104	107.5	108.9	1.4	45.7	-2.1	0.62
12Mm105	108.9	110.4	1.5	48.5	2.9	2.32
12Mm106	110.4	110.6	0.2	40.5	-8.0	0
12Mm107	110.6	110.8	0.2	49.1	8.6	0
12Mm108	110.8	111.1	0.3	44.2	-4.9	0.48
12Mm109	111.1	114.6	3.5	49.2	5.1	2.65
12Mm110	114.6	117.3	2.7	39.5	-9.7	1.36
12Mm111	117.3	118.3	1	43.5	3.9	1.72
12Mm112	118.3	118.6	0.3	46.3	2.9	0.28
12Mm113	118.6	119.3	0.7	42.9	-3.4	1.05
12Mm114	119.3	121.1	1.8	39.5	-3.3	1.48
12Mm115	121.1	121.3	0.2	36.6	-2.9	0
13Mm1	0	3	3	36.0		0
13Mm2	3	3.4	0.4	42.2		0.63
13Mm3	3.4	3.6	0.2	40.8	-1.4	0
13Mm4	3.6	4	0.4	43.2	2.4	0.94
13Mm5	4	6.5	2.5	38.8	-4.4	0.99
13Mm6	6.5	6.8	0.3	43.2	4.4	1.93
13Mm7	6.8	8.5	1.7	38.9	-4.3	1.06

13Mm8	8.5	9.3	0.8	42.5	3.5	0.73
13Mm9	9.3	9.6	0.3	40.1	-2.4	0.56
13Mm10	9.6	10	0.4	41.9	1.8	1.03
13Mm11	10	12	2	39.9	-2.0	0.89
13Mm12	12	12.8	0.8	44.3	4.4	1.4
13Mm13	12.8	13.4	0.6	38.2	-6.2	0.55
13Mm14	13.4	14.2	0.8	42.5	4.4	2.24
13Mm15	14.2	15.5	1.3	39.3	-3.2	0.62
13Mm16	15.5	15.7	0.2	41.9	2.5	0
13Mm17	15.7	20.1	4.4	39.7	-2.2	1.16
13Mm18	20.1	20.8	0.7	42.4	2.8	0.7
13Mm19	20.8	21.2	0.4	40.5	-1.9	0.45
13Mm20	21.2	21.6	0.4	42.3	1.8	0.57
13Mm21	21.6	21.8	0.2	39.4	-2.9	0
13Mm22	21.8	22.2	0.4	42.5	3.2	0.88
13Mm23	22.2	23.5	1.3	40.3	-2.2	0.48
13Mm24	23.5	25.5	2	43.3	3.0	1.32
13Mm25	25.5	28.6	3.1	38.1	-5.2	1.05
13Mm26	28.6	32	3.4	42.9	4.8	1.46
13Mm27	32	32.4	0.4	38.7	-4.2	0.91
13Mm28	32.4	33.2	0.8	42.0	3.2	0.75
13Mm29	33.2	34.1	0.9	40.3	-1.6	1.04
13Mm30	34.1	39.1	5	43.3	3.0	1.63
13Mm31	39.1	40.5	1.4	40.0	-3.2	0.73
13Mm32	40.5	43.2	2.7	43.3	3.2	1.23
13Mm33	43.2	43.4	0.2	47.1	3.9	0
13Mm34	43.4	48.7	5.3	43.9	-3.3	1.36
13Mm35	48.7	49.5	0.8	48.2	4.4	1.81
13Mm36	49.5	51.8	2.3	43.9	-4.4	2.1
13Mm37	51.8	52.3	0.5	48.8	4.9	1.11
13Mm38	52.3	52.7	0.4	45.1	-3.7	0.58
13Mm39	52.7	53.4	0.7	47.5	2.4	0.93
13Mm40	53.4	54.2	0.8	45.0	-2.5	1.01
13Mm41	54.2	54.5	0.3	48.2	3.2	1.56
13Mm42	54.5	54.7	0.2	45.6	-2.6	0
13Mm43	54.7	57	2.3	47.9	2.3	2.42
13Mm44	57	58	1	45.2	-2.7	1.02
13Mm45	58	58.2	0.2	47.7	2.5	0
13Mm46	58.2	60.9	2.7	45.2	-2.5	0.99
13Mm47	60.9	62.9	2	40.2	-5.0	0.96
13Mm48	62.9	64.6	1.7	43.7	3.4	1.97
13Mm49	64.6	67.9	3.3	40.3	-3.4	0.84
13Mm50	67.9	73.6	5.7	43.6	3.3	1.5
13Mm51	73.6	74	0.4	47.8	4.2	1.39
13Mm52	74	74.5	0.5	44.2	-3.6	1.63
13Mm53	74.5	75.9	1.4	39.6	-4.6	1.1
13Mm54	75.9	76.2	0.3	41.5	1.8	0.94
13Mm55	76.2	87.8	11.6	38.1	-3.3	1.35
13Mm56	87.8	89.5	1.7	36.3	-1.8	0.57
13Mm57	89.5	91.3	1.8	38.8	2.5	1.4
13Mm58	91.3	91.6	0.3	42.0	3.2	0.59
13Mm59	91.6	92.2	0.6	40.9	-1.1	1.41
13Mm60	92.2	92.7	0.5	0.0	-40.9	0
13Mm61	92.7	93.9	1.2	43.1	43.1	1.23
13Mm62	93.9	94.3	0.4	40.2	-2.8	1.29
13Mm63	94.3	97.5	3.2	43.5	3.3	1.62

13Mm64	97.5	97.7	0.2	39.8	-3.7	0
13Mm65	97.7	102.7	5	43.4	3.6	1.59
13Mm66	102.7	103.3	0.6	40.1	-3.4	0.65
13Mm67	103.3	104.9	1.6	42.7	2.7	1.6
13Mm68	104.9	105.2	0.3	37.4	-5.3	2.11
13Mm69	105.2	107.8	2.6	39.6	2.1	1.78
13Mm70	107.8	109.2	1.4	43.0	3.4	1.34
13Mm71	109.2	110.4	1.2	39.6	-3.3	0.87
13Mm72	110.4	110.9	0.5	41.9	2.2	0.74
13Mm73	110.9	112	1.1	40.3	-1.6	0.78
13Mm74	112	115	3	43.5	3.3	1.3
13Mm75	115	115.9	0.9	40.9	-2.6	0.6
13Mm76	115.9	118.4	2.5	38.6	-2.4	1.55
13Mm77	118.4	118.8	0.4	36.3	-2.3	0.44
13Mm78	118.8	120.3	1.5	38.3	2.0	1.29
14Mm1	0	3	3	50.0		0
14Mm2	3	9.3	6.3	42.8		1.13
14Mm3	9.3	12.3	3	40.3	-2.5	0.92
14Mm4	12.3	13.5	1.2	43.1	2.8	0.91
14Mm5	13.5	17.3	3.8	40.5	-2.5	0.85
14Mm6	17.3	17.6	0.3	42.6	2.1	0.83
14Mm7	17.6	18.7	1.1	40.5	-2.1	0.58
14Mm8	18.7	20.5	1.8	41.8	1.3	0.95
14Mm9	20.5	20.9	0.4	44.0	2.2	0.77
14Mm10	20.9	21.1	0.2	47.8	3.8	0
14Mm11	21.1	21.9	0.8	43.1	-4.7	3.04
14Mm12	21.9	22.2	0.3	39.3	-3.8	0.81
14Mm13	22.2	24.6	2.4	42.9	3.7	1.72
14Mm14	24.6	25.1	0.5	46.7	3.8	1.08
14Mm15	25.1	25.4	0.3	44.7	-2.0	0.64
14Mm16	25.4	27.2	1.8	47.9	3.2	1.99
14Mm17	27.2	27.8	0.6	40.7	-7.3	0.71
14Mm18	27.8	31.3	3.5	43.4	2.7	1.87
14Mm19	31.3	32.4	1.1	46.3	2.9	2.85
14Mm20	32.4	33.1	0.7	42.9	-3.4	3.35
14Mm21	33.1	34.2	1.1	46.3	3.4	1.47
14Mm22	34.2	34.7	0.5	43.4	-2.9	1.88
14Mm23	34.7	35.8	1.1	45.4	2.0	2.46
14Mm24	35.8	36.1	0.3	43.6	-1.8	1
14Mm25	36.1	37.8	1.7	39.8	-3.8	1.2
14Mm26	37.8	38.1	0.3	42.6	2.8	0.97
14Mm27	38.1	41.6	3.5	38.2	-4.4	1.04
14Mm28	41.6	49.6	8	42.6	4.4	1.58
14Mm29	49.6	51.4	1.8	39.5	-3.0	1.29
14Mm30	51.4	52	0.6	43.4	3.9	1.48
14Mm31	52	52.2	0.2	38.5	-4.9	0
14Mm32	52.2	53	0.8	43.4	4.9	1.9
14Mm33	53	54.7	1.7	41.1	-2.3	0.51
14Mm34	54.7	55	0.3	44.5	3.4	1.21
14Mm35	55	55.8	0.8	47.8	3.3	2.02
14Mm36	55.8	56.1	0.3	43.8	-4.0	1.09
14Mm37	56.1	56.6	0.5	48.3	4.6	1.37
14Mm38	56.6	57.6	1	41.9	-6.5	1.95
14Mm39	57.6	60.2	2.6	42.8	0.9	1.86
14Mm40	60.2	63.7	3.5	43.8	1.0	1.81
14Mm41	63.7	64.1	0.4	47.4	3.7	0.61

14Mm42	64.1	64.3	0.2	43.9	-3.5	0
14Mm43	64.3	64.8	0.5	47.5	3.6	1.04
14Mm44	64.8	65.6	0.8	43.9	-3.6	1.31
14Mm45	65.6	65.8	0.2	46.5	2.6	0
14Mm46	65.8	66.7	0.9	45.1	-1.5	1.9
14Mm47	66.7	67	0.3	46.3	1.3	0.83
14Mm48	67	67.4	0.4	43.4	-3.0	0.62
14Mm49	67.4	67.6	0.2	46.5	3.1	0
14Mm50	67.6	68.9	1.3	43.9	-2.5	1.33
14Mm51	68.9	69.5	0.6	39.8	-4.1	0.55
14Mm52	69.5	69.8	0.3	42.6	2.8	1.58
14Mm53	69.8	70.6	0.8	47.9	5.2	1.59
14Mm54	70.6	70.8	0.2	44.6	-3.3	0
14Mm55	70.8	71.4	0.6	47.1	2.5	2.93
14Mm56	71.4	71.9	0.5	42.2	-4.8	1.11
14Mm57	71.9	72.5	0.6	40.9	-1.4	0.24
14Mm58	72.5	72.9	0.4	43.9	3.1	1.73
14Mm59	72.9	73.2	0.3	40.1	-3.8	0.94
14Mm60	73.2	74.1	0.9	42.1	2.0	1.53
14Mm61	74.1	75	0.9	39.6	-2.5	0.7
14Mm62	75	80.5	5.5	43.3	3.7	1.64
14Mm63	80.5	81.5	1	38.7	-4.6	1.47
14Mm64	81.5	86.2	4.7	36.3	-2.4	0.66
14Mm65	86.2	88.6	2.4	39.2	2.9	1.18
14Mm66	88.6	93.2	4.6	36.2	-3.0	0.73
14Mm67	93.2	93.5	0.3	37.5	1.3	0.49
14Mm68	93.5	98.6	5.1	36.2	-1.3	0.68
14Mm69	98.6	99.9	1.3	40.1	3.9	2
14Mm70	99.9	101.9	2	40.0	-0.1	0.95
14Mm71	101.9	102.3	0.4	42.2	2.1	0.72
14Mm72	102.3	103.2	0.9	39.3	-2.9	0.76
14Mm73	103.2	103.5	0.3	42.5	3.2	1.1
14Mm74	103.5	106.9	3.4	40.3	-2.1	1.35
14Mm75	106.9	114.4	7.5	36.4	-3.9	0.69
14Mm76	114.4	118.2	3.8	38.7	2.3	1.22
14Mm77	118.2	119.4	1.2	44.9	6.2	1.24
14Mm78	119.4	120.5	1.1	41.0	-3.9	0.85
14Mm79	120.5	121.6	1.1	44.5	3.6	1.24
14Mm80	121.6	121.8	0.2	47.7	3.2	0
14Mm81	121.8	122.9	1.1	45.2	-2.5	1
14Mm82	122.9	123.2	0.3	40.6	-4.6	0.29
14Mm83	123.2	123.5	0.3	42.2	1.6	0.84
14Mm84	123.5	125.2	1.7	38.3	-3.9	1.01
15Mm1	0	3	3	0.0		0
15Mm2	2.9	3.2	0.3	42.1		0.19
15Mm3	3.2	5	1.8	39.4	-2.7	0.96
15Mm4	5	5.2	0.2	43.0	3.6	0
15Mm5	5.2	6.8	1.6	39.9	-3.0	1.38
15Mm6	6.8	9.1	2.3	42.3	2.4	1.76
15Mm7	9.1	10.5	1.4	39.7	-2.7	1.21
15Mm8	10.5	11.4	0.9	42.6	2.9	1.18
15Mm9	11.4	11.7	0.3	40.4	-2.1	0.48
15Mm10	11.7	13.2	1.5	43.9	3.5	1.75
15Mm11	13.2	15.1	1.9	37.3	-6.6	0.98
15Mm12	15.1	18.9	3.8	36.1	-1.2	0.61
15Mm13	18.9	20	1.1	37.5	1.4	0.84

15Mm14	20	21.7	1.7	36.4	-1.1	0.62
15Mm15	21.7	21.9	0.2	37.8	1.3	0
15Mm16	21.9	24.2	2.3	36.7	-1.1	0.66
15Mm17	24.2	25.2	1	38.7	2.0	0.92
15Mm18	25.2	25.6	0.4	43.9	5.2	1.38
15Mm19	25.6	25.9	0.3	46.1	2.2	0.59
15Mm20	25.9	27.2	1.3	40.4	-5.7	0.77
15Mm21	27.2	27.6	0.4	44.7	4.3	1.66
15Mm22	27.6	27.9	0.3	47.2	2.5	0.29
15Mm23	27.9	28.3	0.4	42.9	-4.3	1.78
15Mm24	28.3	30.9	2.6	39.0	-3.9	1.6
15Mm25	30.9	31.1	0.2	43.3	4.3	0
15Mm26	31.1	31.4	0.3	47.1	3.8	0.59
15Mm27	31.4	32.3	0.9	42.5	-4.5	1.19
15Mm28	32.3	34.1	1.8	40.0	-2.5	0.92
15Mm29	34.1	34.8	0.7	42.9	2.9	1.32
15Mm30	34.8	35.1	0.3	39.6	-3.3	0.83
15Mm31	35.1	35.3	0.2	42.1	2.5	0
15Mm32	35.3	35.8	0.5	38.7	-3.4	1.45
15Mm33	35.8	38.9	3.1	44.6	5.9	1.8
15Mm34	38.9	39.2	0.3	39.8	-4.8	1.57
15Mm35	39.2	39.4	0.2	36.0	-3.8	0
15Mm36	39.4	40	0.6	41.8	5.8	2.08
15Mm37	40	40.5	0.5	38.9	-2.9	0.98
15Mm38	40.5	40.7	0.2	36.5	-2.4	0
15Mm39	40.7	46.2	5.5	38.1	1.6	1.11
15Mm40	46.2	47.2	1	36.4	-1.8	0.82
15Mm41	47.2	47.4	0.2	38.2	1.9	0
15Mm42	47.4	49	1.6	35.7	-2.5	1.19
15Mm43	49	49.2	0.2	37.8	2.1	0
15Mm44	49.2	50.2	1	36.3	-1.5	0.56
15Mm45	50.2	50.5	0.3	38.0	1.7	0.77
15Mm46	50.5	50.7	0.2	36.3	-1.7	0
15Mm47	50.7	51.4	0.7	37.9	1.6	0.86
15Mm48	51.4	52	0.6	41.7	3.8	0.65
15Mm49	52	52.9	0.9	39.7	-2.0	0.57
15Mm50	52.9	53.2	0.3	42.6	2.9	0.38
15Mm51	53.2	54.6	1.4	39.4	-3.2	0.74
15Mm52	54.6	55.7	1.1	42.1	2.7	1.15
15Mm53	55.7	57.3	1.6	38.9	-3.2	1.13
15Mm54	57.3	57.5	0.2	42.7	3.7	0
15Mm55	57.5	57.7	0.2	46.8	4.2	0
15Mm56	57.7	59.4	1.7	44.5	-2.4	1.7
15Mm57	59.4	59.7	0.3	46.6	2.1	0.82
15Mm58	59.7	60.5	0.8	39.9	-6.7	1.14
15Mm59	60.5	60.7	0.2	41.8	1.9	0
15Mm60	60.7	61.6	0.9	39.6	-2.2	0.7
15Mm61	61.6	62.2	0.6	43.5	3.9	2.11
15Mm62	62.2	63.5	1.3	39.2	-4.4	0.84
15Mm63	63.5	64.6	1.1	43.4	4.2	1.75
15Mm64	64.6	65.7	1.1	39.5	-3.8	0.86
15Mm65	65.7	66.2	0.5	42.2	2.7	0.4
15Mm66	66.2	66.5	0.3	39.4	-2.8	1.61
15Mm67	66.5	67.3	0.8	44.7	5.3	2.06
15Mm68	67.3	67.8	0.5	39.5	-5.1	1.02
15Mm69	67.8	68.7	0.9	42.3	2.8	1.99

15Mm70	68.7	69	0.3	42.9	0.7	0.74
15Mm71	69	71.6	2.6	38.4	-4.5	1.14
15Mm72	71.6	72.5	0.9	43.2	4.8	1.68
15Mm73	72.5	73	0.5	47.3	4.0	0.9
15Mm74	73	73.3	0.3	43.2	-4.1	3.07
15Mm75	73.3	74.8	1.5	49.7	6.6	1.97
15Mm76	74.8	75.4	0.6	44.5	-5.2	0.47
15Mm77	75.4	76.9	1.5	50.1	5.6	2.27
15Mm78	76.9	77.1	0.2	43.4	-6.7	0
15Mm79	77.1	80.5	3.4	49.8	6.4	2.47
15Mm80	80.5	81.9	1.4	45.5	-4.3	2.74
15Mm81	81.9	82.3	0.4	48.5	3.0	1.93
15Mm82	82.3	82.7	0.4	43.4	-5.1	0.12
15Mm83	82.7	86.7	4	49.2	5.8	2.16
15Mm84	86.7	87.3	0.6	45.4	-3.8	0.83
15Mm85	87.3	89.4	2.1	47.6	2.2	1.84
15Mm86	89.4	89.6	0.2	41.6	-5.9	0
15Mm87	89.6	91.1	1.5	40.2	-1.4	0.67
15Mm88	91.1	91.5	0.4	41.8	1.5	0.57
15Mm89	91.5	92.8	1.3	40.5	-1.3	0.92
15Mm90	92.8	97.5	4.7	43.4	2.9	1.54
15Mm91	97.5	98	0.5	49.1	5.7	1.92
15Mm92	98	98.4	0.4	44.0	-5.1	1.32
15Mm93	98.4	99.8	1.4	48.7	4.7	1.64
15Mm94	99.8	100	0.2	44.1	-4.6	0
15Mm95	100	102.2	2.2	48.4	4.3	2.01
15Mm96	102.2	102.8	0.6	44.5	-3.8	1.91
15Mm97	102.8	103.2	0.4	47.5	3.0	2.07
15Mm98	103.2	103.5	0.3	43.6	-3.9	2.31
16Mm1	0	3	3	40.0		0
16Mm2	3	3.6	0.6	40.0		1.53
16Mm3	3.6	3.9	0.3	43.5	3.5	0.63
16Mm4	3.9	4.1	0.2	46.6	3.1	0
16Mm5	4.1	4.5	0.4	43.8	-2.7	2.5
16Mm6	4.5	5.6	1.1	47.4	3.6	1.67
16Mm7	5.6	6.5	0.9	43.5	-3.9	1.08
16Mm8	6.5	6.7	0.2	40.4	-3.1	0
16Mm9	6.7	7.7	1	41.7	1.3	0.62
16Mm10	7.7	7.9	0.2	40.8	-0.9	0
16Mm11	7.9	8.4	0.5	42.4	1.5	0.95
16Mm12	8.4	8.8	0.4	47.1	4.8	1.22
16Mm13	8.8	10.3	1.5	43.7	-3.4	1.33
16Mm14	10.3	11.1	0.8	48.5	4.8	1.31
16Mm15	11.1	11.3	0.2	43.3	-5.2	0
16Mm16	11.3	11.5	0.2	46.9	3.6	0
16Mm17	11.5	14.5	3	43.9	-3.0	1.73
16Mm18	14.5	16.8	2.3	40.2	-3.7	0.82
16Mm19	16.8	17.5	0.7	44.9	4.7	1.68
16Mm20	17.5	18.8	1.3	48.4	3.5	1.62
16Mm21	18.8	19	0.2	43.2	-5.2	0
16Mm22	19	19.8	0.8	39.0	-4.1	0.68
16Mm23	19.8	20.5	0.7	42.6	3.6	1.44
16Mm24	20.5	20.8	0.3	47.6	5.0	0.75
16Mm25	20.8	21.2	0.4	42.9	-4.7	1.05
16Mm26	21.2	21.4	0.2	49.3	6.4	0
16Mm27	21.4	24.6	3.2	44.0	-5.3	1.37

16Mm28	24.6	28.8	4.2	39.4	-4.6	1.28
16Mm29	28.8	30	1.2	43.4	4.1	2.14
16Mm30	30	31.1	1.1	46.6	3.2	2.23
16Mm31	31.1	31.4	0.3	43.6	-3.0	0.42
16Mm32	31.4	31.6	0.2	47.5	3.8	0
16Mm33	31.6	32.5	0.9	43.0	-4.4	2.4
16Mm34	32.5	32.8	0.3	47.1	4.0	0.56
16Mm35	32.8	33.8	1	44.2	-2.9	2.13
16Mm36	33.8	34.1	0.3	46.7	2.5	0.33
16Mm37	34.1	35.1	1	45.0	-1.7	1.81
16Mm38	35.1	35.7	0.6	47.5	2.5	2.74
16Mm39	35.7	36.1	0.4	43.2	-4.3	1.09
16Mm40	36.1	36.4	0.3	40.6	-2.6	0.13
16Mm41	36.4	37.1	0.7	41.8	1.2	1.86
16Mm42	37.1	37.4	0.3	36.6	-5.2	0.65
16Mm43	37.4	39.2	1.8	43.3	6.7	1.48
16Mm44	39.2	43.5	4.3	38.3	-5.1	1.14
16Mm45	43.5	44.8	1.3	42.7	4.4	1.07
16Mm46	44.8	45.6	0.8	40.0	-2.6	0.83
16Mm47	45.6	46.1	0.5	41.5	1.5	0.29
16Mm48	46.1	48.2	2.1	38.6	-2.9	1.3
16Mm49	48.2	48.6	0.4	41.8	3.1	0.51
16Mm50	48.6	49.8	1.2	39.3	-2.4	1.23
16Mm51	49.8	50.2	0.4	42.2	2.9	0.63
16Mm52	50.2	53.2	3	38.6	-3.7	1.38
16Mm53	53.2	54.8	1.6	36.2	-2.3	0.46
16Mm54	54.8	55.7	0.9	38.6	2.4	0.91
16Mm55	55.7	56.1	0.4	42.9	4.3	0.78
16Mm56	56.1	57.2	1.1	40.1	-2.8	0.9
16Mm57	57.2	57.5	0.3	42.7	2.6	0.76
16Mm58	57.5	59.4	1.9	39.5	-3.2	1.5
16Mm59	59.4	59.6	0.2	42.4	2.9	0
16Mm60	59.6	60	0.4	38.7	-3.7	0.38
16Mm61	60	60.7	0.7	36.5	-2.2	0.3
16Mm62	60.7	61	0.3	37.6	1.0	0.5
16Mm63	61	61.2	0.2	35.3	-2.2	0
16Mm64	61.2	61.4	0.2	37.5	2.1	0
16Mm65	61.4	62.2	0.8	36.0	-1.4	0.5
16Mm66	62.2	63.5	1.3	38.7	2.7	1.19
16Mm67	63.5	64.2	0.7	36.5	-2.2	0.55
16Mm68	64.2	66.6	2.4	38.3	1.8	0.96
16Mm69	66.6	69.8	3.2	35.8	-2.5	0.62
16Mm70	69.8	71	1.2	38.2	2.4	0.89
16Mm71	71	72.3	1.3	36.6	-1.6	0.58
16Mm72	72.3	73.9	1.6	38.7	2.2	1.1
16Mm73	73.9	74.1	0.2	36.1	-2.6	0
16Mm74	74.1	74.8	0.7	37.1	1.1	0.63
16Mm75	74.8	75.6	0.8	36.5	-0.6	0.55
16Mm76	75.6	76.1	0.5	39.0	2.5	0.57
16Mm77	76.1	76.4	0.3	41.8	2.9	0.72
16Mm78	76.4	77.7	1.3	38.7	-3.2	1.69
16Mm79	77.7	78.2	0.5	38.8	0.1	0.67
16Mm80	78.2	78.6	0.4	42.9	4.1	0.38
16Mm81	78.6	84.5	5.9	36.7	-6.2	1.07
16Mm82	84.5	85.2	0.7	41.8	5.1	0.9
16Mm83	85.2	87.2	2	38.8	-3.1	1.34

16Mm84	87.2	87.9	0.7	43.2	4.4	1.43
16Mm85	87.9	89.5	1.6	38.9	-4.3	0.87
16Mm86	89.5	89.8	0.3	43.7	4.8	1.35
16Mm87	89.8	90.9	1.1	46.8	3.2	1.27
16Mm88	90.9	91.2	0.3	44.8	-2.0	1.03
16Mm89	91.2	91.6	0.4	47.1	2.3	0.85
16Mm90	91.6	91.8	0.2	44.8	-2.4	0
16Mm91	91.8	92.9	1.1	47.1	2.4	1.2
16Mm92	92.9	93.9	1	45.8	-1.3	1.67
16Mm93	93.9	94.5	0.6	48.2	2.4	0.23
16Mm94	94.5	94.9	0.4	43.5	-4.7	2.5
16Mm95	94.9	95.1	0.2	47.2	3.6	0
16Mm96	95.1	95.8	0.7	45.4	-1.8	0.81
16Mm97	95.8	96.4	0.6	46.1	0.7	1.71
16Mm98	96.4	98.4	2	44.2	-1.9	1.71
17Mm1	0	3	3	58.0		0
17Mm2	3	3.6	0.6	43.5		1.86
17Mm3	3.6	4.4	0.8	40.2	-3.3	0.56
17Mm4	4.4	5.2	0.8	43.6	3.4	1.7
17Mm5	5.2	5.4	0.2	47.1	3.5	0
17Mm6	5.4	5.7	0.3	45.8	-1.3	0.14
17Mm7	5.7	7.7	2	46.9	1.1	1.35
17Mm8	7.7	8	0.3	44.6	-2.2	0.24
17Mm9	8	9.2	1.2	46.2	1.6	0.77
17Mm10	9.2	10.9	1.7	42.6	-3.6	2.37
17Mm11	10.9	11.4	0.5	39.9	-2.7	1.03
17Mm12	11.4	12.3	0.9	43.3	3.4	1.53
17Mm13	12.3	12.5	0.2	47.3	4.0	0
17Mm14	12.5	12.8	0.3	45.2	-2.0	0.02
17Mm15	12.8	13.9	1.1	46.5	1.3	1.04
17Mm16	13.9	14.3	0.4	43.5	-3.0	2.07
17Mm17	14.3	14.6	0.3	47.0	3.5	1.38
17Mm18	14.6	16	1.4	43.5	-3.5	1.81
17Mm19	16	19.8	3.8	38.3	-5.2	1.28
17Mm20	19.8	20	0.2	35.9	-2.4	0
17Mm21	20	20.3	0.3	37.6	1.6	0.49
17Mm22	20.3	20.7	0.4	36.1	-1.5	1.15
17Mm23	20.7	21.1	0.4	38.3	2.1	1.08
17Mm24	21.1	21.4	0.3	42.8	4.5	1.14
17Mm25	21.4	23.6	2.2	39.1	-3.7	1.01
17Mm26	23.6	26.8	3.2	49.1	10.0	2.78
17Mm27	26.8	27	0.2	44.5	-4.6	0
17Mm28	27	27.5	0.5	51.6	7.0	1.59
17Mm29	27.5	27.7	0.2	53.4	1.8	0
17Mm30	27.7	30.1	2.4	49.4	-4.0	2.18
17Mm31	30.1	31	0.9	44.9	-4.5	1.8
17Mm32	31	32.3	1.3	48.9	4.0	1.18
17Mm33	32.3	33.3	1	44.2	-4.8	2
17Mm34	33.3	33.5	0.2	39.9	-4.3	0
17Mm35	33.5	34	0.5	44.3	4.4	1.68
17Mm36	34	34.4	0.4	50.1	5.8	1.56
17Mm37	34.4	34.7	0.3	45.3	-4.8	0.36
17Mm38	34.7	36.4	1.7	49.2	3.9	1.99
17Mm39	36.4	37.4	1	42.7	-6.5	1.4
17Mm40	37.4	38.7	1.3	38.3	-4.4	0.86
17Mm41	38.7	40.5	1.8	37.1	-1.2	0.69

17Mm42	40.5	42.7	2.2	38.3	1.1	0.75
17Mm43	42.7	44.9	2.2	43.5	5.2	1.3
17Mm44	44.9	45.2	0.3	39.6	-3.9	0.92
17Mm45	45.2	49.4	4.2	48.1	8.6	2.07
17Mm46	49.4	50	0.6	45.2	-2.9	1.32
17Mm47	50	50.4	0.4	47.0	1.7	1.17
17Mm48	50.4	50.9	0.5	42.5	-4.4	1.38
17Mm49	50.9	51.1	0.2	39.2	-3.3	0
17Mm50	51.1	51.3	0.2	35.9	-3.3	0
17Mm51	51.3	52.3	1	41.3	5.3	0.68
17Mm52	52.3	53.5	1.2	39.5	-1.8	0.66
17Mm53	53.5	54.2	0.7	41.7	2.3	0.85
17Mm54	54.2	56.1	1.9	38.9	-2.9	0.86
17Mm55	56.1	57.6	1.5	50.1	11.3	2.98
17Mm56	57.6	59.4	1.8	39.1	-11.0	0.91
17Mm57	59.4	62.6	3.2	36.4	-2.7	0.53
17Mm58	62.6	62.9	0.3	39.5	3.0	1.06
17Mm59	62.9	63.6	0.7	41.9	2.4	0.55
17Mm60	63.6	64	0.4	40.8	-1.1	0.45
17Mm61	64	64.2	0.2	43.0	2.2	0
17Mm62	64.2	64.4	0.2	39.7	-3.3	0
17Mm63	64.4	65.2	0.8	42.1	2.5	1.24
17Mm64	65.2	65.7	0.5	40.5	-1.6	0.68
17Mm65	65.7	68.7	3	44.1	3.6	1.55
17Mm66	68.7	68.9	0.2	40.3	-3.9	0
17Mm67	68.9	70	1.1	42.8	2.6	1.07
17Mm68	70	70.7	0.7	40.0	-2.8	0.53
17Mm69	70.7	74.5	3.8	43.8	3.9	1.77
17Mm70	74.5	74.7	0.2	39.7	-4.1	0
17Mm71	74.7	76	1.3	42.4	2.6	1.48
17Mm72	76	78	2	37.5	-4.9	1.37
17Mm73	78	78.6	0.6	39.2	1.7	0.89
17Mm74	78.6	81.6	3	43.0	3.8	1.25
17Mm75	81.6	83.3	1.7	38.9	-4.1	1.26
17Mm76	83.3	83.5	0.2	44.0	5.1	0
17Mm77	83.5	83.7	0.2	48.1	4.1	0
17Mm78	83.7	84.2	0.5	44.4	-3.7	1.71
17Mm79	84.2	84.6	0.4	49.5	5.1	0.92
17Mm80	84.6	85.9	1.3	43.6	-5.9	1.33
17Mm81	85.9	86.1	0.2	47.8	4.2	0
17Mm82	86.1	86.6	0.5	44.4	-3.4	2.34
17Mm83	86.6	88.3	1.7	46.9	2.5	1.73
17Mm84	88.3	89.4	1.1	43.5	-3.4	1.24
17Mm85	89.4	92	2.6	37.9	-5.6	0.99
17Mm86	92	95.3	3.3	37.5	-0.4	1.27
18Mm1	0	3	3	48.0		0
18Mm2	3	3.7	0.7	41.0		0.52
18Mm3	3.7	7.1	3.4	42.6	1.6	1.6
18Mm4	7.1	7.5	0.4	39.9	-2.7	0.42
18Mm5	7.5	7.8	0.3	41.6	1.8	0.12
18Mm6	7.8	10.8	3	40.3	-1.4	1.15
18Mm7	10.8	11.7	0.9	42.7	2.4	1.32
18Mm8	11.7	11.9	0.2	39.8	-2.9	0
18Mm9	11.9	12.4	0.5	45.8	5.9	1.77
18Mm10	12.4	15.6	3.2	42.8	-3.0	1.43
18Mm11	15.6	18.5	2.9	38.3	-4.4	1.35

18Mm12	18.5	18.9	0.4	36.0	-2.3	0.25
18Mm13	18.9	19.2	0.3	38.0	2.0	0.18
18Mm14	19.2	19.4	0.2	35.5	-2.6	0
18Mm15	19.4	20.7	1.3	38.6	3.2	1.02
18Mm16	20.7	21.5	0.8	43.1	4.5	1.51
18Mm17	21.5	23.9	2.4	39.4	-3.8	0.97
18Mm18	23.9	25.3	1.4	44.0	4.6	1.11
18Mm19	25.3	25.6	0.3	39.1	-4.9	0.65
18Mm20	25.6	26	0.4	47.2	8.1	2.82
18Mm21	26	30.4	4.4	38.2	-9.0	0.97
18Mm22	30.4	30.6	0.2	42.1	3.9	0
18Mm23	30.6	31.8	1.2	38.7	-3.4	1.03
18Mm24	31.8	32.8	1	44.3	5.6	2.58
18Mm25	32.8	33.9	1.1	40.7	-3.6	0.58
18Mm26	33.9	36	2.1	44.2	3.5	1.96
18Mm27	36	36.3	0.3	49.8	5.6	1.32
18Mm28	36.3	37.2	0.9	43.1	-6.7	2.5
18Mm29	37.2	37.8	0.6	40.1	-3.0	1.17
18Mm30	37.8	38.1	0.3	44.1	3.9	1.49
18Mm31	38.1	38.5	0.4	48.5	4.4	1.47
18Mm32	38.5	39.9	1.4	44.1	-4.4	2.03
18Mm33	39.9	42	2.1	39.2	-4.8	1.08
18Mm34	42	43	1	42.7	3.4	1.28
18Mm35	43	44.5	1.5	39.9	-2.8	1.46
18Mm36	44.5	44.9	0.4	43.2	3.4	1.4
18Mm37	44.9	46.3	1.4	39.8	-3.4	1.05
18Mm38	46.3	47.9	1.6	42.5	2.7	0.98
18Mm39	47.9	48.4	0.5	38.9	-3.7	0.6
18Mm40	48.4	48.9	0.5	36.9	-2.0	0.29
18Mm41	48.9	50.1	1.2	38.7	1.8	2.08
18Mm42	50.1	50.4	0.3	42.4	3.7	1.25
18Mm43	50.4	51.9	1.5	38.0	-4.4	0.98
18Mm44	51.9	52.2	0.3	36.7	-1.3	0.15
18Mm45	52.2	52.9	0.7	39.6	2.9	1.09
18Mm46	52.9	54	1.1	42.7	3.1	1.1
18Mm47	54	54.7	0.7	40.5	-2.2	0.72
18Mm48	54.7	55.4	0.7	41.7	1.2	0.61
18Mm49	55.4	56.5	1.1	40.0	-1.7	0.85
18Mm50	56.5	58.4	1.9	42.7	2.6	1.43
18Mm51	58.4	60.6	2.2	39.5	-3.2	0.85
18Mm52	60.6	62	1.4	48.0	8.5	2.52
18Mm53	62	63.9	1.9	42.7	-5.3	1.61
18Mm54	63.9	64.1	0.2	39.8	-3.0	0
18Mm55	64.1	66.8	2.7	44.1	4.3	1.38
18Mm56	66.8	67.1	0.3	40.3	-3.8	0.47
18Mm57	67.1	69.1	2	43.2	3.0	1.61
18Mm58	69.1	69.3	0.2	40.6	-2.6	0
18Mm59	69.3	70.1	0.8	42.1	1.5	0.67
18Mm60	70.1	71.5	1.4	39.8	-2.3	1.24
18Mm61	71.5	72	0.5	36.4	-3.4	0.61
18Mm62	72	73.2	1.2	38.1	1.7	0.92
18Mm63	73.2	74	0.8	42.7	4.5	1.2
18Mm64	74	74.2	0.2	46.6	4.0	0
18Mm65	74.2	75.4	1.2	45.5	-1.1	1.15
18Mm66	75.4	77	1.6	47.5	2.0	1.79
18Mm67	77	77.4	0.4	43.1	-4.3	3.23

18Mm68	77.4	77.8	0.4	47.6	4.4	1.02
18Mm69	77.8	78.2	0.4	44.4	-3.1	1.1
18Mm70	78.2	78.4	0.2	46.2	1.7	0
18Mm71	78.4	79.3	0.9	42.4	-3.8	1.41
18Mm72	79.3	80.2	0.9	39.6	-2.8	0.96
18Mm73	80.2	80.9	0.7	48.2	8.6	1.3
18Mm74	80.9	82.6	1.7	44.2	-4.1	1.29
18Mm75	82.6	83.2	0.6	47.4	3.2	1.13
18Mm76	83.2	84.5	1.3	44.3	-3.1	1.33
18Mm77	84.5	84.7	0.2	39.7	-4.5	0
18Mm78	84.7	85.2	0.5	43.3	3.5	1.64
18Mm79	85.2	87.1	1.9	38.4	-4.9	1.32
18Mm80	87.1	87.3	0.2	36.4	-1.9	0
18Mm81	87.3	89.6	2.3	37.4	0.9	0.89
18Mm82	89.6	89.9	0.3	36.4	-0.9	0.42
18Mm83	89.9	90.8	0.9	38.4	2.0	0.79
19Mm1	0	3	3	44.0		0
19Mm2	3	3.3	0.3	42.5		2.28
19Mm3	3.3	3.7	0.4	47.8	5.3	2.78
19Mm4	3.7	3.9	0.2	45.5	-2.2	0
19Mm5	3.9	7.5	3.6	49.4	3.9	2.71
19Mm6	7.5	8.6	1.1	40.8	-8.6	1.39
19Mm7	8.6	9.2	0.6	46.1	5.2	1.38
19Mm8	9.2	9.9	0.7	40.0	-6.0	0.48
19Mm9	9.9	11.1	1.2	48.0	8.0	2.48
19Mm10	11.1	11.8	0.7	40.1	-7.9	0.6
19Mm11	11.8	12.1	0.3	42.8	2.6	1.36
19Mm12	12.1	15.9	3.8	39.4	-3.4	1.47
19Mm13	15.9	16.7	0.8	42.8	3.4	1.12
19Mm14	16.7	16.9	0.2	38.1	-4.8	0
19Mm15	16.9	17.7	0.8	42.5	4.4	0.71
19Mm16	17.7	18.6	0.9	40.1	-2.4	0.49
19Mm17	18.6	19	0.4	42.0	1.9	0.81
19Mm18	19	19.7	0.7	38.2	-3.8	0.99
19Mm19	19.7	19.9	0.2	36.3	-1.9	0
19Mm20	19.9	21.1	1.2	38.4	2.1	0.98
19Mm21	21.1	22.1	1	41.8	3.4	0.74
19Mm22	22.1	22.9	0.8	40.1	-1.7	0.67
19Mm23	22.9	26.1	3.2	43.6	3.5	1.86
19Mm24	26.1	26.5	0.4	40.5	-3.1	0.48
19Mm25	26.5	27.9	1.4	42.4	2.0	1.08
19Mm26	27.9	28.2	0.3	38.3	-4.1	1.85
19Mm27	28.2	30.3	2.1	42.6	4.3	1.73
19Mm28	30.3	32.2	1.9	39.8	-2.8	0.94
19Mm29	32.2	33.2	1	42.8	3.0	1.28
19Mm30	33.2	34.2	1	38.4	-4.4	0.61
19Mm31	34.2	35	0.8	41.6	3.2	1
19Mm32	35	36.1	1.1	39.1	-2.4	1.02
19Mm33	36.1	38.9	2.8	42.9	3.8	1.45
19Mm34	38.9	40.3	1.4	39.2	-3.7	1.19
19Mm35	40.3	41.7	1.4	44.6	5.3	1.52
19Mm36	41.7	42.9	1.2	47.6	3.0	1.86
19Mm37	42.9	43.5	0.6	39.6	-8.0	1.54
19Mm38	43.5	44.1	0.6	45.9	6.3	1.34
19Mm39	44.1	44.4	0.3	44.3	-1.6	2.17
19Mm40	44.4	45.4	1	48.0	3.7	1.54

19Mm41	45.4	45.6	0.2	42.5	-5.5	0
19Mm42	45.6	46.8	1.2	47.1	4.6	2.44
19Mm43	46.8	47.2	0.4	45.0	-2.1	0.41
19Mm44	47.2	48.2	1	47.4	2.3	3.03
19Mm45	48.2	48.6	0.4	43.8	-3.6	1.29
19Mm46	48.6	52.9	4.3	38.7	-5.1	1.09
19Mm47	52.9	54.6	1.7	44.5	5.8	2.5
19Mm48	54.6	54.8	0.2	40.1	-4.4	0
19Mm49	54.8	55.6	0.8	43.6	3.4	1.8
19Mm50	55.6	55.9	0.3	47.2	3.6	0.66
19Mm51	55.9	56.3	0.4	45.8	-1.3	0.22
19Mm52	56.3	57.6	1.3	46.5	0.7	1.38
19Mm53	57.6	58.8	1.2	43.2	-3.3	1.9
19Mm54	58.8	59	0.2	47.8	4.5	0
19Mm55	59	61.4	2.4	42.8	-5.0	1.72
XMm1	0	3	3	28.0		0
XMm2	3	7.1	4.1	40.3		0.8
XMm3	7.1	7.6	0.5	47.1	6.7	0.54
XMm4	7.6	8.7	1.1	43.0	-4.0	1.47
XMm5	8.7	9.1	0.4	40.8	-2.2	1.42
XMm6	9.1	9.3	0.2	42.4	1.6	0
XMm7	9.3	10.6	1.3	39.7	-2.8	0.87
XMm8	10.6	11.3	0.7	44.3	4.7	1.72
XMm9	11.3	12.1	0.8	47.2	2.9	1.35
XMm10	12.1	12.6	0.5	42.8	-4.4	1.53
XMm11	12.6	12.8	0.2	39.7	-3.1	0
XMm12	12.8	13.1	0.3	43.9	4.2	0.18
XMm13	13.1	20.1	7	38.9	-5.0	0.95
XMm14	20.1	20.6	0.5	42.9	4.0	1.64
XMm15	20.6	33.9	13.3	39.4	-3.5	1.16
XMm16	33.9	35.9	2	42.8	3.3	1.54
XMm17	35.9	37.8	1.9	38.2	-4.6	1.36
XMm18	37.8	38.1	0.3	36.3	-2.0	0.46
XMm19	38.1	40	1.9	38.8	2.5	1.49
XMm20	40	40.9	0.9	36.4	-2.4	0.68
XMm21	40.9	41.2	0.3	37.6	1.2	0.48
XMm22	41.2	44.3	3.1	36.8	-0.8	0.71
XMm23	44.3	45.3	1	38.8	2.0	0.75
XMm24	45.3	45.9	0.6	44.8	6.1	1.35
XMm25	45.9	49.2	3.3	39.8	-5.0	1.08
XMm26	49.2	49.6	0.4	42.5	2.7	1.34
XMm27	49.6	50.2	0.6	40.3	-2.2	0.87
XMm28	50.2	51.1	0.9	42.5	2.2	1.99
XMm29	51.1	53.5	2.4	38.4	-4.1	0.71
XMm30	53.5	54.1	0.6	41.8	3.4	0.49
XMm31	54.1	60.3	6.2	39.2	-2.6	1.37
XMm32	60.3	61.3	1	36.6	-2.5	0.61
XMm33	61.3	61.5	0.2	38.5	1.9	0
XMm34	61.5	61.9	0.4	36.6	-1.8	1.08
XMm35	61.9	62.5	0.6	37.7	1.0	0.76
XMm36	62.5	62.9	0.4	36.8	-0.9	0.41
XMm37	62.9	63.4	0.5	37.6	0.8	0.85
XMm38	63.4	63.6	0.2	36.6	-1.0	0
XMm39	63.6	67.6	4	38.1	1.5	0.97
XMm40	67.6	67.8	0.2	44.6	6.5	0
XMm41	67.8	68.1	0.3	40.2	-4.4	0.5

XMm42	68.1	69	0.9	42.1	1.9	0.99
XMm43	69	70.1	1.1	39.7	-2.3	1.4
XMm44	70.1	70.7	0.6	44.3	4.6	1.98
XMm45	70.7	71.2	0.5	47.2	2.9	1.06
XMm46	71.2	72.2	1	43.8	-3.4	1.8
XMm47	72.2	72.9	0.7	39.3	-4.5	1.42
XMm48	72.9	73.1	0.2	42.4	3.1	0
XMm49	73.1	80	6.9	38.5	-3.9	1.45
XMm50	80	80.6	0.6	35.9	-2.6	0.45
XMm51	80.6	80.9	0.3	37.7	1.7	0.96
XMm52	80.9	82.1	1.2	35.5	-2.2	1.18
XMm53	82.1	84	1.9	39.0	3.5	1.14
XMm54	84	85.4	1.4	35.6	-3.4	0.78
XMm55	85.4	86.5	1.1	37.6	2.0	0.54
XMm56	86.5	87	0.5	36.8	-0.8	0.67
XMm57	87	91.1	4.1	38.3	1.5	1.39
XMm58	91.1	92.1	1	42.2	3.9	1.21
XMm59	92.1	96.1	4	38.9	-3.3	1.26
XMm60	96.1	96.8	0.7	46.9	8.0	2.54
XMm61	96.8	97.1	0.3	42.2	-4.7	1.82
XMm62	97.1	97.6	0.5	38.8	-3.4	1.31
XMm63	97.6	98	0.4	42.7	3.8	1.8
XMm64	98	98.4	0.4	39.7	-2.9	1.32
XMm65	98.4	99.5	1.1	44.2	4.5	1.55
XMm66	99.5	99.8	0.3	40.6	-3.6	0.11
XMm67	99.8	100.4	0.6	43.0	2.3	1.21
XMm68	100.4	100.6	0.2	40.5	-2.4	0
XMm69	100.6	101.2	0.6	42.1	1.6	0.86
XMm70	101.2	102.8	1.6	39.5	-2.6	0.83
XMm71	102.8	103.4	0.6	41.0	1.5	2.07
XMm72	103.4	110.4	7	37.6	-3.4	1.2
XMm73	110.4	111	0.6	35.8	-1.8	0.42
XMm74	111	111.4	0.4	38.4	2.6	1.19
XMm75	111.4	111.9	0.5	36.7	-1.7	0.2
XMm76	111.9	113	1.1	37.5	0.8	0.39
XMm77	113	116.4	3.4	36.6	-0.8	0.88
XMm78	116.4	117.3	0.9	37.1	0.4	0.7
XMm79	117.3	118	0.7	34.9	-2.2	0.92
XMm80	118	124.5	6.5	38.0	3.2	1.13
XMm81	124.5	125	0.5	36.2	-1.8	0.53
XMm82	125	126.3	1.3	37.7	1.5	0.8
XMm83	126.3	127	0.7	35.4	-2.3	0.98
XMm84	127	128.3	1.3	37.9	2.5	0.96
XMm85	128.3	129.6	1.3	36.7	-1.2	0.3
XMm86	129.6	130.7	1.1	38.9	2.2	1.45
XMm87	130.7	131.3	0.6	42.5	3.5	0.59
XMm88	131.3	131.5	0.2	39.8	-2.7	0
XMm89	131.5	131.7	0.2	41.7	1.9	0
XMm90	131.7	132.1	0.4	40.0	-1.7	0.24
XMm91	132.1	132.3	0.2	42.2	2.2	0
XMm92	132.3	133.5	1.2	40.7	-1.5	0.67
XMm93	133.5	133.7	0.2	42.3	1.6	0
XMm94	133.7	134.6	0.9	38.2	-4.1	0.95
XMm95	134.6	134.8	0.2	36.7	-1.5	0
XMm96	134.8	136.8	2	38.7	2.0	1.39
XMm97	136.8	137.7	0.9	42.7	4.0	1.75

XMm98	137.7	138.9	1.2	38.8	-3.9	1.12
XMm99	138.9	139.6	0.7	41.6	2.8	1.96
XMm100	139.6	142.5	2.9	39.1	-2.5	1.08
XMm101	142.5	143.5	1	36.4	-2.7	0.64
XMm102	143.5	147.4	3.9	40.4	4.1	1.26
XMm103	147.4	147.6	0.2	43.0	2.6	0
XMm104	147.6	148.4	0.8	39.2	-3.8	0.82
XMm105	148.4	149.5	1.1	41.9	2.7	1.04
XMm106	149.5	151.3	1.8	39.4	-2.5	1.05
XMm107	151.3	151.5	0.2	41.5	2.1	0
XMm108	151.5	152.5	1	38.5	-3.0	1.2
XMm109	152.5	152.7	0.2	36.0	-2.5	0
XMm110	152.7	154.3	1.6	38.9	2.8	1.66
XMm111	154.3	154.5	0.2	35.0	-3.9	0
XMm112	154.5	155.8	1.3	38.8	3.8	1.2
XMm113	155.8	159	3.2	40.6	1.8	1.13
XMm114	159	159.6	0.6	42.3	1.7	0.93
XMm115	159.6	160.4	0.8	40.5	-1.8	0.45
XMm116	160.4	160.6	0.2	42.0	1.5	0
XMm117	160.6	161.6	1	39.8	-2.2	1.06
XMm118	161.6	161.8	0.2	36.7	-3.1	0
XMm119	161.8	162.6	0.8	38.9	2.2	0.59
XMm120	162.6	163.2	0.6	41.8	2.9	0.71
XMm121	163.2	166.4	3.2	39.2	-2.7	1.2
XMm122	166.4	166.7	0.3	49.7	10.5	2
YMm1	0	3	3	39.5		1.57
YMm2	0.6	0.8	0.2	36.7		0
YMm3	0.8	3	2.2	39.1	2.4	1.79
YMm4	3	16	13	0.0		0
