# Supplementary material for: The evolution of isochore patterns in vertebrate genomes
Source: BMC Genomics. 2009 Apr 3;10:146. doi: 10.1186/1471-2164-10-146 (PMC2678159; doi:10.1186/1471-2164-10-146)
Supplement: Additional File 7 — Overview of opossum chromosomes. The color-coded maps show the compositional patterns of the opossum chromosomes. [file 1471-2164-10-146-S7.pdf]

**Additional Table T4**. Coordinates, sizes, GC levels and GC standard deviations (SD) of the opossum isochores. ΔGC indicates the difference in GC between subsequent isoch

| Isochore | Start | End | Length, Mb | GC, % | Δ GC | SD (w=100kb) |
|---|---|---|---|---|---|---|
| 1_1md1 | 0 | 0.2 | 0.2 | 48.2 | | 0 |
| 1_1md2 | 0.2 | 0.7 | 0.5 | 44.0 | -4.3 | 1.33 |
| 1_1md3 | 0.7 | 1.2 | 0.5 | 40.0 | -4.0 | 1.36 |
| 1_1md4 | 1.2 | 1.6 | 0.4 | 43.0 | 3.0 | 0.52 |
| 1_1md5 | 1.6 | 1.8 | 0.2 | 40.1 | -2.9 | 0 |
| 1_1md6 | 1.8 | 2.3 | 0.5 | 44.0 | 3.9 | 0.95 |
| 1_1md7 | 2.3 | 2.5 | 0.2 | 48.3 | 4.3 | 0 |
| 1_1md8 | 2.5 | 2.8 | 0.3 | 55.5 | 7.2 | 2.76 |
| 1_1md9 | 2.8 | 3.1 | 0.3 | 50.2 | -5.3 | 1.29 |
| 1_1md10 | 3.1 | 3.4 | 0.3 | 41.9 | -8.3 | 0.79 |
| 1_1md11 | 3.4 | 3.9 | 0.5 | 51.4 | 9.5 | 4.01 |
| 1_1md12 | 3.9 | 5.5 | 1.6 | 43.4 | -8.0 | 1.67 |
| 1_1md13 | 5.5 | 5.9 | 0.4 | 47.3 | 3.8 | 1.55 |
| 1_1md14 | 5.9 | 7.4 | 1.5 | 43.0 | -4.3 | 1.98 |
| 1_1md15 | 7.4 | 7.6 | 0.2 | 48.0 | 5.1 | 0 |
| 1_1md16 | 7.6 | 7.8 | 0.2 | 43.6 | -4.4 | 0 |
| 1_1md17 | 7.8 | 8.1 | 0.3 | 38.1 | -5.5 | 0.83 |
| 1_1md18 | 8.1 | 9.9 | 1.8 | 42.8 | 4.7 | 1.85 |
| 1_1md19 | 9.9 | 10.3 | 0.4 | 37.9 | -4.9 | 1.45 |
| 1_1md20 | 10.3 | 10.8 | 0.5 | 41.7 | 3.8 | 3.29 |
| 1_1md21 | 10.8 | 11.7 | 0.9 | 39.5 | -2.2 | 1.5 |
| 1_1md22 | 11.7 | 12.9 | 1.2 | 36.0 | -3.5 | 0.57 |
| 1_1md23 | 12.9 | 15.3 | 2.4 | 38.5 | 2.6 | 1.14 |
| 1_1md24 | 15.3 | 15.5 | 0.2 | 44.0 | 5.5 | 0 |
| 1_1md25 | 15.5 | 16.5 | 1 | 39.3 | -4.7 | 0.88 |
| 1_1md26 | 16.5 | 17.4 | 0.9 | 35.8 | -3.4 | 0.56 |
| 1_1md27 | 17.4 | 17.9 | 0.5 | 38.5 | 2.6 | 1.59 |
| 1_1md28 | 17.9 | 18.2 | 0.3 | 36.6 | -1.9 | 0.18 |
| 1_1md29 | 18.2 | 20.1 | 1.9 | 38.3 | 1.7 | 1.52 |
| 1_1md30 | 20.1 | 20.4 | 0.3 | 42.9 | 4.6 | 0.57 |
| 1_1md31 | 20.4 | 20.7 | 0.3 | 38.3 | -4.6 | 0.35 |
| 1_1md32 | 20.7 | 20.9 | 0.2 | 41.8 | 3.5 | 0 |
| 1_1md33 | 20.9 | 21.7 | 0.8 | 40.1 | -1.7 | 0.96 |
| 1_1md34 | 21.7 | 22.5 | 0.8 | 41.6 | 1.5 | 0.84 |
| 1_1md35 | 22.5 | 22.7 | 0.2 | 40.2 | -1.3 | 0 |
| 1_1md36 | 22.7 | 23.2 | 0.5 | 43.1 | 2.8 | 1.1 |
| 1_1md37 | 23.2 | 23.4 | 0.2 | 49.5 | 6.5 | 0 |
| 1_1md38 | 23.4 | 24.1 | 0.7 | 39.6 | -9.9 | 1.59 |
| 1_1md39 | 24.1 | 24.3 | 0.2 | 44.8 | 5.1 | 0 |
| 1_1md40 | 24.3 | 24.5 | 0.2 | 40.4 | -4.4 | 0 |
| 1_1md41 | 24.5 | 26.3 | 1.8 | 44.3 | 3.8 | 3.76 |
| 1_1md42 | 26.3 | 26.5 | 0.2 | 48.5 | 4.2 | 0 |
| 1_1md43 | 26.5 | 26.8 | 0.3 | 45.4 | -3.0 | 0.32 |
| 1_1md44 | 26.8 | 27.2 | 0.4 | 47.4 | 2.0 | 3.15 |
| 1_1md45 | 27.2 | 28.2 | 1 | 45.3 | -2.1 | 3.37 |
| 1_1md46 | 28.2 | 28.5 | 0.3 | 39.3 | -6.1 | 1.22 |
| 1_1md47 | 28.5 | 28.7 | 0.2 | 42.7 | 3.5 | 0 |
| 1_1md48 | 28.7 | 30 | 1.3 | 38.6 | -4.1 | 1.19 |
| 1_1md49 | 30 | 31.9 | 1.9 | 35.3 | -3.4 | 1.14 |
| 1_1md50 | 31.9 | 32.1 | 0.2 | 37.9 | 2.6 | 0 |

| | | | | | | |
|---|---|---|---|---|---|---|
| 1_1md51 | 32.1 | 33.5 | 1.4 | 35.4 | -2.5 | 0.87 |
| 1_1md52 | 33.5 | 34 | 0.5 | 38.5 | 3.1 | 0.83 |
| 1_1md53 | 34 | 34.2 | 0.2 | 43.6 | 5.1 | 0 |
| 1_1md54 | 34.2 | 34.4 | 0.2 | 40.4 | -3.2 | 0 |
| 1_1md55 | 34.4 | 35.1 | 0.7 | 43.3 | 2.9 | 1.34 |
| 1_1md56 | 35.1 | 36.8 | 1.7 | 40.0 | -3.3 | 0.93 |
| 1_1md57 | 36.8 | 37.1 | 0.3 | 41.5 | 1.5 | 0.55 |
| 1_1md58 | 37.1 | 37.6 | 0.5 | 40.2 | -1.3 | 0.63 |
| 1_1md59 | 37.6 | 37.9 | 0.3 | 42.4 | 2.3 | 0.77 |
| 1_1md60 | 37.9 | 38.1 | 0.2 | 56.3 | 13.9 | 0 |
| 1_1md61 | 38.1 | 38.6 | 0.5 | 44.2 | -12.2 | 3.04 |
| 1_1md62 | 38.6 | 39.1 | 0.5 | 39.6 | -4.6 | 1.27 |
| 1_1md63 | 39.1 | 39.3 | 0.2 | 43.4 | 3.8 | 0 |
| 1_1md64 | 39.3 | 39.8 | 0.5 | 48.8 | 5.3 | 3.86 |
| 1_1md65 | 39.8 | 40 | 0.2 | 43.3 | -5.5 | 0 |
| 1_1md66 | 40 | 40.2 | 0.2 | 49.3 | 6.0 | 0 |
| 1_1md67 | 40.2 | 40.8 | 0.6 | 42.8 | -6.5 | 2.29 |
| 1_1md68 | 40.8 | 41.9 | 1.1 | 39.1 | -3.7 | 1.23 |
| 1_1md69 | 41.9 | 42.3 | 0.4 | 42.3 | 3.2 | 0.96 |
| 1_1md70 | 42.3 | 46.4 | 4.1 | 35.4 | -6.9 | 1.13 |
| 1_1md71 | 46.4 | 47.3 | 0.9 | 39.6 | 4.2 | 1.29 |
| 1_1md72 | 47.3 | 47.5 | 0.2 | 43.7 | 4.2 | 0 |
| 1_1md73 | 47.5 | 47.9 | 0.4 | 46.0 | 2.2 | 2.61 |
| 1_1md74 | 47.9 | 48.2 | 0.3 | 40.5 | -5.5 | 0.33 |
| 1_1md75 | 48.2 | 48.8 | 0.6 | 42.3 | 1.8 | 1.94 |
| 1_1md76 | 48.8 | 49 | 0.2 | 39.9 | -2.4 | 0 |
| 1_1md77 | 49 | 49.2 | 0.2 | 45.2 | 5.3 | 0 |
| 1_1md78 | 49.2 | 50.4 | 1.2 | 38.5 | -6.7 | 1.38 |
| 1_1md79 | 50.4 | 50.7 | 0.3 | 44.4 | 5.9 | 4.98 |
| 1_1md80 | 50.7 | 51 | 0.3 | 38.7 | -5.8 | 0.7 |
| 1_1md81 | 51 | 51.5 | 0.5 | 35.6 | -3.0 | 0.98 |
| 1_1md82 | 51.5 | 54.8 | 3.3 | 38.2 | 2.6 | 1.28 |
| 1_1md83 | 54.8 | 55 | 0.2 | 41.3 | 3.0 | 0 |
| 1_1md84 | 55 | 55.3 | 0.3 | 40.6 | -0.6 | 0.45 |
| 1_1md85 | 55.3 | 57.1 | 1.8 | 39.7 | -1.0 | 0.75 |
| 1_1md86 | 57.1 | 58.1 | 1 | 42.8 | 3.1 | 0.9 |
| 1_1md87 | 58.1 | 61 | 2.9 | 38.5 | -4.3 | 1.02 |
| 1_1md88 | 61 | 65.2 | 4.2 | 34.8 | -3.7 | 1.07 |
| 1_1md89 | 65.2 | 65.9 | 0.7 | 39.5 | 4.7 | 1.3 |
| 1_1md90 | 65.9 | 66.2 | 0.3 | 35.4 | -4.1 | 1.23 |
| 1_1md91 | 66.2 | 67.6 | 1.4 | 38.7 | 3.3 | 1.31 |
| 1_1md92 | 67.6 | 68.1 | 0.5 | 36.4 | -2.3 | 0.38 |
| 1_1md93 | 68.1 | 68.6 | 0.5 | 38.5 | 2.1 | 1.01 |
| 1_1md94 | 68.6 | 68.8 | 0.2 | 35.5 | -3.1 | 0 |
| 1_1md95 | 68.8 | 69 | 0.2 | 38.2 | 2.8 | 0 |
| 1_1md96 | 69 | 69.2 | 0.2 | 36.7 | -1.5 | 0 |
| 1_1md97 | 69.2 | 69.6 | 0.4 | 37.9 | 1.2 | 0.9 |
| 1_1md98 | 69.6 | 69.8 | 0.2 | 36.0 | -1.9 | 0 |
| 1_1md99 | 69.8 | 70.2 | 0.4 | 38.3 | 2.2 | 2.87 |
| 1_1md100 | 70.2 | 72.2 | 2 | 39.0 | 0.7 | 1.31 |
| 1_1md101 | 72.2 | 72.4 | 0.2 | 35.8 | -3.2 | 0 |
| 1_1md102 | 72.4 | 73.1 | 0.7 | 39.3 | 3.4 | 2.31 |
| 1_1md103 | 73.1 | 73.9 | 0.8 | 42.1 | 2.8 | 0.83 |
| 1_1md104 | 73.9 | 75.3 | 1.4 | 38.3 | -3.7 | 1.84 |
| 1_1md105 | 75.3 | 75.7 | 0.4 | 36.3 | -2.0 | 0.75 |
| 1_1md106 | 75.7 | 76.5 | 0.8 | 38.4 | 2.1 | 0.55 |

| | | | | | | |
|---|---|---|---|---|---|---|
| 1_1md107 | 76.5 | 76.9 | 0.4 | 36.0 | -2.5 | 0.26 |
| 1_1md108 | 76.9 | 78.3 | 1.4 | 38.0 | 2.0 | 1.14 |
| 1_1md109 | 78.3 | 79.1 | 0.8 | 36.3 | -1.7 | 1.28 |
| 1_1md110 | 79.1 | 79.4 | 0.3 | 37.1 | 0.8 | 1.68 |
| 1_1md111 | 79.4 | 79.6 | 0.2 | 36.5 | -0.6 | 0 |
| 1_1md112 | 79.6 | 79.8 | 0.2 | 38.4 | 1.9 | 0 |
| 1_1md113 | 79.8 | 80.3 | 0.5 | 36.5 | -1.9 | 0.98 |
| 1_1md114 | 80.3 | 80.6 | 0.3 | 37.8 | 1.3 | 0.52 |
| 1_1md115 | 80.6 | 81.8 | 1.2 | 36.3 | -1.5 | 0.79 |
| 1_1md116 | 81.8 | 82.3 | 0.5 | 37.2 | 0.9 | 0.42 |
| 1_1md117 | 82.3 | 82.6 | 0.3 | 36.8 | -0.4 | 0.25 |
| 1_1md118 | 82.6 | 83.6 | 1 | 36.1 | -0.8 | 0.53 |
| 1_1md119 | 83.6 | 84 | 0.4 | 37.3 | 1.3 | 0.45 |
| 1_1md120 | 84 | 85.6 | 1.6 | 36.3 | -1.1 | 0.78 |
| 1_1md121 | 85.6 | 86.1 | 0.5 | 37.3 | 1.0 | 0.3 |
| 1_1md122 | 86.1 | 86.5 | 0.4 | 35.9 | -1.3 | 0.36 |
| 1_1md123 | 86.5 | 86.9 | 0.4 | 37.6 | 1.6 | 1.37 |
| 1_1md124 | 86.9 | 89.2 | 2.3 | 36.2 | -1.4 | 0.77 |
| 1_1md125 | 89.2 | 90.9 | 1.7 | 38.1 | 1.9 | 1.15 |
| 1_1md126 | 90.9 | 91.9 | 1 | 36.2 | -1.9 | 0.98 |
| 1_1md127 | 91.9 | 92.5 | 0.6 | 38.5 | 2.3 | 1.04 |
| 1_1md128 | 92.5 | 92.7 | 0.2 | 36.3 | -2.2 | 0 |
| 1_1md129 | 92.7 | 93.1 | 0.4 | 37.9 | 1.6 | 0.9 |
| 1_1md130 | 93.1 | 93.3 | 0.2 | 36.4 | -1.4 | 0 |
| 1_1md131 | 93.3 | 93.5 | 0.2 | 37.6 | 1.2 | 0 |
| 1_1md132 | 93.5 | 93.9 | 0.4 | 35.4 | -2.2 | 0.94 |
| 1_1md133 | 93.9 | 94.1 | 0.2 | 37.3 | 1.8 | 0 |
| 1_1md134 | 94.1 | 96.3 | 2.2 | 35.3 | -2.0 | 1.32 |
| 1_1md135 | 96.3 | 96.5 | 0.2 | 38.3 | 3.0 | 0 |
| 1_1md136 | 96.5 | 97.2 | 0.7 | 36.0 | -2.3 | 0.87 |
| 1_1md137 | 97.2 | 98.4 | 1.2 | 38.2 | 2.2 | 0.95 |
| 1_1md138 | 98.4 | 100.4 | 2 | 35.9 | -2.3 | 0.96 |
| 1_1md139 | 100.4 | 100.7 | 0.3 | 37.9 | 2.0 | 0.52 |
| 1_1md140 | 100.7 | 100.9 | 0.2 | 35.2 | -2.7 | 0 |
| 1_1md141 | 100.9 | 102 | 1.1 | 38.0 | 2.8 | 1.65 |
| 1_1md142 | 102 | 102.2 | 0.2 | 35.6 | -2.4 | 0 |
| 1_1md143 | 102.2 | 103 | 0.8 | 37.7 | 2.0 | 0.51 |
| 1_1md144 | 103 | 106 | 3 | 36.0 | -1.7 | 0.72 |
| 1_1md145 | 106 | 107.6 | 1.6 | 37.8 | 1.8 | 0.99 |
| 1_1md146 | 107.6 | 107.8 | 0.2 | 35.7 | -2.1 | 0 |
| 1_1md147 | 107.8 | 108.3 | 0.5 | 38.9 | 3.2 | 2.2 |
| 1_1md148 | 108.3 | 108.6 | 0.3 | 43.2 | 4.3 | 0.95 |
| 1_1md149 | 108.6 | 109.1 | 0.5 | 38.8 | -4.4 | 1.85 |
| 1_1md150 | 109.1 | 109.4 | 0.3 | 42.0 | 3.2 | 2.53 |
| 1_1md151 | 109.4 | 110.3 | 0.9 | 40.2 | -1.8 | 3.11 |
| 1_1md152 | 110.3 | 110.7 | 0.4 | 41.9 | 1.7 | 2.71 |
| 1_1md153 | 110.7 | 111 | 0.3 | 38.2 | -3.7 | 2.57 |
| 1_1md154 | 111 | 112.2 | 1.2 | 42.7 | 4.4 | 1.43 |
| 1_1md155 | 112.2 | 113.1 | 0.9 | 39.0 | -3.6 | 1.28 |
| 1_1md156 | 113.1 | 113.9 | 0.8 | 37.0 | -2.1 | 0.75 |
| 1_1md157 | 113.9 | 114.3 | 0.4 | 37.4 | 0.5 | 0.28 |
| 1_1md158 | 114.3 | 115.1 | 0.8 | 36.2 | -1.3 | 0.39 |
| 1_1md159 | 115.1 | 115.4 | 0.3 | 38.9 | 2.7 | 1.05 |
| 1_1md160 | 115.4 | 117.1 | 1.7 | 36.0 | -2.9 | 0.7 |
| 1_1md161 | 117.1 | 117.3 | 0.2 | 37.6 | 1.7 | 0 |
| 1_1md162 | 117.3 | 117.5 | 0.2 | 36.7 | -1.0 | 0 |

| | | | | | | |
|---|---|---|---|---|---|---|
| 1_1md163 | 117.5 | 118.3 | 0.8 | 37.6 | 0.9 | 0.76 |
| 1_1md164 | 118.3 | 118.6 | 0.3 | 36.3 | -1.3 | 0.39 |
| 1_1md165 | 118.6 | 118.8 | 0.2 | 37.3 | 1.0 | 0 |
| 1_1md166 | 118.8 | 119 | 0.2 | 35.6 | -1.7 | 0 |
| 1_1md167 | 119 | 119.2 | 0.2 | 37.3 | 1.7 | 0 |
| 1_1md168 | 119.2 | 120.4 | 1.2 | 36.1 | -1.2 | 0.68 |
| 1_1md169 | 120.4 | 120.6 | 0.2 | 37.5 | 1.4 | 0 |
| 1_1md170 | 120.6 | 120.9 | 0.3 | 36.7 | -0.8 | 0.27 |
| 1_1md171 | 120.9 | 121.4 | 0.5 | 38.2 | 1.5 | 0.31 |
| 1_1md172 | 121.4 | 121.9 | 0.5 | 43.3 | 5.1 | 0.69 |
| 1_1md173 | 121.9 | 122.5 | 0.6 | 39.4 | -3.9 | 1.44 |
| 1_1md174 | 122.5 | 122.7 | 0.2 | 41.5 | 2.1 | 0 |
| 1_1md175 | 122.7 | 124.4 | 1.7 | 39.1 | -2.4 | 1.34 |
| 1_1md176 | 124.4 | 127 | 2.6 | 35.6 | -3.5 | 0.81 |
| 1_1md177 | 127 | 127.3 | 0.3 | 38.7 | 3.1 | 2.35 |
| 1_1md178 | 127.3 | 129 | 1.7 | 36.3 | -2.4 | 0.67 |
| 1_1md179 | 129 | 129.7 | 0.7 | 38.2 | 1.8 | 1.21 |
| 1_1md180 | 129.7 | 129.9 | 0.2 | 34.9 | -3.3 | 0 |
| 1_1md181 | 129.9 | 130.2 | 0.3 | 38.2 | 3.3 | 1.32 |
| 1_1md182 | 130.2 | 139.6 | 9.4 | 35.2 | -2.9 | 1.09 |
| 1_1md183 | 139.6 | 139.8 | 0.2 | 38.4 | 3.2 | 0 |
| 1_1md184 | 139.8 | 142.6 | 2.8 | 35.9 | -2.5 | 1.07 |
| 1_1md185 | 142.6 | 143.2 | 0.6 | 38.5 | 2.6 | 0.82 |
| 1_1md186 | 143.2 | 143.5 | 0.3 | 35.9 | -2.6 | 0.18 |
| 1_1md187 | 143.5 | 143.9 | 0.4 | 37.8 | 1.9 | 0.51 |
| 1_1md188 | 143.9 | 144.1 | 0.2 | 36.0 | -1.9 | 0 |
| 1_1md189 | 144.1 | 144.6 | 0.5 | 37.4 | 1.5 | 0.38 |
| 1_1md190 | 144.6 | 145.3 | 0.7 | 36.2 | -1.3 | 1.11 |
| 1_1md191 | 145.3 | 145.6 | 0.3 | 37.8 | 1.7 | 0.52 |
| 1_1md192 | 145.6 | 147.4 | 1.8 | 36.1 | -1.7 | 1.07 |
| 1_1md193 | 147.4 | 147.6 | 0.2 | 38.2 | 2.1 | 0 |
| 1_1md194 | 147.6 | 147.8 | 0.2 | 36.4 | -1.9 | 0 |
| 1_1md195 | 147.8 | 149.8 | 2 | 38.1 | 1.8 | 1.92 |
| 1_1md196 | 149.8 | 150.1 | 0.3 | 35.8 | -2.3 | 0.94 |
| 1_1md197 | 150.1 | 150.4 | 0.3 | 38.6 | 2.8 | 2.05 |
| 1_1md198 | 150.4 | 150.7 | 0.3 | 43.1 | 4.5 | 1.31 |
| 1_1md199 | 150.7 | 151.1 | 0.4 | 40.1 | -2.9 | 1.38 |
| 1_1md200 | 151.1 | 151.7 | 0.6 | 39.6 | -0.6 | 2.81 |
| 1_1md201 | 151.7 | 151.9 | 0.2 | 35.8 | -3.8 | 0 |
| 1_1md202 | 151.9 | 152.3 | 0.4 | 41.0 | 5.1 | 1.41 |
| 1_1md203 | 152.3 | 154 | 1.7 | 40.2 | -0.8 | 1.61 |
| 1_1md204 | 154 | 154.2 | 0.2 | 36.5 | -3.6 | 0 |
| 1_1md205 | 154.2 | 154.4 | 0.2 | 37.8 | 1.3 | 0 |
| 1_1md206 | 154.4 | 154.6 | 0.2 | 35.1 | -2.7 | 0 |
| 1_1md207 | 154.6 | 155.2 | 0.6 | 38.9 | 3.8 | 3.18 |
| 1_1md208 | 155.2 | 155.4 | 0.2 | 41.2 | 2.3 | 0 |
| 1_1md209 | 155.4 | 159 | 3.6 | 39.4 | -1.8 | 1.29 |
| 1_1md210 | 159 | 161 | 2 | 36.3 | -3.1 | 1.05 |
| 1_1md211 | 161 | 161.2 | 0.2 | 37.5 | 1.2 | 0 |
| 1_1md212 | 161.2 | 161.4 | 0.2 | 36.3 | -1.2 | 0 |
| 1_1md213 | 161.4 | 161.6 | 0.2 | 37.5 | 1.2 | 0 |
| 1_1md214 | 161.6 | 162.3 | 0.7 | 35.6 | -1.9 | 1.14 |
| 1_1md215 | 162.3 | 164.1 | 1.8 | 37.7 | 2.1 | 0.91 |
| 1_1md216 | 164.1 | 168.4 | 4.3 | 35.9 | -1.9 | 0.97 |
| 1_1md217 | 168.4 | 170.2 | 1.8 | 38.8 | 2.9 | 1.67 |
| 1_1md218 | 170.2 | 171.1 | 0.9 | 36.5 | -2.3 | 0.63 |

| | | | | | | |
|---|---|---|---|---|---|---|
| 1_1md219 | 171.1 | 172 | 0.9 | 38.0 | 1.5 | 0.52 |
| 1_1md220 | 172 | 172.4 | 0.4 | 42.7 | 4.7 | 0.78 |
| 1_1md221 | 172.4 | 172.7 | 0.3 | 39.7 | -3.1 | 0.82 |
| 1_1md222 | 172.7 | 173 | 0.3 | 44.4 | 4.8 | 2.77 |
| 1_1md223 | 173 | 173.3 | 0.3 | 38.3 | -6.1 | 0.45 |
| 1_1md224 | 173.3 | 173.9 | 0.6 | 43.4 | 5.1 | 1.72 |
| 1_1md225 | 173.9 | 174.7 | 0.8 | 37.9 | -5.5 | 1.23 |
| 1_1md226 | 174.7 | 176.1 | 1.4 | 35.7 | -2.1 | 0.6 |
| 1_1md227 | 176.1 | 177.2 | 1.1 | 37.4 | 1.7 | 0.32 |
| 1_1md228 | 177.2 | 177.9 | 0.7 | 36.3 | -1.1 | 0.59 |
| 1_1md229 | 177.9 | 178.1 | 0.2 | 37.3 | 1.0 | 0 |
| 1_1md230 | 178.1 | 178.4 | 0.3 | 35.9 | -1.4 | 0.36 |
| 1_1md231 | 178.4 | 178.6 | 0.2 | 37.7 | 1.8 | 0 |
| 1_1md232 | 178.6 | 179.2 | 0.6 | 36.4 | -1.3 | 0.76 |
| 1_1md233 | 179.2 | 179.4 | 0.2 | 39.8 | 3.5 | 0 |
| 1_1md234 | 179.4 | 180.2 | 0.8 | 36.2 | -3.6 | 0.79 |
| 1_1md235 | 180.2 | 180.4 | 0.2 | 38.0 | 1.7 | 0 |
| 1_1md236 | 180.4 | 180.7 | 0.3 | 36.1 | -1.8 | 0.26 |
| 1_1md237 | 180.7 | 181 | 0.3 | 37.8 | 1.7 | 0.5 |
| 1_1md238 | 181 | 181.7 | 0.7 | 36.2 | -1.6 | 0.96 |
| 1_1md239 | 181.7 | 182.3 | 0.6 | 37.6 | 1.4 | 0.67 |
| 1_1md240 | 182.3 | 182.7 | 0.4 | 36.1 | -1.5 | 1.3 |
| 1_1md241 | 182.7 | 183.1 | 0.4 | 37.3 | 1.3 | 0.72 |
| 1_1md242 | 183.1 | 183.7 | 0.6 | 35.9 | -1.4 | 0.63 |
| 1_1md243 | 183.7 | 183.9 | 0.2 | 37.9 | 2.0 | 0 |
| 1_1md244 | 183.9 | 189 | 5.1 | 35.8 | -2.1 | 0.95 |
| 1_1md245 | 189 | 189.9 | 0.9 | 37.5 | 1.7 | 1.15 |
| 1_1md246 | 189.9 | 193.4 | 3.5 | 35.4 | -2.1 | 0.71 |
| 1_1md247 | 193.4 | 193.8 | 0.4 | 37.6 | 2.2 | 0.85 |
| 1_1md248 | 193.8 | 194 | 0.2 | 35.5 | -2.1 | 0 |
| 1_1md249 | 194 | 194.8 | 0.8 | 38.2 | 2.6 | 1.04 |
| 1_1md250 | 194.8 | 195 | 0.2 | 36.0 | -2.1 | 0 |
| 1_1md251 | 195 | 195.4 | 0.4 | 37.0 | 0.9 | 0.77 |
| 1_1md252 | 195.4 | 195.8 | 0.4 | 37.8 | 0.8 | 0.33 |
| 1_1md253 | 195.8 | 196 | 0.2 | 36.3 | -1.5 | 0 |
| 1_1md254 | 196 | 196.8 | 0.8 | 38.4 | 2.0 | 1.89 |
| 1_1md255 | 196.8 | 197.3 | 0.5 | 43.0 | 4.7 | 2.5 |
| 1_1md256 | 197.3 | 197.6 | 0.3 | 38.4 | -4.6 | 1.81 |
| 1_1md257 | 197.6 | 198 | 0.4 | 42.9 | 4.5 | 0.99 |
| 1_1md258 | 198 | 198.4 | 0.4 | 37.8 | -5.2 | 0.46 |
| 1_1md259 | 198.4 | 198.6 | 0.2 | 43.1 | 5.3 | 0 |
| 1_1md260 | 198.6 | 198.8 | 0.2 | 37.7 | -5.4 | 0 |
| 1_1md261 | 198.8 | 199 | 0.2 | 36.8 | -0.8 | 0 |
| 1_1md262 | 199 | 199.9 | 0.9 | 40.4 | 3.5 | 1.7 |
| 1_1md263 | 199.9 | 200.9 | 1 | 37.5 | -2.9 | 3.05 |
| 1_1md264 | 200.9 | 201.9 | 1 | 39.5 | 2.0 | 2.21 |
| 1_1md265 | 201.9 | 202.6 | 0.7 | 36.3 | -3.2 | 0.46 |
| 1_1md266 | 202.6 | 203 | 0.4 | 38.5 | 2.2 | 1.18 |
| 1_1md267 | 203 | 203.2 | 0.2 | 42.6 | 4.1 | 0 |
| 1_1md268 | 203.2 | 205.1 | 1.9 | 38.3 | -4.3 | 0.83 |
| 1_1md269 | 205.1 | 218.9 | 13.8 | 35.4 | -2.9 | 1.03 |
| 1_1md270 | 218.9 | 220.8 | 1.9 | 38.2 | 2.8 | 1.44 |
| 1_1md271 | 220.8 | 221.1 | 0.3 | 35.7 | -2.5 | 0.36 |
| 1_1md272 | 221.1 | 222.8 | 1.7 | 38.6 | 2.9 | 1.72 |
| 1_1md273 | 222.8 | 223.4 | 0.6 | 36.4 | -2.2 | 1.17 |
| 1_1md274 | 223.4 | 223.9 | 0.5 | 39.4 | 3.0 | 0.62 |

| | | | | | | |
|---|---|---|---|---|---|---|
| 1_1md275 | 223.9 | 224.1 | 0.2 | 36.7 | -2.7 | 0 |
| 1_1md276 | 224.1 | 225.7 | 1.6 | 38.0 | 1.3 | 0.55 |
| 1_1md277 | 225.7 | 226.3 | 0.6 | 36.3 | -1.7 | 0.71 |
| 1_1md278 | 226.3 | 227.3 | 1 | 38.3 | 2.0 | 0.94 |
| 1_1md279 | 227.3 | 243.3 | 16 | 35.2 | -3.1 | 1.15 |
| 1_1md280 | 243.3 | 244 | 0.7 | 37.9 | 2.7 | 0.91 |
| 1_1md281 | 244 | 244.2 | 0.2 | 36.4 | -1.5 | 0 |
| 1_1md282 | 244.2 | 245.6 | 1.4 | 38.7 | 2.3 | 1.34 |
| 1_1md283 | 245.6 | 245.8 | 0.2 | 41.9 | 3.1 | 0 |
| 1_1md284 | 245.8 | 246.5 | 0.7 | 39.2 | -2.6 | 1.07 |
| 1_1md285 | 246.5 | 246.7 | 0.2 | 36.4 | -2.8 | 0 |
| 1_1md286 | 246.7 | 247 | 0.3 | 39.8 | 3.4 | 0.44 |
| 1_1md287 | 247 | 247.4 | 0.4 | 35.7 | -4.1 | 0.7 |
| 1_1md288 | 247.4 | 249 | 1.6 | 39.1 | 3.3 | 1.63 |
| 1_1md289 | 249 | 249.6 | 0.6 | 40.9 | 1.9 | 1.08 |
| 1_1md290 | 249.6 | 250.7 | 1.1 | 38.1 | -2.8 | 1.44 |
| 1_1md291 | 250.7 | 250.9 | 0.2 | 36.7 | -1.4 | 0 |
| 1_1md292 | 250.9 | 252 | 1.1 | 38.2 | 1.5 | 0.9 |
| 1_1md293 | 252 | 252.7 | 0.7 | 36.5 | -1.7 | 0.39 |
| 1_1md294 | 252.7 | 253 | 0.3 | 38.1 | 1.6 | 0.59 |
| 1_1md295 | 253 | 253.3 | 0.3 | 35.4 | -2.6 | 0.53 |
| 1_1md296 | 253.3 | 253.6 | 0.3 | 39.1 | 3.7 | 0.75 |
| 1_1md297 | 253.6 | 253.9 | 0.3 | 35.0 | -4.1 | 1.57 |
| 1_1md298 | 253.9 | 254.5 | 0.6 | 37.9 | 2.9 | 0.73 |
| 1_1md299 | 254.5 | 254.7 | 0.2 | 36.6 | -1.3 | 0 |
| 1_1md300 | 254.7 | 255.8 | 1.1 | 37.7 | 1.1 | 1.5 |
| | | | | | | |
| 1_2md1 | 0 | 0.3 | 0.3 | 37.2 | | 0.44 |
| 1_2md2 | 0.3 | 0.8 | 0.5 | 36.0 | -1.2 | 0.72 |
| 1_2md3 | 0.8 | 1 | 0.2 | 37.2 | 1.2 | 0 |
| 1_2md4 | 1 | 4.3 | 3.3 | 36.0 | -1.2 | 0.86 |
| 1_2md5 | 4.3 | 4.5 | 0.2 | 37.3 | 1.3 | 0 |
| 1_2md6 | 4.5 | 11 | 6.5 | 35.8 | -1.5 | 0.9 |
| 1_2md7 | 11 | 11.2 | 0.2 | 37.3 | 1.5 | 0 |
| 1_2md8 | 11.2 | 11.4 | 0.2 | 36.9 | -0.4 | 0 |
| 1_2md9 | 11.4 | 12.7 | 1.3 | 38.4 | 1.5 | 1.24 |
| 1_2md10 | 12.7 | 13.2 | 0.5 | 35.5 | -2.9 | 0.97 |
| 1_2md11 | 13.2 | 13.7 | 0.5 | 37.3 | 1.8 | 1.04 |
| 1_2md12 | 13.7 | 15.5 | 1.8 | 35.9 | -1.4 | 0.92 |
| 1_2md13 | 15.5 | 16.5 | 1 | 38.4 | 2.5 | 1.45 |
| 1_2md14 | 16.5 | 17.2 | 0.7 | 35.7 | -2.7 | 0.52 |
| 1_2md15 | 17.2 | 20 | 2.8 | 38.1 | 2.4 | 0.82 |
| 1_2md16 | 20 | 21.2 | 1.2 | 36.1 | -2.0 | 0.54 |
| 1_2md17 | 21.2 | 21.4 | 0.2 | 37.2 | 1.1 | 0 |
| 1_2md18 | 21.4 | 22.1 | 0.7 | 36.0 | -1.2 | 1.06 |
| 1_2md19 | 22.1 | 22.3 | 0.2 | 37.8 | 1.8 | 0 |
| 1_2md20 | 22.3 | 22.5 | 0.2 | 36.1 | -1.7 | 0 |
| 1_2md21 | 22.5 | 22.7 | 0.2 | 37.2 | 1.1 | 0 |
| 1_2md22 | 22.7 | 28.6 | 5.9 | 35.6 | -1.6 | 1.17 |
| 1_2md23 | 28.6 | 29.3 | 0.7 | 37.1 | 1.5 | 0.67 |
| 1_2md24 | 29.3 | 29.9 | 0.6 | 35.9 | -1.2 | 0.41 |
| 1_2md25 | 29.9 | 30.2 | 0.3 | 38.0 | 2.1 | 0.62 |
| 1_2md26 | 30.2 | 33 | 2.8 | 35.5 | -2.5 | 0.87 |
| 1_2md27 | 33 | 36 | 3 | 38.2 | 2.7 | 0 |
| 1_2md28 | 36 | 36.2 | 0.2 | 36.4 | -1.8 | 0 |
| 1_2md29 | 36.2 | 36.4 | 0.2 | 37.6 | 1.2 | 0 |

| | | | | | | |
|---|---|---|---|---|---|---|
| 1_2md30 | 36.4 | 36.6 | 0.2 | 36.4 | -1.2 | 0 |
| 1_2md31 | 36.6 | 37.2 | 0.6 | 37.3 | 1.0 | 0.58 |
| 1_2md32 | 37.2 | 37.5 | 0.3 | 36.7 | -0.6 | 0.28 |
| 1_2md33 | 37.5 | 37.7 | 0.2 | 37.4 | 0.6 | 0 |
| 1_2md34 | 37.7 | 38.2 | 0.5 | 36.7 | -0.6 | 0.43 |
| 1_2md35 | 38.2 | 40.1 | 1.9 | 37.8 | 1.1 | 0.71 |
| 1_2md36 | 40.1 | 41.3 | 1.2 | 36.3 | -1.5 | 0.9 |
| 1_2md37 | 41.3 | 42.9 | 1.6 | 37.7 | 1.3 | 0.86 |
| 1_2md38 | 42.9 | 43.1 | 0.2 | 36.8 | -0.9 | 0 |
| 1_2md39 | 43.1 | 43.4 | 0.3 | 36.7 | -0.1 | 0.78 |
| 1_2md40 | 43.4 | 44 | 0.6 | 35.4 | -1.3 | 0.27 |
| 1_2md41 | 44 | 45.9 | 1.9 | 37.5 | 2.1 | 1.05 |
| 1_2md42 | 45.9 | 46.5 | 0.6 | 36.4 | -1.1 | 0.27 |
| 1_2md43 | 46.5 | 46.9 | 0.4 | 39.8 | 3.3 | 1.82 |
| 1_2md44 | 46.9 | 47.2 | 0.3 | 36.5 | -3.3 | 0.41 |
| 1_2md45 | 47.2 | 48.4 | 1.2 | 37.8 | 1.3 | 1 |
| 1_2md46 | 48.4 | 49.2 | 0.8 | 35.1 | -2.7 | 1.24 |
| 1_2md47 | 49.2 | 49.4 | 0.2 | 38.2 | 3.1 | 0 |
| 1_2md48 | 49.4 | 50.3 | 0.9 | 36.0 | -2.2 | 0.86 |
| 1_2md49 | 50.3 | 52.3 | 2 | 38.1 | 2.1 | 1.12 |
| 1_2md50 | 52.3 | 52.7 | 0.4 | 37.0 | -1.2 | 0.97 |
| 1_2md51 | 52.7 | 53.5 | 0.8 | 41.6 | 4.6 | 1.8 |
| 1_2md52 | 53.5 | 55.2 | 1.7 | 38.8 | -2.7 | 0.62 |
| 1_2md53 | 55.2 | 55.4 | 0.2 | 42.3 | 3.5 | 0 |
| 1_2md54 | 55.4 | 55.7 | 0.3 | 39.0 | -3.3 | 1.26 |
| 1_2md55 | 55.7 | 55.9 | 0.2 | 35.2 | -3.8 | 0 |
| 1_2md56 | 55.9 | 56.8 | 0.9 | 38.6 | 3.4 | 2.43 |
| 1_2md57 | 56.8 | 57 | 0.2 | 36.0 | -2.6 | 0 |
| 1_2md58 | 57 | 58.7 | 1.7 | 38.8 | 2.8 | 2.02 |
| 1_2md59 | 58.7 | 59.2 | 0.5 | 36.5 | -2.3 | 0.83 |
| 1_2md60 | 59.2 | 59.6 | 0.4 | 38.2 | 1.7 | 0.67 |
| 1_2md61 | 59.6 | 59.8 | 0.2 | 35.9 | -2.3 | 0 |
| 1_2md62 | 59.8 | 60.2 | 0.4 | 38.7 | 2.8 | 1.43 |
| 1_2md63 | 60.2 | 61.5 | 1.3 | 36.5 | -2.2 | 0.48 |
| 1_2md64 | 61.5 | 63.4 | 1.9 | 38.4 | 1.9 | 1.15 |
| 1_2md65 | 63.4 | 63.6 | 0.2 | 35.5 | -2.8 | 0 |
| 1_2md66 | 63.6 | 65.2 | 1.6 | 38.2 | 2.7 | 1.14 |
| 1_2md67 | 65.2 | 69.1 | 3.9 | 35.2 | -3.1 | 0.93 |
| 1_2md68 | 69.1 | 69.4 | 0.3 | 37.7 | 2.5 | 0.34 |
| 1_2md69 | 69.4 | 71 | 1.6 | 36.0 | -1.7 | 0.82 |
| 1_2md70 | 71 | 71.6 | 0.6 | 37.6 | 1.6 | 0.35 |
| 1_2md71 | 71.6 | 72.7 | 1.1 | 36.4 | -1.2 | 0.92 |
| 1_2md72 | 72.7 | 73 | 0.3 | 38.1 | 1.8 | 0.73 |
| 1_2md73 | 73 | 73.2 | 0.2 | 36.2 | -1.9 | 0 |
| 1_2md74 | 73.2 | 75.2 | 2 | 39.1 | 2.8 | 1.61 |
| 1_2md75 | 75.2 | 75.7 | 0.5 | 44.5 | 5.4 | 2.13 |
| 1_2md76 | 75.7 | 77.7 | 2 | 38.7 | -5.9 | 1.38 |
| 1_2md77 | 77.7 | 78 | 0.3 | 44.3 | 5.7 | 0.53 |
| 1_2md78 | 78 | 79.8 | 1.8 | 39.1 | -5.3 | 1.12 |
| 1_2md79 | 79.8 | 84.2 | 4.4 | 36.0 | -3.0 | 0.7 |
| 1_2md80 | 84.2 | 87.2 | 3 | 38.3 | 2.3 | 1.49 |
| 1_2md81 | 87.2 | 87.5 | 0.3 | 42.6 | 4.3 | 0.74 |
| 1_2md82 | 87.5 | 87.9 | 0.4 | 40.4 | -2.2 | 2.28 |
| 1_2md83 | 87.9 | 88.1 | 0.2 | 42.4 | 2.0 | 0 |
| 1_2md84 | 88.1 | 89.8 | 1.7 | 39.0 | -3.4 | 1.17 |
| 1_2md85 | 89.8 | 90 | 0.2 | 42.3 | 3.3 | 0 |

| | | | | | | |
|---|---|---|---|---|---|---|
| 1_2md86 | 90 | 90.7 | 0.7 | 38.7 | -3.6 | 1.12 |
| 1_2md87 | 90.7 | 90.9 | 0.2 | 36.9 | -1.8 | 0 |
| 1_2md88 | 90.9 | 91.1 | 0.2 | 37.5 | 0.6 | 0 |
| 1_2md89 | 91.1 | 91.9 | 0.8 | 35.7 | -1.8 | 0.84 |
| 1_2md90 | 91.9 | 93.9 | 2 | 38.7 | 2.9 | 1.33 |
| 1_2md91 | 93.9 | 95.8 | 1.9 | 35.7 | -2.9 | 0.84 |
| 1_2md92 | 95.8 | 98.2 | 2.4 | 37.9 | 2.1 | 1.05 |
| 1_2md93 | 98.2 | 99.1 | 0.9 | 36.7 | -1.1 | 0.43 |
| 1_2md94 | 99.1 | 100.9 | 1.8 | 38.1 | 1.4 | 0.84 |
| 1_2md95 | 100.9 | 110.3 | 9.4 | 35.0 | -3.2 | 0.92 |
| 1_2md96 | 110.3 | 111.4 | 1.1 | 39.0 | 4.0 | 1.55 |
| 1_2md97 | 111.4 | 111.6 | 0.2 | 41.5 | 2.5 | 0 |
| 1_2md98 | 111.6 | 114.4 | 2.8 | 38.2 | -3.3 | 1.13 |
| 1_2md99 | 114.4 | 114.8 | 0.4 | 35.1 | -3.2 | 1.04 |
| 1_2md100 | 114.8 | 118.4 | 3.6 | 39.2 | 4.1 | 1.13 |
| 1_2md101 | 118.4 | 118.7 | 0.3 | 36.1 | -3.1 | 0.79 |
| 1_2md102 | 118.7 | 118.9 | 0.2 | 37.2 | 1.1 | 0 |
| 1_2md103 | 118.9 | 119.6 | 0.7 | 36.7 | -0.4 | 0.38 |
| 1_2md104 | 119.6 | 120.4 | 0.8 | 37.4 | 0.7 | 0.9 |
| 1_2md105 | 120.4 | 121.1 | 0.7 | 36.1 | -1.2 | 0.51 |
| 1_2md106 | 121.1 | 122.5 | 1.4 | 38.4 | 2.3 | 0.77 |
| 1_2md107 | 122.5 | 123.6 | 1.1 | 36.4 | -2.1 | 0.94 |
| 1_2md108 | 123.6 | 124.9 | 1.3 | 37.7 | 1.3 | 0.44 |
| 1_2md109 | 124.9 | 125.1 | 0.2 | 36.5 | -1.2 | 0 |
| 1_2md110 | 125.1 | 126 | 0.9 | 38.6 | 2.1 | 1 |
| 1_2md111 | 126 | 126.6 | 0.6 | 43.6 | 4.9 | 1.44 |
| 1_2md112 | 126.6 | 127.3 | 0.7 | 38.8 | -4.7 | 2.38 |
| 1_2md113 | 127.3 | 128.2 | 0.9 | 43.7 | 4.9 | 2.21 |
| 1_2md114 | 128.2 | 129.8 | 1.6 | 39.3 | -4.4 | 1.72 |
| 1_2md115 | 129.8 | 130 | 0.2 | 43.3 | 4.0 | 0 |
| 1_2md116 | 130 | 130.5 | 0.5 | 39.2 | -4.1 | 0.93 |
| 1_2md117 | 130.5 | 131.3 | 0.8 | 42.8 | 3.7 | 2.05 |
| 1_2md118 | 131.3 | 131.5 | 0.2 | 40.2 | -2.6 | 0 |
| 1_2md119 | 131.5 | 131.7 | 0.2 | 41.4 | 1.2 | 0 |
| 1_2md120 | 131.7 | 132.8 | 1.1 | 39.5 | -1.9 | 2.19 |
| 1_2md121 | 132.8 | 133 | 0.2 | 42.2 | 2.7 | 0 |
| 1_2md122 | 133 | 133.3 | 0.3 | 40.7 | -1.5 | 2.72 |
| 1_2md123 | 133.3 | 133.7 | 0.4 | 38.3 | -2.4 | 1.31 |
| 1_2md124 | 133.7 | 134.6 | 0.9 | 43.0 | 4.7 | 1.23 |
| 1_2md125 | 134.6 | 135.9 | 1.3 | 38.6 | -4.4 | 1.48 |
| 1_2md126 | 135.9 | 136.1 | 0.2 | 36.5 | -2.1 | 0 |
| 1_2md127 | 136.1 | 137.7 | 1.6 | 38.6 | 2.0 | 1.94 |
| 1_2md128 | 137.7 | 137.9 | 0.2 | 36.3 | -2.2 | 0 |
| 1_2md129 | 137.9 | 139.6 | 1.7 | 39.7 | 3.4 | 1.88 |
| 1_2md130 | 139.6 | 139.9 | 0.3 | 36.5 | -3.2 | 0.53 |
| 1_2md131 | 139.9 | 141.9 | 2 | 38.7 | 2.3 | 1.52 |
| 1_2md132 | 141.9 | 142.1 | 0.2 | 36.2 | -2.5 | 0 |
| 1_2md133 | 142.1 | 142.4 | 0.3 | 38.3 | 2.1 | 1.07 |
| 1_2md134 | 142.4 | 142.6 | 0.2 | 42.1 | 3.8 | 0 |
| 1_2md135 | 142.6 | 145 | 2.4 | 38.9 | -3.2 | 1.84 |
| 1_2md136 | 145 | 145.4 | 0.4 | 41.5 | 2.6 | 3.64 |
| 1_2md137 | 145.4 | 145.7 | 0.3 | 39.4 | -2.0 | 1.08 |
| 1_2md138 | 145.7 | 146.3 | 0.6 | 42.9 | 3.5 | 2.71 |
| 1_2md139 | 146.3 | 147 | 0.7 | 38.7 | -4.2 | 0.52 |
| 1_2md140 | 147 | 147.2 | 0.2 | 42.3 | 3.6 | 0 |
| 1_2md141 | 147.2 | 147.6 | 0.4 | 40.5 | -1.8 | 1.95 |

| | | | | | | |
|---|---|---|---|---|---|---|
| 1_2md142 | 147.6 | 147.9 | 0.3 | 42.5 | 2.0 | 0.6 |
| 1_2md143 | 147.9 | 148.2 | 0.3 | 39.4 | -3.1 | 1.34 |
| 1_2md144 | 148.2 | 148.4 | 0.2 | 42.4 | 3.0 | 0 |
| 1_2md145 | 148.4 | 149.9 | 1.5 | 39.1 | -3.3 | 1.54 |
| 1_2md146 | 149.9 | 151.1 | 1.2 | 36.3 | -2.8 | 0.46 |
| 1_2md147 | 151.1 | 153.4 | 2.3 | 37.3 | 1.0 | 0.86 |
| 1_2md148 | 153.4 | 156.4 | 3 | 35.9 | -1.4 | 0.8 |
| 1_2md149 | 156.4 | 158 | 1.6 | 38.2 | 2.3 | 1.24 |
| 1_2md150 | 158 | 158.3 | 0.3 | 36.5 | -1.7 | 0.52 |
| 1_2md151 | 158.3 | 160.5 | 2.2 | 38.7 | 2.2 | 1.31 |
| 1_2md152 | 160.5 | 160.8 | 0.3 | 36.3 | -2.4 | 0.7 |
| 1_2md153 | 160.8 | 161 | 0.2 | 38.2 | 1.9 | 0 |
| 1_2md154 | 161 | 161.5 | 0.5 | 36.1 | -2.1 | 1.03 |
| 1_2md155 | 161.5 | 161.8 | 0.3 | 41.3 | 5.2 | 2.92 |
| 1_2md156 | 161.8 | 162.4 | 0.6 | 35.9 | -5.4 | 0.52 |
| 1_2md157 | 162.4 | 163.8 | 1.4 | 39.5 | 3.6 | 2.26 |
| 1_2md158 | 163.8 | 164 | 0.2 | 36.0 | -3.5 | 0 |
| 1_2md159 | 164 | 164.5 | 0.5 | 39.1 | 3.0 | 2.43 |
| 1_2md160 | 164.5 | 165.2 | 0.7 | 35.9 | -3.1 | 1.34 |
| 1_2md161 | 165.2 | 166.2 | 1 | 39.8 | 3.8 | 1.37 |
| 1_2md162 | 166.2 | 166.5 | 0.3 | 43.1 | 3.3 | 1.3 |
| 1_2md163 | 166.5 | 168.2 | 1.7 | 37.9 | -5.2 | 0.83 |
| 1_2md164 | 168.2 | 168.7 | 0.5 | 36.7 | -1.2 | 0.91 |
| 1_2md165 | 168.7 | 168.9 | 0.2 | 37.4 | 0.7 | 0 |
| 1_2md166 | 168.9 | 169.5 | 0.6 | 36.0 | -1.4 | 1.07 |
| 1_2md167 | 169.5 | 169.9 | 0.4 | 38.4 | 2.3 | 1.26 |
| 1_2md168 | 169.9 | 170.5 | 0.6 | 36.5 | -1.8 | 0.91 |
| 1_2md169 | 170.5 | 170.9 | 0.4 | 42.2 | 5.7 | 0.95 |
| 1_2md170 | 170.9 | 171.2 | 0.3 | 39.6 | -2.7 | 1.2 |
| 1_2md171 | 171.2 | 171.8 | 0.6 | 41.2 | 1.6 | 2.39 |
| 1_2md172 | 171.8 | 172.5 | 0.7 | 36.0 | -5.2 | 0.88 |
| 1_2md173 | 172.5 | 177.3 | 4.8 | 37.9 | 1.9 | 1.35 |
| 1_2md174 | 177.3 | 177.5 | 0.2 | 36.6 | -1.4 | 0 |
| 1_2md175 | 177.5 | 179.8 | 2.3 | 37.7 | 1.2 | 0.85 |
| 1_2md176 | 179.8 | 180.5 | 0.7 | 35.1 | -2.6 | 1.11 |
| 1_2md177 | 180.5 | 181 | 0.5 | 38.1 | 3.0 | 0.38 |
| 1_2md178 | 181 | 181.2 | 0.2 | 36.3 | -1.8 | 0 |
| 1_2md179 | 181.2 | 182.5 | 1.3 | 38.2 | 1.9 | 0.65 |
| 1_2md180 | 182.5 | 183.1 | 0.6 | 42.3 | 4.1 | 1.2 |
| 1_2md181 | 183.1 | 183.3 | 0.2 | 38.5 | -3.8 | 0 |
| 1_2md182 | 183.3 | 183.5 | 0.2 | 35.5 | -3.0 | 0 |
| 1_2md183 | 183.5 | 183.9 | 0.4 | 39.9 | 4.5 | 2 |
| 1_2md184 | 183.9 | 184.1 | 0.2 | 41.5 | 1.6 | 0 |
| 1_2md185 | 184.1 | 185.4 | 1.3 | 38.1 | -3.4 | 1.67 |
| 1_2md186 | 185.4 | 187.1 | 1.7 | 36.3 | -1.7 | 0.59 |
| 1_2md187 | 187.1 | 187.4 | 0.3 | 37.8 | 1.5 | 0.72 |
| 1_2md188 | 187.4 | 188.3 | 0.9 | 35.8 | -2.0 | 1.44 |
| 1_2md189 | 188.3 | 190.4 | 2.1 | 38.1 | 2.4 | 1.38 |
| 1_2md190 | 190.4 | 190.9 | 0.5 | 36.5 | -1.6 | 0.21 |
| 1_2md191 | 190.9 | 192.3 | 1.4 | 38.9 | 2.4 | 1.61 |
| 1_2md192 | 192.3 | 192.5 | 0.2 | 41.1 | 2.2 | 0 |
| 1_2md193 | 192.5 | 193.6 | 1.1 | 39.8 | -1.3 | 2.18 |
| 1_2md194 | 193.6 | 193.9 | 0.3 | 43.2 | 3.4 | 1.48 |
| 1_2md195 | 193.9 | 194.1 | 0.2 | 40.1 | -3.2 | 0 |
| 1_2md196 | 194.1 | 194.7 | 0.6 | 42.5 | 2.4 | 2.02 |
| 1_2md197 | 194.7 | 195 | 0.3 | 39.8 | -2.7 | 0.79 |

| | | | | | | |
|---|---|---|---|---|---|---|
| 1_2md198 | 195 | 195.3 | 0.3 | 41.5 | 1.7 | 0.45 |
| 1_2md199 | 195.3 | 195.5 | 0.2 | 39.8 | -1.7 | 0 |
| 1_2md200 | 195.5 | 196.1 | 0.6 | 42.1 | 2.3 | 0.61 |
| 1_2md201 | 196.1 | 196.5 | 0.4 | 39.5 | -2.6 | 1.01 |
| 1_2md202 | 196.5 | 196.7 | 0.2 | 43.2 | 3.6 | 0 |
| 1_2md203 | 196.7 | 197.2 | 0.5 | 40.2 | -3.0 | 1.05 |
| 1_2md204 | 197.2 | 198.5 | 1.3 | 42.8 | 2.6 | 2.06 |
| 1_2md205 | 198.5 | 198.7 | 0.2 | 38.1 | -4.7 | 0 |
| 1_2md206 | 198.7 | 199.4 | 0.7 | 41.2 | 3.1 | 0.79 |
| 1_2md207 | 199.4 | 199.6 | 0.2 | 40.0 | -1.2 | 0 |
| 1_2md208 | 199.6 | 200 | 0.4 | 41.5 | 1.5 | 0.55 |
| 1_2md209 | 200 | 200.4 | 0.4 | 40.3 | -1.2 | 0.73 |
| 1_2md210 | 200.4 | 200.7 | 0.3 | 36.1 | -4.2 | 0.41 |
| 1_2md211 | 200.7 | 201.2 | 0.5 | 42.1 | 6.0 | 1.46 |
| 1_2md212 | 201.2 | 201.5 | 0.3 | 38.9 | -3.2 | 1.68 |
| 1_2md213 | 201.5 | 201.7 | 0.2 | 35.6 | -3.3 | 0 |
| 1_2md214 | 201.7 | 202.3 | 0.6 | 41.6 | 5.9 | 1.97 |
| 1_2md215 | 202.3 | 202.5 | 0.2 | 38.2 | -3.3 | 0 |
| 1_2md216 | 202.5 | 202.7 | 0.2 | 42.1 | 3.9 | 0 |
| 1_2md217 | 202.7 | 203.3 | 0.6 | 38.7 | -3.4 | 2.06 |
| 1_2md218 | 203.3 | 203.5 | 0.2 | 43.0 | 4.3 | 0 |
| 1_2md219 | 203.5 | 204.4 | 0.9 | 38.5 | -4.5 | 1.6 |
| 1_2md220 | 204.4 | 204.6 | 0.2 | 43.6 | 5.2 | 0 |
| 1_2md221 | 204.6 | 205 | 0.4 | 39.3 | -4.4 | 1.09 |
| 1_2md222 | 205 | 205.3 | 0.3 | 42.3 | 3.0 | 0.62 |
| 1_2md223 | 205.3 | 206.4 | 1.1 | 39.7 | -2.6 | 1.19 |
| 1_2md224 | 206.4 | 207 | 0.6 | 42.6 | 2.9 | 1.08 |
| 1_2md225 | 207 | 207.3 | 0.3 | 38.0 | -4.6 | 0.44 |
| 1_2md226 | 207.3 | 207.5 | 0.2 | 36.8 | -1.2 | 0 |
| 1_2md227 | 207.5 | 209.1 | 1.6 | 38.6 | 1.8 | 0.98 |
| 1_2md228 | 209.1 | 209.5 | 0.4 | 42.4 | 3.8 | 0.8 |
| 1_2md229 | 209.5 | 209.8 | 0.3 | 37.9 | -4.5 | 1.9 |
| 1_2md230 | 209.8 | 210 | 0.2 | 43.1 | 5.2 | 0 |
| 1_2md231 | 210 | 211.6 | 1.6 | 38.5 | -4.6 | 1.29 |
| 1_2md232 | 211.6 | 212.6 | 1 | 36.3 | -2.2 | 0.58 |
| 1_2md233 | 212.6 | 213.2 | 0.6 | 38.1 | 1.8 | 0.29 |
| 1_2md234 | 213.2 | 214 | 0.8 | 42.5 | 4.4 | 2.45 |
| 1_2md235 | 214 | 214.4 | 0.4 | 37.8 | -4.7 | 1.76 |
| 1_2md236 | 214.4 | 214.9 | 0.5 | 43.9 | 6.1 | 1.19 |
| 1_2md237 | 214.9 | 215.4 | 0.5 | 39.7 | -4.2 | 2.77 |
| 1_2md238 | 215.4 | 215.7 | 0.3 | 35.2 | -4.5 | 0.69 |
| 1_2md239 | 215.7 | 216 | 0.3 | 42.9 | 7.7 | 0.75 |
| 1_2md240 | 216 | 219.8 | 3.8 | 39.1 | -3.8 | 1.54 |
| 1_2md241 | 219.8 | 220 | 0.2 | 36.5 | -2.5 | 0 |
| 1_2md242 | 220 | 223.3 | 3.3 | 38.6 | 2.0 | 1.85 |
| 1_2md243 | 223.3 | 227.7 | 4.4 | 35.8 | -2.8 | 0.76 |
| 1_2md244 | 227.7 | 228.1 | 0.4 | 37.7 | 1.9 | 0.88 |
| 1_2md245 | 228.1 | 228.4 | 0.3 | 34.7 | -3.0 | 1.22 |
| 1_2md246 | 228.4 | 229 | 0.6 | 38.1 | 3.5 | 0.47 |
| 1_2md247 | 229 | 229.2 | 0.2 | 35.7 | -2.4 | 0 |
| 1_2md248 | 229.2 | 230.8 | 1.6 | 38.9 | 3.2 | 1.35 |
| 1_2md249 | 230.8 | 231 | 0.2 | 36.5 | -2.3 | 0 |
| 1_2md250 | 231 | 232 | 1 | 39.0 | 2.5 | 0.5 |
| 1_2md251 | 232 | 232.2 | 0.2 | 36.2 | -2.8 | 0 |
| 1_2md252 | 232.2 | 232.8 | 0.6 | 37.6 | 1.3 | 0.97 |
| 1_2md253 | 232.8 | 235.5 | 2.7 | 35.6 | -1.9 | 0.79 |

| | | | | | | |
|---|---|---|---|---|---|---|
| 1_2md254 | 235.5 | 235.9 | 0.4 | 37.7 | 2.0 | 0.51 |
| 1_2md255 | 235.9 | 236.1 | 0.2 | 36.3 | -1.4 | 0 |
| 1_2md256 | 236.1 | 237 | 0.9 | 38.2 | 1.9 | 0.79 |
| 1_2md257 | 237 | 237.2 | 0.2 | 35.9 | -2.3 | 0 |
| 1_2md258 | 237.2 | 237.7 | 0.5 | 36.9 | 1.0 | 0.22 |
| 1_2md259 | 237.7 | 239.8 | 2.1 | 38.7 | 1.8 | 1.13 |
| 1_2md260 | 239.8 | 240.3 | 0.5 | 42.1 | 3.4 | 1.63 |
| 1_2md261 | 240.3 | 241 | 0.7 | 39.0 | -3.2 | 1.25 |
| 1_2md262 | 241 | 241.3 | 0.3 | 41.6 | 2.6 | 1.11 |
| 1_2md263 | 241.3 | 241.6 | 0.3 | 36.4 | -5.2 | 0.35 |
| 1_2md264 | 241.6 | 241.9 | 0.3 | 39.4 | 3.0 | 1.42 |
| 1_2md265 | 241.9 | 242.2 | 0.3 | 42.0 | 2.5 | 0.57 |
| 1_2md266 | 242.2 | 242.7 | 0.5 | 40.1 | -1.8 | 0.58 |
| 1_2md267 | 242.7 | 242.9 | 0.2 | 43.4 | 3.3 | 0 |
| 1_2md268 | 242.9 | 243.1 | 0.2 | 34.8 | -8.6 | 0 |
| 1_2md269 | 243.1 | 243.9 | 0.8 | 39.7 | 5.0 | 1.27 |
| 1_2md270 | 243.9 | 244.2 | 0.3 | 42.2 | 2.5 | 1.19 |
| 1_2md271 | 244.2 | 244.6 | 0.4 | 40.3 | -1.9 | 1.88 |
| 1_2md272 | 244.6 | 244.9 | 0.3 | 42.2 | 1.8 | 0.09 |
| 1_2md273 | 244.9 | 245.2 | 0.3 | 39.7 | -2.4 | 0.99 |
| 1_2md274 | 245.2 | 245.6 | 0.4 | 42.1 | 2.4 | 0.98 |
| 1_2md275 | 245.6 | 245.9 | 0.3 | 41.3 | -0.9 | 1.04 |
| 1_2md276 | 245.9 | 246.3 | 0.4 | 43.6 | 2.4 | 1.46 |
| 1_2md277 | 246.3 | 247.2 | 0.9 | 39.1 | -4.6 | 1.87 |
| 1_2md278 | 247.2 | 247.4 | 0.2 | 41.9 | 2.8 | 0 |
| 1_2md279 | 247.4 | 249.1 | 1.7 | 39.4 | -2.5 | 0.99 |
| 1_2md280 | 249.1 | 249.3 | 0.2 | 35.7 | -3.7 | 0 |
| 1_2md281 | 249.3 | 249.9 | 0.6 | 38.7 | 3.0 | 0.73 |
| 1_2md282 | 249.9 | 250.1 | 0.2 | 42.4 | 3.7 | 0 |
| 1_2md283 | 250.1 | 250.3 | 0.2 | 39.4 | -3.0 | 0 |
| 1_2md284 | 250.3 | 250.9 | 0.6 | 36.5 | -2.9 | 0.6 |
| 1_2md285 | 250.9 | 251.9 | 1 | 39.3 | 2.8 | 1.69 |
| 1_2md286 | 251.9 | 252.1 | 0.2 | 41.6 | 2.3 | 0 |
| 1_2md287 | 252.1 | 252.5 | 0.4 | 39.5 | -2.1 | 1.13 |
| 1_2md288 | 252.5 | 252.9 | 0.4 | 42.6 | 3.1 | 0.75 |
| 1_2md289 | 252.9 | 253.6 | 0.7 | 39.5 | -3.1 | 1.2 |
| 1_2md290 | 253.6 | 254.2 | 0.6 | 35.6 | -3.9 | 0.57 |
| 1_2md291 | 254.2 | 254.4 | 0.2 | 43.3 | 7.7 | 0 |
| 1_2md292 | 254.4 | 255.8 | 1.4 | 38.9 | -4.4 | 1.48 |
| | | | | | | |
| 1_3md1 | 0 | 1.6 | 1.6 | 38.1 | | 0.72 |
| 1_3md2 | 1.6 | 1.8 | 0.2 | 35.8 | -2.3 | 0 |
| 1_3md3 | 1.8 | 2.1 | 0.3 | 37.5 | 1.7 | 0.33 |
| 1_3md4 | 2.1 | 2.3 | 0.2 | 36.7 | -0.8 | 0 |
| 1_3md5 | 2.3 | 3.6 | 1.3 | 37.8 | 1.1 | 0.91 |
| 1_3md6 | 3.6 | 4.8 | 1.2 | 36.0 | -1.8 | 1.7 |
| 1_3md7 | 4.8 | 5.1 | 0.3 | 37.9 | 1.8 | 0.38 |
| 1_3md8 | 5.1 | 10.7 | 5.6 | 35.3 | -2.6 | 0.81 |
| 1_3md9 | 10.7 | 10.9 | 0.2 | 38.0 | 2.7 | 0 |
| 1_3md10 | 10.9 | 11.5 | 0.6 | 35.5 | -2.5 | 0.81 |
| 1_3md11 | 11.5 | 13.2 | 1.7 | 38.4 | 2.9 | 1.22 |
| 1_3md12 | 13.2 | 15.9 | 2.7 | 36.1 | -2.3 | 0.74 |
| 1_3md13 | 15.9 | 18 | 2.1 | 38.2 | 2.1 | 0.97 |
| 1_3md14 | 18 | 18.2 | 0.2 | 36.3 | -1.9 | 0 |
| 1_3md15 | 18.2 | 19 | 0.8 | 38.1 | 1.8 | 0.98 |
| 1_3md16 | 19 | 19.2 | 0.2 | 36.3 | -1.9 | 0 |

| | | | | | | |
|---|---|---|---|---|---|---|
| 1_3md17 | 19.2 | 19.5 | 0.3 | 37.9 | 1.6 | 0.37 |
| 1_3md18 | 19.5 | 19.9 | 0.4 | 35.1 | -2.8 | 1.28 |
| 1_3md19 | 19.9 | 20.4 | 0.5 | 38.8 | 3.7 | 0.67 |
| 1_3md20 | 20.4 | 22.7 | 2.3 | 35.8 | -3.0 | 0.81 |
| 1_3md21 | 22.7 | 22.9 | 0.2 | 37.2 | 1.4 | 0 |
| 1_3md22 | 22.9 | 27.8 | 4.9 | 35.4 | -1.8 | 0.94 |
| 1_3md23 | 27.8 | 28.3 | 0.5 | 38.1 | 2.8 | 0.58 |
| 1_3md24 | 28.3 | 28.7 | 0.4 | 36.4 | -1.7 | 0.58 |
| 1_3md25 | 28.7 | 29 | 0.3 | 37.6 | 1.2 | 0.16 |
| 1_3md26 | 29 | 30.9 | 1.9 | 36.0 | -1.6 | 0.65 |
| 1_3md27 | 30.9 | 31.1 | 0.2 | 37.2 | 1.3 | 0 |
| 1_3md28 | 31.1 | 35.8 | 4.7 | 35.1 | -2.1 | 1 |
| 1_3md29 | 35.8 | 36 | 0.2 | 37.9 | 2.8 | 0 |
| 1_3md30 | 36 | 48 | 12 | 35.0 | -3.0 | 1.32 |
| 1_3md31 | 48 | 49 | 1 | 38.9 | 4.0 | 0.82 |
| 1_3md32 | 49 | 49.2 | 0.2 | 34.9 | -4.1 | 0 |
| 1_3md33 | 49.2 | 50.2 | 1 | 38.2 | 3.3 | 1.37 |
| 1_3md34 | 50.2 | 50.5 | 0.3 | 36.4 | -1.8 | 0.41 |
| 1_3md35 | 50.5 | 53.7 | 3.2 | 38.4 | 2.0 | 1.26 |
| 1_3md36 | 53.7 | 54.6 | 0.9 | 36.4 | -2.0 | 0.52 |
| 1_3md37 | 54.6 | 55.1 | 0.5 | 39.0 | 2.6 | 1.47 |
| 1_3md38 | 55.1 | 59.8 | 4.7 | 35.6 | -3.4 | 0.73 |
| 1_3md39 | 59.8 | 61.4 | 1.6 | 39.6 | 4.0 | 1.76 |
| 1_3md40 | 61.4 | 61.6 | 0.2 | 35.9 | -3.7 | 0 |
| 1_3md41 | 61.6 | 62.6 | 1 | 39.7 | 3.7 | 0.77 |
| 1_3md42 | 62.6 | 63.2 | 0.6 | 35.6 | -4.1 | 0.71 |
| 1_3md43 | 63.2 | 63.6 | 0.4 | 37.5 | 1.9 | 0.24 |
| 1_3md44 | 63.6 | 63.9 | 0.3 | 36.4 | -1.1 | 0.45 |
| 1_3md45 | 63.9 | 64.1 | 0.2 | 37.6 | 1.2 | 0 |
| 1_3md46 | 64.1 | 64.5 | 0.4 | 36.0 | -1.6 | 0.67 |
| 1_3md47 | 64.5 | 67 | 2.5 | 38.7 | 2.7 | 1.6 |
| 1_3md48 | 67 | 67.2 | 0.2 | 36.1 | -2.6 | 0 |
| 1_3md49 | 67.2 | 67.5 | 0.3 | 37.7 | 1.6 | 1.35 |
| 1_3md50 | 67.5 | 67.8 | 0.3 | 36.5 | -1.2 | 0.16 |
| 1_3md51 | 67.8 | 68.6 | 0.8 | 38.2 | 1.7 | 0.61 |
| 1_3md52 | 68.6 | 69.5 | 0.9 | 36.8 | -1.4 | 0.88 |
| 1_3md53 | 69.5 | 72.4 | 2.9 | 38.6 | 1.8 | 1.33 |
| 1_3md54 | 72.4 | 72.7 | 0.3 | 44.5 | 5.9 | 0.94 |
| 1_3md55 | 72.7 | 73.1 | 0.4 | 38.8 | -5.8 | 2.5 |
| 1_3md56 | 73.1 | 73.7 | 0.6 | 43.4 | 4.6 | 1.84 |
| 1_3md57 | 73.7 | 74.7 | 1 | 39.1 | -4.3 | 1.88 |
| 1_3md58 | 74.7 | 74.9 | 0.2 | 36.3 | -2.7 | 0 |
| 1_3md59 | 74.9 | 75.8 | 0.9 | 38.6 | 2.3 | 1.48 |
| 1_3md60 | 75.8 | 76.3 | 0.5 | 36.2 | -2.4 | 0.38 |
| 1_3md61 | 76.3 | 76.5 | 0.2 | 38.1 | 1.9 | 0 |
| 1_3md62 | 76.5 | 76.7 | 0.2 | 36.7 | -1.4 | 0 |
| 1_3md63 | 76.7 | 76.9 | 0.2 | 37.3 | 0.6 | 0 |
| 1_3md64 | 76.9 | 77.1 | 0.2 | 34.8 | -2.6 | 0 |
| 1_3md65 | 77.1 | 78.1 | 1 | 38.1 | 3.3 | 1.26 |
| 1_3md66 | 78.1 | 78.3 | 0.2 | 36.2 | -1.9 | 0 |
| 1_3md67 | 78.3 | 81.6 | 3.3 | 39.0 | 2.8 | 1.47 |
| 1_3md68 | 81.6 | 82.9 | 1.3 | 36.6 | -2.4 | 1.02 |
| 1_3md69 | 82.9 | 83.2 | 0.3 | 37.5 | 0.9 | 0.04 |
| 1_3md70 | 83.2 | 83.5 | 0.3 | 37.4 | -0.2 | 1.82 |
| 1_3md71 | 83.5 | 84.2 | 0.7 | 37.2 | -0.1 | 1.02 |
| 1_3md72 | 84.2 | 85.8 | 1.6 | 36.0 | -1.3 | 0.89 |

| | | | | | | |
|---|---|---|---|---|---|---|
| 1_3md73 | 85.8 | 86 | 0.2 | 37.3 | 1.4 | 0 |
| 1_3md74 | 86 | 90.8 | 4.8 | 36.0 | -1.4 | 1.15 |
| 1_3md75 | 90.8 | 91.7 | 0.9 | 37.4 | 1.5 | 0.44 |
| 1_3md76 | 91.7 | 91.9 | 0.2 | 36.4 | -1.0 | 0 |
| 1_3md77 | 91.9 | 92.1 | 0.2 | 37.4 | 0.9 | 0 |
| 1_3md78 | 92.1 | 92.3 | 0.2 | 36.9 | -0.5 | 0 |
| 1_3md79 | 92.3 | 92.6 | 0.3 | 37.7 | 0.8 | 0.12 |
| 1_3md80 | 92.6 | 93 | 0.4 | 36.6 | -1.1 | 0.28 |
| 1_3md81 | 93 | 94.4 | 1.4 | 38.3 | 1.7 | 0.79 |
| 1_3md82 | 94.4 | 94.9 | 0.5 | 36.1 | -2.1 | 0.36 |
| 1_3md83 | 94.9 | 95.3 | 0.4 | 37.5 | 1.3 | 0.54 |
| 1_3md84 | 95.3 | 95.6 | 0.3 | 35.6 | -1.8 | 0.6 |
| 1_3md85 | 95.6 | 96 | 0.4 | 37.4 | 1.8 | 0.27 |
| 1_3md86 | 96 | 102.7 | 6.7 | 35.4 | -2.0 | 1.21 |
| 1_3md87 | 102.7 | 103 | 0.3 | 38.6 | 3.2 | 1.44 |
| 1_3md88 | 103 | 103.7 | 0.7 | 36.4 | -2.2 | 0.4 |
| 1_3md89 | 103.7 | 104.1 | 0.4 | 36.6 | 0.2 | 1.57 |
| 1_3md90 | 104.1 | 106.5 | 2.4 | 34.9 | -1.7 | 1.13 |
| 1_3md91 | 106.5 | 106.7 | 0.2 | 37.4 | 2.5 | 0 |
| 1_3md92 | 106.7 | 110.7 | 4 | 35.8 | -1.6 | 0.93 |
| 1_3md93 | 110.7 | 111.1 | 0.4 | 37.2 | 1.5 | 0.13 |
| 1_3md94 | 111.1 | 111.5 | 0.4 | 36.5 | -0.7 | 0.2 |
| 1_3md95 | 111.5 | 112.2 | 0.7 | 37.7 | 1.2 | 0.74 |
| 1_3md96 | 112.2 | 114.5 | 2.3 | 35.9 | -1.8 | 0.76 |
| 1_3md97 | 114.5 | 115.7 | 1.2 | 38.6 | 2.7 | 1.34 |
| 1_3md98 | 115.7 | 115.9 | 0.2 | 36.3 | -2.3 | 0 |
| 1_3md99 | 115.9 | 116.5 | 0.6 | 38.5 | 2.2 | 0.92 |
| 1_3md100 | 116.5 | 117 | 0.5 | 36.4 | -2.1 | 1.03 |
| 1_3md101 | 117 | 117.3 | 0.3 | 37.9 | 1.6 | 0.32 |
| 1_3md102 | 117.3 | 118.4 | 1.1 | 35.7 | -2.2 | 1.42 |
| 1_3md103 | 118.4 | 118.9 | 0.5 | 38.1 | 2.4 | 0.71 |
| 1_3md104 | 118.9 | 120.5 | 1.6 | 36.0 | -2.1 | 1.48 |
| 1_3md105 | 120.5 | 121.8 | 1.3 | 38.2 | 2.2 | 1.02 |
| 1_3md106 | 121.8 | 126.7 | 4.9 | 35.5 | -2.7 | 0.75 |
| 1_3md107 | 126.7 | 126.9 | 0.2 | 37.8 | 2.3 | 0 |
| 1_3md108 | 126.9 | 127.9 | 1 | 35.9 | -1.9 | 0.79 |
| 1_3md109 | 127.9 | 128.3 | 0.4 | 37.7 | 1.8 | 0.86 |
| 1_3md110 | 128.3 | 128.6 | 0.3 | 36.0 | -1.7 | 0.69 |
| 1_3md111 | 128.6 | 128.8 | 0.2 | 38.9 | 3.0 | 0 |
| 1_3md112 | 128.8 | 136.7 | 7.9 | 35.2 | -3.7 | 1.12 |
| 1_3md113 | 136.7 | 137.8 | 1.1 | 37.2 | 2.0 | 0.78 |
| 1_3md114 | 137.8 | 138.1 | 0.3 | 36.6 | -0.6 | 0.05 |
| 1_3md115 | 138.1 | 139 | 0.9 | 39.1 | 2.5 | 1.23 |
| 1_3md116 | 139 | 139.3 | 0.3 | 43.9 | 4.8 | 2.09 |
| 1_3md117 | 139.3 | 141.1 | 1.8 | 39.1 | -4.8 | 1.22 |
| 1_3md118 | 141.1 | 141.4 | 0.3 | 35.2 | -3.9 | 0.43 |
| 1_3md119 | 141.4 | 142.5 | 1.1 | 38.4 | 3.2 | 1.42 |
| 1_3md120 | 142.5 | 143 | 0.5 | 35.5 | -3.0 | 0.53 |
| 1_3md121 | 143 | 143.6 | 0.6 | 37.7 | 2.3 | 1.16 |
| 1_3md122 | 143.6 | 143.8 | 0.2 | 36.8 | -1.0 | 0 |
| 1_3md123 | 143.8 | 145.1 | 1.3 | 39.8 | 3.0 | 1.44 |
| 1_3md124 | 145.1 | 145.3 | 0.2 | 34.7 | -5.1 | 0 |
| 1_3md125 | 145.3 | 146.4 | 1.1 | 38.2 | 3.5 | 1.69 |
| 1_3md126 | 146.4 | 149.3 | 2.9 | 35.2 | -3.1 | 1.01 |
| 1_3md127 | 149.3 | 149.8 | 0.5 | 37.8 | 2.6 | 0.52 |
| 1_3md128 | 149.8 | 150.1 | 0.3 | 36.4 | -1.4 | 0.21 |

| | | | | | | |
|---|---|---|---|---|---|---|
| 1_3md129 | 150.1 | 151.7 | 1.6 | 37.7 | 1.3 | 1.06 |
| 1_3md130 | 151.7 | 151.9 | 0.2 | 36.6 | -1.1 | 0 |
| 1_3md131 | 151.9 | 152.8 | 0.9 | 37.6 | 1.0 | 0.55 |
| 1_3md132 | 152.8 | 153.1 | 0.3 | 35.9 | -1.6 | 0.84 |
| 1_3md133 | 153.1 | 153.3 | 0.2 | 37.4 | 1.5 | 0 |
| 1_3md134 | 153.3 | 153.5 | 0.2 | 36.4 | -1.0 | 0 |
| 1_3md135 | 153.5 | 153.9 | 0.4 | 38.0 | 1.6 | 0.36 |
| 1_3md136 | 153.9 | 154.1 | 0.2 | 36.2 | -1.8 | 0 |
| 1_3md137 | 154.1 | 155.9 | 1.8 | 39.0 | 2.8 | 1.34 |
| 1_3md138 | 155.9 | 156.6 | 0.7 | 35.6 | -3.3 | 0.69 |
| 1_3md139 | 156.6 | 157.9 | 1.3 | 37.8 | 2.2 | 0.9 |
| 1_3md140 | 157.9 | 170.3 | 12.4 | 35.2 | -2.7 | 1.01 |
| 1_3md141 | 170.3 | 171.2 | 0.9 | 38.2 | 3.0 | 1.07 |
| 1_3md142 | 171.2 | 171.7 | 0.5 | 41.3 | 3.1 | 0.98 |
| 1_3md143 | 171.7 | 172 | 0.3 | 39.0 | -2.3 | 1.43 |
| 1_3md144 | 172 | 172.2 | 0.2 | 42.2 | 3.2 | 0 |
| 1_3md145 | 172.2 | 172.8 | 0.6 | 39.1 | -3.1 | 0.82 |
| 1_3md146 | 172.8 | 173.1 | 0.3 | 46.9 | 7.8 | 2.69 |
| 1_3md147 | 173.1 | 173.4 | 0.3 | 42.3 | -4.6 | 2 |
| 1_3md148 | 173.4 | 173.7 | 0.3 | 38.5 | -3.8 | 0.17 |
| 1_3md149 | 173.7 | 173.9 | 0.2 | 44.1 | 5.6 | 0 |
| 1_3md150 | 173.9 | 174.9 | 1 | 40.4 | -3.7 | 2.75 |
| 1_3md151 | 174.9 | 175.1 | 0.2 | 41.5 | 1.1 | 0 |
| 1_3md152 | 175.1 | 177.5 | 2.4 | 40.0 | -1.5 | 1.48 |
| 1_3md153 | 177.5 | 178.8 | 1.3 | 43.6 | 3.6 | 1.39 |
| 1_3md154 | 178.8 | 181.8 | 3 | 38.8 | -4.9 | 1.04 |
| 1_3md155 | 181.8 | 182.3 | 0.5 | 41.5 | 2.7 | 1.59 |
| 1_3md156 | 182.3 | 182.6 | 0.3 | 36.7 | -4.8 | 2.34 |
| 1_3md157 | 182.6 | 183.2 | 0.6 | 39.6 | 2.9 | 0.96 |
| 1_3md158 | 183.2 | 183.8 | 0.6 | 43.6 | 4.1 | 1.22 |
| 1_3md159 | 183.8 | 184.1 | 0.3 | 39.4 | -4.2 | 1.46 |
| 1_3md160 | 184.1 | 185.1 | 1 | 43.8 | 4.4 | 1.38 |
| 1_3md161 | 185.1 | 185.3 | 0.2 | 38.3 | -5.6 | 0 |
| 1_3md162 | 185.3 | 185.5 | 0.2 | 44.3 | 6.1 | 0 |
| 1_3md163 | 185.5 | 185.9 | 0.4 | 36.5 | -7.8 | 0.7 |
| 1_3md164 | 185.9 | 186.8 | 0.9 | 40.3 | 3.8 | 2.79 |
| 1_3md165 | 186.8 | 187.1 | 0.3 | 42.6 | 2.3 | 1.1 |
| 1_3md166 | 187.1 | 188 | 0.9 | 39.9 | -2.7 | 1.88 |
| 1_3md167 | 188 | 188.2 | 0.2 | 35.4 | -4.5 | 0 |
| 1_3md168 | 188.2 | 188.6 | 0.4 | 38.5 | 3.1 | 1.7 |
| 1_3md169 | 188.6 | 189 | 0.4 | 36.3 | -2.2 | 0.71 |
| 1_3md170 | 189 | 190.4 | 1.4 | 38.9 | 2.6 | 1.4 |
| 1_3md171 | 190.4 | 190.6 | 0.2 | 36.6 | -2.3 | 0 |
| 1_3md172 | 190.6 | 191 | 0.4 | 37.7 | 1.1 | 0.69 |
| 1_3md173 | 191 | 191.2 | 0.2 | 36.8 | -0.9 | 0 |
| 1_3md174 | 191.2 | 192.6 | 1.4 | 38.9 | 2.1 | 1.46 |
| 1_3md175 | 192.6 | 193 | 0.4 | 45.0 | 6.1 | 0.48 |
| 1_3md176 | 193 | 193.2 | 0.2 | 50.4 | 5.4 | 0 |
| 1_3md177 | 193.2 | 193.8 | 0.6 | 43.2 | -7.3 | 2.86 |
| 1_3md178 | 193.8 | 194.2 | 0.4 | 39.3 | -3.9 | 0.35 |
| 1_3md179 | 194.2 | 194.5 | 0.3 | 46.5 | 7.2 | 2.44 |
| 1_3md180 | 194.5 | 194.7 | 0.2 | 45.0 | -1.5 | 0 |
| 1_3md181 | 194.7 | 195.1 | 0.4 | 45.9 | 0.9 | 2.54 |
| 1_3md182 | 195.1 | 195.3 | 0.2 | 45.3 | -0.6 | 0 |
| 1_3md183 | 195.3 | 195.6 | 0.3 | 48.0 | 2.7 | 3.33 |
| 1_3md184 | 195.6 | 196.3 | 0.7 | 39.7 | -8.3 | 1.24 |

| | | | | | | |
|---|---|---|---|---|---|---|
| 1_3md185 | 196.3 | 196.9 | 0.6 | 43.6 | 3.9 | 2.52 |
| 1_3md186 | 196.9 | 197.2 | 0.3 | 45.1 | 1.5 | 3.4 |
| 1_3md187 | 197.2 | 197.7 | 0.5 | 38.6 | -6.4 | 0.97 |
| 1_3md188 | 197.7 | 198.1 | 0.4 | 42.6 | 3.9 | 3.86 |
| 1_3md189 | 198.1 | 198.3 | 0.2 | 39.4 | -3.2 | 0 |
| 1_3md190 | 198.3 | 198.7 | 0.4 | 44.4 | 5.0 | 3.13 |
| 1_3md191 | 198.7 | 198.9 | 0.2 | 40.4 | -3.9 | 0 |
| 1_3md192 | 198.9 | 199.2 | 0.3 | 42.7 | 2.3 | 1.73 |
| 1_3md193 | 199.2 | 199.4 | 0.2 | 39.9 | -2.8 | 0 |
| 1_3md194 | 199.4 | 199.9 | 0.5 | 43.1 | 3.1 | 1.12 |
| 1_3md195 | 199.9 | 200.3 | 0.4 | 39.5 | -3.5 | 0.95 |
| 1_3md196 | 200.3 | 200.7 | 0.4 | 42.8 | 3.3 | 1.52 |
| 1_3md197 | 200.7 | 201.8 | 1.1 | 38.3 | -4.6 | 1.31 |
| 1_3md198 | 201.8 | 202.9 | 1.1 | 42.4 | 4.2 | 0.99 |
| 1_3md199 | 202.9 | 204.1 | 1.2 | 39.0 | -3.5 | 1.33 |
| 1_3md200 | 204.1 | 204.7 | 0.6 | 42.2 | 3.3 | 2.15 |
| 1_3md201 | 204.7 | 204.9 | 0.2 | 40.4 | -1.9 | 0 |
| 1_3md202 | 204.9 | 206.7 | 1.8 | 43.7 | 3.3 | 2.19 |
| 1_3md203 | 206.7 | 206.9 | 0.2 | 39.3 | -4.4 | 0 |
| 1_3md204 | 206.9 | 207.2 | 0.3 | 43.3 | 4.0 | 0.8 |
| 1_3md205 | 207.2 | 207.4 | 0.2 | 39.7 | -3.7 | 0 |
| 1_3md206 | 207.4 | 207.6 | 0.2 | 43.5 | 3.8 | 0 |
| 1_3md207 | 207.6 | 208.6 | 1 | 39.2 | -4.3 | 1.88 |
| 1_3md208 | 208.6 | 212.5 | 3.9 | 34.8 | -4.4 | 0.92 |
| 1_3md209 | 212.5 | 213.5 | 1 | 38.0 | 3.2 | 0.8 |
| 1_3md210 | 213.5 | 214.4 | 0.9 | 35.9 | -2.1 | 0.81 |
| 1_3md211 | 214.4 | 215 | 0.6 | 39.0 | 3.0 | 0.79 |
| 1_3md212 | 215 | 215.6 | 0.6 | 43.8 | 4.9 | 2.05 |
| 1_3md213 | 215.6 | 216.1 | 0.5 | 48.1 | 4.3 | 3.03 |
| 1_3md214 | 216.1 | 216.3 | 0.2 | 38.8 | -9.3 | 0 |
| 1_3md215 | 216.3 | 216.6 | 0.3 | 41.5 | 2.7 | 0.07 |
| 1_3md216 | 216.6 | 217 | 0.4 | 50.4 | 8.9 | 3.4 |
| 1_3md217 | 217 | 217.2 | 0.2 | 44.2 | -6.2 | 0 |
| 1_3md218 | 217.2 | 217.8 | 0.6 | 47.5 | 3.3 | 2.74 |
| 1_3md219 | 217.8 | 218.1 | 0.3 | 43.9 | -3.6 | 2.46 |
| 1_3md220 | 218.1 | 218.3 | 0.2 | 50.1 | 6.3 | 0 |
| 1_3md221 | 218.3 | 218.6 | 0.3 | 42.4 | -7.7 | 1.13 |
| 1_3md222 | 218.6 | 218.9 | 0.3 | 38.6 | -3.8 | 2.02 |
| 1_3md223 | 218.9 | 223.8 | 4.9 | 35.4 | -3.2 | 1.04 |
| 1_3md224 | 223.8 | 224.3 | 0.5 | 39.4 | 4.0 | 1.2 |
| 1_3md225 | 224.3 | 225.9 | 1.6 | 43.3 | 3.9 | 1.19 |
| 1_3md226 | 225.9 | 226.4 | 0.5 | 38.6 | -4.7 | 1.17 |
| 1_3md227 | 226.4 | 226.8 | 0.4 | 37.0 | -1.6 | 0.67 |
| 1_3md228 | 226.8 | 227.5 | 0.7 | 38.2 | 1.2 | 0.49 |
| 1_3md229 | 227.5 | 228.1 | 0.6 | 43.0 | 4.8 | 0.94 |
| 1_3md230 | 228.1 | 228.3 | 0.2 | 47.8 | 4.8 | 0 |
| 1_3md231 | 228.3 | 228.6 | 0.3 | 42.0 | -5.8 | 2.34 |
| 1_3md232 | 228.6 | 229 | 0.4 | 40.0 | -1.9 | 0.68 |
| 1_3md233 | 229 | 229.3 | 0.3 | 43.0 | 2.9 | 0.88 |
| 1_3md234 | 229.3 | 230.1 | 0.8 | 48.8 | 5.8 | 2.9 |
| 1_3md235 | 230.1 | 230.9 | 0.8 | 42.5 | -6.3 | 1.82 |
| 1_3md236 | 230.9 | 231.4 | 0.5 | 39.8 | -2.7 | 1.31 |
| 1_3md237 | 231.4 | 232.6 | 1.2 | 36.0 | -3.8 | 0.84 |
| 1_3md238 | 232.6 | 232.8 | 0.2 | 38.6 | 2.6 | 0 |
| 1_3md239 | 232.8 | 233.1 | 0.3 | 41.9 | 3.2 | 1.04 |
| 1_3md240 | 233.1 | 233.3 | 0.2 | 40.4 | -1.4 | 0 |

| | | | | | | |
|---|---|---|---|---|---|---|
| 1_3md241 | 233.3 | 233.7 | 0.4 | 49.3 | 8.9 | 5.97 |
| 1_3md242 | 233.7 | 234.8 | 1.1 | 43.4 | -5.9 | 1.32 |
| 1_3md243 | 234.8 | 235.7 | 0.9 | 50.6 | 7.2 | 2.71 |
| 1_3md244 | 235.7 | 235.9 | 0.2 | 55.3 | 4.7 | 0 |
| 1_3md245 | 235.9 | 236.6 | 0.7 | 48.4 | -7.0 | 2.61 |
| | | | | | | |
| 2_1md1 | 0 | 0.5 | 0.5 | 49.5 | | 1.25 |
| 2_1md2 | 0.5 | 0.8 | 0.3 | 45.4 | -4.1 | 4.04 |
| 2_1md3 | 0.8 | 1.2 | 0.4 | 53.2 | 7.8 | 0.48 |
| 2_1md4 | 1.2 | 2 | 0.8 | 49.8 | -3.4 | 2.28 |
| 2_1md5 | 2 | 2.2 | 0.2 | 43.3 | -6.5 | 0 |
| 2_1md6 | 2.2 | 2.4 | 0.2 | 49.5 | 6.2 | 0 |
| 2_1md7 | 2.4 | 2.9 | 0.5 | 41.8 | -7.7 | 1.22 |
| 2_1md8 | 2.9 | 3.1 | 0.2 | 46.8 | 5.1 | 0 |
| 2_1md9 | 3.1 | 3.5 | 0.4 | 43.1 | -3.8 | 1.48 |
| 2_1md10 | 3.5 | 3.7 | 0.2 | 40.6 | -2.5 | 0 |
| 2_1md11 | 3.7 | 3.9 | 0.2 | 54.0 | 13.4 | 0 |
| 2_1md12 | 3.9 | 4.7 | 0.8 | 39.6 | -14.4 | 1.27 |
| 2_1md13 | 4.7 | 5.8 | 1.1 | 44.8 | 5.2 | 2.05 |
| 2_1md14 | 5.8 | 6 | 0.2 | 50.5 | 5.7 | 0 |
| 2_1md15 | 6 | 6.2 | 0.2 | 40.9 | -9.7 | 0 |
| 2_1md16 | 6.2 | 6.8 | 0.6 | 46.8 | 6.0 | 2.47 |
| 2_1md17 | 6.8 | 7.5 | 0.7 | 44.4 | -2.5 | 2.69 |
| 2_1md18 | 7.5 | 7.7 | 0.2 | 38.3 | -6.1 | 0 |
| 2_1md19 | 7.7 | 7.9 | 0.2 | 42.2 | 3.9 | 0 |
| 2_1md20 | 7.9 | 8.8 | 0.9 | 47.3 | 5.2 | 3.64 |
| 2_1md21 | 8.8 | 9.4 | 0.6 | 42.5 | -4.9 | 2.77 |
| 2_1md22 | 9.4 | 11.2 | 1.8 | 48.5 | 6.1 | 3.29 |
| 2_1md23 | 11.2 | 11.8 | 0.6 | 40.3 | -8.3 | 1.45 |
| 2_1md24 | 11.8 | 12 | 0.2 | 41.7 | 1.4 | 0 |
| 2_1md25 | 12 | 15.9 | 3.9 | 38.7 | -2.9 | 1.52 |
| 2_1md26 | 15.9 | 16.1 | 0.2 | 36.5 | -2.2 | 0 |
| 2_1md27 | 16.1 | 16.5 | 0.4 | 37.5 | 0.9 | 0.98 |
| 2_1md28 | 16.5 | 16.9 | 0.4 | 46.8 | 9.3 | 3.08 |
| 2_1md29 | 16.9 | 17.3 | 0.4 | 41.7 | -5.0 | 1.15 |
| 2_1md30 | 17.3 | 17.7 | 0.4 | 38.6 | -3.1 | 1.22 |
| 2_1md31 | 17.7 | 19.1 | 1.4 | 36.7 | -2.0 | 1.61 |
| 2_1md32 | 19.1 | 19.4 | 0.3 | 38.8 | 2.1 | 0.85 |
| 2_1md33 | 19.4 | 21.8 | 2.4 | 42.3 | 3.5 | 1.81 |
| 2_1md34 | 21.8 | 22.8 | 1 | 39.1 | -3.3 | 1.15 |
| 2_1md35 | 22.8 | 23.1 | 0.3 | 44.1 | 5.0 | 2.74 |
| 2_1md36 | 23.1 | 23.4 | 0.3 | 47.0 | 2.9 | 0.96 |
| 2_1md37 | 23.4 | 23.6 | 0.2 | 44.6 | -2.3 | 0 |
| 2_1md38 | 23.6 | 24.1 | 0.5 | 48.5 | 3.8 | 2.97 |
| 2_1md39 | 24.1 | 25.6 | 1.5 | 42.7 | -5.8 | 1.75 |
| 2_1md40 | 25.6 | 25.9 | 0.3 | 40.3 | -2.4 | 0.78 |
| 2_1md41 | 25.9 | 27 | 1.1 | 44.8 | 4.5 | 1.72 |
| 2_1md42 | 27 | 27.3 | 0.3 | 40.4 | -4.4 | 0.96 |
| 2_1md43 | 27.3 | 27.7 | 0.4 | 46.9 | 6.5 | 0.67 |
| 2_1md44 | 27.7 | 28.7 | 1 | 42.8 | -4.1 | 2 |
| 2_1md45 | 28.7 | 29.1 | 0.4 | 47.7 | 4.9 | 1.61 |
| 2_1md46 | 29.1 | 30.5 | 1.4 | 43.0 | -4.7 | 1.78 |
| 2_1md47 | 30.5 | 32 | 1.5 | 38.5 | -4.5 | 1.05 |
| 2_1md48 | 32 | 32.4 | 0.4 | 41.7 | 3.2 | 0.7 |
| 2_1md49 | 32.4 | 32.9 | 0.5 | 39.0 | -2.7 | 1.35 |
| 2_1md50 | 32.9 | 33.7 | 0.8 | 43.6 | 4.6 | 1.59 |

| | | | | | | |
|---|---|---|---|---|---|---|
| 2_1md51 | 33.7 | 35.7 | 2 | 39.6 | -4.0 | 1.17 |
| 2_1md52 | 35.7 | 36.2 | 0.5 | 42.5 | 2.9 | 2.32 |
| 2_1md53 | 36.2 | 37 | 0.8 | 39.6 | -2.9 | 0.9 |
| 2_1md54 | 37 | 37.2 | 0.2 | 42.2 | 2.6 | 0 |
| 2_1md55 | 37.2 | 37.5 | 0.3 | 40.1 | -2.2 | 1.04 |
| 2_1md56 | 37.5 | 37.9 | 0.4 | 46.8 | 6.7 | 1.86 |
| 2_1md57 | 37.9 | 38.1 | 0.2 | 44.5 | -2.3 | 0 |
| 2_1md58 | 38.1 | 41.8 | 3.7 | 37.9 | -6.6 | 1.1 |
| 2_1md59 | 41.8 | 42.2 | 0.4 | 35.7 | -2.2 | 1.02 |
| 2_1md60 | 42.2 | 44.1 | 1.9 | 37.9 | 2.2 | 0.9 |
| 2_1md61 | 44.1 | 44.3 | 0.2 | 36.6 | -1.4 | 0 |
| 2_1md62 | 44.3 | 44.5 | 0.2 | 38.7 | 2.1 | 0 |
| 2_1md63 | 44.5 | 44.8 | 0.3 | 36.6 | -2.0 | 0.36 |
| 2_1md64 | 44.8 | 45.4 | 0.6 | 37.8 | 1.2 | 0.87 |
| 2_1md65 | 45.4 | 47.8 | 2.4 | 35.6 | -2.2 | 0.83 |
| 2_1md66 | 47.8 | 48.2 | 0.4 | 37.4 | 1.7 | 0.25 |
| 2_1md67 | 48.2 | 52 | 3.8 | 35.2 | -2.2 | 1.29 |
| 2_1md68 | 52 | 52.2 | 0.2 | 42.9 | 7.8 | 0 |
| 2_1md69 | 52.2 | 53.9 | 1.7 | 38.7 | -4.2 | 1.34 |
| 2_1md70 | 53.9 | 54.1 | 0.2 | 36.7 | -2.0 | 0 |
| 2_1md71 | 54.1 | 54.3 | 0.2 | 38.2 | 1.6 | 0 |
| 2_1md72 | 54.3 | 54.9 | 0.6 | 36.7 | -1.6 | 0.69 |
| 2_1md73 | 54.9 | 55.1 | 0.2 | 37.7 | 1.0 | 0 |
| 2_1md74 | 55.1 | 55.8 | 0.7 | 36.2 | -1.4 | 0.54 |
| 2_1md75 | 55.8 | 58.2 | 2.4 | 38.1 | 1.9 | 0.88 |
| 2_1md76 | 58.2 | 58.4 | 0.2 | 35.8 | -2.4 | 0 |
| 2_1md77 | 58.4 | 59.2 | 0.8 | 38.6 | 2.8 | 1.18 |
| 2_1md78 | 59.2 | 59.4 | 0.2 | 36.1 | -2.4 | 0 |
| 2_1md79 | 59.4 | 60.5 | 1.1 | 38.6 | 2.5 | 1.25 |
| 2_1md80 | 60.5 | 61.5 | 1 | 36.9 | -1.8 | 0.84 |
| 2_1md81 | 61.5 | 62.1 | 0.6 | 38.1 | 1.3 | 0.83 |
| 2_1md82 | 62.1 | 62.9 | 0.8 | 36.0 | -2.1 | 1.18 |
| 2_1md83 | 62.9 | 63.1 | 0.2 | 37.3 | 1.3 | 0 |
| 2_1md84 | 63.1 | 63.8 | 0.7 | 36.5 | -0.8 | 0.32 |
| 2_1md85 | 63.8 | 64.1 | 0.3 | 37.9 | 1.4 | 1.17 |
| 2_1md86 | 64.1 | 64.6 | 0.5 | 34.8 | -3.1 | 1.17 |
| 2_1md87 | 64.6 | 65.2 | 0.6 | 37.7 | 2.9 | 1.41 |
| 2_1md88 | 65.2 | 65.5 | 0.3 | 36.7 | -1.0 | 0.29 |
| 2_1md89 | 65.5 | 66.7 | 1.2 | 37.6 | 0.9 | 0.9 |
| 2_1md90 | 66.7 | 67.2 | 0.5 | 36.3 | -1.3 | 0.37 |
| 2_1md91 | 67.2 | 68.7 | 1.5 | 38.2 | 1.9 | 0.52 |
| 2_1md92 | 68.7 | 69 | 0.3 | 34.3 | -3.8 | 0.48 |
| 2_1md93 | 69 | 70.5 | 1.5 | 37.6 | 3.3 | 0.52 |
| 2_1md94 | 70.5 | 71.3 | 0.8 | 36.2 | -1.4 | 0.76 |
| 2_1md95 | 71.3 | 71.8 | 0.5 | 38.5 | 2.3 | 0.75 |
| 2_1md96 | 71.8 | 72.1 | 0.3 | 35.2 | -3.4 | 0.61 |
| 2_1md97 | 72.1 | 72.4 | 0.3 | 39.0 | 3.9 | 0.67 |
| 2_1md98 | 72.4 | 72.8 | 0.4 | 36.3 | -2.7 | 1.82 |
| 2_1md99 | 72.8 | 73.9 | 1.1 | 38.4 | 2.1 | 1.47 |
| 2_1md100 | 73.9 | 74.1 | 0.2 | 35.2 | -3.2 | 0 |
| 2_1md101 | 74.1 | 74.4 | 0.3 | 41.6 | 6.4 | 2.3 |
| 2_1md102 | 74.4 | 74.6 | 0.2 | 35.9 | -5.7 | 0 |
| 2_1md103 | 74.6 | 74.8 | 0.2 | 39.5 | 3.6 | 0 |
| 2_1md104 | 74.8 | 75 | 0.2 | 35.3 | -4.2 | 0 |
| 2_1md105 | 75 | 79.6 | 4.6 | 38.3 | 3.1 | 1.22 |
| 2_1md106 | 79.6 | 79.9 | 0.3 | 36.2 | -2.1 | 0.36 |

| | | | | | | |
|---|---|---|---|---|---|---|
| 2_1md107 | 79.9 | 80.5 | 0.6 | 37.9 | 1.7 | 0.81 |
| 2_1md108 | 80.5 | 80.7 | 0.2 | 35.4 | -2.5 | 0 |
| 2_1md109 | 80.7 | 81.1 | 0.4 | 37.1 | 1.8 | 0.24 |
| 2_1md110 | 81.1 | 101.2 | 20.1 | 34.6 | -2.5 | 1.05 |
| 2_1md111 | 101.2 | 102 | 0.8 | 37.8 | 3.2 | 0.8 |
| 2_1md112 | 102 | 102.2 | 0.2 | 35.8 | -2.0 | 0 |
| 2_1md113 | 102.2 | 102.5 | 0.3 | 42.4 | 6.6 | 0.83 |
| 2_1md114 | 102.5 | 103.1 | 0.6 | 39.4 | -3.0 | 0.87 |
| 2_1md115 | 103.1 | 106.3 | 3.2 | 43.4 | 4.0 | 2.65 |
| 2_1md116 | 106.3 | 106.7 | 0.4 | 38.5 | -4.9 | 1.67 |
| 2_1md117 | 106.7 | 106.9 | 0.2 | 41.9 | 3.4 | 0 |
| 2_1md118 | 106.9 | 107.2 | 0.3 | 39.0 | -2.9 | 1.79 |
| 2_1md119 | 107.2 | 107.9 | 0.7 | 42.1 | 3.1 | 1.32 |
| 2_1md120 | 107.9 | 108.6 | 0.7 | 39.3 | -2.8 | 1.73 |
| 2_1md121 | 108.6 | 108.8 | 0.2 | 42.5 | 3.2 | 0 |
| 2_1md122 | 108.8 | 109.6 | 0.8 | 38.8 | -3.7 | 1.03 |
| 2_1md123 | 109.6 | 110.4 | 0.8 | 41.4 | 2.6 | 1.97 |
| 2_1md124 | 110.4 | 110.6 | 0.2 | 39.3 | -2.2 | 0 |
| 2_1md125 | 110.6 | 111.5 | 0.9 | 42.1 | 2.8 | 1.89 |
| 2_1md126 | 111.5 | 111.7 | 0.2 | 39.6 | -2.5 | 0 |
| 2_1md127 | 111.7 | 112.1 | 0.4 | 41.3 | 1.7 | 1.6 |
| 2_1md128 | 112.1 | 112.4 | 0.3 | 39.6 | -1.8 | 0.92 |
| 2_1md129 | 112.4 | 112.7 | 0.3 | 46.9 | 7.3 | 2.32 |
| 2_1md130 | 112.7 | 112.9 | 0.2 | 42.3 | -4.5 | 0 |
| 2_1md131 | 112.9 | 114.2 | 1.3 | 39.3 | -3.0 | 1.31 |
| 2_1md132 | 114.2 | 114.9 | 0.7 | 42.2 | 2.8 | 1.27 |
| 2_1md133 | 114.9 | 117.3 | 2.4 | 38.5 | -3.7 | 1.1 |
| 2_1md134 | 117.3 | 117.7 | 0.4 | 35.6 | -2.9 | 0.86 |
| 2_1md135 | 117.7 | 119.7 | 2 | 38.0 | 2.4 | 1.36 |
| 2_1md136 | 119.7 | 120.2 | 0.5 | 36.8 | -1.2 | 0.78 |
| 2_1md137 | 120.2 | 121.4 | 1.2 | 38.3 | 1.5 | 1.35 |
| 2_1md138 | 121.4 | 121.7 | 0.3 | 35.8 | -2.5 | 1.15 |
| 2_1md139 | 121.7 | 122.9 | 1.2 | 37.8 | 2.0 | 0.59 |
| 2_1md140 | 122.9 | 126.5 | 3.6 | 35.4 | -2.4 | 1.02 |
| 2_1md141 | 126.5 | 126.8 | 0.3 | 37.2 | 1.7 | 0.11 |
| 2_1md142 | 126.8 | 131.1 | 4.3 | 35.7 | -1.4 | 0.99 |
| 2_1md143 | 131.1 | 131.5 | 0.4 | 38.2 | 2.5 | 0.68 |
| 2_1md144 | 131.5 | 132.9 | 1.4 | 36.8 | -1.5 | 0.46 |
| 2_1md145 | 132.9 | 133.4 | 0.5 | 37.1 | 0.3 | 0.4 |
| 2_1md146 | 133.4 | 133.6 | 0.2 | 37.8 | 0.7 | 0 |
| 2_1md147 | 133.6 | 134.1 | 0.5 | 35.6 | -2.2 | 0.97 |
| 2_1md148 | 134.1 | 135.7 | 1.6 | 38.6 | 3.1 | 1.35 |
| 2_1md149 | 135.7 | 135.9 | 0.2 | 36.6 | -2.0 | 0 |
| 2_1md150 | 135.9 | 136.6 | 0.7 | 38.2 | 1.5 | 0.71 |
| 2_1md151 | 136.6 | 137 | 0.4 | 35.4 | -2.7 | 0.56 |
| 2_1md152 | 137 | 137.2 | 0.2 | 38.7 | 3.3 | 0 |
| 2_1md153 | 137.2 | 137.4 | 0.2 | 35.2 | -3.6 | 0 |
| 2_1md154 | 137.4 | 139.7 | 2.3 | 39.0 | 3.9 | 1.36 |
| 2_1md155 | 139.7 | 140.1 | 0.4 | 35.3 | -3.7 | 1.02 |
| 2_1md156 | 140.1 | 140.3 | 0.2 | 39.3 | 3.9 | 0 |
| 2_1md157 | 140.3 | 141.4 | 1.1 | 36.5 | -2.8 | 0.99 |
| 2_1md158 | 141.4 | 143.1 | 1.7 | 38.3 | 1.8 | 0.69 |
| 2_1md159 | 143.1 | 143.5 | 0.4 | 36.4 | -2.0 | 0.22 |
| 2_1md160 | 143.5 | 143.8 | 0.3 | 38.3 | 1.9 | 0.45 |
| 2_1md161 | 143.8 | 152.5 | 8.7 | 35.2 | -3.1 | 1.26 |
| 2_1md162 | 152.5 | 152.8 | 0.3 | 37.7 | 2.5 | 0.77 |

| | | | | | | |
|---|---|---|---|---|---|---|
| 2_1md163 | 152.8 | 153.5 | 0.7 | 36.6 | -1.1 | 0.62 |
| 2_1md164 | 153.5 | 153.9 | 0.4 | 37.5 | 0.9 | 0.84 |
| 2_1md165 | 153.9 | 154.2 | 0.3 | 35.4 | -2.1 | 0.75 |
| 2_1md166 | 154.2 | 154.5 | 0.3 | 37.5 | 2.1 | 0.39 |
| 2_1md167 | 154.5 | 154.9 | 0.4 | 35.7 | -1.8 | 0.94 |
| 2_1md168 | 154.9 | 156 | 1.1 | 38.0 | 2.3 | 0.87 |
| 2_1md169 | 156 | 156.2 | 0.2 | 35.7 | -2.3 | 0 |
| 2_1md170 | 156.2 | 156.4 | 0.2 | 37.4 | 1.7 | 0 |
| 2_1md171 | 156.4 | 158.8 | 2.4 | 36.3 | -1.1 | 0.71 |
| 2_1md172 | 158.8 | 159.1 | 0.3 | 37.6 | 1.4 | 0.15 |
| 2_1md173 | 159.1 | 160.4 | 1.3 | 36.5 | -1.2 | 0.42 |
| 2_1md174 | 160.4 | 163.6 | 3.2 | 37.8 | 1.3 | 0.76 |
| 2_1md175 | 163.6 | 163.8 | 0.2 | 36.6 | -1.2 | 0 |
| 2_1md176 | 163.8 | 164.8 | 1 | 38.0 | 1.4 | 0.62 |
| 2_1md177 | 164.8 | 165 | 0.2 | 36.5 | -1.5 | 0 |
| 2_1md178 | 165 | 165.5 | 0.5 | 38.8 | 2.3 | 1.24 |
| 2_1md179 | 165.5 | 166.1 | 0.6 | 41.5 | 2.7 | 1.3 |
| 2_1md180 | 166.1 | 166.4 | 0.3 | 38.9 | -2.6 | 1.51 |
| 2_1md181 | 166.4 | 166.9 | 0.5 | 36.3 | -2.6 | 0.41 |
| 2_1md182 | 166.9 | 167.1 | 0.2 | 39.5 | 3.2 | 0 |
| 2_1md183 | 167.1 | 168.2 | 1.1 | 36.2 | -3.2 | 0.56 |
| 2_1md184 | 168.2 | 168.5 | 0.3 | 38.8 | 2.5 | 1.31 |
| 2_1md185 | 168.5 | 168.7 | 0.2 | 42.3 | 3.5 | 0 |
| 2_1md186 | 168.7 | 169.5 | 0.8 | 38.8 | -3.5 | 1.42 |
| 2_1md187 | 169.5 | 170.7 | 1.2 | 35.7 | -3.1 | 0.92 |
| 2_1md188 | 170.7 | 170.9 | 0.2 | 39.0 | 3.3 | 0 |
| 2_1md189 | 170.9 | 171.2 | 0.3 | 43.8 | 4.7 | 1.84 |
| 2_1md190 | 171.2 | 171.7 | 0.5 | 35.7 | -8.1 | 0.99 |
| 2_1md191 | 171.7 | 172.2 | 0.5 | 38.1 | 2.4 | 0.82 |
| 2_1md192 | 172.2 | 172.4 | 0.2 | 35.2 | -2.8 | 0 |
| 2_1md193 | 172.4 | 173.3 | 0.9 | 38.4 | 3.2 | 2.16 |
| 2_1md194 | 173.3 | 173.5 | 0.2 | 36.2 | -2.2 | 0 |
| 2_1md195 | 173.5 | 174.9 | 1.4 | 39.7 | 3.4 | 2.17 |
| 2_1md196 | 174.9 | 175.8 | 0.9 | 41.6 | 1.9 | 0.82 |
| 2_1md197 | 175.8 | 177.1 | 1.3 | 39.0 | -2.6 | 0.83 |
| 2_1md198 | 177.1 | 177.8 | 0.7 | 36.6 | -2.4 | 0.35 |
| 2_1md199 | 177.8 | 178.6 | 0.8 | 38.5 | 1.9 | 1.32 |
| 2_1md200 | 178.6 | 178.8 | 0.2 | 35.9 | -2.6 | 0 |
| 2_1md201 | 178.8 | 179.2 | 0.4 | 37.3 | 1.4 | 0.36 |
| 2_1md202 | 179.2 | 183.3 | 4.1 | 35.4 | -1.8 | 0.66 |
| 2_1md203 | 183.3 | 184.2 | 0.9 | 38.0 | 2.6 | 0.72 |
| 2_1md204 | 184.2 | 184.4 | 0.2 | 36.3 | -1.7 | 0 |
| 2_1md205 | 184.4 | 185.1 | 0.7 | 40.1 | 3.8 | 1.07 |
| 2_1md206 | 185.1 | 186 | 0.9 | 41.2 | 1.1 | 1.68 |
| 2_1md207 | 186 | 187.9 | 1.9 | 38.4 | -2.8 | 1.17 |
| 2_1md208 | 187.9 | 188.3 | 0.4 | 42.9 | 4.5 | 0.36 |
| 2_1md209 | 188.3 | 189.1 | 0.8 | 40.5 | -2.4 | 1.51 |
| 2_1md210 | 189.1 | 190.2 | 1.1 | 43.4 | 2.9 | 3.65 |
| 2_1md211 | 190.2 | 190.4 | 0.2 | 35.8 | -7.6 | 0 |
| 2_1md212 | 190.4 | 190.8 | 0.4 | 39.0 | 3.2 | 1.57 |
| 2_1md213 | 190.8 | 192.1 | 1.3 | 43.6 | 4.6 | 1.61 |
| 2_1md214 | 192.1 | 192.3 | 0.2 | 38.2 | -5.4 | 0 |
| 2_1md215 | 192.3 | 192.5 | 0.2 | 41.4 | 3.3 | 0 |
| 2_1md216 | 192.5 | 192.7 | 0.2 | 40.4 | -1.0 | 0 |
| 2_1md217 | 192.7 | 192.9 | 0.2 | 42.5 | 2.0 | 0 |
| 2_1md218 | 192.9 | 193.2 | 0.3 | 40.1 | -2.4 | 0.66 |

| | | | | | | |
|---|---|---|---|---|---|---|
| 2_1md219 | 193.2 | 193.8 | 0.6 | 41.9 | 1.8 | 2.17 |
| 2_1md220 | 193.8 | 194.2 | 0.4 | 39.0 | -2.9 | 1.59 |
| 2_1md221 | 194.2 | 195.3 | 1.1 | 42.5 | 3.5 | 1.37 |
| 2_1md222 | 195.3 | 195.8 | 0.5 | 38.3 | -4.3 | 0.84 |
| 2_1md223 | 195.8 | 196 | 0.2 | 36.6 | -1.7 | 0 |
| 2_1md224 | 196 | 196.8 | 0.8 | 37.8 | 1.2 | 0.59 |
| | | | | | | |
| 2_2md1 | 0 | 1.1 | 1.1 | 41.7 | | 1.93 |
| 2_2md2 | 1.1 | 1.3 | 0.2 | 39.8 | -1.9 | 0 |
| 2_2md3 | 1.3 | 1.6 | 0.3 | 45.8 | 6.0 | 2.48 |
| 2_2md4 | 1.6 | 1.8 | 0.2 | 37.4 | -8.4 | 0 |
| 2_2md5 | 1.8 | 2 | 0.2 | 42.6 | 5.3 | 0 |
| 2_2md6 | 2 | 2.2 | 0.2 | 38.2 | -4.4 | 0 |
| 2_2md7 | 2.2 | 2.8 | 0.6 | 44.0 | 5.8 | 2.33 |
| 2_2md8 | 2.8 | 3.1 | 0.3 | 38.6 | -5.4 | 1.97 |
| 2_2md9 | 3.1 | 3.9 | 0.8 | 42.4 | 3.8 | 1.35 |
| 2_2md10 | 3.9 | 4.3 | 0.4 | 39.7 | -2.7 | 0.28 |
| 2_2md11 | 4.3 | 4.7 | 0.4 | 41.9 | 2.2 | 2.79 |
| 2_2md12 | 4.7 | 5.7 | 1 | 39.3 | -2.6 | 1.48 |
| 2_2md13 | 5.7 | 5.9 | 0.2 | 42.7 | 3.4 | 0 |
| 2_2md14 | 5.9 | 6.3 | 0.4 | 40.4 | -2.3 | 0.63 |
| 2_2md15 | 6.3 | 7.1 | 0.8 | 43.4 | 3.0 | 1.82 |
| 2_2md16 | 7.1 | 7.3 | 0.2 | 41.0 | -2.4 | 0 |
| 2_2md17 | 7.3 | 8.7 | 1.4 | 41.7 | 0.7 | 2.66 |
| 2_2md18 | 8.7 | 8.9 | 0.2 | 47.0 | 5.3 | 0 |
| 2_2md19 | 8.9 | 9.8 | 0.9 | 40.0 | -7.0 | 1.49 |
| 2_2md20 | 9.8 | 10 | 0.2 | 35.6 | -4.4 | 0 |
| 2_2md21 | 10 | 10.9 | 0.9 | 39.8 | 4.2 | 1.34 |
| 2_2md22 | 10.9 | 11.2 | 0.3 | 36.1 | -3.7 | 0.64 |
| 2_2md23 | 11.2 | 11.5 | 0.3 | 38.6 | 2.5 | 1.37 |
| 2_2md24 | 11.5 | 12.4 | 0.9 | 35.7 | -2.9 | 0.97 |
| 2_2md25 | 12.4 | 12.6 | 0.2 | 39.4 | 3.7 | 0 |
| 2_2md26 | 12.6 | 13.7 | 1.1 | 36.4 | -3.0 | 0.77 |
| 2_2md27 | 13.7 | 14.4 | 0.7 | 39.6 | 3.2 | 1.39 |
| 2_2md28 | 14.4 | 14.6 | 0.2 | 42.3 | 2.6 | 0 |
| 2_2md29 | 14.6 | 15.1 | 0.5 | 39.2 | -3.1 | 0.95 |
| 2_2md30 | 15.1 | 15.3 | 0.2 | 41.4 | 2.2 | 0 |
| 2_2md31 | 15.3 | 16.3 | 1 | 39.7 | -1.7 | 1.66 |
| 2_2md32 | 16.3 | 17 | 0.7 | 42.7 | 3.0 | 1.59 |
| 2_2md33 | 17 | 17.2 | 0.2 | 36.7 | -5.9 | 0 |
| 2_2md34 | 17.2 | 17.8 | 0.6 | 39.8 | 3.1 | 1.23 |
| 2_2md35 | 17.8 | 18.2 | 0.4 | 42.6 | 2.8 | 1.52 |
| 2_2md36 | 18.2 | 18.5 | 0.3 | 39.6 | -3.0 | 0.31 |
| 2_2md37 | 18.5 | 18.7 | 0.2 | 41.3 | 1.7 | 0 |
| 2_2md38 | 18.7 | 20.4 | 1.7 | 39.8 | -1.6 | 0.95 |
| 2_2md39 | 20.4 | 20.6 | 0.2 | 42.2 | 2.4 | 0 |
| 2_2md40 | 20.6 | 22.3 | 1.7 | 38.5 | -3.7 | 0.95 |
| 2_2md41 | 22.3 | 28.4 | 6.1 | 35.3 | -3.2 | 0.67 |
| 2_2md42 | 28.4 | 28.8 | 0.4 | 37.1 | 1.8 | 0.43 |
| 2_2md43 | 28.8 | 29 | 0.2 | 36.2 | -0.9 | 0 |
| 2_2md44 | 29 | 29.3 | 0.3 | 37.3 | 1.0 | 0.26 |
| 2_2md45 | 29.3 | 31 | 1.7 | 35.5 | -1.8 | 1.01 |
| 2_2md46 | 31 | 31.5 | 0.5 | 37.8 | 2.3 | 1.01 |
| 2_2md47 | 31.5 | 31.9 | 0.4 | 35.2 | -2.6 | 1.01 |
| 2_2md48 | 31.9 | 32.4 | 0.5 | 37.3 | 2.1 | 1.19 |
| 2_2md49 | 32.4 | 32.8 | 0.4 | 36.1 | -1.1 | 1.22 |

| | | | | | | |
|---|---|---|---|---|---|---|
| 2_2md50 | 32.8 | 33 | 0.2 | 38.4 | 2.3 | 0 |
| 2_2md51 | 33 | 33.4 | 0.4 | 36.2 | -2.2 | 0.66 |
| 2_2md52 | 33.4 | 34 | 0.6 | 37.9 | 1.7 | 1.01 |
| 2_2md53 | 34 | 34.3 | 0.3 | 36.5 | -1.5 | 0.05 |
| 2_2md54 | 34.3 | 36.2 | 1.9 | 39.7 | 3.3 | 1.43 |
| 2_2md55 | 36.2 | 36.4 | 0.2 | 42.3 | 2.5 | 0 |
| 2_2md56 | 36.4 | 37.6 | 1.2 | 39.2 | -3.0 | 1.66 |
| 2_2md57 | 37.6 | 38.3 | 0.7 | 36.1 | -3.1 | 0.88 |
| 2_2md58 | 38.3 | 39.2 | 0.9 | 38.2 | 2.1 | 1.45 |
| 2_2md59 | 39.2 | 39.4 | 0.2 | 36.4 | -1.8 | 0 |
| 2_2md60 | 39.4 | 40.3 | 0.9 | 38.1 | 1.8 | 1.03 |
| 2_2md61 | 40.3 | 41.1 | 0.8 | 36.3 | -1.9 | 0.48 |
| 2_2md62 | 41.1 | 42 | 0.9 | 38.2 | 1.9 | 1.16 |
| 2_2md63 | 42 | 42.4 | 0.4 | 36.5 | -1.8 | 0.99 |
| 2_2md64 | 42.4 | 43.2 | 0.8 | 39.0 | 2.5 | 0.75 |
| 2_2md65 | 43.2 | 43.5 | 0.3 | 36.0 | -3.0 | 1.25 |
| 2_2md66 | 43.5 | 46.8 | 3.3 | 37.9 | 1.9 | 0.97 |
| 2_2md67 | 46.8 | 47.4 | 0.6 | 36.1 | -1.7 | 0.61 |
| 2_2md68 | 47.4 | 47.9 | 0.5 | 37.8 | 1.6 | 0.37 |
| 2_2md69 | 47.9 | 48.2 | 0.3 | 36.5 | -1.3 | 0.24 |
| 2_2md70 | 48.2 | 48.4 | 0.2 | 37.4 | 0.9 | 0 |
| 2_2md71 | 48.4 | 49 | 0.6 | 35.9 | -1.5 | 0.6 |
| 2_2md72 | 49 | 49.2 | 0.2 | 37.7 | 1.8 | 0 |
| 2_2md73 | 49.2 | 49.7 | 0.5 | 36.4 | -1.3 | 0.34 |
| 2_2md74 | 49.7 | 50 | 0.3 | 37.5 | 1.1 | 0.55 |
| 2_2md75 | 50 | 53 | 3 | 46.4 | 8.9 | 0 |
| 2_2md76 | 53 | 53.2 | 0.2 | 36.3 | -10.0 | 0 |
| 2_2md77 | 53.2 | 53.6 | 0.4 | 37.7 | 1.3 | 0.86 |
| 2_2md78 | 53.6 | 53.9 | 0.3 | 35.7 | -2.0 | 0.66 |
| 2_2md79 | 53.9 | 54.1 | 0.2 | 37.2 | 1.5 | 0 |
| 2_2md80 | 54.1 | 54.3 | 0.2 | 36.1 | -1.1 | 0 |
| 2_2md81 | 54.3 | 55.7 | 1.4 | 37.8 | 1.6 | 0.91 |
| 2_2md82 | 55.7 | 56.2 | 0.5 | 36.0 | -1.7 | 0.6 |
| 2_2md83 | 56.2 | 56.8 | 0.6 | 37.8 | 1.8 | 0.6 |
| 2_2md84 | 56.8 | 57.1 | 0.3 | 36.1 | -1.7 | 0.29 |
| 2_2md85 | 57.1 | 57.6 | 0.5 | 39.6 | 3.5 | 1.52 |
| 2_2md86 | 57.6 | 57.9 | 0.3 | 42.2 | 2.6 | 0.68 |
| 2_2md87 | 57.9 | 58.3 | 0.4 | 39.8 | -2.3 | 1.72 |
| 2_2md88 | 58.3 | 59 | 0.7 | 42.2 | 2.3 | 1.41 |
| 2_2md89 | 59 | 59.2 | 0.2 | 40.1 | -2.1 | 0 |
| 2_2md90 | 59.2 | 59.6 | 0.4 | 41.9 | 1.8 | 0.48 |
| 2_2md91 | 59.6 | 60.4 | 0.8 | 39.9 | -2.1 | 0.93 |
| 2_2md92 | 60.4 | 60.6 | 0.2 | 42.9 | 3.0 | 0 |
| 2_2md93 | 60.6 | 62.2 | 1.6 | 38.8 | -4.0 | 1.23 |
| 2_2md94 | 62.2 | 62.9 | 0.7 | 36.3 | -2.5 | 0.45 |
| 2_2md95 | 62.9 | 63.4 | 0.5 | 38.0 | 1.7 | 2.23 |
| 2_2md96 | 63.4 | 64.7 | 1.3 | 36.2 | -1.7 | 0.77 |
| 2_2md97 | 64.7 | 65.3 | 0.6 | 37.9 | 1.7 | 0.91 |
| 2_2md98 | 65.3 | 67.2 | 1.9 | 36.4 | -1.6 | 0.75 |
| 2_2md99 | 67.2 | 67.4 | 0.2 | 37.9 | 1.6 | 0 |
| 2_2md100 | 67.4 | 69.4 | 2 | 36.1 | -1.8 | 0.79 |
| 2_2md101 | 69.4 | 70.1 | 0.7 | 38.9 | 2.8 | 0.58 |
| 2_2md102 | 70.1 | 70.5 | 0.4 | 41.9 | 3.0 | 1.04 |
| 2_2md103 | 70.5 | 71 | 0.5 | 39.9 | -2.0 | 0.95 |
| 2_2md104 | 71 | 71.8 | 0.8 | 42.7 | 2.8 | 0.97 |
| 2_2md105 | 71.8 | 73.9 | 2.1 | 39.3 | -3.4 | 1.27 |

| | | | | | | |
|---|---|---|---|---|---|---|
| 2_2md106 | 73.9 | 75.1 | 1.2 | 41.9 | 2.6 | 2.04 |
| 2_2md107 | 75.1 | 75.6 | 0.5 | 40.3 | -1.6 | 0.46 |
| 2_2md108 | 75.6 | 76 | 0.4 | 42.3 | 2.1 | 0.57 |
| 2_2md109 | 76 | 77.2 | 1.2 | 39.2 | -3.1 | 2.09 |
| 2_2md110 | 77.2 | 77.8 | 0.6 | 42.4 | 3.1 | 1.76 |
| 2_2md111 | 77.8 | 78 | 0.2 | 36.1 | -6.3 | 0 |
| 2_2md112 | 78 | 79.3 | 1.3 | 39.2 | 3.2 | 2.17 |
| 2_2md113 | 79.3 | 80 | 0.7 | 42.0 | 2.7 | 1.7 |
| 2_2md114 | 80 | 80.5 | 0.5 | 39.2 | -2.8 | 1.03 |
| 2_2md115 | 80.5 | 81 | 0.5 | 41.7 | 2.6 | 0.55 |
| 2_2md116 | 81 | 81.2 | 0.2 | 40.2 | -1.6 | 0 |
| 2_2md117 | 81.2 | 82.1 | 0.9 | 36.6 | -3.5 | 2.4 |
| 2_2md118 | 82.1 | 82.6 | 0.5 | 37.5 | 0.8 | 0.4 |
| 2_2md119 | 82.6 | 83.1 | 0.5 | 35.8 | -1.6 | 0.55 |
| 2_2md120 | 83.1 | 83.8 | 0.7 | 39.9 | 4.1 | 1.39 |
| 2_2md121 | 83.8 | 84.3 | 0.5 | 35.8 | -4.1 | 0.89 |
| 2_2md122 | 84.3 | 86.1 | 1.8 | 38.6 | 2.7 | 1.08 |
| 2_2md123 | 86.1 | 86.4 | 0.3 | 36.3 | -2.3 | 0.86 |
| 2_2md124 | 86.4 | 87.4 | 1 | 39.1 | 2.8 | 1.07 |
| 2_2md125 | 87.4 | 88 | 0.6 | 42.2 | 3.2 | 0.59 |
| 2_2md126 | 88 | 88.3 | 0.3 | 38.8 | -3.4 | 1.9 |
| 2_2md127 | 88.3 | 88.6 | 0.3 | 41.8 | 3.0 | 0.98 |
| 2_2md128 | 88.6 | 89.9 | 1.3 | 39.3 | -2.5 | 1.23 |
| 2_2md129 | 89.9 | 90.1 | 0.2 | 35.5 | -3.8 | 0 |
| 2_2md130 | 90.1 | 90.3 | 0.2 | 39.2 | 3.7 | 0 |
| 2_2md131 | 90.3 | 91.1 | 0.8 | 42.9 | 3.7 | 1.64 |
| 2_2md132 | 91.1 | 91.4 | 0.3 | 40.7 | -2.2 | 0.07 |
| 2_2md133 | 91.4 | 91.6 | 0.2 | 43.3 | 2.6 | 0 |
| 2_2md134 | 91.6 | 92 | 0.4 | 40.5 | -2.8 | 0.95 |
| 2_2md135 | 92 | 92.6 | 0.6 | 42.4 | 1.9 | 2.31 |
| 2_2md136 | 92.6 | 93.3 | 0.7 | 39.6 | -2.8 | 0.93 |
| 2_2md137 | 93.3 | 93.9 | 0.6 | 34.6 | -5.0 | 0.67 |
| 2_2md138 | 93.9 | 95.5 | 1.6 | 37.8 | 3.2 | 0.99 |
| 2_2md139 | 95.5 | 96.1 | 0.6 | 36.6 | -1.3 | 0.68 |
| 2_2md140 | 96.1 | 96.4 | 0.3 | 37.6 | 1.0 | 0.41 |
| 2_2md141 | 96.4 | 96.6 | 0.2 | 36.7 | -0.9 | 0 |
| 2_2md142 | 96.6 | 97 | 0.4 | 37.5 | 0.8 | 0.63 |
| 2_2md143 | 97 | 103.4 | 6.4 | 35.6 | -1.9 | 0.85 |
| 2_2md144 | 103.4 | 105.8 | 2.4 | 38.1 | 2.5 | 1.01 |
| 2_2md145 | 105.8 | 106 | 0.2 | 36.3 | -1.9 | 0 |
| 2_2md146 | 106 | 106.3 | 0.3 | 37.3 | 1.1 | 0.47 |
| 2_2md147 | 106.3 | 125.3 | 19 | 34.9 | -2.4 | 1.09 |
| 2_2md148 | 125.3 | 125.5 | 0.2 | 37.6 | 2.7 | 0 |
| 2_2md149 | 125.5 | 128.4 | 2.9 | 35.5 | -2.1 | 0.94 |
| 2_2md150 | 128.4 | 128.9 | 0.5 | 37.6 | 2.1 | 0.45 |
| 2_2md151 | 128.9 | 136.6 | 7.7 | 35.3 | -2.3 | 0.91 |
| 2_2md152 | 136.6 | 136.8 | 0.2 | 37.7 | 2.4 | 0 |
| 2_2md153 | 136.8 | 137.4 | 0.6 | 36.2 | -1.5 | 1.06 |
| 2_2md154 | 137.4 | 137.7 | 0.3 | 37.5 | 1.3 | 0.33 |
| 2_2md155 | 137.7 | 144.7 | 7 | 35.8 | -1.7 | 1 |
| 2_2md156 | 144.7 | 145.2 | 0.5 | 37.9 | 2.1 | 1.16 |
| 2_2md157 | 145.2 | 145.4 | 0.2 | 35.9 | -2.0 | 0 |
| 2_2md158 | 145.4 | 145.6 | 0.2 | 37.5 | 1.6 | 0 |
| 2_2md159 | 145.6 | 146.6 | 1 | 36.3 | -1.2 | 0.43 |
| 2_2md160 | 146.6 | 147 | 0.4 | 37.4 | 1.0 | 0.28 |
| 2_2md161 | 147 | 147.6 | 0.6 | 35.9 | -1.4 | 0.9 |

| | | | | | | |
|---|---|---|---|---|---|---|
| 2_2md162 | 147.6 | 148.1 | 0.5 | 37.5 | 1.6 | 0.69 |
| 2_2md163 | 148.1 | 149.7 | 1.6 | 36.3 | -1.2 | 1.05 |
| 2_2md164 | 149.7 | 150.4 | 0.7 | 37.2 | 0.9 | 0.63 |
| 2_2md165 | 150.4 | 150.7 | 0.3 | 36.4 | -0.8 | 0.14 |
| 2_2md166 | 150.7 | 151.4 | 0.7 | 38.2 | 1.8 | 0.8 |
| 2_2md167 | 151.4 | 164.2 | 12.8 | 35.0 | -3.2 | 1.11 |
| 2_2md168 | 164.2 | 164.5 | 0.3 | 37.8 | 2.8 | 0.26 |
| 2_2md169 | 164.5 | 173.2 | 8.7 | 35.1 | -2.7 | 1.09 |
| 2_2md170 | 173.2 | 174.1 | 0.9 | 37.9 | 2.8 | 0.7 |
| 2_2md171 | 174.1 | 174.4 | 0.3 | 36.1 | -1.9 | 0.94 |
| 2_2md172 | 174.4 | 175.3 | 0.9 | 38.5 | 2.4 | 0.99 |
| 2_2md173 | 175.3 | 175.7 | 0.4 | 36.6 | -2.0 | 0.48 |
| 2_2md174 | 175.7 | 176.1 | 0.4 | 38.4 | 1.8 | 0.95 |
| 2_2md175 | 176.1 | 176.9 | 0.8 | 37.0 | -1.4 | 0.96 |
| 2_2md176 | 176.9 | 177.5 | 0.6 | 39.0 | 2.0 | 1.4 |
| 2_2md177 | 177.5 | 177.9 | 0.4 | 35.8 | -3.1 | 0.79 |
| 2_2md178 | 177.9 | 178.3 | 0.4 | 39.0 | 3.2 | 1.48 |
| 2_2md179 | 178.3 | 178.9 | 0.6 | 36.6 | -2.5 | 1.22 |
| 2_2md180 | 178.9 | 179.8 | 0.9 | 37.5 | 1.0 | 1.01 |
| 2_2md181 | 179.8 | 180.1 | 0.3 | 36.1 | -1.4 | 0.57 |
| 2_2md182 | 180.1 | 180.3 | 0.2 | 37.9 | 1.8 | 0 |
| 2_2md183 | 180.3 | 180.8 | 0.5 | 35.7 | -2.1 | 0.79 |
| 2_2md184 | 180.8 | 181.6 | 0.8 | 38.7 | 2.9 | 1.21 |
| 2_2md185 | 181.6 | 184 | 2.4 | 35.8 | -2.9 | 0.82 |
| 2_2md186 | 184 | 184.7 | 0.7 | 37.3 | 1.5 | 0.47 |
| 2_2md187 | 184.7 | 190.1 | 5.4 | 35.6 | -1.7 | 0.91 |
| 2_2md188 | 190.1 | 190.3 | 0.2 | 37.1 | 1.5 | 0 |
| 2_2md189 | 190.3 | 196.8 | 6.5 | 35.4 | -1.7 | 1.1 |

| | | | | | | |
|---|---|---|---|---|---|---|
| 2_3md1 | 0 | 3.3 | 3.3 | 34.7 | | 1.15 |
| 2_3md2 | 3.3 | 3.5 | 0.2 | 37.4 | 2.7 | 0 |
| 2_3md3 | 3.5 | 11.7 | 8.2 | 35.5 | -1.9 | 0.94 |
| 2_3md4 | 11.7 | 11.9 | 0.2 | 37.1 | 1.6 | 0 |
| 2_3md5 | 11.9 | 16.3 | 4.4 | 35.6 | -1.5 | 1.05 |
| 2_3md6 | 16.3 | 16.5 | 0.2 | 37.2 | 1.6 | 0 |
| 2_3md7 | 16.5 | 19.8 | 3.3 | 36.2 | -0.9 | 0.89 |
| 2_3md8 | 19.8 | 20 | 0.2 | 37.6 | 1.4 | 0 |
| 2_3md9 | 20 | 20.2 | 0.2 | 36.8 | -0.9 | 0 |
| 2_3md10 | 20.2 | 20.6 | 0.4 | 38.0 | 1.3 | 0.39 |
| 2_3md11 | 20.6 | 21.8 | 1.2 | 36.2 | -1.8 | 0.78 |
| 2_3md12 | 21.8 | 22.4 | 0.6 | 37.5 | 1.3 | 0.57 |
| 2_3md13 | 22.4 | 26.9 | 4.5 | 34.4 | -3.1 | 0.89 |
| 2_3md14 | 26.9 | 27.1 | 0.2 | 37.5 | 3.1 | 0 |
| 2_3md15 | 27.1 | 36.4 | 9.3 | 35.8 | -1.7 | 1.09 |
| 2_3md16 | 36.4 | 36.6 | 0.2 | 37.7 | 1.9 | 0 |
| 2_3md17 | 36.6 | 37.2 | 0.6 | 36.4 | -1.3 | 0.32 |
| 2_3md18 | 37.2 | 37.7 | 0.5 | 37.3 | 0.9 | 0.54 |
| 2_3md19 | 37.7 | 37.9 | 0.2 | 37.9 | 0.5 | 0 |
| 2_3md20 | 37.9 | 41.9 | 4 | 35.1 | -2.8 | 0.91 |
| 2_3md21 | 41.9 | 42.1 | 0.2 | 37.6 | 2.5 | 0 |
| 2_3md22 | 42.1 | 46.1 | 4 | 36.1 | -1.5 | 1.23 |
| 2_3md23 | 46.1 | 46.6 | 0.5 | 38.5 | 2.3 | 0.87 |
| 2_3md24 | 46.6 | 48.2 | 1.6 | 36.7 | -1.8 | 1.06 |
| 2_3md25 | 48.2 | 49.3 | 1.1 | 37.6 | 0.9 | 1.04 |
| 2_3md26 | 49.3 | 53.1 | 3.8 | 35.1 | -2.5 | 1.28 |
| 2_3md27 | 53.1 | 53.3 | 0.2 | 37.5 | 2.4 | 0 |

| | | | | | | |
|---|---|---|---|---|---|---|
| 2_3md28 | 53.3 | 56.8 | 3.5 | 35.4 | -2.1 | 0.76 |
| 2_3md29 | 56.8 | 57 | 0.2 | 37.4 | 2.0 | 0 |
| 2_3md30 | 57 | 58.4 | 1.4 | 35.9 | -1.5 | 0.66 |
| 2_3md31 | 58.4 | 58.8 | 0.4 | 37.6 | 1.7 | 0.29 |
| 2_3md32 | 58.8 | 59.2 | 0.4 | 35.9 | -1.7 | 1.68 |
| 2_3md33 | 59.2 | 59.6 | 0.4 | 37.5 | 1.6 | 0.45 |
| 2_3md34 | 59.6 | 62 | 2.4 | 35.5 | -2.0 | 0.76 |
| 2_3md35 | 62 | 63.4 | 1.4 | 37.9 | 2.4 | 0.6 |
| 2_3md36 | 63.4 | 64.1 | 0.7 | 42.0 | 4.1 | 1.69 |
| 2_3md37 | 64.1 | 64.7 | 0.6 | 37.0 | -5.0 | 0.97 |
| 2_3md38 | 64.7 | 65.3 | 0.6 | 40.0 | 3.0 | 1.1 |
| 2_3md39 | 65.3 | 65.5 | 0.2 | 41.2 | 1.2 | 0 |
| 2_3md40 | 65.5 | 65.9 | 0.4 | 39.0 | -2.2 | 1.01 |
| 2_3md41 | 65.9 | 66.1 | 0.2 | 36.0 | -3.0 | 0 |
| 2_3md42 | 66.1 | 66.8 | 0.7 | 38.5 | 2.5 | 0.78 |
| 2_3md43 | 66.8 | 67.3 | 0.5 | 36.6 | -1.9 | 0.73 |
| 2_3md44 | 67.3 | 67.7 | 0.4 | 36.8 | 0.1 | 0.75 |
| 2_3md45 | 67.7 | 74.6 | 6.9 | 35.6 | -1.2 | 1.18 |
| 2_3md46 | 74.6 | 75 | 0.4 | 37.5 | 1.9 | 0.49 |
| 2_3md47 | 75 | 83.5 | 8.5 | 34.9 | -2.6 | 1.07 |
| 2_3md48 | 83.5 | 84.8 | 1.3 | 37.8 | 2.9 | 1.2 |
| 2_3md49 | 84.8 | 85.5 | 0.7 | 42.7 | 4.8 | 2.81 |
| 2_3md50 | 85.5 | 85.9 | 0.4 | 40.2 | -2.4 | 0.56 |
| 2_3md51 | 85.9 | 86.7 | 0.8 | 41.9 | 1.6 | 1.49 |
| 2_3md52 | 86.7 | 87.2 | 0.5 | 39.6 | -2.2 | 1.08 |
| 2_3md53 | 87.2 | 87.4 | 0.2 | 36.7 | -3.0 | 0 |
| 2_3md54 | 87.4 | 88.2 | 0.8 | 39.2 | 2.5 | 1.99 |
| 2_3md55 | 88.2 | 88.6 | 0.4 | 42.0 | 2.8 | 1.14 |
| 2_3md56 | 88.6 | 90.6 | 2 | 39.3 | -2.7 | 1.83 |
| 2_3md57 | 90.6 | 90.8 | 0.2 | 35.9 | -3.4 | 0 |
| 2_3md58 | 90.8 | 91.8 | 1 | 39.1 | 3.2 | 1.67 |
| 2_3md59 | 91.8 | 92 | 0.2 | 35.6 | -3.5 | 0 |
| 2_3md60 | 92 | 92.4 | 0.4 | 38.2 | 2.6 | 0.76 |
| 2_3md61 | 92.4 | 92.6 | 0.2 | 34.6 | -3.7 | 0 |
| 2_3md62 | 92.6 | 95.9 | 3.3 | 39.1 | 4.6 | 1.11 |
| 2_3md63 | 95.9 | 96.1 | 0.2 | 36.6 | -2.6 | 0 |
| 2_3md64 | 96.1 | 96.6 | 0.5 | 38.2 | 1.6 | 0.94 |
| 2_3md65 | 96.6 | 97.7 | 1.1 | 36.4 | -1.8 | 0.35 |
| 2_3md66 | 97.7 | 100.8 | 3.1 | 39.2 | 2.7 | 1.42 |
| 2_3md67 | 100.8 | 101.2 | 0.4 | 42.7 | 3.6 | 2.17 |
| 2_3md68 | 101.2 | 102.8 | 1.6 | 39.8 | -2.9 | 1.9 |
| 2_3md69 | 102.8 | 103.1 | 0.3 | 43.6 | 3.8 | 0.98 |
| 2_3md70 | 103.1 | 103.5 | 0.4 | 38.9 | -4.8 | 1.73 |
| 2_3md71 | 103.5 | 103.7 | 0.2 | 42.8 | 3.9 | 0 |
| 2_3md72 | 103.7 | 106.8 | 3.1 | 39.8 | -3.0 | 1.33 |
| 2_3md73 | 106.8 | 107.1 | 0.3 | 41.9 | 2.1 | 0.15 |
| 2_3md74 | 107.1 | 110.7 | 3.6 | 39.3 | -2.6 | 1.69 |
| 2_3md75 | 110.7 | 111.2 | 0.5 | 42.6 | 3.3 | 1.11 |
| 2_3md76 | 111.2 | 111.5 | 0.3 | 39.2 | -3.4 | 0.99 |
| 2_3md77 | 111.5 | 112.3 | 0.8 | 42.2 | 2.9 | 0.78 |
| 2_3md78 | 112.3 | 112.6 | 0.3 | 39.3 | -2.9 | 1.49 |
| 2_3md79 | 112.6 | 113.1 | 0.5 | 43.3 | 4.0 | 1.96 |
| 2_3md80 | 113.1 | 113.8 | 0.7 | 47.0 | 3.7 | 2.24 |
| 2_3md81 | 113.8 | 114.2 | 0.4 | 40.1 | -6.9 | 1.44 |
| 2_3md82 | 114.2 | 114.4 | 0.2 | 46.9 | 6.8 | 0 |
| 2_3md83 | 114.4 | 115.7 | 1.3 | 42.9 | -4.0 | 1.4 |

| | | | | | | |
|---|---|---|---|---|---|---|
| 2_3md84 | 115.7 | 117.1 | 1.4 | 39.9 | -3.0 | 1.05 |
| 2_3md85 | 117.1 | 117.6 | 0.5 | 42.1 | 2.2 | 0.58 |
| 2_3md86 | 117.6 | 117.9 | 0.3 | 39.4 | -2.7 | 0.92 |
| 2_3md87 | 117.9 | 118.6 | 0.7 | 43.0 | 3.6 | 2.06 |
| 2_3md88 | 118.6 | 120 | 1.4 | 39.9 | -3.1 | 0.68 |
| 2_3md89 | 120 | 120.8 | 0.8 | 43.1 | 3.1 | 2.47 |
| 2_3md90 | 120.8 | 121 | 0.2 | 49.2 | 6.1 | 0 |
| 2_3md91 | 121 | 121.9 | 0.9 | 40.3 | -8.8 | 2.74 |
| 2_3md92 | 121.9 | 123.5 | 1.6 | 43.3 | 3.0 | 2.2 |
| 2_3md93 | 123.5 | 123.8 | 0.3 | 48.6 | 5.3 | 0.68 |
| 2_3md94 | 123.8 | 124 | 0.2 | 43.8 | -4.9 | 0 |
| 2_3md95 | 124 | 124.2 | 0.2 | 50.8 | 7.0 | 0 |
| 2_3md96 | 124.2 | 124.5 | 0.3 | 45.0 | -5.8 | 2.38 |
| 2_3md97 | 124.5 | 124.7 | 0.2 | 38.1 | -6.9 | 0 |
| 2_3md98 | 124.7 | 126.4 | 1.7 | 43.2 | 5.1 | 1.95 |
| 2_3md99 | 126.4 | 126.6 | 0.2 | 38.0 | -5.2 | 0 |
| 2_3md100 | 126.6 | 126.9 | 0.3 | 43.0 | 5.0 | 1.73 |
| 2_3md101 | 126.9 | 127.3 | 0.4 | 49.1 | 6.1 | 2.63 |
| 2_3md102 | 127.3 | 128.1 | 0.8 | 45.0 | -4.1 | 4.19 |
| 2_3md103 | 128.1 | 128.3 | 0.2 | 48.1 | 3.2 | 0 |
| 2_3md104 | 128.3 | 128.6 | 0.3 | 40.7 | -7.5 | 0.31 |
| 2_3md105 | 128.6 | 129.1 | 0.5 | 42.2 | 1.6 | 2.24 |
| 2_3md106 | 129.1 | 130 | 0.9 | 50.7 | 8.5 | 1.89 |
| 2_3md107 | 130 | 130.2 | 0.2 | 44.4 | -6.3 | 0 |
| 2_3md108 | 130.2 | 130.8 | 0.6 | 53.0 | 8.6 | 3.59 |
| 2_3md109 | 130.8 | 131 | 0.2 | 40.8 | -12.2 | 0 |
| 2_3md110 | 131 | 131.3 | 0.3 | 44.5 | 3.8 | 2.12 |
| 2_3md111 | 131.3 | 131.5 | 0.2 | 38.3 | -6.3 | 0 |
| 2_3md112 | 131.5 | 133.1 | 1.6 | 41.9 | 3.7 | 1.53 |
| 2_3md113 | 133.1 | 134.2 | 1.1 | 39.1 | -2.8 | 1.14 |
| 2_3md114 | 134.2 | 134.7 | 0.5 | 43.4 | 4.3 | 2.21 |
| 2_3md115 | 134.7 | 135.1 | 0.4 | 53.2 | 9.8 | 1.23 |
| 2_3md116 | 135.1 | 135.6 | 0.5 | 47.1 | -6.1 | 2.53 |
| 2_3md117 | 135.6 | 136.2 | 0.6 | 43.7 | -3.3 | 3.05 |
| 2_3md118 | 136.2 | 136.8 | 0.6 | 47.3 | 3.5 | 2.11 |
| 2_3md119 | 136.8 | 137.6 | 0.8 | 44.8 | -2.4 | 2.11 |
| 2_3md120 | 137.6 | 138 | 0.4 | 48.6 | 3.8 | 2.69 |
| 2_3md121 | 138 | 138.4 | 0.4 | 42.9 | -5.8 | 1.62 |
| 2_3md122 | 138.4 | 138.6 | 0.2 | 47.2 | 4.3 | 0 |
| 2_3md123 | 138.6 | 139 | 0.4 | 43.0 | -4.2 | 1.31 |
| 2_3md124 | 139 | 139.5 | 0.5 | 38.9 | -4.1 | 1.16 |
| 2_3md125 | 139.5 | 140.1 | 0.6 | 44.9 | 6.0 | 1.85 |
| 2_3md126 | 140.1 | 140.3 | 0.2 | 47.6 | 2.7 | 0 |
| 2_3md127 | 140.3 | 140.8 | 0.5 | 42.0 | -5.6 | 0.96 |
| 2_3md128 | 140.8 | 141.4 | 0.6 | 50.2 | 8.2 | 2.67 |
| 2_3md129 | 141.4 | 142.6 | 1.2 | 43.4 | -6.8 | 2.59 |
| 2_3md130 | 142.6 | 142.8 | 0.2 | 47.8 | 4.4 | 0 |
| 2_3md131 | 142.8 | 143.1 | 0.3 | 55.4 | 7.6 | 1.4 |
| 2_3md132 | 143.1 | 143.8 | 0.7 | 44.8 | -10.7 | 3.44 |
| 2_3md133 | 143.8 | 144.4 | 0.6 | 48.2 | 3.5 | 2.3 |
| 2_3md134 | 144.4 | 144.7 | 0.3 | 37.7 | -10.6 | 1.05 |
| 2_3md135 | 144.7 | 145.6 | 0.9 | 45.9 | 8.2 | 4.73 |
| 2_3md136 | 145.6 | 145.8 | 0.2 | 59.1 | 13.2 | 0 |
| 2_3md137 | 145.8 | 146.7 | 0.9 | 40.1 | -19.1 | 0.64 |
| 2_3md138 | 146.7 | 147.6 | 0.9 | 48.2 | 8.1 | 3.55 |
| 2_3md139 | 147.6 | 148.2 | 0.6 | 45.2 | -3.0 | 3.06 |

| | | | | | | |
|---|---|---|---|---|---|---|
| 3_1md1 | 0 | 0.6 | 0.6 | 42.0 | | 1.63 |
| 3_1md2 | 0.6 | 0.9 | 0.3 | 39.5 | -2.5 | 0.77 |
| 3_1md3 | 0.9 | 1.5 | 0.6 | 42.6 | 3.1 | 1.41 |
| 3_1md4 | 1.5 | 3.2 | 1.7 | 38.5 | -4.1 | 1.54 |
| 3_1md5 | 3.2 | 4 | 0.8 | 36.4 | -2.1 | 0.62 |
| 3_1md6 | 4 | 5.1 | 1.1 | 37.9 | 1.6 | 1.35 |
| 3_1md7 | 5.1 | 5.6 | 0.5 | 41.8 | 3.8 | 0.77 |
| 3_1md8 | 5.6 | 5.9 | 0.3 | 38.6 | -3.2 | 2 |
| 3_1md9 | 5.9 | 10.9 | 5 | 34.7 | -3.8 | 1.01 |
| 3_1md10 | 10.9 | 11.1 | 0.2 | 37.7 | 3.0 | 0 |
| 3_1md11 | 11.1 | 11.5 | 0.4 | 36.7 | -1.0 | 0.3 |
| 3_1md12 | 11.5 | 12.2 | 0.7 | 38.6 | 2.0 | 1.61 |
| 3_1md13 | 12.2 | 12.4 | 0.2 | 35.6 | -3.1 | 0 |
| 3_1md14 | 12.4 | 12.6 | 0.2 | 38.0 | 2.4 | 0 |
| 3_1md15 | 12.6 | 14.6 | 2 | 35.0 | -3.0 | 1.03 |
| 3_1md16 | 14.6 | 18.4 | 3.8 | 38.4 | 3.4 | 1.56 |
| 3_1md17 | 18.4 | 18.6 | 0.2 | 36.9 | -1.5 | 0 |
| 3_1md18 | 18.6 | 22 | 3.4 | 38.0 | 1.1 | 1.1 |
| 3_1md19 | 22 | 22.2 | 0.2 | 36.3 | -1.8 | 0 |
| 3_1md20 | 22.2 | 23 | 0.8 | 37.1 | 0.8 | 0.37 |
| 3_1md21 | 23 | 24.1 | 1.1 | 35.9 | -1.1 | 0.75 |
| 3_1md22 | 24.1 | 24.4 | 0.3 | 37.5 | 1.6 | 0.15 |
| 3_1md23 | 24.4 | 25.1 | 0.7 | 36.8 | -0.8 | 1.26 |
| 3_1md24 | 25.1 | 25.4 | 0.3 | 39.1 | 2.3 | 0.8 |
| 3_1md25 | 25.4 | 25.6 | 0.2 | 34.9 | -4.2 | 0 |
| 3_1md26 | 25.6 | 26.6 | 1 | 39.4 | 4.5 | 1.06 |
| 3_1md27 | 26.6 | 29.2 | 2.6 | 35.7 | -3.7 | 0.91 |
| 3_1md28 | 29.2 | 29.7 | 0.5 | 37.6 | 1.9 | 1.51 |
| 3_1md29 | 29.7 | 30.1 | 0.4 | 35.8 | -1.8 | 0.52 |
| 3_1md30 | 30.1 | 31.2 | 1.1 | 38.0 | 2.2 | 1.13 |
| 3_1md31 | 31.2 | 31.5 | 0.3 | 35.7 | -2.3 | 1.32 |
| 3_1md32 | 31.5 | 32.4 | 0.9 | 37.9 | 2.2 | 0.82 |
| 3_1md33 | 32.4 | 32.6 | 0.2 | 36.5 | -1.4 | 0 |
| 3_1md34 | 32.6 | 33.2 | 0.6 | 38.6 | 2.1 | 1 |
| 3_1md35 | 33.2 | 33.4 | 0.2 | 36.4 | -2.2 | 0 |
| 3_1md36 | 33.4 | 38 | 4.6 | 38.2 | 1.8 | 1.04 |
| 3_1md37 | 38 | 38.4 | 0.4 | 34.9 | -3.2 | 1.27 |
| 3_1md38 | 38.4 | 38.9 | 0.5 | 37.5 | 2.6 | 1.49 |
| 3_1md39 | 38.9 | 39.1 | 0.2 | 36.7 | -0.8 | 0 |
| 3_1md40 | 39.1 | 39.7 | 0.6 | 38.0 | 1.3 | 1.43 |
| 3_1md41 | 39.7 | 39.9 | 0.2 | 36.3 | -1.7 | 0 |
| 3_1md42 | 39.9 | 40.2 | 0.3 | 39.4 | 3.0 | 1.41 |
| 3_1md43 | 40.2 | 40.4 | 0.2 | 43.7 | 4.3 | 0 |
| 3_1md44 | 40.4 | 40.9 | 0.5 | 37.6 | -6.1 | 1 |
| 3_1md45 | 40.9 | 44.8 | 3.9 | 35.4 | -2.2 | 0.79 |
| 3_1md46 | 44.8 | 45 | 0.2 | 37.5 | 2.2 | 0 |
| 3_1md47 | 45 | 45.3 | 0.3 | 35.8 | -1.7 | 0.96 |
| 3_1md48 | 45.3 | 46.9 | 1.6 | 38.2 | 2.3 | 0.59 |
| 3_1md49 | 46.9 | 47.3 | 0.4 | 35.7 | -2.4 | 0.76 |
| 3_1md50 | 47.3 | 48.2 | 0.9 | 38.5 | 2.8 | 1.03 |
| 3_1md51 | 48.2 | 48.4 | 0.2 | 36.6 | -1.9 | 0 |
| 3_1md52 | 48.4 | 50.6 | 2.2 | 38.2 | 1.6 | 0.84 |
| 3_1md53 | 50.6 | 50.9 | 0.3 | 35.5 | -2.7 | 0.46 |
| 3_1md54 | 50.9 | 51.3 | 0.4 | 37.7 | 2.2 | 1.49 |
| 3_1md55 | 51.3 | 51.6 | 0.3 | 36.5 | -1.2 | 0.41 |

| | | | | | | |
|---|---|---|---|---|---|---|
| 3_1md56 | 51.6 | 52.3 | 0.7 | 38.3 | 1.7 | 1.38 |
| 3_1md57 | 52.3 | 53.8 | 1.5 | 35.8 | -2.4 | 0.87 |
| 3_1md58 | 53.8 | 54.2 | 0.4 | 37.2 | 1.3 | 0.17 |
| 3_1md59 | 54.2 | 56.5 | 2.3 | 35.8 | -1.4 | 0.76 |
| 3_1md60 | 56.5 | 56.9 | 0.4 | 37.4 | 1.7 | 0.68 |
| 3_1md61 | 56.9 | 59.8 | 2.9 | 0.0 | -37.4 | 0 |
| 3_1md62 | 59.8 | 62.2 | 2.4 | 35.4 | 35.4 | 0.73 |
| 3_1md63 | 62.2 | 62.7 | 0.5 | 37.7 | 2.4 | 0.28 |
| 3_1md64 | 62.7 | 62.9 | 0.2 | 36.7 | -1.0 | 0 |
| 3_1md65 | 62.9 | 68.1 | 5.2 | 38.9 | 2.2 | 1.51 |
| 3_1md66 | 68.1 | 68.6 | 0.5 | 42.1 | 3.2 | 0.58 |
| 3_1md67 | 68.6 | 69.2 | 0.6 | 36.1 | -5.9 | 1.15 |
| 3_1md68 | 69.2 | 71.5 | 2.3 | 38.7 | 2.6 | 1.23 |
| 3_1md69 | 71.5 | 75 | 3.5 | 35.5 | -3.3 | 0.94 |
| 3_1md70 | 75 | 76.1 | 1.1 | 37.7 | 2.2 | 0.71 |
| 3_1md71 | 76.1 | 84.3 | 8.2 | 35.6 | -2.1 | 0.82 |
| 3_1md72 | 84.3 | 84.7 | 0.4 | 37.7 | 2.1 | 0.47 |
| 3_1md73 | 84.7 | 85.1 | 0.4 | 36.9 | -0.8 | 0.36 |
| 3_1md74 | 85.1 | 85.7 | 0.6 | 41.6 | 4.7 | 0.31 |
| 3_1md75 | 85.7 | 86.3 | 0.6 | 36.0 | -5.7 | 0.97 |
| 3_1md76 | 86.3 | 86.7 | 0.4 | 37.7 | 1.7 | 0.67 |
| 3_1md77 | 86.7 | 87.1 | 0.4 | 36.6 | -1.1 | 0.39 |
| 3_1md78 | 87.1 | 87.3 | 0.2 | 37.5 | 0.9 | 0 |
| 3_1md79 | 87.3 | 87.5 | 0.2 | 35.9 | -1.6 | 0 |
| 3_1md80 | 87.5 | 87.8 | 0.3 | 38.6 | 2.7 | 1.07 |
| 3_1md81 | 87.8 | 88 | 0.2 | 36.5 | -2.1 | 0 |
| 3_1md82 | 88 | 89.5 | 1.5 | 37.6 | 1.2 | 0.49 |
| 3_1md83 | 89.5 | 89.7 | 0.2 | 35.8 | -1.8 | 0 |
| 3_1md84 | 89.7 | 89.9 | 0.2 | 37.6 | 1.8 | 0 |
| 3_1md85 | 89.9 | 90.8 | 0.9 | 36.6 | -1.0 | 0.55 |
| 3_1md86 | 90.8 | 91.9 | 1.1 | 38.2 | 1.6 | 0.77 |
| 3_1md87 | 91.9 | 92.7 | 0.8 | 36.6 | -1.7 | 1.04 |
| 3_1md88 | 92.7 | 92.9 | 0.2 | 37.6 | 1.1 | 0 |
| 3_1md89 | 92.9 | 99.6 | 6.7 | 35.2 | -2.5 | 1.03 |
| 3_1md90 | 99.6 | 99.8 | 0.2 | 39.1 | 4.0 | 0 |
| 3_1md91 | 99.8 | 101 | 1.2 | 36.1 | -3.1 | 0.65 |
| 3_1md92 | 101 | 101.2 | 0.2 | 37.2 | 1.1 | 0 |
| 3_1md93 | 101.2 | 105.8 | 4.6 | 35.0 | -2.2 | 1.23 |
| 3_1md94 | 105.8 | 106.8 | 1 | 37.3 | 2.3 | 0.99 |
| 3_1md95 | 106.8 | 107 | 0.2 | 36.7 | -0.6 | 0 |
| 3_1md96 | 107 | 107.2 | 0.2 | 37.7 | 1.1 | 0 |
| 3_1md97 | 107.2 | 112.4 | 5.2 | 34.6 | -3.1 | 0.95 |
| 3_1md98 | 112.4 | 112.8 | 0.4 | 37.6 | 3.0 | 0.45 |
| 3_1md99 | 112.8 | 113.4 | 0.6 | 36.5 | -1.2 | 0.58 |
| 3_1md100 | 113.4 | 113.6 | 0.2 | 38.6 | 2.2 | 0 |
| 3_1md101 | 113.6 | 114.9 | 1.3 | 35.8 | -2.9 | 1.28 |
| 3_1md102 | 114.9 | 115.1 | 0.2 | 38.1 | 2.3 | 0 |
| 3_1md103 | 115.1 | 115.6 | 0.5 | 36.9 | -1.2 | 0.84 |
| 3_1md104 | 115.6 | 116 | 0.4 | 37.5 | 0.6 | 0.56 |
| 3_1md105 | 116 | 141 | 25 | 34.4 | -3.1 | 0.94 |
| 3_1md106 | 141 | 141.6 | 0.6 | 37.9 | 3.4 | 0.91 |
| 3_1md107 | 141.6 | 142 | 0.4 | 35.2 | -2.7 | 0.32 |
| 3_1md108 | 142 | 143.8 | 1.8 | 37.7 | 2.5 | 1.04 |
| 3_1md109 | 143.8 | 144.3 | 0.5 | 36.6 | -1.1 | 0.26 |
| 3_1md110 | 144.3 | 145 | 0.7 | 40.1 | 3.5 | 2.04 |
| 3_1md111 | 145 | 151.5 | 6.5 | 35.0 | -5.1 | 1.11 |

| | | | | | | |
|---|---|---|---|---|---|---|
| 3_1md112 | 151.5 | 152.5 | 1 | 37.8 | 2.8 | 0.75 |
| 3_1md113 | 152.5 | 152.7 | 0.2 | 36.1 | -1.7 | 0 |
| 3_1md114 | 152.7 | 152.9 | 0.2 | 38.0 | 1.9 | 0 |
| 3_1md115 | 152.9 | 153.2 | 0.3 | 36.2 | -1.7 | 0.18 |
| 3_1md116 | 153.2 | 153.8 | 0.6 | 38.1 | 1.8 | 1.11 |
| 3_1md117 | 153.8 | 154.3 | 0.5 | 36.8 | -1.2 | 0.56 |
| 3_1md118 | 154.3 | 154.6 | 0.3 | 38.6 | 1.8 | 0.13 |
| 3_1md119 | 154.6 | 162.2 | 7.6 | 35.0 | -3.6 | 0.95 |
| 3_1md120 | 162.2 | 162.5 | 0.3 | 38.0 | 2.9 | 0.77 |
| 3_1md121 | 162.5 | 163.1 | 0.6 | 36.3 | -1.7 | 0.48 |
| 3_1md122 | 163.1 | 163.6 | 0.5 | 38.6 | 2.3 | 1.23 |
| 3_1md123 | 163.6 | 165.9 | 2.3 | 35.7 | -2.9 | 0.84 |
| 3_1md124 | 165.9 | 166.6 | 0.7 | 37.6 | 1.9 | 0.61 |
| 3_1md125 | 166.6 | 166.9 | 0.3 | 35.0 | -2.6 | 1.01 |
| 3_1md126 | 166.9 | 167.1 | 0.2 | 38.0 | 2.9 | 0 |
| 3_1md127 | 167.1 | 170.7 | 3.6 | 35.6 | -2.4 | 1.05 |
| 3_1md128 | 170.7 | 171.9 | 1.2 | 37.8 | 2.2 | 1.3 |
| 3_1md129 | 171.9 | 175.5 | 3.6 | 35.9 | -2.0 | 0.75 |
| 3_1md130 | 175.5 | 175.8 | 0.3 | 37.4 | 1.5 | 0.25 |
| 3_1md131 | 175.8 | 177.5 | 1.7 | 35.7 | -1.7 | 1.11 |
| 3_1md132 | 177.5 | 178 | 0.5 | 38.0 | 2.4 | 0.54 |
| 3_1md133 | 178 | 178.4 | 0.4 | 35.5 | -2.5 | 1.4 |
| 3_1md134 | 178.4 | 178.6 | 0.2 | 37.3 | 1.8 | 0 |
| 3_1md135 | 178.6 | 185.9 | 7.3 | 35.8 | -1.6 | 0.8 |
| 3_1md136 | 185.9 | 186.5 | 0.6 | 37.2 | 1.4 | 0.35 |
| 3_1md137 | 186.5 | 194 | 7.5 | 35.1 | -2.1 | 0.99 |
| 3_1md138 | 194 | 194.5 | 0.5 | 37.7 | 2.6 | 0.36 |
| 3_1md139 | 194.5 | 194.7 | 0.2 | 36.6 | -1.1 | 0 |
| 3_1md140 | 194.7 | 195 | 0.3 | 37.5 | 0.9 | 3.8 |
| 3_1md141 | 195 | 196 | 1 | 36.0 | -1.4 | 0.96 |
| 3_1md142 | 196 | 196.3 | 0.3 | 37.5 | 1.5 | 0.42 |
| 3_1md143 | 196.3 | 200.4 | 4.1 | 36.1 | -1.4 | 1.02 |
| 3_1md144 | 200.4 | 200.8 | 0.4 | 37.3 | 1.2 | 0.87 |
| 3_1md145 | 200.8 | 203.9 | 3.1 | 34.8 | -2.5 | 0.88 |
| 3_1md146 | 203.9 | 204.1 | 0.2 | 40.2 | 5.4 | 0 |
| 3_1md147 | 204.1 | 204.8 | 0.7 | 36.7 | -3.5 | 0.48 |
| 3_1md148 | 204.8 | 205.2 | 0.4 | 37.4 | 0.7 | 0.51 |
| 3_1md149 | 205.2 | 205.7 | 0.5 | 36.6 | -0.8 | 0.4 |
| 3_1md150 | 205.7 | 206.3 | 0.6 | 36.7 | 0.0 | 1.13 |
| 3_1md151 | 206.3 | 206.5 | 0.2 | 37.7 | 1.0 | 0 |
| 3_1md152 | 206.5 | 211.9 | 5.4 | 35.3 | -2.4 | 1.01 |
| 3_1md153 | 211.9 | 212.5 | 0.6 | 37.7 | 2.4 | 0.42 |
| 3_1md154 | 212.5 | 225.2 | 12.7 | 35.6 | -2.1 | 0.95 |
| 3_1md155 | 225.2 | 225.4 | 0.2 | 38.5 | 3.0 | 0 |
| 3_1md156 | 225.4 | 225.7 | 0.3 | 35.7 | -2.8 | 0.63 |
| 3_1md157 | 225.7 | 226.1 | 0.4 | 37.9 | 2.2 | 0.46 |
| 3_1md158 | 226.1 | 226.5 | 0.4 | 34.5 | -3.4 | 0.92 |
| 3_1md159 | 226.5 | 227.6 | 1.1 | 37.6 | 3.2 | 0.69 |
| 3_1md160 | 227.6 | 231.1 | 3.5 | 35.0 | -2.6 | 0.82 |
| 3_1md161 | 231.1 | 232.4 | 1.3 | 38.3 | 3.2 | 1.18 |
| 3_1md162 | 232.4 | 233.8 | 1.4 | 36.7 | -1.5 | 0.57 |
| 3_1md163 | 233.8 | 234 | 0.2 | 37.6 | 0.9 | 0 |
| 3_1md164 | 234 | 234.2 | 0.2 | 36.4 | -1.3 | 0 |
| 3_1md165 | 234.2 | 234.5 | 0.3 | 37.6 | 1.2 | 0.54 |
| 3_1md166 | 234.5 | 235.6 | 1.1 | 36.0 | -1.6 | 0.65 |
| 3_1md167 | 235.6 | 236.3 | 0.7 | 38.0 | 2.0 | 0.61 |

| | | | | | | |
|---|---|---|---|---|---|---|
| 3_1md168 | 236.3 | 236.6 | 0.3 | 35.9 | -2.1 | 0.95 |
| 3_1md169 | 236.6 | 237 | 0.4 | 38.7 | 2.8 | 1.14 |
| 3_1md170 | 237 | 237.2 | 0.2 | 35.6 | -3.1 | 0 |
| 3_1md171 | 237.2 | 238.3 | 1.1 | 38.0 | 2.4 | 1.02 |
| 3_1md172 | 238.3 | 239.4 | 1.1 | 35.6 | -2.5 | 1.21 |
| 3_1md173 | 239.4 | 240.1 | 0.7 | 38.1 | 2.5 | 0.59 |
| 3_1md174 | 240.1 | 240.3 | 0.2 | 36.4 | -1.7 | 0 |
| 3_1md175 | 240.3 | 240.5 | 0.2 | 38.0 | 1.6 | 0 |
| 3_1md176 | 240.5 | 241.3 | 0.8 | 35.7 | -2.3 | 0.79 |
| 3_1md177 | 241.3 | 243.6 | 2.3 | 37.4 | 1.7 | 0.75 |
| 3_1md178 | 243.6 | 244 | 0.4 | 36.7 | -0.7 | 0.32 |
| 3_1md179 | 244 | 244.6 | 0.6 | 37.8 | 1.1 | 0.83 |
| 3_1md180 | 244.6 | 244.8 | 0.2 | 36.5 | -1.2 | 0 |
| 3_1md181 | 244.8 | 247.5 | 2.7 | 38.1 | 1.5 | 1.31 |
| 3_1md182 | 247.5 | 247.9 | 0.4 | 35.6 | -2.4 | 0.67 |
| 3_1md183 | 247.9 | 249.4 | 1.5 | 37.7 | 2.0 | 0.78 |
| 3_1md184 | 249.4 | 250 | 0.6 | 34.6 | -3.0 | 1.03 |
| 3_1md185 | 250 | 250.4 | 0.4 | 38.1 | 3.5 | 0.16 |
| 3_1md186 | 250.4 | 251.9 | 1.5 | 35.5 | -2.6 | 1.26 |
| 3_1md187 | 251.9 | 252.4 | 0.5 | 37.7 | 2.2 | 0.67 |
| 3_1md188 | 252.4 | 256.9 | 4.5 | 35.3 | -2.4 | 1.07 |
| 3_1md189 | 256.9 | 257.5 | 0.6 | 37.7 | 2.4 | 0.39 |
| 3_1md190 | 257.5 | 257.9 | 0.4 | 36.0 | -1.7 | 0.81 |
| 3_1md191 | 257.9 | 258.6 | 0.7 | 37.5 | 1.5 | 0.74 |
| 3_1md192 | 258.6 | 259.2 | 0.6 | 36.7 | -0.8 | 0.83 |
| 3_1md193 | 259.2 | 259.8 | 0.6 | 37.6 | 0.9 | 0.28 |
| 3_1md194 | 259.8 | 260.9 | 1.1 | 36.5 | -1.1 | 0.82 |
| 3_1md195 | 260.9 | 261.8 | 0.9 | 39.3 | 2.8 | 1.23 |
| 3_1md196 | 261.8 | 262 | 0.2 | 35.4 | -3.9 | 0 |
| 3_1md197 | 262 | 263.3 | 1.3 | 38.3 | 2.9 | 0.72 |
| 3_1md198 | 263.3 | 263.6 | 0.3 | 36.1 | -2.2 | 0.98 |
| 3_1md199 | 263.6 | 265.2 | 1.6 | 37.2 | 1.1 | 1.16 |
| 3_1md200 | 265.2 | 265.4 | 0.2 | 36.1 | -1.1 | 0 |
| 3_1md201 | 265.4 | 265.6 | 0.2 | 37.6 | 1.5 | 0 |
| | | | | | | |
| 3_2md1 | 0 | 2.6 | 2.6 | 35.3 | | 0.87 |
| 3_2md2 | 2.6 | 3.8 | 1.2 | 38.1 | 2.8 | 1.24 |
| 3_2md3 | 3.8 | 6.3 | 2.5 | 35.8 | -2.3 | 0.81 |
| 3_2md4 | 6.3 | 6.8 | 0.5 | 38.1 | 2.3 | 0.65 |
| 3_2md5 | 6.8 | 7.6 | 0.8 | 36.5 | -1.6 | 0.91 |
| 3_2md6 | 7.6 | 8 | 0.4 | 37.9 | 1.4 | 0.4 |
| 3_2md7 | 8 | 8.2 | 0.2 | 35.7 | -2.2 | 0 |
| 3_2md8 | 8.2 | 8.7 | 0.5 | 37.4 | 1.7 | 0.29 |
| 3_2md9 | 8.7 | 10.4 | 1.7 | 35.9 | -1.5 | 1.16 |
| 3_2md10 | 10.4 | 10.6 | 0.2 | 37.3 | 1.3 | 0 |
| 3_2md11 | 10.6 | 11.6 | 1 | 36.5 | -0.8 | 0.97 |
| 3_2md12 | 11.6 | 12.4 | 0.8 | 37.7 | 1.2 | 0.49 |
| 3_2md13 | 12.4 | 14.3 | 1.9 | 35.6 | -2.2 | 0.9 |
| 3_2md14 | 14.3 | 14.9 | 0.6 | 37.7 | 2.1 | 0.67 |
| 3_2md15 | 14.9 | 15.8 | 0.9 | 36.0 | -1.7 | 1.24 |
| 3_2md16 | 15.8 | 16 | 0.2 | 38.3 | 2.3 | 0 |
| 3_2md17 | 16 | 36.6 | 20.6 | 34.8 | -3.5 | 1.02 |
| 3_2md18 | 36.6 | 37.1 | 0.5 | 37.8 | 3.0 | 0.52 |
| 3_2md19 | 37.1 | 37.7 | 0.6 | 33.4 | -4.5 | 1.62 |
| 3_2md20 | 37.7 | 37.9 | 0.2 | 39.0 | 5.6 | 0 |
| 3_2md21 | 37.9 | 45 | 7.1 | 35.5 | -3.5 | 0.99 |

| | | | | | | |
|---|---|---|---|---|---|---|
| 3_2md22 | 45 | 45.4 | 0.4 | 37.9 | 2.5 | 0.52 |
| 3_2md23 | 45.4 | 45.8 | 0.4 | 36.4 | -1.5 | 1.45 |
| 3_2md24 | 45.8 | 46.2 | 0.4 | 37.2 | 0.8 | 0.31 |
| 3_2md25 | 46.2 | 46.7 | 0.5 | 36.4 | -0.9 | 0.5 |
| 3_2md26 | 46.7 | 47.3 | 0.6 | 38.4 | 2.0 | 0.43 |
| 3_2md27 | 47.3 | 47.7 | 0.4 | 35.5 | -2.9 | 0.78 |
| 3_2md28 | 47.7 | 48.2 | 0.5 | 37.8 | 2.3 | 0.5 |
| 3_2md29 | 48.2 | 48.4 | 0.2 | 37.0 | -0.8 | 0 |
| 3_2md30 | 48.4 | 48.9 | 0.5 | 37.8 | 0.8 | 0.8 |
| 3_2md31 | 48.9 | 52.3 | 3.4 | 35.6 | -2.3 | 0.73 |
| 3_2md32 | 52.3 | 52.7 | 0.4 | 38.0 | 2.4 | 0.46 |
| 3_2md33 | 52.7 | 53.9 | 1.2 | 35.7 | -2.3 | 1.16 |
| 3_2md34 | 53.9 | 54.1 | 0.2 | 37.2 | 1.5 | 0 |
| 3_2md35 | 54.1 | 55.7 | 1.6 | 36.4 | -0.8 | 0.67 |
| 3_2md36 | 55.7 | 56.5 | 0.8 | 37.5 | 1.0 | 0.83 |
| 3_2md37 | 56.5 | 57.4 | 0.9 | 36.2 | -1.3 | 0.89 |
| 3_2md38 | 57.4 | 57.8 | 0.4 | 37.6 | 1.4 | 0.46 |
| 3_2md39 | 57.8 | 58 | 0.2 | 36.7 | -0.9 | 0 |
| 3_2md40 | 58 | 59 | 1 | 37.7 | 1.0 | 0.82 |
| 3_2md41 | 59 | 59.2 | 0.2 | 36.5 | -1.2 | 0 |
| 3_2md42 | 59.2 | 62.7 | 3.5 | 38.3 | 1.7 | 1.26 |
| 3_2md43 | 62.7 | 64.5 | 1.8 | 36.2 | -2.1 | 0.78 |
| 3_2md44 | 64.5 | 64.7 | 0.2 | 37.4 | 1.2 | 0 |
| 3_2md45 | 64.7 | 65 | 0.3 | 36.6 | -0.8 | 0.26 |
| 3_2md46 | 65 | 65.5 | 0.5 | 38.2 | 1.6 | 0.83 |
| 3_2md47 | 65.5 | 65.9 | 0.4 | 36.1 | -2.1 | 0.48 |
| 3_2md48 | 65.9 | 66.8 | 0.9 | 37.8 | 1.7 | 0.7 |
| 3_2md49 | 66.8 | 71.1 | 4.3 | 35.6 | -2.2 | 0.92 |
| 3_2md50 | 71.1 | 71.5 | 0.4 | 37.5 | 1.9 | 0.98 |
| 3_2md51 | 71.5 | 71.7 | 0.2 | 36.2 | -1.3 | 0 |
| 3_2md52 | 71.7 | 73.8 | 2.1 | 38.3 | 2.1 | 1.24 |
| 3_2md53 | 73.8 | 74.7 | 0.9 | 36.7 | -1.5 | 0.52 |
| 3_2md54 | 74.7 | 74.9 | 0.2 | 37.9 | 1.1 | 0 |
| 3_2md55 | 74.9 | 75.7 | 0.8 | 36.5 | -1.3 | 1.21 |
| 3_2md56 | 75.7 | 76.3 | 0.6 | 37.1 | 0.6 | 0.98 |
| 3_2md57 | 76.3 | 76.5 | 0.2 | 35.3 | -1.8 | 0 |
| 3_2md58 | 76.5 | 78.3 | 1.8 | 38.5 | 3.2 | 0.92 |
| 3_2md59 | 78.3 | 79 | 0.7 | 35.5 | -3.0 | 1.15 |
| 3_2md60 | 79 | 79.2 | 0.2 | 37.2 | 1.7 | 0 |
| 3_2md61 | 79.2 | 79.4 | 0.2 | 36.6 | -0.6 | 0 |
| 3_2md62 | 79.4 | 79.9 | 0.5 | 38.9 | 2.2 | 1.25 |
| 3_2md63 | 79.9 | 81.4 | 1.5 | 35.9 | -2.9 | 1.05 |
| 3_2md64 | 81.4 | 81.7 | 0.3 | 37.3 | 1.4 | 0.05 |
| 3_2md65 | 81.7 | 91.6 | 9.9 | 35.5 | -1.7 | 0.98 |
| 3_2md66 | 91.6 | 92.2 | 0.6 | 37.9 | 2.3 | 0.7 |
| 3_2md67 | 92.2 | 92.9 | 0.7 | 36.1 | -1.8 | 0.86 |
| 3_2md68 | 92.9 | 93.5 | 0.6 | 38.3 | 2.3 | 0.77 |
| 3_2md69 | 93.5 | 93.9 | 0.4 | 36.7 | -1.7 | 0.3 |
| 3_2md70 | 93.9 | 94.1 | 0.2 | 37.1 | 0.5 | 0 |
| 3_2md71 | 94.1 | 94.3 | 0.2 | 36.6 | -0.5 | 0 |
| 3_2md72 | 94.3 | 95.2 | 0.9 | 37.6 | 1.0 | 0.9 |
| 3_2md73 | 95.2 | 95.8 | 0.6 | 36.3 | -1.3 | 0.57 |
| 3_2md74 | 95.8 | 97.6 | 1.8 | 38.2 | 1.9 | 0.86 |
| 3_2md75 | 97.6 | 98.1 | 0.5 | 35.2 | -3.1 | 0.58 |
| 3_2md76 | 98.1 | 98.3 | 0.2 | 38.6 | 3.5 | 0 |
| 3_2md77 | 98.3 | 99.3 | 1 | 34.7 | -3.9 | 1.03 |

| | | | | | | |
|---|---|---|---|---|---|---|
| 3_2md78 | 99.3 | 99.5 | 0.2 | 39.0 | 4.3 | 0 |
| 3_2md79 | 99.5 | 99.7 | 0.2 | 36.1 | -2.9 | 0 |
| 3_2md80 | 99.7 | 102.6 | 2.9 | 38.1 | 2.0 | 0.88 |
| 3_2md81 | 102.6 | 102.9 | 0.3 | 35.1 | -3.0 | 1.46 |
| 3_2md82 | 102.9 | 104.1 | 1.2 | 38.2 | 3.0 | 1.37 |
| 3_2md83 | 104.1 | 104.4 | 0.3 | 36.5 | -1.7 | 0.85 |
| 3_2md84 | 104.4 | 106.1 | 1.7 | 35.3 | -1.1 | 1.74 |
| 3_2md85 | 106.1 | 106.3 | 0.2 | 37.4 | 2.1 | 0 |
| 3_2md86 | 106.3 | 112.3 | 6 | 35.4 | -2.0 | 0.83 |
| 3_2md87 | 112.3 | 112.5 | 0.2 | 37.4 | 2.0 | 0 |
| 3_2md88 | 112.5 | 123.1 | 10.6 | 34.8 | -2.6 | 1.1 |
| 3_2md89 | 123.1 | 123.9 | 0.8 | 37.5 | 2.7 | 1.74 |
| 3_2md90 | 123.9 | 124.1 | 0.2 | 36.8 | -0.7 | 0 |
| 3_2md91 | 124.1 | 124.3 | 0.2 | 37.6 | 0.8 | 0 |
| 3_2md92 | 124.3 | 124.6 | 0.3 | 36.5 | -1.1 | 0.16 |
| 3_2md93 | 124.6 | 125.5 | 0.9 | 37.8 | 1.3 | 0.66 |
| 3_2md94 | 125.5 | 127.3 | 1.8 | 36.6 | -1.3 | 0.98 |
| 3_2md95 | 127.3 | 129.2 | 1.9 | 37.7 | 1.1 | 1.12 |
| 3_2md96 | 129.2 | 129.8 | 0.6 | 35.8 | -1.9 | 0.42 |
| 3_2md97 | 129.8 | 130 | 0.2 | 37.9 | 2.1 | 0 |
| 3_2md98 | 130 | 131 | 1 | 36.4 | -1.5 | 0.7 |
| 3_2md99 | 131 | 131.5 | 0.5 | 38.8 | 2.4 | 1.21 |
| 3_2md100 | 131.5 | 131.7 | 0.2 | 43.1 | 4.4 | 0 |
| 3_2md101 | 131.7 | 135.7 | 4 | 38.3 | -4.8 | 1.44 |
| 3_2md102 | 135.7 | 136.5 | 0.8 | 36.3 | -2.0 | 0.83 |
| 3_2md103 | 136.5 | 137.2 | 0.7 | 37.7 | 1.3 | 0.95 |
| 3_2md104 | 137.2 | 137.7 | 0.5 | 36.1 | -1.6 | 0.6 |
| 3_2md105 | 137.7 | 139.3 | 1.6 | 38.5 | 2.4 | 0.87 |
| 3_2md106 | 139.3 | 141 | 1.7 | 35.9 | -2.6 | 0.72 |
| 3_2md107 | 141 | 141.7 | 0.7 | 37.5 | 1.6 | 1.68 |
| 3_2md108 | 141.7 | 142 | 0.3 | 35.8 | -1.6 | 0.31 |
| 3_2md109 | 142 | 142.7 | 0.7 | 38.2 | 2.4 | 0.55 |
| 3_2md110 | 142.7 | 143.9 | 1.2 | 36.0 | -2.1 | 0.44 |
| 3_2md111 | 143.9 | 144.1 | 0.2 | 37.5 | 1.5 | 0 |
| 3_2md112 | 144.1 | 144.8 | 0.7 | 36.3 | -1.2 | 0.8 |
| 3_2md113 | 144.8 | 145 | 0.2 | 37.3 | 1.0 | 0 |
| 3_2md114 | 145 | 145.3 | 0.3 | 36.9 | -0.4 | 0.1 |
| 3_2md115 | 145.3 | 145.5 | 0.2 | 37.2 | 0.3 | 0 |
| 3_2md116 | 145.5 | 146.1 | 0.6 | 36.0 | -1.2 | 1.4 |
| 3_2md117 | 146.1 | 147.5 | 1.4 | 38.7 | 2.8 | 1.08 |
| 3_2md118 | 147.5 | 148.4 | 0.9 | 36.4 | -2.3 | 0.55 |
| 3_2md119 | 148.4 | 148.6 | 0.2 | 37.2 | 0.8 | 0 |
| 3_2md120 | 148.6 | 149.2 | 0.6 | 36.4 | -0.8 | 0.89 |
| 3_2md121 | 149.2 | 149.5 | 0.3 | 37.4 | 1.0 | 0.32 |
| 3_2md122 | 149.5 | 154.5 | 5 | 35.4 | -2.0 | 0.87 |
| 3_2md123 | 154.5 | 154.8 | 0.3 | 37.4 | 2.0 | 0.27 |
| 3_2md124 | 154.8 | 155.9 | 1.1 | 36.2 | -1.1 | 0.39 |
| 3_2md125 | 155.9 | 156.8 | 0.9 | 38.3 | 2.1 | 0.81 |
| 3_2md126 | 156.8 | 158.4 | 1.6 | 36.3 | -2.0 | 1 |
| 3_2md127 | 158.4 | 159.3 | 0.9 | 39.2 | 2.9 | 1.42 |
| 3_2md128 | 159.3 | 159.5 | 0.2 | 42.0 | 2.8 | 0 |
| 3_2md129 | 159.5 | 160.9 | 1.4 | 37.9 | -4.0 | 1.22 |
| 3_2md130 | 160.9 | 161.7 | 0.8 | 36.5 | -1.4 | 0.4 |
| 3_2md131 | 161.7 | 162.4 | 0.7 | 39.5 | 3.0 | 1.03 |
| 3_2md132 | 162.4 | 162.7 | 0.3 | 42.3 | 2.8 | 0.24 |
| 3_2md133 | 162.7 | 163.5 | 0.8 | 40.0 | -2.2 | 1.01 |

| | | | | | | |
|---|---|---|---|---|---|---|
| 3_2md134 | 163.5 | 164.4 | 0.9 | 44.5 | 4.4 | 2.59 |
| 3_2md135 | 164.4 | 165.1 | 0.7 | 39.9 | -4.6 | 1.29 |
| 3_2md136 | 165.1 | 165.6 | 0.5 | 44.3 | 4.3 | 2.25 |
| 3_2md137 | 165.6 | 165.8 | 0.2 | 39.7 | -4.6 | 0 |
| 3_2md138 | 165.8 | 166.3 | 0.5 | 43.2 | 3.6 | 2.96 |
| 3_2md139 | 166.3 | 168.8 | 2.5 | 38.7 | -4.5 | 0.88 |
| 3_2md140 | 168.8 | 169.4 | 0.6 | 43.7 | 5.0 | 2.75 |
| 3_2md141 | 169.4 | 169.6 | 0.2 | 38.9 | -4.8 | 0 |
| 3_2md142 | 169.6 | 169.8 | 0.2 | 43.4 | 4.5 | 0 |
| 3_2md143 | 169.8 | 170 | 0.2 | 39.2 | -4.1 | 0 |
| 3_2md144 | 170 | 170.3 | 0.3 | 42.6 | 3.3 | 1.08 |
| 3_2md145 | 170.3 | 171.4 | 1.1 | 40.5 | -2.0 | 2 |
| 3_2md146 | 171.4 | 171.6 | 0.2 | 36.3 | -4.2 | 0 |
| 3_2md147 | 171.6 | 172.1 | 0.5 | 38.4 | 2.1 | 1.12 |
| 3_2md148 | 172.1 | 172.3 | 0.2 | 42.5 | 4.1 | 0 |
| 3_2md149 | 172.3 | 172.9 | 0.6 | 39.1 | -3.4 | 0.98 |
| 3_2md150 | 172.9 | 173.1 | 0.2 | 43.6 | 4.5 | 0 |
| 3_2md151 | 173.1 | 174.9 | 1.8 | 38.8 | -4.8 | 1.59 |
| 3_2md152 | 174.9 | 175.3 | 0.4 | 43.8 | 5.0 | 2.65 |
| 3_2md153 | 175.3 | 175.5 | 0.2 | 38.8 | -5.0 | 0 |
| 3_2md154 | 175.5 | 176.2 | 0.7 | 43.6 | 4.8 | 2.25 |
| 3_2md155 | 176.2 | 176.6 | 0.4 | 37.9 | -5.7 | 1.25 |
| 3_2md156 | 176.6 | 177.6 | 1 | 42.6 | 4.8 | 2.86 |
| 3_2md157 | 177.6 | 177.8 | 0.2 | 39.0 | -3.6 | 0 |
| 3_2md158 | 177.8 | 178 | 0.2 | 44.6 | 5.5 | 0 |
| 3_2md159 | 178 | 178.3 | 0.3 | 37.2 | -7.4 | 0.83 |
| 3_2md160 | 178.3 | 179.2 | 0.9 | 39.8 | 2.6 | 1.71 |
| 3_2md161 | 179.2 | 179.5 | 0.3 | 43.5 | 3.7 | 1.18 |
| 3_2md162 | 179.5 | 180.2 | 0.7 | 38.5 | -5.0 | 0.61 |
| 3_2md163 | 180.2 | 181.1 | 0.9 | 45.1 | 6.6 | 2.3 |
| 3_2md164 | 181.1 | 181.4 | 0.3 | 39.7 | -5.4 | 0.56 |
| 3_2md165 | 181.4 | 181.6 | 0.2 | 42.9 | 3.3 | 0 |
| 3_2md166 | 181.6 | 182 | 0.4 | 45.5 | 2.6 | 2.16 |
| 3_2md167 | 182 | 183.1 | 1.1 | 38.6 | -6.9 | 0.71 |
| 3_2md168 | 183.1 | 183.4 | 0.3 | 42.4 | 3.8 | 2.23 |
| 3_2md169 | 183.4 | 183.6 | 0.2 | 46.5 | 4.0 | 0 |
| 3_2md170 | 183.6 | 184 | 0.4 | 42.8 | -3.6 | 0.87 |
| 3_2md171 | 184 | 184.4 | 0.4 | 37.4 | -5.4 | 1.21 |
| 3_2md172 | 184.4 | 184.6 | 0.2 | 43.5 | 6.0 | 0 |
| 3_2md173 | 184.6 | 185.1 | 0.5 | 37.1 | -6.3 | 1.1 |
| 3_2md174 | 185.1 | 185.5 | 0.4 | 46.8 | 9.7 | 3.43 |
| 3_2md175 | 185.5 | 185.9 | 0.4 | 43.5 | -3.3 | 1.76 |
| 3_2md176 | 185.9 | 186.1 | 0.2 | 39.0 | -4.6 | 0 |
| 3_2md177 | 186.1 | 186.8 | 0.7 | 44.2 | 5.3 | 2.82 |
| 3_2md178 | 186.8 | 187 | 0.2 | 38.3 | -5.9 | 0 |
| 3_2md179 | 187 | 188.8 | 1.8 | 43.1 | 4.8 | 2.57 |
| 3_2md180 | 188.8 | 189.3 | 0.5 | 37.9 | -5.2 | 1.27 |
| 3_2md181 | 189.3 | 190 | 0.7 | 43.2 | 5.2 | 1.74 |
| 3_2md182 | 190 | 190.6 | 0.6 | 39.4 | -3.8 | 0.96 |
| 3_2md183 | 190.6 | 191.2 | 0.6 | 43.6 | 4.2 | 2.34 |
| 3_2md184 | 191.2 | 191.6 | 0.4 | 48.8 | 5.2 | 3.97 |
| 3_2md185 | 191.6 | 192 | 0.4 | 39.6 | -9.2 | 0.66 |
| 3_2md186 | 192 | 192.7 | 0.7 | 45.9 | 6.3 | 1.9 |
| 3_2md187 | 192.7 | 193.5 | 0.8 | 40.3 | -5.6 | 2.54 |
| 3_2md188 | 193.5 | 195.1 | 1.6 | 43.0 | 2.7 | 2.63 |
| 3_2md189 | 195.1 | 195.9 | 0.8 | 38.5 | -4.5 | 0.84 |

| | | | | | | |
|---|---|---|---|---|---|---|
| 3_2md190 | 195.9 | 196.1 | 0.2 | 42.5 | 4.0 | 0 |
| 3_2md191 | 196.1 | 197.1 | 1 | 39.4 | -3.1 | 1.21 |
| 3_2md192 | 197.1 | 197.3 | 0.2 | 43.2 | 3.8 | 0 |
| 3_2md193 | 197.3 | 197.5 | 0.2 | 39.8 | -3.4 | 0 |
| 3_2md194 | 197.5 | 198.3 | 0.8 | 43.2 | 3.4 | 2.86 |
| 3_2md195 | 198.3 | 198.6 | 0.3 | 40.1 | -3.1 | 0.83 |
| 3_2md196 | 198.6 | 198.9 | 0.3 | 41.8 | 1.7 | 0.33 |
| 3_2md197 | 198.9 | 199.2 | 0.3 | 40.3 | -1.5 | 0.25 |
| 3_2md198 | 199.2 | 199.5 | 0.3 | 50.0 | 9.7 | 2.78 |
| 3_2md199 | 199.5 | 200.1 | 0.6 | 42.9 | -7.1 | 0.78 |
| 3_2md200 | 200.1 | 201.2 | 1.1 | 40.0 | -2.9 | 1.4 |
| 3_2md201 | 201.2 | 201.4 | 0.2 | 45.3 | 5.3 | 0 |
| 3_2md202 | 201.4 | 202.5 | 1.1 | 38.6 | -6.7 | 1.12 |
| 3_2md203 | 202.5 | 202.7 | 0.2 | 36.4 | -2.1 | 0 |
| 3_2md204 | 202.7 | 207.8 | 5.1 | 38.8 | 2.3 | 1.09 |
| 3_2md205 | 207.8 | 208.5 | 0.7 | 36.6 | -2.2 | 0.45 |
| 3_2md206 | 208.5 | 209.1 | 0.6 | 39.1 | 2.4 | 1.19 |
| 3_2md207 | 209.1 | 209.5 | 0.4 | 41.7 | 2.6 | 0.39 |
| 3_2md208 | 209.5 | 209.7 | 0.2 | 39.8 | -1.9 | 0 |
| 3_2md209 | 209.7 | 211.2 | 1.5 | 43.9 | 4.1 | 1.9 |
| 3_2md210 | 211.2 | 211.8 | 0.6 | 38.4 | -5.5 | 1 |
| 3_2md211 | 211.8 | 212.1 | 0.3 | 42.8 | 4.5 | 2.05 |
| 3_2md212 | 212.1 | 213 | 0.9 | 38.9 | -3.9 | 2.2 |
| 3_2md213 | 213 | 213.4 | 0.4 | 35.8 | -3.1 | 0.29 |
| 3_2md214 | 213.4 | 213.9 | 0.5 | 38.8 | 3.0 | 1.29 |
| 3_2md215 | 213.9 | 214.1 | 0.2 | 42.2 | 3.4 | 0 |
| 3_2md216 | 214.1 | 214.8 | 0.7 | 38.4 | -3.8 | 1.8 |
| 3_2md217 | 214.8 | 215 | 0.2 | 42.8 | 4.4 | 0 |
| 3_2md218 | 215 | 215.4 | 0.4 | 39.0 | -3.8 | 1.46 |
| 3_2md219 | 215.4 | 215.7 | 0.3 | 45.8 | 6.8 | 2.44 |
| 3_2md220 | 215.7 | 217.3 | 1.6 | 39.2 | -6.6 | 1.25 |
| 3_2md221 | 217.3 | 217.9 | 0.6 | 44.2 | 5.0 | 2.11 |
| 3_2md222 | 217.9 | 218.6 | 0.7 | 40.6 | -3.6 | 0.44 |
| 3_2md223 | 218.6 | 220.2 | 1.6 | 43.8 | 3.3 | 2.88 |
| 3_2md224 | 220.2 | 220.6 | 0.4 | 37.9 | -6.0 | 1.02 |
| 3_2md225 | 220.6 | 220.8 | 0.2 | 41.1 | 3.2 | 0 |
| 3_2md226 | 220.8 | 221.3 | 0.5 | 47.2 | 6.1 | 2.89 |
| 3_2md227 | 221.3 | 222.1 | 0.8 | 41.2 | -6.0 | 1.65 |
| 3_2md228 | 222.1 | 222.5 | 0.4 | 43.2 | 2.0 | 1.69 |
| 3_2md229 | 222.5 | 222.7 | 0.2 | 57.1 | 14.0 | 0 |
| 3_2md230 | 222.7 | 223.8 | 1.1 | 44.1 | -13.0 | 2.01 |
| 3_2md231 | 223.8 | 224.3 | 0.5 | 40.8 | -3.3 | 1.24 |
| 3_2md232 | 224.3 | 224.9 | 0.6 | 44.4 | 3.6 | 1.8 |
| 3_2md233 | 224.9 | 225.4 | 0.5 | 37.9 | -6.5 | 2.26 |
| 3_2md234 | 225.4 | 225.9 | 0.5 | 41.2 | 3.4 | 1.29 |
| 3_2md235 | 225.9 | 226.1 | 0.2 | 39.7 | -1.6 | 0 |
| 3_2md236 | 226.1 | 227.5 | 1.4 | 41.9 | 2.2 | 1.48 |
| 3_2md237 | 227.5 | 229.5 | 2 | 39.1 | -2.8 | 0.88 |
| 3_2md238 | 229.5 | 230.3 | 0.8 | 42.5 | 3.4 | 1.67 |
| 3_2md239 | 230.3 | 230.6 | 0.3 | 48.5 | 6.0 | 2.73 |
| 3_2md240 | 230.6 | 231.6 | 1 | 39.7 | -8.8 | 1.36 |
| 3_2md241 | 231.6 | 232.9 | 1.3 | 43.5 | 3.7 | 1.83 |
| 3_2md242 | 232.9 | 233.2 | 0.3 | 40.2 | -3.2 | 0.53 |
| 3_2md243 | 233.2 | 233.8 | 0.6 | 41.9 | 1.6 | 0.68 |
| 3_2md244 | 233.8 | 234.1 | 0.3 | 48.7 | 6.8 | 2.69 |
| 3_2md245 | 234.1 | 236.7 | 2.6 | 43.2 | -5.5 | 1.35 |

| | | | | | | |
|---|---|---|---|---|---|---|
| 3_2md246 | 236.7 | 236.9 | 0.2 | 40.8 | -2.4 | 0 |
| 3_2md247 | 236.9 | 237.5 | 0.6 | 42.3 | 1.5 | 1.46 |
| 3_2md248 | 237.5 | 237.7 | 0.2 | 40.3 | -2.0 | 0 |
| 3_2md249 | 237.7 | 240.2 | 2.5 | 42.9 | 2.6 | 1.65 |
| 3_2md250 | 240.2 | 240.8 | 0.6 | 39.7 | -3.2 | 0.44 |
| 3_2md251 | 240.8 | 242.6 | 1.8 | 40.7 | 1.0 | 1.38 |
| 3_2md252 | 242.6 | 242.9 | 0.3 | 42.1 | 1.5 | 0.89 |
| 3_2md253 | 242.9 | 243.2 | 0.3 | 39.2 | -3.0 | 1.17 |
| 3_2md254 | 243.2 | 243.4 | 0.2 | 44.4 | 5.2 | 0 |
| 3_2md255 | 243.4 | 243.9 | 0.5 | 48.1 | 3.8 | 1.58 |
| 3_2md256 | 243.9 | 244.5 | 0.6 | 44.7 | -3.5 | 3.58 |
| 3_2md257 | 244.5 | 245.8 | 1.3 | 49.5 | 4.8 | 3.22 |
| 3_2md258 | 245.8 | 246.2 | 0.4 | 44.2 | -5.3 | 1.11 |
| 3_2md259 | 246.2 | 246.7 | 0.5 | 47.5 | 3.3 | 2.81 |
| 3_2md260 | 246.7 | 246.9 | 0.2 | 44.3 | -3.2 | 0 |
| 3_2md261 | 246.9 | 247.3 | 0.4 | 48.7 | 4.4 | 2.04 |
| 3_2md262 | 247.3 | 247.5 | 0.2 | 43.5 | -5.2 | 0 |
| 3_2md263 | 247.5 | 247.7 | 0.2 | 49.0 | 5.5 | 0 |
| 3_2md264 | 247.7 | 248.1 | 0.4 | 41.0 | -8.0 | 0.71 |
| 3_2md265 | 248.1 | 248.3 | 0.2 | 42.7 | 1.7 | 0 |
| 3_2md266 | 248.3 | 249.3 | 1 | 50.2 | 7.5 | 3.57 |
| 3_2md267 | 249.3 | 250.2 | 0.9 | 40.4 | -9.8 | 0.83 |
| 3_2md268 | 250.2 | 251 | 0.8 | 43.9 | 3.5 | 3.19 |
| 3_2md269 | 251 | 251.5 | 0.5 | 47.7 | 3.8 | 3.37 |
| 3_2md270 | 251.5 | 252.1 | 0.6 | 44.0 | -3.7 | 1.28 |
| 3_2md271 | 252.1 | 252.5 | 0.4 | 49.3 | 5.2 | 1.81 |
| 3_2md272 | 252.5 | 252.7 | 0.2 | 42.8 | -6.5 | 0 |
| 3_2md273 | 252.7 | 252.9 | 0.2 | 51.7 | 8.9 | 0 |
| 3_2md274 | 252.9 | 253.5 | 0.6 | 41.3 | -10.4 | 1.73 |
| 3_2md275 | 253.5 | 254 | 0.5 | 48.5 | 7.1 | 3.02 |
| 3_2md276 | 254 | 254.6 | 0.6 | 40.8 | -7.7 | 3.56 |
| 3_2md277 | 254.6 | 256.3 | 1.7 | 43.0 | 2.2 | 2.49 |
| 3_2md278 | 256.3 | 256.5 | 0.2 | 40.2 | -2.8 | 0 |
| 3_2md279 | 256.5 | 256.9 | 0.4 | 42.2 | 1.9 | 0.19 |
| 3_2md280 | 256.9 | 257.1 | 0.2 | 37.6 | -4.5 | 0 |
| 3_2md281 | 257.1 | 257.3 | 0.2 | 42.2 | 4.6 | 0 |
| 3_2md282 | 257.3 | 257.5 | 0.2 | 40.5 | -1.7 | 0 |
| 3_2md283 | 257.5 | 257.8 | 0.3 | 42.2 | 1.6 | 0.52 |
| 3_2md284 | 257.8 | 258.1 | 0.3 | 38.5 | -3.7 | 1.27 |
| 3_2md285 | 258.1 | 258.7 | 0.6 | 43.5 | 5.0 | 1.59 |
| 3_2md286 | 258.7 | 258.9 | 0.2 | 46.5 | 3.0 | 0 |
| 3_2md287 | 258.9 | 259.1 | 0.2 | 45.3 | -1.2 | 0 |
| 3_2md288 | 259.1 | 259.3 | 0.2 | 46.3 | 1.0 | 0 |
| 3_2md289 | 259.3 | 259.6 | 0.3 | 38.4 | -7.9 | 0.94 |
| 3_2md290 | 259.6 | 259.8 | 0.2 | 42.0 | 3.6 | 0 |
| 3_2md291 | 259.8 | 260.4 | 0.6 | 39.4 | -2.6 | 1.77 |
| 3_2md292 | 260.4 | 260.6 | 0.2 | 42.0 | 2.6 | 0 |
| 3_2md293 | 260.6 | 261.5 | 0.9 | 40.2 | -1.8 | 0.96 |
| 3_2md294 | 261.5 | 261.7 | 0.2 | 36.7 | -3.5 | 0 |
| 3_2md295 | 261.7 | 261.9 | 0.2 | 37.5 | 0.8 | 0 |
| 3_2md296 | 261.9 | 262.2 | 0.3 | 47.4 | 9.9 | 3.42 |
| 3_2md297 | 262.2 | 262.4 | 0.2 | 55.3 | 7.9 | 0 |
| | | | | | | |
| 4_1md1 | 0 | 0.3 | 0.3 | 39.3 | | 1.64 |
| 4_1md2 | 0.3 | 0.5 | 0.2 | 43.5 | 4.2 | 0 |
| 4_1md3 | 0.5 | 0.7 | 0.2 | 39.5 | -4.0 | 0 |

| | | | | | | |
|---|---|---|---|---|---|---|
| 4_1md4 | 0.7 | 1.7 | 1 | 43.2 | 3.7 | 2.24 |
| 4_1md5 | 1.7 | 1.9 | 0.2 | 38.2 | -5.0 | 0 |
| 4_1md6 | 1.9 | 3 | 1.1 | 35.3 | -2.9 | 0.91 |
| 4_1md7 | 3 | 3.4 | 0.4 | 38.7 | 3.4 | 0.87 |
| 4_1md8 | 3.4 | 3.8 | 0.4 | 40.8 | 2.1 | 1.68 |
| 4_1md9 | 3.8 | 4.2 | 0.4 | 38.2 | -2.6 | 1.31 |
| 4_1md10 | 4.2 | 4.4 | 0.2 | 36.7 | -1.5 | 0 |
| 4_1md11 | 4.4 | 6.4 | 2 | 38.2 | 1.5 | 1.55 |
| 4_1md12 | 6.4 | 7 | 0.6 | 40.8 | 2.7 | 2.43 |
| 4_1md13 | 7 | 8.3 | 1.3 | 38.6 | -2.2 | 0.77 |
| 4_1md14 | 8.3 | 8.6 | 0.3 | 36.2 | -2.4 | 0.46 |
| 4_1md15 | 8.6 | 10.4 | 1.8 | 39.0 | 2.8 | 1.67 |
| 4_1md16 | 10.4 | 10.7 | 0.3 | 36.4 | -2.6 | 0.14 |
| 4_1md17 | 10.7 | 11.4 | 0.7 | 38.0 | 1.6 | 1.01 |
| 4_1md18 | 11.4 | 12.5 | 1.1 | 35.9 | -2.1 | 0.83 |
| 4_1md19 | 12.5 | 13.9 | 1.4 | 38.0 | 2.2 | 1.45 |
| 4_1md20 | 13.9 | 14.1 | 0.2 | 36.4 | -1.6 | 0 |
| 4_1md21 | 14.1 | 16.9 | 2.8 | 38.1 | 1.7 | 0.95 |
| 4_1md22 | 16.9 | 18.3 | 1.4 | 36.4 | -1.7 | 1.35 |
| 4_1md23 | 18.3 | 19.3 | 1 | 37.0 | 0.6 | 0.59 |
| 4_1md24 | 19.3 | 21.2 | 1.9 | 36.2 | -0.8 | 0.71 |
| 4_1md25 | 21.2 | 21.5 | 0.3 | 37.7 | 1.5 | 0.48 |
| 4_1md26 | 21.5 | 22.1 | 0.6 | 36.3 | -1.5 | 0.64 |
| 4_1md27 | 22.1 | 22.5 | 0.4 | 38.1 | 1.8 | 0.94 |
| 4_1md28 | 22.5 | 22.9 | 0.4 | 36.2 | -1.8 | 1.63 |
| 4_1md29 | 22.9 | 23.9 | 1 | 38.6 | 2.4 | 1.6 |
| 4_1md30 | 23.9 | 24.1 | 0.2 | 43.3 | 4.7 | 0 |
| 4_1md31 | 24.1 | 26.1 | 2 | 39.4 | -3.9 | 1.15 |
| 4_1md32 | 26.1 | 26.3 | 0.2 | 36.4 | -2.9 | 0 |
| 4_1md33 | 26.3 | 26.9 | 0.6 | 37.1 | 0.6 | 0.97 |
| 4_1md34 | 26.9 | 33.9 | 7 | 34.8 | -2.3 | 1.21 |
| 4_1md35 | 33.9 | 34.4 | 0.5 | 36.6 | 1.8 | 1.24 |
| 4_1md36 | 34.4 | 41.8 | 7.4 | 34.9 | -1.7 | 1.25 |
| 4_1md37 | 41.8 | 42 | 0.2 | 38.5 | 3.6 | 0 |
| 4_1md38 | 42 | 42.2 | 0.2 | 36.5 | -2.0 | 0 |
| 4_1md39 | 42.2 | 42.4 | 0.2 | 37.6 | 1.1 | 0 |
| 4_1md40 | 42.4 | 43 | 0.6 | 36.0 | -1.7 | 0.88 |
| 4_1md41 | 43 | 43.4 | 0.4 | 38.1 | 2.2 | 0.92 |
| 4_1md42 | 43.4 | 45.6 | 2.2 | 35.6 | -2.5 | 0.67 |
| 4_1md43 | 45.6 | 45.9 | 0.3 | 37.2 | 1.6 | 0.08 |
| 4_1md44 | 45.9 | 47.5 | 1.6 | 35.8 | -1.4 | 1.21 |
| 4_1md45 | 47.5 | 48.1 | 0.6 | 37.1 | 1.4 | 0.39 |
| 4_1md46 | 48.1 | 48.3 | 0.2 | 35.6 | -1.6 | 0 |
| 4_1md47 | 48.3 | 48.6 | 0.3 | 37.6 | 2.0 | 0.18 |
| 4_1md48 | 48.6 | 49 | 0.4 | 36.7 | -0.9 | 0.33 |
| 4_1md49 | 49 | 49.4 | 0.4 | 37.9 | 1.3 | 1.79 |
| 4_1md50 | 49.4 | 50.7 | 1.3 | 35.5 | -2.4 | 1.3 |
| 4_1md51 | 50.7 | 53.6 | 2.9 | 0.0 | -35.5 | 0 |
| 4_1md52 | 53.6 | 61.6 | 8 | 35.2 | 35.2 | 1.16 |
| 4_1md53 | 61.6 | 62.4 | 0.8 | 37.7 | 2.5 | 0.18 |
| 4_1md54 | 62.4 | 62.7 | 0.3 | 35.6 | -2.2 | 0.77 |
| 4_1md55 | 62.7 | 62.9 | 0.2 | 37.9 | 2.3 | 0 |
| 4_1md56 | 62.9 | 63.5 | 0.6 | 36.5 | -1.4 | 0.72 |
| 4_1md57 | 63.5 | 64.4 | 0.9 | 38.1 | 1.7 | 0.99 |
| 4_1md58 | 64.4 | 64.6 | 0.2 | 36.3 | -1.8 | 0 |
| 4_1md59 | 64.6 | 65.1 | 0.5 | 37.7 | 1.4 | 0.47 |

| | | | | | | |
|---|---|---|---|---|---|---|
| 4_1md60 | 65.1 | 67 | 1.9 | 36.4 | -1.4 | 0.73 |
| 4_1md61 | 67 | 67.8 | 0.8 | 37.9 | 1.5 | 1.11 |
| 4_1md62 | 67.8 | 68 | 0.2 | 36.5 | -1.4 | 0 |
| 4_1md63 | 68 | 68.8 | 0.8 | 37.7 | 1.2 | 0.75 |
| 4_1md64 | 68.8 | 69 | 0.2 | 36.3 | -1.4 | 0 |
| 4_1md65 | 69 | 69.6 | 0.6 | 37.9 | 1.6 | 0.56 |
| 4_1md66 | 69.6 | 72.5 | 2.9 | 36.0 | -2.0 | 0.91 |
| 4_1md67 | 72.5 | 76.6 | 4.1 | 38.3 | 2.3 | 1.42 |
| 4_1md68 | 76.6 | 81.8 | 5.2 | 35.9 | -2.4 | 0.82 |
| 4_1md69 | 81.8 | 83.6 | 1.8 | 38.5 | 2.5 | 1.19 |
| 4_1md70 | 83.6 | 83.9 | 0.3 | 35.8 | -2.6 | 0.85 |
| 4_1md71 | 83.9 | 85 | 1.1 | 38.9 | 3.1 | 0.85 |
| 4_1md72 | 85 | 85.4 | 0.4 | 35.6 | -3.3 | 0.5 |
| 4_1md73 | 85.4 | 86.9 | 1.5 | 38.4 | 2.8 | 1.05 |
| 4_1md74 | 86.9 | 87.2 | 0.3 | 36.5 | -1.8 | 0.45 |
| 4_1md75 | 87.2 | 87.6 | 0.4 | 37.4 | 0.9 | 0.66 |
| 4_1md76 | 87.6 | 87.9 | 0.3 | 42.3 | 4.9 | 1.13 |
| 4_1md77 | 87.9 | 88.3 | 0.4 | 38.8 | -3.6 | 1.08 |
| 4_1md78 | 88.3 | 88.7 | 0.4 | 41.9 | 3.2 | 0.45 |
| 4_1md79 | 88.7 | 91 | 2.3 | 39.9 | -2.0 | 1.28 |
| 4_1md80 | 91 | 91.3 | 0.3 | 42.5 | 2.6 | 1.17 |
| 4_1md81 | 91.3 | 91.6 | 0.3 | 37.6 | -4.9 | 2.83 |
| 4_1md82 | 91.6 | 91.8 | 0.2 | 36.5 | -1.1 | 0 |
| 4_1md83 | 91.8 | 92.1 | 0.3 | 38.2 | 1.7 | 0.63 |
| 4_1md84 | 92.1 | 92.3 | 0.2 | 36.0 | -2.1 | 0 |
| 4_1md85 | 92.3 | 96.7 | 4.4 | 38.8 | 2.8 | 1.45 |
| 4_1md86 | 96.7 | 97.9 | 1.2 | 36.4 | -2.4 | 0.52 |
| 4_1md87 | 97.9 | 98.2 | 0.3 | 37.7 | 1.2 | 0.55 |
| 4_1md88 | 98.2 | 98.8 | 0.6 | 34.9 | -2.7 | 1.12 |
| 4_1md89 | 98.8 | 99 | 0.2 | 37.8 | 2.9 | 0 |
| 4_1md90 | 99 | 100.5 | 1.5 | 36.1 | -1.7 | 0.88 |
| 4_1md91 | 100.5 | 101 | 0.5 | 38.2 | 2.1 | 1.08 |
| 4_1md92 | 101 | 101.2 | 0.2 | 35.9 | -2.4 | 0 |
| 4_1md93 | 101.2 | 102.5 | 1.3 | 37.9 | 2.0 | 0.93 |
| 4_1md94 | 102.5 | 103.7 | 1.2 | 36.5 | -1.4 | 0.51 |
| 4_1md95 | 103.7 | 106.5 | 2.8 | 38.5 | 2.0 | 1.35 |
| 4_1md96 | 106.5 | 108.6 | 2.1 | 35.7 | -2.8 | 0.85 |
| 4_1md97 | 108.6 | 110.3 | 1.7 | 38.1 | 2.4 | 1.67 |
| 4_1md98 | 110.3 | 110.5 | 0.2 | 43.3 | 5.2 | 0 |
| 4_1md99 | 110.5 | 112.1 | 1.6 | 38.6 | -4.7 | 1.66 |
| 4_1md100 | 112.1 | 118.6 | 6.5 | 35.4 | -3.2 | 1.26 |
| 4_1md101 | 118.6 | 120.7 | 2.1 | 39.4 | 4.0 | 1.41 |
| 4_1md102 | 120.7 | 121.1 | 0.4 | 35.7 | -3.7 | 1.09 |
| 4_1md103 | 121.1 | 124.1 | 3 | 38.1 | 2.4 | 1.36 |
| 4_1md104 | 124.1 | 129 | 4.9 | 35.1 | -3.0 | 1.15 |
| 4_1md105 | 129 | 129.4 | 0.4 | 37.5 | 2.4 | 0.22 |
| 4_1md106 | 129.4 | 130.4 | 1 | 35.3 | -2.2 | 0.76 |
| 4_1md107 | 130.4 | 131 | 0.6 | 37.8 | 2.6 | 0.57 |
| 4_1md108 | 131 | 131.7 | 0.7 | 36.7 | -1.2 | 0.96 |
| 4_1md109 | 131.7 | 132.4 | 0.7 | 37.2 | 0.6 | 0.7 |
| 4_1md110 | 132.4 | 132.7 | 0.3 | 35.4 | -1.8 | 0.85 |
| 4_1md111 | 132.7 | 134 | 1.3 | 38.3 | 2.9 | 1.38 |
| 4_1md112 | 134 | 148.9 | 14.9 | 35.0 | -3.3 | 1 |
| 4_1md113 | 148.9 | 149.1 | 0.2 | 37.5 | 2.5 | 0 |
| 4_1md114 | 149.1 | 150 | 0.9 | 35.3 | -2.3 | 1.18 |
| 4_1md115 | 150 | 150.2 | 0.2 | 39.3 | 4.0 | 0 |

| | | | | | | |
|---|---|---|---|---|---|---|
| 4_1md116 | 150.2 | 150.4 | 0.2 | 35.1 | -4.2 | 0 |
| 4_1md117 | 150.4 | 151.1 | 0.7 | 37.2 | 2.1 | 0.46 |
| 4_1md118 | 151.1 | 154.7 | 3.6 | 36.3 | -0.9 | 0.72 |
| 4_1md119 | 154.7 | 154.9 | 0.2 | 37.7 | 1.4 | 0 |
| 4_1md120 | 154.9 | 155.5 | 0.6 | 36.5 | -1.2 | 0.52 |
| 4_1md121 | 155.5 | 155.8 | 0.3 | 37.5 | 1.0 | 0.67 |
| 4_1md122 | 155.8 | 162.8 | 7 | 35.2 | -2.3 | 1.06 |
| 4_1md123 | 162.8 | 163.3 | 0.5 | 37.4 | 2.2 | 0.6 |
| 4_1md124 | 163.3 | 163.8 | 0.5 | 35.2 | -2.2 | 1.41 |
| 4_1md125 | 163.8 | 164.4 | 0.6 | 38.6 | 3.4 | 0.75 |
| 4_1md126 | 164.4 | 172.7 | 8.3 | 35.5 | -3.1 | 0.95 |
| 4_1md127 | 172.7 | 173.1 | 0.4 | 37.4 | 1.9 | 0.53 |
| 4_1md128 | 173.1 | 177 | 3.9 | 35.2 | -2.1 | 0.74 |
| 4_1md129 | 177 | 177.6 | 0.6 | 38.2 | 3.0 | 1.23 |
| 4_1md130 | 177.6 | 178.2 | 0.6 | 36.2 | -2.0 | 1.3 |
| 4_1md131 | 178.2 | 178.4 | 0.2 | 37.6 | 1.4 | 0 |
| 4_1md132 | 178.4 | 179.9 | 1.5 | 35.8 | -1.8 | 1.07 |
| 4_1md133 | 179.9 | 181.1 | 1.2 | 37.7 | 1.9 | 0.81 |
| 4_1md134 | 181.1 | 181.6 | 0.5 | 36.1 | -1.6 | 1.35 |
| 4_1md135 | 181.6 | 181.8 | 0.2 | 37.7 | 1.5 | 0 |
| 4_1md136 | 181.8 | 182.2 | 0.4 | 36.1 | -1.6 | 0.21 |
| 4_1md137 | 182.2 | 182.8 | 0.6 | 38.3 | 2.2 | 0.76 |
| 4_1md138 | 182.8 | 183.4 | 0.6 | 36.4 | -1.9 | 0.29 |
| 4_1md139 | 183.4 | 183.6 | 0.2 | 37.4 | 1.0 | 0 |
| 4_1md140 | 183.6 | 183.9 | 0.3 | 36.1 | -1.3 | 0.7 |
| 4_1md141 | 183.9 | 184.9 | 1 | 38.0 | 1.9 | 1.16 |
| 4_1md142 | 184.9 | 185.1 | 0.2 | 34.8 | -3.2 | 0 |
| 4_1md143 | 185.1 | 185.9 | 0.8 | 37.7 | 3.0 | 0.93 |
| 4_1md144 | 185.9 | 186.5 | 0.6 | 36.3 | -1.4 | 0.87 |
| 4_1md145 | 186.5 | 187.2 | 0.7 | 37.3 | 1.0 | 0.41 |
| 4_1md146 | 187.2 | 187.4 | 0.2 | 35.1 | -2.2 | 0 |
| 4_1md147 | 187.4 | 187.6 | 0.2 | 40.8 | 5.7 | 0 |
| 4_1md148 | 187.6 | 187.8 | 0.2 | 36.2 | -4.6 | 0 |
| 4_1md149 | 187.8 | 189.3 | 1.5 | 37.8 | 1.6 | 0.68 |
| 4_1md150 | 189.3 | 189.5 | 0.2 | 35.4 | -2.4 | 0 |
| 4_1md151 | 189.5 | 189.8 | 0.3 | 38.0 | 2.6 | 0.5 |
| 4_1md152 | 189.8 | 190.6 | 0.8 | 36.4 | -1.6 | 0.64 |
| 4_1md153 | 190.6 | 190.8 | 0.2 | 39.1 | 2.7 | 0 |
| 4_1md154 | 190.8 | 207.3 | 16.5 | 35.2 | -3.9 | 1.18 |
| 4_1md155 | 207.3 | 208.1 | 0.8 | 37.8 | 2.5 | 0.5 |
| 4_1md156 | 208.1 | 208.4 | 0.3 | 36.1 | -1.7 | 0.2 |
| 4_1md157 | 208.4 | 209.2 | 0.8 | 37.8 | 1.7 | 0.36 |
| 4_1md158 | 209.2 | 218.2 | 9 | 35.1 | -2.7 | 0.87 |
| 4_1md159 | 218.2 | 218.8 | 0.6 | 37.2 | 2.1 | 0.72 |
| 4_1md160 | 218.8 | 221.6 | 2.8 | 35.6 | -1.6 | 0.98 |
| 4_1md161 | 221.6 | 221.8 | 0.2 | 37.8 | 2.2 | 0 |
| 4_1md162 | 221.8 | 224.1 | 2.3 | 36.5 | -1.3 | 0.95 |
| 4_1md163 | 224.1 | 224.8 | 0.7 | 39.5 | 3.0 | 0.92 |
| 4_1md164 | 224.8 | 225.1 | 0.3 | 41.9 | 2.5 | 0.78 |
| 4_1md165 | 225.1 | 226.3 | 1.2 | 38.9 | -3.0 | 1.89 |
| | | | | | | |
| 4_2md1 | 0 | 2.3 | 2.3 | 39.0 | | 1.27 |
| 4_2md2 | 2.3 | 2.5 | 0.2 | 35.8 | -3.2 | 0 |
| 4_2md3 | 2.5 | 3.6 | 1.1 | 40.7 | 4.9 | 1.11 |
| 4_2md4 | 3.6 | 4 | 0.4 | 42.9 | 2.1 | 0.73 |
| 4_2md5 | 4 | 4.2 | 0.2 | 40.0 | -2.9 | 0 |

| | | | | | | |
|---|---|---|---|---|---|---|
| 4_2md6 | 4.2 | 5.2 | 1 | 42.3 | 2.3 | 3.56 |
| 4_2md7 | 5.2 | 6.4 | 1.2 | 39.3 | -3.0 | 1.22 |
| 4_2md8 | 6.4 | 7.3 | 0.9 | 41.9 | 2.6 | 0.87 |
| 4_2md9 | 7.3 | 7.8 | 0.5 | 38.3 | -3.6 | 2.53 |
| 4_2md10 | 7.8 | 8.2 | 0.4 | 41.6 | 3.2 | 0.63 |
| 4_2md11 | 8.2 | 8.4 | 0.2 | 39.8 | -1.8 | 0 |
| 4_2md12 | 8.4 | 8.6 | 0.2 | 41.4 | 1.5 | 0 |
| 4_2md13 | 8.6 | 9.5 | 0.9 | 39.3 | -2.1 | 0.99 |
| 4_2md14 | 9.5 | 9.7 | 0.2 | 41.8 | 2.5 | 0 |
| 4_2md15 | 9.7 | 10.7 | 1 | 38.3 | -3.4 | 1.13 |
| 4_2md16 | 10.7 | 11.3 | 0.6 | 36.4 | -1.9 | 0.4 |
| 4_2md17 | 11.3 | 11.7 | 0.4 | 38.3 | 1.9 | 0.89 |
| 4_2md18 | 11.7 | 11.9 | 0.2 | 41.6 | 3.3 | 0 |
| 4_2md19 | 11.9 | 13 | 1.1 | 39.4 | -2.2 | 1.15 |
| 4_2md20 | 13 | 13.4 | 0.4 | 36.0 | -3.4 | 0.84 |
| 4_2md21 | 13.4 | 17.1 | 3.7 | 38.2 | 2.2 | 1.05 |
| 4_2md22 | 17.1 | 17.6 | 0.5 | 36.2 | -2.0 | 0.89 |
| 4_2md23 | 17.6 | 17.8 | 0.2 | 37.3 | 1.0 | 0 |
| 4_2md24 | 17.8 | 18.7 | 0.9 | 35.7 | -1.6 | 0.6 |
| 4_2md25 | 18.7 | 19.1 | 0.4 | 37.2 | 1.5 | 0.64 |
| 4_2md26 | 19.1 | 19.5 | 0.4 | 35.5 | -1.6 | 0.73 |
| 4_2md27 | 19.5 | 19.7 | 0.2 | 38.1 | 2.6 | 0 |
| 4_2md28 | 19.7 | 20.1 | 0.4 | 35.6 | -2.6 | 0.88 |
| 4_2md29 | 20.1 | 20.4 | 0.3 | 37.6 | 2.0 | 0.12 |
| 4_2md30 | 20.4 | 20.8 | 0.4 | 35.9 | -1.7 | 0.87 |
| 4_2md31 | 20.8 | 21 | 0.2 | 37.5 | 1.5 | 0 |
| 4_2md32 | 21 | 30.1 | 9.1 | 34.8 | -2.6 | 0.93 |
| 4_2md33 | 30.1 | 30.5 | 0.4 | 38.2 | 3.3 | 0.99 |
| 4_2md34 | 30.5 | 35.8 | 5.3 | 35.8 | -2.3 | 1.04 |
| 4_2md35 | 35.8 | 36.4 | 0.6 | 38.1 | 2.2 | 0.9 |
| 4_2md36 | 36.4 | 36.6 | 0.2 | 36.0 | -2.1 | 0 |
| 4_2md37 | 36.6 | 39.2 | 2.6 | 37.8 | 1.8 | 0.98 |
| 4_2md38 | 39.2 | 39.5 | 0.3 | 36.2 | -1.6 | 0.62 |
| 4_2md39 | 39.5 | 39.9 | 0.4 | 38.4 | 2.2 | 1.8 |
| 4_2md40 | 39.9 | 40.2 | 0.3 | 36.2 | -2.2 | 0.89 |
| 4_2md41 | 40.2 | 40.8 | 0.6 | 37.8 | 1.6 | 0.68 |
| 4_2md42 | 40.8 | 41.2 | 0.4 | 36.3 | -1.5 | 0.23 |
| 4_2md43 | 41.2 | 41.8 | 0.6 | 37.5 | 1.2 | 0.52 |
| 4_2md44 | 41.8 | 45.5 | 3.7 | 35.3 | -2.2 | 0.81 |
| 4_2md45 | 45.5 | 46.8 | 1.3 | 39.1 | 3.8 | 1.71 |
| 4_2md46 | 46.8 | 47.4 | 0.6 | 36.3 | -2.8 | 0.83 |
| 4_2md47 | 47.4 | 48.5 | 1.1 | 38.9 | 2.6 | 1.99 |
| 4_2md48 | 48.5 | 48.7 | 0.2 | 41.7 | 2.8 | 0 |
| 4_2md49 | 48.7 | 49.6 | 0.9 | 38.9 | -2.8 | 1.13 |
| 4_2md50 | 49.6 | 51.4 | 1.8 | 36.2 | -2.7 | 0.89 |
| 4_2md51 | 51.4 | 52.6 | 1.2 | 38.1 | 1.9 | 0.98 |
| 4_2md52 | 52.6 | 53 | 0.4 | 35.3 | -2.7 | 0.49 |
| 4_2md53 | 53 | 56.6 | 3.6 | 38.1 | 2.7 | 1.1 |
| 4_2md54 | 56.6 | 56.8 | 0.2 | 35.9 | -2.2 | 0 |
| 4_2md55 | 56.8 | 57.7 | 0.9 | 37.4 | 1.5 | 0.56 |
| 4_2md56 | 57.7 | 58.3 | 0.6 | 36.5 | -0.9 | 0.55 |
| 4_2md57 | 58.3 | 58.5 | 0.2 | 37.5 | 1.0 | 0 |
| 4_2md58 | 58.5 | 58.7 | 0.2 | 36.5 | -1.0 | 0 |
| 4_2md59 | 58.7 | 59.9 | 1.2 | 38.7 | 2.2 | 1.68 |
| 4_2md60 | 59.9 | 60.1 | 0.2 | 36.2 | -2.6 | 0 |
| 4_2md61 | 60.1 | 62 | 1.9 | 39.1 | 3.0 | 1.75 |

| | | | | | | |
|---|---|---|---|---|---|---|
| 4_2md62 | 62 | 62.9 | 0.9 | 35.7 | -3.5 | 1.52 |
| 4_2md63 | 62.9 | 63.7 | 0.8 | 37.2 | 1.5 | 1.11 |
| 4_2md64 | 63.7 | 64.1 | 0.4 | 36.5 | -0.6 | 1.47 |
| 4_2md65 | 64.1 | 64.3 | 0.2 | 37.2 | 0.7 | 0 |
| 4_2md66 | 64.3 | 68 | 3.7 | 35.4 | -1.8 | 0.94 |
| 4_2md67 | 68 | 68.6 | 0.6 | 37.6 | 2.1 | 0.79 |
| 4_2md68 | 68.6 | 68.8 | 0.2 | 35.4 | -2.1 | 0 |
| 4_2md69 | 68.8 | 69 | 0.2 | 38.7 | 3.3 | 0 |
| 4_2md70 | 69 | 69.6 | 0.6 | 35.9 | -2.8 | 0.57 |
| 4_2md71 | 69.6 | 69.8 | 0.2 | 37.9 | 2.0 | 0 |
| 4_2md72 | 69.8 | 70.3 | 0.5 | 35.9 | -1.9 | 0.78 |
| 4_2md73 | 70.3 | 71 | 0.7 | 37.9 | 2.0 | 0.72 |
| 4_2md74 | 71 | 71.3 | 0.3 | 36.6 | -1.4 | 0.22 |
| 4_2md75 | 71.3 | 71.6 | 0.3 | 38.0 | 1.4 | 0.49 |
| 4_2md76 | 71.6 | 72 | 0.4 | 36.4 | -1.6 | 0.78 |
| 4_2md77 | 72 | 72.5 | 0.5 | 38.4 | 2.0 | 1.64 |
| 4_2md78 | 72.5 | 72.9 | 0.4 | 36.5 | -1.8 | 1.78 |
| 4_2md79 | 72.9 | 74.3 | 1.4 | 38.0 | 1.4 | 1.06 |
| 4_2md80 | 74.3 | 74.5 | 0.2 | 36.1 | -1.9 | 0 |
| 4_2md81 | 74.5 | 76.7 | 2.2 | 38.2 | 2.2 | 1.09 |
| 4_2md82 | 76.7 | 78.4 | 1.7 | 36.2 | -2.1 | 0.96 |
| 4_2md83 | 78.4 | 78.7 | 0.3 | 38.8 | 2.6 | 0.41 |
| 4_2md84 | 78.7 | 79 | 0.3 | 36.4 | -2.4 | 0.19 |
| 4_2md85 | 79 | 79.2 | 0.2 | 37.2 | 0.8 | 0 |
| 4_2md86 | 79.2 | 83.2 | 4 | 35.7 | -1.5 | 1.06 |
| 4_2md87 | 83.2 | 83.5 | 0.3 | 37.2 | 1.6 | 0.11 |
| 4_2md88 | 83.5 | 88 | 4.5 | 35.8 | -1.4 | 0.92 |
| 4_2md89 | 88 | 91 | 3 | 37.7 | 1.9 | 0.98 |
| 4_2md90 | 91 | 91.3 | 0.3 | 36.5 | -1.2 | 0.14 |
| 4_2md91 | 91.3 | 91.9 | 0.6 | 37.3 | 0.8 | 0.73 |
| 4_2md92 | 91.9 | 92.1 | 0.2 | 36.3 | -1.0 | 0 |
| 4_2md93 | 92.1 | 92.7 | 0.6 | 37.3 | 1.0 | 1.04 |
| 4_2md94 | 92.7 | 93.5 | 0.8 | 36.2 | -1.1 | 0.95 |
| 4_2md95 | 93.5 | 93.8 | 0.3 | 39.2 | 3.0 | 1.5 |
| 4_2md96 | 93.8 | 94 | 0.2 | 35.4 | -3.8 | 0 |
| 4_2md97 | 94 | 94.3 | 0.3 | 37.6 | 2.3 | 0.51 |
| 4_2md98 | 94.3 | 96.3 | 2 | 36.2 | -1.4 | 0.64 |
| 4_2md99 | 96.3 | 97.2 | 0.9 | 38.6 | 2.4 | 1.28 |
| 4_2md100 | 97.2 | 97.6 | 0.4 | 35.3 | -3.4 | 1.32 |
| 4_2md101 | 97.6 | 98.8 | 1.2 | 38.0 | 2.8 | 1.03 |
| 4_2md102 | 98.8 | 99 | 0.2 | 34.8 | -3.2 | 0 |
| 4_2md103 | 99 | 99.3 | 0.3 | 38.1 | 3.3 | 0.14 |
| 4_2md104 | 99.3 | 101.5 | 2.2 | 36.0 | -2.1 | 0.64 |
| 4_2md105 | 101.5 | 103.2 | 1.7 | 38.2 | 2.1 | 0.73 |
| 4_2md106 | 103.2 | 106.3 | 3.1 | 35.1 | -3.1 | 1.04 |
| 4_2md107 | 106.3 | 108.1 | 1.8 | 38.5 | 3.5 | 1.31 |
| 4_2md108 | 108.1 | 108.4 | 0.3 | 42.3 | 3.8 | 0.44 |
| 4_2md109 | 108.4 | 108.7 | 0.3 | 37.9 | -4.4 | 2.6 |
| 4_2md110 | 108.7 | 109 | 0.3 | 42.1 | 4.1 | 0.47 |
| 4_2md111 | 109 | 110.2 | 1.2 | 38.6 | -3.4 | 1.62 |
| 4_2md112 | 110.2 | 110.5 | 0.3 | 41.3 | 2.7 | 0.19 |
| 4_2md113 | 110.5 | 112 | 1.5 | 39.5 | -1.8 | 1.64 |
| 4_2md114 | 112 | 112.3 | 0.3 | 41.9 | 2.5 | 0.54 |
| 4_2md115 | 112.3 | 114 | 1.7 | 35.7 | -6.2 | 1.28 |
| 4_2md116 | 114 | 114.7 | 0.7 | 37.5 | 1.7 | 0.96 |
| 4_2md117 | 114.7 | 115.2 | 0.5 | 36.2 | -1.2 | 0.67 |

| | | | | | | |
|---|---|---|---|---|---|---|
| 4_2md118 | 115.2 | 115.8 | 0.6 | 38.6 | 2.3 | 1.86 |
| 4_2md119 | 115.8 | 116.1 | 0.3 | 35.6 | -2.9 | 0.51 |
| 4_2md120 | 116.1 | 116.5 | 0.4 | 38.8 | 3.1 | 0.54 |
| 4_2md121 | 116.5 | 117.3 | 0.8 | 42.7 | 3.9 | 1.61 |
| 4_2md122 | 117.3 | 119.6 | 2.3 | 39.1 | -3.6 | 1.53 |
| 4_2md123 | 119.6 | 120.2 | 0.6 | 43.1 | 4.0 | 0.92 |
| 4_2md124 | 120.2 | 120.6 | 0.4 | 38.0 | -5.1 | 1.46 |
| 4_2md125 | 120.6 | 121.2 | 0.6 | 44.1 | 6.1 | 2.36 |
| 4_2md126 | 121.2 | 121.5 | 0.3 | 38.6 | -5.5 | 0.19 |
| 4_2md127 | 121.5 | 121.8 | 0.3 | 42.3 | 3.7 | 2.17 |
| 4_2md128 | 121.8 | 123 | 1.2 | 39.7 | -2.6 | 1.49 |
| 4_2md129 | 123 | 123.3 | 0.3 | 41.5 | 1.7 | 0.51 |
| 4_2md130 | 123.3 | 123.5 | 0.2 | 38.9 | -2.5 | 0 |
| 4_2md131 | 123.5 | 123.7 | 0.2 | 36.6 | -2.3 | 0 |
| 4_2md132 | 123.7 | 124 | 0.3 | 37.6 | 1.0 | 0.36 |
| 4_2md133 | 124 | 126.1 | 2.1 | 35.6 | -1.9 | 0.82 |
| 4_2md134 | 126.1 | 126.6 | 0.5 | 37.6 | 1.9 | 0.42 |
| 4_2md135 | 126.6 | 127.2 | 0.6 | 35.9 | -1.7 | 0.56 |
| 4_2md136 | 127.2 | 127.4 | 0.2 | 38.3 | 2.4 | 0 |
| 4_2md137 | 127.4 | 127.8 | 0.4 | 36.2 | -2.1 | 0.37 |
| 4_2md138 | 127.8 | 128.2 | 0.4 | 39.2 | 3.0 | 2.21 |
| 4_2md139 | 128.2 | 129.3 | 1.1 | 43.0 | 3.8 | 1.51 |
| 4_2md140 | 129.3 | 129.5 | 0.2 | 39.9 | -3.0 | 0 |
| 4_2md141 | 129.5 | 130.9 | 1.4 | 43.0 | 3.1 | 2.7 |
| 4_2md142 | 130.9 | 131.4 | 0.5 | 38.6 | -4.4 | 1.6 |
| 4_2md143 | 131.4 | 131.9 | 0.5 | 42.3 | 3.7 | 1.74 |
| 4_2md144 | 131.9 | 133.1 | 1.2 | 39.6 | -2.8 | 1.45 |
| 4_2md145 | 133.1 | 136.5 | 3.4 | 42.3 | 2.7 | 1.72 |
| 4_2md146 | 136.5 | 136.7 | 0.2 | 39.9 | -2.4 | 0 |
| 4_2md147 | 136.7 | 140.3 | 3.6 | 43.5 | 3.6 | 2.05 |
| 4_2md148 | 140.3 | 140.5 | 0.2 | 49.1 | 5.6 | 0 |
| 4_2md149 | 140.5 | 141.1 | 0.6 | 39.7 | -9.4 | 0.59 |
| 4_2md150 | 141.1 | 141.3 | 0.2 | 41.3 | 1.7 | 0 |
| 4_2md151 | 141.3 | 141.5 | 0.2 | 39.0 | -2.3 | 0 |
| 4_2md152 | 141.5 | 141.9 | 0.4 | 41.2 | 2.2 | 2.29 |
| 4_2md153 | 141.9 | 142.1 | 0.2 | 37.9 | -3.3 | 0 |
| 4_2md154 | 142.1 | 142.4 | 0.3 | 43.6 | 5.7 | 1.65 |
| 4_2md155 | 142.4 | 143.8 | 1.4 | 40.5 | -3.0 | 1.53 |
| 4_2md156 | 143.8 | 144.4 | 0.6 | 42.8 | 2.3 | 1.83 |
| 4_2md157 | 144.4 | 145 | 0.6 | 49.0 | 6.2 | 4.44 |
| 4_2md158 | 145 | 145.5 | 0.5 | 38.7 | -10.4 | 1.97 |
| 4_2md159 | 145.5 | 146.9 | 1.4 | 43.2 | 4.6 | 1.55 |
| 4_2md160 | 146.9 | 147.4 | 0.5 | 40.6 | -2.6 | 0.71 |
| 4_2md161 | 147.4 | 151 | 3.6 | 43.2 | 2.6 | 1.78 |
| 4_2md162 | 151 | 151.2 | 0.2 | 40.6 | -2.6 | 0 |
| 4_2md163 | 151.2 | 152 | 0.8 | 43.6 | 3.0 | 1.73 |
| 4_2md164 | 152 | 152.3 | 0.3 | 49.8 | 6.2 | 0.89 |
| 4_2md165 | 152.3 | 153.4 | 1.1 | 42.7 | -7.1 | 1.45 |
| 4_2md166 | 153.4 | 155.6 | 2.2 | 39.2 | -3.5 | 2.36 |
| 4_2md167 | 155.6 | 155.8 | 0.2 | 41.7 | 2.5 | 0 |
| 4_2md168 | 155.8 | 156 | 0.2 | 52.7 | 11.0 | 0 |
| 4_2md169 | 156 | 156.3 | 0.3 | 42.3 | -10.4 | 2.26 |
| 4_2md170 | 156.3 | 157.4 | 1.1 | 48.3 | 6.0 | 2.69 |
| 4_2md171 | 157.4 | 157.6 | 0.2 | 43.6 | -4.7 | 0 |
| 4_2md172 | 157.6 | 158.2 | 0.6 | 49.1 | 5.6 | 4.01 |
| 4_2md173 | 158.2 | 158.4 | 0.2 | 53.5 | 4.3 | 0 |

| | | | | | | |
|---|---|---|---|---|---|---|
| 4_2md174 | 158.4 | 158.7 | 0.3 | 51.3 | -2.2 | 1.55 |
| 4_2md175 | 158.7 | 159 | 0.3 | 42.2 | -9.1 | 1.81 |
| 4_2md176 | 159 | 160.3 | 1.3 | 46.5 | 4.3 | 3.57 |
| 4_2md177 | 160.3 | 160.7 | 0.4 | 39.8 | -6.6 | 0.66 |
| 4_2md178 | 160.7 | 161.6 | 0.9 | 42.3 | 2.4 | 2 |
| 4_2md179 | 161.6 | 161.8 | 0.2 | 39.4 | -2.9 | 0 |
| 4_2md180 | 161.8 | 164 | 2.2 | 43.4 | 4.0 | 2.51 |
| 4_2md181 | 164 | 165.6 | 1.6 | 37.9 | -5.5 | 1.17 |
| 4_2md182 | 165.6 | 166.5 | 0.9 | 36.4 | -1.5 | 0.68 |
| 4_2md183 | 166.5 | 167.7 | 1.2 | 38.9 | 2.5 | 1.1 |
| 4_2md184 | 167.7 | 168.7 | 1 | 44.0 | 5.1 | 1.94 |
| 4_2md185 | 168.7 | 169 | 0.3 | 39.4 | -4.6 | 1.6 |
| 4_2md186 | 169 | 169.4 | 0.4 | 41.0 | 1.6 | 0.31 |
| 4_2md187 | 169.4 | 169.7 | 0.3 | 40.3 | -0.7 | 0.75 |
| 4_2md188 | 169.7 | 170.1 | 0.4 | 42.1 | 1.8 | 0.53 |
| 4_2md189 | 170.1 | 170.8 | 0.7 | 40.6 | -1.5 | 1.21 |
| 4_2md190 | 170.8 | 171.3 | 0.5 | 50.9 | 10.3 | 3.28 |
| 4_2md191 | 171.3 | 171.7 | 0.4 | 42.3 | -8.6 | 1.16 |
| 4_2md192 | 171.7 | 173.8 | 2.1 | 38.0 | -4.4 | 1.53 |
| 4_2md193 | 173.8 | 174.3 | 0.5 | 41.0 | 3.0 | 0.97 |
| 4_2md194 | 174.3 | 174.6 | 0.3 | 39.2 | -1.8 | 0.63 |
| 4_2md195 | 174.6 | 174.8 | 0.2 | 50.7 | 11.5 | 0 |
| 4_2md196 | 174.8 | 175.4 | 0.6 | 42.6 | -8.1 | 1.69 |
| 4_2md197 | 175.4 | 176.2 | 0.8 | 40.3 | -2.3 | 0.79 |
| 4_2md198 | 176.2 | 177 | 0.8 | 42.7 | 2.4 | 1.05 |
| 4_2md199 | 177 | 177.6 | 0.6 | 39.4 | -3.3 | 0.98 |
| 4_2md200 | 177.6 | 177.8 | 0.2 | 42.7 | 3.3 | 0 |
| 4_2md201 | 177.8 | 178 | 0.2 | 47.0 | 4.2 | 0 |
| 4_2md202 | 178 | 179.6 | 1.6 | 43.5 | -3.5 | 1.98 |
| 4_2md203 | 179.6 | 179.8 | 0.2 | 40.6 | -2.9 | 0 |
| 4_2md204 | 179.8 | 180.3 | 0.5 | 43.4 | 2.8 | 2.65 |
| 4_2md205 | 180.3 | 180.7 | 0.4 | 39.9 | -3.6 | 1.59 |
| 4_2md206 | 180.7 | 181.2 | 0.5 | 35.8 | -4.0 | 0.68 |
| 4_2md207 | 181.2 | 181.4 | 0.2 | 38.3 | 2.5 | 0 |
| 4_2md208 | 181.4 | 182.9 | 1.5 | 43.0 | 4.6 | 2.53 |
| 4_2md209 | 182.9 | 183.3 | 0.4 | 39.3 | -3.7 | 0.57 |
| 4_2md210 | 183.3 | 183.5 | 0.2 | 41.4 | 2.2 | 0 |
| 4_2md211 | 183.5 | 183.7 | 0.2 | 38.5 | -2.9 | 0 |
| 4_2md212 | 183.7 | 183.9 | 0.2 | 36.7 | -1.8 | 0 |
| 4_2md213 | 183.9 | 185.9 | 2 | 39.3 | 2.7 | 1.03 |
| 4_2md214 | 185.9 | 186.5 | 0.6 | 41.6 | 2.3 | 1.23 |
| 4_2md215 | 186.5 | 187 | 0.5 | 53.7 | 12.1 | 3.18 |
| 4_2md216 | 187 | 188 | 1 | 41.0 | -12.6 | 0.66 |
| 4_2md217 | 188 | 188.2 | 0.2 | 41.5 | 0.5 | 0 |
| 4_2md218 | 188.2 | 188.5 | 0.3 | 39.0 | -2.5 | 1.22 |
| 4_2md219 | 188.5 | 188.7 | 0.2 | 42.7 | 3.6 | 0 |
| 4_2md220 | 188.7 | 189 | 0.3 | 38.9 | -3.8 | 0.92 |
| 4_2md221 | 189 | 189.4 | 0.4 | 36.3 | -2.6 | 0.56 |
| 4_2md222 | 189.4 | 189.6 | 0.2 | 37.8 | 1.5 | 0 |
| 4_2md223 | 189.6 | 190 | 0.4 | 36.5 | -1.3 | 0.29 |
| 4_2md224 | 190 | 191.3 | 1.3 | 40.0 | 3.5 | 0.57 |
| 4_2md225 | 191.3 | 191.6 | 0.3 | 43.9 | 3.9 | 0.19 |
| 4_2md226 | 191.6 | 192.8 | 1.2 | 39.2 | -4.7 | 2.55 |
| 4_2md227 | 192.8 | 193 | 0.2 | 43.3 | 4.1 | 0 |
| 4_2md228 | 193 | 193.3 | 0.3 | 40.8 | -2.6 | 0.14 |
| 4_2md229 | 193.3 | 193.5 | 0.2 | 42.1 | 1.4 | 0 |

| | | | | | | |
|---|---|---|---|---|---|---|
| 4_2md230 | 193.5 | 193.8 | 0.3 | 40.6 | -1.5 | 0.41 |
| 4_2md231 | 193.8 | 194.2 | 0.4 | 41.8 | 1.1 | 0.14 |
| 4_2md232 | 194.2 | 194.6 | 0.4 | 40.5 | -1.2 | 0.77 |
| 4_2md233 | 194.6 | 195.7 | 1.1 | 42.0 | 1.5 | 1.99 |
| 4_2md234 | 195.7 | 197.2 | 1.5 | 40.0 | -2.0 | 1.12 |
| 4_2md235 | 197.2 | 197.8 | 0.6 | 43.5 | 3.4 | 1.62 |
| 4_2md236 | 197.8 | 198.2 | 0.4 | 39.3 | -4.2 | 1.92 |
| 4_2md237 | 198.2 | 198.5 | 0.3 | 42.0 | 2.7 | 2.26 |
| 4_2md238 | 198.5 | 199 | 0.5 | 48.9 | 6.9 | 1.19 |
| 4_2md239 | 199 | 199.3 | 0.3 | 39.8 | -9.1 | 1 |
| 4_2md240 | 199.3 | 199.5 | 0.2 | 45.9 | 6.2 | 0 |
| 4_2md241 | 199.5 | 199.9 | 0.4 | 39.4 | -6.6 | 0.58 |
| 4_2md242 | 199.9 | 200.2 | 0.3 | 45.0 | 5.6 | 1.19 |
| 4_2md243 | 200.2 | 201.1 | 0.9 | 39.9 | -5.1 | 1.28 |
| 4_2md244 | 201.1 | 201.9 | 0.8 | 44.4 | 4.4 | 2.07 |
| 4_2md245 | 201.9 | 203 | 1.1 | 38.8 | -5.6 | 1.67 |
| 4_2md246 | 203 | 203.2 | 0.2 | 42.4 | 3.6 | 0 |
| 4_2md247 | 203.2 | 204 | 0.8 | 38.8 | -3.7 | 1.25 |
| 4_2md248 | 204 | 204.8 | 0.8 | 42.5 | 3.7 | 2.01 |
| 4_2md249 | 204.8 | 205 | 0.2 | 39.4 | -3.1 | 0 |
| 4_2md250 | 205 | 205.4 | 0.4 | 42.6 | 3.2 | 1.33 |
| 4_2md251 | 205.4 | 206.8 | 1.4 | 40.2 | -2.3 | 0.92 |
| 4_2md252 | 206.8 | 207.8 | 1 | 43.7 | 3.4 | 1.73 |
| 4_2md253 | 207.8 | 208.4 | 0.6 | 36.2 | -7.5 | 1.92 |
| 4_2md254 | 208.4 | 208.8 | 0.4 | 41.3 | 5.1 | 0.45 |
| 4_2md255 | 208.8 | 209 | 0.2 | 40.6 | -0.7 | 0 |
| | | | | | | |
| 5_1md1 | 0 | 0.2 | 0.2 | 35.7 | | 0 |
| 5_1md2 | 0.2 | 0.8 | 0.6 | 38.4 | 2.7 | 1.24 |
| 5_1md3 | 0.8 | 1 | 0.2 | 43.1 | 4.7 | 0 |
| 5_1md4 | 1 | 1.2 | 0.2 | 46.8 | 3.7 | 0 |
| 5_1md5 | 1.2 | 2.4 | 1.2 | 42.7 | -4.1 | 1.98 |
| 5_1md6 | 2.4 | 3.9 | 1.5 | 35.7 | -6.9 | 0.81 |
| 5_1md7 | 3.9 | 4.2 | 0.3 | 38.3 | 2.5 | 1.12 |
| 5_1md8 | 4.2 | 5 | 0.8 | 36.2 | -2.1 | 0.78 |
| 5_1md9 | 5 | 5.3 | 0.3 | 37.6 | 1.3 | 0.19 |
| 5_1md10 | 5.3 | 6.2 | 0.9 | 36.1 | -1.5 | 0.4 |
| 5_1md11 | 6.2 | 6.4 | 0.2 | 38.2 | 2.1 | 0 |
| 5_1md12 | 6.4 | 7.2 | 0.8 | 43.4 | 5.2 | 2.62 |
| 5_1md13 | 7.2 | 7.7 | 0.5 | 38.8 | -4.6 | 0.87 |
| 5_1md14 | 7.7 | 8.1 | 0.4 | 41.7 | 3.0 | 0.62 |
| 5_1md15 | 8.1 | 8.9 | 0.8 | 37.6 | -4.1 | 1.15 |
| 5_1md16 | 8.9 | 9.9 | 1 | 35.8 | -1.8 | 0.55 |
| 5_1md17 | 9.9 | 10.2 | 0.3 | 38.2 | 2.4 | 1.13 |
| 5_1md18 | 10.2 | 11.3 | 1.1 | 42.1 | 4.0 | 1.22 |
| 5_1md19 | 11.3 | 13.4 | 2.1 | 37.9 | -4.2 | 0.94 |
| 5_1md20 | 13.4 | 13.6 | 0.2 | 42.2 | 4.3 | 0 |
| 5_1md21 | 13.6 | 14.1 | 0.5 | 46.2 | 4.0 | 3 |
| 5_1md22 | 14.1 | 14.5 | 0.4 | 42.7 | -3.5 | 2.34 |
| 5_1md23 | 14.5 | 14.8 | 0.3 | 40.0 | -2.7 | 1.59 |
| 5_1md24 | 14.8 | 15.4 | 0.6 | 44.6 | 4.7 | 1.44 |
| 5_1md25 | 15.4 | 15.6 | 0.2 | 48.2 | 3.5 | 0 |
| 5_1md26 | 15.6 | 16.1 | 0.5 | 43.6 | -4.6 | 1.46 |
| 5_1md27 | 16.1 | 17.3 | 1.2 | 38.9 | -4.7 | 1.4 |
| 5_1md28 | 17.3 | 17.7 | 0.4 | 36.5 | -2.5 | 0.56 |
| 5_1md29 | 17.7 | 17.9 | 0.2 | 38.5 | 2.1 | 0 |

| | | | | | | |
|---|---|---|---|---|---|---|
| 5_1md30 | 17.9 | 18.4 | 0.5 | 44.6 | 6.1 | 1.21 |
| 5_1md31 | 18.4 | 19.5 | 1.1 | 38.5 | -6.2 | 1.03 |
| 5_1md32 | 19.5 | 20.5 | 1 | 36.2 | -2.2 | 0.58 |
| 5_1md33 | 20.5 | 20.7 | 0.2 | 38.8 | 2.6 | 0 |
| 5_1md34 | 20.7 | 20.9 | 0.2 | 36.0 | -2.8 | 0 |
| 5_1md35 | 20.9 | 21.3 | 0.4 | 37.2 | 1.3 | 0.25 |
| 5_1md36 | 21.3 | 21.8 | 0.5 | 36.4 | -0.9 | 0.37 |
| 5_1md37 | 21.8 | 22 | 0.2 | 37.8 | 1.4 | 0 |
| 5_1md38 | 22 | 22.2 | 0.2 | 36.4 | -1.4 | 0 |
| 5_1md39 | 22.2 | 23.2 | 1 | 38.1 | 1.7 | 1.08 |
| 5_1md40 | 23.2 | 23.4 | 0.2 | 36.2 | -1.9 | 0 |
| 5_1md41 | 23.4 | 24 | 0.6 | 37.5 | 1.3 | 0.76 |
| 5_1md42 | 24 | 28.9 | 4.9 | 35.5 | -2.0 | 0.85 |
| 5_1md43 | 28.9 | 29.1 | 0.2 | 38.3 | 2.8 | 0 |
| 5_1md44 | 29.1 | 31.7 | 2.6 | 34.8 | -3.6 | 1.17 |
| 5_1md45 | 31.7 | 34.3 | 2.6 | 37.9 | 3.2 | 0.73 |
| 5_1md46 | 34.3 | 34.5 | 0.2 | 36.5 | -1.4 | 0 |
| 5_1md47 | 34.5 | 35.3 | 0.8 | 38.2 | 1.6 | 0.67 |
| 5_1md48 | 35.3 | 38.2 | 2.9 | 0.0 | -38.2 | 0 |
| 5_1md49 | 38.2 | 40.9 | 2.7 | 38.6 | 38.6 | 0.92 |
| 5_1md50 | 40.9 | 42.4 | 1.5 | 36.5 | -2.1 | 0.69 |
| 5_1md51 | 42.4 | 42.9 | 0.5 | 37.9 | 1.4 | 1.13 |
| 5_1md52 | 42.9 | 43.2 | 0.3 | 36.3 | -1.5 | 1.3 |
| 5_1md53 | 43.2 | 43.6 | 0.4 | 37.4 | 1.1 | 0.24 |
| 5_1md54 | 43.6 | 46.2 | 2.6 | 35.7 | -1.8 | 0.7 |
| 5_1md55 | 46.2 | 46.9 | 0.7 | 37.6 | 1.9 | 0.82 |
| 5_1md56 | 46.9 | 47.2 | 0.3 | 36.5 | -1.1 | 0.37 |
| 5_1md57 | 47.2 | 47.9 | 0.7 | 38.2 | 1.8 | 0.49 |
| 5_1md58 | 47.9 | 50.5 | 2.6 | 35.7 | -2.5 | 0.7 |
| 5_1md59 | 50.5 | 51.6 | 1.1 | 38.1 | 2.4 | 1 |
| 5_1md60 | 51.6 | 52 | 0.4 | 36.8 | -1.3 | 0.35 |
| 5_1md61 | 52 | 53.6 | 1.6 | 37.8 | 1.0 | 0.96 |
| 5_1md62 | 53.6 | 54.3 | 0.7 | 36.5 | -1.3 | 0.37 |
| 5_1md63 | 54.3 | 54.5 | 0.2 | 37.1 | 0.7 | 0 |
| 5_1md64 | 54.5 | 61.4 | 6.9 | 36.2 | -0.9 | 0.93 |
| 5_1md65 | 61.4 | 63.6 | 2.2 | 38.1 | 1.9 | 1.08 |
| 5_1md66 | 63.6 | 65.3 | 1.7 | 35.3 | -2.8 | 0.74 |
| 5_1md67 | 65.3 | 66.4 | 1.1 | 37.8 | 2.5 | 1.07 |
| 5_1md68 | 66.4 | 77.9 | 11.5 | 34.9 | -2.9 | 0.95 |
| 5_1md69 | 77.9 | 78.3 | 0.4 | 37.8 | 2.9 | 0.53 |
| 5_1md70 | 78.3 | 78.6 | 0.3 | 36.6 | -1.2 | 0.48 |
| 5_1md71 | 78.6 | 83.2 | 4.6 | 38.0 | 1.4 | 1 |
| 5_1md72 | 83.2 | 95.9 | 12.7 | 35.1 | -2.9 | 1.12 |
| 5_1md73 | 95.9 | 96.9 | 1 | 39.0 | 3.8 | 1.09 |
| 5_1md74 | 96.9 | 100.5 | 3.6 | 35.2 | -3.8 | 1.01 |
| 5_1md75 | 100.5 | 100.8 | 0.3 | 37.3 | 2.1 | 0.17 |
| 5_1md76 | 100.8 | 101 | 0.2 | 36.3 | -1.0 | 0 |
| 5_1md77 | 101 | 101.7 | 0.7 | 38.7 | 2.4 | 1.17 |
| 5_1md78 | 101.7 | 102 | 0.3 | 35.8 | -2.9 | 0.95 |
| 5_1md79 | 102 | 102.9 | 0.9 | 38.6 | 2.8 | 1.31 |
| 5_1md80 | 102.9 | 107.5 | 4.6 | 35.1 | -3.5 | 1.05 |
| 5_1md81 | 107.5 | 108.1 | 0.6 | 38.2 | 3.0 | 0.42 |
| 5_1md82 | 108.1 | 111 | 2.9 | 36.2 | -1.9 | 0.78 |
| 5_1md83 | 111 | 111.2 | 0.2 | 37.3 | 1.1 | 0 |
| 5_1md84 | 111.2 | 115.9 | 4.7 | 35.0 | -2.3 | 1.13 |
| 5_1md85 | 115.9 | 116.7 | 0.8 | 38.0 | 3.1 | 0.95 |

| | | | | | | |
|---|---|---|---|---|---|---|
| 5_1md86 | 116.7 | 120.4 | 3.7 | 34.9 | -3.1 | 1.06 |
| 5_1md87 | 120.4 | 120.7 | 0.3 | 37.8 | 2.9 | 0.17 |
| 5_1md88 | 120.7 | 122.2 | 1.5 | 36.3 | -1.5 | 0.77 |
| 5_1md89 | 122.2 | 122.9 | 0.7 | 37.8 | 1.5 | 0.72 |
| 5_1md90 | 122.9 | 123.1 | 0.2 | 36.3 | -1.5 | 0 |
| 5_1md91 | 123.1 | 124.4 | 1.3 | 38.8 | 2.6 | 0.92 |
| 5_1md92 | 124.4 | 124.6 | 0.2 | 36.2 | -2.6 | 0 |
| 5_1md93 | 124.6 | 124.9 | 0.3 | 39.6 | 3.4 | 0.41 |
| 5_1md94 | 124.9 | 125.2 | 0.3 | 34.5 | -5.1 | 0.48 |
| 5_1md95 | 125.2 | 127 | 1.8 | 38.8 | 4.3 | 0.87 |
| 5_1md96 | 127 | 127.8 | 0.8 | 34.9 | -4.0 | 1.27 |
| 5_1md97 | 127.8 | 128.2 | 0.4 | 38.0 | 3.2 | 1.32 |
| 5_1md98 | 128.2 | 129.5 | 1.3 | 36.1 | -2.0 | 0.7 |
| 5_1md99 | 129.5 | 129.9 | 0.4 | 36.9 | 0.9 | 0.81 |
| 5_1md100 | 129.9 | 130.3 | 0.4 | 34.8 | -2.2 | 1.46 |
| 5_1md101 | 130.3 | 131.2 | 0.9 | 37.7 | 3.0 | 0.93 |
| 5_1md102 | 131.2 | 132.2 | 1 | 36.1 | -1.6 | 0.43 |
| 5_1md103 | 132.2 | 133.9 | 1.7 | 38.7 | 2.5 | 1.39 |
| 5_1md104 | 133.9 | 134.3 | 0.4 | 36.1 | -2.5 | 0.77 |
| 5_1md105 | 134.3 | 134.7 | 0.4 | 37.6 | 1.5 | 0.58 |
| 5_1md106 | 134.7 | 135.2 | 0.5 | 36.8 | -0.8 | 0.53 |
| 5_1md107 | 135.2 | 136.6 | 1.4 | 37.6 | 0.8 | 0.79 |
| 5_1md108 | 136.6 | 137.9 | 1.3 | 36.5 | -1.1 | 0.68 |
| 5_1md109 | 137.9 | 141.5 | 3.6 | 38.8 | 2.3 | 1.26 |
| 5_1md110 | 141.5 | 141.7 | 0.2 | 36.4 | -2.3 | 0 |
| 5_1md111 | 141.7 | 141.9 | 0.2 | 37.6 | 1.2 | 0 |
| 5_1md112 | 141.9 | 154.1 | 12.2 | 34.6 | -3.0 | 0.88 |
| 5_1md113 | 154.1 | 154.6 | 0.5 | 39.9 | 5.3 | 2.67 |
| 5_1md114 | 154.6 | 157.4 | 2.8 | 35.4 | -4.6 | 0.98 |
| | | | | | | |
| 5_2md1 | 0 | 11 | 11 | 34.3 | | 0.99 |
| 5_2md2 | 11 | 12.3 | 1.3 | 38.1 | 3.8 | 1.17 |
| 5_2md3 | 12.3 | 13 | 0.7 | 35.7 | -2.3 | 0.79 |
| 5_2md4 | 13 | 15.1 | 2.1 | 38.6 | 2.8 | 0.91 |
| 5_2md5 | 15.1 | 15.6 | 0.5 | 35.8 | -2.7 | 1.05 |
| 5_2md6 | 15.6 | 16.5 | 0.9 | 38.1 | 2.2 | 0.63 |
| 5_2md7 | 16.5 | 17.3 | 0.8 | 35.8 | -2.2 | 1.17 |
| 5_2md8 | 17.3 | 17.7 | 0.4 | 37.1 | 1.2 | 1.15 |
| 5_2md9 | 17.7 | 24.4 | 6.7 | 35.1 | -2.0 | 0.9 |
| 5_2md10 | 24.4 | 25.7 | 1.3 | 37.4 | 2.3 | 1.1 |
| 5_2md11 | 25.7 | 25.9 | 0.2 | 36.6 | -0.8 | 0 |
| 5_2md12 | 25.9 | 27.1 | 1.2 | 38.0 | 1.5 | 1.16 |
| 5_2md13 | 27.1 | 28.4 | 1.3 | 35.8 | -2.2 | 0.74 |
| 5_2md14 | 28.4 | 29.9 | 1.5 | 38.5 | 2.7 | 0.9 |
| 5_2md15 | 29.9 | 43 | 13.1 | 34.5 | -4.0 | 0.94 |
| 5_2md16 | 43 | 43.2 | 0.2 | 37.5 | 3.0 | 0 |
| 5_2md17 | 43.2 | 44 | 0.8 | 35.8 | -1.7 | 1.11 |
| 5_2md18 | 44 | 45.7 | 1.7 | 37.7 | 1.9 | 0.83 |
| 5_2md19 | 45.7 | 45.9 | 0.2 | 36.3 | -1.4 | 0 |
| 5_2md20 | 45.9 | 46.4 | 0.5 | 38.1 | 1.8 | 0.37 |
| 5_2md21 | 46.4 | 46.6 | 0.2 | 36.2 | -1.9 | 0 |
| 5_2md22 | 46.6 | 46.9 | 0.3 | 37.7 | 1.5 | 0.42 |
| 5_2md23 | 46.9 | 47.5 | 0.6 | 35.3 | -2.4 | 1.05 |
| 5_2md24 | 47.5 | 47.7 | 0.2 | 37.4 | 2.1 | 0 |
| 5_2md25 | 47.7 | 54 | 6.3 | 35.3 | -2.1 | 1.04 |
| 5_2md26 | 54 | 54.3 | 0.3 | 37.2 | 1.9 | 0.78 |

| | | | | | | |
|---|---|---|---|---|---|---|
| 5_2md27 | 54.3 | 54.8 | 0.5 | 37.8 | 0.6 | 1.35 |
| 5_2md28 | 54.8 | 55.3 | 0.5 | 36.3 | -1.5 | 0.54 |
| 5_2md29 | 55.3 | 55.5 | 0.2 | 37.5 | 1.2 | 0 |
| 5_2md30 | 55.5 | 55.9 | 0.4 | 35.7 | -1.8 | 0.75 |
| 5_2md31 | 55.9 | 56.5 | 0.6 | 37.7 | 2.0 | 0.69 |
| 5_2md32 | 56.5 | 57.2 | 0.7 | 36.3 | -1.4 | 1.04 |
| 5_2md33 | 57.2 | 57.5 | 0.3 | 37.3 | 1.0 | 0.19 |
| 5_2md34 | 57.5 | 58.3 | 0.8 | 36.2 | -1.1 | 0.45 |
| 5_2md35 | 58.3 | 58.9 | 0.6 | 37.7 | 1.4 | 0.63 |
| 5_2md36 | 58.9 | 62 | 3.1 | 35.5 | -2.2 | 0.75 |
| 5_2md37 | 62 | 62.4 | 0.4 | 37.0 | 1.5 | 0.74 |
| 5_2md38 | 62.4 | 62.6 | 0.2 | 35.9 | -1.1 | 0 |
| 5_2md39 | 62.6 | 65 | 2.4 | 38.1 | 2.1 | 1.35 |
| 5_2md40 | 65 | 66 | 1 | 36.3 | -1.8 | 0.69 |
| 5_2md41 | 66 | 66.9 | 0.9 | 38.7 | 2.4 | 1.25 |
| 5_2md42 | 66.9 | 67.2 | 0.3 | 35.8 | -2.9 | 1.1 |
| 5_2md43 | 67.2 | 68 | 0.8 | 38.3 | 2.4 | 0.37 |
| 5_2md44 | 68 | 68.7 | 0.7 | 36.5 | -1.8 | 0.37 |
| 5_2md45 | 68.7 | 71.8 | 3.1 | 38.0 | 1.6 | 0.91 |
| 5_2md46 | 71.8 | 72 | 0.2 | 36.2 | -1.8 | 0 |
| 5_2md47 | 72 | 73.1 | 1.1 | 38.1 | 1.9 | 1.65 |
| 5_2md48 | 73.1 | 73.3 | 0.2 | 36.3 | -1.8 | 0 |
| 5_2md49 | 73.3 | 74.5 | 1.2 | 38.3 | 2.0 | 1.97 |
| 5_2md50 | 74.5 | 75.2 | 0.7 | 38.0 | -0.3 | 4.24 |
| 5_2md51 | 75.2 | 75.9 | 0.7 | 39.2 | 1.2 | 2.57 |
| 5_2md52 | 75.9 | 76.6 | 0.7 | 43.1 | 3.8 | 1.27 |
| 5_2md53 | 76.6 | 78 | 1.4 | 39.8 | -3.3 | 1.65 |
| 5_2md54 | 78 | 78.5 | 0.5 | 36.5 | -3.3 | 0.39 |
| 5_2md55 | 78.5 | 79.3 | 0.8 | 39.1 | 2.7 | 1.6 |
| 5_2md56 | 79.3 | 79.6 | 0.3 | 44.3 | 5.1 | 1.99 |
| 5_2md57 | 79.6 | 79.9 | 0.3 | 37.7 | -6.6 | 2.3 |
| 5_2md58 | 79.9 | 80.8 | 0.9 | 43.8 | 6.1 | 3.52 |
| 5_2md59 | 80.8 | 81.1 | 0.3 | 38.9 | -4.9 | 1.94 |
| 5_2md60 | 81.1 | 81.5 | 0.4 | 43.5 | 4.6 | 1.88 |
| 5_2md61 | 81.5 | 81.8 | 0.3 | 38.5 | -5.0 | 1.35 |
| 5_2md62 | 81.8 | 82 | 0.2 | 41.4 | 2.9 | 0 |
| 5_2md63 | 82 | 82.7 | 0.7 | 39.0 | -2.3 | 1.32 |
| 5_2md64 | 82.7 | 82.9 | 0.2 | 42.7 | 3.7 | 0 |
| 5_2md65 | 82.9 | 83.3 | 0.4 | 37.7 | -5.0 | 0.93 |
| 5_2md66 | 83.3 | 84 | 0.7 | 36.5 | -1.2 | 0.67 |
| 5_2md67 | 84 | 84.4 | 0.4 | 37.9 | 1.4 | 0.34 |
| 5_2md68 | 84.4 | 84.7 | 0.3 | 35.8 | -2.1 | 0.47 |
| 5_2md69 | 84.7 | 86.2 | 1.5 | 38.2 | 2.4 | 1.55 |
| 5_2md70 | 86.2 | 86.8 | 0.6 | 41.8 | 3.6 | 0.96 |
| 5_2md71 | 86.8 | 87.1 | 0.3 | 37.2 | -4.6 | 0.84 |
| 5_2md72 | 87.1 | 87.4 | 0.3 | 43.2 | 6.0 | 0.8 |
| 5_2md73 | 87.4 | 90.1 | 2.7 | 38.8 | -4.4 | 1.79 |
| 5_2md74 | 90.1 | 90.4 | 0.3 | 42.4 | 3.6 | 0.38 |
| 5_2md75 | 90.4 | 90.7 | 0.3 | 35.9 | -6.5 | 1.24 |
| 5_2md76 | 90.7 | 92 | 1.3 | 39.9 | 4.0 | 2.06 |
| 5_2md77 | 92 | 92.3 | 0.3 | 42.7 | 2.7 | 1.16 |
| 5_2md78 | 92.3 | 94.6 | 2.3 | 38.9 | -3.8 | 1.87 |
| 5_2md79 | 94.6 | 95 | 0.4 | 41.8 | 2.9 | 0.75 |
| 5_2md80 | 95 | 96.9 | 1.9 | 38.8 | -3.0 | 1.27 |
| 5_2md81 | 96.9 | 97.2 | 0.3 | 36.1 | -2.7 | 0.99 |
| 5_2md82 | 97.2 | 98.6 | 1.4 | 38.1 | 2.1 | 0.68 |

| | | | | | | |
|---|---|---|---|---|---|---|
| 5_2md83 | 98.6 | 98.9 | 0.3 | 35.9 | -2.2 | 0.59 |
| 5_2md84 | 98.9 | 99.2 | 0.3 | 38.4 | 2.5 | 0.47 |
| 5_2md85 | 99.2 | 100.6 | 1.4 | 35.5 | -2.9 | 0.74 |
| 5_2md86 | 100.6 | 101.1 | 0.5 | 37.5 | 2.0 | 0.36 |
| 5_2md87 | 101.1 | 104.4 | 3.3 | 35.5 | -2.0 | 0.82 |
| 5_2md88 | 104.4 | 105.7 | 1.3 | 38.7 | 3.2 | 0.94 |
| 5_2md89 | 105.7 | 106.1 | 0.4 | 35.0 | -3.7 | 0.91 |
| 5_2md90 | 106.1 | 106.5 | 0.4 | 38.3 | 3.3 | 0.4 |
| 5_2md91 | 106.5 | 108 | 1.5 | 36.0 | -2.3 | 0.7 |
| 5_2md92 | 108 | 109.1 | 1.1 | 38.6 | 2.6 | 0.98 |
| 5_2md93 | 109.1 | 110 | 0.9 | 35.8 | -2.9 | 0.33 |
| 5_2md94 | 110 | 111 | 1 | 40.1 | 4.3 | 1.67 |
| 5_2md95 | 111 | 111.2 | 0.2 | 36.2 | -3.9 | 0 |
| 5_2md96 | 111.2 | 112 | 0.8 | 39.3 | 3.0 | 2.31 |
| 5_2md97 | 112 | 112.2 | 0.2 | 42.6 | 3.3 | 0 |
| 5_2md98 | 112.2 | 112.5 | 0.3 | 40.2 | -2.4 | 1.4 |
| 5_2md99 | 112.5 | 113 | 0.5 | 43.6 | 3.4 | 3.43 |
| 5_2md100 | 113 | 113.7 | 0.7 | 39.9 | -3.7 | 1.77 |
| 5_2md101 | 113.7 | 113.9 | 0.2 | 41.2 | 1.4 | 0 |
| 5_2md102 | 113.9 | 115 | 1.1 | 40.2 | -1.0 | 0.62 |
| 5_2md103 | 115 | 115.3 | 0.3 | 44.8 | 4.6 | 3.75 |
| 5_2md104 | 115.3 | 115.8 | 0.5 | 38.8 | -6.0 | 1.26 |
| 5_2md105 | 115.8 | 121.9 | 6.1 | 35.6 | -3.2 | 0.94 |
| 5_2md106 | 121.9 | 122.8 | 0.9 | 39.2 | 3.6 | 1.96 |
| 5_2md107 | 122.8 | 124.4 | 1.6 | 42.7 | 3.5 | 1.64 |
| 5_2md108 | 124.4 | 124.6 | 0.2 | 40.2 | -2.5 | 0 |
| 5_2md109 | 124.6 | 125.5 | 0.9 | 42.9 | 2.7 | 2.12 |
| 5_2md110 | 125.5 | 125.7 | 0.2 | 48.9 | 6.0 | 0 |
| 5_2md111 | 125.7 | 125.9 | 0.2 | 43.0 | -5.9 | 0 |
| 5_2md112 | 125.9 | 127.2 | 1.3 | 46.3 | 3.3 | 4.45 |
| 5_2md113 | 127.2 | 127.4 | 0.2 | 40.2 | -6.1 | 0 |
| 5_2md114 | 127.4 | 127.6 | 0.2 | 43.2 | 3.0 | 0 |
| 5_2md115 | 127.6 | 127.9 | 0.3 | 37.5 | -5.8 | 1.2 |
| 5_2md116 | 127.9 | 133.5 | 5.6 | 34.6 | -2.9 | 1.19 |
| 5_2md117 | 133.5 | 133.9 | 0.4 | 37.9 | 3.3 | 0.7 |
| 5_2md118 | 133.9 | 134.6 | 0.7 | 44.1 | 6.2 | 2.37 |
| 5_2md119 | 134.6 | 135.2 | 0.6 | 39.1 | -5.0 | 1.36 |
| 5_2md120 | 135.2 | 136.1 | 0.9 | 35.8 | -3.3 | 0.62 |
| 5_2md121 | 136.1 | 138.6 | 2.5 | 39.0 | 3.1 | 2.36 |
| 5_2md122 | 138.6 | 140.4 | 1.8 | 35.9 | -3.1 | 0.89 |
| 5_2md123 | 140.4 | 141.3 | 0.9 | 38.0 | 2.1 | 1.37 |
| 5_2md124 | 141.3 | 141.6 | 0.3 | 42.7 | 4.7 | 1.42 |
| 5_2md125 | 141.6 | 142.6 | 1 | 48.1 | 5.4 | 4.04 |
| 5_2md126 | 142.6 | 142.9 | 0.3 | 43.4 | -4.7 | 2.84 |
| 5_2md127 | 142.9 | 143.6 | 0.7 | 48.7 | 5.2 | 4.88 |
| 5_2md128 | 143.6 | 143.8 | 0.2 | 40.1 | -8.5 | 0 |
| 5_2md129 | 143.8 | 144.1 | 0.3 | 45.0 | 4.8 | 1.03 |
| 5_2md130 | 144.1 | 144.6 | 0.5 | 52.6 | 7.6 | 3 |
| 5_2md131 | 144.6 | 145.1 | 0.5 | 40.9 | -11.7 | 1.7 |
| 5_2md132 | 145.1 | 145.3 | 0.2 | 38.8 | -2.1 | 0 |
| 5_2md133 | 145.3 | 147.3 | 2 | 49.2 | 10.4 | 2.06 |
| 5_2md134 | 147.3 | 147.5 | 0.2 | 45.6 | -3.5 | 0 |
| | | | | | | |
| 6_1md1 | 0 | 0.7 | 0.7 | 39.1 | | 2.07 |
| 6_1md2 | 0.7 | 1 | 0.3 | 45.5 | 6.4 | 3.96 |
| 6_1md3 | 1 | 1.4 | 0.4 | 40.8 | -4.7 | 0.72 |

| | | | | | | |
|---|---|---|---|---|---|---|
| 6_1md4 | 1.4 | 1.7 | 0.3 | 42.5 | 1.7 | 0.61 |
| 6_1md5 | 1.7 | 2.8 | 1.1 | 39.4 | -3.0 | 1.19 |
| 6_1md6 | 2.8 | 3.2 | 0.4 | 41.3 | 1.8 | 2.99 |
| 6_1md7 | 3.2 | 5 | 1.8 | 38.7 | -2.6 | 1.05 |
| 6_1md8 | 5 | 6.6 | 1.6 | 35.5 | -3.2 | 1.13 |
| 6_1md9 | 6.6 | 7.8 | 1.2 | 38.5 | 3.1 | 1.21 |
| 6_1md10 | 7.8 | 10.3 | 2.5 | 35.1 | -3.5 | 0.74 |
| 6_1md11 | 10.3 | 12 | 1.7 | 38.7 | 3.7 | 1.15 |
| 6_1md12 | 12 | 12.5 | 0.5 | 46.4 | 7.7 | 3.1 |
| 6_1md13 | 12.5 | 12.8 | 0.3 | 39.6 | -6.8 | 1.34 |
| 6_1md14 | 12.8 | 13.1 | 0.3 | 45.2 | 5.6 | 3.36 |
| 6_1md15 | 13.1 | 15.6 | 2.5 | 38.7 | -6.4 | 1.94 |
| 6_1md16 | 15.6 | 16.1 | 0.5 | 36.7 | -2.0 | 0.64 |
| 6_1md17 | 16.1 | 16.3 | 0.2 | 37.5 | 0.7 | 0 |
| 6_1md18 | 16.3 | 16.5 | 0.2 | 36.3 | -1.1 | 0 |
| 6_1md19 | 16.5 | 17.2 | 0.7 | 38.5 | 2.1 | 0.88 |
| 6_1md20 | 17.2 | 17.6 | 0.4 | 36.3 | -2.1 | 0.26 |
| 6_1md21 | 17.6 | 18.4 | 0.8 | 37.6 | 1.3 | 1.14 |
| 6_1md22 | 18.4 | 18.8 | 0.4 | 35.7 | -1.9 | 0.56 |
| 6_1md23 | 18.8 | 19.7 | 0.9 | 37.4 | 1.7 | 0.76 |
| 6_1md24 | 19.7 | 19.9 | 0.2 | 36.3 | -1.1 | 0 |
| 6_1md25 | 19.9 | 20.1 | 0.2 | 37.2 | 0.9 | 0 |
| 6_1md26 | 20.1 | 25.8 | 5.7 | 34.5 | -2.7 | 1.08 |
| 6_1md27 | 25.8 | 26.1 | 0.3 | 42.8 | 8.3 | 1.11 |
| 6_1md28 | 26.1 | 28.5 | 2.4 | 38.6 | -4.2 | 1.35 |
| 6_1md29 | 28.5 | 28.8 | 0.3 | 34.8 | -3.8 | 0.89 |
| 6_1md30 | 28.8 | 29.9 | 1.1 | 39.0 | 4.2 | 1.07 |
| 6_1md31 | 29.9 | 30.3 | 0.4 | 37.0 | -2.0 | 0.8 |
| 6_1md32 | 30.3 | 33.7 | 3.4 | 37.7 | 0.7 | 0.83 |
| 6_1md33 | 33.7 | 34.2 | 0.5 | 36.4 | -1.3 | 0.32 |
| 6_1md34 | 34.2 | 34.6 | 0.4 | 37.3 | 0.8 | 0.29 |
| 6_1md35 | 34.6 | 36 | 1.4 | 35.8 | -1.5 | 1.05 |
| 6_1md36 | 36 | 36.5 | 0.5 | 37.5 | 1.7 | 0.79 |
| 6_1md37 | 36.5 | 51.4 | 14.9 | 35.2 | -2.3 | 1.14 |
| 6_1md38 | 51.4 | 52.3 | 0.9 | 37.7 | 2.4 | 0.74 |
| 6_1md39 | 52.3 | 52.5 | 0.2 | 41.7 | 4.1 | 0 |
| 6_1md40 | 52.5 | 53.4 | 0.9 | 38.1 | -3.7 | 1.56 |
| 6_1md41 | 53.4 | 53.6 | 0.2 | 36.3 | -1.8 | 0 |
| 6_1md42 | 53.6 | 55.7 | 2.1 | 37.5 | 1.2 | 1.2 |
| 6_1md43 | 55.7 | 56.5 | 0.8 | 36.5 | -1.0 | 0.37 |
| 6_1md44 | 56.5 | 58.5 | 2 | 38.6 | 2.2 | 1.22 |
| 6_1md45 | 58.5 | 58.7 | 0.2 | 36.2 | -2.4 | 0 |
| 6_1md46 | 58.7 | 60.6 | 1.9 | 38.0 | 1.8 | 0.74 |
| 6_1md47 | 60.6 | 61.4 | 0.8 | 36.5 | -1.5 | 0.72 |
| 6_1md48 | 61.4 | 62 | 0.6 | 38.1 | 1.7 | 1.33 |
| 6_1md49 | 62 | 62.8 | 0.8 | 36.2 | -1.9 | 0.5 |
| 6_1md50 | 62.8 | 63.3 | 0.5 | 38.0 | 1.8 | 2.44 |
| 6_1md51 | 63.3 | 63.8 | 0.5 | 36.8 | -1.2 | 0.55 |
| 6_1md52 | 63.8 | 64.5 | 0.7 | 37.6 | 0.9 | 0.54 |
| 6_1md53 | 64.5 | 64.7 | 0.2 | 34.9 | -2.7 | 0 |
| 6_1md54 | 64.7 | 65 | 0.3 | 38.0 | 3.1 | 0.75 |
| 6_1md55 | 65 | 66.8 | 1.8 | 36.2 | -1.8 | 0.73 |
| 6_1md56 | 66.8 | 68.6 | 1.8 | 38.3 | 2.1 | 1.03 |
| 6_1md57 | 68.6 | 68.9 | 0.3 | 36.9 | -1.5 | 2.43 |
| 6_1md58 | 68.9 | 69.1 | 0.2 | 42.1 | 5.2 | 0 |
| 6_1md59 | 69.1 | 69.9 | 0.8 | 39.4 | -2.7 | 0.86 |

| | | | | | | |
|---|---|---|---|---|---|---|
| 6_1md60 | 69.9 | 70.1 | 0.2 | 41.7 | 2.3 | 0 |
| 6_1md61 | 70.1 | 70.5 | 0.4 | 40.5 | -1.2 | 0.48 |
| 6_1md62 | 70.5 | 70.9 | 0.4 | 41.8 | 1.4 | 0.58 |
| 6_1md63 | 70.9 | 71.1 | 0.2 | 40.4 | -1.5 | 0 |
| 6_1md64 | 71.1 | 71.5 | 0.4 | 42.5 | 2.2 | 0.87 |
| 6_1md65 | 71.5 | 72 | 0.5 | 38.8 | -3.8 | 1.42 |
| 6_1md66 | 72 | 72.4 | 0.4 | 35.9 | -2.8 | 0.2 |
| 6_1md67 | 72.4 | 72.7 | 0.3 | 37.7 | 1.7 | 0.22 |
| 6_1md68 | 72.7 | 73.1 | 0.4 | 40.6 | 2.9 | 2.39 |
| 6_1md69 | 73.1 | 73.5 | 0.4 | 40.5 | -0.1 | 0.49 |
| 6_1md70 | 73.5 | 73.7 | 0.2 | 42.6 | 2.1 | 0 |
| 6_1md71 | 73.7 | 73.9 | 0.2 | 54.0 | 11.4 | 0 |
| 6_1md72 | 73.9 | 76.8 | 2.9 | 0.0 | -54.0 | 0 |
| 6_1md73 | 76.8 | 77.1 | 0.3 | 39.4 | 39.4 | 1.43 |
| 6_1md74 | 77.1 | 77.7 | 0.6 | 41.6 | 2.2 | 0.86 |
| 6_1md75 | 77.7 | 78.7 | 1 | 38.5 | -3.0 | 1.58 |
| 6_1md76 | 78.7 | 79.7 | 1 | 35.1 | -3.4 | 0.81 |
| 6_1md77 | 79.7 | 80.3 | 0.6 | 38.0 | 2.9 | 1.15 |
| 6_1md78 | 80.3 | 81 | 0.7 | 35.7 | -2.3 | 0.55 |
| 6_1md79 | 81 | 81.9 | 0.9 | 38.3 | 2.6 | 1.53 |
| 6_1md80 | 81.9 | 82.7 | 0.8 | 42.8 | 4.6 | 1.59 |
| 6_1md81 | 82.7 | 83.5 | 0.8 | 39.3 | -3.6 | 2.1 |
| 6_1md82 | 83.5 | 84.5 | 1 | 42.0 | 2.8 | 1.08 |
| 6_1md83 | 84.5 | 84.8 | 0.3 | 39.0 | -3.1 | 1.33 |
| 6_1md84 | 84.8 | 85.1 | 0.3 | 36.8 | -2.1 | 2.68 |
| 6_1md85 | 85.1 | 85.4 | 0.3 | 41.9 | 5.0 | 0.86 |
| 6_1md86 | 85.4 | 85.8 | 0.4 | 39.9 | -2.0 | 2.03 |
| 6_1md87 | 85.8 | 86 | 0.2 | 44.5 | 4.6 | 0 |
| 6_1md88 | 86 | 87.4 | 1.4 | 38.6 | -6.0 | 0.99 |
| 6_1md89 | 87.4 | 88.6 | 1.2 | 36.6 | -2.0 | 0.66 |
| 6_1md90 | 88.6 | 89.2 | 0.6 | 37.4 | 0.8 | 0.61 |
| 6_1md91 | 89.2 | 93.9 | 4.7 | 35.6 | -1.8 | 0.89 |
| 6_1md92 | 93.9 | 94.3 | 0.4 | 38.3 | 2.8 | 0.4 |
| 6_1md93 | 94.3 | 94.5 | 0.2 | 36.2 | -2.1 | 0 |
| 6_1md94 | 94.5 | 94.7 | 0.2 | 37.5 | 1.3 | 0 |
| 6_1md95 | 94.7 | 95.9 | 1.2 | 35.2 | -2.3 | 0.74 |
| 6_1md96 | 95.9 | 96.3 | 0.4 | 39.1 | 3.9 | 1.7 |
| 6_1md97 | 96.3 | 97.4 | 1.1 | 36.8 | -2.3 | 0.46 |
| 6_1md98 | 97.4 | 97.6 | 0.2 | 39.2 | 2.4 | 0 |
| 6_1md99 | 97.6 | 97.8 | 0.2 | 35.9 | -3.3 | 0 |
| 6_1md100 | 97.8 | 98 | 0.2 | 37.8 | 1.9 | 0 |
| 6_1md101 | 98 | 98.4 | 0.4 | 36.1 | -1.7 | 0.65 |
| 6_1md102 | 98.4 | 100.1 | 1.7 | 38.5 | 2.4 | 1.06 |
| 6_1md103 | 100.1 | 101.3 | 1.2 | 36.4 | -2.1 | 0.62 |
| 6_1md104 | 101.3 | 102.6 | 1.3 | 37.6 | 1.2 | 0.81 |
| 6_1md105 | 102.6 | 103.4 | 0.8 | 36.5 | -1.2 | 0.5 |
| 6_1md106 | 103.4 | 105.3 | 1.9 | 38.0 | 1.5 | 1.05 |
| 6_1md107 | 105.3 | 106 | 0.7 | 36.0 | -2.0 | 0.62 |
| 6_1md108 | 106 | 106.4 | 0.4 | 37.5 | 1.6 | 0.71 |
| 6_1md109 | 106.4 | 107 | 0.6 | 37.2 | -0.3 | 0.5 |
| 6_1md110 | 107 | 107.2 | 0.2 | 37.2 | 0.0 | 0 |
| 6_1md111 | 107.2 | 108.6 | 1.4 | 36.4 | -0.8 | 1.02 |
| 6_1md112 | 108.6 | 108.9 | 0.3 | 39.4 | 3.0 | 1.58 |
| 6_1md113 | 108.9 | 109.1 | 0.2 | 34.9 | -4.5 | 0 |
| 6_1md114 | 109.1 | 109.7 | 0.6 | 38.9 | 4.0 | 2.22 |
| 6_1md115 | 109.7 | 110.3 | 0.6 | 41.5 | 2.7 | 0.75 |

| | | | | | | |
|---|---|---|---|---|---|---|
| 6_1md116 | 110.3 | 110.5 | 0.2 | 35.9 | -5.6 | 0 |
| 6_1md117 | 110.5 | 111.8 | 1.3 | 38.2 | 2.3 | 1.39 |
| 6_1md118 | 111.8 | 112.9 | 1.1 | 36.2 | -2.0 | 0.67 |
| 6_1md119 | 112.9 | 115 | 2.1 | 37.8 | 1.5 | 1 |
| 6_1md120 | 115 | 117.7 | 2.7 | 36.3 | -1.5 | 0.85 |
| 6_1md121 | 117.7 | 122.6 | 4.9 | 39.1 | 2.8 | 1.23 |
| 6_1md122 | 122.6 | 122.9 | 0.3 | 43.7 | 4.7 | 2.44 |
| 6_1md123 | 122.9 | 123.6 | 0.7 | 37.8 | -6.0 | 1.15 |
| 6_1md124 | 123.6 | 123.8 | 0.2 | 44.1 | 6.3 | 0 |
| 6_1md125 | 123.8 | 124 | 0.2 | 39.2 | -4.8 | 0 |
| 6_1md126 | 124 | 124.5 | 0.5 | 44.6 | 5.4 | 2.16 |
| 6_1md127 | 124.5 | 125.6 | 1.1 | 38.7 | -5.9 | 1.08 |
| 6_1md128 | 125.6 | 125.8 | 0.2 | 35.1 | -3.7 | 0 |
| 6_1md129 | 125.8 | 126.6 | 0.8 | 38.4 | 3.4 | 0.84 |
| 6_1md130 | 126.6 | 127.2 | 0.6 | 36.9 | -1.5 | 0.62 |
| 6_1md131 | 127.2 | 127.8 | 0.6 | 37.7 | 0.8 | 0.63 |
| 6_1md132 | 127.8 | 128 | 0.2 | 35.2 | -2.4 | 0 |
| 6_1md133 | 128 | 128.7 | 0.7 | 37.8 | 2.6 | 0.7 |
| 6_1md134 | 128.7 | 130.6 | 1.9 | 35.9 | -1.9 | 0.55 |
| 6_1md135 | 130.6 | 130.8 | 0.2 | 37.2 | 1.3 | 0 |
| 6_1md136 | 130.8 | 135.2 | 4.4 | 36.1 | -1.1 | 0.75 |
| 6_1md137 | 135.2 | 135.5 | 0.3 | 37.9 | 1.9 | 0.51 |
| 6_1md138 | 135.5 | 137.8 | 2.3 | 36.6 | -1.3 | 0.9 |
| 6_1md139 | 137.8 | 138.1 | 0.3 | 37.4 | 0.8 | 0.23 |
| 6_1md140 | 138.1 | 138.4 | 0.3 | 36.3 | -1.1 | 0.61 |
| 6_1md141 | 138.4 | 139.1 | 0.7 | 37.7 | 1.4 | 0.27 |
| 6_1md142 | 139.1 | 139.4 | 0.3 | 36.6 | -1.1 | 0.03 |
| 6_1md143 | 139.4 | 139.6 | 0.2 | 37.4 | 0.8 | 0 |
| 6_1md144 | 139.6 | 141 | 1.4 | 35.7 | -1.8 | 1.38 |
| 6_1md145 | 141 | 141.2 | 0.2 | 38.2 | 2.5 | 0 |
| 6_1md146 | 141.2 | 142.3 | 1.1 | 36.3 | -1.9 | 1.04 |
| 6_1md147 | 142.3 | 142.7 | 0.4 | 38.2 | 1.9 | 0.83 |
| 6_1md148 | 142.7 | 142.9 | 0.2 | 36.2 | -2.0 | 0 |
| 6_1md149 | 142.9 | 145.6 | 2.7 | 38.4 | 2.2 | 1.36 |
| 6_1md150 | 145.6 | 145.8 | 0.2 | 46.7 | 8.3 | 0 |
| 6_1md151 | 145.8 | 146.2 | 0.4 | 38.0 | -8.7 | 2.11 |
| 6_1md152 | 146.2 | 146.5 | 0.3 | 35.6 | -2.4 | 1.35 |
| 6_1md153 | 146.5 | 147.6 | 1.1 | 38.2 | 2.7 | 0.95 |
| | | | | | | |
| 6_2md1 | 0 | 0.2 | 0.2 | 41.3 | | 0 |
| 6_2md2 | 0.2 | 0.5 | 0.3 | 42.3 | 1.0 | 0.8 |
| 6_2md3 | 0.5 | 1.5 | 1 | 38.7 | -3.5 | 1.4 |
| 6_2md4 | 1.5 | 1.7 | 0.2 | 42.7 | 4.0 | 0 |
| 6_2md5 | 1.7 | 2.6 | 0.9 | 39.5 | -3.2 | 2.9 |
| 6_2md6 | 2.6 | 2.9 | 0.3 | 36.9 | -2.6 | 1.16 |
| 6_2md7 | 2.9 | 3.1 | 0.2 | 43.7 | 6.8 | 0 |
| 6_2md8 | 3.1 | 3.4 | 0.3 | 39.3 | -4.4 | 1.88 |
| 6_2md9 | 3.4 | 4.1 | 0.7 | 43.2 | 3.8 | 1.76 |
| 6_2md10 | 4.1 | 4.6 | 0.5 | 38.8 | -4.4 | 1.87 |
| 6_2md11 | 4.6 | 5.3 | 0.7 | 35.6 | -3.2 | 0.75 |
| 6_2md12 | 5.3 | 7.3 | 2 | 38.7 | 3.2 | 1.26 |
| 6_2md13 | 7.3 | 7.5 | 0.2 | 41.7 | 3.0 | 0 |
| 6_2md14 | 7.5 | 9.1 | 1.6 | 39.0 | -2.8 | 2.25 |
| 6_2md15 | 9.1 | 10.3 | 1.2 | 43.2 | 4.2 | 2.64 |
| 6_2md16 | 10.3 | 10.6 | 0.3 | 36.3 | -6.9 | 0.73 |
| 6_2md17 | 10.6 | 11 | 0.4 | 39.0 | 2.7 | 1.44 |

| | | | | | | |
|---|---|---|---|---|---|---|
| 6_2md18 | 11 | 11.8 | 0.8 | 42.9 | 3.9 | 1.37 |
| 6_2md19 | 11.8 | 12.6 | 0.8 | 38.3 | -4.6 | 0.93 |
| 6_2md20 | 12.6 | 12.8 | 0.2 | 36.2 | -2.1 | 0 |
| 6_2md21 | 12.8 | 14 | 1.2 | 38.4 | 2.2 | 1.17 |
| 6_2md22 | 14 | 14.3 | 0.3 | 36.5 | -1.9 | 0.11 |
| 6_2md23 | 14.3 | 14.5 | 0.2 | 37.8 | 1.3 | 0 |
| 6_2md24 | 14.5 | 14.7 | 0.2 | 36.4 | -1.3 | 0 |
| 6_2md25 | 14.7 | 16.2 | 1.5 | 38.4 | 1.9 | 1.34 |
| 6_2md26 | 16.2 | 16.6 | 0.4 | 36.0 | -2.4 | 0.44 |
| 6_2md27 | 16.6 | 17 | 0.4 | 37.1 | 1.2 | 0.28 |
| 6_2md28 | 17 | 18.5 | 1.5 | 35.9 | -1.2 | 0.91 |
| 6_2md29 | 18.5 | 19.2 | 0.7 | 37.9 | 2.0 | 0.89 |
| 6_2md30 | 19.2 | 20.8 | 1.6 | 36.3 | -1.6 | 1.07 |
| 6_2md31 | 20.8 | 21 | 0.2 | 37.5 | 1.2 | 0 |
| 6_2md32 | 21 | 21.4 | 0.4 | 34.5 | -3.1 | 0.82 |
| 6_2md33 | 21.4 | 22.3 | 0.9 | 38.0 | 3.5 | 1.05 |
| 6_2md34 | 22.3 | 30 | 7.7 | 34.8 | -3.2 | 1.03 |
| 6_2md35 | 30 | 30.5 | 0.5 | 37.5 | 2.8 | 0.76 |
| 6_2md36 | 30.5 | 31.6 | 1.1 | 36.2 | -1.3 | 0.98 |
| 6_2md37 | 31.6 | 31.8 | 0.2 | 37.5 | 1.3 | 0 |
| 6_2md38 | 31.8 | 32.5 | 0.7 | 35.0 | -2.5 | 0.83 |
| 6_2md39 | 32.5 | 33.1 | 0.6 | 39.0 | 4.0 | 1.21 |
| 6_2md40 | 33.1 | 33.5 | 0.4 | 35.4 | -3.6 | 0.92 |
| 6_2md41 | 33.5 | 33.7 | 0.2 | 37.7 | 2.3 | 0 |
| 6_2md42 | 33.7 | 35.9 | 2.2 | 35.5 | -2.1 | 1.08 |
| 6_2md43 | 35.9 | 36.1 | 0.2 | 38.8 | 3.3 | 0 |
| 6_2md44 | 36.1 | 38.1 | 2 | 36.3 | -2.5 | 0.84 |
| 6_2md45 | 38.1 | 38.6 | 0.5 | 38.6 | 2.3 | 0.8 |
| 6_2md46 | 38.6 | 38.8 | 0.2 | 36.2 | -2.4 | 0 |
| 6_2md47 | 38.8 | 39.4 | 0.6 | 38.1 | 1.9 | 0.52 |
| 6_2md48 | 39.4 | 45.3 | 5.9 | 35.1 | -2.9 | 0.88 |
| 6_2md49 | 45.3 | 45.8 | 0.5 | 38.5 | 3.4 | 1.19 |
| 6_2md50 | 45.8 | 46 | 0.2 | 36.6 | -1.9 | 0 |
| 6_2md51 | 46 | 46.6 | 0.6 | 37.7 | 1.0 | 0.99 |
| 6_2md52 | 46.6 | 46.9 | 0.3 | 41.9 | 4.3 | 0.52 |
| 6_2md53 | 46.9 | 48.1 | 1.2 | 39.1 | -2.8 | 1.27 |
| 6_2md54 | 48.1 | 48.4 | 0.3 | 35.9 | -3.3 | 0.85 |
| 6_2md55 | 48.4 | 49.4 | 1 | 38.0 | 2.1 | 1.6 |
| 6_2md56 | 49.4 | 49.6 | 0.2 | 42.9 | 4.9 | 0 |
| 6_2md57 | 49.6 | 49.8 | 0.2 | 39.2 | -3.7 | 0 |
| 6_2md58 | 49.8 | 52.1 | 2.3 | 42.2 | 3.0 | 1.68 |
| 6_2md59 | 52.1 | 52.9 | 0.8 | 38.7 | -3.5 | 1.13 |
| 6_2md60 | 52.9 | 53.1 | 0.2 | 43.9 | 5.2 | 0 |
| 6_2md61 | 53.1 | 53.9 | 0.8 | 41.8 | -2.1 | 3.4 |
| 6_2md62 | 53.9 | 54.4 | 0.5 | 38.0 | -3.8 | 0.93 |
| 6_2md63 | 54.4 | 54.9 | 0.5 | 42.3 | 4.2 | 1.84 |
| 6_2md64 | 54.9 | 55.5 | 0.6 | 39.4 | -2.9 | 1.02 |
| 6_2md65 | 55.5 | 56.4 | 0.9 | 42.7 | 3.3 | 2.91 |
| 6_2md66 | 56.4 | 57 | 0.6 | 47.7 | 5.1 | 3.79 |
| 6_2md67 | 57 | 58 | 1 | 42.0 | -5.7 | 1.15 |
| 6_2md68 | 58 | 58.5 | 0.5 | 35.9 | -6.1 | 0.97 |
| 6_2md69 | 58.5 | 59.2 | 0.7 | 43.1 | 7.3 | 1.71 |
| 6_2md70 | 59.2 | 60.1 | 0.9 | 38.8 | -4.4 | 1.55 |
| 6_2md71 | 60.1 | 60.3 | 0.2 | 36.4 | -2.4 | 0 |
| 6_2md72 | 60.3 | 61.5 | 1.2 | 42.2 | 5.9 | 2.15 |
| 6_2md73 | 61.5 | 61.9 | 0.4 | 46.4 | 4.2 | 2.97 |

| | | | | | | |
|---|---|---|---|---|---|---|
| 6_2md74 | 61.9 | 62.3 | 0.4 | 43.2 | -3.2 | 1.31 |
| 6_2md75 | 62.3 | 62.8 | 0.5 | 38.5 | -4.7 | 0.41 |
| 6_2md76 | 62.8 | 63 | 0.2 | 36.7 | -1.8 | 0 |
| 6_2md77 | 63 | 67.2 | 4.2 | 38.4 | 1.6 | 1.39 |
| 6_2md78 | 67.2 | 67.9 | 0.7 | 36.7 | -1.7 | 0.69 |
| 6_2md79 | 67.9 | 68.5 | 0.6 | 37.9 | 1.2 | 0.65 |
| 6_2md80 | 68.5 | 69.1 | 0.6 | 42.6 | 4.7 | 3.13 |
| 6_2md81 | 69.1 | 70.9 | 1.8 | 39.7 | -3.0 | 0.77 |
| 6_2md82 | 70.9 | 71.2 | 0.3 | 41.2 | 1.5 | 0.15 |
| 6_2md83 | 71.2 | 71.7 | 0.5 | 38.7 | -2.5 | 0.69 |
| 6_2md84 | 71.7 | 71.9 | 0.2 | 42.1 | 3.4 | 0 |
| 6_2md85 | 71.9 | 73 | 1.1 | 38.4 | -3.7 | 0.99 |
| 6_2md86 | 73 | 73.8 | 0.8 | 36.1 | -2.3 | 0.83 |
| 6_2md87 | 73.8 | 76.2 | 2.4 | 38.8 | 2.7 | 1.39 |
| 6_2md88 | 76.2 | 78.2 | 2 | 36.4 | -2.4 | 0.76 |
| 6_2md89 | 78.2 | 80.6 | 2.4 | 39.6 | 3.2 | 0.96 |
| 6_2md90 | 80.6 | 86.5 | 5.9 | 34.9 | -4.6 | 0.93 |
| 6_2md91 | 86.5 | 87.1 | 0.6 | 37.1 | 2.2 | 0.33 |
| 6_2md92 | 87.1 | 87.8 | 0.7 | 36.8 | -0.3 | 0.28 |
| 6_2md93 | 87.8 | 88.1 | 0.3 | 37.4 | 0.7 | 0.16 |
| 6_2md94 | 88.1 | 88.3 | 0.2 | 36.5 | -1.0 | 0 |
| 6_2md95 | 88.3 | 89.8 | 1.5 | 38.8 | 2.3 | 1.09 |
| 6_2md96 | 89.8 | 93.6 | 3.8 | 35.5 | -3.3 | 0.84 |
| 6_2md97 | 93.6 | 93.9 | 0.3 | 39.2 | 3.8 | 0.32 |
| 6_2md98 | 93.9 | 94.1 | 0.2 | 41.3 | 2.1 | 0 |
| 6_2md99 | 94.1 | 95 | 0.9 | 39.0 | -2.3 | 1.76 |
| 6_2md100 | 95 | 95.6 | 0.6 | 43.2 | 4.2 | 3.3 |
| 6_2md101 | 95.6 | 98.7 | 3.1 | 39.8 | -3.4 | 1.13 |
| 6_2md102 | 98.7 | 99.4 | 0.7 | 42.6 | 2.8 | 1.89 |
| 6_2md103 | 99.4 | 99.7 | 0.3 | 37.2 | -5.4 | 1.28 |
| 6_2md104 | 99.7 | 100.9 | 1.2 | 39.6 | 2.4 | 1.01 |
| 6_2md105 | 100.9 | 101.3 | 0.4 | 41.1 | 1.5 | 0.7 |
| 6_2md106 | 101.3 | 102.6 | 1.3 | 40.3 | -0.8 | 0.98 |
| 6_2md107 | 102.6 | 102.9 | 0.3 | 41.9 | 1.6 | 0.43 |
| 6_2md108 | 102.9 | 103.4 | 0.5 | 38.0 | -3.9 | 1.19 |
| 6_2md109 | 103.4 | 103.6 | 0.2 | 43.6 | 5.6 | 0 |
| 6_2md110 | 103.6 | 103.8 | 0.2 | 51.0 | 7.4 | 0 |
| 6_2md111 | 103.8 | 104 | 0.2 | 43.7 | -7.3 | 0 |
| 6_2md112 | 104 | 104.3 | 0.3 | 39.8 | -3.9 | 0.17 |
| 6_2md113 | 104.3 | 104.6 | 0.3 | 43.1 | 3.3 | 1.57 |
| 6_2md114 | 104.6 | 105.6 | 1 | 39.2 | -3.9 | 1.2 |
| 6_2md115 | 105.6 | 105.9 | 0.3 | 43.1 | 3.9 | 0.39 |
| 6_2md116 | 105.9 | 106.8 | 0.9 | 40.1 | -3.0 | 1.2 |
| 6_2md117 | 106.8 | 107 | 0.2 | 42.2 | 2.1 | 0 |
| 6_2md118 | 107 | 108.6 | 1.6 | 39.3 | -2.9 | 1.12 |
| 6_2md119 | 108.6 | 108.8 | 0.2 | 41.5 | 2.2 | 0 |
| 6_2md120 | 108.8 | 109.1 | 0.3 | 37.5 | -4.0 | 0.5 |
| 6_2md121 | 109.1 | 109.6 | 0.5 | 39.3 | 1.8 | 0.77 |
| 6_2md122 | 109.6 | 109.8 | 0.2 | 42.5 | 3.2 | 0 |
| 6_2md123 | 109.8 | 110.4 | 0.6 | 37.0 | -5.5 | 1.65 |
| 6_2md124 | 110.4 | 111.5 | 1.1 | 39.3 | 2.3 | 1.3 |
| 6_2md125 | 111.5 | 111.7 | 0.2 | 41.4 | 2.1 | 0 |
| 6_2md126 | 111.7 | 112.5 | 0.8 | 38.1 | -3.3 | 0.59 |
| 6_2md127 | 112.5 | 118.7 | 6.2 | 34.6 | -3.5 | 0.87 |
| 6_2md128 | 118.7 | 119.6 | 0.9 | 37.5 | 2.9 | 0.89 |
| 6_2md129 | 119.6 | 127.2 | 7.6 | 35.5 | -1.9 | 0.92 |

| | | | | | | |
|---|---|---|---|---|---|---|
| 6_2md130 | 127.2 | 128.9 | 1.7 | 39.1 | 3.6 | 2.1 |
| 6_2md131 | 128.9 | 129.6 | 0.7 | 43.8 | 4.7 | 2.22 |
| 6_2md132 | 129.6 | 129.9 | 0.3 | 38.7 | -5.1 | 1.29 |
| 6_2md133 | 129.9 | 130.2 | 0.3 | 44.1 | 5.5 | 1.87 |
| 6_2md134 | 130.2 | 130.4 | 0.2 | 48.9 | 4.7 | 0 |
| 6_2md135 | 130.4 | 131 | 0.6 | 38.5 | -10.4 | 1.94 |
| 6_2md136 | 131 | 131.3 | 0.3 | 34.9 | -3.6 | 0.68 |
| 6_2md137 | 131.3 | 131.5 | 0.2 | 48.9 | 14.0 | 0 |
| 6_2md138 | 131.5 | 131.7 | 0.2 | 45.7 | -3.1 | 0 |
| 6_2md139 | 131.7 | 132 | 0.3 | 47.1 | 1.4 | 0.17 |
| 6_2md140 | 132 | 132.7 | 0.7 | 42.7 | -4.4 | 1.39 |
| 6_2md141 | 132.7 | 132.9 | 0.2 | 39.9 | -2.7 | 0 |
| 6_2md142 | 132.9 | 133.4 | 0.5 | 42.5 | 2.6 | 1.09 |
| 6_2md143 | 133.4 | 134.6 | 1.2 | 39.5 | -3.0 | 1.4 |
| 6_2md144 | 134.6 | 134.9 | 0.3 | 42.0 | 2.5 | 1.01 |
| 6_2md145 | 134.9 | 135.1 | 0.2 | 40.9 | -1.1 | 0 |
| 6_2md146 | 135.1 | 136.6 | 1.5 | 45.1 | 4.2 | 3.29 |
| 6_2md147 | 136.6 | 136.8 | 0.2 | 46.7 | 1.6 | 0 |
| 6_2md148 | 136.8 | 137 | 0.2 | 40.7 | -6.0 | 0 |
| 6_2md149 | 137 | 137.8 | 0.8 | 43.0 | 2.4 | 2.73 |
| 6_2md150 | 137.8 | 138.1 | 0.3 | 50.4 | 7.4 | 2.06 |
| 6_2md151 | 138.1 | 139.1 | 1 | 44.3 | -6.2 | 1.35 |
| 6_2md152 | 139.1 | 139.8 | 0.7 | 39.4 | -4.9 | 0.79 |
| 6_2md153 | 139.8 | 140.2 | 0.4 | 47.1 | 7.7 | 3.37 |
| 6_2md154 | 140.2 | 140.4 | 0.2 | 41.9 | -5.2 | 0 |
| 6_2md155 | 140.4 | 140.8 | 0.4 | 52.4 | 10.5 | 2.43 |
| 6_2md156 | 140.8 | 141.2 | 0.4 | 40.9 | -11.5 | 2.08 |
| 6_2md157 | 141.2 | 142.3 | 1.1 | 52.9 | 12.0 | 3.35 |
| 6_2md158 | 142.3 | 142.9 | 0.6 | 49.8 | -3.1 | 3.31 |
| 6_2md159 | 142.9 | 143.3 | 0.4 | 45.2 | -4.6 | 1.11 |
| 6_2md160 | 143.3 | 143.7 | 0.4 | 53.0 | 7.8 | 3.56 |
| 6_2md161 | 143.7 | 144.3 | 0.6 | 42.4 | -10.5 | 2.38 |
| 6_2md162 | 144.3 | 144.6 | 0.3 | 48.6 | 6.2 | 2.61 |
| | | | | | | |
| 7md1 | 0 | 1.4 | 1.4 | 38.4 | | 1.93 |
| 7md2 | 1.4 | 1.8 | 0.4 | 36.8 | -1.6 | 0.7 |
| 7md3 | 1.8 | 2.4 | 0.6 | 43.3 | 6.5 | 1.7 |
| 7md4 | 2.4 | 3 | 0.6 | 38.6 | -4.7 | 1.56 |
| 7md5 | 3 | 4.1 | 1.1 | 35.6 | -3.0 | 0.9 |
| 7md6 | 4.1 | 4.3 | 0.2 | 42.2 | 6.6 | 0 |
| 7md7 | 4.3 | 4.5 | 0.2 | 38.1 | -4.1 | 0 |
| 7md8 | 4.5 | 8.9 | 4.4 | 35.2 | -2.9 | 0.99 |
| 7md9 | 8.9 | 9.2 | 0.3 | 37.5 | 2.4 | 0.35 |
| 7md10 | 9.2 | 9.4 | 0.2 | 35.5 | -2.0 | 0 |
| 7md11 | 9.4 | 9.7 | 0.3 | 38.5 | 3.0 | 0.71 |
| 7md12 | 9.7 | 11.1 | 1.4 | 36.2 | -2.3 | 0.97 |
| 7md13 | 11.1 | 11.5 | 0.4 | 38.5 | 2.3 | 1.34 |
| 7md14 | 11.5 | 11.7 | 0.2 | 36.8 | -1.7 | 0 |
| 7md15 | 11.7 | 11.9 | 0.2 | 38.2 | 1.4 | 0 |
| 7md16 | 11.9 | 13.9 | 2 | 35.5 | -2.7 | 0.8 |
| 7md17 | 13.9 | 14.5 | 0.6 | 38.1 | 2.6 | 1.32 |
| 7md18 | 14.5 | 15.6 | 1.1 | 34.8 | -3.3 | 1.06 |
| 7md19 | 15.6 | 15.6 | 0 | 0.0 | -34.8 | 0 |
| 7md20 | 15.7 | 18.6 | 2.9 | 34.7 | 34.7 | 1.05 |
| 7md21 | 18.6 | 20.1 | 1.5 | 38.3 | 3.6 | 1.82 |
| 7md22 | 20.1 | 20.9 | 0.8 | 36.6 | -1.7 | 1.57 |

| | | | | | | |
|---|---|---|---|---|---|---|
| 7md23 | 20.9 | 21.7 | 0.8 | 39.2 | 2.6 | 1.57 |
| 7md24 | 21.7 | 21.9 | 0.2 | 41.1 | 1.9 | 0 |
| 7md25 | 21.9 | 25.2 | 3.3 | 38.0 | -3.1 | 1.11 |
| 7md26 | 25.2 | 26.5 | 1.3 | 36.4 | -1.7 | 0.79 |
| 7md27 | 26.5 | 26.7 | 0.2 | 37.4 | 1.1 | 0 |
| 7md28 | 26.7 | 26.9 | 0.2 | 35.9 | -1.5 | 0 |
| 7md29 | 26.9 | 28.9 | 2 | 37.7 | 1.8 | 0.86 |
| 7md30 | 28.9 | 30.8 | 1.9 | 35.7 | -2.0 | 0.56 |
| 7md31 | 30.8 | 31.4 | 0.6 | 37.2 | 1.5 | 0.24 |
| 7md32 | 31.4 | 32 | 0.6 | 36.1 | -1.1 | 0.94 |
| 7md33 | 32 | 32.2 | 0.2 | 37.5 | 1.4 | 0 |
| 7md34 | 32.2 | 42.5 | 10.3 | 35.4 | -2.1 | 0.95 |
| 7md35 | 42.5 | 45.4 | 2.9 | 0.0 | | 0 |
| 7md36 | 45.4 | 45.7 | 0.3 | 37.1 | | 1.82 |
| 7md37 | 45.7 | 45.9 | 0.2 | 37.7 | 0.7 | 0 |
| 7md38 | 45.9 | 49.3 | 3.4 | 35.8 | -2.0 | 0.7 |
| 7md39 | 49.3 | 49.6 | 0.3 | 37.7 | 1.9 | 0.73 |
| 7md40 | 49.6 | 52.1 | 2.5 | 35.9 | -1.8 | 0.87 |
| 7md41 | 52.1 | 52.4 | 0.3 | 37.6 | 1.8 | 0.23 |
| 7md42 | 52.4 | 52.7 | 0.3 | 36.0 | -1.6 | 0.3 |
| 7md43 | 52.7 | 53.5 | 0.8 | 37.7 | 1.7 | 0.57 |
| 7md44 | 53.5 | 57.8 | 4.3 | 35.4 | -2.3 | 0.89 |
| 7md45 | 57.8 | 58.5 | 0.7 | 37.7 | 2.3 | 0.63 |
| 7md46 | 58.5 | 58.7 | 0.2 | 35.6 | -2.1 | 0 |
| 7md47 | 58.7 | 59.3 | 0.6 | 37.7 | 2.1 | 0.29 |
| 7md48 | 59.3 | 59.6 | 0.3 | 35.5 | -2.1 | 0.7 |
| 7md49 | 59.6 | 60.5 | 0.9 | 37.9 | 2.3 | 1.35 |
| 7md50 | 60.5 | 61 | 0.5 | 36.8 | -1.1 | 0.99 |
| 7md51 | 61 | 61.4 | 0.4 | 37.8 | 1.0 | 0.97 |
| 7md52 | 61.4 | 61.8 | 0.4 | 36.1 | -1.7 | 0.52 |
| 7md53 | 61.8 | 63.1 | 1.3 | 37.7 | 1.6 | 1.05 |
| 7md54 | 63.1 | 63.7 | 0.6 | 37.0 | -0.7 | 1.03 |
| 7md55 | 63.7 | 64.5 | 0.8 | 39.4 | 2.5 | 1.2 |
| 7md56 | 64.5 | 64.7 | 0.2 | 41.8 | 2.3 | 0 |
| 7md57 | 64.7 | 65.7 | 1 | 37.8 | -3.9 | 0.61 |
| 7md58 | 65.7 | 66.1 | 0.4 | 36.1 | -1.7 | 0.59 |
| 7md59 | 66.1 | 66.3 | 0.2 | 37.4 | 1.3 | 0 |
| 7md60 | 66.3 | 69.1 | 2.8 | 35.2 | -2.2 | 0.85 |
| 7md61 | 69.1 | 69.3 | 0.2 | 38.1 | 2.9 | 0 |
| 7md62 | 69.3 | 69.5 | 0.2 | 36.0 | -2.0 | 0 |
| 7md63 | 69.5 | 70.3 | 0.8 | 37.8 | 1.7 | 0.88 |
| 7md64 | 70.3 | 70.5 | 0.2 | 35.5 | -2.3 | 0 |
| 7md65 | 70.5 | 70.9 | 0.4 | 37.9 | 2.4 | 0.77 |
| 7md66 | 70.9 | 73.7 | 2.8 | 36.2 | -1.6 | 0.87 |
| 7md67 | 73.7 | 73.9 | 0.2 | 37.8 | 1.6 | 0 |
| 7md68 | 73.9 | 74.1 | 0.2 | 36.0 | -1.8 | 0 |
| 7md69 | 74.1 | 75.7 | 1.6 | 38.6 | 2.6 | 1.06 |
| 7md70 | 75.7 | 75.9 | 0.2 | 35.8 | -2.8 | 0 |
| 7md71 | 75.9 | 78.8 | 2.9 | 38.4 | 2.5 | 1.18 |
| 7md72 | 78.8 | 79.1 | 0.3 | 35.3 | -3.1 | 1.31 |
| 7md73 | 79.1 | 79.8 | 0.7 | 37.7 | 2.4 | 0.37 |
| 7md74 | 79.8 | 80.7 | 0.9 | 36.1 | -1.6 | 0.76 |
| 7md75 | 80.7 | 81.2 | 0.5 | 36.3 | 0.2 | 1.09 |
| 7md76 | 81.2 | 82.2 | 1 | 38.1 | 1.8 | 1.08 |
| 7md77 | 82.2 | 82.6 | 0.4 | 35.8 | -2.3 | 0.56 |
| 7md78 | 82.6 | 82.9 | 0.3 | 37.5 | 1.7 | 0.34 |

| | | | | | | |
|---|---|---|---|---|---|---|
| 7md79 | 82.9 | 83.9 | 1 | 36.2 | -1.2 | 0.41 |
| 7md80 | 83.9 | 84.3 | 0.4 | 37.3 | 1.1 | 0.12 |
| 7md81 | 84.3 | 94.2 | 9.9 | 34.9 | -2.4 | 1.14 |
| 7md82 | 94.2 | 94.4 | 0.2 | 37.4 | 2.4 | 0 |
| 7md83 | 94.4 | 97.6 | 3.2 | 35.8 | -1.6 | 0.95 |
| 7md84 | 97.6 | 98.4 | 0.8 | 38.0 | 2.2 | 1.43 |
| 7md85 | 98.4 | 98.6 | 0.2 | 36.3 | -1.7 | 0 |
| 7md86 | 98.6 | 99.4 | 0.8 | 37.2 | 1.0 | 0.91 |
| 7md87 | 99.4 | 101.5 | 2.1 | 36.6 | -0.7 | 0.81 |
| 7md88 | 101.5 | 101.7 | 0.2 | 37.5 | 1.0 | 0 |
| 7md89 | 101.7 | 103.7 | 2 | 35.8 | -1.7 | 0.86 |
| 7md90 | 103.7 | 104.6 | 0.9 | 37.5 | 1.7 | 0.82 |
| 7md91 | 104.6 | 137.1 | 32.5 | 34.6 | -2.9 | 1.12 |
| 7md92 | 137.1 | 137.3 | 0.2 | 37.5 | 2.9 | 0 |
| 7md93 | 137.3 | 139.4 | 2.1 | 36.0 | -1.4 | 1.04 |
| 7md94 | 139.4 | 139.6 | 0.2 | 38.0 | 2.0 | 0 |
| 7md95 | 139.6 | 148.3 | 8.7 | 34.7 | -3.3 | 1.19 |
| 7md96 | 148.3 | 148.3 | 0 | 0.0 | -34.7 | 0 |
| 7md97 | 148.4 | 155.1 | 6.7 | 34.5 | 34.5 | 1.07 |
| 7md98 | 155.1 | 155.3 | 0.2 | 37.6 | 3.0 | 0 |
| 7md99 | 155.3 | 163.9 | 8.6 | 34.5 | -3.1 | 0.84 |
| 7md100 | 163.9 | 164.8 | 0.9 | 38.1 | 3.6 | 1.24 |
| 7md101 | 164.8 | 165 | 0.2 | 36.0 | -2.1 | 0 |
| 7md102 | 165 | 165.4 | 0.4 | 38.0 | 2.1 | 0.98 |
| 7md103 | 165.4 | 166.6 | 1.2 | 36.0 | -2.0 | 1.07 |
| 7md104 | 166.6 | 167.5 | 0.9 | 37.8 | 1.8 | 0.58 |
| 7md105 | 167.5 | 168.1 | 0.6 | 36.2 | -1.6 | 0.76 |
| 7md106 | 168.1 | 169.7 | 1.6 | 38.4 | 2.2 | 0.92 |
| 7md107 | 169.7 | 169.9 | 0.2 | 36.3 | -2.1 | 0 |
| 7md108 | 169.9 | 170.7 | 0.8 | 38.2 | 1.9 | 0.95 |
| 7md109 | 170.7 | 170.9 | 0.2 | 35.9 | -2.2 | 0 |
| 7md110 | 170.9 | 171.2 | 0.3 | 37.4 | 1.4 | 0.37 |
| 7md111 | 171.2 | 171.4 | 0.2 | 35.6 | -1.8 | 0 |
| 7md112 | 171.4 | 173 | 1.6 | 38.0 | 2.3 | 0.78 |
| 7md113 | 173 | 173.2 | 0.2 | 48.5 | 10.6 | 0 |
| 7md114 | 173.2 | 175 | 1.8 | 42.6 | -5.9 | 2.33 |
| 7md115 | 175 | 177.9 | 2.9 | 38.6 | -4.0 | 1.49 |
| 7md116 | 177.9 | 181.2 | 3.3 | 35.3 | -3.3 | 1.2 |
| 7md117 | 181.2 | 181.4 | 0.2 | 37.4 | 2.1 | 0 |
| 7md118 | 181.4 | 186.8 | 5.4 | 35.5 | -1.9 | 1.22 |
| 7md119 | 186.8 | 187.3 | 0.5 | 37.6 | 2.1 | 0.85 |
| 7md120 | 187.3 | 187.6 | 0.3 | 36.5 | -1.0 | 0.27 |
| 7md121 | 187.6 | 188.5 | 0.9 | 37.7 | 1.2 | 0.47 |
| 7md122 | 188.5 | 189.2 | 0.7 | 36.3 | -1.4 | 0.77 |
| 7md123 | 189.2 | 189.8 | 0.6 | 38.1 | 1.8 | 1.12 |
| 7md124 | 189.8 | 190 | 0.2 | 41.6 | 3.5 | 0 |
| 7md125 | 190 | 190.2 | 0.2 | 38.8 | -2.8 | 0 |
| 7md126 | 190.2 | 191.4 | 1.2 | 34.8 | -4.0 | 1.26 |
| 7md127 | 191.4 | 192 | 0.6 | 37.9 | 3.1 | 0.33 |
| 7md128 | 192 | 192.3 | 0.3 | 36.6 | -1.2 | 0.3 |
| 7md129 | 192.3 | 192.5 | 0.2 | 37.5 | 0.9 | 0 |
| 7md130 | 192.5 | 193.7 | 1.2 | 36.1 | -1.3 | 0.81 |
| 7md131 | 193.7 | 195 | 1.3 | 37.8 | 1.7 | 1.29 |
| 7md132 | 195 | 195.3 | 0.3 | 42.5 | 4.7 | 1.1 |
| 7md133 | 195.3 | 196.1 | 0.8 | 39.2 | -3.3 | 1.24 |
| 7md134 | 196.1 | 196.4 | 0.3 | 36.5 | -2.7 | 0.19 |

| | | | | | | |
|---|---|---|---|---|---|---|
| 7md135 | 196.4 | 196.7 | 0.3 | 37.3 | 0.8 | 0.26 |
| 7md136 | 196.7 | 199.2 | 2.5 | 36.2 | -1.1 | 0.82 |
| 7md137 | 199.2 | 199.4 | 0.2 | 37.4 | 1.2 | 0 |
| 7md138 | 199.4 | 200 | 0.6 | 36.7 | -0.7 | 0.51 |
| 7md139 | 200 | 200.9 | 0.9 | 39.4 | 2.7 | 2.23 |
| 7md140 | 200.9 | 201.1 | 0.2 | 35.9 | -3.5 | 0 |
| 7md141 | 201.1 | 204.4 | 3.3 | 38.5 | 2.5 | 1.03 |
| 7md142 | 204.4 | 204.7 | 0.3 | 35.8 | -2.7 | 0.5 |
| 7md143 | 204.7 | 206.3 | 1.6 | 39.4 | 3.7 | 1.3 |
| 7md144 | 206.3 | 206.8 | 0.5 | 42.2 | 2.8 | 0.58 |
| 7md145 | 206.8 | 210.3 | 3.5 | 38.9 | -3.3 | 1.32 |
| 7md146 | 210.3 | 215.5 | 5.2 | 35.4 | -3.5 | 1.07 |
| 7md147 | 215.5 | 217.2 | 1.7 | 37.9 | 2.5 | 1.23 |
| 7md148 | 217.2 | 217.7 | 0.5 | 35.9 | -2.0 | 0.91 |
| 7md149 | 217.7 | 218.9 | 1.2 | 38.3 | 2.4 | 1.67 |
| 7md150 | 218.9 | 219.1 | 0.2 | 36.2 | -2.1 | 0 |
| 7md151 | 219.1 | 220 | 0.9 | 38.1 | 1.8 | 0.81 |
| 7md152 | 220 | 221.1 | 1.1 | 36.1 | -1.9 | 0.75 |
| 7md153 | 221.1 | 222.8 | 1.7 | 37.5 | 1.4 | 0.81 |
| 7md154 | 222.8 | 225.3 | 2.5 | 35.9 | -1.6 | 1.07 |
| 7md155 | 225.3 | 226.2 | 0.9 | 37.6 | 1.7 | 0.87 |
| 7md156 | 226.2 | 226.8 | 0.6 | 36.8 | -0.9 | 0.65 |
| 7md157 | 226.8 | 228.6 | 1.8 | 37.5 | 0.8 | 0.6 |
| 7md158 | 228.6 | 229.7 | 1.1 | 35.4 | -2.1 | 0.69 |
| 7md159 | 229.7 | 231.8 | 2.1 | 39.6 | 4.2 | 1.18 |
| 7md160 | 231.8 | 232.3 | 0.5 | 42.0 | 2.4 | 1.61 |
| 7md161 | 232.3 | 235.7 | 3.4 | 38.5 | -3.5 | 1.31 |
| 7md162 | 235.7 | 241.5 | 5.8 | 34.9 | -3.6 | 0.95 |
| 7md163 | 241.5 | 242.1 | 0.6 | 38.5 | 3.6 | 1.19 |
| 7md164 | 242.1 | 242.3 | 0.2 | 44.2 | 5.7 | 0 |
| 7md165 | 242.3 | 242.5 | 0.2 | 49.8 | 5.7 | 0 |
| 7md166 | 242.5 | 243.4 | 0.9 | 43.0 | -6.9 | 2.23 |
| 7md167 | 243.4 | 243.7 | 0.3 | 36.6 | -6.4 | 0.39 |
| 7md168 | 243.7 | 244.7 | 1 | 39.2 | 2.6 | 0.55 |
| 7md169 | 244.7 | 245.4 | 0.7 | 42.7 | 3.5 | 1.15 |
| 7md170 | 245.4 | 245.8 | 0.4 | 39.7 | -3.0 | 1.3 |
| 7md171 | 245.8 | 246 | 0.2 | 42.3 | 2.6 | 0 |
| 7md172 | 246 | 246.9 | 0.9 | 40.2 | -2.1 | 0.68 |
| 7md173 | 246.9 | 247.6 | 0.7 | 36.4 | -3.8 | 0.86 |
| 7md174 | 247.6 | 248.3 | 0.7 | 38.9 | 2.5 | 1.58 |
| 7md175 | 248.3 | 248.5 | 0.2 | 36.3 | -2.6 | 0 |
| 7md176 | 248.5 | 250.6 | 2.1 | 38.9 | 2.6 | 1.13 |
| 7md177 | 250.6 | 250.9 | 0.3 | 50.1 | 11.2 | 5.48 |
| 7md178 | 250.9 | 251.2 | 0.3 | 44.8 | -5.3 | 1.69 |
| 7md179 | 251.2 | 252.5 | 1.3 | 48.5 | 3.7 | 3.68 |
| 7md180 | 252.5 | 253.2 | 0.7 | 44.0 | -4.5 | 1.82 |
| 7md181 | 253.2 | 254.4 | 1.2 | 48.7 | 4.7 | 3.5 |
| 7md182 | 254.4 | 254.6 | 0.2 | 42.5 | -6.2 | 0 |
| 7md183 | 254.6 | 255.1 | 0.5 | 39.8 | -2.7 | 1.02 |
| 7md184 | 255.1 | 255.3 | 0.2 | 36.4 | -3.3 | 0 |
| 7md185 | 255.3 | 256 | 0.7 | 39.5 | 3.1 | 2.88 |
| 7md186 | 256 | 256.2 | 0.2 | 35.5 | -4.0 | 0 |
| 7md187 | 256.2 | 256.6 | 0.4 | 38.8 | 3.3 | 1.16 |
| 7md188 | 256.6 | 257.2 | 0.6 | 41.6 | 2.9 | 1.4 |
| 7md189 | 257.2 | 257.4 | 0.2 | 47.6 | 6.0 | 0 |
| 7md190 | 257.4 | 257.7 | 0.3 | 43.3 | -4.3 | 1.29 |

| | | | | | | |
|---|---|---|---|---|---|---|
| 7md191 | 257.7 | 257.9 | 0.2 | 39.7 | -3.6 | 0 |
| 7md192 | 257.9 | 258.1 | 0.2 | 41.2 | 1.5 | 0 |
| 7md193 | 258.1 | 258.5 | 0.4 | 40.7 | -0.5 | 2.1 |
| 7md194 | 258.5 | 258.8 | 0.3 | 47.5 | 6.7 | 1.03 |
| 7md195 | 258.8 | 259.2 | 0.4 | 43.3 | -4.2 | 1.09 |
| 7md196 | 259.2 | 260 | 0.8 | 39.3 | -4.0 | 0.86 |
| 7md197 | 260 | 260.6 | 0.6 | 51.6 | 12.4 | 5.62 |
| 7md198 | 260.6 | 260.9 | 0.3 | 42.8 | -8.8 | 0.63 |
| | | | | | | |
| 8_1md1 | 0 | 3 | 3 | 65.0 | | 0 |
| 8_1md2 | 3 | 3.6 | 0.6 | 44.1 | | 2.99 |
| 8_1md3 | 3.6 | 4.9 | 1.3 | 38.7 | -5.4 | 1.26 |
| 8_1md4 | 4.9 | 5.3 | 0.4 | 42.9 | 4.2 | 0.77 |
| 8_1md5 | 5.3 | 7.1 | 1.8 | 38.8 | -4.1 | 1.48 |
| 8_1md6 | 7.1 | 7.4 | 0.3 | 43.9 | 5.1 | 0.78 |
| 8_1md7 | 7.4 | 8.4 | 1 | 48.2 | 4.3 | 3.24 |
| 8_1md8 | 8.4 | 9.6 | 1.2 | 43.1 | -5.1 | 1.89 |
| 8_1md9 | 9.6 | 10.5 | 0.9 | 48.3 | 5.2 | 4.12 |
| 8_1md10 | 10.5 | 10.7 | 0.2 | 45.1 | -3.1 | 0 |
| 8_1md11 | 10.7 | 11.2 | 0.5 | 48.1 | 3.0 | 1.68 |
| 8_1md12 | 11.2 | 11.6 | 0.4 | 42.2 | -5.9 | 1.47 |
| 8_1md13 | 11.6 | 12.1 | 0.5 | 52.9 | 10.7 | 1.5 |
| 8_1md14 | 12.1 | 13.3 | 1.2 | 49.7 | -3.2 | 3.79 |
| 8_1md15 | 13.3 | 13.7 | 0.4 | 55.3 | 5.6 | 2.2 |
| 8_1md16 | 13.7 | 14.1 | 0.4 | 41.7 | -13.6 | 2.67 |
| 8_1md17 | 14.1 | 14.9 | 0.8 | 48.5 | 6.8 | 5.17 |
| 8_1md18 | 14.9 | 15.1 | 0.2 | 54.6 | 6.1 | 0 |
| 8_1md19 | 15.1 | 15.3 | 0.2 | 43.7 | -11.0 | 0 |
| 8_1md20 | 15.3 | 15.7 | 0.4 | 48.5 | 4.8 | 3.08 |
| 8_1md21 | 15.7 | 16.1 | 0.4 | 43.7 | -4.8 | 1.29 |
| 8_1md22 | 16.1 | 17.9 | 1.8 | 50.2 | 6.5 | 3.2 |
| 8_1md23 | 17.9 | 18.5 | 0.6 | 34.7 | -15.5 | 0.46 |
| 8_1md24 | 18.5 | 18.8 | 0.3 | 38.3 | 3.6 | 1.17 |
| 8_1md25 | 18.8 | 21.6 | 2.8 | 35.2 | -3.1 | 0.6 |
| 8_1md26 | 21.6 | 21.8 | 0.2 | 37.2 | 2.0 | 0 |
| 8_1md27 | 21.8 | 22.2 | 0.4 | 35.7 | -1.5 | 1.38 |
| 8_1md28 | 22.2 | 22.4 | 0.2 | 39.3 | 3.6 | 0 |
| 8_1md29 | 22.4 | 22.9 | 0.5 | 37.9 | -1.4 | 0.76 |
| 8_1md30 | 22.9 | 23.1 | 0.2 | 36.3 | -1.6 | 0 |
| 8_1md31 | 23.1 | 23.4 | 0.3 | 39.1 | 2.7 | 1 |
| 8_1md32 | 23.4 | 24 | 0.6 | 35.9 | -3.2 | 0.65 |
| 8_1md33 | 24 | 24.2 | 0.2 | 37.7 | 1.8 | 0 |
| 8_1md34 | 24.2 | 25.8 | 1.6 | 36.0 | -1.7 | 0.47 |
| 8_1md35 | 25.8 | 26 | 0.2 | 37.5 | 1.5 | 0 |
| 8_1md36 | 26 | 26.2 | 0.2 | 36.4 | -1.0 | 0 |
| 8_1md37 | 26.2 | 27.6 | 1.4 | 38.3 | 1.8 | 1.29 |
| 8_1md38 | 27.6 | 27.8 | 0.2 | 36.5 | -1.8 | 0 |
| 8_1md39 | 27.8 | 29.9 | 2.1 | 39.0 | 2.5 | 2.65 |
| 8_1md40 | 29.9 | 31 | 1.1 | 36.3 | -2.7 | 1.03 |
| 8_1md41 | 31 | 31.4 | 0.4 | 39.4 | 3.1 | 0.61 |
| 8_1md42 | 31.4 | 31.9 | 0.5 | 35.8 | -3.6 | 0.62 |
| 8_1md43 | 31.9 | 32.8 | 0.9 | 38.8 | 3.0 | 1.16 |
| 8_1md44 | 32.8 | 33.2 | 0.4 | 37.0 | -1.7 | 0.49 |
| 8_1md45 | 33.2 | 33.7 | 0.5 | 37.8 | 0.8 | 0.76 |
| 8_1md46 | 33.7 | 34 | 0.3 | 36.1 | -1.7 | 0.47 |
| 8_1md47 | 34 | 35.3 | 1.3 | 38.5 | 2.4 | 1.48 |

| | | | | | | |
|---|---|---|---|---|---|---|
| 8_1md48 | 35.3 | 35.5 | 0.2 | 36.3 | -2.2 | 0 |
| 8_1md49 | 35.5 | 36.3 | 0.8 | 42.5 | 6.2 | 2.81 |
| 8_1md50 | 36.3 | 37.7 | 1.4 | 38.1 | -4.4 | 0.77 |
| 8_1md51 | 37.7 | 37.9 | 0.2 | 35.9 | -2.2 | 0 |
| 8_1md52 | 37.9 | 38.1 | 0.2 | 38.9 | 3.0 | 0 |
| 8_1md53 | 38.1 | 38.3 | 0.2 | 36.7 | -2.2 | 0 |
| 8_1md54 | 38.3 | 38.5 | 0.2 | 38.0 | 1.3 | 0 |
| 8_1md55 | 38.5 | 38.8 | 0.3 | 36.1 | -1.8 | 0.97 |
| 8_1md56 | 38.8 | 40.1 | 1.3 | 37.5 | 1.4 | 1.03 |
| 8_1md57 | 40.1 | 44.2 | 4.1 | 35.0 | -2.5 | 0.87 |
| 8_1md58 | 44.2 | 45.4 | 1.2 | 38.3 | 3.3 | 0.89 |
| 8_1md59 | 45.4 | 45.6 | 0.2 | 35.9 | -2.4 | 0 |
| 8_1md60 | 45.6 | 46.7 | 1.1 | 38.0 | 2.0 | 1.27 |
| 8_1md61 | 46.7 | 49.1 | 2.4 | 35.2 | -2.8 | 0.62 |
| 8_1md62 | 49.1 | 49.6 | 0.5 | 38.6 | 3.4 | 1.16 |
| 8_1md63 | 49.6 | 50.8 | 1.2 | 36.2 | -2.4 | 0.74 |
| 8_1md64 | 50.8 | 51.2 | 0.4 | 37.3 | 1.1 | 0.58 |
| 8_1md65 | 51.2 | 53.2 | 2 | 36.1 | -1.2 | 1.03 |
| 8_1md66 | 53.2 | 53.5 | 0.3 | 37.6 | 1.5 | 0.44 |
| 8_1md67 | 53.5 | 60.6 | 7.1 | 35.0 | -2.5 | 0.96 |
| 8_1md68 | 60.6 | 60.8 | 0.2 | 37.7 | 2.6 | 0 |
| 8_1md69 | 60.8 | 61.2 | 0.4 | 35.8 | -1.8 | 1.14 |
| 8_1md70 | 61.2 | 61.8 | 0.6 | 38.3 | 2.4 | 0.81 |
| 8_1md71 | 61.8 | 62 | 0.2 | 36.3 | -2.0 | 0 |
| 8_1md72 | 62 | 62.7 | 0.7 | 39.7 | 3.4 | 3.14 |
| 8_1md73 | 62.7 | 63.3 | 0.6 | 35.7 | -4.0 | 0.64 |
| 8_1md74 | 63.3 | 63.6 | 0.3 | 40.4 | 4.7 | 1.16 |
| 8_1md75 | 63.6 | 63.8 | 0.2 | 42.9 | 2.5 | 0 |
| 8_1md76 | 63.8 | 64 | 0.2 | 40.3 | -2.6 | 0 |
| 8_1md77 | 64 | 64.6 | 0.6 | 44.7 | 4.4 | 1.39 |
| 8_1md78 | 64.6 | 65.1 | 0.5 | 47.9 | 3.2 | 1.97 |
| 8_1md79 | 65.1 | 65.5 | 0.4 | 42.1 | -5.8 | 0.75 |
| 8_1md80 | 65.5 | 66.3 | 0.8 | 38.1 | -4.0 | 0.96 |
| 8_1md81 | 66.3 | 68.2 | 1.9 | 35.8 | -2.3 | 0.78 |
| 8_1md82 | 68.2 | 74.6 | 6.4 | 39.1 | 3.3 | 1.52 |
| 8_1md83 | 74.6 | 75 | 0.4 | 36.6 | -2.5 | 0.44 |
| 8_1md84 | 75 | 76.9 | 1.9 | 38.7 | 2.1 | 1.33 |
| 8_1md85 | 76.9 | 77.4 | 0.5 | 37.0 | -1.7 | 0.47 |
| 8_1md86 | 77.4 | 79.2 | 1.8 | 37.8 | 0.8 | 1.11 |
| 8_1md87 | 79.2 | 79.4 | 0.2 | 36.3 | -1.5 | 0 |
| 8_1md88 | 79.4 | 79.8 | 0.4 | 38.8 | 2.5 | 0.44 |
| 8_1md89 | 79.8 | 80.1 | 0.3 | 35.7 | -3.1 | 0.54 |
| 8_1md90 | 80.1 | 80.3 | 0.2 | 37.3 | 1.6 | 0 |
| 8_1md91 | 80.3 | 80.7 | 0.4 | 36.1 | -1.2 | 0.62 |
| 8_1md92 | 80.7 | 83.9 | 3.2 | 38.9 | 2.7 | 1.08 |
| 8_1md93 | 83.9 | 84.6 | 0.7 | 42.4 | 3.5 | 0.96 |
| 8_1md94 | 84.6 | 85 | 0.4 | 40.9 | -1.5 | 0.85 |
| 8_1md95 | 85 | 85.7 | 0.7 | 40.6 | -0.3 | 1.09 |
| 8_1md96 | 85.7 | 87.8 | 2.1 | 42.3 | 1.7 | 1.52 |
| 8_1md97 | 87.8 | 89.9 | 2.1 | 38.9 | -3.4 | 1.11 |
| 8_1md98 | 89.9 | 90.4 | 0.5 | 44.4 | 5.5 | 1.81 |
| 8_1md99 | 90.4 | 90.7 | 0.3 | 38.7 | -5.6 | 0.2 |
| 8_1md100 | 90.7 | 93 | 2.3 | 43.4 | 4.7 | 1.69 |
| 8_1md101 | 93 | 93.3 | 0.3 | 36.7 | -6.8 | 1.5 |
| 8_1md102 | 93.3 | 103.7 | 10.4 | 35.1 | -1.6 | 1.19 |
| 8_1md103 | 103.7 | 103.9 | 0.2 | 37.2 | 2.2 | 0 |

| | | | | | | |
|---|---|---|---|---|---|---|
| 8_1md104 | 103.9 | 104.4 | 0.5 | 36.7 | -0.5 | 0.57 |
| 8_1md105 | 104.4 | 104.9 | 0.5 | 39.5 | 2.7 | 0.4 |
| 8_1md106 | 104.9 | 105.5 | 0.6 | 36.8 | -2.6 | 0.13 |
| 8_1md107 | 105.5 | 105.8 | 0.3 | 37.8 | 1.0 | 0.24 |
| 8_1md108 | 105.8 | 106.3 | 0.5 | 36.2 | -1.6 | 0.59 |
| 8_1md109 | 106.3 | 106.5 | 0.2 | 37.4 | 1.2 | 0 |
| 8_1md110 | 106.5 | 107 | 0.5 | 36.3 | -1.1 | 0.55 |
| 8_1md111 | 107 | 107.9 | 0.9 | 39.6 | 3.4 | 1.23 |
| 8_1md112 | 107.9 | 108.5 | 0.6 | 43.3 | 3.6 | 1.85 |
| 8_1md113 | 108.5 | 109.1 | 0.6 | 38.9 | -4.4 | 1.66 |
| 8_1md114 | 109.1 | 109.3 | 0.2 | 35.0 | -3.9 | 0 |
| 8_1md115 | 109.3 | 109.7 | 0.4 | 37.5 | 2.5 | 0.11 |
| 8_1md116 | 109.7 | 111.1 | 1.4 | 36.4 | -1.1 | 0.81 |
| 8_1md117 | 111.1 | 112 | 0.9 | 38.4 | 2.0 | 1.51 |
| 8_1md118 | 112 | 112.2 | 0.2 | 35.8 | -2.7 | 0 |
| 8_1md119 | 112.2 | 114.9 | 2.7 | 37.8 | 2.0 | 0.66 |
| 8_1md120 | 114.9 | 115.2 | 0.3 | 36.4 | -1.3 | 0.44 |
| 8_1md121 | 115.2 | 117 | 1.8 | 39.0 | 2.5 | 1.29 |
| 8_1md122 | 117 | 117.2 | 0.2 | 36.1 | -2.9 | 0 |
| 8_1md123 | 117.2 | 117.9 | 0.7 | 38.5 | 2.5 | 1.77 |
| 8_1md124 | 117.9 | 118.3 | 0.4 | 36.3 | -2.2 | 0.49 |
| 8_1md125 | 118.3 | 118.9 | 0.6 | 38.5 | 2.2 | 1.98 |
| 8_1md126 | 118.9 | 119.1 | 0.2 | 35.9 | -2.6 | 0 |
| 8_1md127 | 119.1 | 121.8 | 2.7 | 38.4 | 2.5 | 1.1 |
| 8_1md128 | 121.8 | 122 | 0.2 | 35.5 | -2.9 | 0 |
| 8_1md129 | 122 | 122.2 | 0.2 | 38.6 | 3.1 | 0 |
| 8_1md130 | 122.2 | 125.1 | 2.9 | 35.5 | -3.2 | 0.86 |
| 8_1md131 | 125.1 | 125.3 | 0.2 | 37.2 | 1.7 | 0 |
| 8_1md132 | 125.3 | 125.9 | 0.6 | 36.3 | -0.9 | 0.44 |
| 8_1md133 | 125.9 | 126.1 | 0.2 | 37.9 | 1.7 | 0 |
| 8_1md134 | 126.1 | 126.8 | 0.7 | 35.8 | -2.2 | 1.28 |
| 8_1md135 | 126.8 | 127.9 | 1.1 | 37.8 | 2.0 | 1.31 |
| 8_1md136 | 127.9 | 128.7 | 0.8 | 36.2 | -1.6 | 0.54 |
| 8_1md137 | 128.7 | 129.6 | 0.9 | 37.9 | 1.7 | 1.19 |
| 8_1md138 | 129.6 | 129.8 | 0.2 | 35.1 | -2.8 | 0 |
| 8_1md139 | 129.8 | 130.5 | 0.7 | 38.1 | 3.0 | 1.07 |
| 8_1md140 | 130.5 | 134.4 | 3.9 | 35.1 | -3.0 | 0.86 |
| 8_1md141 | 134.4 | 135.2 | 0.8 | 38.0 | 2.8 | 1.09 |
| 8_1md142 | 135.2 | 135.4 | 0.2 | 35.0 | -3.0 | 0 |
| 8_1md143 | 135.4 | 135.8 | 0.4 | 38.4 | 3.5 | 0.85 |
| 8_1md144 | 135.8 | 136.2 | 0.4 | 35.6 | -2.9 | 1.66 |
| 8_1md145 | 136.2 | 136.4 | 0.2 | 37.3 | 1.7 | 0 |
| 8_1md146 | 136.4 | 136.7 | 0.3 | 36.9 | -0.4 | 0.15 |
| 8_1md147 | 136.7 | 137.7 | 1 | 38.1 | 1.2 | 0.91 |
| 8_1md148 | 137.7 | 138.3 | 0.6 | 36.7 | -1.4 | 0.9 |
| 8_1md149 | 138.3 | 139.7 | 1.4 | 37.6 | 0.9 | 0.86 |
| 8_1md150 | 139.7 | 152.5 | 12.8 | 34.6 | -3.0 | 0.99 |
| 8_1md151 | 152.5 | 152.7 | 0.2 | 37.7 | 3.1 | 0 |
| 8_1md152 | 152.7 | 156 | 3.3 | 35.9 | -1.8 | 0.99 |
| 8_1md153 | 156 | 156.3 | 0.3 | 38.6 | 2.8 | 0.51 |
| 8_1md154 | 156.3 | 156.8 | 0.5 | 35.1 | -3.5 | 1.18 |
| 8_1md155 | 156.8 | 157.4 | 0.6 | 38.7 | 3.5 | 1.2 |
| | | | | | | |
| 8_2md1 | 0 | 0.3 | 0.3 | 39.0 | | 1.75 |
| 8_2md2 | 0.3 | 0.5 | 0.2 | 36.4 | -2.6 | 0 |
| 8_2md3 | 0.5 | 1.3 | 0.8 | 37.5 | 1.0 | 0.57 |

| | | | | | | |
|---|---|---|---|---|---|---|
| 8_2md4 | 1.3 | 2 | 0.7 | 35.9 | -1.6 | 1.23 |
| 8_2md5 | 2 | 2.6 | 0.6 | 37.4 | 1.5 | 0.79 |
| 8_2md6 | 2.6 | 8.7 | 6.1 | 35.0 | -2.4 | 0.9 |
| 8_2md7 | 8.7 | 9 | 0.3 | 37.7 | 2.7 | 0.25 |
| 8_2md8 | 9 | 29.3 | 20.3 | 35.5 | -2.2 | 1.03 |
| 8_2md9 | 29.3 | 29.6 | 0.3 | 37.6 | 2.1 | 0.29 |
| 8_2md10 | 29.6 | 29.8 | 0.2 | 36.4 | -1.2 | 0 |
| 8_2md11 | 29.8 | 30 | 0.2 | 39.1 | 2.7 | 0 |
| 8_2md12 | 30 | 30.4 | 0.4 | 37.0 | -2.2 | 0.65 |
| 8_2md13 | 30.4 | 30.6 | 0.2 | 41.7 | 4.7 | 0 |
| 8_2md14 | 30.6 | 30.8 | 0.2 | 39.9 | -1.8 | 0 |
| 8_2md15 | 30.8 | 31 | 0.2 | 42.4 | 2.5 | 0 |
| 8_2md16 | 31 | 31.2 | 0.2 | 38.6 | -3.8 | 0 |
| 8_2md17 | 31.2 | 31.5 | 0.3 | 45.4 | 6.8 | 3.54 |
| 8_2md18 | 31.5 | 32.2 | 0.7 | 38.8 | -6.6 | 2.2 |
| 8_2md19 | 32.2 | 32.4 | 0.2 | 41.9 | 3.1 | 0 |
| 8_2md20 | 32.4 | 32.9 | 0.5 | 37.6 | -4.3 | 1.2 |
| 8_2md21 | 32.9 | 33.1 | 0.2 | 42.9 | 5.2 | 0 |
| 8_2md22 | 33.1 | 33.7 | 0.6 | 38.6 | -4.2 | 0.81 |
| 8_2md23 | 33.7 | 33.9 | 0.2 | 35.0 | -3.7 | 0 |
| 8_2md24 | 33.9 | 35.9 | 2 | 39.5 | 4.6 | 0.95 |
| 8_2md25 | 35.9 | 36.3 | 0.4 | 35.1 | -4.4 | 0.89 |
| 8_2md26 | 36.3 | 36.6 | 0.3 | 37.7 | 2.6 | 0.66 |
| 8_2md27 | 36.6 | 37.1 | 0.5 | 36.2 | -1.5 | 0.63 |
| 8_2md28 | 37.1 | 38.6 | 1.5 | 38.4 | 2.1 | 1.26 |
| 8_2md29 | 38.6 | 38.8 | 0.2 | 41.5 | 3.1 | 0 |
| 8_2md30 | 38.8 | 39.2 | 0.4 | 35.6 | -5.9 | 0.61 |
| 8_2md31 | 39.2 | 39.5 | 0.3 | 40.0 | 4.4 | 0.67 |
| 8_2md32 | 39.5 | 42.1 | 2.6 | 35.6 | -4.4 | 0.71 |
| 8_2md33 | 42.1 | 43 | 0.9 | 37.8 | 2.2 | 1.11 |
| 8_2md34 | 43 | 43.2 | 0.2 | 36.0 | -1.8 | 0 |
| 8_2md35 | 43.2 | 43.5 | 0.3 | 38.2 | 2.2 | 0.42 |
| 8_2md36 | 43.5 | 43.7 | 0.2 | 36.5 | -1.7 | 0 |
| 8_2md37 | 43.7 | 43.9 | 0.2 | 37.8 | 1.3 | 0 |
| 8_2md38 | 43.9 | 44.2 | 0.3 | 36.2 | -1.6 | 0.29 |
| 8_2md39 | 44.2 | 45.7 | 1.5 | 39.0 | 2.8 | 1.52 |
| 8_2md40 | 45.7 | 46.1 | 0.4 | 36.6 | -2.4 | 0.26 |
| 8_2md41 | 46.1 | 46.9 | 0.8 | 38.1 | 1.5 | 0.76 |
| 8_2md42 | 46.9 | 47.2 | 0.3 | 36.4 | -1.7 | 0.51 |
| 8_2md43 | 47.2 | 49.3 | 2.1 | 38.5 | 2.1 | 1.22 |
| 8_2md44 | 49.3 | 49.9 | 0.6 | 36.1 | -2.4 | 0.59 |
| 8_2md45 | 49.9 | 50.2 | 0.3 | 38.0 | 1.9 | 1.27 |
| 8_2md46 | 50.2 | 55.1 | 4.9 | 35.0 | -3.0 | 1.02 |
| 8_2md47 | 55.1 | 55.5 | 0.4 | 37.9 | 2.9 | 0.56 |
| 8_2md48 | 55.5 | 55.7 | 0.2 | 35.7 | -2.2 | 0 |
| 8_2md49 | 55.7 | 56.1 | 0.4 | 38.3 | 2.5 | 0.45 |
| 8_2md50 | 56.1 | 56.9 | 0.8 | 42.3 | 4.0 | 1.53 |
| 8_2md51 | 56.9 | 58.1 | 1.2 | 39.2 | -3.1 | 1.33 |
| 8_2md52 | 58.1 | 58.4 | 0.3 | 47.9 | 8.7 | 1.4 |
| 8_2md53 | 58.4 | 58.8 | 0.4 | 42.3 | -5.6 | 2.16 |
| 8_2md54 | 58.8 | 59.7 | 0.9 | 38.3 | -4.0 | 1.12 |
| 8_2md55 | 59.7 | 60.2 | 0.5 | 35.0 | -3.3 | 1.25 |
| 8_2md56 | 60.2 | 60.4 | 0.2 | 38.5 | 3.5 | 0 |
| 8_2md57 | 60.4 | 61 | 0.6 | 36.8 | -1.7 | 0.61 |
| 8_2md58 | 61 | 61.2 | 0.2 | 37.5 | 0.7 | 0 |
| 8_2md59 | 61.2 | 63 | 1.8 | 35.7 | -1.8 | 0.65 |

| | | | | | | |
|---|---|---|---|---|---|---|
| 8_2md60 | 63 | 65.4 | 2.4 | 38.0 | 2.3 | 1.52 |
| 8_2md61 | 65.4 | 65.9 | 0.5 | 36.3 | -1.6 | 0.72 |
| 8_2md62 | 65.9 | 66.3 | 0.4 | 38.2 | 1.8 | 0.81 |
| 8_2md63 | 66.3 | 66.5 | 0.2 | 34.9 | -3.2 | 0 |
| 8_2md64 | 66.5 | 67.1 | 0.6 | 38.6 | 3.7 | 1.99 |
| 8_2md65 | 67.1 | 68.5 | 1.4 | 36.3 | -2.4 | 0.53 |
| 8_2md66 | 68.5 | 68.7 | 0.2 | 37.7 | 1.4 | 0 |
| 8_2md67 | 68.7 | 70.3 | 1.6 | 35.7 | -2.0 | 0.84 |
| 8_2md68 | 70.3 | 70.5 | 0.2 | 37.5 | 1.8 | 0 |
| 8_2md69 | 70.5 | 70.9 | 0.4 | 36.1 | -1.4 | 0.36 |
| 8_2md70 | 70.9 | 71.1 | 0.2 | 37.6 | 1.5 | 0 |
| 8_2md71 | 71.1 | 71.3 | 0.2 | 35.4 | -2.3 | 0 |
| 8_2md72 | 71.3 | 71.7 | 0.4 | 37.1 | 1.8 | 0.38 |
| 8_2md73 | 71.7 | 71.9 | 0.2 | 35.8 | -1.4 | 0 |
| 8_2md74 | 71.9 | 72.2 | 0.3 | 37.9 | 2.1 | 0.24 |
| 8_2md75 | 72.2 | 74.9 | 2.7 | 35.2 | -2.7 | 0.81 |
| 8_2md76 | 74.9 | 75.1 | 0.2 | 37.4 | 2.1 | 0 |
| 8_2md77 | 75.1 | 75.3 | 0.2 | 36.4 | -1.0 | 0 |
| 8_2md78 | 75.3 | 75.5 | 0.2 | 37.1 | 0.7 | 0 |
| 8_2md79 | 75.5 | 79.5 | 4 | 35.3 | -1.8 | 0.91 |
| 8_2md80 | 79.5 | 80.2 | 0.7 | 37.6 | 2.3 | 0.98 |
| 8_2md81 | 80.2 | 80.4 | 0.2 | 35.8 | -1.8 | 0 |
| 8_2md82 | 80.4 | 80.6 | 0.2 | 38.6 | 2.8 | 0 |
| 8_2md83 | 80.6 | 81.3 | 0.7 | 34.9 | -3.7 | 1.27 |
| 8_2md84 | 81.3 | 82.6 | 1.3 | 37.5 | 2.5 | 0.4 |
| 8_2md85 | 82.6 | 83.5 | 0.9 | 37.0 | -0.5 | 0.87 |
| 8_2md86 | 83.5 | 83.9 | 0.4 | 39.3 | 2.3 | 2.25 |
| 8_2md87 | 83.9 | 84.1 | 0.2 | 36.7 | -2.6 | 0 |
| 8_2md88 | 84.1 | 84.4 | 0.3 | 38.4 | 1.7 | 1.54 |
| 8_2md89 | 84.4 | 84.6 | 0.2 | 36.5 | -1.9 | 0 |
| 8_2md90 | 84.6 | 87.3 | 2.7 | 38.7 | 2.3 | 1.22 |
| 8_2md91 | 87.3 | 87.8 | 0.5 | 35.4 | -3.3 | 0.77 |
| 8_2md92 | 87.8 | 88.3 | 0.5 | 37.5 | 2.1 | 0.85 |
| 8_2md93 | 88.3 | 88.8 | 0.5 | 36.9 | -0.6 | 1.4 |
| 8_2md94 | 88.8 | 89.2 | 0.4 | 38.0 | 1.1 | 0.71 |
| 8_2md95 | 89.2 | 90.3 | 1.1 | 36.0 | -2.0 | 0.52 |
| 8_2md96 | 90.3 | 90.5 | 0.2 | 37.7 | 1.8 | 0 |
| 8_2md97 | 90.5 | 90.7 | 0.2 | 36.4 | -1.3 | 0 |
| 8_2md98 | 90.7 | 92.9 | 2.2 | 37.8 | 1.4 | 1.07 |
| 8_2md99 | 92.9 | 93.1 | 0.2 | 35.6 | -2.3 | 0 |
| 8_2md100 | 93.1 | 94.8 | 1.7 | 37.5 | 1.9 | 1.69 |
| 8_2md101 | 94.8 | 99.1 | 4.3 | 35.6 | -1.8 | 0.94 |
| 8_2md102 | 99.1 | 100.2 | 1.1 | 37.8 | 2.1 | 0.32 |
| 8_2md103 | 100.2 | 101.4 | 1.2 | 36.1 | -1.7 | 1.01 |
| 8_2md104 | 101.4 | 102.3 | 0.9 | 37.9 | 1.8 | 0.75 |
| 8_2md105 | 102.3 | 103 | 0.7 | 37.0 | -1.0 | 1.14 |
| 8_2md106 | 103 | 103.5 | 0.5 | 37.8 | 0.8 | 1.36 |
| 8_2md107 | 103.5 | 105.1 | 1.6 | 35.6 | -2.1 | 0.85 |
| 8_2md108 | 105.1 | 105.8 | 0.7 | 38.3 | 2.7 | 0.57 |
| 8_2md109 | 105.8 | 106 | 0.2 | 35.5 | -2.8 | 0 |
| 8_2md110 | 106 | 106.9 | 0.9 | 38.1 | 2.6 | 0.76 |
| 8_2md111 | 106.9 | 107.1 | 0.2 | 36.5 | -1.6 | 0 |
| 8_2md112 | 107.1 | 107.9 | 0.8 | 38.6 | 2.1 | 1.36 |
| 8_2md113 | 107.9 | 108.1 | 0.2 | 36.7 | -1.9 | 0 |
| 8_2md114 | 108.1 | 109.5 | 1.4 | 39.5 | 2.8 | 1.28 |
| 8_2md115 | 109.5 | 110 | 0.5 | 36.3 | -3.2 | 0.59 |

| | | | | | | |
|---|---|---|---|---|---|---|
| 8_2md116 | 110 | 110.2 | 0.2 | 38.6 | 2.3 | 0 |
| 8_2md117 | 110.2 | 110.4 | 0.2 | 36.8 | -1.7 | 0 |
| 8_2md118 | 110.4 | 111 | 0.6 | 38.0 | 1.2 | 0.5 |
| 8_2md119 | 111 | 111.3 | 0.3 | 36.2 | -1.8 | 0.79 |
| 8_2md120 | 111.3 | 118 | 6.7 | 39.2 | 3.1 | 1.65 |
| 8_2md121 | 118 | 118.3 | 0.3 | 43.5 | 4.2 | 0.55 |
| 8_2md122 | 118.3 | 118.6 | 0.3 | 37.8 | -5.7 | 1.67 |
| 8_2md123 | 118.6 | 119.2 | 0.6 | 42.5 | 4.7 | 1.83 |
| 8_2md124 | 119.2 | 119.7 | 0.5 | 39.4 | -3.0 | 1.03 |
| 8_2md125 | 119.7 | 119.9 | 0.2 | 43.6 | 4.1 | 0 |
| 8_2md126 | 119.9 | 120.4 | 0.5 | 36.1 | -7.5 | 0.62 |
| 8_2md127 | 120.4 | 121 | 0.6 | 38.2 | 2.1 | 0.95 |
| 8_2md128 | 121 | 121.2 | 0.2 | 36.4 | -1.7 | 0 |
| 8_2md129 | 121.2 | 121.6 | 0.4 | 38.1 | 1.7 | 0.54 |
| 8_2md130 | 121.6 | 122.6 | 1 | 36.6 | -1.5 | 0.64 |
| 8_2md131 | 122.6 | 123.6 | 1 | 38.6 | 2.0 | 1.53 |
| 8_2md132 | 123.6 | 125.6 | 2 | 36.2 | -2.5 | 0.89 |
| 8_2md133 | 125.6 | 126.1 | 0.5 | 38.6 | 2.4 | 0.86 |
| 8_2md134 | 126.1 | 127.1 | 1 | 42.9 | 4.3 | 2.67 |
| 8_2md135 | 127.1 | 128.3 | 1.2 | 39.2 | -3.6 | 0.87 |
| 8_2md136 | 128.3 | 128.7 | 0.4 | 40.5 | 1.3 | 2.66 |
| 8_2md137 | 128.7 | 131.2 | 2.5 | 38.9 | -1.6 | 1.12 |
| 8_2md138 | 131.2 | 131.5 | 0.3 | 35.4 | -3.6 | 0.77 |
| 8_2md139 | 131.5 | 131.9 | 0.4 | 38.3 | 2.9 | 0.37 |
| 8_2md140 | 131.9 | 132.1 | 0.2 | 36.6 | -1.7 | 0 |
| 8_2md141 | 132.1 | 134.3 | 2.2 | 39.0 | 2.4 | 1.57 |
| 8_2md142 | 134.3 | 134.5 | 0.2 | 41.9 | 2.9 | 0 |
| 8_2md143 | 134.5 | 134.8 | 0.3 | 47.5 | 5.6 | 0.89 |
| 8_2md144 | 134.8 | 135.4 | 0.6 | 44.2 | -3.3 | 2.61 |
| 8_2md145 | 135.4 | 135.6 | 0.2 | 39.4 | -4.8 | 0 |
| 8_2md146 | 135.6 | 135.8 | 0.2 | 47.3 | 7.9 | 0 |
| 8_2md147 | 135.8 | 136.4 | 0.6 | 42.1 | -5.2 | 2.13 |
| 8_2md148 | 136.4 | 136.6 | 0.2 | 48.9 | 6.7 | 0 |
| 8_2md149 | 136.6 | 136.8 | 0.2 | 44.5 | -4.4 | 0 |
| 8_2md150 | 136.8 | 137.4 | 0.6 | 46.7 | 2.2 | 2.38 |
| 8_2md151 | 137.4 | 139 | 1.6 | 43.9 | -2.8 | 1.01 |
| 8_2md152 | 139 | 139.3 | 0.3 | 46.8 | 2.9 | 2.37 |
| 8_2md153 | 139.3 | 139.5 | 0.2 | 40.4 | -6.4 | 0 |
| 8_2md154 | 139.5 | 139.7 | 0.2 | 42.2 | 1.8 | 0 |
| 8_2md155 | 139.7 | 139.9 | 0.2 | 40.9 | -1.3 | 0 |
| 8_2md156 | 139.9 | 140.4 | 0.5 | 47.1 | 6.2 | 1.26 |
| 8_2md157 | 140.4 | 140.7 | 0.3 | 44.4 | -2.7 | 0.64 |
| 8_2md158 | 140.7 | 141.1 | 0.4 | 48.3 | 3.8 | 1.67 |
| 8_2md159 | 141.1 | 142.3 | 1.2 | 44.2 | -4.1 | 3.05 |
| 8_2md160 | 142.3 | 142.7 | 0.4 | 39.7 | -4.5 | 0.71 |
| 8_2md161 | 142.7 | 143.9 | 1.2 | 43.9 | 4.1 | 2.61 |
| 8_2md162 | 143.9 | 144.2 | 0.3 | 48.5 | 4.7 | 1.4 |
| 8_2md163 | 144.2 | 145.2 | 1 | 41.5 | -7.1 | 1.7 |
| 8_2md164 | 145.2 | 145.7 | 0.5 | 39.9 | -1.5 | 1.61 |
| 8_2md165 | 145.7 | 146.2 | 0.5 | 36.5 | -3.5 | 0.76 |
| 8_2md166 | 146.2 | 147.2 | 1 | 38.3 | 1.9 | 1.91 |
| 8_2md167 | 147.2 | 149.7 | 2.5 | 43.7 | 5.4 | 2.13 |
| 8_2md168 | 149.7 | 150.7 | 1 | 38.9 | -4.8 | 1.32 |
| 8_2md169 | 150.7 | 150.9 | 0.2 | 42.6 | 3.7 | 0 |
| 8_2md170 | 150.9 | 152.5 | 1.6 | 38.8 | -3.8 | 1.28 |
| 8_2md171 | 152.5 | 153.1 | 0.6 | 48.2 | 9.4 | 4.12 |

| | | | | | | |
|---|---|---|---|---|---|---|
| 8_2md172 | 153.1 | 153.6 | 0.5 | 42.7 | -5.5 | 1.15 |
| 8_2md173 | 153.6 | 154.1 | 0.5 | 49.5 | 6.8 | 2.69 |
| 8_2md174 | 154.1 | 154.4 | 0.3 | 42.1 | -7.4 | 1.58 |
| 8_2md175 | 154.4 | 154.7 | 0.3 | 38.4 | -3.7 | 0.86 |
| 8_2md176 | 154.7 | 155.2 | 0.5 | 44.9 | 6.6 | 4.19 |
| | | | | | | |
| Xmd1 | 0 | 3 | 3 | 36.7 | | 0 |
| Xmd2 | 3 | 4.1 | 1.1 | 48.0 | 11.3 | 2.89 |
| Xmd3 | 4.1 | 4.3 | 0.2 | 39.6 | -8.4 | 0 |
| Xmd4 | 4.3 | 5.2 | 0.9 | 44.7 | 5.2 | 3.38 |
| Xmd5 | 5.2 | 5.9 | 0.7 | 44.0 | -0.8 | 2.01 |
| Xmd6 | 5.9 | 6.2 | 0.3 | 40.5 | -3.5 | 0.43 |
| Xmd7 | 6.2 | 8.1 | 1.9 | 43.5 | 3.1 | 1.83 |
| Xmd8 | 8.1 | 8.3 | 0.2 | 47.6 | 4.1 | 0 |
| Xmd9 | 8.3 | 9.2 | 0.9 | 39.7 | -7.9 | 1.11 |
| Xmd10 | 9.2 | 9.7 | 0.5 | 41.9 | 2.2 | 0.93 |
| Xmd11 | 9.7 | 9.7 | 0 | 0.0 | -41.9 | 0 |
| Xmd12 | 9.8 | 10.3 | 0.5 | 47.0 | 47.0 | 2.03 |
| Xmd13 | 10.3 | 10.9 | 0.6 | 41.6 | -5.4 | 1.15 |
| Xmd14 | 10.9 | 11.9 | 1 | 48.1 | 6.5 | 2.07 |
| Xmd15 | 11.9 | 12.3 | 0.4 | 40.2 | -7.9 | 1.03 |
| Xmd16 | 12.3 | 12.7 | 0.4 | 42.7 | 2.6 | 2.29 |
| Xmd17 | 12.7 | 13.2 | 0.5 | 39.2 | -3.6 | 0.6 |
| Xmd18 | 13.2 | 13.8 | 0.6 | 44.2 | 5.1 | 2.64 |
| Xmd19 | 13.8 | 14.3 | 0.5 | 38.5 | -5.7 | 1.5 |
| Xmd20 | 14.3 | 15.1 | 0.8 | 43.5 | 4.9 | 2.58 |
| Xmd21 | 15.1 | 16.8 | 1.7 | 38.8 | -4.7 | 1.17 |
| Xmd22 | 16.8 | 17.3 | 0.5 | 43.1 | 4.3 | 1.19 |
| Xmd23 | 17.3 | 18.6 | 1.3 | 38.2 | -4.9 | 1.74 |
| Xmd24 | 18.6 | 19.7 | 1.1 | 35.9 | -2.3 | 0.76 |
| Xmd25 | 19.7 | 26.1 | 6.4 | 39.3 | 3.4 | 1.65 |
| Xmd26 | 26.1 | 26.4 | 0.3 | 44.4 | 5.1 | 2.01 |
| Xmd27 | 26.4 | 26.6 | 0.2 | 39.3 | -5.0 | 0 |
| Xmd28 | 26.6 | 27.1 | 0.5 | 48.2 | 8.8 | 1.67 |
| Xmd29 | 27.1 | 27.6 | 0.5 | 40.5 | -7.6 | 1.69 |
| Xmd30 | 27.6 | 27.8 | 0.2 | 42.4 | 1.9 | 0 |
| Xmd31 | 27.8 | 28 | 0.2 | 40.8 | -1.6 | 0 |
| Xmd32 | 28 | 28.6 | 0.6 | 43.9 | 3.1 | 1.99 |
| Xmd33 | 28.6 | 30.4 | 1.8 | 39.2 | -4.7 | 0.97 |
| Xmd34 | 30.4 | 31.1 | 0.7 | 41.7 | 2.5 | 1.2 |
| Xmd35 | 31.1 | 31.6 | 0.5 | 48.3 | 6.6 | 2.05 |
| Xmd36 | 31.6 | 31.9 | 0.3 | 41.4 | -6.9 | 0.85 |
| Xmd37 | 31.9 | 32.2 | 0.3 | 40.1 | -1.3 | 0.45 |
| Xmd38 | 32.2 | 32.4 | 0.2 | 41.9 | 1.8 | 0 |
| Xmd39 | 32.4 | 32.7 | 0.3 | 38.5 | -3.4 | 0.7 |
| Xmd40 | 32.7 | 33 | 0.3 | 41.2 | 2.8 | 0.16 |
| Xmd41 | 33 | 33.2 | 0.2 | 40.5 | -0.7 | 0 |
| Xmd42 | 33.2 | 33.4 | 0.2 | 42.3 | 1.8 | 0 |
| Xmd43 | 33.4 | 33.7 | 0.3 | 39.3 | -3.0 | 0.34 |
| Xmd44 | 33.7 | 34.4 | 0.7 | 42.8 | 3.5 | 1.28 |
| Xmd45 | 34.4 | 35.1 | 0.7 | 39.9 | -2.9 | 1.11 |
| Xmd46 | 35.1 | 35.3 | 0.2 | 42.7 | 2.8 | 0 |
| Xmd47 | 35.3 | 35.6 | 0.3 | 38.7 | -4.0 | 0.71 |
| Xmd48 | 35.6 | 36 | 0.4 | 43.6 | 4.9 | 2.27 |
| Xmd49 | 36 | 39 | 3 | 39.1 | -4.5 | 1.42 |
| Xmd50 | 39 | 39.8 | 0.8 | 42.3 | 3.2 | 1.37 |

| | | | | | | |
|---|---|---|---|---|---|---|
| Xmd51 | 39.8 | 40.2 | 0.4 | 38.9 | -3.5 | 1.16 |
| Xmd52 | 40.2 | 40.6 | 0.4 | 45.3 | 6.4 | 2.62 |
| Xmd53 | 40.6 | 40.8 | 0.2 | 39.7 | -5.5 | 0 |
| Xmd54 | 40.8 | 42.1 | 1.3 | 42.4 | 2.7 | 1.55 |
| Xmd55 | 42.1 | 43 | 0.9 | 40.1 | -2.3 | 1 |
| Xmd56 | 43 | 45.9 | 2.9 | 42.9 | 2.8 | 1.92 |
| Xmd57 | 45.9 | 47.2 | 1.3 | 38.9 | -4.0 | 1.64 |
| Xmd58 | 47.2 | 48 | 0.8 | 35.7 | -3.2 | 1.62 |
| Xmd59 | 48 | 48.2 | 0.2 | 38.7 | 3.0 | 0 |
| Xmd60 | 48.2 | 48.4 | 0.2 | 36.6 | -2.1 | 0 |
| Xmd61 | 48.4 | 49.1 | 0.7 | 38.1 | 1.4 | 0.93 |
| Xmd62 | 49.1 | 49.8 | 0.7 | 35.8 | -2.3 | 0.28 |
| Xmd63 | 49.8 | 52.1 | 2.3 | 39.1 | 3.3 | 2 |
| Xmd64 | 52.1 | 52.3 | 0.2 | 41.8 | 2.7 | 0 |
| Xmd65 | 52.3 | 55.1 | 2.8 | 38.9 | -2.9 | 1.88 |
| Xmd66 | 55.1 | 55.4 | 0.3 | 37.8 | -1.1 | 1.86 |
| Xmd67 | 55.4 | 55.9 | 0.5 | 44.2 | 6.5 | 1.8 |
| Xmd68 | 55.9 | 56.5 | 0.6 | 38.3 | -5.9 | 2.25 |
| Xmd69 | 56.5 | 57.3 | 0.8 | 42.8 | 4.5 | 1.37 |
| Xmd70 | 57.3 | 58.6 | 1.3 | 39.0 | -3.8 | 1.88 |
| Xmd71 | 58.6 | 59.1 | 0.5 | 43.6 | 4.6 | 2.07 |
| Xmd72 | 59.1 | 60 | 0.9 | 38.7 | -4.9 | 1.06 |
| Xmd73 | 60 | 61 | 1 | 37.0 | -1.7 | 0.75 |
| Xmd74 | 61 | 62.1 | 1.1 | 38.1 | 1.1 | 0.8 |
| Xmd75 | 62.1 | 62.9 | 0.8 | 36.3 | -1.8 | 0.28 |
| Xmd76 | 62.9 | 63.1 | 0.2 | 37.7 | 1.3 | 0 |
| Xmd77 | 63.1 | 63.4 | 0.3 | 36.7 | -1.0 | 0.23 |
| Xmd78 | 63.4 | 64 | 0.6 | 37.5 | 0.9 | 0.6 |
| Xmd79 | 64 | 64.2 | 0.2 | 36.4 | -1.1 | 0 |
| Xmd80 | 64.2 | 64.8 | 0.6 | 39.1 | 2.7 | 2.15 |
| Xmd81 | 64.8 | 65.1 | 0.3 | 43.5 | 4.3 | 1.92 |
| Xmd82 | 65.1 | 65.3 | 0.2 | 47.6 | 4.2 | 0 |
| Xmd83 | 65.3 | 65.7 | 0.4 | 44.1 | -3.5 | 1.34 |
| Xmd84 | 65.7 | 66.4 | 0.7 | 38.6 | -5.5 | 1.93 |
| Xmd85 | 66.4 | 68.6 | 2.2 | 42.1 | 3.5 | 1.9 |
| Xmd86 | 68.6 | 68.9 | 0.3 | 48.6 | 6.5 | 2.08 |
| Xmd87 | 68.9 | 69.3 | 0.4 | 45.4 | -3.3 | 0.82 |
| Xmd88 | 69.3 | 69.7 | 0.4 | 48.9 | 3.5 | 2.91 |
| Xmd89 | 69.7 | 69.9 | 0.2 | 44.3 | -4.6 | 0 |
| Xmd90 | 69.9 | 70.1 | 0.2 | 51.4 | 7.1 | 0 |
| Xmd91 | 70.1 | 70.5 | 0.4 | 43.4 | -7.9 | 1.81 |
| Xmd92 | 70.5 | 71.7 | 1.2 | 50.4 | 7.0 | 2.79 |
| Xmd93 | 71.7 | 71.9 | 0.2 | 54.9 | 4.5 | 0 |
| Xmd94 | 71.9 | 73.5 | 1.6 | 49.4 | -5.6 | 4.14 |
| Xmd95 | 73.5 | 73.7 | 0.2 | 43.5 | -5.9 | 0 |
| Xmd96 | 73.7 | 74 | 0.3 | 37.7 | -5.8 | 0.34 |
| Xmd97 | 74 | 74.2 | 0.2 | 36.8 | -0.8 | 0 |
| Xmd98 | 74.2 | 75.2 | 1 | 38.5 | 1.6 | 1.65 |
| Xmd99 | 75.2 | 75.5 | 0.3 | 36.0 | -2.5 | 0.69 |
| Xmd100 | 75.5 | 76.2 | 0.7 | 38.3 | 2.3 | 1.3 |
| Xmd101 | 76.2 | 77.1 | 0.9 | 36.1 | -2.1 | 0.51 |
| Xmd102 | 77.1 | 78.1 | 1 | 42.4 | 6.2 | 1.09 |
| Xmd103 | 78.1 | 78.5 | 0.4 | 39.9 | -2.5 | 0.19 |
| Xmd104 | 78.5 | 79.4 | 0.9 | 43.7 | 3.8 | 1.65 |

ores.
