# Supplementary material for: The evolution of isochore patterns in vertebrate genomes
Source: BMC Genomics. 2009 Apr 3;10:146. doi: 10.1186/1471-2164-10-146 (PMC2678159; doi:10.1186/1471-2164-10-146)
Supplement: Additional File 8 — Overview of platypus chromosomes. The color-coded maps show the compositional patterns of platypus chromosomes. [file 1471-2164-10-146-S8.pdf]

Additional Table T5. Coordinates, sizes, GC levels and GC standard deviations (SD) of the platypus isochores. ΔGC indicates the difference in GC between subsequent isochores.

Isochore	Start	End	Length, Mb	GC, %	Δ GC	SD (w=100kb)
1Oa1	0	0.4	0.4	40.3		0.35
1Oa2	0.4	2.8	2.4	43.0	2.8	1.58
1Oa3	2.8	6.8	4	39.5	-3.5	0.96
1Oa4	6.8	7.1	0.3	42.1	2.5	0.19
1Oa5	7.1	8.3	1.2	40.3	-1.8	0.73
1Oa6	8.3	9.7	1.4	43.8	3.5	1.79
1Oa7	9.7	10.7	1	40.6	-3.1	0.4
1Oa8	10.7	11.1	0.4	41.8	1.2	1.32
1Oa9	11.1	15.4	4.3	42.9	1.1	1.82
1Oa10	15.4	15.6	0.2	40.7	-2.2	0
1Oa11	15.6	18.7	3.1	42.0	1.3	1.04
1Oa12	18.7	19	0.3	40.9	-1.1	0.85
1Oa13	19	20.2	1.2	42.8	1.9	1.14
1Oa14	20.2	20.8	0.6	47.8	5.0	1.96
1Oa15	20.8	22.6	1.8	43.2	-4.5	0.59
1Oa16	22.6	24.8	2.2	40.5	-2.7	1.11
1Oa17	24.8	25.8	1	42.8	2.3	0
1Oa18	25.8	27.5	1.7	40.4	-2.4	0.2
1Oa19	27.5	28.2	0.7	42.3	1.9	1.39
1Oa20	28.2	28.4	0.2	40.5	-1.8	0.37
1Oa21	28.4	31.5	3.1	42.7	2.2	1.58
1Oa22	31.5	31.8	0.3	40.6	-2.1	0
1Oa23	31.8	33	1.2	43.3	2.6	1.86
1Oa24	33	33.4	0.4	47.8	4.5	1.56
1Oa25	33.4	33.6	0.2	42.1	-5.7	0.92
1Oa26	33.6	34.2	0.6	46.7	4.6	1.2
1Oa27	34.2	37.6	3.4	44.3	-2.3	1.52
1Oa28	37.6	38.4	0.8	48.6	4.3	2.58
1Oa29	38.4	39.6	1.2	40.3	-8.3	1.24
1Oa30	39.6	42.2	2.6	42.3	1.9	1.19
1Oa31	42.2	42.4	0.2	46.7	4.4	0
1Oa32	42.4	43.2	0.8	46.0	-0.7	3
1Oa33	43.2	44	0.8	48.3	2.3	1.52
1Oa34	44	44.7	0.7	45.3	-3.0	1.08
1Oa35	44.7	45.2	0.5	46.5	1.2	1.09
1Oa36	45.2	47.2	2	43.0	-3.5	1.64
1Oa37	47.2	47.6	0.4	39.6	-3.4	1.65
2Oa1	0	0.4	0.4	46.6		0.64
2Oa2	0.4	1.4	1	42.9	-3.7	0.93
2Oa3	1.4	2.3	0.9	40.8	-2.1	0.64
2Oa4	2.3	3.1	0.8	42.2	1.4	0.76
2Oa5	3.1	3.9	0.8	39.9	-2.3	0.92
2Oa6	3.9	4.2	0.3	41.5	1.6	0.16
2Oa7	4.2	4.4	0.2	39.6	-1.9	0
2Oa8	4.4	4.9	0.5	41.3	1.6	0.59

2Oa9	4.9	5.7	0.8	47.5	6.2	2.49
2Oa10	5.7	10.6	4.9	42.7	-4.8	1.39
2Oa11	10.6	10.8	0.2	47.6	4.9	0
2Oa12	10.8	11.3	0.5	44.8	-2.9	0.42
2Oa13	11.3	11.8	0.5	50.5	5.7	2.04
2Oa14	11.8	12	0.2	44.4	-6.0	0
2Oa15	12	12.6	0.6	47.7	3.3	2.14
2Oa16	12.6	13.8	1.2	42.3	-5.4	1.22
2Oa17	13.8	14	0.2	47.6	5.3	0
2Oa18	14	14.2	0.2	45.3	-2.3	0
2Oa19	14.2	14.4	0.2	47.6	2.3	0
2Oa20	14.4	18.9	4.5	44.2	-3.4	1.34
2Oa21	18.9	19.2	0.3	47.5	3.3	2.21
2Oa22	19.2	21.7	2.5	43.7	-3.8	1.28
2Oa23	21.7	21.9	0.2	49.9	6.1	0
2Oa24	21.9	22.7	0.8	45.1	-4.8	1.12
2Oa25	22.7	23	0.3	47.7	2.7	1.24
2Oa26	23	23.2	0.2	45.4	-2.3	0
2Oa27	23.2	23.4	0.2	47.8	2.4	0
2Oa28	23.4	23.8	0.4	44.1	-3.6	1.05
2Oa29	23.8	24.1	0.3	46.5	2.4	0.47
2Oa30	24.1	27.3	3.2	42.8	-3.7	1.42
2Oa31	27.3	28.6	1.3	40.0	-2.7	0.85
2Oa32	28.6	29.1	0.5	43.3	3.2	0.68
2Oa33	29.1	29.3	0.2	50.0	6.7	0
2Oa34	29.3	30.4	1.1	45.2	-4.8	1.36
2Oa35	30.4	30.9	0.5	47.4	2.3	2.13
2Oa36	30.9	31.8	0.9	44.2	-3.3	1
2Oa37	31.8	32.2	0.4	46.3	2.1	0.68
2Oa38	32.2	34.9	2.7	42.7	-3.5	0.92
2Oa39	34.9	36.3	1.4	40.8	-2.0	0.75
2Oa40	36.3	37.4	1.1	41.8	1.1	0.81
2Oa41	37.4	38.9	1.5	40.5	-1.3	0.77
2Oa42	38.9	42.4	3.5	43.5	3.0	1.33
2Oa43	42.4	42.7	0.3	48.7	5.3	2.2
2Oa44	42.7	46.3	3.6	43.1	-5.6	1.38
2Oa45	46.3	46.5	0.2	40.7	-2.4	0
2Oa46	46.5	48.2	1.7	41.6	0.9	0.82
2Oa47	48.2	49.4	1.2	48.4	6.9	2.34
2Oa48	49.4	49.8	0.4	44.9	-3.5	1.09
2Oa49	49.8	50.5	0.7	50.2	5.3	3.05
2Oa50	50.5	51.1	0.6	44.9	-5.3	1.3
2Oa51	51.1	51.3	0.2	47.6	2.7	0
2Oa52	51.3	53	1.7	42.6	-5.0	1.62
2Oa53	53	53.5	0.5	48.3	5.7	1.5
2Oa54	53.5	54.3	0.8	44.0	-4.3	1.04
2Oa55	54.3	54.8	0.5	48.9	4.9	2.52
3Oa1	0	3.8	3.8	44.1		1.41
3Oa2	3.8	4.2	0.4	46.9	2.8	0.56
3Oa3	4.2	4.6	0.4	44.7	-2.2	0.79
3Oa4	4.6	6.1	1.5	43.6	-1.1	1.96

3Oa5	6.1	14.9	8.8	41.6	-2.1	1.62
3Oa6	14.9	15.1	0.2	41.6	0.0	0
3Oa7	15.1	15.5	0.4	39.5	-2.1	0.54
3Oa8	15.5	15.8	0.3	47.3	7.8	0.73
3Oa9	15.8	19.1	3.3	44.3	-2.9	1.69
3Oa10	19.1	19.5	0.4	47.4	3.1	0.65
3Oa11	19.5	20.6	1.1	42.4	-5.0	1.16
3Oa12	20.6	20.8	0.2	40.3	-2.1	0
3Oa13	20.8	23.7	2.9	42.9	2.7	1.08
3Oa14	23.7	24.3	0.6	40.2	-2.8	1.73
3Oa15	24.3	24.6	0.3	41.6	1.5	0.14
3Oa16	24.6	27.9	3.3	41.0	-0.7	0.7
3Oa17	27.9	29.8	1.9	43.0	2.0	1.58
3Oa18	29.8	32	2.2	39.0	-4.0	0.58
3Oa19	32	33.6	1.6	44.8	5.8	1.87
3Oa20	33.6	34.9	1.3	40.1	-4.7	1.01
3Oa21	34.9	35.3	0.4	43.4	3.3	1.68
3Oa22	35.3	35.6	0.3	40.1	-3.3	0.79
3Oa23	35.6	36.5	0.9	42.4	2.3	0.91
3Oa24	36.5	36.7	0.2	40.0	-2.4	0
3Oa25	36.7	37.7	1	42.7	2.7	1.18
3Oa26	37.7	38.1	0.4	40.6	-2.1	0.4
3Oa27	38.1	38.5	0.4	41.7	1.0	0.55
3Oa28	38.5	39.5	1	40.5	-1.2	0.47
3Oa29	39.5	42.9	3.4	43.3	2.8	1.63
3Oa30	42.9	44.3	1.4	47.0	3.7	1.16
3Oa31	44.3	46.6	2.3	43.6	-3.4	1.44
3Oa32	46.6	47.7	1.1	39.9	-3.7	0.56
3Oa33	47.7	48.1	0.4	42.2	2.3	0.71
3Oa34	48.1	48.5	0.4	40.0	-2.2	0.43
3Oa35	48.5	49.3	0.8	42.1	2.0	0.74
3Oa36	49.3	49.5	0.2	47.4	5.4	0
3Oa37	49.5	49.7	0.2	43.5	-4.0	0
3Oa38	49.7	50.2	0.5	47.3	3.8	2.02
3Oa39	50.2	50.7	0.5	40.4	-6.8	0.23
3Oa40	50.7	51.7	1	41.7	1.3	0.75
3Oa41	51.7	53.9	2.2	39.5	-2.2	0.84
3Oa42	53.9	54.1	0.2	41.4	1.9	0
3Oa43	54.1	54.7	0.6	40.3	-1.1	0.8
3Oa44	54.7	55.2	0.5	44.1	3.8	2.43
3Oa45	55.2	55.8	0.6	40.7	-3.4	0.68
3Oa46	55.8	57.5	1.7	43.0	2.3	1.86
3Oa47	57.5	58.2	0.7	40.2	-2.9	0.57
3Oa48	58.2	59.6	1.4	43.0	2.8	1.62
4Oa1	0	2.2	2.2	44.8		1.97
4Oa2	2.2	2.4	0.2	46.3	1.6	0
4Oa3	2.4	4.4	2	42.7	-3.6	1.17
4Oa4	4.4	5	0.6	40.8	-1.9	1.2
4Oa5	5	9.1	4.1	43.8	3.0	1.9
4Oa6	9.1	9.4	0.3	40.6	-3.3	0.26
4Oa7	9.4	11.1	1.7	43.3	2.7	1.69

4Oa8	11.1	11.6	0.5	47.9	4.6	1.54
4Oa9	11.6	12.1	0.5	44.7	-3.2	2.02
4Oa10	12.1	12.4	0.3	47.8	3.1	0.87
4Oa11	12.4	13.9	1.5	44.9	-2.8	1.4
4Oa12	13.9	15.8	1.9	42.5	-2.4	2
4Oa13	15.8	16	0.2	40.7	-1.8	0
4Oa14	16	16.2	0.2	41.6	0.9	0
4Oa15	16.2	16.6	0.4	40.2	-1.4	0.34
4Oa16	16.6	16.8	0.2	41.5	1.3	0
4Oa17	16.8	17	0.2	40.4	-1.1	0
4Oa18	17	17.3	0.3	41.8	1.4	0.6
4Oa19	17.3	20	2.7	39.8	-2.0	0.8
4Oa20	20	20.3	0.3	41.5	1.7	0.59
4Oa21	20.3	20.6	0.3	39.2	-2.2	0.99
4Oa22	20.6	21.3	0.7	42.4	3.2	1.67
4Oa23	21.3	22	0.7	47.9	5.5	1.67
4Oa24	22	22.7	0.7	45.2	-2.7	0.93
4Oa25	22.7	23	0.3	47.8	2.7	0.25
4Oa26	23	24.4	1.4	43.2	-4.7	1.11
4Oa27	24.4	24.6	0.2	40.3	-2.8	0
4Oa28	24.6	29.3	4.7	43.2	2.9	0.99
4Oa29	29.3	29.5	0.2	50.0	6.8	0
4Oa30	29.5	29.7	0.2	44.1	-5.9	0
4Oa31	29.7	30.6	0.9	40.9	-3.2	1.19
4Oa32	30.6	31.3	0.7	41.9	1.0	0.87
4Oa33	31.3	31.7	0.4	40.5	-1.4	0.54
4Oa34	31.7	31.9	0.2	41.9	1.4	0
4Oa35	32	32.3	0.3	40.11275	-1.8	1.13
4Oa36	32.3	34.1	1.8	43.2	3.0	1.21
4Oa37	34.1	34.3	0.2	46.8	3.6	0
4Oa38	34.3	37	2.7	43.1	-3.7	0.76
4Oa39	37	37.1	0.1	48.3	5.2	0
4Oa40	37.3	37.4	0.1	44.9	-3.4	0
4Oa41	38.8	38.9	0.1	47.9	3.0	0
4Oa42	39.3	39.8	0.5	45.0	-2.9	1.14
4Oa43	39.8	40.3	0.5	50.3	5.3	4.42
4Oa44	40.3	40.7	0.4	45.0	-5.3	0.7
4Oa45	40.7	42.1	1.4	49.3	4.3	2.45
4Oa46	42.1	42.6	0.5	44.7	-4.7	1.15
4Oa47	42.6	43	0.4	50.5	5.9	3.24
4Oa48	43	43.5	0.5	45.3	-5.2	2.49
4Oa49	43.5	44.9	1.4	40.8	-4.5	1.86
4Oa50	44.9	47.4	2.5	42.9	2.1	1.27
4Oa51	47.4	47.6	0.2	40.1	-2.9	0
4Oa52	47.6	53.9	6.3	43.2	3.1	1.22
4Oa53	53.9	54.6	0.7	48.9	5.7	2.5
4Oa54	54.6	55.3	0.7	45.8	-3.1	0.77
4Oa55	55.3	55.6	0.3	47.7	1.9	1.48
4Oa56	55.6	57	1.4	43.2	-4.5	0.91
4Oa57	57	57.5	0.5	48.8	5.7	1.5
4Oa58	57.5	57.7	0.2	45.6	-3.2	0
4Oa59	57.7	58.2	0.5	48.7	3.1	1.77

4Oa60	58.2	58.4	0.2	43.1	-5.7	0
4Oa61	58.4	59	0.6	50.0	7.0	2
5Oa1	0	0.6	0.6	47.9		1.02
5Oa2	0.6	0.8	0.2	42.3	-5.6	0
5Oa3	0.8	1	0.2	47.0	4.6	0
5Oa4	1	2.4	1.4	44.1	-2.9	1.97
5Oa5	2.4	3	0.6	41.2	-2.9	0.65
5Oa6	3	4.1	1.1	41.0	-0.3	0.61
5Oa7	4.1	5.3	1.2	42.1	1.1	1.09
5Oa8	5.3	5.5	0.2	47.0	4.9	0
5Oa9	5.5	7.4	1.9	43.2	-3.7	1.73
5Oa10	7.4	7.9	0.5	48.6	5.4	4.03
5Oa11	7.9	8.1	0.2	45.2	-3.4	0
5Oa12	8.1	8.8	0.7	50.2	4.9	1.88
5Oa13	8.8	10.5	1.7	42.8	-7.4	1.68
5Oa14	10.5	10.7	0.2	40.1	-2.7	0
5Oa15	10.7	10.9	0.2	43.2	3.1	0
5Oa16	10.9	11.2	0.3	40.5	-2.7	0.23
5Oa17	11.2	11.8	0.6	41.4	0.9	0.5
5Oa18	11.8	13.7	1.9	39.8	-1.6	1.04
5Oa19	13.7	17.4	3.7	43.7	4.0	1.81
5Oa20	17.4	17.6	0.2	39.8	-3.9	0
5Oa21	17.6	19	1.4	43.7	3.9	1.08
5Oa22	19	21	2	48.1	4.4	1.65
5Oa23	21	22.9	1.9	42.7	-5.4	1.28
5Oa24	22.9	23.2	0.3	47.8	5.1	0.49
5Oa25	23.2	24.6	1.4	43.8	-4.1	1.7
6Oa1	0	0.8	0.8	40.8		0.82
6Oa2	0.8	2.4	1.6	42.6	1.8	0.98
6Oa3	2.4	3.5	1.1	40.7	-2.0	0.82
6Oa4	3.5	4.1	0.6	42.7	2.0	0.54
6Oa5	4.1	4.7	0.6	40.1	-2.6	0.65
6Oa6	4.7	7.9	3.2	41.8	1.7	0.76
6Oa7	7.9	8.1	0.2	40.7	-1.2	0
6Oa8	8.1	8.4	0.3	42.6	2.0	0.22
6Oa9	8.4	10.4	2	49.0	6.3	2.33
6Oa10	10.4	10.9	0.5	46.6	-2.3	3.16
6Oa11	10.9	11.2	0.3	48.6	2.0	2.02
6Oa12	11.2	11.4	0.2	45.1	-3.5	0
6Oa13	11.4	11.8	0.4	48.5	3.4	0.99
6Oa14	11.8	14.4	2.6	42.8	-5.7	0.91
6Oa15	14.4	15.9	1.5	47.1	4.3	0.95
6Oa16	15.9	16.1	0.2	45.7	-1.4	0
6Oa17	16.1	16.3	0.2	51.7	6.0	0
7Oa1	2.6	3.5	0.9	39.9		0.62
7Oa2	16.7	17	0.3	39.9	0.0	0.27
7Oa3	4.2	4.4	0.2	39.9	0.0	0
7Oa4	28	28.2	0.2	40.4	0.5	0
7Oa5	38.1	38.3	0.2	40.6	0.3	0

7Oa6	24.8	25.7	0.9	40.7	0.0	0.53
7Oa7	38.6	39.3	0.7	40.7	0.1	0.58
7Oa8	36.5	37.2	0.7	40.8	0.0	0.44
7Oa9	4.8	5.3	0.5	40.9	0.1	0.19
7Oa10	29.5	30.3	0.8	40.9	0.1	0.33
7Oa11	3.5	4.2	0.7	41.6	0.7	0.67
7Oa12	4.4	4.8	0.4	41.7	0.1	0.06
7Oa13	39.3	40.1	0.8	41.9	0.2	1.09
7Oa14	1.1	2.6	1.5	42.1	0.1	1.6
7Oa15	8.6	11.9	3.3	42.5	0.4	1.22
7Oa16	22.5	24.8	2.3	42.5	0.0	1.18
7Oa17	30.3	31.8	1.5	42.6	0.0	0.97
7Oa18	25.7	28	2.3	42.8	0.2	1.44
7Oa19	38.3	38.6	0.3	43.0	0.2	1.26
7Oa20	15.9	16.7	0.8	43.1	0.1	1.24
7Oa21	37.2	38.1	0.9	43.2	0.1	1.66
7Oa22	28.2	29.5	1.3	43.2	0.0	1.6
7Oa23	34.3	36.5	2.2	43.5	0.3	1.71
7Oa24	18.7	22.3	3.6	43.7	0.2	1.66
7Oa25	5.3	7.8	2.5	43.9	0.3	1.78
7Oa26	32	33.2	1.2	44.0	0.1	0.68
7Oa27	13.3	13.6	0.3	44.3	0.3	0.88
7Oa28	0	0.9	0.9	44.5	0.2	0.84
7Oa29	17	18.4	1.4	44.7	0.2	1.56
7Oa30	14.5	15.2	0.7	45.1	0.4	0.44
7Oa31	0.9	1.1	0.2	46.6	1.4	0
7Oa32	18.4	18.7	0.3	46.7	0.1	0.14
7Oa33	22.3	22.5	0.2	46.8	0.2	0
7Oa34	31.8	32	0.2	47.8	0.9	0
7Oa35	7.8	8.6	0.8	48.1	0.3	1.61
7Oa36	13.6	14.5	0.9	48.1	0.1	1.72
7Oa37	11.9	13.3	1.4	48.4	0.3	2.14
7Oa38	15.2	15.9	0.7	48.5	0.1	2.37
7Oa39	33.2	34.3	1.1	49.7	1.2	1.25
10Oa1	0	1.9	1.9	42.5		0.46
10Oa2	1.9	3.1	1.2	40.5	-2.1	1.42
10Oa3	3.1	5.7	2.6	47.3	6.9	1.27
10Oa4	5.7	6.4	0.7	50.0	2.7	1.68
10Oa5	6.4	7.4	1	43.2	-6.8	3.81
10Oa6	7.4	9.4	2	49.4	6.2	2.41
10Oa7	9.4	11.3	1.9	43.5	-5.9	1.28
11Oa1	0	1.3	1.3	43.2		1.49
11Oa2	1.3	1.6	0.3	48.1	4.9	2.06
11Oa3	1.6	2.1	0.5	45.3	-2.8	0.56
11Oa4	2.1	2.7	0.6	47.7	2.4	2.05
11Oa5	2.7	5.2	2.5	43.3	-4.4	1.6
11Oa6	5.2	5.9	0.7	49.3	6.0	2.77
11Oa7	5.9	6.4	0.5	45.6	-3.6	3.5
11Oa8	6.4	6.8	0.4	48.5	2.9	1.44

12Oa1	0	2.6	2.6	42.6		1.51
12Oa2	2.6	2.9	0.3	46.2	3.6	0.21
12Oa3	2.9	3.2	0.3	44.0	-2.3	0.48
12Oa4	3.2	3.8	0.6	48.3	4.4	2.15
12Oa5	3.8	4.3	0.5	44.2	-4.1	1.1
12Oa6	4.3	4.5	0.2	48.1	3.9	0
12Oa7	4.5	5.1	0.6	43.4	-4.7	0.66
12Oa8	5.1	5.4	0.3	47.0	3.7	0.98
12Oa9	5.4	7	1.6	42.1	-4.9	1.23
12Oa10	7	7.2	0.2	47.2	5.0	0
12Oa11	7.2	8.9	1.7	43.4	-3.8	1.65
12Oa12	8.9	10.1	1.2	40.1	-3.2	0.69
12Oa13	10.1	10.7	0.6	43.6	3.5	1.24
12Oa14	10.7	11.2	0.5	41.2	-2.5	0.57
12Oa15	11.2	11.5	0.3	44.0	2.8	0.96
12Oa16	11.5	13.3	1.8	40.0	-4.0	1.13
12Oa17	13.3	13.5	0.2	44.2	4.2	0
12Oa18	13.5	15.1	1.6	40.0	-4.2	1.09
12Oa19	15.1	15.9	0.8	41.7	1.7	0.66
14Oa1	0	0.9	0.9	44.8		1.26
14Oa2	0.9	1.1	0.2	49.7	4.9	0
14Oa3	1.1	1.3	0.2	45.2	-4.5	0
14Oa4	1.3	1.9	0.6	47.9	2.7	2.24
14Oa5	1.9	2.7	0.8	43.3	-4.7	1.46
15Oa1	0	1.2	1.2	52.3		2.46
15Oa2	1.2	1.4	0.2	45.0	-7.3	0
15Oa3	1.4	1.8	0.4	48.0	2.9	1
15Oa4	1.8	3.8	2	43.4	-4.6	1.18
17Oa1	0	1.4	1.4	49.2		3.24
18Oa1	0	0.3	0.3	46.9		0
18Oa2	0.3	0.5	0.2	45.4	-1.6	0
18Oa3	0.5	1.4	0.9	46.5	1.2	0.61
18Oa4	1.4	2.2	0.8	41.9	-4.6	0.72
18Oa5	2.2	2.7	0.5	40.3	-1.6	0.55
18Oa6	2.7	3.1	0.4	41.6	1.4	0.84
18Oa7	3.1	3.4	0.3	40.2	-1.4	0.41
18Oa8	3.4	4.9	1.5	42.3	2.1	0.49
18Oa9	4.9	5.1	0.2	46.4	4.1	0
18Oa10	5.1	5.5	0.4	45.5	-1.0	1.74
18Oa11	5.5	5.7	0.2	47.4	1.9	0
18Oa12	5.7	6.7	1	42.9	-4.5	2.91
20Oa1	0	0.3	0.3	46.7		0
20Oa2	0.3	1	0.7	44.3	-2.3	1
20Oa3	1	1.2	0.2	46.7	2.4	0
20Oa4	1.2	1.4	0.2	55.3	8.6	0
20Oa5	1.4	1.9	0.5	48.3	-7.1	1.45

X1Oa1	0	0.3	0.3	47.1		0
X1Oa2	0.3	1.4	1.1	45.7	-1.4	1
X1Oa3	1.4	1.6	0.2	47.6	2.0	0
X1Oa4	1.6	1.9	0.3	44.6	-3.0	1.28
X1Oa5	1.9	2.2	0.3	46.6	2.0	0.98
X1Oa6	2.2	2.4	0.2	38.6	-8.0	0
X1Oa7	2.4	5	2.6	43.6	5.0	1.29
X1Oa8	5	7.9	2.9	38.8	-4.8	0.88
X1Oa9	7.9	8.1	0.2	42.6	3.8	0
X1Oa10	8.1	9.5	1.4	39.0	-3.6	0.91
X1Oa11	9.5	10.6	1.1	43.3	4.3	1.75
X1Oa12	10.6	11.2	0.6	39.6	-3.6	0.88
X1Oa13	11.2	12.2	1	42.7	3.1	1.45
X1Oa14	12.2	13	0.8	40.5	-2.2	0.94
X1Oa15	13	13.5	0.5	46.5	6.0	2.03
X1Oa16	13.5	15.6	2.1	43.1	-3.4	1.56
X1Oa17	15.6	16.1	0.5	49.7	6.6	1.97
X1Oa18	16.1	16.7	0.6	53.2	3.6	1.02
X1Oa19	16.7	18.4	1.7	43.2	-10.0	1.45
X1Oa20	18.4	18.8	0.4	46.2	3.0	0.33
X1Oa21	18.8	19.8	1	45.5	-0.7	0.98
X1Oa22	19.8	20.5	0.7	49.0	3.5	2.34
X1Oa23	20.5	23.7	3.2	43.0	-6.0	1.01
X1Oa24	23.7	26	2.3	40.3	-2.7	1.03
X1Oa25	26	26.4	0.4	42.5	2.3	0.88
X1Oa26	26.4	28.7	2.3	40.5	-2.0	0.86
X1Oa27	28.7	29.6	0.9	42.2	1.7	1.17
X1Oa28	29.6	30.1	0.5	40.5	-1.7	1.66
X1Oa29	30.1	30.5	0.4	47.2	6.6	1.24
X1Oa30	30.5	30.8	0.3	43.8	-3.4	1.39
X1Oa31	30.8	31	0.2	47.0	3.2	0
X1Oa32	31	33.1	2.1	42.9	-4.1	1.8
X1Oa33	33.1	33.3	0.2	40.7	-2.1	0
X1Oa34	33.3	34.7	1.4	43.2	2.4	2.56
X1Oa35	34.7	35	0.3	47.1	4.0	1.05
X1Oa36	35	35.2	0.2	44.9	-2.3	0
X1Oa37	35.2	35.4	0.2	47.2	2.3	0
X1Oa38	35.4	37	1.6	42.8	-4.4	1.49
X1Oa39	37	37.4	0.4	49.4	6.6	1.85
X1Oa40	37.4	39.4	2	44.2	-5.2	0.97
X1Oa41	39.4	39.6	0.2	46.5	2.3	0
X1Oa42	39.6	42.3	2.7	43.6	-2.9	1.55
X1Oa43	42.3	44.9	2.6	40.0	-3.6	0.63
X1Oa44	44.9	45.6	0.7	44.8	4.8	2.68
X2Oa1	0	1.5	1.5	40.7		0.71
X2Oa2	1.5	3.2	1.7	42.7	2.0	1.81
X2Oa3	3.2	4.2	1	47.0	4.2	1.87
X2Oa4	4.2	4.6	0.4	43.2	-3.8	1.02
X2Oa5	4.6	5.1	0.5	40.0	-3.2	0.69
X2Oa6	5.1	5.7	0.6	44.2	4.2	1.71

X3Oa1	0	0.4	0.4	40.0		0.17
X3Oa2	0.4	0.7	0.3	42.8	2.7	1.74
X3Oa3	0.7	1.1	0.4	40.1	-2.7	0.55
X3Oa4	1.1	2.4	1.3	42.8	2.7	1.68
X3Oa5	2.4	2.6	0.2	46.5	3.7	0
X3Oa6	2.6	3	0.4	42.0	-4.5	0.62
X3Oa7	3	3.2	0.2	40.6	-1.4	0
X3Oa8	3.2	5	1.8	42.7	2.1	1.49
X3Oa9	5	5.8	0.8	47.8	5.1	1.04
X3Oa10	5.8	6	0.2	45.7	-2.1	0
X5Oa1	0	0.2	0.2	41.8		0
X5Oa2	0.2	3.5	3.3	40.0	-1.8	0.7
X5Oa3	3.5	3.8	0.3	41.5	1.5	0.57
X5Oa4	3.8	4.4	0.6	40.0	-1.6	1.5
X5Oa5	4.4	4.7	0.3	42.0	2.0	0.48
X5Oa6	4.7	4.9	0.2	39.5	-2.5	0
X5Oa7	4.9	5.4	0.5	42.0	2.5	1.29
X5Oa8	5.4	5.9	0.5	40.2	-1.7	0.88
X5Oa9	5.9	6.2	0.3	42.0	1.8	0.9
X5Oa10	6.2	6.7	0.5	40.6	-1.4	0.37
X5Oa11	6.7	9	2.3	42.1	1.5	1.1
X5Oa12	9	10.2	1.2	40.3	-1.8	0.45
X5Oa13	10.2	11	0.8	42.5	2.1	1.2
X5Oa14	11	11.2	0.2	40.0	-2.5	0
X5Oa15	11.2	12.6	1.4	41.0	1.0	0.53
X5Oa16	12.6	15	2.4	43.7	2.7	1.26
X5Oa17	15	15.7	0.7	49.5	5.8	3.08
X5Oa18	15.7	17.9	2.2	43.4	-6.1	1.36
X5Oa19	17.9	18.6	0.7	50.7	7.3	4.14
X5Oa20	18.6	20.4	1.8	44.0	-6.6	1.2
X5Oa21	20.4	21.4	1	51.1	7.0	3.31
X5Oa22	21.4	27.5	6.1	42.9	-8.2	1.71
X5Oa23	27.5	27.8	0.3	49.3	6.4	1.04
